# Comprehensive Survey and Comparative Assessment of RNA-Binding Residue Predictions with Analysis by RNA Type

**DOI:** 10.3390/ijms21186879

**Published:** 2020-09-19

**Authors:** Kui Wang, Gang Hu, Zhonghua Wu, Hong Su, Jianyi Yang, Lukasz Kurgan

**Affiliations:** 1 School of Mathematical Sciences and LPMC, Nankai University, Tianjin 300071, China; wangkui@nankai.edu.cn (K.W.); wuzhh@nankai.edu.cn (Z.W.); xd07121026@126.com (H.S.); yangjy@nankai.edu.cn (J.Y.); 2 School of Statistics and Data Science, LPMC and KLMDASR, Nankai University, Tianjin 300071, China; huggs@nankai.edu.cn; 3 Department of Computer Science, Virginia Commonwealth University, Richmond, VA 23284, USA

**Keywords:** RNA-binding residues, protein-RNA interactions, ribosomal RNA, transfer RNA, small nuclear RNA, messenger RNA, signal recognition particle, protein-DNA interactions, benchmark, predictive performance

## Abstract

With close to 30 sequence-based predictors of RNA-binding residues (RBRs), this comparative survey aims to help with understanding and selection of the appropriate tools. We discuss past reviews on this topic, survey a comprehensive collection of predictors, and comparatively assess six representative methods. We provide a novel and well-designed benchmark dataset and we are the first to report and compare protein-level and datasets-level results, and to contextualize performance to specific types of RNAs. The methods considered here are well-cited and rely on machine learning algorithms on occasion combined with homology-based prediction. Empirical tests reveal that they provide relatively accurate predictions. Virtually all methods perform well for the proteins that interact with rRNAs, some generate accurate predictions for mRNAs, snRNA, SRP and IRES, while proteins that bind tRNAs are predicted poorly. Moreover, except for DRNApred, they confuse DNA and RNA-binding residues. None of the six methods consistently outperforms the others when tested on individual proteins. This variable and complementary protein-level performance suggests that users should not rely on applying just the single best dataset-level predictor. We recommend that future work should focus on the development of approaches that facilitate protein-level selection of accurate predictors and the consensus-based prediction of RBRs.

## 1. Introduction

Proteins interact with many types of RNAs including ribosomal RNA (rRNA), transfer RNA (tRNA), messenger RNA (mRNA), internal ribosome entry site RNA (IRES; specialized type of structured mRNAs often found in RNA viruses that is involved in ribosomal translation), small nuclear RNA (snRNA), microRNA (miRNA), signal recognition particle RNA (SRP), among others [1,2]. These interactions are crucial for a wide spectrum of cellular functions, such as protein synthesis, posttranscriptional regulation and regulation of gene expression [3,4,5]. Recent works associate some of the RNA-binding proteins with human diseases, from neurodegenerative and cardiovascular disorders to cancer [6,7,8,9,10]. While many experimental methods are currently used to characterize the RNA-binding proteins [11], these approaches do not keep up with the rapid accumulation of protein sequence data. The UniProt database has 180.7 million proteins, including 41.5 million in the eukaryotic organisms (data as of 11 June, 2020) [12]. Only about 10 thousand eukaryotic proteins are manually annotated to bind RNA, while another 650 thousand are predicted to interact with RNA based on sequence similarity [12]. Even if we combine these annotations and predictions, they account for only 1.6% of the eukaryotic proteins while the number of the RNA-binding proteins is approximated to range between 2% and 8% across the eukaryotic organisms [5], with as many as 8% of human proteins identified to interact with RNAs and another 16% predicted to bind RNAs [13]. Computational approaches offer an opportunity to mitigate this knowledge gap.

Many computational models that predict protein-RNA interactions from the protein sequence or structure have been published in the past quindecennial [14,15,16,17,18,19,20,21]. We also note recent efforts to predict protein binding nucleotides in the RNA sequences [22,23,24]. There are many more methods that make these predictions on the protein side. They are implemented at three distinct levels of resolutions [16]. At the lowest, whole-protein resolution level, they predict whether a given protein binds RNA, without further details about this interaction. At the medium-resolution level, they predict residues in the protein sequence that bind RNA, while at the highest resolution level they model these interactions at the atomic scale using the three-dimensional structures of protein and RNA. The ability to use these computational tools depends on the availability of the corresponding protein data. The highest resolution structure-based methods are limited to a relatively small collection of proteins that have three-dimensional structures. As of June 2020, the worldwide database of protein structures, Protein Data Bank (PDB) [25], provides access to 169,000 protein structures. While a high-quality predicted structure can be also used, this inadvertently reduces the quality of the protein-RNA interaction predictions and still significantly limits the coverage. For example, protein structures can be accurately predicted for only about 28% of human proteins [26]. On the other hand, the medium- and low-resolution predictors rely solely on the protein sequences that are available for over 180 million of proteins. Our focus is on the medium resolution methods since they can be applied on the millions of the currently available protein sequences and since they offer more detailed insights about the interactions compared to the low-resolution approaches.

Table 1 summarizes recent reviews that cover the sequence-based predictors of RNA-binding residues (RBRs) [14,15,16,17,18,19,20]. These surveys provide useful insights about the availability, features, and predictive performance of the medium-resolution predictors, assisting users in the understanding and selection of suitable tools. Nearly all out of the 30 published predictors of RBRs are partner agnostic, which means that they use the protein sequence as the only input and do not consider characteristics of the RNA partner [15,16,19,20]. According to a recent survey [19], there are only two sequence-based partner-specific predictors of RBRs: PRIdictor [27] and PS-PRIP [28]. These two methods use sequences of both protein and RNA as the input. However, this survey reveals that the partner-specific predictors are outperformed by modern partner-agnostic methods [19], which is why we concentrate on the latter category of predictors. The surveys listed in Table 1 summarize between seven and 18 partner-agnostic sequence-based predictors of RBRs. They also perform empirical comparative analysis that covers between three and eight tools. Most of these reviews investigate the issue of the cross-predictions between RNA and DNA interactions, which we also consider in this study. More specifically, several studies found that predictors of RBRs often predict DNA-binding residues as RBRs and vice versa [15,16,20,29]. Table 1 also identifies several limitations of the past surveys. They miss some of the available predictors, their empirical analysis does not consider specific types of RNA, and they exclusively rely on the dataset-level quantification of the predictive performance. We implement four innovative features to produce a comprehensive survey that addresses these drawbacks. First, we improve the coverage by discussing a comprehensive collection of 28 partner-agnostic predictors. Second, we are the first to evaluate predictive performance for several specific RNA types, besides the commonly assessed overall performance that is insensitive to the RNA types. As part of this effort, we release the first benchmark dataset that annotates RBRs to specific RNA types. Third, we analyze predictive performance at the commonly used dataset level as well as at the protein level. The past studies, including several surveys [14,15,16,17,18,19,20] and comparative analyses that accompany publication of the individual predictors of RBRs [29,30,31,32,33,34,35,36,37,38,39,40,41,42,43,44,45], assess the predictive quality by summarizing it on a dataset of proteins, rather than for individual proteins. This is an important drawback since, arguably, these predictors are used more often to identify RBRs for individual proteins rather than for datasets of hundreds of proteins. For instance, our DRNApred predictor [29] was recently used to predict RBRs in the human brain expressed x-linked protein 3 (hBEX3) [46], the ankyrin repeat domain-55 (ANKRD55) [47], and in a few proteins from the Japanese Encephalitis Virus [48]. Specific levels of performance on benchmark datasets, which can be gleaned from prior studies, do not guarantee that the same quality should be expected for individual proteins. A recent analysis of the protein-level performance for the prediction of the intrinsic disorder indeed shows a substantial variability of the protein-level performance [49]. Motivated by these results, we are the first to analyze differences in the quality of the predictions of RBRs across proteins and compare them to the corresponding dataset-level results. Fourth, we investigate the impact of the sequence similarity on the predictive quality for a few predictors that rely on homology modelling. Our empirical analysis reveals that these methods produce relatively accurate predictions and thus we analyze whether these advantages are driven by the similarity of their template datasets to the benchmark proteins that we employ. The comprehensive nature and the broad range of novel aspects tackled in this survey allow us to produce several unique insights that facilitate better understanding and selection of the tools for the partner-agnostic protein-sequence based prediction of RBRs.

## 2. Survey of Partner-Agnostic Sequence-Based Predictors of RNA-Binding Residues

We analyze past surveys [14,15,16,17,18,19,20] and perform a comprehensive Pubmed search to produce a list of 28 partner-agnostic sequence-based predictors of RBRs [29,30,31,32,33,34,35,36,37,38,39,40,41,42,43,44,45]. We summarize these methods in Table 2. The first predictor was published in 2004 by a group from the University of Tokyo [50]. This predictive model relies on a simple three-layer feedforward neural network that uses the protein sequence and the sequence-predicted secondary structure as the only inputs. The next two predictors, BindN [51] and RNABindR [52], are developed by the Liangjiang Wang’s group and the labs of Vasant Honavar and Drena Dobbs, respectively. The first group went on to release an upgraded version of this method, BindN+ [53], in 2010. Similarly, the Honavar and Dobbs labs continued the development of these predictors with two subsequent methods, RNABindRPlus [39] in 2014 and FastRNABindR [35] in 2016. 

Table 2 reveals that the peak of the development efforts stretches between 2008 and 2011 when half of the 28 methods were published. On average two predictors are released in the last few years. Virtually all surveyed predictors utilize machine learning algorithms to produce their predictive models. The only exception is SPOT-Seq-RNA that performs predictions via homology, i.e., by transferring annotations of RBRs from a similar protein found in its dataset of templates, i.e., proteins with known structure in complex with an RNA molecule. The machine learning models cover a broad range of algorithms including by far the most popular support vector machines (14 out of 28 predictors), neural networks (five predictors), decision trees including random forests (five predictors), and several other less popular options, such as logistic regression and naïve Bayes. In a few recent cases, including aaRNA, SNBRFinder, and NucBind, the results of the machine learning model are combined with the prediction derived via homology transfer from the dataset of templates.

According to Google Scholar (as of 9 June 2020), the 28 predictors are collectively cited 2110 times, which corresponds to an impressive average rate of 75 citations per method. The annual citations counts, which accommodate for the differences in the number of years of use, reveal that the most cited predictors are BindN (30 citations per year), iDeepE (23), PPrint (21), DRNApred (17), BindN+ (17), and RNABindR (15). We note that several tools, particularly among those released recently, predict both RNA and DNA-binding residues. They include BindN [51], BindN+ [53], DRNApred [29], NucBind [31], and SNBRFinder [37]. This may explain higher citation numbers for these methods.

Analysis of Table 2 shows that 22 out of the 28 methods were originally available to the end user via a webserver (17 predictors) or at least as a standalone software (five predictors). The six methods that were published without offering neither option suffer a poor uptake, as reflected by their average number of just four citations per year. To compare, the predictors that were made available are cited on average 10 times per year. Similarly, the six methods were published in venues with low impact factor (average of 3.2), compared to the predictors that offer webservers (average of 5.9). Unfortunately, many of the methods that were originally available are no longer supported and the corresponding websites are down. In particular, only seven out of the 17 webservers (41%) were available to us when we performed the empirical study in 2019. A similar observation is made in the 2013 survey [16]. The lack of the ongoing post-publication support is a serious concern, as this deflates confidence and trust among the end user community.

## 3. Materials and Methods

Our empirical study is motivated by the shortcomings of the past comparative surveys discussed in the introduction. We evaluate and compare predictive quality for a selected set of currently available predictors on a novel benchmark dataset. The key characteristics of our study are that: (1) we assess the predictive performance at both protein-level and dataset-level; (2) we evaluate the performance for several specific RNA types, besides the typically done overall evaluation across all RNA types; and (3) we study the impact of the similarity between the benchmark proteins and the proteins in the template datasets used by the predictors that utilize homology modelling module.

### 3.1. Selection of Partner-Agnostic Sequence-Based Predictors of RBRs

The empirical study compares six carefully selected, publicly available and diverse predictors of RBRs. Inspired by recent prior studies [14,15], we focus on the methods that have functioning webservers and that were published in the last 10 years (Table 2). The methods that are available solely as a standalone software depend on third party software to generate inputs, which is often no longer available, making it impossible to obtain predictions. Using these criteria we select six predictors highlighted in bold font in Table 2: NucBind [31], DRNApred [29], FastRNABindR [35], aaRNA [38], RNABindRPlus [39], and BindN+ [53]. These methods rely on a diverse set of predictive models including support vector machines (NucBind, FastRNABindR, RNABindRPlus and BindN+), logistic regression (DRNApred), and neural networks (aaRNA). Moreover, they include methods that utilize homology modelling (NucBind, aaRNA, and RNABindRPlus) and approaches which are capable of predicting both RNA and DNA binding residues (NucBind and DRNApred). This selection allows us to address the abovementioned three key characteristics. Finally, these methods remain available to the end users. More specifically, NucBind, DRNApred, FastRNABindR, aaRNA, and RNABindRPlus were available at the time of submission (August 2020) via the websites listed in Table 2. Although the BindN+’s webserver is no longer supported, the authors provide a standalone version upon request by email.

### 3.2. Benchmark Dataset

We collect experimental annotations of RBRs from BioLip [61]. This weekly updated database provides access to a complete set of high-quality curated annotations of protein-ligand interactions extracted from PDB, i.e., in our case from the structurally solved protein-RNA complexes. After parsing the BioLip data, we found 3988 RNAs bound to 1222 unique proteins (UniProt accession numbers). We remove data for 667 short RNAs fragments (sequence length < 10) since we would not be able to identify the corresponding RNA type for these ligands. We map the remaining 3321 RNAs that are associated with 1678 PDB structures to RNAcentral, the largest centralized resource that combines RNA data coming from 41 smaller databases [1], to identify the RNA types. We use PDB IDs to map data in RNAcentral for 3282 RNAs and we identify RNA types for 1154 of them. We utilize the structural data in PDB to process the unresolved set of 3321 − 3282 = 39 proteins and we identify the RNA type in 21 cases. In total, we annotate RNA type for 1154 + 21 = 1175 RNAs. They include 754 ribosomal RNAs (rRNAs), 249 transfer RNAs (tRNAs), 37 small nuclear RNAs (snRNAs), 36 messenger RNAs (mRNAs), 36 riboswitch RNAs, 32 ribozyme RNAs, 15 signal recognition particle RNAs (SRPs), six internal ribosome entry site RNAs (IRESs), five transfer-messenger RNAs (tmRNAs), three small conditional RNAs (scRNAs), one antitoxin RNA, and one microRNA (miRNA). Mapping these RNAs to the corresponding proteins reveals that we annotate RNA type for 754 out of the 1222 RNA-binding proteins that we originally collected.

The next step is to ensure that the RNA-binding proteins that we use in the empirical analysis are non-redundant (we do not use multiple similar RNA-binding proteins, as this would bias the results toward this family of proteins) and dissimilar to the training datasets of the six predictors that we assess. We collect the training datasets from NucBind, DRNApred, aaRNA, FastRNABindR, nd BindN+, which total to 1511 unique sequences. Next, we cluster these proteins with our set of 1222 RNA-binding proteins at 30% similarity using BlastClust. We remove all proteins that are in clusters with any of the training proteins. This ensures that the proteins in the remaining clusters share low, <30%, similarity to the training proteins. Next, we represent each of the remaining clusters with one protein to make sure that the selected benchmark proteins are non-redundant. We select the protein with the highest number of annotated RBRs if all proteins in a given cluster bind the same type of RNA, or the protein that interacts with the largest number of different RNAs that are underrepresented in the dataset if the cluster includes proteins that bind multiple RNA types. We also remove the RNA types for which number of binding residues is insufficient to perform a reliable statistical analysis, i.e., the number of the corresponding RBRs < 30. As a result, we obtain a set of 150 RNA-binding proteins with the annotated RNA types. They have 3500 rRNA-binding residues, 442 tRNA-binding residues, 306 snRNA-binding residues, 54 SRP-binding residues, 44 mRNA-binding residues, and 37 IRES-binding residues.

In the final step, we supplement the set of the RNA-binding proteins with the proteins that do not interact with RNAs. This allows us to quantify the amount of the false positive predictions in the proteins that do not bind RNA, which constitute significant errors. We consider two distinct types of false positives, the cross-predictions (predictions of RBRs among the DNA-binding proteins) and over-predictions (predictions of RBRs among the proteins that do not interact with the nucleic acids). We collect a set of proteins that do not interact with the nucleic acids from SWISS-PROT, the manually curated part of UniProt. First, we remove peptides (chains shorter than 50 residues) and proteins that include the “RNA”, “DNA”, “nucle”, and “ribosom” keywords. Next, we cluster the remaining set of proteins together with the above RNA-binding proteins using BlastClust at 30% similarity. We select at random 75 clusters that do not have any RNA-binding proteins and represent each of these clusters with one protein, for the total of 75 non-nucleic acids binding proteins. We collect the DNA-binding proteins from BioLip. We cluster the corresponding 9829 DNA-binding proteins together with the above RNA-binding proteins at 30% using BlastClust. Next, we select at random 75 clusters that exclude RNA-binding proteins. We pick one protein from each of these clusters to obtain the set of 75 DNA-binding proteins. Altogether, the benchmark dataset includes 300 proteins, with 150 RNA-binding and 150 non-RNA-binding proteins, where the latter set is divided into two equal-size subsets of proteins that interact with DNA and that do not interact with the nucleic acids.

We emphasize that this careful design process results in four key benefits. First, the benchmark dataset provides a balanced sampling of the RNA-binding proteins. It broadly covers the taxonomic space with 70% eukaryotic, 22% bacterial, 4% viral, and 4% archaeal proteins. Moreover, the dataset uniformly samples the protein space because of the BlastClust clustering that we perform early in the process. We further investigate this aspect based on analysis of the Pfam domains [62] that we extract from the RNA-binding proteins. We find 155 unique domains where 77% of them that appear once and with only three most frequent domains which appear just four times. This supports the claim that proteins included in our dataset uniformly sample the space of the RNA-binding proteins. Second, the benchmark proteins share low similarity (<30%) with the training datasets used to develop the six selected predictors. This ensures that the assessment is fair across the considered predictors (none of these tools was an advantage of being developed using similar proteins) and that these proteins cannot be accurately predicted using sequence alignment to the training proteins. Recent surveys use a more relaxed criteria where they limit the benchmark proteins based on their date of deposition, i.e., they use proteins that were released after the date where the corresponding training datasets are collected [14,15,16,19,20]. Third, the availability of the experimental annotations allows us to analyze predictive performance for specific types of RNAs. Fourth, inclusion of the DNA-binding proteins and proteins that do not interact with the nucleic acids facilitates assessment of the amount of the cross-predictions and over-predictions, respectively. Several studies investigate the cross-predictions and point to the fact that some predictors that generate high amounts of the cross-predictions are effectively incapable to differentiate between RNA- and DNA-binding residues [15,16,20,29]. We expand these studies by testing more recent predictors and by comparing the rates of cross-predictions with the rates of the over-predictions. We provide the complete benchmark dataset, together with the annotation of the RBRs and the corresponding RNA type, in the Supplement.

### 3.3. Assessment of Predictive Performance

The partner-agnostic sequence-based predictors of RBRs generate two types of outputs for every residue in the input protein sequence: the real-valued putative propensity for RNA binding and the binary prediction (RNA-binding vs. non-RNA-binding). The binary predictions are typically generated from the propensities such that the amino acids with propensities above a predictor-specific threshold are predicted as RBRs, while the remaining residues are assumed not to bind RNA.

We assess the predictive quality for the binary predictions with several metrics that were utilized in the recent surveys [14,15,16,19,20]:

$$sensitivity=\frac{\mathrm{TP}}{\mathrm{TP}+\mathrm{FN}}$$

$$specificity=\frac{\mathrm{TN}}{\mathrm{TN}+\mathrm{FP}}$$

$$F1=\frac{2*\mathrm{TP}}{2*\mathrm{TP}+\mathrm{FP}+\mathrm{FN}}$$

$$\mathrm{Matthews}’s\;\mathrm{correlation}\;\mathrm{coefficient}\;\left(\mathrm{MCC}\right)=\frac{\mathrm{TP}*\mathrm{TN}\;+\;\mathrm{FP}*\mathrm{FN}}{\sqrt{\left(\mathrm{TP}\;+\;\mathrm{FP}\right)*\left(\mathrm{TP}\;+\;\mathrm{FN}\right)*\left(\mathrm{TN}\;+\;\mathrm{FP}\right)*\left(\mathrm{TN}\;+\;\mathrm{FN}\right)}\;}$$

where TP and TN denote the number of the correctly predicted RBRs and non-RBRs, respectively; FP denotes the number of the non-RBRs predicted as RBRs; and FN is the number of RBRs predicted as non-RBRs. The sensitivity and specificity quantify the rates of correct predictions among the experimentally annotated RBRs and experimentally annotated non-RBRs, respectively. F1 is harmonic mean of sensitivity and precision and ranges between 0 (when no true positives are predicted) and 1 (when no incorrect predictions are generated). MCC is a correlation between the experimental and putative binary annotations, for which value of 0 is equivalent to a random predictor and value of 1 denotes a prefect prediction. The predictors that we consider use different ways to establish the binary prediction threshold, which results in vastly dissimilar rates of the predicted RBRs for the same proteins. This renders side-by-side comparisons of the predictive performance unreliable, i.e., one should not compare the binary metrics between a tool that predicts majority of the residues as RBRs (high sensitivity coupled with low specificity) and another method that predicts only a small fraction of residues as RBRs (low sensitivity coupled with high specificity). Following recent comparative studies [29,63,64], we equalize the binary predictions between the six considered predictors by selecting the threshold that generates the correct number of RBRs, i.e., the number of the predicted RBRs is equal to the number of the experimentally annotated RBRs. With this setup we can direct compare predictive performance between the six considered predictors using a well-defined prediction rate.

We apply a commonly used AUC (area under the receiver operator characteristic curve) to measure the quality of putative propensities. The curve plots TPR = TP/(TP+FN) against FPR = FP/(FP+TN). We compute TPR and FPR by binarizing the propensities with thresholds equal to all unique values of the propensities. Since the benchmark dataset is unbalanced (a small minority of residues bind RNA) and following studies that use similarly unbalanced datasets [14,63,64,65,66], we also measure AULC (area under the low false positive rate receiver operator characteristic curve). The AULC values quantify the area under the curve where the number of predicted RBRs ≤ number of experimentally annotated RBRs, i.e., where the predictors do not over-predict RBRs and the corresponding FPR is relatively low. Since AULC values are rather small, we normalize them by dividing the measured AULC by the AULC of a random predictor. AULCratio = 1 correspond to the predictions that are equivalent to a random prediction while AULCratio > 1 gives the rate of improvement over the random predictor.

## 4. Predictive Performance of Partner-Agnostic Sequence-Based Predictors of RBRs

### 4.1. Prediction of RBRs Measured at the Dataset-Level

We summarize evaluation of the six representative partner-agnostic sequence-based methods for the prediction of RBRs in the top row in Table 3. We provide the corresponding ROC curves in Supplementary Figure S1A. We assess statistical significance of differences between the results generated by the overall best predictor, RNABindRPlus, and each of the other five predictors. This test investigates whether the differences would hold over different datasets. We perform 10 repetitions of stratified random sampling of half the benchmark proteins. For normal data (we test normality with the Kolmogorov–Smirnov test) we use the paired t-test; otherwise we use the Wilcoxon signed rank test.

The best performing RNABindRPlus generates AUC = 0.87, AULCratio = 26, and MCC = 0.41. These results are significantly better than the predictions produced by the other five methods (*p*-value < 0.01), except for the AUC of aaRNA where the difference is not statistically significant. A recent comparative article that includes RNABindRPlus similarly finds that this predictor consistently outperforms several other methods [19]. The RNABindRPlus’s AULCratio reveals that this tool improves by 26-fold over a random predictor when applied to make predictions with low false positive rates (i.e., when setup not to over-predict RBRs). We emphasize that the other methods (with the exception of DRNApred) also provide very accurate predictions, with the AULCratio values ranging between 10.3 (for BindN+) and 17.8 (for aaRNA). Using the binary predictions that are normalized to predict the correct number of RBRs (the threshold is setup to ensure that the number of predicted and experimentally annotated RBRs is equal), we find that the current methods secure sensitivity values between 13.2% (DRNApred) and 44.4% (RNABindRPlus), which are coupled with specificity at about 97%.

**Figure 1:**
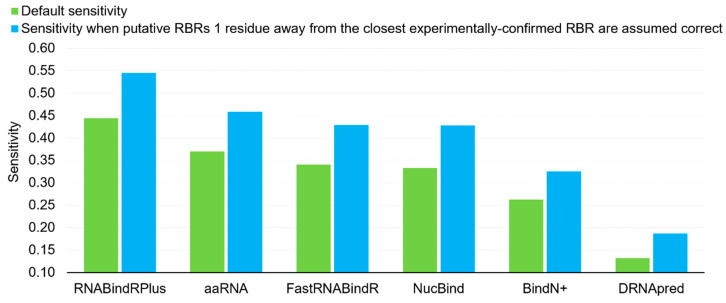
Comparison of the default sensitivity (green bars) and the sensitivity when putative RBRs that are immediate neighbors (one residue away in the sequence) of the experimentally annotated RBRs are assumed correct (blue bars).

Furthermore, we investigate impact of proximity in the sequence on the sensitivity. The false positives (incorrectly predicted RBRs) localized as immediate neighbors of the experimentally annotated RBRs could be considered as useful predictions. We argue that they provide useful clues for the location of the RBRs and they could be even considered as correct predictions since the definition of the RBRs depends on a somehow arbitrary atomic distance. In Figure 1 we compare the default sensitivity with the sensitivity when putative RBRs that are one residue away in the sequence from the experimentally-confirmed RBRs (immediate neighbors) are assumed correct. This analysis reveals that the sensitivity of the six predictors increases by a substantial margin if we assume that the predictions for the immediate sequence neighbors are correct, from 0.13 to 0.19 for the worst-performing DRNApred, and from 0.44 to 0.55 for the best-performing RNABindRPlus. The average increase, across the six predictors, equals 0.09. Interestingly, this result demonstrates that majority of the putative RBRs generated by RNABindRPlus are within one position of the experimentally annotated RBRs. Overall, we find that the currently available methods provide accurate predictions of RBRs, with RNABindRPlus being the best option.

### 4.2. Prediction of RBRs for Specific RNA Types Measured at the Dataset-Level

We summarize the results when the predictions are applied to identify RBRs that interact with specific RNA types including rRNA, tRNA, snRNA, mRNA, SRP, and IRES in Table 3. We give the corresponding ROC curves in Supplementary Figure S1B (for rRNA), S1C (mRNA), S1D (snRNA), S1E (SRP), S1F (IRES), and S1G (tRNA). Statistical tests follow the procedure described in Section 4.1.

The results suggest that the overall performance for the prediction of RBRs shown in the first row of Table 1 is primarily driven by the strong predictions for the rRNA-binding residues, which constitute 80% of the RBRs in the benchmark set. RNABindRPlus provides the most accurate and significantly better predictions for rRNA when compared to the other five predictors (*p*-value < 0.01), except for aaRNA’s AUC which is still lower but not significantly. The RNABindRPlus’s AUC = 0.89, AULCratio = 30, MCC = 0.44, and F1 = 0.46. Four other methods, which include aaRNA, BindN+, FastRNABindR, and NucBind, also provide very accurate predictions for rRNAs. Their AULCratio values span from 12 (for BindN+) to 20.5 (for aaRNA), and AUCs range between 0.79 (NucBind) and 0.87 (aaRNA). The results for the mRNA, snRNA, SRP and IRES RNAs are mixed, with some accurate and some poorly performing methods. The mRNAs are predicted well by NucBind (AUC = 0.84 and AULCratio = 10.6), FastRNABindR (AUC = 0.81 and AULCratio = 7.8) and BindN+ (AUC = 0.80 and AULCratio = 7.2). Although RNABindRPlus secures the top AUC = 0.87, it does relatively poorly when predicting mRNAs with low FPRs (AULCratio = 2.3). This is visible for the low FPR values in Supplementary Figure S1C where RNABindRPlus’s ROC curve underperforms, particularly when compared to aaRNA and NucBind. The snRNAs and SRPs are predicted accurately only by RNABindRPlus and aaRNA. These methods secure both high AUCs > 0.77 and high AULCratio values > 7. The IRES RNAs can be accurately predicted by three methods, RNABindRPlus, aaRNA and DRNApred (AUCs > 0.8 and AULCratio values > 7). Finally, predictions for tRNAs suffer relatively low performance across all methods. AULCratio values vary between 1.8 (DRNApred) and 5.4 (RNABindRPlus) and AUCs between 0.69 (BindN+) and 0.75 (NucBind).

To summarize, our first-of-its-kind analysis that considers RNA types shows that the quality of the predictions varies widely between specific types of RNAs. While high quality predictions for rRNA are produced with virtually all tools, the predictions for mRNAs, snRNA, SRP, and IRES RNAs vary in quality, with only a few tools providing strong predictive performance. Furthermore, none of the six evaluated here methods provides accurate results for the tRNAs. This analysis suggests that the users should tailor the selection of the predictor to the type of RNA that is expected to interact with their protein of interest.

### 4.3. Cross-Prediction and Over-Prediction of RBRs

Motivated by prior studies [15,16,20], we quantify the amount of the cross-predictions (RBRs predicted in the DNA-binding proteins) and over-predictions (RBRs predicted in the proteins that do not interact with RNA). Statistical tests follow the protocol explained in Section 4.1. We summarize results in Table 4. Our analysis reveals that the methods considered here incorrectly predict between 1.1% (for RNABindRPlus) and 2.6% (BindN+) of RBRs among the proteins that do not interact with RNA. Moreover, these rates go up to between 1.7% (RNABindRPlus) and 3.2% (BindN+) for the proteins that interact with DNA. We normalize these prediction rates by the rate of prediction of RBRs among the RNA-binding proteins. We note that the overall rate of the predictions of RBRs is matched for all predictors and set to equal to the rate of the experimentally annotated RBRs. This ensures that the results can be reliably compared between predictors. The ratioRNA/DNA values, defined as the rate of the prediction of RBRs in RNA-binding proteins divided by rate for the DNA-binding proteins (higher values are better), reveal that the best-performing DRNApred predicts 6.6 times more RBRs among the RNA-binding proteins. This ratio is significantly better (*p*-value < 0.01) than the ratios of the other five methods that vary from 2.1 (BindN+) to 6.0 (RNABindRPlus). Moreover, values of ratioRNA/non-RNA, defined as the rate of the prediction of RBRs in the RNA-binding proteins to the rate for the non-RNA-binding proteins (higher values are better), show that five of the six methods provide statistically equivalent ratios ranging between 4.4 (for aaRNA) and 9.6 (for RNABindRPlus), with BindN+ that secures a significantly lower ratio of 2.5 (*p*-value < 0.01 compared to DRNApred). Overall, these results agree with prior studies that similarly suggest that current partner-agnostic sequence-based predictors of RBRs have difficulty differentiating RNA and DNA-binding [15,16,20]. We find that DRNApred is the only tool that solves this problem (Table 4), however, at the cost of the lower overall predictive performance (Table 3). This is in line with the original motivation for DRNApred, which specially aims to improve separation between the prediction of residues that interact with DNA vs. RNA [29].

### 4.4. Prediction of RBRs Measured at the Protein-Level

**Figure 2:**
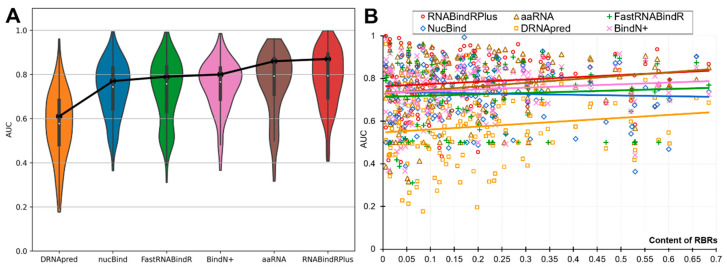
Protein-level predictive performance measured with AUC for the six partner-agnostic sequence-based predictors of RBRs on the benchmark dataset. This analysis focuses on the RNA-binding proteins as the calculation of the per-protein AUC is not possible for the other proteins. The violin plots in Panel (**A**) represent the distributions of the per-protein AUC values. The box plots inside the violin plots represent the first quartile (bottom of the box), the second quartile/median (white dot) and the third quartile (top of the box) for these distributions. The black points connected by the black solid lines denote the dataset-level AUC values. Panel (**B**) shows relation between per-protein AUC and the content of RBRs (fraction of RBR in the protein chain). The color-coded solid lines correspond to the linear fit between the content and the AUC values for a given predictor.

The partner-agnostic sequence-based predictors of RBRs are often used to predict individual proteins, while the past assessments quantify the performance on datasets of proteins [14,15,16,17,18,19,20]. We analyze the per-protein performance to investigate whether and to what degree it varies from the dataset-level assessments. In Figure 2 we show the protein-level AUCs across all RNA-binding proteins (panel B) and as distributions (panel A). Figure 2A reveals that each of the six predictors produces a wide range of the per-protein AUCs. Even the best-performing predictors predict a substantial number of proteins poorly (AUC < 0.65), with 17% of such weak predictions for the best-performing RNABindRPlus, 20% for aaRNA, and 19% for the popular BindN+. On the other hand, equally substantial numbers of proteins are predicted very accurately (AUC > 0.85), including 35% for RNABindRPlus, 31% for aaRNA, and 17% for BindN+. The median per-protein AUCs are 0.58 for DRNApred, 0.74 for NucBind, 0.76 for FastRNABindR, 0.77 for BindN+, 0.79 for aaRNA and RNABindRPlus. To compare, the dataset-level AUCs (Table 3) are 0.61, 0.78, 0.79, 0.80, 0.85, and 0.87, respectively. This reveals that on average the users should expect that the protein-level performance is lower than the values produced at the dataset level suggest.

**Figure 3:**
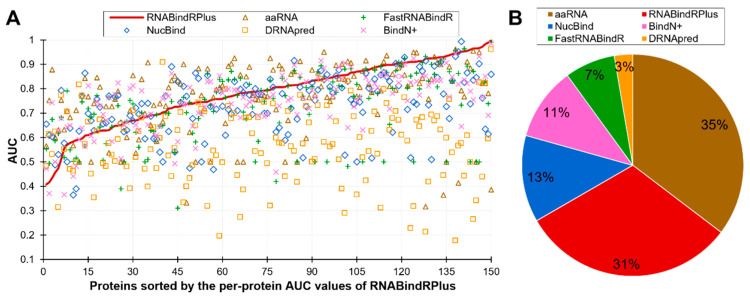
Complementarity of the six partner-agnostic sequence-based predictors of RBRs. This analysis focuses on the RNA-binding proteins as the calculation of the per-protein AUC is not possible for the other proteins. Panel (**A**) shows the per-proteins AUC values for proteins sorted by the AUCs of the best performing RNABindRPlus that are represented by the red line. Panel (**B**) shows the fractions of the RNA-binding proteins for which a given predictor secures the highest value of AUC. Predictors are sorted in descending order by the value of the fraction.

Figure 2B is a scatter plot that shows the diversity of the per-protein AUCs across the predictors and analyzes relation between these values and the content of RBRs (fraction of RBRs in a given protein). The scatter confirms that each predictor offers both excellent and poor predictions and also reveals that the predictive performance does not depend on the content of RBRs. The Pearson correlations between the content and AUC values range from −0.04 (for NucBind) to 0.18 (for aaRNA). The diversity of the AUC values across methods and proteins prompt us to take a closer look at the complementarity of the per-protein predictions across the six methods. We summarize this analysis is Figure 3. Figure 3A sorts the proteins according to the per-protein AUC of the best performing RNABindRPlus, shown as the red line. AUCs of the other five methods are often located above the red line, which means that they outperform RNABindRPlus for many of the benchmark proteins, even when RNABindRPlus generates accurate predictions (per-protein AUC > 0.85). Figure 3B visualizes the fractions of the proteins for which a given predictor secures the highest value of the per-protein AUC. Interestingly, aaRNA outperforms RNABindRPlus by securing the best AUC for 35% of the proteins. Moreover, each of the six predictors, even including the worst performing DRNApred, outclasses all other methods for some proteins. This suggests that the six predictors provide different and complementary predictions, which can be explained by the fact that they use different training datasets and different predictive models (Table 2). Our analysis further suggests that building consensus methods, which combine predictions generated by multiple methods, could lead to improvements in the predictive performance. We simulate an oracle approach that always selects the most accurate predictor across the six method for a given benchmark protein. Such predictor produces median per-protein AUC of 0.86 (compared to 0.79 for the currently best method) and generates only 3% of poor-quality predictions (AUC < 0.65). To sum up, we show that none of the six predictors outperforms the other methods when tested on individual proteins. Each method produces both very accurate and rather poor predictions. The predictions of the considered here six methods complement each other and could be collectively used to produce accurate consensus predictors.

### 4.5. Impact of Similarity to the Template Datasets

Three of the six predictors use homology transfer to make predictions, namely NucBind [31], aaRNA [38], and RNABindRPlus [39] (Table 2). Their predictive models include two parts, a search against a database of templates (proteins that have experimental annotations of RBRs) and a machine learning predictor. They combine predictions generated by transferring the RBR from sufficiently similar templates with the ab initio predictions from the machine learning predictor in order to produce putative RBRs. While we specifically design the benchmark dataset to share low similarity to the training datasets used to build the machine learning predictors, the templates could be similar to the benchmark proteins. In the case of NucBind, we modify the template database to remove the proteins that share >30% with the benchmark dataset, ensuring that we can apply the full set of the benchmark proteins. This option was not available for aaRNA and RNABindRPlus. Removal of the benchmark proteins that share >30% similarity with the template datasets of these two predictors collectively shrinks the benchmark set to only a couple dozen RNA-binding proteins. Instead, we analyze the impact of the similarity to the templates on the predictive performance for aaRNA and RNABindRPlus by evaluating their predictions for subsets of the benchmark that share specific range of sequence similarity. We consider four intervals of similarity: below 30%, 30–50%, 50–80%, and over 80%. We summarize these results in Table 5.

We perform two comparisons. First, we compare the predictions from aaRNA (top of the Table 5) and from RNABindRPlus (bottom of Table 5) for the benchmark proteins that share low (<30%) similarity to their templates against the benchmark proteins that share higher levels of similarity to the templates of the same predictor; we show these results in Table 5 using bold font. We provide the corresponding ROC curves in the Supplementary Figure S2. The analysis reveals that aaRNA is sensitive to the similarity between template and benchmark proteins. Its results drop to AUC = 0.66 and MCC = 0.27 for the low similarity benchmark proteins, compared to AUC > 0.83 and MCC > 0.34 when higher similarity is shared. Using statistical tests described in Section 4.1, we find that the differences between the results on the <30% similarity subset and each of the higher-similarity subsets are statistically significant for both AUC and MCC (*p*-value < 0.05). This suggests that aaRNA heavily relies on the homology transfer to secure high-quality predictions. In contrast, we discover that the differences for RNABindRPlus are smaller in magnitude, AUC = 0.84 for the low similarity benchmark proteins vs. AUCs between 0.88 and 0.90 for the higher similarity proteins, and that some of these differences lack statistical significance. This means that the machine learning predictor for this method provides accurate results.

The second comparison analyzes differences between aaRNA (top of Table 5) or RNABindRPlus (bottom of Table 5) and the other five predictors on the proteins that share the same range of similarity. We investigate whether the differences observed on the complete benchmark dataset (Table 3) are consistent with the results when the similarity to the templates is factored in (Table 5). The results in Table 5 for aaRNA show that when tested on benchmark proteins dissimilar to its templates (similarity < 30%), its performance (AUC = 0.66) becomes significantly worse (*p*-value < 0.05) than the results of RNABindRPlus (AUC = 0.85) and FastRNABindR (AUC = 0.83) and worse but not significantly than two other methods, BindN+ (AUC = 0.76) and NucBind (AUC = 0.77). This is in contrast to Table 3 where aaRNA outperforms three of these methods (FastRNABindR, BindN+, and NucBind). This demonstrates that the drop in the aaRNA’s performance for the low similarity proteins results is so substantial that other predictors overtake its results. Furthermore, Table 3 reveals that RNABindRPlus outperforms all other methods on the complete benchmark set. Table 5 confirms this result and shows that RNABindRPlus again significantly outperforms the other five predictors (*p*-value < 0.05), with the only exception of aaRNA where the AUC of RNABindRPlus is higher but the difference is not significant. However, this exception can be explained by the use of the homology transfer by aaRNA for these proteins.

In the nutshell, we show that RNABindRPlus provides robust and high-quality predictions, as it outperforms the other five methods irrespective of the similarity between its templates and the benchmark proteins. On the other hand, we demonstrate that aaRNA relies on the homology transfer and its machine learning predictor underperforms when applied to the benchmark proteins that share low similarity with the templates.

## 5. Conclusions

We survey close to 30 sequence-based predictors of RBRs. We find that this field has entered a mature stage, with on average two new methods released annually, after the spike in the late 2000s where 14 predictors were developed in the span of just four years. The current predictors primarily rely on machine learning models, which in some cases are combined with the homology transfer from template datasets. We expose a major flaw related to the lack of support for the webservers and implementation of these methods after the publication. The availability of the webservers and implementations is limited to only a handful of the predictors.

We perform empirical assessment of predictive performance for a representative set of six methods using a novel benchmark dataset that features low similarity to the training datasets of the six predictors and annotates types of the interacting RNA molecules. We produce several interesting and novel observations. We find that the six methods provide useful predictions of RBRs. Furthermore, the most accurate predictor, RNABindRPlus, significantly outperforms the other five tools, both on the complete benchmark dataset and on the set of benchmark proteins that share low similarity to the temples that this method employs. This contrasts with the other homology-transfer based methods, aaRNA, which underperforms when applied to the proteins sharing low similarity with its templates. Analysis that considers performance for specific types of RNAs reveals that virtually all methods produce accurate predictions for rRNA. On the other hand, the predictions for mRNAs, snRNA, SRP and IRES RNAs vary in quality, with only a few tools producing accurate predictions. Finally, we show that predictions of the interactions with tRNAs suffer low quality across the six tools. Consequently, we suggest that the end users should alter the selection of the predictive tool to the type of RNA, if known. We also find that the current methods make major mistakes by predicting large numbers of RBRs in the proteins that do not interact with RNA, particularly in the DNA-binding proteins. Our result confirms findings of a few recent studies that these methods have a difficult time differentiating between RNA and DNA-binding [15,16,20]. We find that DRNApred is the only tool that accurately differentiates between interactions with the two nucleic acids, but at the cost of a lower overall predictive performance.

The protein-level analysis demonstrates that none of the six methods consistently outperforms the other tools when tested on individual proteins. We show that these methods produce both very accurate and very poor results, suggesting that the end users should not limit themselves to using only the most accurate tool. Instead, the selection should be tailored to the performance of a given method for a given protein. While we currently lack tool that would facilitate such selection, recent research in the context of the prediction of related intrinsically disordered residues [67,68,69] offers two options for the future development of suitable solutions. The first option is a quality assessment tool which generates residue-level scores that quantify likelihood that a given residue is accurately predicted by a given method [70,71]. These scores are used to identify poorly predicted proteins for a given predictor. The second option are methods that directly suggests the most accurate predictor for a given input protein sequence [72]. Use of these tools leads to a two-step prediction process where the users first select a well-performing predictor (using either option) and then use this specific tool to collect the predictions.

Moreover, the protein-level analysis demonstrates that the predictions of the six tools complement each other. This means that the best predictions for different proteins come from different predictors. This suggests that a consensus approach that combines predictions generated by multiple methods to generate results that outperform any of the individual tools should be possible to build. Feasibility of such consensus-based predictor is motivated by the success of the consensus methods for several related residue-level predictive tasks, such as the prediction of intrinsic disorder [73,74,75,76,77,78,79] and secondary structure [80,81,82].

## Figures and Tables

**Table 1:** Surveys of the sequence-based predictors of RBRs. While some of these surveys cover the structure-based methods and methods that consider protein-DNA interactions, we specifically focus on their coverage of the sequence-based predictors of RBRs.

Ref.	Year Released	No. of Predictors Surveyed	No. of Predictors Assessed Empirically	Evaluates or Analyzes			
				Cross-Prediction between RNA and DNA	Specific Types of RNAs	Protein-Level Performance and Complementarity	Dependence on Sequence Similarity For Homology-Based Predictions
This article		28	6	Yes	Yes	Yes	Yes
[19]	2019	9	6	No	No	No	No
[14]	2019	18	4	Yes	No	No	No
[15]	2016	16	3	Yes	No	No	No
[20]	2015	17	8	Yes	No	No	No
[16]	2013	10	8	Yes	No	No	No
[17]	2012	13	3	No	No	No	No
[18]	2012	7	7	No	No	No	No

**Table 2:** Partner-agnostic sequence-based predictors of RBRs.

Ref.	Name	Year Published	Model Type	Citations		Impact Factor	Availa-Bility	Webpage	Webserver Available at the Time of Analysis
				Total	Annual				
[30]	CNN model	2019	Convolutional NN	0	0	N/A	N	N/A	N/A
**[31]**	**NucBind**	**2019**	**SVM+HT**	**5**	**5**	**4.5**	**W**	**http://yanglab.nankai.edu.cn/NucBind/**	**yes**
[32]	iDeepE	2018	Convolutional NN	47	23	4.5	S	https://github.com/xypan1232/iDeepE/	N/A
**[29]**	**DRNApred**	**2017**	**Logistic regression**	**51**	**17**	**11.2**	**W**	**http://biomine.cs.vcu.edu/servers/DRNApred/**	**yes**
[33]	PredRBR	2017	Gradient boosted DT	28	13	2.5	S	http://dlab.org.cn/PredRBR/	N/A
[34]	DORAEMON	2017	Bayesian classifier	5	2	1.9	S	https://github.com/ABCgrp/DORAEMON/	N/A
**[35]**	**FastRNABindR**	**2016**	**SVM**	**9**	**2**	**2.8**	**W**	**http://ailab.ist.psu.edu/FastRNABindR/**	**yes**
[36]	RNAProSite	2016	RF	14	3	2.5	W	http://lilab.ecust.edu.cn/NABind/	no
[37]	SNBRFinder	2015	SVM+HT	13	3	2.8	W	http://ibi.hzau.edu.cn/SNBRFinder/	no
**[38]**	**aaRNA**	**2014**	**Feedforward NN+HT**	**31**	**5**	**11.2**	**W**	**http://sysimm.ifrec.osaka-u.ac.jp/aarna/**	**yes**
**[39]**	**RNABindRPlus**	**2014**	**SVM+HT**	**63**	**10**	**2.8**	**W**	**http://ailab1.ist.psu.edu/RNABindRPlus/**	**yes**
[40]	SRCpred	2011	Feedforward NN	34	4	2.5	W	http://tardis.nibio.go.jp/netasa/srcpred/	no
[41]	PredictRBP	2011	SVM	33	4	2.5	S	http://cic.scu.edu.cn/bioinformatics/Predict_RBP.rar	N/A
[42]	SVM model	2011	SVM	24	3	2.5	N	N/A	N/A
[43]	PRBR	2011	RF	62	7	2.5	W	http://www.cbi.seu.edu.cn/PRBR/	no
[44]	SPOT-Seq-RNA	2011	HT	52	6	5.5	W	http://sparks-lab.org/server/SPOT-Seq-RNA/	no
[45]	NAPS	2010	DT	64	6	11.2	W	http://proteomics.bioengr.uic.edu/NAPS/	no
[54]	RBRpred	2010	SVM	52	5	1.9	N	N/A	N/A
[55]	PRNA	2010	RF	134	13	4.5	S	http://www.aporc.org/doc/wiki/PRNA/	N/A
[56]	PiRaNhA	2010	SVM	69	7	11.2	W	http://www.bioinformatics.sussex.ac.uk/PIRANHA/	no
**[53]**	**BindN+**	**2010**	**SVM**	**168**	**17**	**2.1**	**W**	**http://bioinfo.ggc.org/bindn+/**	**yes**
[57]	ProteRNA	2010	SVM	22	2	3.5	N	N/A	N/A
[58]	Pprint	2008	SVM	247	21	2.5	W	http://crdd.osdd.net/raghava/pprint/	yes
[59]	PRINTR	2008	SVM	71	6	2.5	W	http://210.42.106.80/printr/	no
[60]	RNAProB	2008	SVM	119	10	2.5	N	N/A	N/A
[52]	RNABindR	2007	Naive Bayes	198	15	11.2	W	http://bindr.gdcb.iastate.edu/RNABindR/	no
[51]	BindN	2006	SVM	416	30	11.2	W	http://bioinformatics.ksu.edu/bindn/	no
[50]	NN model	2004	Feedforward NN	79	5	N/A	N	N/A	N/A

We describe the type of the model, which includes neural network (NNs), random forest (RF), support vector machine (SVM), decision tree (DT), and homology transfer (HT). The citations were collected from Google Scholar on 9 June 2020. The most recent impact factor was obtained from Clarivariate Analytics in June 2020; the impact factor is not available (N/A) for the two methods that were published in the conference proceedings. The availability is encoded as W, S and N if webserver, only standalone software, and neither the webserver nor code are available, respectively. Methods shown in bold font are used in the empirical comparative analysis performed in this survey.

**Table 3:** Predictive performance of the six partner-agnostic sequence-based predictors of RBRs on the benchmark dataset.

RNA Type	Predictor	AUC	AULCratio	MCC	F1	Sensitivity	Specificity
All RBRs	RNABindRPlus	**0.869**	**25.9**	**0.414**	**0.437**	**0.444**	**0.976**
	aaRNA	0.848^=^	17.8^+^	0.344^+^	0.370	0.370	0.974
	BindN+	0.803^+^	10.3^+^	0.233^+^	0.263	0.263	0.970
	FastRNABindR	0.792^+^	17.1^+^	0.312^+^	0.339	0.341	0.972
	NucBind	0.775^+^	16.0^+^	0.307^+^	0.335	0.333	0.973
	DRNApred	0.608^+^	4.1^+^	0.097^+^	0.132	0.132	0.964
rRNA	RNABindRPlus	**0.893**	**30.2**	**0.441**	**0.458**	**0.518**	**0.976**
	aaRNA	0.870^=^	20.5^+^	0.356^+^	0.377	0.418	0.974
	BindN+	0.829^+^	12.0^+^	0.246^+^	0.271	0.303	0.970
	FastRNABindR	0.820^+^	20.5^+^	0.334^+^	0.355	0.400	0.972
	NucBind	0.790^+^	18.7^+^	0.325^+^	0.347	0.385	0.973
	DRNApred	0.601^+^	4.5^+^	0.095^+^	0.126	0.141	0.964
mRNA	RNABindRPlus	**0.869**	2.3	0.009	0.003	0.091	**0.976**
	aaRNA	0.637^+^	**12.4^–^**	**0.034^–^**	**0.009**	**0.295**	0.974
	BindN+	0.798^+^	7.2^–^	0.020^–^	0.005	0.205	0.970
	FastRNABindR	0.814^+^	7.8^–^	0.025^–^	0.007	0.227	0.972
	NucBind	0.844^=^	10.6^–^	0.030^–^	0.008	0.273	0.973
	DRNApred	0.383^+^	4.0^=^	0.006^=^	0.002	0.091	0.964
snRNA	RNABindRPlus	**0.806**	**13.1**	**0.068**	**0.046**	**0.222**	**0.976**
	aaRNA	0.777^=^	8.6^=^	0.065^=^	0.043	**0.222**	0.974
	BindN+	0.716^+^	2.7^+^	0.018^+^	0.015	0.088	0.970
	FastRNABindR	0.769^+^	5.5^+^	0.040^+^	0.028	0.150	0.972
	NucBind	0.685^+^	5.9^+^	0.038^=^	0.027	0.144	0.973
	DRNApred	0.535^+^	0.9^+^	−0.002^+^	0.004	0.029	0.964
SRP	RNABindRPlus	0.774	**29.6**	**0.058**	**0.017**	0.426	**0.976**
	aaRNA	**0.880^=^**	7.8^+^	0.025^=^	0.008	0.204	0.974
	BindN+	0.625^+^	4.3^+^	0.013^+^	0.004	0.130	0.970
	FastRNABindR	0.288^+^	0.3^+^	−0.001^+^	0.001	0.019	0.972
	NucBind	0.608^+^	21.4^=^	0.037^=^	0.011	0.296	0.973
	DRNApred	0.543^+^	15.2^=^	0.051^=^	0.013	**0.463**	0.964
IRES	RNABindRPlus	0.818	7.3	0.023	0.006	0.216	**0.976**
	aaRNA	**0.921^–^**	8.5^=^	0.022^=^	0.006	0.216	0.974
	BindN+	0.729^+^	4.5^=^	0.008^+^	0.002	0.108	0.970
	FastRNABindR	0.758^+^	0.7^+^	0.000^+^	0.001	0.027	0.972
	NucBind	0.780^=^	0.7^+^	0.000^+^	0.001	0.027	0.973
	DRNApred	0.855^=^	**15.5^=^**	**0.031^=^**	**0.007**	**0.351**	0.964
tRNA	RNABindRPlus	0.745	**5.4**	0.029	0.027	0.095	**0.976**
	aaRNA	0.735^=^	5.1^=^	**0.043^=^**	**0.036**	**0.133**	0.974
	BindN+	0.689^+^	3.7^=^	0.030^=^	0.026	0.111	0.970
	FastRNABindR	0.739^=^	5.2^=^	0.040^=^	0.033	0.131	0.972
	NucBind	**0.751^=^**	3.1^=^	0.025^=^	0.024	0.090	0.973
	DRNApred	0.742^=^	1.8^=^	0.016^=^	0.017	0.084	0.964

Tests for a specific RNA type include RBRs that bind this RNA type and the non-RNA-binding residues; residues that interact with the other RNA types are excluded. The rate of the binary predictions was equalized between predictors such that the numbers of the predicted and the experimentally annotated RBRs are equal, allowing for side-by-side comparison of the binary metrics. The predictors are sorted in the order of their AUCs when tested using all RBRs. =/+/– summarize results of statistical tests and denote that the difference is not significant (*p*-value > 0.01)/that RNABindRPlus is significantly better (*p*-value ≤ 0.01)/that RNABindRPlus is significantly worse (*p*-value ≤ 0.01). The best results for each test are shown in bold font.

**Table 4:** Assessment of the over-predictions and cross-predictions for the six partner-agnostic sequence-based predictors of RBRs on the benchmark dataset.

Predictor	PPR on RNA-Binding Proteins	PPR on DNA-Binding Proteins	RatioRNA/DNA	PPR on Non-RNA Binding Proteins	RatioRNA/Non-RNA
DRNApred	0.084	**0.013**	**6.6**	0.018	**4.6**
RNABindRPlus	**0.103 ^=^**	0.017^+^	6.0^+^	**0.011 ^=^**	9.6^=^
NucBind	0.083 ^=^	0.019^+^	4.3^+^	0.018 ^=^	4.5^=^
aaRNA	0.083 ^=^	0.026^+^	3.2^+^	0.019 ^=^	4.4^=^
FastRNABindR	0.084 ^=^	0.028^+^	3.1^+^	0.019 ^=^	4.5^=^
BindN+	0.067 ^+^	0.032^+^	2.1^+^	0.026 ^+^	2.5^+^

The over-predictions (cross-predictions) are quantified with predictive positive rate (PPR) defined as the number of putative RBRs divided by the number of all residues in the subset of the benchmark set that covers 150 proteins that do not bind RNA (75 proteins that interact with DNA). Higher PPR values in these two datasets indicate worse predictions since these values correspond to false positive rates. We also give PPR on the set of 150 RNA-binding proteins, ratioRNA/DNA = PPR for the RNA-binding proteins divided by PPR for the DNA-binding proteins (higher value is better), and ratioRNA/non-RNA = PPR for the DNA-binding proteins divided by the PPR for the non-RNA-binding proteins (higher value is better). The binary predictions of RBRs were equalized between predictors such that the numbers of the predicted and the experimentally annotated RBRs on the benchmark dataset are equal, allowing for side-by-side comparison of the PPR and ratio metrics. The predictors are sorted by their ratioRNA/DNA values. =/+/– summarize results of statistical tests and denote that the difference is not significant (*p*-value > 0.01)/that DRNApred is significantly better (*p*-value ≤ 0.01)/that DRNApred is significantly worse (*p*-value ≤ 0.01). The best results for each test are shown in bold font.

**Table 5:** Predictive performance of the six partner-agnostic sequence-based predictors of RBRs on the subsets of the benchmark set that share pre-defined levels of similarity to the templates of aaRNA (top of the table) and RNABindRPlus (bottom of the table).

Benchmark Proteins Sharing a Given Range of Similarity to Templates of aaRNA	AUC						MCC					
	aaRNA	RNABindRPlus	BindN +	FastRNABindR	NucBind	DRNApred	aaRNA	RNABindRPlus	BindN +	FastRNABindR	NucBind	DRNApred
Below 30%	**0.66**	0.85 ^–^	0.76 ^=^	0.83 ^–^	0.77 ^=^	0.65 ^=^	**0.27**	0.33 ^–^	0.18 ^+^	0.22 ^=^	0.25 ^=^	0.10 ^+^
30–50%	**0.83**	0.92 ^–^	0.86 ^=^	0.90 ^–^	0.85 ^=^	0.72 ^+^	**0.36**	0.45 ^–^	0.22 ^+^	0.40 ^–^	0.31 ^+^	0.25 ^+^
50–80%	**0.90**	0.86 ^+^	0.78 ^+^	0.83 ^+^	0.77 ^+^	0.66 ^+^	**0.36**	0.37 ^=^	0.19 ^+^	0.28 ^+^	0.21 ^+^	0.10 ^+^
Above 80%	**0.86**	0.86 ^=^	0.81 ^+^	0.75 ^+^	0.78 ^+^	0.56 ^+^	**0.34**	0.42 ^–^	0.26 ^+^	0.32 ^=^	0.35 ^=^	0.09 ^+^
**Benchmark proteins sharing a given range of similarity to templates of RNABindRPlus**	**RNABindRPlus**	**aaRNA**	**BindN +**	**FastRNABindR**	**NucBind**	**DRNApred**	**RNABindRPlus**	**aaRNA**	**BindN**+	**FastRNABindR**	**NucBind**	**DRNApred**
Below 30%	**0.84**	0.82 ^=^	0.79 ^+^	0.74 ^+^	0.80 ^+^	0.57 ^+^	**0.29**	0.32 ^–^	0.18 ^+^	0.20 ^+^	0.22 ^+^	0.06 ^+^
30–50%	**0.90**	0.86 ^+^	0.83 ^+^	0.86 ^+^	0.80 ^+^	0.62 ^+^	**0.49**	0.35 ^+^	0.30 ^+^	0.38 ^+^	0.42 ^+^	0.10 ^+^
50–80%	**0.88**	0.82 ^+^	0.79 ^+^	0.78 ^+^	0.67 ^+^	0.42 ^+^	**0.47**	0.33 ^+^	0.24 ^+^	0.35 ^+^	0.31 ^+^	−0.10 ^+^
Above 80%	**0.89**	0.84 ^+^	0.80 ^+^	0.85 ^+^	0.75 ^+^	0.67 ^+^	**0.62**	0.37 ^+^	0.31 ^+^	0.54 ^+^	0.43 ^+^	0.20 ^+^

The rate of the binary predictions was equalized between predictors such that the numbers of the predicted and the experimentally annotated RBRs are equal, allowing for side-by-side comparison of MCCs. We summarize significance of differences between results generated by aaRNA/RNABindRPlus and each of the other five predictors for the set of proteins that share the same level of similarity; =/+/– denote that the difference between aaRNA/RNABindRPlus and another predictor for the set of proteins that share the same level of similarity is not significant (*p*-value > 0.05)/that aaRNA/RNABindRPlus is significantly better (*p*-value ≤ 0.05)/that aaRNA/RNABindRPlus is significantly worse (*p*-value ≤ 0.05). Comparison of the predictions from aaRNA and RNABindRPlus for the benchmark proteins that share <30% similarity to their templates against the benchmark proteins that share higher levels of similarity to the templates of the same predictor and shown in bold font.

## Supplementary Materials

The following are available online at https://www.mdpi.com/1422-0067/21/18/6879/s1, Figure S1: ROC curves for the six representative partner-agnostic sequence-based predictors of RBRs, Figure S2: ROC curves for the two partner-agnostic sequence-based predictors of RBRs that apply template proteins, aaRNA (panel A) and RNABindRPlus (panel B), and benchmark dataset.
