# Supplementary material for: Comprehensive Survey and Comparative Assessment of RNA-Binding Residue Predictions with Analysis by RNA Type
Source: Int J Mol Sci. 2020 Sep 19;21(18):6879. doi: 10.3390/ijms21186879 (PMC7554811; doi:10.3390/ijms21186879)
Supplement: Supplementary file 1 [file ijms-21-06879-s001.pdf]

Supplement for "Comprehensive survey and comparative assessment of RNA-binding residue predictions with the analysis by RNA type"

Supplementary Figures

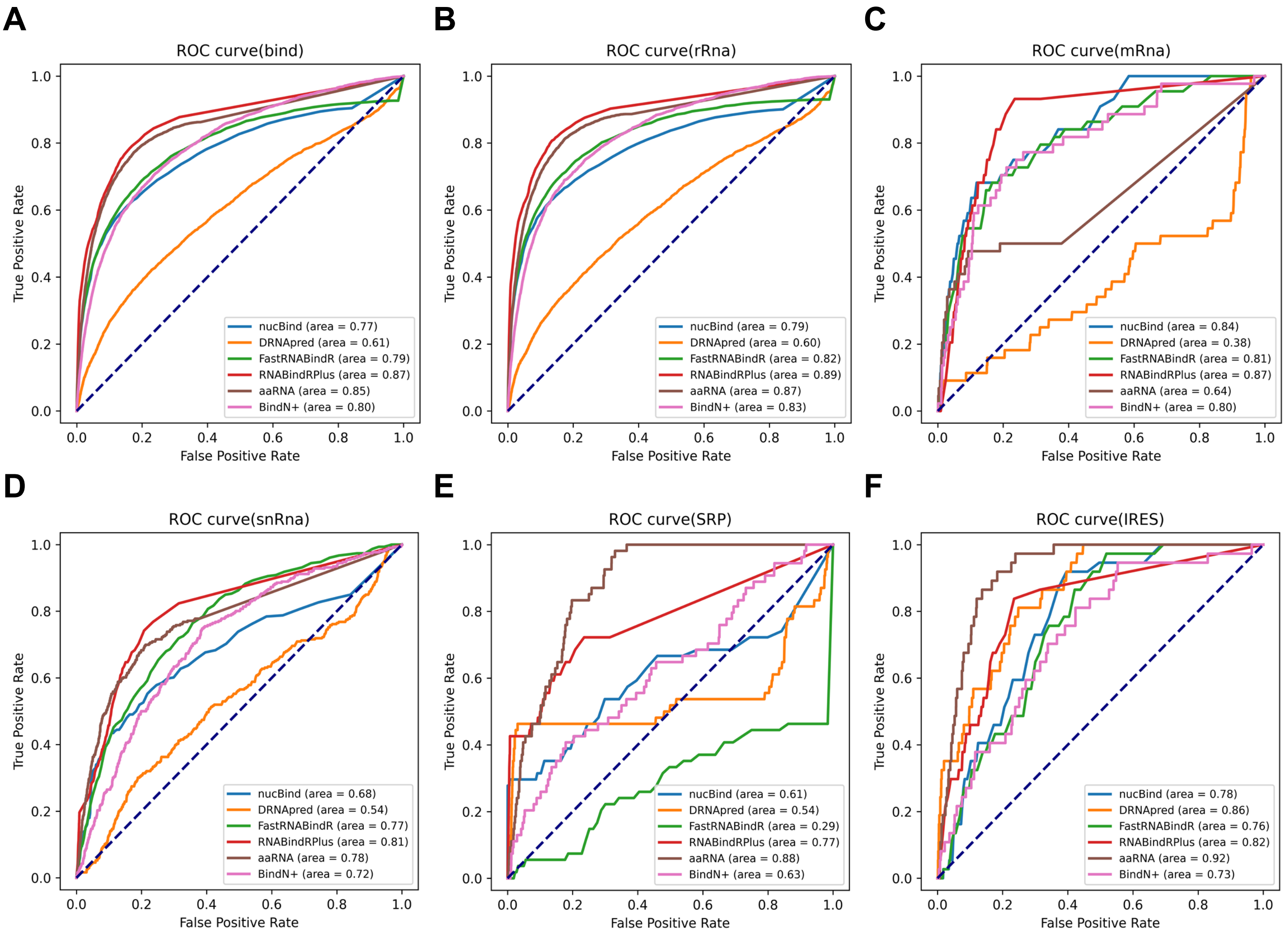

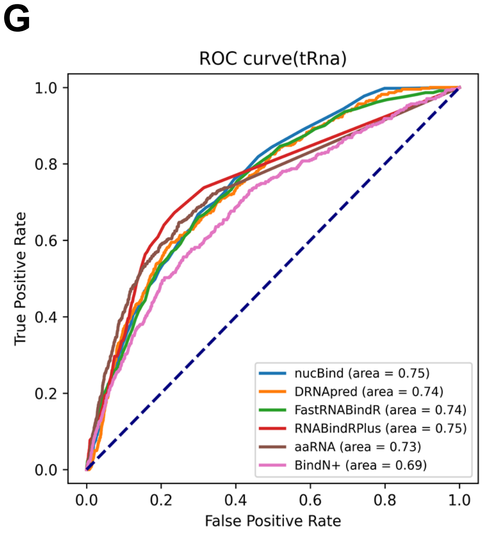

Supplementary Figure S1. ROC curves for the six representative partner-agnostic sequence-based predictors of RBRs. Panels A, B, C, D, E, F and G correspond to the results across all RBRs, rRNAs, mRNAs, snRNAs, SRP, IRES, and tRNAs, respectively.

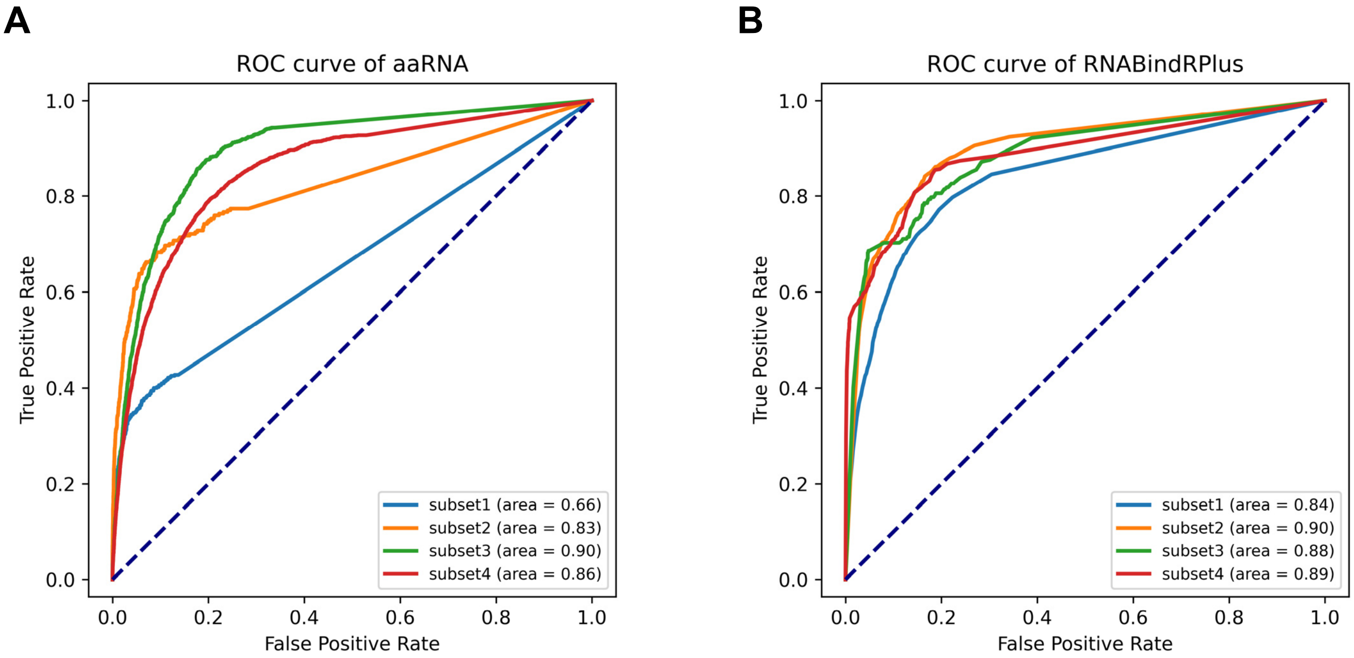

Supplementary Figure S2. ROC curves for the two partner-agnostic sequence-based predictors of RBRs that apply template proteins, aaRNA (panel A) and RNABindRPlus (panel B). The color-coded curves corresponds to subsets of benchmark proteins that share specific levels of similarity to the template proteins including <30% similarity (subset1 in blue), between 30% and 50% similarity (subset2 in orange), between 50% and 80% (subset3 in green), and over 80% (subset3 in red).

Benchmark dataset

Each of the 300 benchmark proteins are annotated using 9 lines:
#line1 >UniProt accession number (with DNA_ prefix for the set of 75 DNA-binding proteins)
#line2 protein sequence
#line3 RNA-binding residues (1=binding, 0=non-binding)
#line4 rRna binding residues (1=binding, 0=non-binding)
#line5 tRna binding residues (1=binding, 0=non-binding)
#line6 snRna binding residues (1=binding, 0=non-binding)
#line7 mRna binding residues (1=binding, 0=non-binding)
#line8 IRES binding residues (1=binding, 0=non-binding)
#line9 SRP binding residues (1=binding, 0=non-binding)

```
>O42661
MKLVRFLMKLTNETVSIELKNGTIVHGTITSVDMQMNTHLKAVKMTVKGREPVPVETLSIRGNNIRYYILPDSLPLDTLLIDDSTKPKQKKKEVVRGRGRGRGTRGRGRGASRGF
0001000000000000000100000000000001111000000000000000000000001110010000000000000000000000000000000000000000000000000
0000000000000000000000000000000000000000000000000000000000000000000000000000000000000000000000000000000000000000000
0000000000000000000000000000000000000000000000000000000000000000000000000000000000000000000000000000000000000000000
0001000000000000000100000000000001111000000000000000000000001110010000000000000000000000000000000000000000000000000
0000000000000000000000000000000000000000000000000000000000000000000000000000000000000000000000000000000000000000000
0000000000000000000000000000000000000000000000000000000000000000000000000000000000000000000000000000000000000000000
0000000000000000000000000000000000000000000000000000000000000000000000000000000000000000000000000000000000000000000
>E6PBU2
MTIVIRTKKILVNPLLSRRQLSLDVLHPDSPTASKEKIREELAKQLKVDARNVVVYGFSTQYGGGKSTGFALVYDNQQYLLKYEPNYRLRKVKILGEKPNTRRSFKELKRKIKRTSGKAITKLLSEKKGDTWASVQSKKSDHLKNFVAK
00000111110100000001000000000011111000000000000000000000011011111100000000000000000000010001000000010011101100110011001101100100000001000000000000000000
00000111110100000001000000000011111000000000000000000000011011111100000000000000000000010001000000010011101100110011001101100100000001000000000000000000
0000000000000000000000000000000000000000000000000000000000000000000000000000000000000000000000000000000000000000000000000000000000000000000000000
0000000000000000000000000000000000000000000000000000000000000000000000000000000000000000000000000000000000000000000000000000000000000000000000000
0000000000000000000000000000000000000000000000000000000000000000000000000000000000000000000000000000000000000000000000000000000000000000000000000
0000000000000000000000000000000000000000000000000000000000000000000000000000000000000000000000000000000000000000000000000000000000000000000000000
0000000000000000000000000000000000000000000000000000000000000000000000000000000000000000000000000000000000000000000000000000000000000000000000000
>P82249
MVLTLSNQFLAKIPATPKTLTLPKTSSSTLRPQWSCRKSDIHPEFREDAKVYCNGELVMTTGGTQKDYTVEVWSGNHPFYLGNRSALLLDADQVEKFRKKYGELTQIMEIPVLKGEIILPPKKKSKAKKK
0000000000000000000000000000000000000100010000000000000000000000000000000000000000000000000000000000000000000000000000000000000000
0000000000000000000000000000000000000100010000000000000000000000000000000000000000000000000000000000000000000000000000000000000000
0000000000000000000000000000000000000000000000000000000000000000000000000000000000000000000000000000000000000000000000000000000000
0000000000000000000000000000000000000000000000000000000000000000000000000000000000000000000000000000000000000000000000000000000000
0000000000000000000000000000000000000000000000000000000000000000000000000000000000000000000000000000000000000000000000000000000000
0000000000000000000000000000000000000000000000000000000000000000000000000000000000000000000000000000000000000000000000000000000000
0000000000000000000000000000000000000000000000000000000000000000000000000000000000000000000000000000000000000000000000000000000000
>E9BNI3
MPAKAAAKPVKPATKAAPKPANKAPAPKAAAAKPVAKAAPKAAAPAVRPASGSSNGVYVKNWGTGSVADATNVFSAAGKVVKVQLRRQRYALVFFENSAAVKKAIDLFNEKEVLGQTVLVVPAKASPKPDAHENSSCVFVSPIFRPSTTKAQVMELFAGVKVQRLRMYRQNFAYA
YLDSPAAAKKFVEEKNGTAFRGHTLRVALSARSLEKLRARQEVANVVIAAHRHYKVHERQ
0000000000000000000000000000000000000000000000000000100100000000000000000000000000011000000010001111100100010000000011110000100000000100101111111101010000000000101111111111010
000000000000000000001010010011110001100110010011000000000000
0000000000000000000000000000000000000000000000000000100100000000000000000000000000011000000010001111100100010000000011110000100000000100101111111101010000000000101111111111010
000000000000000000001010010011110001100110010011000000000000
0000000000000000000000000000000000000000000000000000000000000000000000000000000000000000000000000000000000000000000000000000000000000000000000000000000000000000000000000000000
000000000000000000000000000000000000000000000000000000000000
0000000000000000000000000000000000000000000000000000000000000000000000000000000000000000000000000000000000000000000000000000000000000000000000000000000000000000000000000000000
000000000000000000000000000000000000000000000000000000000000
0000000000000000000000000000000000000000000000000000000000000000000000000000000000000000000000000000000000000000000000000000000000000000000000000000000000000000000000000000000
000000000000000000000000000000000000000000000000000000000000
0000000000000000000000000000000000000000000000000000000000000000000000000000000000000000000000000000000000000000000000000000000000000000000000000000000000000000000000000000000
000000000000000000000000000000000000000000000000000000000000
0000000000000000000000000000000000000000000000000000000000000000000000000000000000000000000000000000000000000000000000000000000000000000000000000000000000000000000000000000000
000000000000000000000000000000000000000000000000000000000000
>Q02753
MGKSHGYRSRTRYMFQRDFRKHGAVHLSTYLKVYKVGDIVDIKANGSIQKGMPHKFYQGKTGVVYNVTKSSVGVIINKMVGNRYLEKRLNLRVEHIKHSKCRQEFLERVKANAAKRAEAKAQGVAVQLKRQPAQPRESRIVSTEGNVPQTLAPVPYETFI
0011111111111111110010111011100000001000101001101110010101111100000111110101011111110011010000000011000000000100000000000000000000000000000000000000000000000000
```

0011111111111111100101110111000000010000101001101110010101111100000111110101011111110011010000000011000000000100000000000000000000000000000000000000000000000000
0000000000000000000000000000000000000000000000000000000000000000000000000000000000000000000000000000000000000000000000000000000000000000000000000000000000000000
0000000000000000000000000000000000000000000000000000000000000000000000000000000000000000000000000000000000000000000000000000000000000000000000000000000000000000
0000000000000000000000000000000000000000000000000000000000000000000000000000000000000000000000000000000000000000000000000000000000000000000000000000000000000000
0000000000000000000000000000000000000000000000000000000000000000000000000000000000000000000000000000000000000000000000000000000000000000000000000000000000000000
0000000000000000000000000000000000000000000000000000000000000000000000000000000000000000000000000000000000000000000000000000000000000000000000000000000000000000
>Q97R12
MLKKNDIVEVEIVDLTHEGAGVAKVDGLVFFVENALPSEKILMRVLKVNKKIGFGKVEKYLVQSPHRNQDLDLAYLRSGIADLGHLSYPEQLKFKTKQVKDSLYKIAGIADVEVAETLGMEHPVKYRNKAQVPVRRVNGVLETGFFRKNSHNLMPLEDFFIQDPVIDQVVVALRD
LLRRFDLKPYDEKEQSGLIRNLVVRRGHYSGQIMVVLVTTRPKVFRVDQLIEQVIKQFPEIVSVMQNINDQNTNAIFGKEWRTLYGQDYITDQMLGNDFQIAGPAFYQVNTEMAEKLYQTAIDFAELKKDDVIIDAYSGIGTIGLSVAKHVKEVYGVELIPEAVENSQKNASLNK
ITNAHYVCDTAENAMKKWLKEGIQPTVILVDPPRKGLTESFIKASAQTGADRIAYISCNVATMARDIKLYQELGYELKKVQPVDLFPQTHHVETVALLSKLDVDKHISVEIELDEMDLTSAESKATYAQIKEYVWNKFELKVSTLYIAQIKKKCGIELREHYNKSKKDKQIIPQC
TPEKEEAIMDALRHFKMI
0000000000000001110000000000001000000000000000100000010000000000000000000000000000000000000000000000000000000000000000000000000000000000000000000000000000000000000000000
0000000000000000000000000000000000000000000000000000000000000000000000000000000000000000000000000000000000000000000000000000000000000000000000000000000000000000000000000000
0000000000000000000000000000000001000000000000000000000000000001000000000000000000000000000000000000000000000000000000000000000000000000000000000000000000000000000000000000
000000000000000000
0000000000000001110000000000001000000000000000100000010000000000000000000000000000000000000000000000000000000000000000000000000000000000000000000000000000000000000000000
0000000000000000000000000000000000000000000000000000000000000000000000000000000000000000000000000000000000000000000000000000000000000000000000000000000000000000000000000000
0000000000000000000000000000000001000000000000000000000000000001000000000000000000000000000000000000000000000000000000000000000000000000000000000000000000000000000000000000
000000000000000000
0000000000000000000000000000000000000000000000000000000000000000000000000000000000000000000000000000000000000000000000000000000000000000000000000000000000000000000000000000
0000000000000000000000000000000000000000000000000000000000000000000000000000000000000000000000000000000000000000000000000000000000000000000000000000000000000000000000000000
0000000000000000000000000000000000000000000000000000000000000000000000000000000000000000000000000000000000000000000000000000000000000000000000000000000000000000000000000000
000000000000000000
0000000000000000000000000000000000000000000000000000000000000000000000000000000000000000000000000000000000000000000000000000000000000000000000000000000000000000000000000000
0000000000000000000000000000000000000000000000000000000000000000000000000000000000000000000000000000000000000000000000000000000000000000000000000000000000000000000000000000
0000000000000000000000000000000000000000000000000000000000000000000000000000000000000000000000000000000000000000000000000000000000000000000000000000000000000000000000000000
000000000000000000
0000000000000000000000000000000000000000000000000000000000000000000000000000000000000000000000000000000000000000000000000000000000000000000000000000000000000000000000000000
0000000000000000000000000000000000000000000000000000000000000000000000000000000000000000000000000000000000000000000000000000000000000000000000000000000000000000000000000000
0000000000000000000000000000000000000000000000000000000000000000000000000000000000000000000000000000000000000000000000000000000000000000000000000000000000000000000000000000
000000000000000000
0000000000000000000000000000000000000000000000000000000000000000000000000000000000000000000000000000000000000000000000000000000000000000000000000000000000000000000000000000
0000000000000000000000000000000000000000000000000000000000000000000000000000000000000000000000000000000000000000000000000000000000000000000000000000000000000000000000000000
0000000000000000000000000000000000000000000000000000000000000000000000000000000000000000000000000000000000000000000000000000000000000000000000000000000000000000000000000000
000000000000000000
0000000000000000000000000000000000000000000000000000000000000000000000000000000000000000000000000000000000000000000000000000000000000000000000000000000000000000000000000000
0000000000000000000000000000000000000000000000000000000000000000000000000000000000000000000000000000000000000000000000000000000000000000000000000000000000000000000000000000
0000000000000000000000000000000000000000000000000000000000000000000000000000000000000000000000000000000000000000000000000000000000000000000000000000000000000000000000000000
000000000000000000
>P08199
MVKLAKAGKTHGEAKKMAPPPKEVEEDSEDEEMSEEEDDSSGEEVVIPQKKGKKATATPAKKVVVSQTKKVAVPTPAKKAAVTPGKKAAATPAKKAVTPAKAVATPGKKGATQAKALVATPGKKGAVTPAKGAKNGKNAKKEDSDEDEDDDDEDDSDEDEEDEEEDEFEPPVVK
GKQGKVAAAAPASEDEDEEEDEEEEEEDEEEEDDSEEEEAMEITPAKGKKAPAKVVPVKAKNVAEEDDDDEEEDEDEEEDEEEEEDEEEEEEEEEEEPVKPAPGKRKKEMTKQKEVPEAKKQKVEGSESTTPFNLFIGNLNPNKSVAELKVAISEPFAKNDLAVVDVRTGTNRKF
GYVDFESAEDLEKALELTGLKVFGNEIKLEKPKGRDSKKVRAARTLLAKNLSFNITEDELKEVFEDALEIRLVSQDGKSKGIAYIEFKSEADAEKNLEEKQGAEIDGRSVSLYYTGEKGQRQERTGKNSTWSGESKTLVLSNLSYSATEETLQEVFEKATFIKVPQNQQGKSKGY
AFIEFASFEDAKEALNSCNKMEIEGRTIRLELQGPRGSPNARSQPSKTLFVKGLSEDTTEETLKESFEGSVRARIVTDRETGSSKGFGFVDFNSEEDAKAAKEAMEDGEIDGNKVTLDWAKPKGEGGFGGRGGGRGGFGGRGGGRGGGRGGFGGRGRGGFGGRGGFRGGRGGGGG
GGDFKPQGKKTKFE
0000000000000000000000000000000000000000000000000000000000000000000000000000000000000000000000000000000000000000000000000000000000000000000000000000000000000000000000000000
0000000000000000000000000000000000000000000000000000000000000000000000000000000000000000000000000000000000000000000000000000000000000010000000000000000000000000000010111001
0100000000000000000000000000010110111110010000101000000000000000000000101000000101110000000000000000000000000000000000000000000000000000000000000000000000000000000000000000
0000000000000000000000000000000000000000000000000000000000000000000000000000000000000000000000000000000000000000000000000000000000000000000000000000000000000000000000000000
00000000000000
0000000000000000000000000000000000000000000000000000000000000000000000000000000000000000000000000000000000000000000000000000000000000000000000000000000000000000000000000000
0000000000000000000000000000000000000000000000000000000000000000000000000000000000000000000000000000000000000000000000000000000000000010000000000000000000000000000010111001
0100000000000000000000000000010110111110010000101000000000000000000000101000000101110000000000000000000000000000000000000000000000000000000000000000000000000000000000000000
0000000000000000000000000000000000000000000000000000000000000000000000000000000000000000000000000000000000000000000000000000000000000000000000000000000000000000000000000000
00000000000000
0000000000000000000000000000000000000000000000000000000000000000000000000000000000000000000000000000000000000000000000000000000000000000000000000000000000000000000000000000
0000000000000000000000000000000000000000000000000000000000000000000000000000000000000000000000000000000000000000000000000000000000000000000000000000000000000000000000000000
0000000000000000000000000000000000000000000000000000000000000000000000000000000000000000000000000000000000000000000000000000000000000000000000000000000000000000000000000000
0000000000000000000000000000000000000000000000000000000000000000000000000000000000000000000000000000000000000000000000000000000000000000000000000000000000000000000000000000
00000000000000
0000000000000000000000000000000000000000000000000000000000000000000000000000000000000000000000000000000000000000000000000000000000000000000000000000000000000000000000000000
0000000000000000000000000000000000000000000000000000000000000000000000000000000000000000000000000000000000000000000000000000000000000000000000000000000000000000000000000000
0000000000000000000000000000000000000000000000000000000000000000000000000000000000000000000000000000000000000000000000000000000000000000000000000000000000000000000000000000
0000000000000000000000000000000000000000000000000000000000000000000000000000000000000000000000000000000000000000000000000000000000000000000000000000000000000000000000000000
00000000000000
0000000000000000000000000000000000000000000000000000000000000000000000000000000000000000000000000000000000000000000000000000000000000000000000000000000000000000000000000000
0000000000000000000000000000000000000000000000000000000000000000000000000000000000000000000000000000000000000000000000000000000000000000000000000000000000000000000000000000
0000000000000000000000000000000000000000000000000000000000000000000000000000000000000000000000000000000000000000000000000000000000000000000000000000000000000000000000000000

0000000000000000000000000000000000000000000000000000000000000000000000000000000000000000000000000000000000000000000000000000000000000000000000000000000000000000000000000000000
00000000000000
0000000000000000000000000000000000000000000000000000000000000000000000000000000000000000000000000000000000000000000000000000000000000000000000000000000000000000000000000000000
0000000000000000000000000000000000000000000000000000000000000000000000000000000000000000000000000000000000000000000000000000000000000000000000000000000000000000000000000000000
0000000000000000000000000000000000000000000000000000000000000000000000000000000000000000000000000000000000000000000000000000000000000000000000000000000000000000000000000000000
0000000000000000000000000000000000000000000000000000000000000000000000000000000000000000000000000000000000000000000000000000000000000000000000000000000000000000000000000000000
00000000000000
0000000000000000000000000000000000000000000000000000000000000000000000000000000000000000000000000000000000000000000000000000000000000000000000000000000000000000000000000000000
0000000000000000000000000000000000000000000000000000000000000000000000000000000000000000000000000000000000000000000000000000000000000000000000000000000000000000000000000000000
0000000000000000000000000000000000000000000000000000000000000000000000000000000000000000000000000000000000000000000000000000000000000000000000000000000000000000000000000000000
0000000000000000000000000000000000000000000000000000000000000000000000000000000000000000000000000000000000000000000000000000000000000000000000000000000000000000000000000000000
00000000000000
>E6PBU3
MGKQPAAGQKKTKEAIAKAAQAKKGGKKKWTKGKAKDKVNHAVFIEKKNVESIINNPSKVGKVLTVSTVVEKLKVNGSLARQLMRTMADRKLVEKVAKNGNQWVYSVIGGVKEDKTAAPAAAGGKKQQQSKKGAAADKEEVQA
00000000000000000000000000111000000011101000000000000000000000000100000001111100100010000000000001101000000000000000000000000000000000000000000
00000000000000000000000000111000000011101000000000000000000000000100000001111100100010000000000001101000000000000000000000000000000000000000000
00000000000000000000000000000000000000000000000000000000000000000000000000000000000000000000000000000000000000000000000000000000000000000000000
00000000000000000000000000000000000000000000000000000000000000000000000000000000000000000000000000000000000000000000000000000000000000000000000
00000000000000000000000000000000000000000000000000000000000000000000000000000000000000000000000000000000000000000000000000000000000000000000000
00000000000000000000000000000000000000000000000000000000000000000000000000000000000000000000000000000000000000000000000000000000000000000000000
00000000000000000000000000000000000000000000000000000000000000000000000000000000000000000000000000000000000000000000000000000000000000000000000
>Q08963
MKFTPSIVIDAPQYYVDHFNGKYNVDKCVILRDLQLETDSESMPSSLKHLTKPTHILDLTNNDLIMIPDLSRRDDIHTLLLGRNNIVEVDGRLLPMNVQNLTLSNNSIRRFEDLQRLRRAPRTLKNLTLIGNQVCHLANYREHVLRLVPHLETLDFQNVTAEERKSAMSFPRQAD
GDTLGPVNTAIRDNGSRDKTMEIMNLVVSKMTVERRNELKKQLAEATSLEEIARLEKLLSGGV
0000000000000000000000000000000000000000000000000000000000000000000000000000000000000000000000000000000000000000000000000100000000000000000000000000000000000100100000000000000
000000000000000000000000000000000000000000000000000000000000000
0000000000000000000000000000000000000000000000000000000000000000000000000000000000000000000000000000000000000000000000000000000000000000000000000000000000000000000000000000000
000000000000000000000000000000000000000000000000000000000000000
0000000000000000000000000000000000000000000000000000000000000000000000000000000000000000000000000000000000000000000000000000000000000000000000000000000000000000000000000000000
000000000000000000000000000000000000000000000000000000000000000
0000000000000000000000000000000000000000000000000000000000000000000000000000000000000000000000000000000000000000000000000100000000000000000000000000000000000100100000000000000
000000000000000000000000000000000000000000000000000000000000000
0000000000000000000000000000000000000000000000000000000000000000000000000000000000000000000000000000000000000000000000000000000000000000000000000000000000000000000000000000000
000000000000000000000000000000000000000000000000000000000000000
0000000000000000000000000000000000000000000000000000000000000000000000000000000000000000000000000000000000000000000000000000000000000000000000000000000000000000000000000000000
000000000000000000000000000000000000000000000000000000000000000
0000000000000000000000000000000000000000000000000000000000000000000000000000000000000000000000000000000000000000000000000000000000000000000000000000000000000000000000000000000
000000000000000000000000000000000000000000000000000000000000000
>P32639
MTEHETKDKAKKIREIYRYDEMSNKVLKVDKRFMNTSQNPQRDAEISQPKSMSGRISAKDMGQGLCNNINKGLKENDVAVEKTGKSASLKKIQQHNTILNSSSDFRLHYYPKDPSNVETYEQILQWVTEVLGNDIPHDLIIGTADIFIRQLKENEENEDGNIEERKEKIQHELGI
NIDSLKFNELVKLMKNITDYETHPDNSNKQAVAILADDEKSDEEEVTEMSNNANVLGGEINDNEDDDEEYDYNDVEVNSKKKNKRALPNIENDIIKLSDSKTSNIESVPIYSIDEFFLQRKLRSELGYKDTSVIQDLSEKILNDIETLEHNPVALEQKLVDLLKFENISLAEFIL
KNRSTIFWGIRLAKSTENEIPNLIEKMVAKGLNDLVEQYKFRETTHSKRELDSGDDQPQSSEAKRTKFSNPAIPPVIDLEKIKFDESSKLMTVTKVSLPEGSFKRVKPQYDEIHIPAPSKPVIDYELKEITSLPDWCQEAFPSSETTSLNPIQSKVFHAAFEGDSNMLICAPTGS
GKTNIALLTVLKALSHHYNPKTKKLNLSAFKIVYIAPLKALVQEQVREFQRRLAFLGIKVAELTGDSRLSRKQIDETQVLVSTPEKWDITTRNSNNLAIVELVRLLIIDEIHLLHDDRGPVLESIVARTFWASKYGQEYPRIIGLSATLPNYEDVGRFLRVPKEGLFYFDSSFRP
CPLSQQFCGIKERNSLKKLKAMNDACYEKVLESINEGNQIIVFVHSRKETSRTATWLKNKFAEENITHKLTKNDAGSKQILKTEAANVLDPSLRKLIESGIGTHHAGLTRSDRSLSEDLFADGLLQVLVCTATLAWGVNLPAHTVIIKGTDVYSPEKGSWEQLSPQDVLQMLGRA
GRPRYDTFGEGIIITDQSNVQYYLSVLNQQLPIESQFVSKLVDNLNAEVVAGNIKCRNDAVNWLAYTYLYVRMLASPMLYKVPDISSDGQLKKFRESLVHSALCILKEQELVLYDAENDVIEATDLGNIASSFYINHASMDVYNRELDEHTTQIDLFRIFSMSEEFKYVSVRYEE
KRELKQLLEKAPIPIREDIDDPLAKVNVLLQSYFSQLKFEGFALNSDIVFIHQNAGRLLRAMFEICLKRGWGHPTRMLLNLCKSATTKMWPTNCPLRQFKTCPVEVIKRLEASTVPWGDYLQLETPAEVGRAIRSEKYGKQVYDLLKRFPKMSVTCNAQPITRSVMRFNIEIIAD
WIWDMNVHGSLEPFLLMLEDTDGDSILYYDVLFITPDIVGHEFTLSFTYELKQHNQNNLPPNFFLTLISENWWHSEFEIPVSFNGFKLPKKFPPPTPLLENISISTSELGNDDFSEVFEFKTFNKIQSQVFESLYNSNDSVFVGSGKGTGKTAMAELALLNHWRQNKGRAVYINP
SGEKIDFLLSDWNKRFSHLAGGKIINKLGNDPSLNLKLLAKSHVLLATPVQFELLSRRWRQRKNIQSLELMIYDDAHEISQGVYGAVYETLISRMIFIATQLEKKIRFVCLSNCLANARDFGEWAGMTKSNIYNFSPSERIEPLEINIQSFKDVEHISFNFSMLQMAFEASAAAA
GNRNSSSVFLPSRKDCMEVASAFMKFSKAIEWDMLNVEEEQIVPYIEKLTDGHLRAPLKHGVGILYKGMASNDERIVKRLYEYGAVSVLLISKDCSAFACKTDEVIILGTNLYDGAEHKYMPYTINELLEMVGLASGNDSMAGKVLILTSHNMKAYYKKFLIEPLPTESYLQYII
HDTLNNEIANSIIQSKQDCVDWFTYSYFYRRIHVNPSYYGVRDTSPHGISVFLSNLVETCLNDLVESSFIEIDDTEAEVTAEVNGGDDEATEIISTLSNGLIASHYGVSFFTIQSFVSSLSNTSTLKNMLYVLSTAVEFESVPLRKGDRALLVKLSKRLPLRFPEHTSSGSVSFK
VFLLLQAYFSRLELPVDFQNDLKDILEKVVPLINVVVDILSANGYLNATTAMDLAQMLIQGVWDVDNPLRQIPHFNNKILEKCKEINVETVYDIMALEDEERDEILTLTDSQLAQVAAFVNNYPNVELTYSLNNSDSLISGVKQKITIQLTRDVEPENLQVTSEKYPFDKLESWW
LVLGEVSKKELYAIKKVTLNKETQQYELEFDTPTSGKHNLTIWCVCDSYLDADKELSFEINVK
0000000000000000000000000000000000000000000000000000000000000000000000000000000000000000000000000000000000000000000000000000000000000000000000000000000000000000000000000000000
0000000000000000000000000000000000000000000000000000000000000000000000000000000000000000000000000000000000000000000000000000000000000000000000000000000000000000000000000000000
0000000000000000000000000000000000000111000000000000000000000000000000000000000000000000000000000000000000000000000000000000000000000000000000000000000000000000000000000000000
0000000000000000000000000000000000000010000000000000000000000000001111101100000000000110010001000000000000000000000000100000000000000000000000000000000000000000000000000000000
0000000000000001000000000000000000000000000011110001000000000000000000000000000000000000000000000000000001000000000000000000000000111001000000000000000010000000000000000000000
0000000000000000000000000000000000000000000000000000000000000000000000000000000000000010010000000000000000000000000000000000000000001100000000000000000000000000000010010001000
0000000000000000000000000000000000000000000000000100110010010000000010000000000000000000000000000001000000000000000000000000110000000011101100100010000000000000000000000000000
0000010001100000000000000000000000000000000000000000000000000000000000000000000000000000000000000000000000000000000000000000000000000000000000000000000000000000000000000000000
0000000000000000000000000000000000000000000000000000000000000000000000000000000000000000000000000000000000000000000000000000000000000000000000100000000000000000000000000000000
0000000000000000000000000010000000000000000000000000000000000000000000000000000000000000000000000000000000000000100000000000000000000000000000000000000000000000000000000000000
0000000000000000000000000000000000000000000000000000000000000000000000000000000000000000000000000000000000000000000000000000000000000000000000000010000000000000000000000000000
0000000000000000000000000000000000000000000000000000000000000000000000000000000000000000000000000000000000000000000000000000000000000000000000000000000000000000000000000000000
000000000000000000000000000000000000000000000000000000000000000
0000000000000000000000000000000000000000000000000000000000000000000000000000000000000000000000000000000000000000000000000000000000000000000000000000000000000000000000000000000
0000000000000000000000000000000000000000000000000000000000000000000000000000000000000000000000000000000000000000000000000000000000000000000000000000000000000000000000000000000
0000000000000000000000000000000000000000000000000000000000000000000000000000000000000000000000000000000000000000000000000000000000000000000000000000000000000000000000000000000
0000000000000000000000000000000000000000000000000000000000000000000000000000000000000000000000000000000000000000000000000000000000000000000000000000000000000000000000000000000

0000000000000000000000000000000000000000000000000000000000000000000000000000000000000000000000000000000000000000000000000000000000000000000000000000000000000000000000000000000
0000000000000000000000000000000000000000000000000000000000000000000000000000000000000000000000000000000000000000000000000000000000000000000000000000000000000000000000000000000
0000000000000000000000000000000000000000000000000000000000000000000000000000000000000000000000000000000000000000000000000000000000000000000000000000000000000000000000000000000
0000000000000000000000000000000000000000000000000000000000000000000000000000000000000000000000000000000000000000000000000000000000000000000000000000000000000000000000000000000
0000000000000000000000000000000000000000000000000000000000000000000000000000000000000000000000000000000000000000000000000000000000000000000000000000000000000000000000000000000
0000000000000000000000000000000000000000000000000000000000000000000000000000000000000000000000000000000000000000000000000000000000000000000000000000000000000000000000000000000
0000000000000000000000000000000000000000000000000000000000000000000000000000000000000000000000000000000000000000000000000000000000000000000000000000000000000000000000000000000
0000000000000000000000000000000000000000000000000000000000000000000000000000000000000000000000000000000000000000000000000000000000000000000000000000000000000000000000000000000
000000000000000000000000000000000000000000000000000000000000000
0000000000000000000000000000000000000000000000000000000000000000000000000000000000000000000000000000000000000000000000000000000000000000000000000000000000000000000000000000000
0000000000000000000000000000000000000000000000000000000000000000000000000000000000000000000000000000000000000000000000000000000000000000000000000000000000000000000000000000000
0000000000000000000000000000000000000000000000000000000000000000000000000000000000000000000000000000000000000000000000000000000000000000000000000000000000000000000000000000000
0000000000000000000000000000000000000000000000000000000000000000000000000000000000000000000000000000000000000000000000000000000000000000000000000000000000000000000000000000000
0000000000000000000000000000000000000000000000000000000000000000000000000000000000000000000000000000000000000000000000000000000000000000000000000000000000000000000000000000000
0000000000000000000000000000000000000000000000000000000000000000000000000000000000000000000000000000000000000000000000000000000000000000000000000000000000000000000000000000000
0000000000000000000000000000000000000000000000000000000000000000000000000000000000000000000000000000000000000000000000000000000000000000000000000000000000000000000000000000000
0000000000000000000000000000000000000000000000000000000000000000000000000000000000000000000000000000000000000000000000000000000000000000000000000000000000000000000000000000000
0000000000000000000000000000000000000000000000000000000000000000000000000000000000000000000000000000000000000000000000000000000000000000000000000000000000000000000000000000000
0000000000000000000000000000000000000000000000000000000000000000000000000000000000000000000000000000000000000000000000000000000000000000000000000000000000000000000000000000000
0000000000000000000000000000000000000000000000000000000000000000000000000000000000000000000000000000000000000000000000000000000000000000000000000000000000000000000000000000000
0000000000000000000000000000000000000000000000000000000000000000000000000000000000000000000000000000000000000000000000000000000000000000000000000000000000000000000000000000000
000000000000000000000000000000000000000000000000000000000000000
0000000000000000000000000000000000000000000000000000000000000000000000000000000000000000000000000000000000000000000000000000000000000000000000000000000000000000000000000000000
0000000000000000000000000000000000000000000000000000000000000000000000000000000000000000000000000000000000000000000000000000000000000000000000000000000000000000000000000000000
0000000000000000000000000000000000000111000000000000000000000000000000000000000000000000000000000000000000000000000000000000000000000000000000000000000000000000000000000000000
0000000000000000000000000000000000000010000000000000000000000000001111101100000000000110010001000000000000000000000001000000000000000000000000000000000000000000000000000000000
0000000000000001000000000000000000000000000011110001000000000000000000000000000000000000000000000000000001000000000000000000000000111001000000000000000010000000000000000000000
0000000000000000000000000000000000000000000000000000000000000000000000000000000000000000001001000000000000000000000000000000000000000000110000000000000000000000000000001001000100
0000000000000000000000000000000000000000000000000100110010010000000010000000000000000000000000000000100000000000000000000000001100000000111011001000100000000000000000000000000
0000010001100000000000000000000000000000000000000000000000000000000000000000000000000000000000000000000000000000000000000000000000000000000000000000000000000000000000000000000
0000000000000000000000000000000000000000000000000000000000000000000000000000000000000000000000000000000000000000000000000000000000000000001000000000000000000000000000000000
0000000000000000000000000010000000000000000000000000000000000000000000000000000000000000000000000000000000000000100000000000000000000000000000000000000000000000000000000000000
0000000000000000000000000000000000000000000000000000000000000000000000000000000000000000000000000000000000000000000000000000000000000000000000000000000000000010000000000000000000000000000
0000000000000000000000000000000000000000000000000000000000000000000000000000000000000000000000000000000000000000000000000000000000000000000000000000000000000000000000000000000
000000000000000000000000000000000000000000000000000000000000000
0000000000000000000000000000000000000000000000000000000000000000000000000000000000000000000000000000000000000000000000000000000000000000000000000000000000000000000000000000000
0000000000000000000000000000000000000000000000000000000000000000000000000000000000000000000000000000000000000000000000000000000000000000000000000000000000000000000000000000000
0000000000000000000000000000000000000000000000000000000000000000000000000000000000000000000000000000000000000000000000000000000000000000000000000000000000000000000000000000000
0000000000000000000000000000000000000000000000000000000000000000000000000000000000000000000000000000000000000000000000000000000000000000000000000000000000000000000000000000000
0000000000000000000000000000000000000000000000000000000000000000000000000000000000000000000000000000000000000000000000000000000000000000000000000000000000000000000000000000000
0000000000000000000000000000000000000000000000000000000000000000000000000000000000000000000000000000000000000000000000000000000000000000000000000000000000000000000000000000000
0000000000000000000000000000000000000000000000000000000000000000000000000000000000000000000000000000000000000000000000000000000000000000000000000000000000000000000000000000000
0000000000000000000000000000000000000000000000000000000000000000000000000000000000000000000000000000000000000000000000000000000000000000000000000000000000000000000000000000000
0000000000000000000000000000000000000000000000000000000000000000000000000000000000000000000000000000000000000000000000000000000000000000000000000000000000000000000000000000000
0000000000000000000000000000000000000000000000000000000000000000000000000000000000000000000000000000000000000000000000000000000000000000000000000000000000000000000000000000000
0000000000000000000000000000000000000000000000000000000000000000000000000000000000000000000000000000000000000000000000000000000000000000000000000000000000000000000000000000000
0000000000000000000000000000000000000000000000000000000000000000000000000000000000000000000000000000000000000000000000000000000000000000000000000000000000000000000000000000000
000000000000000000000000000000000000000000000000000000000000000
0000000000000000000000000000000000000000000000000000000000000000000000000000000000000000000000000000000000000000000000000000000000000000000000000000000000000000000000000000000
0000000000000000000000000000000000000000000000000000000000000000000000000000000000000000000000000000000000000000000000000000000000000000000000000000000000000000000000000000000
0000000000000000000000000000000000000000000000000000000000000000000000000000000000000000000000000000000000000000000000000000000000000000000000000000000000000000000000000000000
0000000000000000000000000000000000000000000000000000000000000000000000000000000000000000000000000000000000000000000000000000000000000000000000000000000000000000000000000000000
0000000000000000000000000000000000000000000000000000000000000000000000000000000000000000000000000000000000000000000000000000000000000000000000000000000000000000000000000000000
0000000000000000000000000000000000000000000000000000000000000000000000000000000000000000000000000000000000000000000000000000000000000000000000000000000000000000000000000000000
0000000000000000000000000000000000000000000000000000000000000000000000000000000000000000000000000000000000000000000000000000000000000000000000000000000000000000000000000000000
0000000000000000000000000000000000000000000000000000000000000000000000000000000000000000000000000000000000000000000000000000000000000000000000000000000000000000000000000000000
0000000000000000000000000000000000000000000000000000000000000000000000000000000000000000000000000000000000000000000000000000000000000000000000000000000000000000000000000000000
0000000000000000000000000000000000000000000000000000000000000000000000000000000000000000000000000000000000000000000000000000000000000000000000000000000000000000000000000000000
0000000000000000000000000000000000000000000000000000000000000000000000000000000000000000000000000000000000000000000000000000000000000000000000000000000000000000000000000000000
0000000000000000000000000000000000000000000000000000000000000000000000000000000000000000000000000000000000000000000000000000000000000000000000000000000000000000000000000000000
000000000000000000000000000000000000000000000000000000000000000
0000000000000000000000000000000000000000000000000000000000000000000000000000000000000000000000000000000000000000000000000000000000000000000000000000000000000000000000000000000
0000000000000000000000000000000000000000000000000000000000000000000000000000000000000000000000000000000000000000000000000000000000000000000000000000000000000000000000000000000
0000000000000000000000000000000000000000000000000000000000000000000000000000000000000000000000000000000000000000000000000000000000000000000000000000000000000000000000000000000
0000000000000000000000000000000000000000000000000000000000000000000000000000000000000000000000000000000000000000000000000000000000000000000000000000000000000000000000000000000
0000000000000000000000000000000000000000000000000000000000000000000000000000000000000000000000000000000000000000000000000000000000000000000000000000000000000000000000000000000
0000000000000000000000000000000000000000000000000000000000000000000000000000000000000000000000000000000000000000000000000000000000000000000000000000000000000000000000000000000
0000000000000000000000000000000000000000000000000000000000000000000000000000000000000000000000000000000000000000000000000000000000000000000000000000000000000000000000000000000
0000000000000000000000000000000000000000000000000000000000000000000000000000000000000000000000000000000000000000000000000000000000000000000000000000000000000000000000000000000

0000000000000000000000000000000000000000000000000000000000000000000000000000000000000000000000000000000000000000000000000000000000000000000000000000000000000000000000000000000
0000000000000000000000000000000000000000000000000000000000000000000000000000000000000000000000000000000000000000000000000000000000000000000000000000000000000000000000000000000
0000000000000000000000000000000000000000000000000000000000000000000000000000000000000000000000000000000000000000000000000000000000000000000000000000000000000000000000000000000
0000000000000000000000000000000000000000000000000000000000000000000000000000000000000000000000000000000000000000000000000000000000000000000000000000000000000000000000000000000
000000000000000000000000000000000000000000000000000000000000000
>P0A9W3
MAQFVYTMHRVGKVVPPKRHILKNISLSFFPGAKIGVLGLNGAGKSTLLRIMAGIDKDIEGEARPQPDIKIGYLPQEPQLNPEHTVRESIEEAVSEVVNALKRLDEVYALYADPDADFDKLAAEQGRLEEIIQAHDGHNLNVQLERAADALRLPDWDAKIANLSGGERRRVALCR
LLLEKPDMLLLDEPTNHLDAESVAWLERFLHDFEGTVVAITHDRYFLDNVAGWILELDRGEGIPWEGNYSSWLEQKDQRLAQEASQEAARRKSIEKELEWVRQGTKGRQSKGKARLARFEELNSTEYQKRNETNELFIPPGPRLGDKVLEVSNLRKSYGDRLLIDDLSFSIPKGA
IVGIIGPNGAGKSTLFRMISGQEQPDSGTITLGETVKLASVDQFRDSMDNSKTVWEEVSGGLDIMKIGNTEMPSRAYVGRFNFKGVDQGKRVGELSGGERGRLHLAKLLQVGGNMLLLDEPTNDLDIETLRALENALLEFPGCAMVISHDRWFLDRIATHILDYQDEGKVEFFEG
NFTEYEEYKKRTLGADALEPKRIKYKRIAK
0001000010000000000000000001000000000000000000000000000000000001000000000000000000000000000000000000000000000000000000000000000000000000000011100111010000000000000000000000000
0000000000000000000000000001100001000000000000000000000000000010100000000000000001000100000010000000011000000000000000000000000000000000000000001110000000000000000000000000000
0000000000000000000000000000000000000000000000000000000000000000000000000001000100000000000000000000000000000000000000000000000000000000000000000000000000000000000000000000000
000000000000000000000000000000
0001000010000000000000000001000000000000000000000000000000000001000000000000000000000000000000000000000000000000000000000000000000000000000011100111010000000000000000000000000
0000000000000000000000000001100001000000000000000000000000000010100000000000000001000100000010000000000000000000000000000000000000000000000000001110000000000000000000000000000
0000000000000000000000000000000000000000000000000000000000000000000000000000000000000000000000000000000000000000000000000000000000000000000000000000000000000000000000000000000
000000000000000000000000000000
0000000000000000000000000000000000000000000000000000000000000000000000000000000000000000000000000000000000000000000000000000000000000000000000000000000000000000000000000000000
0000000000000000000000000000000000000000000000000000000000000000000000000000000000000000000000000110000000000000000000000000000000000000000000000000000000000000000000000000000
0000000000000000000000000000000000000000000000000000000000000000000000000001000100000000000000000000000000000000000000000000000000000000000000000000000000000000000000000000000
000000000000000000000000000000
0000000000000000000000000000000000000000000000000000000000000000000000000000000000000000000000000000000000000000000000000000000000000000000000000000000000000000000000000000000
0000000000000000000000000000000000000000000000000000000000000000000000000000000000000000000000000000000000000000000000000000000000000000000000000000000000000000000000000000000
0000000000000000000000000000000000000000000000000000000000000000000000000000000000000000000000000000000000000000000000000000000000000000000000000000000000000000000000000000000
000000000000000000000000000000
0000000000000000000000000000000000000000000000000000000000000000000000000000000000000000000000000000000000000000000000000000000000000000000000000000000000000000000000000000000
0000000000000000000000000000000000000000000000000000000000000000000000000000000000000000000000000000000000000000000000000000000000000000000000000000000000000000000000000000000
0000000000000000000000000000000000000000000000000000000000000000000000000000000000000000000000000000000000000000000000000000000000000000000000000000000000000000000000000000000
000000000000000000000000000000
0000000000000000000000000000000000000000000000000000000000000000000000000000000000000000000000000000000000000000000000000000000000000000000000000000000000000000000000000000000
0000000000000000000000000000000000000000000000000000000000000000000000000000000000000000000000000000000000000000000000000000000000000000000000000000000000000000000000000000000
0000000000000000000000000000000000000000000000000000000000000000000000000000000000000000000000000000000000000000000000000000000000000000000000000000000000000000000000000000000
000000000000000000000000000000
0000000000000000000000000000000000000000000000000000000000000000000000000000000000000000000000000000000000000000000000000000000000000000000000000000000000000000000000000000000
0000000000000000000000000000000000000000000000000000000000000000000000000000000000000000000000000000000000000000000000000000000000000000000000000000000000000000000000000000000
0000000000000000000000000000000000000000000000000000000000000000000000000000000000000000000000000000000000000000000000000000000000000000000000000000000000000000000000000000000
000000000000000000000000000000
>E6PBT9
MNSGRANQRSKPMTGLINDKKEKIDAYLPRKCDWSNKLIFSNDQSSVQIAIAEVGENGQATGSKTNVVLCGSVRSKGEAHIALENILRERGLYPIQE
0000000000000000000010000000000000000000000010000000000000000000000000110011000000000000000000000
0000000000000000000010000000000000000000000010000000000000000000000000110011000000000000000000000
0000000000000000000000000000000000000000000000000000000000000000000000000000000000000000000000000
0000000000000000000000000000000000000000000000000000000000000000000000000000000000000000000000000
0000000000000000000000000000000000000000000000000000000000000000000000000000000000000000000000000
0000000000000000000000000000000000000000000000000000000000000000000000000000000000000000000000000
0000000000000000000000000000000000000000000000000000000000000000000000000000000000000000000000000
>Q8TEA1
MSIFPKISLRPEVENYLKEGFMNKEIVTALGKQEAERKFETLLKHLSHPPSFTTVRVNTHLASVQHVKNLLLDELQKQFNGLSVPILQHPDLQDVLLIPVIGPRKNIKKQQCEAIVGAQCGNAVLRGAHVYAPGIVSASQFMKAGDVISVYSDIKGKCKKGAKEFDGTKVFLGNG
ISELSRKEIFSGLPELKGMGIRMTEPVYLSPSFDSVLPRYLFLQNLPSALVSHVLNPQPGEKILDLCAAPGGKTTHIAALMHDQGEVIALDKIFNKVEKIKQNALLLGLNSIRAFCFDGTKAVKLDMVEDTEGEPPFLPESFDRILLDAPCSGMGQRPNMACTWSVKEVASYQPL
QRKLFTAAVQLLKPEGVLVYSTCTITLAENEEQVAWALTKFPCLQLQPQEPQIGGEGMRGAGLSCEQLKQLQRFDPSAVPLPDTDMDSLREARREDMLRLANKDSIGFFIAKFVKCKST
0000000000000000000000000000000000000000000000000000110000000000000000000000000000000000000000000000000000000000000000110010011110101100000110000000000000000111100000000000000
0000011001111000110000000000111100000000011100100000000000000000000001001000000000000000000010010000000000000000000000000000000000000000000000000001101000010000000100110011011
01100000000000000000111000000000000000000000000000000000000000000000000000000000000000000000000000000000000100001000000
0000000000000000000000000000000000000000000000000000000000000000000000000000000000000000000000000000000000000000000000000000000000000000000000000000000000000000000000000000000
0000000000000000000000000000000000000000000000000000000000000000000000000000000000000000000000000000000000000000000000000000000000000000000000000000000000000000000000000000000
00000000000000000000000000000000000000000000000000000000000000000000000000000000000000000000000000000000000000000000000
0000000000000000000000000000000000000000000000000000110000000000000000000000000000000000000000000000000000000000000000110010011110101100000110000000000000000111100000000000000
0000011001111000110000000000111100000000011100100000000000000000000001001000000000000000000010010000000000000000000000000000000000000000000000000001101000010000000100110011011
01100000000000000000111000000000000000000000000000000000000000000000000000000000000000000000000000000000000100001000000
0000000000000000000000000000000000000000000000000000000000000000000000000000000000000000000000000000000000000000000000000000000000000000000000000000000000000000000000000000000
0000000000000000000000000000000000000000000000000000000000000000000000000000000000000000000000000000000000000000000000000000000000000000000000000000000000000000000000000000000
00000000000000000000000000000000000000000000000000000000000000000000000000000000000000000000000000000000000000000000000
0000000000000000000000000000000000000000000000000000000000000000000000000000000000000000000000000000000000000000000000000000000000000000000000000000000000000000000000000000000
0000000000000000000000000000000000000000000000000000000000000000000000000000000000000000000000000000000000000000000000000000000000000000000000000000000000000000000000000000000
00000000000000000000000000000000000000000000000000000000000000000000000000000000000000000000000000000000000000000000000
0000000000000000000000000000000000000000000000000000000000000000000000000000000000000000000000000000000000000000000000000000000000000000000000000000000000000000000000000000000
0000000000000000000000000000000000000000000000000000000000000000000000000000000000000000000000000000000000000000000000000000000000000000000000000000000000000000000000000000000
00000000000000000000000000000000000000000000000000000000000000000000000000000000000000000000000000000000000000000000000

0000000000000000000000000000000000000000000000000000000000000000000000000000000000000000000000000000000000000000000000000000000000000000000000000000000000000000000000000000000
0000000000000000000000000000000000000000000000000000000000000000000000000000000000000000000000000000000000000000000000000000000000000000000000000000000000000000000000000000000
00000000000000000000000000000000000000000000000000000000000000000000000000000000000000000000000000000000000000000000000
>A0R215
MKTGIHPEYVDTTVQCGCGHSFTTRSTKQSGTIVVEVCSQCHPFYTGKQKILDSGGRVARFEKRYGKRNKAAADK
110011000000000000000000000000000000000000000000000000000000000000000000000
110011000000000000000000000000000000000000000000000000000000000000000000000
000000000000000000000000000000000000000000000000000000000000000000000000000
000000000000000000000000000000000000000000000000000000000000000000000000000
000000000000000000000000000000000000000000000000000000000000000000000000000
000000000000000000000000000000000000000000000000000000000000000000000000000
000000000000000000000000000000000000000000000000000000000000000000000000000
>Q96NC0
MASGSGTKNLDFRRKWDKDEYEKLAEKRLTEEREKKDGKPVQPVKRELLRHRDYKVDLESKLGKTIVITKTTPQSEMGGYYCNVCDCVVKDSINFLDHINGKKHQRNLGMSMRVERSTLDQVKKRFEVNKKKMEEKQKDYDFEERMKELREEEEKAKAYKKEKQKEKKRRAEEDL
TFEEDDEMAAVMGFSGFGSTKKSY
0000000000000000000000000000000000000000000000000000000000000000000000000000000000000011000001001100110000000000000000000000000000000000000000000000000000000000000000000000000
000000000000000000000000
0000000000000000000000000000000000000000000000000000000000000000000000000000000000000000000000000000000000000000000000000000000000000000000000000000000000000000000000000000000
000000000000000000000000
0000000000000000000000000000000000000000000000000000000000000000000000000000000000000000000000000000000000000000000000000000000000000000000000000000000000000000000000000000000
000000000000000000000000
0000000000000000000000000000000000000000000000000000000000000000000000000000000000000000000000000000000000000000000000000000000000000000000000000000000000000000000000000000000
000000000000000000000000
0000000000000000000000000000000000000000000000000000000000000000000000000000000000000011000001001100110000000000000000000000000000000000000000000000000000000000000000000000000
000000000000000000000000
0000000000000000000000000000000000000000000000000000000000000000000000000000000000000000000000000000000000000000000000000000000000000000000000000000000000000000000000000000000
000000000000000000000000
0000000000000000000000000000000000000000000000000000000000000000000000000000000000000000000000000000000000000000000000000000000000000000000000000000000000000000000000000000000
000000000000000000000000
>Q24F70
MVHVLKATKIRIYKQLLQDGVFVLKKDFEGHHEETGVPNLHCYILVRSLKDRGFLEEIFNWGFTYYYLNKEGCEYLKTKLGISADNVIPKTFKASNVNFISKEEDEEERPRRQFNKGGRTGERDGRNKRGVGRGTRREGEEAAKEEGAAETAQGNQETPAQE
011101001000000000000000010100000000000100110011001100000010000000000000000000000000000000000000000000000000000000000000000000000000000000000000000000000000000000
011101001000000000000000010100000000000100110011001100000010000000000000000000000000000000000000000000000000000000000000000000000000000000000000000000000000000000
000000000000000000000000000000000000000000000000000000000000000000000000000000000000000000000000000000000000000000000000000000000000000000000000000000000000000000
000000000000000000000000000000000000000000000000000000000000000000000000000000000000000000000000000000000000000000000000000000000000000000000000000000000000000000
000000000000000000000000000000000000000000000000000000000000000000000000000000000000000000000000000000000000000000000000000000000000000000000000000000000000000000
000000000000000000000000000000000000000000000000000000000000000000000000000000000000000000000000000000000000000000000000000000000000000000000000000000000000000000
000000000000000000000000000000000000000000000000000000000000000000000000000000000000000000000000000000000000000000000000000000000000000000000000000000000000000000
>P46198
MNRKILKLENLLRFHTICRQLHSLCQRRMLAQWRHMFSSAYAVHTAQLYTRPWQTDALLRAALSQRRLLVTKKEKRSQKSPLPSTKSKKEVEVWLGMTVEELARAMEKDIDCVYESLMNTAIDIDSLETHSRLDEVWIKEVIKKSGMKLKWSKLKQDKVRENKDAVKRPQADPAL
LIPRSPVVTIMGHVDHGKTTLLDKLRKTQVAAMEAGGITQHIGAFLVSLPSGEKITFLDTPGHAAFSAMRARGTQVTDIVILVVAADDGVMKQTVESIQHAKDAHVPIVLAINKCDKAEADPEKVKKELLAYDVVCEDYGGDVQAVHVSALTGENMMALAEATIALAEMLELKAD
PTGAVEGTVIESFTDKGRGPVTTAIIQRGTLRKGSILVAGKSWAKVRLMFDENGRAVNEAYPSMPVGIIGWRDLPSAGDEILEVESEPRAREVVDWRKYEQEQEKNKEDLKLIEEKRKEHQEAHRKDREKYGTVHWKERSYIKYREKRQQQPLKPKEKLERDSNVLPVIVKGDVD
GSVEAILNVMDTYDASHECELDLVHFGVGDISENDVNLAETFHGVIYGFNVNAGNVIQQLAAKKGVKIKLHKIIYRLIEDLQEELSSRLPCIVEEHPIGEASILATFSITEGKKKVPVAGCRVQKGQIEKQKKFKLIRNGHVIWKGSLISLKHHKDDTSVVKTGMDCGLSLDEEK
IEFKVGDAIICYEEKEVPAKTSWDPGF
0000000000000000000000000000000000000000000000000000000000000000000000000000000000000000000000000000000000000000000000000000000000000000000000000000000000000000000000000000000
0000000000000000000000000000000000000000000000000000000000000000000000000000000000000000000000000000000000000000000000000000000000000000000000000000000000000000000000000000000
0000000000000000000000000000000000000000000000000000000000000000000000000000000000000000000000000000000000000000000000000000000000000000000000000000000000000000000000000000000
0000000000000000000000000000000000000000000000000000000000000000000000000000000000000000000000000001000000111000000000000000000000000000000000000000010000000000000000000000
000000000000000000000000000
0000000000000000000000000000000000000000000000000000000000000000000000000000000000000000000000000000000000000000000000000000000000000000000000000000000000000000000000000000000
0000000000000000000000000000000000000000000000000000000000000000000000000000000000000000000000000000000000000000000000000000000000000000000000000000000000000000000000000000000
0000000000000000000000000000000000000000000000000000000000000000000000000000000000000000000000000000000000000000000000000000000000000000000000000000000000000000000000000000000
0000000000000000000000000000000000000000000000000000000000000000000000000000000000000000000000000000000000000000000000000000000000000000000000000000000000000000000000000000000
000000000000000000000000000
0000000000000000000000000000000000000000000000000000000000000000000000000000000000000000000000000000000000000000000000000000000000000000000000000000000000000000000000000000000
0000000000000000000000000000000000000000000000000000000000000000000000000000000000000000000000000000000000000000000000000000000000000000000000000000000000000000000000000000000
0000000000000000000000000000000000000000000000000000000000000000000000000000000000000000000000000000000000000000000000000000000000000000000000000000000000000000000000000000000
0000000000000000000000000000000000000000000000000000000000000000000000000000000000000000000000000001000000111000000000000000000000000000000000000000010000000000000000000000
000000000000000000000000000
0000000000000000000000000000000000000000000000000000000000000000000000000000000000000000000000000000000000000000000000000000000000000000000000000000000000000000000000000000000
0000000000000000000000000000000000000000000000000000000000000000000000000000000000000000000000000000000000000000000000000000000000000000000000000000000000000000000000000000000
0000000000000000000000000000000000000000000000000000000000000000000000000000000000000000000000000000000000000000000000000000000000000000000000000000000000000000000000000000000
0000000000000000000000000000000000000000000000000000000000000000000000000000000000000000000000000000000000000000000000000000000000000000000000000000000000000000000000000000000
000000000000000000000000000
0000000000000000000000000000000000000000000000000000000000000000000000000000000000000000000000000000000000000000000000000000000000000000000000000000000000000000000000000000000
0000000000000000000000000000000000000000000000000000000000000000000000000000000000000000000000000000000000000000000000000000000000000000000000000000000000000000000000000000000
0000000000000000000000000000000000000000000000000000000000000000000000000000000000000000000000000000000000000000000000000000000000000000000000000000000000000000000000000000000
0000000000000000000000000000000000000000000000000000000000000000000000000000000000000000000000000000000000000000000000000000000000000000000000000000000000000000000000000000000
000000000000000000000000000

```
0000000000000000000000000000000000000000000000000000000000000000000000000000000000000000000000000000000000000000000000000000000000000000000000000000000000000000000000000000000
0000000000000000000000000000000000000000000000000000000000000000000000000000000000000000000000000000000000000000000000000000000000000000000000000000000000000000000000000000000
0000000000000000000000000000000000000000000000000000000000000000000000000000000000000000000000000000000000000000000000000000000000000000000000000000000000000000000000000000000
0000000000000000000000000000000000000000000000000000000000000000000000000000000000000000000000000000000000000000000000000000000000000000000000000000000000000000000000000000000
000000000000000000000000000
0000000000000000000000000000000000000000000000000000000000000000000000000000000000000000000000000000000000000000000000000000000000000000000000000000000000000000000000000000000
0000000000000000000000000000000000000000000000000000000000000000000000000000000000000000000000000000000000000000000000000000000000000000000000000000000000000000000000000000000
0000000000000000000000000000000000000000000000000000000000000000000000000000000000000000000000000000000000000000000000000000000000000000000000000000000000000000000000000000000
0000000000000000000000000000000000000000000000000000000000000000000000000000000000000000000000000000000000000000000000000000000000000000000000000000000000000000000000000000000
000000000000000000000000000
>P02406
MPSRFTKTRKHRGHVSAGKGRIGKHRKHPGGRGMAGGQHHHRINMDKYHPGYFGKVGMRYFHKQQAHFWKPVLNLDKLWTLIPEDKRDQYLKSASKETAPVIDTLAAGYGKILGKGRIPNVPVIVKARFVSKLAEEKIRAAGGVVELIA
11011111111111111111110111111111111111111111111110000000111111111110111001010010110001000000000000000000000000000000111111000000000000111000000000000000000
11011111111111111111110111111111111111111111111110000000111111111110111001010010110001000000000000000000000000000000111111000000000000111000000000000000000
00000000000000000000000000000000000000000000000000000000000000000000000000000000000000000000000000000000000000000000000000000000000000000000000000000
00000000000000000000000000000000000000000000000000000000000000000000000000000000000000000000000000000000000000000000000000000000000000000000000000000
00000000000000000000000000000000000000000000000000000000000000000000000000000000000000000000000000000000000000000000000000000000000000000000000000000
00000000000000000000000000000000000000000000000000000000000000000000000000000000000000000000000000000000000000000000000000000000000000000000000000000
00000000000000000000000000000000000000000000000000000000000000000000000000000000000000000000000000000000000000000000000000000000000000000000000000000
>P0A7V0
MATVSMRDMLKAGVHFGHQTRYWNPKMKPFIFGARNKVHIINLEKTVPMFNEALAELNKIASRKGKILFVGTKRAASEAVKDAALSCDQFFVNHRWLGGMLTNWKTVRQSIKRLKDLETQSQDGTFDKLTKKEALMRTRELEKLENSLGGIKDMGGLPDALFVIDADHEHIAIKE
ANNLGIPVFAIVDTNSDPDGVDFVIPGNDDAIRAVTLYLGAVAATVREGRSQDLASQAEESFVEAE
0000000000000000011111111111111100111111110011000000000000000000000000000110000000000000000000111111101101110110110000000001110111111101111110011000000000000000000000000110011
001000000000000111111100000010000000000000000000000000000000000000
0000000000000000011111111111111100111111110011000000000000000000000000000110000000000000000000111111101101110110110000000001110111111101111110011000000000000000000000000110011
001000000000000111111100000010000000000000000000000000000000000000
0000000000000000000000000000000000000000000000000000000000000000000000000000000000000000000000000000000000000000000000000000000000000000000000000000000000000000000000000000000
000000000000000000000000000000000000000000000000000000000000000000
0000000000000000000000000000000000000000000000000000000000000000000000000000000000000000000000000000000000000000000000000000000000000000000000000000000000000000000000000000000
000000000000000000000000000000000000000000000000000000000000000000
0000000000000000000000000000000000000000000000000000000000000000000000000000000000000000000000000000000000000000000000000000000000000000000000000000000000000000000000000000000
000000000000000000000000000000000000000000000000000000000000000000
0000000000000000000000000000000000000000000000000000000000000000000000000000000000000000000000000000000000000000000000000000000000000000000000000000000000000000000000000000000
000000000000000000000000000000000000000000000000000000000000000000
0000000000000000000000000000000000000000000000000000000000000000000000000000000000000000000000000000000000000000000000000000000000000000000000000000000000000000000000000000000
000000000000000000000000000000000000000000000000000000000000000000
>E6PBV1
MNDNDFQFENNIDDFQTHIHIRVEQRRGRKCFTTVEGIPPEFDYEKIMKYWKKWLSCNATIVEEDEGKKVIKLNGDHRNQIQQFLSEEGIAAVDNITIHGI
00000000000000000000000001001101000000000000000010011001110100000000000001100000000000000000000000000
00000000000000000000000001001101000000000000000010011001110100000000000001100000000000000000000000000
00000000000000000000000000000000000000000000000000000000000000000000000000000000000000000000000000000
00000000000000000000000000000000000000000000000000000000000000000000000000000000000000000000000000000
00000000000000000000000000000000000000000000000000000000000000000000000000000000000000000000000000000
00000000000000000000000000000000000000000000000000000000000000000000000000000000000000000000000000000
00000000000000000000000000000000000000000000000000000000000000000000000000000000000000000000000000000
>E6PBT3
MADNKKSIKKFTFRGKGLEELTALASGSNSEKLISDELAALFDAKTRRRVKRGISEKYAKFVNKVRRSKEKCPAGEKPVPVKTHYRSMIVIPELVGGIVGVYNGKEFVNVEVKFDMIGKYLAEFAMTYKPTTHGKTGAAGGKGK
000000000000010000000000000000000000000000011011100110011001000100000000000000000111111000000000000011110100000100000010110101111111111000000000
000000000000010000000000000000000000000000011011100110011001000100000000000000000111111000000000000011110100000100000010110101111111111000000000
000000000000000000000000000000000000000000000000000000000000000000000000000000000000000000000000000000000000000000000000000000000000000000000000
000000000000000000000000000000000000000000000000000000000000000000000000000000000000000000000000000000000000000000000000000000000000000000000000
000000000000000000000000000000000000000000000000000000000000000000000000000000000000000000000000000000000000000000000000000000000000000000000000
000000000000000000000000000000000000000000000000000000000000000000000000000000000000000000000000000000000000000000000000000000000000000000000000
000000000000000000000000000000000000000000000000000000000000000000000000000000000000000000000000000000000000000000000000000000000000000000000000
>P0DJ24
MTRGTPAFGKRHQKTHTLCRRCGKATYHKQKLRCAACGYPDAKMRRYDGWGQKVRDRKGQGTGRMRYMKTIARRAKNGFRSGTQAAPKVKAATN
0111110111111111000000011101011110110000011111111111111110010000000000000000011101000001110100000
0111110111111111000000011101011110110000011111111111111110010000000000000000011101000001110100000
0000000000000000000000000000000000000000000000000000000000000000000000000000000000000000000000
0000000000000000000000000000000000000000000000000000000000000000000000000000000000000000000000
0000000000000000000000000000000000000000000000000000000000000000000000000000000000000000000000
0000000000000000000000000000000000000000000000000000000000000000000000000000000000000000000000
0000000000000000000000000000000000000000000000000000000000000000000000000000000000000000000000
>P54999
MSESSDISAMQPVNPKPFLKGLVNHRVGVKLKFNSTEYRGTLVSTDNYFNLQLNEAEEFVAGVSHGTLGEIFIRCNNVLYIRELPN
00000000000000000000000000000001100000000000001111000000000000000000000001110000000000
00000000000000000000000000000000000000000000000000000000000000000000000000000000000000
00000000000000000000000000000000000000000000000000000000000000000000000000000000000000
00000000000000000000000000000001100000000000001111000000000000000000000001110000000000
```

00000000000000000000000000000000000000000000000000000000000000000000000000000000000000
00000000000000000000000000000000000000000000000000000000000000000000000000000000000000
00000000000000000000000000000000000000000000000000000000000000000000000000000000000000
>O34687
MAVPFRRTSKMKKRLRRTHFKLNVPGMTECPSCGEMKLSHRVCKACGSYNGKDINVKSN
01111111111111111111111010011010000010111010000111000000000
01111111111111111111111010011010000010111010000111000000000
00000000000000000000000000000000000000000000000000000000000
00000000000000000000000000000000000000000000000000000000000
00000000000000000000000000000000000000000000000000000000000
00000000000000000000000000000000000000000000000000000000000
00000000000000000000000000000000000000000000000000000000000
>Q03195
MSDKNSRIAIVSADKCKPKKCRQECKRSCPVVKTGKLCIEVTPTSKIAFISEILCIGCGICVKKCPFDAIQIINLPTNLEAHVTHRYSANSFKLHRLPTPRPGQVLGLVGTNGIGKSTALKILAGKQKPNLGRFDDPPEWQEIIKYFRGSELQNYFTKMLEDDIKAIIKPQYVDN
IPRAIKGPVQKVGELLKLRMEKSPEDVKRYIKILQLENVLKRDIEKLSGGELQRFAIGMSCVQEADVYMFDEPSSYLDVKQRLNAAQIIRSLLAPTKYVICVEHDLSVLDYLSDFVCIIYGVPSVYGVVTLPASVREGINIFLDGHIPAENLRFRTEALQFRIADATEDLQNDSA
SRAFSYPSLKKTQGDFVLNVEEGEFSDSEILVMMGENGTGKTTLIKLLAGALKPDEGQDIPKLNVSMKPQKIAPKFPGTVRQLFFKKIRGQFLNPQFQTDVVKPLRIDDIIDQEVQHLSGGELQRVAIVLALGIPADIYLIDEPSAYLDSEQRIICSKVIRRFILHNKKTAFIVE
HDFIMATYLADKVIVFEGIPSKNAHARAPESLLTGCNRFLKNLNVTFRRDPNSFRPRINKLDSQMDKEQKSSGNYFFLDNTGI
0000000000000000000000000000000000000000000000000000000000000000000000000000000000000000000000000000000000000000000000000000000000000000000000000001001010000000000000000000000
0000000000000000000000000000000000000000000000000000000000000000000000000000000000000000000000000000000000000000000000000000000000000101100000000000000000001000010000000000000
0000000000000000000000000000000000000000000000000000000000000000000000000000000000000000000000110000000000000000000000000000000000000000000000000000000000000000010000000000000
00000000000000000000000000000000000000000000000100000000110101110000000000000000000
0000000000000000000000000000000000000000000000000000000000000000000000000000000000000000000000000000000000000000000000000000000000000000000000000001001010000000000000000000000
0000000000000000000000000000000000000000000000000000000000000000000000000000000000000000000000000000000000000000000000000000000000000101100000000000000000001000010000000000000
0000000000000000000000000000000000000000000000000000000000000000000000000000000000000000000000110000000000000000000000000000000000000000000000000000000000000000010000000000000
00000000000000000000000000000000000000000000000100000000110101110000000000000000000
0000000000000000000000000000000000000000000000000000000000000000000000000000000000000000000000000000000000000000000000000000000000000000000000000000000000000000000000000000000
0000000000000000000000000000000000000000000000000000000000000000000000000000000000000000000000000000000000000000000000000000000000000000000000000000000000000000000000000000000
0000000000000000000000000000000000000000000000000000000000000000000000000000000000000000000000000000000000000000000000000000000000000000000000000000000000000000000000000000000
00000000000000000000000000000000000000000000000000000000000000000000000000000000000
0000000000000000000000000000000000000000000000000000000000000000000000000000000000000000000000000000000000000000000000000000000000000000000000000000000000000000000000000000000
0000000000000000000000000000000000000000000000000000000000000000000000000000000000000000000000000000000000000000000000000000000000000000000000000000000000000000000000000000000
0000000000000000000000000000000000000000000000000000000000000000000000000000000000000000000000000000000000000000000000000000000000000000000000000000000000000000000000000000000
00000000000000000000000000000000000000000000000000000000000000000000000000000000000
0000000000000000000000000000000000000000000000000000000000000000000000000000000000000000000000000000000000000000000000000000000000000000000000000000000000000000000000000000000
0000000000000000000000000000000000000000000000000000000000000000000000000000000000000000000000000000000000000000000000000000000000000000000000000000000000000000000000000000000
0000000000000000000000000000000000000000000000000000000000000000000000000000000000000000000000000000000000000000000000000000000000000000000000000000000000000000000000000000000
00000000000000000000000000000000000000000000000000000000000000000000000000000000000
0000000000000000000000000000000000000000000000000000000000000000000000000000000000000000000000000000000000000000000000000000000000000000000000000000000000000000000000000000000
0000000000000000000000000000000000000000000000000000000000000000000000000000000000000000000000000000000000000000000000000000000000000000000000000000000000000000000000000000000
0000000000000000000000000000000000000000000000000000000000000000000000000000000000000000000000000000000000000000000000000000000000000000000000000000000000000000000000000000000
00000000000000000000000000000000000000000000000000000000000000000000000000000000000
0000000000000000000000000000000000000000000000000000000000000000000000000000000000000000000000000000000000000000000000000000000000000000000000000000000000000000000000000000000
0000000000000000000000000000000000000000000000000000000000000000000000000000000000000000000000000000000000000000000000000000000000000000000000000000000000000000000000000000000
0000000000000000000000000000000000000000000000000000000000000000000000000000000000000000000000000000000000000000000000000000000000000000000000000000000000000000000000000000000
00000000000000000000000000000000000000000000000000000000000000000000000000000000000
>Q9LE95
MATAAPPSLSLCYASSSFQYQQDPSFQTHFKPLLLNSSLCRLTLNQRERSCLKWVDFTSQKQSPKSVSFRVLAAVAAEAEVADMEEEEGESGGVATLPSPTKPKKGKAALPLKSDRTRSKRFLEIQKLREIKQEYDLKTALSLMKQMSSTKFVETAEAHFRLNIDPKYNDQQLRA
TVSLPKGTGKTVKIAVLAQGDKIDEAKAAGADIVGGEELIEQIKGGFMDFDKLIATSDMMAKVASLGRILGPRGLMPTPKAGTVTPNVAQAVEEFKKGKVEFRVDKTGIVHIPFGKLNFEEEDLLINLFATIKSVETNKPTGAKGVYWKSAHISSSMGPSIRLNIREMLDYKPPS
NA
0000000000000000000000000000000000000000000000000000000000000000000000000000000000000000000000000000000000000000000000000000000000000000000000000000000010000000001000000000
0000000000000000000000000000000000000000000000000000000000000001001000011001000100000000000000000000101011101011100000000000000000000000000000000000000000001100000000000000000
00
0000000000000000000000000000000000000000000000000000000000000000000000000000000000000000000000000000000000000000000000000000000000000000000000000000000010000000001000000000
0000000000000000000000000000000000000000000000000000000000000001001000011001000100000000000000000000101011101011100000000000000000000000000000000000000000001100000000000000000
00
0000000000000000000000000000000000000000000000000000000000000000000000000000000000000000000000000000000000000000000000000000000000000000000000000000000000000000000000000000000
0000000000000000000000000000000000000000000000000000000000000000000000000000000000000000000000000000000000000000000000000000000000000000000000000000000000000000000000000000000
00
0000000000000000000000000000000000000000000000000000000000000000000000000000000000000000000000000000000000000000000000000000000000000000000000000000000000000000000000000000000
0000000000000000000000000000000000000000000000000000000000000000000000000000000000000000000000000000000000000000000000000000000000000000000000000000000000000000000000000000000
00
0000000000000000000000000000000000000000000000000000000000000000000000000000000000000000000000000000000000000000000000000000000000000000000000000000000000000000000000000000000
0000000000000000000000000000000000000000000000000000000000000000000000000000000000000000000000000000000000000000000000000000000000000000000000000000000000000000000000000000000
00
0000000000000000000000000000000000000000000000000000000000000000000000000000000000000000000000000000000000000000000000000000000000000000000000000000000000000000000000000000000
0000000000000000000000000000000000000000000000000000000000000000000000000000000000000000000000000000000000000000000000000000000000000000000000000000000000000000000000000000000
00

0000000000000000000000000000000000000000000000000000000000000000000000000000000000000000000000000000000000000000000000000000000000000000000000000000000000000000000000000000
0000000000000000000000000000000000000000000000000000000000000000000000000000000000000000000000000000000000000000000000000000000000000000000000000000000000000000000000000000
00
>P0DJ56
MARGPAGKNRGVSKISKWYKADDEATPFHRRQLKKATATKLRNDIAPGTVLILLAGRFRGKRVVFLKQLKSGLLLVTGPYKVNGVPLKRVNQAYTLSTSTKVDLTGVNTAKFEDDYFGREKARKNHKNLFKAELTEEQKKKETERKNARKQDQQAVDTPLLAAVKKVEFLKNYLA
SKFTLNNNDRPHEMKF
0110100011101111000001100111111111110000000000000000001011101000000000001100000110110101111001000000000000000000000000100011011011100000000010011001100000000000000000000000000
1100011100000000
0110100011101111000001100111111111110000000000000000001011101000000000001100000110110101111001000000000000000000000000100011011011100000000010011001100000000000000000000000000
1100011100000000
0000000000000000000000000000000000000000000000000000000000000000000000000000000000000000000000000000000000000000000000000000000000000000000000000000000000000000000000000000
0000000000000000
0000000000000000000000000000000000000000000000000000000000000000000000000000000000000000000000000000000000000000000000000000000000000000000000000000000000000000000000000000
0000000000000000
0000000000000000000000000000000000000000000000000000000000000000000000000000000000000000000000000000000000000000000000000000000000000000000000000000000000000000000000000000
0000000000000000
0000000000000000000000000000000000000000000000000000000000000000000000000000000000000000000000000000000000000000000000000000000000000000000000000000000000000000000000000000
0000000000000000
0000000000000000000000000000000000000000000000000000000000000000000000000000000000000000000000000000000000000000000000000000000000000000000000000000000000000000000000000000
0000000000000000
>E6PBS6
MGISRDSKHKRRATGGRMPVHRKKRAFEKGRPISMTKLTTQSTTITEKRRIRPVRVRGGHLKFRALRLCEGNFSWGSENITRKTKILDVKYNATNNELVRTKTLVKNSIVEIDSTPFREWYKLHYGIDLGLKKDRTVLGNKEKSRHVQKRVKRTKAQALEKNIEEQFVSQRILAC
ITSRPGQSGRADGYILEGKELEFYIRKLQSKKK
0101101111111111111011111110111011000000000000001111101111110111011000010100001111100000000011110000001110000000000000000110000000000000000110111101110110000000000000000000000
010101110101000000000000000000000
0101101111111111111011111110111011000000000000001111101111110111011000010100001111100000000011110000001110000000000000000110000000000000000110111101110110000000000000000000000
010101110101000000000000000000000
0000000000000000000000000000000000000000000000000000000000000000000000000000000000000000000000000000000000000000000000000000000000000000000000000000000000000000000000000000
000000000000000000000000000000000
0000000000000000000000000000000000000000000000000000000000000000000000000000000000000000000000000000000000000000000000000000000000000000000000000000000000000000000000000000
000000000000000000000000000000000
0000000000000000000000000000000000000000000000000000000000000000000000000000000000000000000000000000000000000000000000000000000000000000000000000000000000000000000000000000
000000000000000000000000000000000
0000000000000000000000000000000000000000000000000000000000000000000000000000000000000000000000000000000000000000000000000000000000000000000000000000000000000000000000000000
000000000000000000000000000000000
0000000000000000000000000000000000000000000000000000000000000000000000000000000000000000000000000000000000000000000000000000000000000000000000000000000000000000000000000000
000000000000000000000000000000000
>P49166
MGKGTPSFGKRHNKSHTLCNRCGRRSFHVQKKTCSSCGYPAAKTRSYNWGAKAKRRHTTGTGRMRYLKHVSRRFKNGFQTGSASKASA
0111111111111111111111111011110000110000111100111111111111110001100100011100000000000000
0111111111111111111111111011110000110000111100111111111111110001100100011100000000000000
0000000000000000000000000000000000000000000000000000000000000000000000000000000000000000
0000000000000000000000000000000000000000000000000000000000000000000000000000000000000000
0000000000000000000000000000000000000000000000000000000000000000000000000000000000000000
0000000000000000000000000000000000000000000000000000000000000000000000000000000000000000
0000000000000000000000000000000000000000000000000000000000000000000000000000000000000000
>P0AFX0
MQLNITGNNVEITEALREFVTAKFAKLEQYFDRINQVYVVLKVEKVTHTSDATLHVNGGEIHASAEGQDMYAAIDGLIDKLARQLTKHKDKLKQH
00000100000000000000000001001101000000000001101000000000000011100000000000000001000100101110111
00000100000000000000000001001101000000000001101000000000000011100000000000000001000100101110111
00000000000000000000000000000000000000000000000000000000000000000000000000000000000000000000000
00000000000000000000000000000000000000000000000000000000000000000000000000000000000000000000000
00000000000000000000000000000000000000000000000000000000000000000000000000000000000000000000000
00000000000000000000000000000000000000000000000000000000000000000000000000000000000000000000000
00000000000000000000000000000000000000000000000000000000000000000000000000000000000000000000000
>P0CX84
MAGVKAYELRTKSKEQLASQLVDLKKELAELKVQKLSRPSLPKIKTVRKSIACVLTVINEQQREAVRQLYKGKKYQPKDLRAKKTRALRRALTKFEASQVTEKQRKKQIAFPQRKYAIKA
010111101100000000000000000000001000111110110110111111111111001001110000000000000000000000000000000000000000000000000000
010111101100000000000000000000001000111110110110111111111111001001110000000000000000000000000000000000000000000000000000
000000000000000000000000000000000000000000000000000000000000000000000000000000000000000000000000000000000000000000000000
000000000000000000000000000000000000000000000000000000000000000000000000000000000000000000000000000000000000000000000000
000000000000000000000000000000000000000000000000000000000000000000000000000000000000000000000000000000000000000000000000
000000000000000000000000000000000000000000000000000000000000000000000000000000000000000000000000000000000000000000000000
000000000000000000000000000000000000000000000000000000000000000000000000000000000000000000000000000000000000000000000000
>P0DJ55
MTSRPQIHVHDAKEANKQTATKLTLPAVFTAPIRTDIVHKVFTDLNKNRKQASGVKISTRGTAGMGHSAESWGTGRAVARIPRVGGSGTHRSGQAAFGNQCRKGRMFAPLKTYRRVHRRVNVNQKRHAVAAALAASALVPLVFARGHRISNVQELPYVFDDSVESYEKTKQAVAF
LKRVGAYDDVLRVAETKALRAGQGKLRNRRYKLRRGPLVVYGNEKSTLTRALRNIPGVDVCNVNRLNLLQLAPGGHVGRFIIWTESAFKKLNEIFGTYSTTGVQKSGYQLQRPLLANADIARIINSNEVQSVVKVAGTTETHERKKNPLTNNNALFKLNPAAKIVKEQAKKAAEA
SKAKRQATLKANRKAAKTHKKGSQAWIAAFNKANEEAIAKARQEDADFIAQGQEIKEGDE

00000000000000000000000000000000000000000000000000011101100000000000000000000000000000000000000000000000000000000100000000000000000000000000000000000000000000000000000000000000
00000000000000000000000011000000000000000000000000000000000000000000000000000000000000000000000000000000000000000000000000000000000000000000000000000000000000000000000000000
000000000000000000000000000000000000000000000000000000000000
00000000000000000000000000000000000000000000000000011101100000000000000000000000000000000000000000000000000000000100000000000000000000000000000000000000000000000000000000000000
00000000000000000000000011000000000000000000000000000000000000000000000000000000000000000000000000000000000000000000000000000000000000000000000000000000000000000000000000000
000000000000000000000000000000000000000000000000000000000000
0000000000000000000000000000000000000000000000000000000000000000000000000000000000000000000000000000000000000000000000000000000000000000000000000000000000000000000000000000000
0000000000000000000000000000000000000000000000000000000000000000000000000000000000000000000000000000000000000000000000000000000000000000000000000000000000000000000000000000000
000000000000000000000000000000000000000000000000000000000000
0000000000000000000000000000000000000000000000000000000000000000000000000000000000000000000000000000000000000000000000000000000000000000000000000000000000000000000000000000000
0000000000000000000000000000000000000000000000000000000000000000000000000000000000000000000000000000000000000000000000000000000000000000000000000000000000000000000000000000000
000000000000000000000000000000000000000000000000000000000000
0000000000000000000000000000000000000000000000000000000000000000000000000000000000000000000000000000000000000000000000000000000000000000000000000000000000000000000000000000000
0000000000000000000000000000000000000000000000000000000000000000000000000000000000000000000000000000000000000000000000000000000000000000000000000000000000000000000000000000000
000000000000000000000000000000000000000000000000000000000000
0000000000000000000000000000000000000000000000000000000000000000000000000000000000000000000000000000000000000000000000000000000000000000000000000000000000000000000000000000000
0000000000000000000000000000000000000000000000000000000000000000000000000000000000000000000000000000000000000000000000000000000000000000000000000000000000000000000000000000000
000000000000000000000000000000000000000000000000000000000000
0000000000000000000000000000000000000000000000000000000000000000000000000000000000000000000000000000000000000000000000000000000000000000000000000000000000000000000000000000000
0000000000000000000000000000000000000000000000000000000000000000000000000000000000000000000000000000000000000000000000000000000000000000000000000000000000000000000000000000000
000000000000000000000000000000000000000000000000000000000000
>P04802
MSQDENIVKAVEESAEPAQVILGEDGKPLSKKALKKLQKEQEKQRKKEERALQLEAEREAREKKAAAEDTAKDNYGKLPLIQSRDSDRTGQKRVKFVDLDEAKDSDKEVLFRARVHNTRQQGATLAFLTLRQQASLIQGLVKANKEGTISKNMVKWAGSLNLESIVLVRGIVKKV
DEPIKSATVQNLEIHITKIYTISETPEALPILLEDASRSEAEAEAAGLPVVNLDTRLDYRVIDLRTVTNQAIFRIQAGVCELFREYLATKKFTEVHTPKLLGAPSEGGSSVFEVTYFKGKAYLAQSPQFNKQQLIVADFERVYEIGPVFRAENSNTHRHMTEFTGLDMEMAFEEH
YHEVLDTLSELFVFIFSELPKRFAHEIELVRKQYPVEEFKLPKDGKMVRLTYKEGIEMLRAAGKEIGDFEDLSTENEKFLGKLVRDKYDTDFYILDKFPLEIRPFYTMPDPANPKYSNSYDFFMRGEEILSGAQRIHDHALLQERMKAHGLSPEDPGLKDYCDGFSYGCPPHAGG
GIGLERVVMFYLDLKNIRRASLFPRDPKRLRP
0000000000000000000000000000000000000000000000000000000000000000000000000000000000000000000000000000000000000000000000001011110110100000000001010100000000000010000000000000000000
0111110000001000000000000011010100100000000001111111011000000000000000000000000000000000000000100000000001111100000000000001101100000000000000000000101111101110010000000001000
0000000000000000000000000000000000000000000000000000000000000000000001110010000000000000000000000000000111000000000000000000011110000000000000000000000000000000000000010000
10000100000000001000010000010000
0000000000000000000000000000000000000000000000000000000000000000000000000000000000000000000000000000000000000000000000000000000000000000000000000000000000000000000000000000000
0000000000000000000000000000000000000000000000000000000000000000000000000000000000000000000000000000000000000000000000000000000000000000000000000000000000000000000000000000000
0000000000000000000000000000000000000000000000000000000000000000000000000000000000000000000000000000000000000000000000000000000000000000000000000000000000000000000000000000000
00000000000000000000000000000000
0000000000000000000000000000000000000000000000000000000000000000000000000000000000000000000000000000000000000000000000001011110110100000000001010100000000000010000000000000000000
0111110000001000000000000011010100100000000001111111011000000000000000000000000000000000000000100000000001111100000000000001101100000000000000000000101111101110010000000001000
0000000000000000000000000000000000000000000000000000000000000000000001110010000000000000000000000000000111000000000000000000011110000000000000000000000000000000000000010000
10000100000000001000010000010000
0000000000000000000000000000000000000000000000000000000000000000000000000000000000000000000000000000000000000000000000000000000000000000000000000000000000000000000000000000000
0000000000000000000000000000000000000000000000000000000000000000000000000000000000000000000000000000000000000000000000000000000000000000000000000000000000000000000000000000000
0000000000000000000000000000000000000000000000000000000000000000000000000000000000000000000000000000000000000000000000000000000000000000000000000000000000000000000000000000000
00000000000000000000000000000000
0000000000000000000000000000000000000000000000000000000000000000000000000000000000000000000000000000000000000000000000000000000000000000000000000000000000000000000000000000000
0000000000000000000000000000000000000000000000000000000000000000000000000000000000000000000000000000000000000000000000000000000000000000000000000000000000000000000000000000000
0000000000000000000000000000000000000000000000000000000000000000000000000000000000000000000000000000000000000000000000000000000000000000000000000000000000000000000000000000000
00000000000000000000000000000000
0000000000000000000000000000000000000000000000000000000000000000000000000000000000000000000000000000000000000000000000000000000000000000000000000000000000000000000000000000000
0000000000000000000000000000000000000000000000000000000000000000000000000000000000000000000000000000000000000000000000000000000000000000000000000000000000000000000000000000000
0000000000000000000000000000000000000000000000000000000000000000000000000000000000000000000000000000000000000000000000000000000000000000000000000000000000000000000000000000000
00000000000000000000000000000000
0000000000000000000000000000000000000000000000000000000000000000000000000000000000000000000000000000000000000000000000000000000000000000000000000000000000000000000000000000000
0000000000000000000000000000000000000000000000000000000000000000000000000000000000000000000000000000000000000000000000000000000000000000000000000000000000000000000000000000000
0000000000000000000000000000000000000000000000000000000000000000000000000000000000000000000000000000000000000000000000000000000000000000000000000000000000000000000000000000000
00000000000000000000000000000000
>P0DJ19
MAKFLKYGRVVILLQGRFAGKKAVIVKSSEDGTKDRKFGHVLVAGVERSPKKVTKRMGSKKIQKRTSVKPFIKYVNLNHIMPTRYSVKELCDFKELVKEDKIKNNAKSEVRDTLKKVFVEKYRTINPEEKSASHTKFFFSKLRF
000000000000001110000000000000000001010000000001001101111100100110101000111101100000000000000000000000000010001000000000000000000000000000000011
000000000000001110000000000000000001010000000001001101111100100110101000111101100000000000000000000000000010001000000000000000000000000000000011
000000000000000000000000000000000000000000000000000000000000000000000000000000000000000000000000000000000000000000000000000000000000000000000000
000000000000000000000000000000000000000000000000000000000000000000000000000000000000000000000000000000000000000000000000000000000000000000000000
000000000000000000000000000000000000000000000000000000000000000000000000000000000000000000000000000000000000000000000000000000000000000000000000
000000000000000000000000000000000000000000000000000000000000000000000000000000000000000000000000000000000000000000000000000000000000000000000000
000000000000000000000000000000000000000000000000000000000000000000000000000000000000000000000000000000000000000000000000000000000000000000000000
>P41567
MSAIQNLHSFDPFADASKGDDLLPAGTEDYIHIRIQQRNGRKTLTTVQGIADDYDKKKLVKAFKKKFACNGTVIEHPEYGEVIQLQGDQRKNICQFLVEIGLAKDDQLKVHGF
00000000000000000000000000000000000011001111000000000000001000100110011111000000000101110000000000000000000000000
00000000000000000000000000000000000011001111000000000000001000100110011111000000000101110000000000000000000000000
00000000000000000000000000000000000000000000000000000000000000000000000000000000000000000000000000000000000000000
00000000000000000000000000000000000000000000000000000000000000000000000000000000000000000000000000000000000000000

```
00000000000000000000000000000000000000000000000000000000000000000000000000000000000000000000000000000000000000000
00000000000000000000000000000000000000000000000000000000000000000000000000000000000000000000000000000000000000000
00000000000000000000000000000000000000000000000000000000000000000000000000000000000000000000000000000000000000000
>Q22DC6
MKTEKEEKIMKLTKGKLSQKRKEKIGRSQIKKEKEKIKLIKDKIQKINIKMDATKETKKGEEEVKKVRVRITLTCKNLKSVEKATSEIVSRAKKTEQVEVKGPVRMPTKTLVITVRKSPCGEGSKTWDRFEMRIYKRIIDLTCNVPDVKTITNFRIDPGVEIELTMTADQ
00000000000000000000000000000000000000000000000000000000000000000000010000000100010000000000000000001101111111000111111111110110100010111100000000001010000000000000000000
00000000000000000000000000000000000000000000000000000000000000000000010000000100010000000000000000001101111111000111111111110110100010111100000000001010000000000000000000
00000000000000000000000000000000000000000000000000000000000000000000000000000000000000000000000000000000000000000000000000000000000000000000000000000000000000000000000000
00000000000000000000000000000000000000000000000000000000000000000000000000000000000000000000000000000000000000000000000000000000000000000000000000000000000000000000000000
00000000000000000000000000000000000000000000000000000000000000000000000000000000000000000000000000000000000000000000000000000000000000000000000000000000000000000000000000
00000000000000000000000000000000000000000000000000000000000000000000000000000000000000000000000000000000000000000000000000000000000000000000000000000000000000000000000000
00000000000000000000000000000000000000000000000000000000000000000000000000000000000000000000000000000000000000000000000000000000000000000000000000000000000000000000000000
>Q6QAP6
MGPQGFYWSHPRKFGQGSRSCRVCSNRHGLIRKYGLNMCRQCFRQYAKDIGFIKLD
00000111111111111101001010111101111000011110110000000111
00000111111111111101001010111101111000011110110000000111
00000000000000000000000000000000000000000000000000000000
00000000000000000000000000000000000000000000000000000000
00000000000000000000000000000000000000000000000000000000
00000000000000000000000000000000000000000000000000000000
00000000000000000000000000000000000000000000000000000000
>I7MK25
MPKNKGRGGKNYRRGKNENETKRELVFKEEGMEYAQVIKMLGNGRLEVFCFDGKRRIGHICGRLKKKVWISMDDIVLVGLRDFQDDKCDILLKYLPDEVRNLKQYGEIDENIKINDKSGADQADVVFEDSDSDDKDDDDDDEDEQPTRQTNTRVNQMVESSDEEDNKETGNYEEQ
KYDQKHQNKKDDIDIDDI
0000000000000000000011100000000000000011111110000000000010001111100111000000001000000010000000000000000000000000000000000000000000000000000000000000000000000000000000000000000
000000000000000000
0000000000000000000011100000000000000011111110000000000010001111100111000000001000000010000000000000000000000000000000000000000000000000000000000000000000000000000000000000000
000000000000000000
0000000000000000000000000000000000000000000000000000000000000000000000000000000000000000000000000000000000000000000000000000000000000000000000000000000000000000000000000000000
000000000000000000
0000000000000000000000000000000000000000000000000000000000000000000000000000000000000000000000000000000000000000000000000000000000000000000000000000000000000000000000000000000
000000000000000000
0000000000000000000000000000000000000000000000000000000000000000000000000000000000000000000000000000000000000000000000000000000000000000000000000000000000000000000000000000000
000000000000000000
0000000000000000000000000000000000000000000000000000000000000000000000000000000000000000000000000000000000000000000000000000000000000000000000000000000000000000000000000000000
000000000000000000
0000000000000000000000000000000000000000000000000000000000000000000000000000000000000000000000000000000000000000000000000000000000000000000000000000000000000000000000000000000
000000000000000000
>E6PBT5
MGRVRTKTVKRAAKSLIEHYYSKLTNDFHFNKKILSEVAQVPSKRLRNKIAGFATHLMKRIQKGPVRGISLKVQEEERERRLDYVPEKSIIDIEKVTIDNETKEMLKKLGYQNIPGLVSASAPANKKRGQ
0111111101100000000000000001100110000000011110110010001001100101010000000000000000000000000000000000000000000000000000000000000000
0111111101100000000000000001100110000000011110110010001001100101010000000000000000000000000000000000000000000000000000000000000000
0000000000000000000000000000000000000000000000000000000000000000000000000000000000000000000000000000000000000000000000000000000000
0000000000000000000000000000000000000000000000000000000000000000000000000000000000000000000000000000000000000000000000000000000000
0000000000000000000000000000000000000000000000000000000000000000000000000000000000000000000000000000000000000000000000000000000000
0000000000000000000000000000000000000000000000000000000000000000000000000000000000000000000000000000000000000000000000000000000000
0000000000000000000000000000000000000000000000000000000000000000000000000000000000000000000000000000000000000000000000000000000000
>E6PBS7
MGKTYINTSKTYSTPRRPYEKERLDNEMKLIGTFGLKNKREVWRVQMILAKFRKAARELLTLDPKDPRRLFEGSALLRRMFKYGLLSEQERKLDYVLGLTIHKLMERRLQTRVFKLNLANSIHHSRVLIRQRHIKVGKNLVNVPSFMVRTDSEKSIDFASTSPLGGGREGRTKRK
NAKKSA
0111111111100111111100010000000000001111001100000000000000001000001100100010011000000000000000000000000000000000000000110010110011110000011111111010000000000000001000011111111
100000
0111111111100111111100010000000000001111001100000000000000001000001100100010011000000000000000000000000000000000000000110010110011110000011111111010000000000000001000011111111
100000
0000000000000000000000000000000000000000000000000000000000000000000000000000000000000000000000000000000000000000000000000000000000000000000000000000000000000000000000000000000
000000
0000000000000000000000000000000000000000000000000000000000000000000000000000000000000000000000000000000000000000000000000000000000000000000000000000000000000000000000000000000
000000
0000000000000000000000000000000000000000000000000000000000000000000000000000000000000000000000000000000000000000000000000000000000000000000000000000000000000000000000000000000
000000
0000000000000000000000000000000000000000000000000000000000000000000000000000000000000000000000000000000000000000000000000000000000000000000000000000000000000000000000000000000
000000
0000000000000000000000000000000000000000000000000000000000000000000000000000000000000000000000000000000000000000000000000000000000000000000000000000000000000000000000000000000
000000
>Q245F2
MARRCQFENSNDIGVFCKLTSAYCLVSVGASENFYSVFESELVPHIPVIHTSIGGTRIVGRVTCGNKNGLLVPNTCNDNELRNIRNSLPDNVRVRRIEEKLSALGNCVVANDYVALIHPDLDRESEEIIADTLGVEVFRTTIANNVLVGTYCVINNRGGLVHPLASVEELDELAN
LLQIPLCAGTINRGSDVIGAGLVVNDWAAFCGLDTTSTEISVVENIFKLNEMKDENMDNEMRKDFVMNLE
0000000100000000000000000000001010000000000000000000000000000000000000000000000000000000000000000000000000000000000000000000000000000000000000000000000000000000000000000
0000000000000000000000000000000000000000000000000000000000000000000000
```

0000000100000000000000000000001010000000000000000000000000000000000000000000000000000000000000000000000000000000000000000000000000000000000000000000000000000000000000000000000
0000000000000000000000000000000000000000000000000000000000000000000000
0000000000000000000000000000000000000000000000000000000000000000000000000000000000000000000000000000000000000000000000000000000000000000000000000000000000000000000000000000000
0000000000000000000000000000000000000000000000000000000000000000000000
0000000000000000000000000000000000000000000000000000000000000000000000000000000000000000000000000000000000000000000000000000000000000000000000000000000000000000000000000000000
0000000000000000000000000000000000000000000000000000000000000000000000
0000000000000000000000000000000000000000000000000000000000000000000000000000000000000000000000000000000000000000000000000000000000000000000000000000000000000000000000000000000
0000000000000000000000000000000000000000000000000000000000000000000000
0000000000000000000000000000000000000000000000000000000000000000000000000000000000000000000000000000000000000000000000000000000000000000000000000000000000000000000000000000000
0000000000000000000000000000000000000000000000000000000000000000000000
0000000000000000000000000000000000000000000000000000000000000000000000000000000000000000000000000000000000000000000000000000000000000000000000000000000000000000000000000000000
0000000000000000000000000000000000000000000000000000000000000000000000
>Q230V2
MIKYKGRKLVGINLKEKFWACGEESTKIIQREKYSIQIKVLSKVKQKIYKTKSIQIKMGKTYINTSKTYSTPRRPYEKERLDNEMKLIGTFGLKNKREVWRVQMILAKFRKAARELLTLDPKDPRRLFEGSALLRRMFKYGLLSEQERKLDYVLGLTIHKLMERRLQTRVFKLNL
ANSIHHSRVLIRQRHIKVGKNLVNVPSFMVRTDSEKSIDFASTSPLGGGREGRTKRKNAKKSA
0000000000000000000000000000000000000000000000000000000001111111111100101111100010000000000011110011000000000000010000001000001100100110010000000000000000000000000000000000000
011001001001111000001111101110100100000000010000011110111000000
0000000000000000000000000000000000000000000000000000000001111111111100101111100010000000000011110011000000000000010000001000001100100110010000000000000000000000000000000000000
011001001001111000001111101110100100000000010000011110111000000
0000000000000000000000000000000000000000000000000000000000000000000000000000000000000000000000000000000000000000000000000000000000000000000000000000000000000000000000000000000
000000000000000000000000000000000000000000000000000000000000000
0000000000000000000000000000000000000000000000000000000000000000000000000000000000000000000000000000000000000000000000000000000000000000000000000000000000000000000000000000000
000000000000000000000000000000000000000000000000000000000000000
0000000000000000000000000000000000000000000000000000000000000000000000000000000000000000000000000000000000000000000000000000000000000000000000000000000000000000000000000000000
000000000000000000000000000000000000000000000000000000000000000
0000000000000000000000000000000000000000000000000000000000000000000000000000000000000000000000000000000000000000000000000000000000000000000000000000000000000000000000000000000
000000000000000000000000000000000000000000000000000000000000000
0000000000000000000000000000000000000000000000000000000000000000000000000000000000000000000000000000000000000000000000000000000000000000000000000000000000000000000000000000000
000000000000000000000000000000000000000000000000000000000000000
>O13516
MPRAPRTYSKTYSTPKRPYESSRLDAELKLAGEFGLKNKKEIYRISFQLSKIRRAARDLLTRDEKDPKRLFEGNALIRRLVRVGVLSEDKKKLDYVLALKVEDFLERRLQTQVYKLGLAKSVHHARVLITQRHIAVGKQIVNIPSFMVRLDSEKHIDFAPTSPFGGARPGRVARR
NAARKAEASGEAADEADEADEE
0111111111110111111111111101100010111110001111001010100000000110011001110110111011001011110000000000000011011101000000011110111111110011111111111111111010000000001100011111101
1000000000000000000000
0111111111110111111111111101100010111110001111001010100000000110011001110110111011001011110000000000000011011101000000011110111111110011111111111111111010000000001100011111101
1000000000000000000000
0000000000000000000000000000000000000000000000000000000000000000000000000000000000000000000000000000000000000000000000000000000000000000000000000000000000000000000000000000000
0000000000000000000000
0000000000000000000000000000000000000000000000000000000000000000000000000000000000000000000000000000000000000000000000000000000000000000000000000000000000000000000000000000000
0000000000000000000000
0000000000000000000000000000000000000000000000000000000000000000000000000000000000000000000000000000000000000000000000000000000000000000000000000000000000000000000000000000000
0000000000000000000000
0000000000000000000000000000000000000000000000000000000000000000000000000000000000000000000000000000000000000000000000000000000000000000000000000000000000000000000000000000000
0000000000000000000000
0000000000000000000000000000000000000000000000000000000000000000000000000000000000000000000000000000000000000000000000000000000000000000000000000000000000000000000000000000000
0000000000000000000000
>P0AFW2
MKRQKRDRLERAHQRGYQAGIAGRSKEMCPYQTLNQRSQWLGGWREAMADRVVMA
0011000000101010000000000000000111001011000000000000000
0011000000101010000000000000000111001011000000000000000
0000000000000000000000000000000000000000000000000000000
0000000000000000000000000000000000000000000000000000000
0000000000000000000000000000000000000000000000000000000
0000000000000000000000000000000000000000000000000000000
0000000000000000000000000000000000000000000000000000000
>P39990
MSAPNPKAFPLADAALTQQILDVVQQAANLRQLKKGANEATKTLNRGISEFIIMAADCEPIEILLHLPLLCEDKNVPYVFVPSRVALGRACGVSRPVIAASITTNDASAIKTQIYAVKDKIETLLI
000000000000000000001000100111001111110010001000000000000110010000000000000000000001000000001010110000000000100110000000000000
000000000000000000000000000000000000000000000000000000000000000000000000000000000000000000000000000000000000000000000000000000
000000000000000000000000000000000000000000000000000000000000000000000000000000000000000000000000000000000000000000000000000000
000000000000000000001000100111001111110010001000000000000110010000000000000000000001000000001010110000000000100110000000000000
000000000000000000000000000000000000000000000000000000000000000000000000000000000000000000000000000000000000000000000000000000
000000000000000000000000000000000000000000000000000000000000000000000000000000000000000000000000000000000000000000000000000000
000000000000000000000000000000000000000000000000000000000000000000000000000000000000000000000000000000000000000000000000000000
>P42677
MPLAKDLLHPSPEEEKRKHKKKRLVQSPNSYFMDVKCPGCYKITTVFSHAQTVVLCVGCSTVLCQPTGGKARLTEGCSFRRKQH
000000000000000000000000000000000101000111100000000000000000000000000000000000111100
000000000000000000000000000000000000000000000000000000000000000000000000000000000000
000000000000000000000000000000000000000000000000000000000000000000000000000000000000

000000000000000000000000000000000000000000000000000000000000000000000000000000000000
000000000000000000000000000000000000000000000000000000000000000000000000000000000000
000000000000000000000000000000000101000111100000000000000000000000000000000000000111100
000000000000000000000000000000000000000000000000000000000000000000000000000000000000
>P0CX29
MGKGKPRGLNSARKLRVHRRNNRWAENNYKKRLLGTAFKSSPFGGSSHAKGIVLEKLGIESKQPNSAIRKCVRVQLIKNGKKVTAFVPNDGCLNFVDENDEVLLAGFGRKGKAKGDIPGVRFKVVKVSGVSLLALWKEKKEKPRS
0111111111101100011101111101011111010111111111111110000000000111111111001001110000000101111000000000001111111110111110001110010101101001100111100
0111111111101100011101111101011111010111111111111110000000000111111111001001110000000101111000000000001111111110111110001110010101101001100111100
0000000000000000000000000000000000000000000000000000000000000000000000000000000000000000000000000000000000000000000000000000000000000000000000000
0000000000000000000000000000000000000000000000000000000000000000000000000000000000000000000000000000000000000000000000000000000000000000000000000
0000000000000000000000000000000000000000000000000000000000000000000000000000000000000000000000000000000000000000000000000000000000000000000000000
0000000000000000000000000000000000000000000000000000000000000000000000000000000000000000000000000000000000000000000000000000000000000000000000000
0000000000000000000000000000000000000000000000000000000000000000000000000000000000000000000000000000000000000000000000000000000000000000000000000
>P62857
MDTSRVQPIKLARVTKVLGRTGSQGQCTQVRVEFMDDTSRSIIRNVKGPVREGDVLTLLESEREARRLR
000000000000000101000000000010101001111110100000000000000000000001100
000000000000000000000000000000000000000000000000000000000000000000000
000000000000000000000000000000000000000000000000000000000000000000000
000000000000000000000000000000000000000000000000000000000000000000000
000000000000000000000000000000000000000000000000000000000000000000000
000000000000000101000000000010101001111110100000000000000000000001100
000000000000000000000000000000000000000000000000000000000000000000000
>F1SVC5
MAASAFAGAVRAATGILRPLHILASSAYQNCAKNACLSSVLSSRHFSHIQTSALLSAPRLITSVRNLMCGPTAPVLNRVAPLLPHVLKPPVRTVTYFSSRKGKRKTVKAVIYRFLRLHSGLWLRRKAGYKKKLWKKTAARKRRLREFVFCNKTQSKLLDKMTTSFWKRRNWYADD
PYQKYQDRTNLKV
0000000000000000000000000000000000000000000000000000000000000000000000000000000000000000000110111110111101100110000100000101110111111100101111000000011110100010010110110000000
0000001000001
0000000000000000000000000000000000000000000000000000000000000000000000000000000000000000000110111110111101100110000100000101110111111100101111000000011110100010010110110000000
0000001000001
0000000000000000000000000000000000000000000000000000000000000000000000000000000000000000000000000000000000000000000000000000000000000000000000000000000000000000000000000000000
0000000000000
0000000000000000000000000000000000000000000000000000000000000000000000000000000000000000000000000000000000000000000000000000000000000000000000000000000000000000000000000000000
0000000000000
0000000000000000000000000000000000000000000000000000000000000000000000000000000000000000000000000000000000000000000000000000000000000000000000000000000000000000000000000000000
0000000000000
0000000000000000000000000000000000000000000000000000000000000000000000000000000000000000000000000000000000000000000000000000000000000000000000000000000000000000000000000000000
0000000000000
0000000000000000000000000000000000000000000000000000000000000000000000000000000000000000000000000000000000000000000000000000000000000000000000000000000000000000000000000000000
0000000000000
>P82923
MATGAVLPRLLGAGVRAAPRGRAAQRGRTLGSAAAAAAREPERDSDRSARILSEPLKHSDFFNVKELFSVRSLFNARVHLGHKAGCRHRFMEPYIFGSRLGQDIIDLEQTATHLQLALNFTAHVAFRGGIILFVSRARQFSHLIESTARSCGEYAHTRYFKGGLLTNAPLLLGAR
VRLPDLIIFLHTLNNVFEPHVAVRDAAKMSIPTVGVVDTNCNPCLITYPVPGNDDSPPAVQLFCQLFQTAVTRAKEKRRQLEALYRLQGAPGPHPANPAAPGAPSPGAQAQLGMGHSP
0000000000000000000000000000000000000000000000000000000000000000000000000000000000000000000000000000000000000000000000000000000000000000100000000000000000001111110011011000000
0000000000000000000000000000000000000000010000000000000000000000000000000000000000000000000000000000000000000000000000
0000000000000000000000000000000000000000000000000000000000000000000000000000000000000000000000000000000000000000000000000000000000000000100000000000000000001111110011011000000
0000000000000000000000000000000000000000010000000000000000000000000000000000000000000000000000000000000000000000000000
0000000000000000000000000000000000000000000000000000000000000000000000000000000000000000000000000000000000000000000000000000000000000000000000000000000000000000000000000000000
0000000000000000000000000000000000000000000000000000000000000000000000000000000000000000000000000000000000000000000000
0000000000000000000000000000000000000000000000000000000000000000000000000000000000000000000000000000000000000000000000000000000000000000000000000000000000000000000000000000000
0000000000000000000000000000000000000000000000000000000000000000000000000000000000000000000000000000000000000000000000
0000000000000000000000000000000000000000000000000000000000000000000000000000000000000000000000000000000000000000000000000000000000000000000000000000000000000000000000000000000
0000000000000000000000000000000000000000000000000000000000000000000000000000000000000000000000000000000000000000000000
0000000000000000000000000000000000000000000000000000000000000000000000000000000000000000000000000000000000000000000000000000000000000000000000000000000000000000000000000000000
0000000000000000000000000000000000000000000000000000000000000000000000000000000000000000000000000000000000000000000000
0000000000000000000000000000000000000000000000000000000000000000000000000000000000000000000000000000000000000000000000000000000000000000000000000000000000000000000000000000000
0000000000000000000000000000000000000000000000000000000000000000000000000000000000000000000000000000000000000000000000
>Q08745
MLMPKEDRNKIHQYLFQEGVVVAKKDFNQAKHEEIDTKNLYVIKALQSLTSKGYVKTQFSWQYYYYTLTEEGVEYLREYLNLPEHIVPGTYIQERNPTQRPQRRY
111010010000000000000000000100000000000100010011001100000100001000000000000000000000000000000000000000000
111010010000000000000000000100000000000100010011001100000100001000000000000000000000000000000000000000000
000000000000000000000000000000000000000000000000000000000000000000000000000000000000000000000000000000000
000000000000000000000000000000000000000000000000000000000000000000000000000000000000000000000000000000000
000000000000000000000000000000000000000000000000000000000000000000000000000000000000000000000000000000000
000000000000000000000000000000000000000000000000000000000000000000000000000000000000000000000000000000000
000000000000000000000000000000000000000000000000000000000000000000000000000000000000000000000000000000000
>W5IDC1
MAFLARSVGRLLDPVSRSAALVGGRWLQPQAWLGFPDTWGLPAMQQTRGKARGNEYQPSNIKRKHKHGWVRRLRTPTGVQVILRRMHKGRKSLSH
00000000000000000000000000000000000000000000000001111111111111111111110111001000100110011110001
00000000000000000000000000000000000000000000000001111111111111111111110111001000100110011110001

00000000000000000000000000000000000000000000000000000000000000000000000000000000000000000000000
00000000000000000000000000000000000000000000000000000000000000000000000000000000000000000000000
00000000000000000000000000000000000000000000000000000000000000000000000000000000000000000000000
00000000000000000000000000000000000000000000000000000000000000000000000000000000000000000000000
00000000000000000000000000000000000000000000000000000000000000000000000000000000000000000000000
>E1BBB4
MLRAAWRALSSIRTQAVTQPPVLGLPGGGCAKLLSVQRDLPSSLILQAARGYATQKPVQQSQEDDPPPSMLLKDYQNVPGIEKVDDVVKRLISLEMANKKEMLKIKKEQLMSKVVENPKDTSSLEAQIVALTVKIRSYEEHMQKHQKDKAHKRYLLMSIDKRQKMLKNLRKTNYP
VFEKTCKELGIEYTFPPPYHRKIHRRLATKKALCIRVFQEVQKLKKQKRALKTAAAAAQKQGQRNPESPSEARSEAIKETQ
000000000000000000000000000000000000000000000000000000000000000000000000000000000000000000000000000000000110010000000000000000100110010001000000101011001100100110001000000000
000000001000000000110011111110000000000000000000000000000000000000000000000000000
000000000000000000000000000000000000000000000000000000000000000000000000000000000000000000000000000000000110010000000000000000100110010001000000101011001100100110001000000000
000000001000000000110011111110000000000000000000000000000000000000000000000000000
0000000000000000000000000000000000000000000000000000000000000000000000000000000000000000000000000000000000000000000000000000000000000000000000000000000000000000000000000000000
000000000000000000000000000000000000000000000000000000000000000000000000000000000
0000000000000000000000000000000000000000000000000000000000000000000000000000000000000000000000000000000000000000000000000000000000000000000000000000000000000000000000000000000
000000000000000000000000000000000000000000000000000000000000000000000000000000000
0000000000000000000000000000000000000000000000000000000000000000000000000000000000000000000000000000000000000000000000000000000000000000000000000000000000000000000000000000000
000000000000000000000000000000000000000000000000000000000000000000000000000000000
0000000000000000000000000000000000000000000000000000000000000000000000000000000000000000000000000000000000000000000000000000000000000000000000000000000000000000000000000000000
000000000000000000000000000000000000000000000000000000000000000000000000000000000
0000000000000000000000000000000000000000000000000000000000000000000000000000000000000000000000000000000000000000000000000000000000000000000000000000000000000000000000000000000
000000000000000000000000000000000000000000000000000000000000000000000000000000000
>F1RWJ0
MAAVVAGMLRGGLLPQAGRLPTFQTVRYGSKAVTRHRRVMHFERQKLMAVTEYIPPKPTINPRCLPPPPTPPQEETGLVRLLRREIAAVFRDNRMIAVCQHVALSAEDKLLLRHQLRKHKILMKIFPNQVLKPFLEESKYQNLLPLFVGHNLLLVSEEPKVKEMVRILKGIPFLP
LLGGCIDDTILSRQGFINYSKLPSLALVQGELVGGLTFLTAQTYSMLQHQPRQLTALLDQYVKQQQEGHPAVPASGQPEPPDPVPDP
0000000000000000000000000000111011100100100001000000000000000010000000000000000000000000000000000000000000100010010000000000010110000000000000000000101100000000000000000000000
000000000000000000000000000000000000000000000000000000000000000000000000000000000000000
0000000000000000000000000000111011100100100001000000000000000010000000000000000000000000000000000000000000100010010000000000010110000000000000000000101100000000000000000000000
000000000000000000000000000000000000000000000000000000000000000000000000000000000000000
0000000000000000000000000000000000000000000000000000000000000000000000000000000000000000000000000000000000000000000000000000000000000000000000000000000000000000000000000000000
000000000000000000000000000000000000000000000000000000000000000000000000000000000000000
0000000000000000000000000000000000000000000000000000000000000000000000000000000000000000000000000000000000000000000000000000000000000000000000000000000000000000000000000000000
000000000000000000000000000000000000000000000000000000000000000000000000000000000000000
0000000000000000000000000000000000000000000000000000000000000000000000000000000000000000000000000000000000000000000000000000000000000000000000000000000000000000000000000000000
000000000000000000000000000000000000000000000000000000000000000000000000000000000000000
0000000000000000000000000000000000000000000000000000000000000000000000000000000000000000000000000000000000000000000000000000000000000000000000000000000000000000000000000000000
000000000000000000000000000000000000000000000000000000000000000000000000000000000000000
0000000000000000000000000000000000000000000000000000000000000000000000000000000000000000000000000000000000000000000000000000000000000000000000000000000000000000000000000000000
000000000000000000000000000000000000000000000000000000000000000000000000000000000000000
>I3LNJ0
MGVVVSTQRAGMAASITRGSWAAVGLGQSFPPTRALLPAPASVICRTLAGSGRQQVTGSSEPGAFQPPPKPVIVDKRRPQRPETRFLSPEFIPPRGRTNPLKFQIERKDMLERRKILHIPEFYVGSILRVTTADPYASAKTSQFLGICIQRSGSGLGATFILRNTIEGQGVEICF
ELYNPRIQEIQVVKLEKRLDNSLLYLRDALPEYSTFDVNMKPITQEFNQEVPVNQLKVKMKPKPWSKRWERPKFNIKGIRFDLCLTEEQMREAQKWSQPWLEFDMMREYDTSKIEAAIWDEIEASKNS
0000000000000000000000000000000000000000000000000000000000000000000000000000000000000000000000111000100000000000000000000000000000000000000000000000000110001010001000000000000
00000000000000000000000000000000000000000000000000000000000000011011001010000000000000000000000100001000001000000000000000000000
0000000000000000000000000000000000000000000000000000000000000000000000000000000000000000000000111000100000000000000000000000000000000000000000000000000110001010001000000000000
00000000000000000000000000000000000000000000000000000000000000011011001010000000000000000000000100001000001000000000000000000000
0000000000000000000000000000000000000000000000000000000000000000000000000000000000000000000000000000000000000000000000000000000000000000000000000000000000000000000000000000000
00000000000000000000000000000000000000000000000000000000000000000000000000000000000000000000000000000000000000000000000000000000
0000000000000000000000000000000000000000000000000000000000000000000000000000000000000000000000000000000000000000000000000000000000000000000000000000000000000000000000000000000
00000000000000000000000000000000000000000000000000000000000000000000000000000000000000000000000000000000000000000000000000000000
0000000000000000000000000000000000000000000000000000000000000000000000000000000000000000000000000000000000000000000000000000000000000000000000000000000000000000000000000000000
00000000000000000000000000000000000000000000000000000000000000000000000000000000000000000000000000000000000000000000000000000000
0000000000000000000000000000000000000000000000000000000000000000000000000000000000000000000000000000000000000000000000000000000000000000000000000000000000000000000000000000000
00000000000000000000000000000000000000000000000000000000000000000000000000000000000000000000000000000000000000000000000000000000
0000000000000000000000000000000000000000000000000000000000000000000000000000000000000000000000000000000000000000000000000000000000000000000000000000000000000000000000000000000
00000000000000000000000000000000000000000000000000000000000000000000000000000000000000000000000000000000000000000000000000000000
>P32905
MSLPATFDLTPEDAQLLLAANTHLGARNVQVHQEPYVFNARPDGVHVINVGKTWEKLVLAARIIAAIPNPEDVVAISSRTFGQRAVLKFAAHTGATPIAGRFTPGSFTNYITRSFKEPRLVIVTDPRSDAQAIKEASYVNIPVIALTDLDSPSEFVDVAIPCNNRGKHSIGLIWY
LLAREVLRLRGALVDRTQPWSIMPDLYFYRDPEEVEQQVAEEATTEEAGEEEAKEEVTEEQAEATEWAEENADNVEW
0000000000000000000000000001010100000000000000000000000000000000000000000000000000000000000000000000101110011011100000000000100100010011001000000000000000000000000000000000000
00000000000000000000000000000000000000000000000000000000000000000000000000000
0000000000000000000000000001010100000000000000000000000000000000000000000000000000000000000000000000101110011011100000000000100100010011001000000000000000000000000000000000000
00000000000000000000000000000000000000000000000000000000000000000000000000000
0000000000000000000000000000000000000000000000000000000000000000000000000000000000000000000000000000000000000000000000000000000000000000000000000000000000000000000000000000000
00000000000000000000000000000000000000000000000000000000000000000000000000000
0000000000000000000000000000000000000000000000000000000000000000000000000000000000000000000000000000000000000000000000000000000000000000000000000000000000000000000000000000000
00000000000000000000000000000000000000000000000000000000000000000000000000000
0000000000000000000000000000000000000000000000000000000000000000000000000000000000000000000000000000000000000000000000000000000000000000000000000000000000000000000000000000000
00000000000000000000000000000000000000000000000000000000000000000000000000000

0000000000000000000000000000000000000000000000000000000000000000000000000000000000000000000000000000000000000000000000000000000000000000000000000000000000000000000000000000000
00000000000000000000000000000000000000000000000000000000000000000000000000000
0000000000000000000000000000000000000000000000000000000000000000000000000000000000000000000000000000000000000000000000000000000000000000000000000000000000000000000000000000000
00000000000000000000000000000000000000000000000000000000000000000000000000000
>Q01855
MSQAVNAKKRVFKTHSYRGVDLEKLLEMSTEDFVKLAPARVRRRFARGMTSKPAGFMKKLRAAKLAAPENEKPAPVRTHMRNMIIVPEMIGSVVGIYNGKAFNQVEIRPEMLGHYLGEFSITYTPVRHGRAGATTSRFIPLK
0000000000001111110000000000000000000111011111111011111111000000000000010001111111110000000001111111110000000000011111111111111100000000000000
0000000000001111110000000000000000000111011111111011111111000000000000010001111111110000000001111111110000000000011111111111111100000000000000
0000000000000000000000000000000000000000000000000000000000000000000000000000000000000000000000000000000000000000000000000000000000000000000000
0000000000000000000000000000000000000000000000000000000000000000000000000000000000000000000000000000000000000000000000000000000000000000000000
0000000000000000000000000000000000000000000000000000000000000000000000000000000000000000000000000000000000000000000000000000000000000000000000
0000000000000000000000000000000000000000000000000000000000000000000000000000000000000000000000000000000000000000000000000000000000000000000000
0000000000000000000000000000000000000000000000000000000000000000000000000000000000000000000000000000000000000000000000000000000000000000000000
>F1RRN2
MALRVLTRALSSLSLTPRIAVAPGLNLLPAVQVTNNVLLTLPSGLMSLPCRPILTSVALSATSVSWKSRTKYTVMPVKMRKSGGRNHTGQIQVHGIGGGHKQRYRMIDFLRFRPEHESKPGPFEEKVIAVRYDPCRSADIALVAGGNRKRWIIATENMKAGDTVLNSDHIGRMAV
AAREGDAHPLGALPVGTLINNVESEPGRGAQYIRAAGTCGVLLRKVNGTAIIQLPSKRQMQVLETCIATVGRVSNVDHNKRVIGKAGRNRWLGKRPNSGLWQRKGGWAGRKIRPLPPMKSYVKLPSAAAQS
0000000000000000000000000000000000000000000000000000000000001000111010100011011111111011111111011101111110000000000000000000000000110111100001000000101000000000000000000010000
00000000000000000000000000000000000000000000000000000111010100000000000000010110101111111111000111111111111111101000001010000000000
0000000000000000000000000000000000000000000000000000000000001000111010100011011111111011111111011101111110000000000000000000000000110111100001000000101000000000000000000010000
00000000000000000000000000000000000000000000000000000111010100000000000000010110101111111111000111111111111111101000001010000000000
0000000000000000000000000000000000000000000000000000000000000000000000000000000000000000000000000000000000000000000000000000000000000000000000000000000000000000000000000000000
00000000000000000000000000000000000000000000000000000000000000000000000000000000000000000000000000000000000000000000000000000000000
0000000000000000000000000000000000000000000000000000000000000000000000000000000000000000000000000000000000000000000000000000000000000000000000000000000000000000000000000000000
00000000000000000000000000000000000000000000000000000000000000000000000000000000000000000000000000000000000000000000000000000000000
0000000000000000000000000000000000000000000000000000000000000000000000000000000000000000000000000000000000000000000000000000000000000000000000000000000000000000000000000000000
00000000000000000000000000000000000000000000000000000000000000000000000000000000000000000000000000000000000000000000000000000000000
0000000000000000000000000000000000000000000000000000000000000000000000000000000000000000000000000000000000000000000000000000000000000000000000000000000000000000000000000000000
00000000000000000000000000000000000000000000000000000000000000000000000000000000000000000000000000000000000000000000000000000000000
0000000000000000000000000000000000000000000000000000000000000000000000000000000000000000000000000000000000000000000000000000000000000000000000000000000000000000000000000000000
00000000000000000000000000000000000000000000000000000000000000000000000000000000000000000000000000000000000000000000000000000000000
>F1SB64
MVLRSSVPLKQVAHARLHFLVPSRKGCRAAALSTSCKPATNPETDPVENEAVAPEFTNRNPRNLELLAVARKERGWGTVWPSREFWHRLRVIRTQHHIEALVEHRNGQVVVSASTREWAIKKHLYSTRNVVACESVGRVLAERCLEAGINFMVYHPTPWEAASDSIKRLQHAMTE
GGVVLREPRRIYE
0000000000000000000000000000000000000000000000000000000000000000000000000100000000000000000011001000000000000000000100000000101110000000000000000000000000000001001010000000000
0000000010001
0000000000000000000000000000000000000000000000000000000000000000000000000100000000000000000000000000000000000000000000000000000000000000000000000000000000000000000000000000000
0000000010001
0000000000000000000000000000000000000000000000000000000000000000000000000000000000000000000000000000000000000000000000000000000000000000000000000000000000000000000000000000000
0000000000000
0000000000000000000000000000000000000000000000000000000000000000000000000000000000000000000000000000000000000000000000000000000000000000000000000000000000000000000000000000000
0000000000000
0000000000000000000000000000000000000000000000000000000000000000000000000000000000000000000011001000000000000000000100000000101110000000000000000000000000000001001010000000000
0000000000000
0000000000000000000000000000000000000000000000000000000000000000000000000000000000000000000000000000000000000000000000000000000000000000000000000000000000000000000000000000000
0000000000000
0000000000000000000000000000000000000000000000000000000000000000000000000000000000000000000000000000000000000000000000000000000000000000000000000000000000000000000000000000000
0000000000000
>F1RQV7
MRRRRTTGRVGGKAALGQWGLQRDIPGRRRRSLLESSLPERGLPSRDCMAFCTGLRGPFAQVSRALSQRCFSTTGSLSAIQKMTRVRVVDNSALGTTPYHRPPRCIHVYNKTGVGKVGDRILLAIRGQKKKALIVGHRMPGPRMTPRFDSNNVVLLEDNGNPVGTRIKTPIPSSL
RQREGEFSKVLAIAQNFV
0000000000000000000000000000000000000000000000000000000000000000000000000000001011111000000000000001100101101011000000000100011010000001110001000000000000000000000000000000000
000000000000000000
0000000000000000000000000000000000000000000000000000000000000000000000000000001011111000000000000001100101101011000000000100011010000001110001000000000000000000000000000000000
000000000000000000
0000000000000000000000000000000000000000000000000000000000000000000000000000000000000000000000000000000000000000000000000000000000000000000000000000000000000000000000000000000
000000000000000000
0000000000000000000000000000000000000000000000000000000000000000000000000000000000000000000000000000000000000000000000000000000000000000000000000000000000000000000000000000000
000000000000000000
0000000000000000000000000000000000000000000000000000000000000000000000000000000000000000000000000000000000000000000000000000000000000000000000000000000000000000000000000000000
000000000000000000
0000000000000000000000000000000000000000000000000000000000000000000000000000000000000000000000000000000000000000000000000000000000000000000000000000000000000000000000000000000
000000000000000000
0000000000000000000000000000000000000000000000000000000000000000000000000000000000000000000000000000000000000000000000000000000000000000000000000000000000000000000000000000000
000000000000000000
>F1RMQ4
MRLSVAAAISHGRVFRRLGLGPESRIHLLQNLLTGLVRHERIEASWARVDELRGYAEKLIDYGKLGDTNERAMRMADFWLTEKDLIPKLFQVLAPRFQGQNGGYTRMLQIPNRNEQDRAKMAVIEYKGNCLPPLPLPRRDSNLTLLNQLLQGLRQDQEASSHTAQTPAV
0000000011111111111111011011011100100010011011110010000000000000000000000000000001100000000000000000000000000000000000011111000000000000000000000000000000000000000000000
0000000011111111111111011011011100100010011011110010000000000000000000000000000001100000000000000000000000000000000000011111000000000000000000000000000000000000000000000
0000000000000000000000000000000000000000000000000000000000000000000000000000000000000000000000000000000000000000000000000000000000000000000000000000000000000000000000000

0000000000000000000000000000000000000000000000000000000000000000000000000000000000000000000000000000000000000000000000000000000000000000000000000000000000000000000000000
0000000000000000000000000000000000000000000000000000000000000000000000000000000000000000000000000000000000000000000000000000000000000000000000000000000000000000000000000
0000000000000000000000000000000000000000000000000000000000000000000000000000000000000000000000000000000000000000000000000000000000000000000000000000000000000000000000000
0000000000000000000000000000000000000000000000000000000000000000000000000000000000000000000000000000000000000000000000000000000000000000000000000000000000000000000000000
>P38701
MSDFQKEKVEEQEQQQQIIKIRITLTSTKVKQLENVSSNIVKNAEQHNLVKKGPVRLPTKVLKISTRKTPNGEGSKTWETYEMRIHKRYIDLEAPVQIVKRITQITIEPGVDVEVVVASN
000000000111111110000010000000100010000000000000010111111111111011111111111111010101111110101000000000001000000000000000
000000000111111110000010000000100010000000000000010111111111111011111111111111010101111110101000000000001000000000000000
000000000000000000000000000000000000000000000000000000000000000000000000000000000000000000000000000000000000000000000000
000000000000000000000000000000000000000000000000000000000000000000000000000000000000000000000000000000000000000000000000
000000000000000000000000000000000000000000000000000000000000000000000000000000000000000000000000000000000000000000000000
000000000000000000000000000000000000000000000000000000000000000000000000000000000000000000000000000000000000000000000000
000000000000000000000000000000000000000000000000000000000000000000000000000000000000000000000000000000000000000000000000
>P82916
MSVVRSSVHAKWIVGKVIGTAMQKTAKVRVTRLVLDPYLLKYFNKRKTYFAHDALQQCTVGDIVLLKALPVPRTKHVKHELAEIVFKVGQVVDPVTGKRCAGTTYLESPVDLETTPLAKNLEELSLSTTQ
1110110100111000000000000000000101111011111100001110000000000000000000000011111010000000000000000000000000000000000000000000001111
1110110100111000000000000000000101111011111100001110000000000000000000000011111010000000000000000000000000000000000000000000001111
0000000000000000000000000000000000000000000000000000000000000000000000000000000000000000000000000000000000000000000000000000000000
0000000000000000000000000000000000000000000000000000000000000000000000000000000000000000000000000000000000000000000000000000000000
0000000000000000000000000000000000000000000000000000000000000000000000000000000000000000000000000000000000000000000000000000000000
0000000000000000000000000000000000000000000000000000000000000000000000000000000000000000000000000000000000000000000000000000000000
0000000000000000000000000000000000000000000000000000000000000000000000000000000000000000000000000000000000000000000000000000000000
>Q3T040
MAAPTAKVSRGWSGLALGVRIAVLRLPGLTQVRWSRYGPEYQDPQIDKEYYRKPLAQLTEEETYERELRKTQVIKAAPATKTSSVFEDPVISKFTNMMMKGGNKILARSLMTQTLEAVKRKQFEKYHAASAEEQATVERNPYTIFHQALKNCEPVIGLVPILKGGHFYQVPVPLA
ERRRRFLAMKWMITECREKKPRRMLMPEKLSQELLEAFCNRGPVIKRKHDMHKMAEANRALAHYRWW
0000000000000000000000000000000000000011111000000000000000000000000000000000000000000000000000000000000000000000000000000000000000000000000000000000000000011101101000000000
0000000000000000101111011000000100010000101001000000000000000000000
0000000000000000000000000000000000000011111000000000000000000000000000000000000000000000000000000000000000000000000000000000000000000000000000000000000000011101101000000000
0000000000000000101111011000000100010000101001000000000000000000000
0000000000000000000000000000000000000000000000000000000000000000000000000000000000000000000000000000000000000000000000000000000000000000000000000000000000000000000000000000000
0000000000000000000000000000000000000000000000000000000000000000000
0000000000000000000000000000000000000000000000000000000000000000000000000000000000000000000000000000000000000000000000000000000000000000000000000000000000000000000000000000000
0000000000000000000000000000000000000000000000000000000000000000000
0000000000000000000000000000000000000000000000000000000000000000000000000000000000000000000000000000000000000000000000000000000000000000000000000000000000000000000000000000000
0000000000000000000000000000000000000000000000000000000000000000000
0000000000000000000000000000000000000000000000000000000000000000000000000000000000000000000000000000000000000000000000000000000000000000000000000000000000000000000000000000000
0000000000000000000000000000000000000000000000000000000000000000000
0000000000000000000000000000000000000000000000000000000000000000000000000000000000000000000000000000000000000000000000000000000000000000000000000000000000000000000000000000000
0000000000000000000000000000000000000000000000000000000000000000000
>P05759
MQIFVKTLTGKTITLEVESSDTIDNVKSKIQDKEGIPPDQQRLIFAGKQLEDGRTLSDYNIQKESTLHLVLRLRGGGKKRKKKVYTTPKKIKHKHKKVKLAVLSYYKVDAEGKVTKLRRECSNPTCGAGVFLANHKDRLYCGKCHSVYKVNA
00000000000000000000000000000000000000000000000000000000000000000000000000000000000001011001110000101001000000000000010000000000011111100101111011001000
00000000000000000000000000000000000000000000000000000000000000000000000000000000000001011001110000101001000000000000010000000000011111100101111011001000
00000000000000000000000000000000000000000000000000000000000000000000000000000000000000000000000000000000000000000000000000000000000000000000000000000000
00000000000000000000000000000000000000000000000000000000000000000000000000000000000000000000000000000000000000000000000000000000000000000000000000000000
00000000000000000000000000000000000000000000000000000000000000000000000000000000000000000000000000000000000000000000000000000000000000000000000000000000
00000000000000000000000000000000000000000000000000000000000000000000000000000000000000000000000000000000000000000000000000000000000000000000000000000000
00000000000000000000000000000000000000000000000000000000000000000000000000000000000000000000000000000000000000000000000000000000000000000000000000000000
>Q29RU1
MSWSGLLRGLSMSLNYGLALAPRPWGTRPMATLNQLHRRGPPKFPPSKPGPTEGRPQLKGVVLRTFIRKPKKPNSANRKCCRVLSTGREAVCFIPGEGHNLQEHHVVLVQGGRTQDLPGVKLKVVRGKYDCGHVQKKK
000000000000000000000000000000100001001011011111100110111101000000000100101101100000010000000101111000000000000111111111110001111000000111
000000000000000000000000000000100001001011011111100110111101000000000100101101100000010000000101111000000000000111111111110001111000000111
000000000000000000000000000000000000000000000000000000000000000000000000000000000000000000000000000000000000000000000000000000000000000000
000000000000000000000000000000000000000000000000000000000000000000000000000000000000000000000000000000000000000000000000000000000000000000
000000000000000000000000000000000000000000000000000000000000000000000000000000000000000000000000000000000000000000000000000000000000000000
000000000000000000000000000000000000000000000000000000000000000000000000000000000000000000000000000000000000000000000000000000000000000000
000000000000000000000000000000000000000000000000000000000000000000000000000000000000000000000000000000000000000000000000000000000000000000
>P0CX31
MSDAVTIRTRKVISNPLLARKQFVVDVLHPNRANVSKDELREKLAEVYKAEKDAVSVFGFRTQFGGGKSVGFGLVYNSVAEAKKFEPTYRLVRYGLAEKVEKASRQQRKQKKNRDKKIFGTGKRLAKKVARRNAD
000000011111000000000000000000111111000000000111000000000111111111100000000000000000000010001000000000001110110110011011101100110011001
000000011111000000000000000000111111000000000111000000000111111111100000000000000000000010001000000000001110110110011011101100110011001
000000000000000000000000000000000000000000000000000000000000000000000000000000000000000000000000000000000000000000000000000000000000000
000000000000000000000000000000000000000000000000000000000000000000000000000000000000000000000000000000000000000000000000000000000000000
000000000000000000000000000000000000000000000000000000000000000000000000000000000000000000000000000000000000000000000000000000000000000
000000000000000000000000000000000000000000000000000000000000000000000000000000000000000000000000000000000000000000000000000000000000000
000000000000000000000000000000000000000000000000000000000000000000000000000000000000000000000000000000000000000000000000000000000000000
>Q6B860
MATSMLGSLLRIVRQVVPSSASGQARSYYVDWRMLRDVKRRKMAYEYADERLRINSLRKNTILPKHLQEVADEEIAALPRDSCPVRIRNRCVMTSRPRGVKRRWRLSRIVFRHLADHGQLSGIQRAIW
00000000000000011110001110111011111100110010000000000000000000000000000000110111100100101000000011111000011111000000000000000010

00000000000000011110001110111011111100110010000000000000000000000000000000110111100100101000000011111000011111000000000000000000010
00000000000000000000000000000000000000000000000000000000000000000000000000000000000000000000000000000000000000000000000000000000
00000000000000000000000000000000000000000000000000000000000000000000000000000000000000000000000000000000000000000000000000000000
00000000000000000000000000000000000000000000000000000000000000000000000000000000000000000000000000000000000000000000000000000000
00000000000000000000000000000000000000000000000000000000000000000000000000000000000000000000000000000000000000000000000000000000
00000000000000000000000000000000000000000000000000000000000000000000000000000000000000000000000000000000000000000000000000000000
>P82915
MVQLTTVLCKAYRGGHLTIRLALGGCTNRPFYRIVAAHNKCPRDGRFVEQLGSYDPMPNSHGEKLVALNLDRIRHWIGCGAHLSKPVEKLLGLSGFFPLHPMVITNAERLRRKRAQEVLLAAQKTDTEATETKEN
000000000000000100011010011000000000001111110000000000000000000000000000000011111000000000000010000000000000000000000011001000111001000
000000000000000100011010011000000000001111110000000000000000000000000000000011111000000000000010000000000000000000000011001000111001000
000000000000000000000000000000000000000000000000000000000000000000000000000000000000000000000000000000000000000000000000000000000000000
000000000000000000000000000000000000000000000000000000000000000000000000000000000000000000000000000000000000000000000000000000000000000
000000000000000000000000000000000000000000000000000000000000000000000000000000000000000000000000000000000000000000000000000000000000000
000000000000000000000000000000000000000000000000000000000000000000000000000000000000000000000000000000000000000000000000000000000000000
000000000000000000000000000000000000000000000000000000000000000000000000000000000000000000000000000000000000000000000000000000000000000
>P82931
MPRYELALILKAMQRPETAAALKRTLEALMDRGAVVRSLENLGERTLPYKMSAHSQRHTRGGYFLVDFYAPTTTVASIMEHLSRDIDVIRPNVVKHPLTQEVKECEGIVPVPLEEKLYSTKKRK
1001000000000000000000000000000000000000000000000110111000000000000001000000000000000011101010000000000000000000000000000000
1001000000000000000000000000000000000000000000000110111000000000000001000000000000000011101010000000000000000000000000000000
0000000000000000000000000000000000000000000000000000000000000000000000000000000000000000000000000000000000000000000000000000
0000000000000000000000000000000000000000000000000000000000000000000000000000000000000000000000000000000000000000000000000000
0000000000000000000000000000000000000000000000000000000000000000000000000000000000000000000000000000000000000000000000000000
0000000000000000000000000000000000000000000000000000000000000000000000000000000000000000000000000000000000000000000000000000
0000000000000000000000000000000000000000000000000000000000000000000000000000000000000000000000000000000000000000000000000000
>O67140
MKSLLRQIPQISKVVEIFKKKYPEIYVVKAAREVAEKYRKEIIEGKRKDLNGFLEDVERKIKSLMKPNIKRVINATGVVINTNLGRAPLSKDVINFISEIANGYSNLEYNLEEGKRGSRIAHIEKYLNELTGAESSFVVNNNAGAVFLVLNTLAEGKEVIISRGELVEIGGSFRI
PDIMKKSGAILREVGTTNKTKVSDYEGAINQNTALLMKVHKSNFYMEGFVEEVKLEDLVKLGHKYGIPTYYDAGSGLLINLKEFGISVDEPNFRDCISLGIDLVSGSGDKLLGGPQAGIIVGKKNLIEKIKKNPIARALRIDKLTLSGLEMTLKLYFEKRYEDIPVIRMLTQDEK
ALRQKAKRLEKLLKDIPGLKISVIKDKAKPGGGSLPELELPTYCVAIRHDRLSSQELSRRLRLAEPPIVCRIREDQLLFDMRTVFHEDLKTIKKTLQELLSI
0100110111111100100000001000100010000001000000000000000000000000000000000000000000000000000000000000000000000000000000000000000000000000000000000000000000000000000000000000
0000000000000000000000000000000000000000000000000000000000000000000001000010000000000000000000000000000000000000000000000000000000000000000000000000000000000000000000000000000
000000000000000000000000000000000000000000000000000000010000000000000001111000000000000000000000000000
0000000000000000000000000000000000000000000000000000000000000000000000000000000000000000000000000000000000000000000000000000000000000000000000000000000000000000000000000000000
0000000000000000000000000000000000000000000000000000000000000000000000000000000000000000000000000000000000000000000000000000000000000000000000000000000000000000000000000000000
000000000000000000000000000000000000000000000000000000000000000000000000000000000000000000000000000000
0100110111111100100000001000100010000001000000000000000000000000000000000000000000000000000000000000000000000000000000000000000000000000000000000000000000000000000000000000
0000000000000000000000000000000000000000000000000000000000000000000001000010000000000000000000000000000000000000000000000000000000000000000000000000000000000000000000000000000
000000000000000000000000000000000000000000000000000000010000000000000001111000000000000000000000000000
0000000000000000000000000000000000000000000000000000000000000000000000000000000000000000000000000000000000000000000000000000000000000000000000000000000000000000000000000000000
0000000000000000000000000000000000000000000000000000000000000000000000000000000000000000000000000000000000000000000000000000000000000000000000000000000000000000000000000000000
000000000000000000000000000000000000000000000000000000000000000000000000000000000000000000000000000000
0000000000000000000000000000000000000000000000000000000000000000000000000000000000000000000000000000000000000000000000000000000000000000000000000000000000000000000000000000000
0000000000000000000000000000000000000000000000000000000000000000000000000000000000000000000000000000000000000000000000000000000000000000000000000000000000000000000000000000000
000000000000000000000000000000000000000000000000000000000000000000000000000000000000000000000000000000
0000000000000000000000000000000000000000000000000000000000000000000000000000000000000000000000000000000000000000000000000000000000000000000000000000000000000000000000000000000
0000000000000000000000000000000000000000000000000000000000000000000000000000000000000000000000000000000000000000000000000000000000000000000000000000000000000000000000000000000
000000000000000000000000000000000000000000000000000000000000000000000000000000000000000000000000000000
0000000000000000000000000000000000000000000000000000000000000000000000000000000000000000000000000000000000000000000000000000000000000000000000000000000000000000000000000000000
0000000000000000000000000000000000000000000000000000000000000000000000000000000000000000000000000000000000000000000000000000000000000000000000000000000000000000000000000000000
000000000000000000000000000000000000000000000000000000000000000000000000000000000000000000000000000000
>Q8TVD2
MELKFSAEVELTLSREVDPAEIEPTVEEFVKEANEDLLQRGVPTGKEGAKIESYRVLEDTIEMEITGTRYLRPHEAAMRVRKRLAERLGRKHRVGVRDLKIPRYEVVLRFDREVTRDDVGYVPVADDVVVEDGTVRLTFQDVDEEMLRRHVIDRVIRLVAWAVEERSELVERVTK
VEPGTVVDESGPREIRFDGDVTEEARRRGWVKEFPGRGQWIYTPPMAALFEVLRDFLLERVTRKLGFEPALFPKLIPLETMFRMRYLHGLPDGMYYVCPPKRDPELFDDFKRELYVWGELNERTLGSLKEKLRDPGYVLAPAQCEPFYELLRDEVVDPERLPIKLYDCSGWTYRW
EGGAAKGLERVNEFQRIEHVWIAEPEEAERIREELLEATKRVAEELELEWKVVVSDDPFYLEGRLLEDRDIELPDVPSYEFEVYLPFKGERSSEEAWISVGSFNVHGEHFVDGFNVKEKSGRTLFTGCAGLGVTRWVVGLLAQHGFEPEEWPEPILERIDEKFGGLPEVPKTLTW
PE
0000000000000100000000000000000000000000001101000000000000000000000001101001000100110010011001110000000000000000000000000000000000000000000000000000010000000000000000000000000
0000000000000000000000000000000000000000000000000000000000000000000000000000000000000000000000000000000000000000000000000000000000000000000000000000000000000000000000000000000
0000000000000000000000000000000000000000000000000000000000000000000000000000000000000000000000000000000000000000000000000000000000000000000000000000000000000000000000000000000
00
0000000000000000000000000000000000000000000000000000000000000000000000000000000000000000000000000000000000000000000000000000000000000000000000000000000000000000000000000000000
0000000000000000000000000000000000000000000000000000000000000000000000000000000000000000000000000000000000000000000000000000000000000000000000000000000000000000000000000000000
0000000000000000000000000000000000000000000000000000000000000000000000000000000000000000000000000000000000000000000000000000000000000000000000000000000000000000000000000000000
00
0000000000000100000000000000000000000000001101000000000000000000000001101001000100110010011001110000000000000000000000000000000000000000000000000000010000000000000000000000000
0000000000000000000000000000000000000000000000000000000000000000000000000000000000000000000000000000000000000000000000000000000000000000000000000000000000000000000000000000000
0000000000000000000000000000000000000000000000000000000000000000000000000000000000000000000000000000000000000000000000000000000000000000000000000000000000000000000000000000000
00
0000000000000000000000000000000000000000000000000000000000000000000000000000000000000000000000000000000000000000000000000000000000000000000000000000000000000000000000000000000
0000000000000000000000000000000000000000000000000000000000000000000000000000000000000000000000000000000000000000000000000000000000000000000000000000000000000000000000000000000

0000000000000000000000000000000000000000000000000000000000000000000000000000000000000000000000000000000000000000000000000000000000000000000000000000000000000000000000000000000
00
0000000000000000000000000000000000000000000000000000000000000000000000000000000000000000000000000000000000000000000000000000000000000000000000000000000000000000000000000000000
0000000000000000000000000000000000000000000000000000000000000000000000000000000000000000000000000000000000000000000000000000000000000000000000000000000000000000000000000000000
0000000000000000000000000000000000000000000000000000000000000000000000000000000000000000000000000000000000000000000000000000000000000000000000000000000000000000000000000000000
00
0000000000000000000000000000000000000000000000000000000000000000000000000000000000000000000000000000000000000000000000000000000000000000000000000000000000000000000000000000000
0000000000000000000000000000000000000000000000000000000000000000000000000000000000000000000000000000000000000000000000000000000000000000000000000000000000000000000000000000000
0000000000000000000000000000000000000000000000000000000000000000000000000000000000000000000000000000000000000000000000000000000000000000000000000000000000000000000000000000000
00
0000000000000000000000000000000000000000000000000000000000000000000000000000000000000000000000000000000000000000000000000000000000000000000000000000000000000000000000000000000
0000000000000000000000000000000000000000000000000000000000000000000000000000000000000000000000000000000000000000000000000000000000000000000000000000000000000000000000000000000
0000000000000000000000000000000000000000000000000000000000000000000000000000000000000000000000000000000000000000000000000000000000000000000000000000000000000000000000000000000
00
>O49937
MATSTSSSLSLSFFSSSLFSSKSRNFSSKPILKLPSSSHSQTSLSLSIKSELIPLPILNFSGEKVGETFLNLKTAPPEKARAVVHRGLITHLQNKRRGTASTLTRAEVRGGGRKPYPQKKTGRARRGSQGSPLRPGGGVIFGPKPRDWTIKMNKKERRLALSTAIASAVGNSFVV
EEFAENFEKPKTKDFIAAMQRWGLDPAEKSLFFLMDLVENVEKSGRNIRTLKLLTPRSLNLFDVLNAEKLVFTEGTIQYLNQRYGVDTLEDEDEEEEEEEEGEEVDDGVEDGTPEPAE
0000000000000000000000000000000000000000000000000000000000000000000000000000000001100100000111111011101110110011011001111111110010101110001110100101111111000000000000000000000
0000000001111001000010000000100000000000101101111100111101100010001000000000000000010000000000000000000000000000000000
0000000000000000000000000000000000000000000000000000000000000000000000000000000001100100000111111011101110110011011001111111110010101110001110100101111111000000000000000000000
0000000001111001000010000000100000000000101101111100111101100010001000000000000000010000000000000000000000000000000000
0000000000000000000000000000000000000000000000000000000000000000000000000000000000000000000000000000000000000000000000000000000000000000000000000000000000000000000000000000000
0000000000000000000000000000000000000000000000000000000000000000000000000000000000000000000000000000000000000000000000
0000000000000000000000000000000000000000000000000000000000000000000000000000000000000000000000000000000000000000000000000000000000000000000000000000000000000000000000000000000
0000000000000000000000000000000000000000000000000000000000000000000000000000000000000000000000000000000000000000000000
0000000000000000000000000000000000000000000000000000000000000000000000000000000000000000000000000000000000000000000000000000000000000000000000000000000000000000000000000000000
0000000000000000000000000000000000000000000000000000000000000000000000000000000000000000000000000000000000000000000000
0000000000000000000000000000000000000000000000000000000000000000000000000000000000000000000000000000000000000000000000000000000000000000000000000000000000000000000000000000000
0000000000000000000000000000000000000000000000000000000000000000000000000000000000000000000000000000000000000000000000
0000000000000000000000000000000000000000000000000000000000000000000000000000000000000000000000000000000000000000000000000000000000000000000000000000000000000000000000000000000
0000000000000000000000000000000000000000000000000000000000000000000000000000000000000000000000000000000000000000000000
>P03355
MGQTVTTPLSLTLGHWKDVERIAHNQSVDVKKRRWVTFCSAEWPTFNVGWPRDGTFNRDLITQVKIKVFSPGPHGHPDQVPYIVTWEALAFDPPPWVKPFVHPKPPPPLPPSAPSLPLEPPRSTPPRSSLYPALTPSLGAKPKPQVLSDSGGPLIDLLTEDPPPYRDPRPPPSDR
DGNGGEATPAGEAPDPSPMASRLRGRREPPVADSTTSQAFPLRAGGNGQLQYWPFSSSDLYNWKNNNPSFSEDPGKLTALIESVLITHQPTWDDCQQLLGTLLTGEEKQRVLLEARKAVRGDDGRPTQLPNEVDAAFPLERPDWDYTTQAGRNHLVHYRQLLLAGLQNAGRSPTN
LAKVKGITQGPNESPSAFLERLKEAYRRYTPYDPEDPGQETNVSMSFIWQSAPDIGRKLERLEDLKNKTLGDLVREAEKIFNKRETPEERREERIRRETEEKEERRRTEDEQKEKERDRRRHREMSKLLATVVSGQKQDRQGGERRRSQLDRDQCAYCKEKGHWAKDCPKKPRGPR
GPRPQTSLLTLDDGGGQGQEPPPEPRITLKVGGQPVTFLVDTGAQHSVLTQNPGPLSDKSAWVQGATGGKRYRWTTDRKVHLATGKVTHSFLHVPDCPYPLLGRDLLTKLKAQIHFEGSGAQVMGPMGQPLQVLTLNIEDEHRLHETSKEPDVSLGSTWLSDFPQAWAETGGMGL
AVRQAPLIIPLKATSTPVSIKQYPMSQEARLGIKPHIQRLLDQGILVPCQSPWNTPLLPVKKPGTNDYRPVQDLREVNKRVEDIHPTVPNPYNLLSGLPPSHQWYTVLDLKDAFFCLRLHPTSQPLFAFEWRDPEMGISGQLTWTRLPQGFKNSPTLFDEALHRDLADFRIQHPD
LILLQYVDDLLLAATSELDCQQGTRALLQTLGNLGYRASAKKAQICQKQVKYLGYLLKEGQRWLTEARKETVMGQPTPKTPRQLREFLGTAGFCRLWIPGFAEMAAPLYPLTKTGTLFNWGPDQQKAYQEIKQALLTAPALGLPDLTKPFELFVDEKQGYAKGVLTQKLGPWRRP
VAYLSKKLDPVAAGWPPCLRMVAAIAVLTKDAGKLTMGQPLVILAPHAVEALVKQPPDRWLSNARMTHYQALLLDTDRVQFGPVVALNPATLLPLPEEGLQHNCLDILAEAHGTRPDLTDQPLPDADHTWYTDGSSLLQEGQRKAGAAVTTETEVIWAKALPAGTSAQRAELIAL
TQALKMAEGKKLNVYTDSRYAFATAHIHGEIYRRRGLLTSEGKEIKNKDEILALLKALFLPKRLSIIHCPGHQKGHSAEARGNRMADQAARKAAITETPDTSTLLIENSSPYTSEHFHYTVTDIKDLTKLGAIYDKTKKYWVYQGKPVMPDQFTFELLDFLHQLTHLSFSKMKAL
LERSHSPYYMLNRDRTLKNITETCKACAQVNASKSAVKQGTRVRGHRPGTHWEIDFTEIKPGLYGYKYLLVFIDTFSGWIEAFPTKKETAKVVTKKLLEEIFPRFGMPQVLGTDNGPAFVSKVSQTVADLLGIDWKLHCAYRPQSSGQVERMNRTIKETLTKLTLATGSRDWVLL
LPLALYRARNTPGPHGLTPYEILYGAPPPLVNFPDPDMTRVTNSPSLQAHLQALYLVQHEVWRPLAAAYQEQLDRPVVPHPYRVGDTVWVRRHQTKNLEPRWKGPYTVLLTTPTALKVDGIAAWIHAAHVKAADPGGGPSSRLTWRVQRSQNPLKIRLTREAP
0000000000000000000000000000000000000000000000000000000000000000000000000000000000000000000000000000000000000000000000000000000000000000000000000000000000000000000000000000000
0000000000000000000000000000000000000000000000000000000000000000000000000000000000000000000000000000000000000000000000000000000000000000000000000000000000000000000000000000000
0000000000000000000000000000000000000000000000000000000000000000000000000000000000000000000000000000000000000000000000000000000000000000001000111011111111101110111101000011100011000000
0000000000000000000000000000000000000000000000000000000000000000000000000000000000000000000000000000000000000000000000000000000000000000000000000000000000000000000000000000000
0000000000000000000000000000000000000000000000000000000000000000000000000000000000000000000000000000000000000000000000000000000000000000000000000000000000000000000000000000000
0000000000000000000000000000000000000000000000000000000000000000000000000000000000000000000000000000000000000000000000000000000000000000000000000000000000000000000000000000000
0000000000000000000000000000000000000000000000000000000000000000000000000000000000000000000000000000000000000000000000000000000000000000000000000000000000000000000000000000000
0000000000000000000000000000000000000000000000000000000000000000000000000000000000000000000000000000000000000000000000000000000000000000000000000000000000000000000000000000000
0000000000000000000000000000000000000000000000000000000000000000000000000000000000000000000000000000000000000000000000000000000000000000000000000000000000000000000000000000000
0000000000000000000000000000000000000000000000000000000000000000000000000000000000000000000000000000000000000000000000000000000000000000000000000000000000000000000
0000000000000000000000000000000000000000000000000000000000000000000000000000000000000000000000000000000000000000000000000000000000000000000000000000000000000000000000000000000
0000000000000000000000000000000000000000000000000000000000000000000000000000000000000000000000000000000000000000000000000000000000000000000000000000000000000000000000000000000
0000000000000000000000000000000000000000000000000000000000000000000000000000000000000000000000000000000000000000000000000000000000000000000000000000000000000000000000000000000
0000000000000000000000000000000000000000000000000000000000000000000000000000000000000000000000000000000000000000000000000000000000000000000000000000000000000000000000000000000
0000000000000000000000000000000000000000000000000000000000000000000000000000000000000000000000000000000000000000000000000000000000000000000000000000000000000000000000000000000
0000000000000000000000000000000000000000000000000000000000000000000000000000000000000000000000000000000000000000000000000000000000000000000000000000000000000000000000000000000
0000000000000000000000000000000000000000000000000000000000000000000000000000000000000000000000000000000000000000000000000000000000000000000000000000000000000000000000000000000
0000000000000000000000000000000000000000000000000000000000000000000000000000000000000000000000000000000000000000000000000000000000000000000000000000000000000000000000000000000
0000000000000000000000000000000000000000000000000000000000000000000000000000000000000000000000000000000000000000000000000000000000000000000000000000000000000000000000000000000
0000000000000000000000000000000000000000000000000000000000000000000000000000000000000000000000000000000000000000000000000000000000000000000000000000000000000000000
0000000000000000000000000000000000000000000000000000000000000000000000000000000000000000000000000000000000000000000000000000000000000000000000000000000000000000000000000000000
0000000000000000000000000000000000000000000000000000000000000000000000000000000000000000000000000000000000000000000000000000000000000000000000000000000000000000000000000000000
0000000000000000000000000000000000000000000000000000000000000000000000000000000000000000000000000000000000000000000000000000000000000000001000111011111111101110111101000011100011000000
0000000000000000000000000000000000000000000000000000000000000000000000000000000000000000000000000000000000000000000000000000000000000000000000000000000000000000000000000000000
0000000000000000000000000000000000000000000000000000000000000000000000000000000000000000000000000000000000000000000000000000000000000000000000000000000000000000000000000000000
0000000000000000000000000000000000000000000000000000000000000000000000000000000000000000000000000000000000000000000000000000000000000000000000000000000000000000000000000000000
0000000000000000000000000000000000000000000000000000000000000000000000000000000000000000000000000000000000000000000000000000000000000000000000000000000000000000000000000000000

0000000000000000000000000000000000000000000000000000000000000000000000000000000000000000000000000000000000000000000000000000000000000000000000000000000000000000000000000000000
0000000000000000000000000000000000000000000000000000000000000000000000000000000000000000000000000000000000000000000000000000000000000000000000000000000000000000000000000000000
0000000000000000000000000000000000000000000000000000000000000000000000000000000000000000000000000000000000000000000000000000000000000000000000000000000000000000000
0000000000000000000000000000000000000000000000000000000000000000000000000000000000000000000000000000000000000000000000000000000000000000000000000000000000000000000000000000000
0000000000000000000000000000000000000000000000000000000000000000000000000000000000000000000000000000000000000000000000000000000000000000000000000000000000000000000000000000000
0000000000000000000000000000000000000000000000000000000000000000000000000000000000000000000000000000000000000000000000000000000000000000000000000000000000000000000000000000000
0000000000000000000000000000000000000000000000000000000000000000000000000000000000000000000000000000000000000000000000000000000000000000000000000000000000000000000000000000000
0000000000000000000000000000000000000000000000000000000000000000000000000000000000000000000000000000000000000000000000000000000000000000000000000000000000000000000000000000000
0000000000000000000000000000000000000000000000000000000000000000000000000000000000000000000000000000000000000000000000000000000000000000000000000000000000000000000000000000000
0000000000000000000000000000000000000000000000000000000000000000000000000000000000000000000000000000000000000000000000000000000000000000000000000000000000000000000000000000000
0000000000000000000000000000000000000000000000000000000000000000000000000000000000000000000000000000000000000000000000000000000000000000000000000000000000000000000000000000000
0000000000000000000000000000000000000000000000000000000000000000000000000000000000000000000000000000000000000000000000000000000000000000000000000000000000000000000000000000000
0000000000000000000000000000000000000000000000000000000000000000000000000000000000000000000000000000000000000000000000000000000000000000000000000000000000000000000
0000000000000000000000000000000000000000000000000000000000000000000000000000000000000000000000000000000000000000000000000000000000000000000000000000000000000000000000000000000
0000000000000000000000000000000000000000000000000000000000000000000000000000000000000000000000000000000000000000000000000000000000000000000000000000000000000000000000000000000
0000000000000000000000000000000000000000000000000000000000000000000000000000000000000000000000000000000000000000000000000000000000000000000000000000000000000000000000000000000
0000000000000000000000000000000000000000000000000000000000000000000000000000000000000000000000000000000000000000000000000000000000000000000000000000000000000000000000000000000
0000000000000000000000000000000000000000000000000000000000000000000000000000000000000000000000000000000000000000000000000000000000000000000000000000000000000000000000000000000
0000000000000000000000000000000000000000000000000000000000000000000000000000000000000000000000000000000000000000000000000000000000000000000000000000000000000000000000000000000
0000000000000000000000000000000000000000000000000000000000000000000000000000000000000000000000000000000000000000000000000000000000000000000000000000000000000000000000000000000
0000000000000000000000000000000000000000000000000000000000000000000000000000000000000000000000000000000000000000000000000000000000000000000000000000000000000000000000000000000
0000000000000000000000000000000000000000000000000000000000000000000000000000000000000000000000000000000000000000000000000000000000000000000000000000000000000000000000000000000
0000000000000000000000000000000000000000000000000000000000000000000000000000000000000000000000000000000000000000000000000000000000000000000000000000000000000000000
0000000000000000000000000000000000000000000000000000000000000000000000000000000000000000000000000000000000000000000000000000000000000000000000000000000000000000000000000000000
0000000000000000000000000000000000000000000000000000000000000000000000000000000000000000000000000000000000000000000000000000000000000000000000000000000000000000000000000000000
0000000000000000000000000000000000000000000000000000000000000000000000000000000000000000000000000000000000000000000000000000000000000000000000000000000000000000000000000000000
0000000000000000000000000000000000000000000000000000000000000000000000000000000000000000000000000000000000000000000000000000000000000000000000000000000000000000000000000000000
0000000000000000000000000000000000000000000000000000000000000000000000000000000000000000000000000000000000000000000000000000000000000000000000000000000000000000000000000000000
0000000000000000000000000000000000000000000000000000000000000000000000000000000000000000000000000000000000000000000000000000000000000000000000000000000000000000000000000000000
0000000000000000000000000000000000000000000000000000000000000000000000000000000000000000000000000000000000000000000000000000000000000000000000000000000000000000000000000000000
0000000000000000000000000000000000000000000000000000000000000000000000000000000000000000000000000000000000000000000000000000000000000000000000000000000000000000000000000000000
0000000000000000000000000000000000000000000000000000000000000000000000000000000000000000000000000000000000000000000000000000000000000000000000000000000000000000000000000000000
0000000000000000000000000000000000000000000000000000000000000000000000000000000000000000000000000000000000000000000000000000000000000000000000000000000000000000000
0000000000000000000000000000000000000000000000000000000000000000000000000000000000000000000000000000000000000000000000000000000000000000000000000000000000000000000000000000000
0000000000000000000000000000000000000000000000000000000000000000000000000000000000000000000000000000000000000000000000000000000000000000000000000000000000000000000000000000000
0000000000000000000000000000000000000000000000000000000000000000000000000000000000000000000000000000000000000000000000000000000000000000000000000000000000000000000000000000000
0000000000000000000000000000000000000000000000000000000000000000000000000000000000000000000000000000000000000000000000000000000000000000000000000000000000000000000000000000000
0000000000000000000000000000000000000000000000000000000000000000000000000000000000000000000000000000000000000000000000000000000000000000000000000000000000000000000000000000000
0000000000000000000000000000000000000000000000000000000000000000000000000000000000000000000000000000000000000000000000000000000000000000000000000000000000000000000000000000000
0000000000000000000000000000000000000000000000000000000000000000000000000000000000000000000000000000000000000000000000000000000000000000000000000000000000000000000000000000000
0000000000000000000000000000000000000000000000000000000000000000000000000000000000000000000000000000000000000000000000000000000000000000000000000000000000000000000000000000000
0000000000000000000000000000000000000000000000000000000000000000000000000000000000000000000000000000000000000000000000000000000000000000000000000000000000000000000000000000000
0000000000000000000000000000000000000000000000000000000000000000000000000000000000000000000000000000000000000000000000000000000000000000000000000000000000000000000
>P07012
MFEINPVNNRIQDLTERSDVLRGYLDYDAKKERLEEVNAELEQPDVWNEPERAQALGKERSSLEAVVDTLDQMKQGLEDVSGLLELAVEADDEETFNEAVAELDALEEKLAQLEFRRMFSGEYDSADCYLDIQAGSGGTEAQDWASMLERMYLRWAESRGFKTEIIEESEGEVAG
IKSVTIKISGDYAYGWLRTETGVHRLVRKSPFDSGGRRHTSFSSAFVYPEVDDDIDIEINPADLRIDVYRTSGAGGQHVNRTESAVRITHIPTGIVTQCQNDRSQHKNKDQAMKQMKAKLYELEMQKKNAEKQAMEDNKSDIGWGSQIRSYVLDDSRIKDLRTGVETRNTQAVLD
GSLDQFIEASLKAGL
0000000000000000000000000000000000000000000000000000000000000000000000000000000000000000000000000000000000000000000000000000000000000000000000000000000000000000000001101100
0000000000000000000000000000000000000000000000000000000000000000000000001001000000000000000000000000000000000000000000000000000000000000000000000000000000000000000000000000000
000000000000000
0000000000000000000000000000000000000000000000000000000000000000000000000000000000000000000000000000000000000000000000000000000000000000000000000000000000000000000001000000
0000000000000000000000000000000000000000000000000000000000000000000000001001000000000000000000000000000000000000000000000000000000000000000000000000000000000000000000000000000
000000000000000
0000000000000000000000000000000000000000000000000000000000000000000000000000000000000000000000000000000000000000000000000000000000000000000000000000000000000000000000101100
0000000000000000000000000000000000000000000000000000000000000000000000000000000000000000000000000000000000000000000000000000000000000000000000000000000000000000000000000000000
000000000000000
0000000000000000000000000000000000000000000000000000000000000000000000000000000000000000000000000000000000000000000000000000000000000000000000000000000000000000000000000000000
0000000000000000000000000000000000000000000000000000000000000000000000000000000000000000000000000000000000000000000000000000000000000000000000000000000000000000000000000000000
000000000000000
0000000000000000000000000000000000000000000000000000000000000000000000000000000000000000000000000000000000000000000000000000000000000000000000000000000000000000000000000000000
0000000000000000000000000000000000000000000000000000000000000000000000000000000000000000000000000000000000000000000000000000000000000000000000000000000000000000000000000000000
000000000000000
0000000000000000000000000000000000000000000000000000000000000000000000000000000000000000000000000000000000000000000000000000000000000000000000000000000000000000000000000000000
0000000000000000000000000000000000000000000000000000000000000000000000000000000000000000000000000000000000000000000000000000000000000000000000000000000000000000000000000000000
000000000000000
0000000000000000000000000000000000000000000000000000000000000000000000000000000000000000000000000000000000000000000000000000000000000000000000000000000000000000000000000000000
0000000000000000000000000000000000000000000000000000000000000000000000000000000000000000000000000000000000000000000000000000000000000000000000000000000000000000000000000000000
000000000000000
>P0A7J3

MALNLQDKQAIVAEVSEVAKGALSAVVADSRGVTVDKMTELRKAGREAGVYMRVVRNTLLRRAVEGTPFECLKDAFVGPTLIAYSMEHPGAAARLFKEFAKANAKFEVKAAAFEGELIPASQIDRLATLPTYEEAIARLMATMKEASAGKLVRTLAAVRDAKEAA
100100100010000000000000000011111100110010000000000001111110110010000000000000011000000000000000000000000000000000000000000000000000000000000000000000000000000000000
100100100010000000000000000011111100110010000000000001111110110010000000000000011000000000000000000000000000000000000000000000000000000000000000000000000000000000000
000000000000000000000000000000000000000000000000000000000000000000000000000000000000000000000000000000000000000000000000000000000000000000000000000000000000000000000
000000000000000000000000000000000000000000000000000000000000000000000000000000000000000000000000000000000000000000000000000000000000000000000000000000000000000000000
000000000000000000000000000000000000000000000000000000000000000000000000000000000000000000000000000000000000000000000000000000000000000000000000000000000000000000000
000000000000000000000000000000000000000000000000000000000000000000000000000000000000000000000000000000000000000000000000000000000000000000000000000000000000000000000
000000000000000000000000000000000000000000000000000000000000000000000000000000000000000000000000000000000000000000000000000000000000000000000000000000000000000000000
>P82920
MAKHLKFIARTVMVQEGNVEGAYRTLNRILTMDGLIEDIKRRRYYEKPCRRRQRESYETCRRIYNMEMARKINFLMRKNRADPWQGC
000000000000000000000000000000000010000000110110010111011001000000000000000000000000000
000000000000000000000000000000000010000000110110010111011001000000000000000000000000000
000000000000000000000000000000000000000000000000000000000000000000000000000000000000000
000000000000000000000000000000000000000000000000000000000000000000000000000000000000000
000000000000000000000000000000000000000000000000000000000000000000000000000000000000000
000000000000000000000000000000000000000000000000000000000000000000000000000000000000000
000000000000000000000000000000000000000000000000000000000000000000000000000000000000000
>Q6QAP7
MGRVRTKTVKKAARVIIEKYYTRLGNDFHTNKRVCEEIAIIPSKKLRNKIAGYVTHLMKRIQRGPVRGISIKLQEEERERRDNYVPEVSALDQEIIEVDPDTKEMLKLLDFGSLSNLQVTQPTVGMNFKTPRGAV
111111110110000000000000000100010000000000111101100110010011000000100000000000000000000000000000000000000000000000000000011110001000000
111111110110000000000000000100010000000000111101100110010011000000100000000000000000000000000000000000000000000000000000011110001000000
000000000000000000000000000000000000000000000000000000000000000000000000000000000000000000000000000000000000000000000000000000000000000
000000000000000000000000000000000000000000000000000000000000000000000000000000000000000000000000000000000000000000000000000000000000000
000000000000000000000000000000000000000000000000000000000000000000000000000000000000000000000000000000000000000000000000000000000000000
000000000000000000000000000000000000000000000000000000000000000000000000000000000000000000000000000000000000000000000000000000000000000
000000000000000000000000000000000000000000000000000000000000000000000000000000000000000000000000000000000000000000000000000000000000000
>Q9X1H4
MTESFTRRERLRLRRDFLLIFKEGKSLQNEYFVVLFRKNGLDYSRLGIVVKRKFGKATRRNKLKRWVREIFRRNKGVIPKGFDIVVIPRKKLSEEFERVDFWTVREKLLNLLKRIEG
000001100101111100100000000000000000000000000000000000011100110111011001100000000000000000000000000000000000000000000
000000000000000000000000000000000000000000000000000000000000000000000000000000000000000000000000000000000000000000000
000000000000000000000000000000000000000000000000000000000000000000000000000000000000000000000000000000000000000000000
000000000000000000000000000000000000000000000000000000000000000000000000000000000000000000000000000000000000000000000
000000000000000000000000000000000000000000000000000000000000000000000000000000000000000000000000000000000000000000000
000000000000000000000000000000000000000000000000000000000000000000000000000000000000000000000000000000000000000000000
000000000000000000000000000000000000000000000000000000000000000000000000000000000000000000000000000000000000000000000
>A7ZPW0
MSEQLVTPENVTTKDGKINLLDLNRQQMREFFKDLGEKPFRADQVMKWMYHYCCDNFDEMTDINKVLRGKLKEVAEIRAPEVVEEQRSSDGTIKWAIAVGDQRVETVYIPEDDRATLCVSSQVGCALECKFCSTAQQGFNRNLRVSEIIGQVWRAAKIVGAAKVTGQRPITNVVM
MGMGEPLLNLNNVVPAMEIMLDDFGFGLSKRRVTLSTSGVVPALDKLGDMIDVALAISLHAPNDEIRDEIVPINKKYNIETFLAAVRRYLEKSNANQGRVTIEYVMLDHVNDGTEHAHQLAELLKDTPCKINLIPWNPFPGAPYGRSSNSRIDRFSKVLMSYGFTTIVRKTRGDD
IDAACGQLAGDVIDRTKRTLRKRMQGEAIDIKAV
0000000000000000000000000000000000000101100000100010000010000111000100000000000000000000000000000000000000000100011010100000000000000001000000000000000000000000001000010001010
1000000000101000000000110010111101000000000000000000000110000000000000000000000000000000000001011011100000000001000000000000000001010000000000000000100000000001001011101101000
0011100000000000000000000000000000
0000000000000000000000000000000000000000000000000000000000000000000000000000000000000000000000000000000000000000000000000000000000000000000000000000000000000000000000000000000
0000000000000000000000000000000000000000000000000000000000000000000000000000000000000000000000000000000000000000000000000000000000000000000000000000000000000000000000000000000
0000000000000000000000000000000000
0000000000000000000000000000000000000101100000100010000010000111000100000000000000000000000000000000000000000100011010100000000000000001000000000000000000000000001000010001010
1000000000101000000000110010111101000000000000000000000110000000000000000000000000000000000001011011100000000001000000000000000001010000000000000000100000000001001011101101000
0011100000000000000000000000000000
0000000000000000000000000000000000000000000000000000000000000000000000000000000000000000000000000000000000000000000000000000000000000000000000000000000000000000000000000000000
0000000000000000000000000000000000000000000000000000000000000000000000000000000000000000000000000000000000000000000000000000000000000000000000000000000000000000000000000000000
0000000000000000000000000000000000
0000000000000000000000000000000000000000000000000000000000000000000000000000000000000000000000000000000000000000000000000000000000000000000000000000000000000000000000000000000
0000000000000000000000000000000000000000000000000000000000000000000000000000000000000000000000000000000000000000000000000000000000000000000000000000000000000000000000000000000
0000000000000000000000000000000000
0000000000000000000000000000000000000000000000000000000000000000000000000000000000000000000000000000000000000000000000000000000000000000000000000000000000000000000000000000000
0000000000000000000000000000000000000000000000000000000000000000000000000000000000000000000000000000000000000000000000000000000000000000000000000000000000000000000000000000000
0000000000000000000000000000000000
0000000000000000000000000000000000000000000000000000000000000000000000000000000000000000000000000000000000000000000000000000000000000000000000000000000000000000000000000000000
0000000000000000000000000000000000000000000000000000000000000000000000000000000000000000000000000000000000000000000000000000000000000000000000000000000000000000000000000000000
0000000000000000000000000000000000
>A8K7J6
MAALVTVLFTGVRRLHCSAAAWAGGQWRLQQGLAANPSGYGPLTELPDWSYADGRPAPPMKGQLRRKAERETFARRVVLLSQEMDAGLQAWQLRQQKLQEEQRKQENALKPKGASLKSPLPSQ
000000000000000000000000111010000011111000000000000000000011100010000000001000000000000000000000000000000000000000000000000
000000000000000000000000111010000011111000000000000000000011100010000000001000000000000000000000000000000000000000000000000
000000000000000000000000000000000000000000000000000000000000000000000000000000000000000000000000000000000000000000000000000
000000000000000000000000000000000000000000000000000000000000000000000000000000000000000000000000000000000000000000000000000
000000000000000000000000000000000000000000000000000000000000000000000000000000000000000000000000000000000000000000000000000
000000000000000000000000000000000000000000000000000000000000000000000000000000000000000000000000000000000000000000000000000
000000000000000000000000000000000000000000000000000000000000000000000000000000000000000000000000000000000000000000000000000

>E6PBS5
MALAKFHKKQSLTKIEEQVGSALVELQNTHPDLKTSLESIILTQVKEFQINKTNKKAKSAVLIYVHFQSYRVLLSAARKLIIELEKKLKQIVFFTAQRKIESKWVKEHKSQQRPRSRCLTYVYDALLDDLLLPSTLIGKRIRARLDGTSFYRIQLDQNDRDFLEEKLDAITHIYK
TVTTREVTFEFKEDKTFHTFKK
0000001000000000000000000000000000000000000000000000000000111000000000000000000000000000011111111111111111111111111000000000000000000000000000000000000000000000000000000
0011100000000000011111
0000001000000000000000000000000000000000000000000000000000111000000000000000000000000000011111111111111111111111111000000000000000000000000000000000000000000000000000000
0011100000000000011111
0000000000000000000000000000000000000000000000000000000000000000000000000000000000000000000000000000000000000000000000000000000000000000000000000000000000000000000000000000000
0000000000000000000000
0000000000000000000000000000000000000000000000000000000000000000000000000000000000000000000000000000000000000000000000000000000000000000000000000000000000000000000000000000000
0000000000000000000000
0000000000000000000000000000000000000000000000000000000000000000000000000000000000000000000000000000000000000000000000000000000000000000000000000000000000000000000000000000000
0000000000000000000000
0000000000000000000000000000000000000000000000000000000000000000000000000000000000000000000000000000000000000000000000000000000000000000000000000000000000000000000000000000000
0000000000000000000000
0000000000000000000000000000000000000000000000000000000000000000000000000000000000000000000000000000000000000000000000000000000000000000000000000000000000000000000000000000000
0000000000000000000000
>G1T168
MRTRAGRAAARASRPSGTAEATPDSARVLAGGGATPARKRRCAAHAPSFPASAPNPTAAAMLMPKKNRIAIYELLFKEGVMVAKKDVHMPKHPELADKNVPNLHVMKAMQSLKSRGYVKEQFAWRHFYWYLTNEGIQYLRDYLHLPPEIVPATLRRSRPETGRPRPKGLEGERPA
RLTRGEADRDTYRRSAVPPGADKKAEAGAGSATEFQFRGGFGRGRGQPPQ
0000000000000000000000000000000000000000000000000000000000001110100100000000000000001010000000000000000000100110011000000100001000000000000000000000000000000000000000000000000
00000000000000000000000000000000000000000000000000
0000000000000000000000000000000000000000000000000000000000001110100100000000000000001010000000000000000000100110011000000100001000000000000000000000000000000000000000000000000
00000000000000000000000000000000000000000000000000
0000000000000000000000000000000000000000000000000000000000000000000000000000000000000000000000000000000000000000000000000000000000000000000000000000000000000000000000000000000
00000000000000000000000000000000000000000000000000
0000000000000000000000000000000000000000000000000000000000000000000000000000000000000000000000000000000000000000000000000000000000000000000000000000000000000000000000000000000
00000000000000000000000000000000000000000000000000
0000000000000000000000000000000000000000000000000000000000000000000000000000000000000000000000000000000000000000000000000000000000000000000000000000000000000000000000000000000
00000000000000000000000000000000000000000000000000
0000000000000000000000000000000000000000000000000000000000000000000000000000000000000000000000000000000000000000000000000000000000000000000000000000000000000000000000000000000
00000000000000000000000000000000000000000000000000
0000000000000000000000000000000000000000000000000000000000000000000000000000000000000000000000000000000000000000000000000000000000000000000000000000000000000000000000000000000
00000000000000000000000000000000000000000000000000
>O28029
MTLDEEYLDITFLTENGFVRKRCPKCGKHFWTADPEREICGDPPCESYSFIGNPVFKKPFELDEMREYYLNFFERRGHGRIERYPVVARWRTDIYLTIASIADFQPFVTSGVAPPPANPLTISQPCIRLDDLDSVGRTGRHLTLFEMMAHHAFNYPGKEIYWKNETVAYCTELLN
ELGVKKEDIVYKEEPWAGGGNAGPCLEAIVGGLEVATLVFMNLEEHPEGDIEIKGARYRKMDNYIVDTGYGLERFVWASKGTPTVYDAIFPEVVDTIIDNSNVSFNREDERVRRIVAESSKLAGIMGELRGERLNQLRKSVADTVGVSVEELEGIVVPLEKVYSLADHTRCILFM
LGDGLVPSNAGAGYLARLMIRRSLRLAEELELGLDLYDLVEMHKKILGFEFDVPLSTVQEILELEKERYRTTVSKGTRLVERLVERKKKLEKDDLIELYDSHGIPVELAVGIAAEKGAEVEMPKDIYAELAKRHSKAEKVQEKKITLQNEYPATEKLYYDDPTLLEFEAEVIGVE
GDFVILNRSAFYPESGGQDNDVGYLIANGGKFEVVDVLEADGVVLHVVKGAKPEVGTKVKGVIDSDVRWRHMRHHSATHVLLYSLQKVLGNHVWQAGARKEFSKARLDVTHFRRPSEEEIKEIEMLANREILANKPIKWEWMDRIEAERKFGFRLYQGGVPPGRKIRVVQVGDDV
QACGGTHCRSTGEIGMLKILKVESIQDGVIRFEFAAGEAAIEAVEEMERLLREASSILRVEPAKLPKTVERFFEEWKDQRKEIERLKSVIADLWADILMERAEEFDSMKVVAEVVDADMQALQKLAERLAEKGAVGCLMAKGEGKVFVVTFSGQKYDARELLREIGRVAKGSGGG
RKDVAQGAVQQLLDREEMLDVIFRFLSEHEG
0000000000000000000000000000000000000000000000000000000000000000000000000000000000000000000000100000000000000000000000000000000100000000000000000000000000000000000000000000000
0000000000000111000000000000000000000000000010000000000000000000000000000000000000000000000000000000000000000000000000000000000000000000000000000000000000000000000000000000000
0000000011100100100011000000000000000000000000000000000000000000000000010000010001000100000000001011111000000000000000000000000000110110000000000000000000000000000000000000000
0000000000000000000000000000000000000000000000000000000000000000000000000000000000000000000000000000000000000000000000000000000000000000000000000000000000000000000000000000000
0000000000000000000000100010000000000000000000000000000001010000000000001000000001000000000000000000000000000000000000000000010001000000000000000001010000000000000000000000001111
1010011100000000000000000000000
0000000000000000000000000000000000000000000000000000000000000000000000000000000000000000000000000000000000000000000000000000000000000000000000000000000000000000000000000000000
0000000000000000000000000000000000000000000000000000000000000000000000000000000000000000000000000000000000000000000000000000000000000000000000000000000000000000000000000000000
0000000000000000000000000000000000000000000000000000000000000000000000000000000000000000000000000000000000000000000000000000000000000000000000000000000000000000000000000000000
0000000000000000000000000000000000000000000000000000000000000000000000000000000000000000000000000000000000000000000000000000000000000000000000000000000000000000000000000000000
0000000000000000000000000000000000000000000000000000000000000000000000000000000000000000000000000000000000000000000000000000000000000000000000000000000000000000000000000000000
0000000000000000000000000000000
0000000000000000000000000000000000000000000000000000000000000000000000000000000000000000000000100000000000000000000000000000000100000000000000000000000000000000000000000000000
0000000000000111000000000000000000000000000010000000000000000000000000000000000000000000000000000000000000000000000000000000000000000000000000000000000000000000000000000000000
0000000011100100100011000000000000000000000000000000000000000000000000010000010001000100000000001011111000000000000000000000000000110110000000000000000000000000000000000000000
0000000000000000000000000000000000000000000000000000000000000000000000000000000000000000000000000000000000000000000000000000000000000000000000000000000000000000000000000000000
0000000000000000000000100010000000000000000000000000000001010000000000001000000001000000000000000000000000000000000000000000010001000000000000000001010000000000000000000000001111
1010011100000000000000000000000
0000000000000000000000000000000000000000000000000000000000000000000000000000000000000000000000000000000000000000000000000000000000000000000000000000000000000000000000000000000
0000000000000000000000000000000000000000000000000000000000000000000000000000000000000000000000000000000000000000000000000000000000000000000000000000000000000000000000000000000
0000000000000000000000000000000000000000000000000000000000000000000000000000000000000000000000000000000000000000000000000000000000000000000000000000000000000000000000000000000
0000000000000000000000000000000000000000000000000000000000000000000000000000000000000000000000000000000000000000000000000000000000000000000000000000000000000000000000000000000
0000000000000000000000000000000000000000000000000000000000000000000000000000000000000000000000000000000000000000000000000000000000000000000000000000000000000000000000000000000
0000000000000000000000000000000
0000000000000000000000000000000000000000000000000000000000000000000000000000000000000000000000000000000000000000000000000000000000000000000000000000000000000000000000000000000
0000000000000000000000000000000000000000000000000000000000000000000000000000000000000000000000000000000000000000000000000000000000000000000000000000000000000000000000000000000
0000000000000000000000000000000000000000000000000000000000000000000000000000000000000000000000000000000000000000000000000000000000000000000000000000000000000000000000000000000
0000000000000000000000000000000000000000000000000000000000000000000000000000000000000000000000000000000000000000000000000000000000000000000000000000000000000000000000000000000

0000000000000000000000000000000000000000000000000000000000000000000000000000000000000000000000000000000000000000000000000000000000000000000000000000000000000000000000000000000
0000000000000000000000000000000
0000000000000000000000000000000000000000000000000000000000000000000000000000000000000000000000000000000000000000000000000000000000000000000000000000000000000000000000000000000
0000000000000000000000000000000000000000000000000000000000000000000000000000000000000000000000000000000000000000000000000000000000000000000000000000000000000000000000000000000
0000000000000000000000000000000000000000000000000000000000000000000000000000000000000000000000000000000000000000000000000000000000000000000000000000000000000000000000000000000
0000000000000000000000000000000000000000000000000000000000000000000000000000000000000000000000000000000000000000000000000000000000000000000000000000000000000000000000000000000
0000000000000000000000000000000000000000000000000000000000000000000000000000000000000000000000000000000000000000000000000000000000000000000000000000000000000000000000000000000
0000000000000000000000000000000
0000000000000000000000000000000000000000000000000000000000000000000000000000000000000000000000000000000000000000000000000000000000000000000000000000000000000000000000000000000
0000000000000000000000000000000000000000000000000000000000000000000000000000000000000000000000000000000000000000000000000000000000000000000000000000000000000000000000000000000
0000000000000000000000000000000000000000000000000000000000000000000000000000000000000000000000000000000000000000000000000000000000000000000000000000000000000000000000000000000
0000000000000000000000000000000000000000000000000000000000000000000000000000000000000000000000000000000000000000000000000000000000000000000000000000000000000000000000000000000
0000000000000000000000000000000000000000000000000000000000000000000000000000000000000000000000000000000000000000000000000000000000000000000000000000000000000000000000000000000
0000000000000000000000000000000
>O66640
MGKSDKIIPIAENKEAKAKYDILETYEAGIVLKGSEVKSLREKGTVSFKDSFVRIENGEAWLYNLYIAPYKHATIENHDPLRKRKLLLHKREIMRLYGKVQEKGYTIIPLKLYWKNNKVKVLIALAKGKKLYDRRRELKEKAMKRELEREFKGKIHL
0000000000000000000110000000000000000000000000000000000000000000000000000000000000000000000000000000000000000000000000000101110000000000000000000000000000000
0000000000000000000110000000000000000000000000000000000000000000000000000000000000000000000000000000000000000000000000000101110000000000000000000000000000000
0000000000000000000000000000000000000000000000000000000000000000000000000000000000000000000000000000000000000000000000000000000000000000000000000000000000000
0000000000000000000000000000000000000000000000000000000000000000000000000000000000000000000000000000000000000000000000000000000000000000000000000000000000000
0000000000000000000000000000000000000000000000000000000000000000000000000000000000000000000000000000000000000000000000000000000000000000000000000000000000000
0000000000000000000000000000000000000000000000000000000000000000000000000000000000000000000000000000000000000000000000000000000000000000000000000000000000000
0000000000000000000000000000000000000000000000000000000000000000000000000000000000000000000000000000000000000000000000000000000000000000000000000000000000000
>O75394
MFLSAVFFAKSKSKNILVRMVSEAGTGFCFNTKRNRLREKLTLLHYDPVVKQRVLFVEKKKIRSL
00000000000001100010000000001111100000001011111000001000000001100
00000000000001100010000000001111100000001011111000001000000001100
00000000000000000000000000000000000000000000000000000000000000000
00000000000000000000000000000000000000000000000000000000000000000
00000000000000000000000000000000000000000000000000000000000000000
00000000000000000000000000000000000000000000000000000000000000000
00000000000000000000000000000000000000000000000000000000000000000
>O94620
MDKRKESESATNGHLVKRIRIQDSSLITEGSVLQRTSDLNVPNLQMFGHTAEVLVARFDPSGSYFASGGMDRQILLWNVFGDVKNYGVLNGCKGAITDLQWSRDSRVVYCSSSDTHLMSWDAVSGQKIRKHKGHAGVVNALDVLKVGSELLTSVSDDCTMKVWDSRSKDCIKTIE
EKYPLTAVAIAQQGTQVFIGGIDGAIKIWDLRNNHCSHVLKGHKDIITSLAISKDGSSLLSNSMDNTVRIFDVKPFASAQRQLQIFEGAIHGQEHNLLGVAWSRNSRFVGAGSSDKNVYVWSATGDLRYVLPGHEGSVNHVDFHPHQDIILSCSSDRTIFLGELN
0000000000000000000000000000000000000000000000000000000000000000000000010000000000000000000001000000000000000000000000000000000000000101000000000000000000000000000000000001
0000000
000000000000000000000000000000000000000000000000000000000000000000000000000000000000000000000000000000000000000000000000000000000000000000000000000000000000000000000
0000000000000000000000000000000000000000000000000000000000000000000000000000000000000000000000000000000000000000000000000000000000000000000000000000000000000000000000000000000
000000000000000000000000000000000000000000000000000000000000000000000000000000000000000000000000000000000000000000000000000000000000000000000000000000000000000000000
0000000000000000000000000000000000000000000000000000000000000000000000000000000000000000000000000000000000000000000000000000000000000000000000000000000000000000000000000000000
000000000000000000000000000000000000000000000000000000000000000000000000000000000000000000000000000000000000000000000000000000000000000000000000000000000000000000000
0000000000000000000000000000000000000000000000000000000000000000000000010000000000000000000001000000000000000000000000000000000000000101000000000000000000000000000000000001
0000000
000000000000000000000000000000000000000000000000000000000000000000000000000000000000000000000000000000000000000000000000000000000000000000000000000000000000000000000
0000000000000000000000000000000000000000000000000000000000000000000000000000000000000000000000000000000000000000000000000000000000000000000000000000000000000000000000000000000
000000000000000000000000000000000000000000000000000000000000000000000000000000000000000000000000000000000000000000000000000000000000000000000000000000000000000000000
0000000000000000000000000000000000000000000000000000000000000000000000000000000000000000000000000000000000000000000000000000000000000000000000000000000000000000000000000000000
000000000000000000000000000000000000000000000000000000000000000000000000000000000000000000000000000000000000000000000000000000000000000000000000000000000000000000000
0000000000000000000000000000000000000000000000000000000000000000000000000000000000000000000000000000000000000000000000000000000000000000000000000000000000000000000000000000000
000000000000000000000000000000000000000000000000000000000000000000000000000000000000000000000000000000000000000000000000000000000000000000000000000000000000000000000
>P02359
MPRRRVIGQRKILPDPKFGSELLAKFVNILMVDGKKSTAESIVYSALETLAQRSGKSELEAFEVALENVRPTVEVKSRRVGGSTYQVPVEVRPVRRNALAMRWIVEAARKRGDKSMALRLANELSDAAENKGTAVKKREDVHRMAEANKAFAHYRWLSLRSFSHQAGASSKQPAL
GYLN
0111111111110000000000001001111111111110010000000000000000000000000000000000011111110000000001011101100100010011101111001000000000000000000000000010000000000000000000000000000000
0000
0111111111110000000000001001111111111110010000000000000000000000000000000000011111110000000001011101100100010011101111001000000000000000000000000010000000000000000000000000000000
0000
0000000000000000000000000000000000000000000000000000000000000000000000000000000000000000000000000000000000000000000000000000000000000000000000000000000000000000000000000000000
0000
0000000000000000000000000000000000000000000000000000000000000000000000000000000000000000000000000000000000000000000000000000000000000000000000000000000000000000000000000000000
0000
0000000000000000000000000000000000000000000000000000000000000000000000000000000000000000000000000000000000000000000000000000000000000000000000000000000000000000000000000000000
0000
0000000000000000000000000000000000000000000000000000000000000000000000000000000000000000000000000000000000000000000000000000000000000000000000000000000000000000000000000000000
0000
0000000000000000000000000000000000000000000000000000000000000000000000000000000000000000000000000000000000000000000000000000000000000000000000000000000000000000000000000000000
0000
>P04650
MAAQKSFRIKQKMAKAKKQNRPLPQWIRLRTNNTIRYNAKRRNWRRTKMNI
001111100001001110101111011111000011010000010011100

001111100001001110101111011111100001101000010011100
000000000000000000000000000000000000000000000000000
000000000000000000000000000000000000000000000000000
000000000000000000000000000000000000000000000000000
000000000000000000000000000000000000000000000000000
000000000000000000000000000000000000000000000000000
>P05317
MGGIREKKAEYFAKLREYLEEYKSLFVVGVDNVSSQQMHEVRKELRGRAVVLMGKNTMVRRAIRGFLSDLPDFEKLLPFVKGNVGFVFTNEPLTEIKNVIVSNRVAAPARAGAVAPEDIWVRAVNTGMEPGKTSFFQALGVPTKIARGTIEIVSDVKVVDAGNKVGQSEASLLNL
LNISPFTFGLTVVQVYDNGQVFPSSILDITDEELVSHFVSAVSTIASISLAIGYPTLPSVGHTLINNYKDLLAVAIAASYHYPEIEDLVDRIENPEKYAAAAPAATSAASGDAAPAEEAAAEEEEESDDDMGFGLFD
1000010000110000000000000000001111110110000000000011101111001000000000000000000001110000000000000000000000000100000000000000000000000000000000000000000000000000000000000000000
00000000000000000000000000000000000000000000000000000000000000000000000000000000000000000000000000000000000000000000000000000000000000000
1000010000110000000000000000001111110110000000000011101111001000000000000000000001110000000000000000000000000100000000000000000000000000000000000000000000000000000000000000000
00000000000000000000000000000000000000000000000000000000000000000000000000000000000000000000000000000000000000000000000000000000000000000
00000000000000000000000000000000000000000000000000000000000000000000000000000000000000000000000000000000000000000000000000000000000000000000000000000000000000000000000000000000000
00000000000000000000000000000000000000000000000000000000000000000000000000000000000000000000000000000000000000000000000000000000000000000
00000000000000000000000000000000000000000000000000000000000000000000000000000000000000000000000000000000000000000000000000000000000000000000000000000000000000000000000000000000000
00000000000000000000000000000000000000000000000000000000000000000000000000000000000000000000000000000000000000000000000000000000000000000
00000000000000000000000000000000000000000000000000000000000000000000000000000000000000000000000000000000000000000000000000000000000000000000000000000000000000000000000000000000000
00000000000000000000000000000000000000000000000000000000000000000000000000000000000000000000000000000000000000000000000000000000000000000
00000000000000000000000000000000000000000000000000000000000000000000000000000000000000000000000000000000000000000000000000000000000000000000000000000000000000000000000000000000000
00000000000000000000000000000000000000000000000000000000000000000000000000000000000000000000000000000000000000000000000000000000000000000
00000000000000000000000000000000000000000000000000000000000000000000000000000000000000000000000000000000000000000000000000000000000000000000000000000000000000000000000000000000000
00000000000000000000000000000000000000000000000000000000000000000000000000000000000000000000000000000000000000000000000000000000000000000
>P05740
MARYGATSTNPAKSASARGSYLRVSFKNTRETAQAINGWELTKAQKYLEQVLDHQRAIPFRRFNSSIGRTAQGKEFGVTKARWPAKSVKFVQGLLQNAAANAEAKGLDATKLYVSHIQVNQAPKQRRRTYRAHGRINKYESSPSHIELVVTEKEEAVAKAAEKKVVRLTSRQRGR
IAAQKRIAA
1011011010100111111111111111010001000000000000000000011000001111111110110010111111111111000000011001110100000000001011001001111111111111111110100000000000000000000000000000000
000000000
1011011010100111111111111111010001000000000000000000011000001111111110110010111111111111000000011001110100000000001011001001111111111111111110100000000000000000000000000000000
000000000
0000000000000000000000000000000000000000000000000000000000000000000000000000000000000000000000000000000000000000000000000000000000000000000000000000000000000000000000000000000
000000000
0000000000000000000000000000000000000000000000000000000000000000000000000000000000000000000000000000000000000000000000000000000000000000000000000000000000000000000000000000000
000000000
0000000000000000000000000000000000000000000000000000000000000000000000000000000000000000000000000000000000000000000000000000000000000000000000000000000000000000000000000000000
000000000
0000000000000000000000000000000000000000000000000000000000000000000000000000000000000000000000000000000000000000000000000000000000000000000000000000000000000000000000000000000
000000000
0000000000000000000000000000000000000000000000000000000000000000000000000000000000000000000000000000000000000000000000000000000000000000000000000000000000000000000000000000000
000000000
>P09594
MGFFKKKEKKAEFDVFRLYGLHHPEPGKCDEITTRGYSISMSVDKARRVIDQIRGRSYAETLMILELMPYRACYPIFKLIYSAAANASHNKQFNKANLIISKAEVNKGITLKKVKPRARGRSYMIKRPTCHITIVLRDITHFDSYDKFLESLTPKKLIALLGLMSTGRRRELLCG
RFENHKIKSFLYKIALFKRYEVM
0000000000000000000000000000000011111000010110100000000000000000000011100100010010000100100000000000001000111110011111111010111100010000000000000000001000000000000000000000000
00000000000000000000000
0000000000000000000000000000000011111000010110100000000000000000000011100100010010000100100000000000001000111110011111111010111100010000000000000000001000000000000000000000000
00000000000000000000000
0000000000000000000000000000000000000000000000000000000000000000000000000000000000000000000000000000000000000000000000000000000000000000000000000000000000000000000000000000000
00000000000000000000000
0000000000000000000000000000000000000000000000000000000000000000000000000000000000000000000000000000000000000000000000000000000000000000000000000000000000000000000000000000000
00000000000000000000000
0000000000000000000000000000000000000000000000000000000000000000000000000000000000000000000000000000000000000000000000000000000000000000000000000000000000000000000000000000000
00000000000000000000000
0000000000000000000000000000000000000000000000000000000000000000000000000000000000000000000000000000000000000000000000000000000000000000000000000000000000000000000000000000000
00000000000000000000000
0000000000000000000000000000000000000000000000000000000000000000000000000000000000000000000000000000000000000000000000000000000000000000000000000000000000000000000000000000000
00000000000000000000000
>P0A6X3
MAKGQSLQDPFLNALRRERVPVSIYLVNGIKLQGQIESFDQFVILLKNTVSQMVYKHAISTVVPSRPVSHHSNNAGGGTSSNYHHGSSAQNTSAQQDSEETE
001111011000100110100000000000000000011111000000000001110000000000000000000000000000000000000000000000
000000000000000000000000000000000000000000000000000000000000000000000000000000000000000000000000000000
000000000000000000000000000000000000000000000000000000000000000000000000000000000000000000000000000000
000000000000000000000000000000000000000000000000000000000000000000000000000000000000000000000000000000
000000000000000000000000000000000000000000000000000000000000000000000000000000000000000000000000000000
000000000000000000000000000000000000000000000000000000000000000000000000000000000000000000000000000000
000000000000000000000000000000000000000000000000000000000000000000000000000000000000000000000000000000
>P0A7K6
MSNIIKQLEQEQMKQDVPSFRPGDTVEVKVWVVEGSKKRLQAFEGVVIAIRNRGLHSAFTVRKISNGEGVERVFQTHSPVVDSISVKRRGAVRKAKLYYLRERTGKAARIKERLN
0111011000000000000110000000000000000000000000001111111011100100000000101010000000000000000101111111011100010100111

0111011000000000000011000000000000000000000000000111111011100100000001010100000000000000001011111110111000101000111
0000000000000000000000000000000000000000000000000000000000000000000000000000000000000000000000000000000000000000000
0000000000000000000000000000000000000000000000000000000000000000000000000000000000000000000000000000000000000000000
0000000000000000000000000000000000000000000000000000000000000000000000000000000000000000000000000000000000000000000
0000000000000000000000000000000000000000000000000000000000000000000000000000000000000000000000000000000000000000000
0000000000000000000000000000000000000000000000000000000000000000000000000000000000000000000000000000000000000000000
>P0A7M2
MSRVCQVTGKRPVTGNNRSHALNATKRRFLPNLHSHRFWVESEKRFVTLRVSAKGMRVIDKKGIDTVLAELRARGEKY
011000000111111111111111011111111011110000011110101001101110011000000000000000011
011000000111111111111111011111111011110000011110101001101110011000000000000000011
000000000000000000000000000000000000000000000000000000000000000000000000000000
000000000000000000000000000000000000000000000000000000000000000000000000000000
000000000000000000000000000000000000000000000000000000000000000000000000000000
000000000000000000000000000000000000000000000000000000000000000000000000000000
000000000000000000000000000000000000000000000000000000000000000000000000000000
>P0A7N4
MAVQQNKPTRSKRGMRRSHDALTAVTSLSVDKTSGEKHLRHHITADGYYRGRKVIAK
011111111111111111111000111111110000111111000000110110001
011111111111111111111000111111110000111111000000110110001
000000000000000000000000000000000000000000000000000000000
000000000000000000000000000000000000000000000000000000000
000000000000000000000000000000000000000000000000000000000
000000000000000000000000000000000000000000000000000000000
000000000000000000000000000000000000000000000000000000000
>P0A7T3
MVTIRLARHGAKKRPFYQVVVADSRNARNGRFIERVGFFNPIASEKEEGTRLDLDRIAHWVGQGATISDRVAALIKEVNKAA
1110110111111111100001111111111101001101111111110010000000101111101111111001100001
1110110111111111100001111111111101001101111111110010000000101111101111111001100001
0000000000000000000000000000000000000000000000000000000000000000000000000000000000
0000000000000000000000000000000000000000000000000000000000000000000000000000000000
0000000000000000000000000000000000000000000000000000000000000000000000000000000000
0000000000000000000000000000000000000000000000000000000000000000000000000000000000
0000000000000000000000000000000000000000000000000000000000000000000000000000000000
>P0A7T7
MARYFRRRKFCRFTAEGVQEIDYKDIATLKNYITESGKIVPSRITGTRAKYQRQLARAIKRARYLSLLPYTDRHQ
000000000000000000000000000000000011111111100001110111011001101100000111110
000000000000000000000000000000000011111111100001110111011001101100000111110
000000000000000000000000000000000000000000000000000000000000000000000000000
000000000000000000000000000000000000000000000000000000000000000000000000000
000000000000000000000000000000000000000000000000000000000000000000000000000
000000000000000000000000000000000000000000000000000000000000000000000000000
000000000000000000000000000000000000000000000000000000000000000000000000000
>P0A7W1
MAHIEKQAGELQEKLIAVNRVSKTVKGGRIFSFTALTVVGDGNGRVGFGYGKAREVPAAIQKAMEKARRNMINVALNNGTLQHPVKGVHTGSRVFMQPASEGTGIIAGGAMRAVLEVAGVHNVLAKAYGSTNPINVVRATIDGLENMNSPEMVAAKRGKSVEEILGK
00000000000000000011111111111111010000000000000001011100000001001100100000000000000000001111101010111111111110110000000000011111111101100101000000000000000000000000000
00000000000000000011111111111111010000000000000001011100000001001100100000000000000000001111101010111111111110110000000000011111111101100101000000000000000000000000000
00000000000000000000000000000000000000000000000000000000000000000000000000000000000000000000000000000000000000000000000000000000000000000000000000000000000000000000000
00000000000000000000000000000000000000000000000000000000000000000000000000000000000000000000000000000000000000000000000000000000000000000000000000000000000000000000000
00000000000000000000000000000000000000000000000000000000000000000000000000000000000000000000000000000000000000000000000000000000000000000000000000000000000000000000000
00000000000000000000000000000000000000000000000000000000000000000000000000000000000000000000000000000000000000000000000000000000000000000000000000000000000000000000000
00000000000000000000000000000000000000000000000000000000000000000000000000000000000000000000000000000000000000000000000000000000000000000000000000000000000000000000000
>P0AG63
MTDKIRTLQGRVVSDKMEKSIVVAIERFVKHPIYGKFIKRTTKLHVHDENNECGIGDVVEIRECRPLSKTKSWTLVRVVEKAVL
000111000000010111110100111100001101111111111010110000000000011111111111000000000000
000111000000010111110100111100001101111111111010110000000000011111111111000000000000
000000000000000000000000000000000000000000000000000000000000000000000000000000000000
000000000000000000000000000000000000000000000000000000000000000000000000000000000000
000000000000000000000000000000000000000000000000000000000000000000000000000000000000
000000000000000000000000000000000000000000000000000000000000000000000000000000000000
000000000000000000000000000000000000000000000000000000000000000000000000000000000000
>P0C2H8
MAGLKDVVTREYTINLHKRLHGVSFKKRAPRAVKEIKKFAKLHMGTDDVRLAPELNQAIWKRGVKGVEYRLRLRISRKRNEEEDAKNPLFSYVEPVLVASAKGLQTVVVEEDA
00000000000111100001100111101110110011001110000011111011100111111000100000000000000000000000000000000000000000000
00000000000111100001100111101110110011001110000011111011100111111000100000000000000000000000000000000000000000000
00000000000000000000000000000000000000000000000000000000000000000000000000000000000000000000000000000000000000000
00000000000000000000000000000000000000000000000000000000000000000000000000000000000000000000000000000000000000000
00000000000000000000000000000000000000000000000000000000000000000000000000000000000000000000000000000000000000000
00000000000000000000000000000000000000000000000000000000000000000000000000000000000000000000000000000000000000000
00000000000000000000000000000000000000000000000000000000000000000000000000000000000000000000000000000000000000000

>P10121
MAKEKKRGFFSWLGFGQKEQTPEKETEVQNEQPVVEEIVQAQEPVKASEQAVEEQPQAHTEAEAETFAADVVEVTEQVAESEKAQPEAEVVAQPEPVVEETPEPVAIEREELPLPEDVNAEAVSPEEWQAEAETVEIVEAAEEEEAAKEEITDEELETALAAEAAEEEAVMVVPPAE
EEQPVEEIAQEQEKPTKEGFFARLKRSLLKTKENLGSGFISLFRGKKIDDDLFEELEEQLLIADVGVETTRKIITNLTEGASRKQLRDAEALYGLLKEEMGEILAKVDEPLNVEGKAPFVILMVGVNGVGKTTTIGKLARQFEQQGKSVMLAAGDTFRAAAVEQLQVWGQRNNIP
VIAQHTGADSASVIFDAIQAAKARNIDVLIADTAGRLQNKSHLMEELKKIVRVMKKLDVEAPHEVMLTIDASTGQNAVSQAKLFHEAVGLTGITLTKLDGTAKGGVIFSVADQFGIPIRYIGVERIEDLRPFKADDFIEALFARED
000000000000000000000000000000000000000000000000000000000000000000000000000000000000000000000000000000000000000000000000000000000000000000000000000000000000000000000000000000
000000000000000000000000000000000000000000000000000000000000000000000000000000000000000000000000000000000000000000000000000000000000000000000000000000000001000000000000000000
01111011100100110011001100000000011001011110110010011001000000000000000000000000000000000000000000000000000000000000000000000000000000000000000000
000000000000000000000000000000000000000000000000000000000000000000000000000000000000000000000000000000000000000000000000000000000000000000000000000000000000000000000000000000
000000000000000000000000000000000000000000000000000000000000000000000000000000000000000000000000000000000000000000000000000000000000000000000000000000000001000000000000000000
01111001100100010011001100000000000001010010000010000000000000000000000000000000000000000000000000000000000000000000000000000000000000000000000000
000000000000000000000000000000000000000000000000000000000000000000000000000000000000000000000000000000000000000000000000000000000000000000000000000000000000000000000000000000
000000000000000000000000000000000000000000000000000000000000000000000000000000000000000000000000000000000000000000000000000000000000000000000000000000000000000000000000000000
00000000000000000000000000000000000000000000000000000000000000000000000000000000000000000000000000000000000000000000000000000000000000000000000000
000000000000000000000000000000000000000000000000000000000000000000000000000000000000000000000000000000000000000000000000000000000000000000000000000000000000000000000000000000
000000000000000000000000000000000000000000000000000000000000000000000000000000000000000000000000000000000000000000000000000000000000000000000000000000000000000000000000000000
00000000000000000000000000000000000000000000000000000000000000000000000000000000000000000000000000000000000000000000000000000000000000000000000000
000000000000000000000000000000000000000000000000000000000000000000000000000000000000000000000000000000000000000000000000000000000000000000000000000000000000000000000000000000
000000000000000000000000000000000000000000000000000000000000000000000000000000000000000000000000000000000000000000000000000000000000000000000000000000000000000000000000000000
00000000000000000000000000000000000000000000000000000000000000000000000000000000000000000000000000000000000000000000000000000000000000000000000000
000000000000000000000000000000000000000000000000000000000000000000000000000000000000000000000000000000000000000000000000000000000000000000000000000000000000000000000000000000
000000000000000000000000000000000000000000000000000000000000000000000000000000000000000000000000000000000000000000000000000000000000000000000000000000000000000000000000000000
00000000000000000000000000000000000000000000000000000000000000000000000000000000000000000000000000000000000000000000000000000000000000000000000000
000000000000000000000000000000000000000000000000000000000000000000000000000000000000000000000000000000000000000000000000000000000000000000000000000000000000000000000000000000
000000000000000000000000000000000000000000000000000000000000000000000000000000000000000000000000000000000000000000000000000000000000000000000000000000000001000000000000000000
01111010100100110011001100000000011000001110110010011001000000000000000000000000000000000000000000000000000000000000000000000000000000000000000000
>P25337
MPRIKTRRSKPAPDGFEKIKPTLTDFEIQLRDAQKDKSSKLAAKSNEQLWEIMQLHHQRSRYIYTLYYKRKAISKDLYDWLIKEKYADKLLIAKWRKTGYEKLCCLRCIQKNETNNGSTCICRVPRAQLEEEARKKGTQVSFHQCVHCGCRGCASTD
0000000000000000000000000000000000000001111000000000000000000000000000000000000000000001110110111111100000010000111011110111001100100000001010011100000000110
0000000000000000000000000000000000000000000000000000000000000000000000000000000000000000000000000000000000000000000000000000000000000000000000000000000000000
0000000000000000000000000000000000000000000000000000000000000000000000000000000000000000000000000000000000000000000000000000000000000000000000000000000000000
0000000000000000000000000000000000000001111000000000000000000000000000000000000000000001110110111111100000010000111011110111001100100000001010011100000000110
0000000000000000000000000000000000000000000000000000000000000000000000000000000000000000000000000000000000000000000000000000000000000000000000000000000000000
0000000000000000000000000000000000000000000000000000000000000000000000000000000000000000000000000000000000000000000000000000000000000000000000000000000000000
0000000000000000000000000000000000000000000000000000000000000000000000000000000000000000000000000000000000000000000000000000000000000000000000000000000000000
>P28804
MAVPKKRTSIYKKRIRKNIWKKKGYWAALKAFSLAKSLSTGNSKSFFVRKISNQTLE
111111111110111111010000011111011001100101111110000000000
111111111110111111010000011111011001100101111110000000000
000000000000000000000000000000000000000000000000000000000
000000000000000000000000000000000000000000000000000000000
000000000000000000000000000000000000000000000000000000000
000000000000000000000000000000000000000000000000000000000
000000000000000000000000000000000000000000000000000000000
>P31164
MAQPLVAAPSSSSITSPIPRKLCSSLLTPSSLSLSSNPRNSLQFLNSKLFLSPPSTSHRRLSIVAMAPKPGKAKKVIGVIKLALEAGKATPAPPVGPALGSKGVNIMAFCKDYNARTADKPGFVIPVEITVFDDKSFTFILKTPPASVLLLKASGAEKGSKDPQMEKVGKITIDQ
LRGIATEKLPDLNCTTIESAMRIIAGTAANMGIDIDPPILVKKKKEVIF
000000000000000000000000000000000000000000000000000000000000000000001000000010000001000000000000000000000000000000000000000000100000000000000000111000000000111001000000000000000
0000000100111111000100110011001000000000000000000
000000000000000000000000000000000000000000000000000000000000000000001000000010000001000000000000000000000000000000000000000000100000000000000000111000000000111001000000000000000
0000000100111111000100110011001000000000000000000
000000000000000000000000000000000000000000000000000000000000000000000000000000000000000000000000000000000000000000000000000000000000000000000000000000000000000000000000000000
0000000000000000000000000000000000000000000000000
000000000000000000000000000000000000000000000000000000000000000000000000000000000000000000000000000000000000000000000000000000000000000000000000000000000000000000000000000000
0000000000000000000000000000000000000000000000000
000000000000000000000000000000000000000000000000000000000000000000000000000000000000000000000000000000000000000000000000000000000000000000000000000000000000000000000000000000
0000000000000000000000000000000000000000000000000
000000000000000000000000000000000000000000000000000000000000000000000000000000000000000000000000000000000000000000000000000000000000000000000000000000000000000000000000000000
0000000000000000000000000000000000000000000000000
000000000000000000000000000000000000000000000000000000000000000000000000000000000000000000000000000000000000000000000000000000000000000000000000000000000000000000000000000000
0000000000000000000000000000000000000000000000000
>P32324
MVAFTVDQMRSLMDKVTNVRNMSVIAHVDHGKSTLTDSLVQRAGIISAAKAGEARFTDTRKDEQERGITIKSTAISLYSEMSDEDVKEIKQKTDGNSFLINLIDSPGHVDFSSEVTAALRVTDGALVVVDTIEGVCVQTETVLRQALGERIKPVVVINKVDRALLELQVSKEDLY
QTFARTVESVNVIVSTYADEVLGDVQVYPARGTVAFGSGLHGWAFTIRQFATRYAKKFGVDKAKMMDRLWGDSFFNPKTKKWTNKDTDAEGKPLERAFNMFILDPIFRLFTAIMNFKKDEIPVLLEKLEIVLKGDEKDLEGKALLKVVMRKFLPAADALLEMIVLHLPSPVTAQA
YRAEQLYEGPADDANCIAIKNCDPKADLMLYVSKMVPTSDKGRFYAFGRVFAGTVKSGQKVRIQGPNYVPGKKDDLFIKAIQRVVLMMGRFVEPIDDCPAGNIIGLVGIDQFLLKTGTLTTSETAHNMKVMKFSVSPVVQVAVEVKNANDLPKLVEGLKRLSKSDPCVLTYMSES
GEHIVAGTGELHLEICLQDLEHDHAGVPLKISPPVVAYRETVESESSQTALSKSPNKHNRIYLKAEPIDEEVSLAIENGIINPRDDFKARARIMADDYGWDVTDARKIWCFGPDGNGPNLVIDQTKAVQYLHEIKDSVVAAFQWATKEGPIFGEEMRSVRVNILDVTLHADAIHR
GGGQIIPTMRRATYAGFLLADPKIQEPVFLVEIQCPEQAVGGIYSVLNKKRGQVVSEEQRPGTPLFTVKAYLPVNESFGFTGELRQATGGQAFPQMVFDHWSTLGSDPLDPTSKAGEIVLAARKRHGMKEEVPGWQEYYDKL
000000000000000000000000000000000000000000000000000000000000000000110000000000000000000000000000000000000000000000000000000000000000000000000000000000000000000000000000000000
000000000000000000000000000000000000000000000000000000000000000000000000000000000000000000000000000000000000000000000000000000000000000000000000000000000000000000000000000000
000000000000000000000000000000000000001111100000000000000000000000000000000000000000000000000000000000000000000000000000000000000000000000000000000000000000000000000000000000

00000000000000000000000000000000000000000000000000000000000000000000000000000000000000000000000000000000000000000000000000000000000000000000000000000000000000000000000000000000
00000000000000000000000000000000000000000000000000000000000000000000000000000000000000000000000000000000000000000000000000000000000000000000000
00000000000000000000000000000000000000000000000000000000000000000001100000000000000000000000000000000000000000000000000000000000000000000000000000000000000000000000000000000000
00000000000000000000000000000000000000000000000000000000000000000000000000000000000000000000000000000000000000000000000000000000000000000000000000000000000000000000000000000000
00000000000000000000000000000000000000011111000000000000000000000000000000000000000000000000000000000000000000000000000000000000000000000000000000000000000000000000000000000000
00000000000000000000000000000000000000000000000000000000000000000000000000000000000000000000000000000000000000000000000000000000000000000000000000000000000000000000000000000000
0000000000000000000000000000000000000000000000000000000000000000000000000000000000000000000000000000000000000000000000000000000000000000000000
00000000000000000000000000000000000000000000000000000000000000000000000000000000000000000000000000000000000000000000000000000000000000000000000000000000000000000000000000000000
00000000000000000000000000000000000000000000000000000000000000000000000000000000000000000000000000000000000000000000000000000000000000000000000000000000000000000000000000000000
00000000000000000000000000000000000000000000000000000000000000000000000000000000000000000000000000000000000000000000000000000000000000000000000000000000000000000000000000000000
00000000000000000000000000000000000000000000000000000000000000000000000000000000000000000000000000000000000000000000000000000000000000000000000000000000000000000000000000000000
0000000000000000000000000000000000000000000000000000000000000000000000000000000000000000000000000000000000000000000000000000000000000000000000
00000000000000000000000000000000000000000000000000000000000000000000000000000000000000000000000000000000000000000000000000000000000000000000000000000000000000000000000000000000
00000000000000000000000000000000000000000000000000000000000000000000000000000000000000000000000000000000000000000000000000000000000000000000000000000000000000000000000000000000
00000000000000000000000000000000000000000000000000000000000000000000000000000000000000000000000000000000000000000000000000000000000000000000000000000000000000000000000000000000
00000000000000000000000000000000000000000000000000000000000000000000000000000000000000000000000000000000000000000000000000000000000000000000000000000000000000000000000000000000
0000000000000000000000000000000000000000000000000000000000000000000000000000000000000000000000000000000000000000000000000000000000000000000000
00000000000000000000000000000000000000000000000000000000000000000000000000000000000000000000000000000000000000000000000000000000000000000000000000000000000000000000000000000000
00000000000000000000000000000000000000000000000000000000000000000000000000000000000000000000000000000000000000000000000000000000000000000000000000000000000000000000000000000000
00000000000000000000000000000000000000000000000000000000000000000000000000000000000000000000000000000000000000000000000000000000000000000000000000000000000000000000000000000000
00000000000000000000000000000000000000000000000000000000000000000000000000000000000000000000000000000000000000000000000000000000000000000000000000000000000000000000000000000000
0000000000000000000000000000000000000000000000000000000000000000000000000000000000000000000000000000000000000000000000000000000000000000000000
00000000000000000000000000000000000000000000000000000000000000000000000000000000000000000000000000000000000000000000000000000000000000000000000000000000000000000000000000000000
00000000000000000000000000000000000000000000000000000000000000000000000000000000000000000000000000000000000000000000000000000000000000000000000000000000000000000000000000000000
00000000000000000000000000000000000000000000000000000000000000000000000000000000000000000000000000000000000000000000000000000000000000000000000000000000000000000000000000000000
00000000000000000000000000000000000000000000000000000000000000000000000000000000000000000000000000000000000000000000000000000000000000000000000000000000000000000000000000000000
0000000000000000000000000000000000000000000000000000000000000000000000000000000000000000000000000000000000000000000000000000000000000000000000
00000000000000000000000000000000000000000000000000000000000000000000000000000000000000000000000000000000000000000000000000000000000000000000000000000000000000000000000000000000
00000000000000000000000000000000000000000000000000000000000000000000000000000000000000000000000000000000000000000000000000000000000000000000000000000000000000000000000000000000
00000000000000000000000000000000000000000000000000000000000000000000000000000000000000000000000000000000000000000000000000000000000000000000000000000000000000000000000000000000
00000000000000000000000000000000000000000000000000000000000000000000000000000000000000000000000000000000000000000000000000000000000000000000000000000000000000000000000000000000
0000000000000000000000000000000000000000000000000000000000000000000000000000000000000000000000000000000000000000000000000000000000000000000000
>P37765
MSEKLQKVLARAGHGSRREIESIIEAGRVSVDGKIAKLGDRVEVTPGLKIRIDGHLISVRESAEQICRVLAYYKPEGELCTRNDPEGRPTVFDRLPKLRGARWIAVGRLDVNTCGLLLFTTDGELANRLMHPSREVEREYAVRVFGQVDDAKLRDLSRGVQLEDGPAAFKTIKFS
GGEGINQWYNVTLTEGRNREVRRLWEAVGVQVSRLIRVRYGDIPLPKGLPRGGWTELDLAQTNYLRELVELPPETSSKVAVEKDRRRMKANQIRRAVKRHSQVSGGRRSGGRNNNG
00010110010000011100100000000000000000111110000000000000000000000000000000000000111110110100000000000001010011111010000000001101100111101001010000000000000000000000000000000000000
00000000000000010111110000000000000101001000000000000000000000000000000000000000000000000000000000000000000000000000
00010110010000011100100000000000000000111110000000000000000000000000000000000000111110110100000000000001010011111010000000001101100111101001010000000000000000000000000000000000000
00000000000000010111110000000000000101001000000000000000000000000000000000000000000000000000000000000000000000000000
00000000000000000000000000000000000000000000000000000000000000000000000000000000000000000000000000000000000000000000000000000000000000000000000000000000000000000000000000000000
00000000000000000000000000000000000000000000000000000000000000000000000000000000000000000000000000000000000000000000
00000000000000000000000000000000000000000000000000000000000000000000000000000000000000000000000000000000000000000000000000000000000000000000000000000000000000000000000000000000
00000000000000000000000000000000000000000000000000000000000000000000000000000000000000000000000000000000000000000000
00000000000000000000000000000000000000000000000000000000000000000000000000000000000000000000000000000000000000000000000000000000000000000000000000000000000000000000000000000000
00000000000000000000000000000000000000000000000000000000000000000000000000000000000000000000000000000000000000000000
00000000000000000000000000000000000000000000000000000000000000000000000000000000000000000000000000000000000000000000000000000000000000000000000000000000000000000000000000000000
00000000000000000000000000000000000000000000000000000000000000000000000000000000000000000000000000000000000000000000
00000000000000000000000000000000000000000000000000000000000000000000000000000000000000000000000000000000000000000000000000000000000000000000000000000000000000000000000000000000
00000000000000000000000000000000000000000000000000000000000000000000000000000000000000000000000000000000000000000000
>P39805
MKIAKVINNNVISVVNEQGKELVVMGRGLAFQKKSGDDVDEARIEKVFTLDNKDVSEKFKTLLYDIPIECMEVSEEIISYAKLQLGKKLNDSIYVSLTDHINFAIQRNQKGLDIKNALLWETKRLYKDEFAIGKEALVMVKNKTGVSLPEDEAGFIALHIVNAELNEEMPNIINI
TKVMQEILSIVKYHFKIEFNEESLHYYRFVTHLKFFAQRLFNGTHMESQDDFLLDTVKEKYHRAYECTKKIQTYIEREYEHKLTSDELLYLTIHIERVVKQA
00001111111000000000000111100111000000000000100000000000000000000000000000000000000000000000000000000000000000000000000000000000000000000000000000000000000000000000000000000000
000000000000000000000000000000000000000000000000000000000000000000000000000000000000000000000000000000
00000000000000000000000000000000000000000000000000000000000000000000000000000000000000000000000000000000000000000000000000000000000000000000000000000000000000000000000000000000
000000000000000000000000000000000000000000000000000000000000000000000000000000000000000000000000000000
00000000000000000000000000000000000000000000000000000000000000000000000000000000000000000000000000000000000000000000000000000000000000000000000000000000000000000000000000000000
000000000000000000000000000000000000000000000000000000000000000000000000000000000000000000000000000000
00000000000000000000000000000000000000000000000000000000000000000000000000000000000000000000000000000000000000000000000000000000000000000000000000000000000000000000000000000000
000000000000000000000000000000000000000000000000000000000000000000000000000000000000000000000000000000
00001111111000000000000111100111000000000000100000000000000000000000000000000000000000000000000000000000000000000000000000000000000000000000000000000000000000000000000000000000
000000000000000000000000000000000000000000000000000000000000000000000000000000000000000000000000000000
00000000000000000000000000000000000000000000000000000000000000000000000000000000000000000000000000000000000000000000000000000000000000000000000000000000000000000000000000000000
000000000000000000000000000000000000000000000000000000000000000000000000000000000000000000000000000000
00000000000000000000000000000000000000000000000000000000000000000000000000000000000000000000000000000000000000000000000000000000000000000000000000000000000000000000000000000000
000000000000000000000000000000000000000000000000000000000000000000000000000000000000000000000000000000
>P40567
MVEPARKKQRIDRDTHHTVAEPVTEAKNTLYVSQLNEKINMQRLRVNLFLLFATFGEVLKVSMNFKKQRGQAFITMRTIDQASLAQISLNGERFFGKPLKVEFSKSETKTL
000000000000000000000000000000100100100110100000000000000000000000001000000000000000000000000000000000100000000

000000000000000000000000000000000000000000000000000000000000000000000000000000000000000000000000000000000000000
000000000000000000000000000000000000000000000000000000000000000000000000000000000000000000000000000000000000000
000000000000000000000000000000100100100110100000000000000000000000001000000000000000000000000000000000000100000000
000000000000000000000000000000000000000000000000000000000000000000000000000000000000000000000000000000000000000
000000000000000000000000000000000000000000000000000000000000000000000000000000000000000000000000000000000000000
000000000000000000000000000000000000000000000000000000000000000000000000000000000000000000000000000000000000000
>P41805
MARRPARCYRYQKNKPYPKSRYNRAVPDSKIRIYDLGKKKATVDEFPLCVHLVSNELEQLSSEALEAARICANKYMTTVSGRDAFHLRVRVHPFHVLRINKMLSCAGADRLQQGMRGAWGKPHGLAARVDIGQIIFSVRTKDSNKDVVVEGLRRARYKFPGQQKIILSKKWGFTN
LDRPEYLKKREAGEVKDDGAFVKFLSKKGSLENNIREFPEYFAAQA
0001101101001111101001000000000000011000110000000000000000000000001001100000000001000000000000000011110110011100011111111101111011000000000000000000000000000000000010011111010000000000000
0000000000000000000000000000000000000000000000
0001101101001111101001000000000000011000110000000000000000000000001001100000000001000000000000000011110110011100011111111101111011000000000000000000000000000000000010011111010000000000000
0000000000000000000000000000000000000000000000
0000000000000000000000000000000000000000000000000000000000000000000000000000000000000000000000000000000000000000000000000000000000000000000000000000000000000000000000000000000
0000000000000000000000000000000000000000000000
0000000000000000000000000000000000000000000000000000000000000000000000000000000000000000000000000000000000000000000000000000000000000000000000000000000000000000000000000000000
0000000000000000000000000000000000000000000000
0000000000000000000000000000000000000000000000000000000000000000000000000000000000000000000000000000000000000000000000000000000000000000000000000000000000000000000000000000000
0000000000000000000000000000000000000000000000
0000000000000000000000000000000000000000000000000000000000000000000000000000000000000000000000000000000000000000000000000000000000000000000000000000000000000000000000000000000
0000000000000000000000000000000000000000000000
0000000000000000000000000000000000000000000000000000000000000000000000000000000000000000000000000000000000000000000000000000000000000000000000000000000000000000000000000000000
0000000000000000000000000000000000000000000000
>P43321
MTMNGIPVKLLNEAQGHIVSLELTTGATYRGKLVESEDSMNVQLRDVIATEPQGAVTHMDQIFVRGSQIKFIVVPDLLKNAPLFKKNSSRPMPPIRGPKRR
00010000000000000000000000000000000000101000000000011110000000001110000000000000000000000000000000000
00000000000000000000000000000000000000000000000000000000000000000000000000000000000000000000000000000
00000000000000000000000000000000000000000000000000000000000000000000000000000000000000000000000000000
00010000000000000000000000000000000000101000000000011110000000001110000000000000000000000000000000000
00000000000000000000000000000000000000000000000000000000000000000000000000000000000000000000000000000
00000000000000000000000000000000000000000000000000000000000000000000000000000000000000000000000000000
00000000000000000000000000000000000000000000000000000000000000000000000000000000000000000000000000000
>P43912
MWIGVISLFPEMFKAITEFGVTGRAVKHNLLKVECWNPRDFTFDKHKTVDDRPYGGGPGMLMMVQPLRDAIHTAKAAAGEGAKVIYLSPQGRKLDQGGVTELAQNQKLILVCGRYEGIDERLIQTEIDEEWSIGDYVLTGGELPAMTLIDAVARFIPGVLGKQASAEEDSFADGL
LDCPHYTRPEVLEGLTVPPVLMSGHHEEIRKWRLKQSLQRTWLRRPELLEGLALTDEQRKLLKEAQAEHNS
0000000000000000010110110000000000000000000000000000000000000000000000000000000000000000000000000000000000000000000000000000000000000000000000000000001000001010010111000000000
00000001010000000000001111000000000000000000000000000000000000000000000
0000000000000000000000000000000000000000000000000000000000000000000000000000000000000000000000000000000000000000000000000000000000000000000000000000000000000000000000000000000
00000000000000000000000000000000000000000000000000000000000000000000000
0000000000000000010110110000000000000000000000000000000000000000000000000000000000000000000000000000000000000000000000000000000000000000000000000000001000001010010111000000000
00000001010000000000001111000000000000000000000000000000000000000000000
0000000000000000000000000000000000000000000000000000000000000000000000000000000000000000000000000000000000000000000000000000000000000000000000000000000000000000000000000000000
00000000000000000000000000000000000000000000000000000000000000000000000
0000000000000000000000000000000000000000000000000000000000000000000000000000000000000000000000000000000000000000000000000000000000000000000000000000000000000000000000000000000
00000000000000000000000000000000000000000000000000000000000000000000000
0000000000000000000000000000000000000000000000000000000000000000000000000000000000000000000000000000000000000000000000000000000000000000000000000000000000000000000000000000000
00000000000000000000000000000000000000000000000000000000000000000000000
0000000000000000000000000000000000000000000000000000000000000000000000000000000000000000000000000000000000000000000000000000000000000000000000000000000000000000000000000000000
00000000000000000000000000000000000000000000000000000000000000000000000
>P46350
MSYDKVSQAKSIIIGTKQTVKALKRGSVKEVVVAKDADPILTSSVVSLAEDQGISVSMVESMKKLGKACGIEVGAAAVAIIL
0000000000000111110000000000000000000000110010000000000000010000000001011110000000000
0000000000000000000000000000000000000000000000000000000000000000000000000000000000
0000000000000000000000000000000000000000000000000000000000000000000000000000000000
0000000000000000000000000000000000000000000000000000000000000000000000000000000000
0000000000000000000000000000000000000000000000000000000000000000000000000000000000
0000000000000000000000000000000000000000000000000000000000000000000000000000000000
0000000000000000000000000000000000000000000000000000000000000000000000000000000000
>P47813
MPKNKGKGGKNRRRGKNENESEKRELVFKEDGQEYAQVIKMLGNGRLEAMCFDGVKRLCHIRGKLRKKVWINTSDIILVGLRDYQDNKADVILKYNADEARSLKAYGELPEHAKINETDTFGPGDDDEIQFDDIGDDDEDIDDI
000000000000000000000000000000000000000011111100000000001000101110100000000000000000000000000000100000000000000000000000000000000000000000000000
000000000000000000000000000000000000000011111100000000001000100000000000000000000000000000000000100000000000000000000000000000000000000000000000
000000000000000000000000000000000000000000000000000000000000000000000000000000000000000000000000000000000000000000000000000000000000000000000000
000000000000000000000000000000000000000000000000000000000000000000000000000000000000000000000000000000000000000000000000000000000000000000000000
000000000000000000000000000000000000000000000000000001110100000000000000000000000000000000000000000000000000000000000000000000000000000000000000
000000000000000000000000000000000000000000000000000000000000000000000000000000000000000000000000000000000000000000000000000000000000000000000000
000000000000000000000000000000000000000000000000000000000000000000000000000000000000000000000000000000000000000000000000000000000000000000000000
>P53854

MGSGDLNLLKSWNPKLMKNRKKVWETEQDLITEQQKLNTRLKEIEKERELNELLNESSKDKPETLKNDLALKKSGLEWMYQDAKLSDEKEDYLLGKKKLDSSILNQPATPPVRAATTISASGAATSISSQKKKSKLLKDDPMSKFKVTKQQRRTPDSTKKRAMSQRGKPLSKPAP
DLDY
0011010111110001100000000000000000000000000000000000000000000000000000000000000000000000000000000000000000000000000000000000000000000000000000000000000000000000000000000000000
0000
0000000000000000000000000000000000000000000000000000000000000000000000000000000000000000000000000000000000000000000000000000000000000000000000000000000000000000000000000000000
0000
0000000000000000000000000000000000000000000000000000000000000000000000000000000000000000000000000000000000000000000000000000000000000000000000000000000000000000000000000000000
0000
0011000110000001100000000000000000000000000000000000000000000000000000000000000000000000000000000000000000000000000000000000000000000000000000000000000000000000000000000000000
0000
0010010111110000000000000000000000000000000000000000000000000000000000000000000000000000000000000000000000000000000000000000000000000000000000000000000000000000000000000000000
0000
0000000000000000000000000000000000000000000000000000000000000000000000000000000000000000000000000000000000000000000000000000000000000000000000000000000000000000000000000000000
0000
0000000000000000000000000000000000000000000000000000000000000000000000000000000000000000000000000000000000000000000000000000000000000000000000000000000000000000000000000000000
0000
>P61247
MAVGKNKRLTKGGKKGAKKKVVDPFSKKDWYDVKAPAMFNIRNIGKTLVTRTQGTKIASDGLKGRVFEVSLADLQNDEVAFRKFKLITEDVQGKNCLTNFHGMDLTRDKMCSMVKKWQTMIEAHVDVKTTDGYLLRLFCVGFTKKRNNQIRKTSYAQHQQVRQIRKKMMEIMTRE
VQTNDLKEVVNKLIPDSIGKDIEKACQSIYPLHDVFVRKVKMLKKPKFELGKLMELHGEGSSSGKATGDETGAKVERADGYEPPVQESV
0000000000000000000000000000000000000000000000000000000000000000000000000000000000000000000000000000000000000000000000000000000000000000000000010010000000000000000000100000000
00000000000100000001000000010000000000001000000000000000000000000000000000000000000000000
0000000000000000000000000000000000000000000000000000000000000000000000000000000000000000000000000000000000000000000000000000000000000000000000000000000000000000000000000000000
00000000000000000000000000000000000000000000000000000000000000000000000000000000000000000
0000000000000000000000000000000000000000000000000000000000000000000000000000000000000000000000000000000000000000000000000000000000000000000000000000000000000000000000000000000
00000000000000000000000000000000000000000000000000000000000000000000000000000000000000000
0000000000000000000000000000000000000000000000000000000000000000000000000000000000000000000000000000000000000000000000000000000000000000000000000000000000000000000000000000000
00000000000000000000000000000000000000000000000000000000000000000000000000000000000000000
0000000000000000000000000000000000000000000000000000000000000000000000000000000000000000000000000000000000000000000000000000000000000000000000000000000000000000000000000000000
00000000000000000000000000000000000000000000000000000000000000000000000000000000000000000
0000000000000000000000000000000000000000000000000000000000000000000000000000000000000000000000000000000000000000000000000000000000000000000000010010000000000000000000100000000
00000000000100000001000000010000000000001000000000000000000000000000000000000000000000000
0000000000000000000000000000000000000000000000000000000000000000000000000000000000000000000000000000000000000000000000000000000000000000000000000000000000000000000000000000000
00000000000000000000000000000000000000000000000000000000000000000000000000000000000000000
>P62318
MSIGVPIKVLHEAEGHIVTCETNTGEVYRGKLIEAEDNMNCQMSNITVTYRDGRVAQLEQVYIRGSKIRFLILPDMLKNAPMLKSMKNKNQGSGAGRGKAAILKAQVAARGRGRGMGRGNIFQKRR
000001000000000000000010000000000000010000000000000000000000000000110000000000000000000000000000000000000000000000000000000000
000000000000000000000000000000000000000000000000000000000000000000000000000000000000000000000000000000000000000000000000000000
000000000000000000000000000000000000000000000000000000000000000000000000000000000000000000000000000000000000000000000000000000
000001000000000000000010000000000000010000000000000000000000000000110000000000000000000000000000000000000000000000000000000000
000000000000000000000000000000000000000000000000000000000000000000000000000000000000000000000000000000000000000000000000000000
000000000000000000000000000000000000000000000000000000000000000000000000000000000000000000000000000000000000000000000000000000
000000000000000000000000000000000000000000000000000000000000000000000000000000000000000000000000000000000000000000000000000000
>P62851
MPPKDDKKKKDAGKSAKKDKDPVNKSGGKAKKKKWSKGKVRDKLNNLVLFDKATYDKLCKEVPNYKLITPAVVSERLKIRGSLARAALQELLSKGLIKLVSKHRAQVIYTRNTKGGDAPAAGEDA
00000000000000000000000000000000000000000000000000000000000010011000000000110100000000000000000000000000000000000000000000000
00000000000000000000000000000000000000000000000000000000000000000000000000000000000000000000000000000000000000000000000000000
00000000000000000000000000000000000000000000000000000000000000000000000000000000000000000000000000000000000000000000000000000
00000000000000000000000000000000000000000000000000000000000000000000000000000000000000000000000000000000000000000000000000000
00000000000000000000000000000000000000000000000000000000000000000000000000000000000000000000000000000000000000000000000000000
00000000000000000000000000000000000000000000000000000000000010011000000000110100000000000000000000000000000000000000000000000
00000000000000000000000000000000000000000000000000000000000000000000000000000000000000000000000000000000000000000000000000000
>P82191
MAAILPTFSIKSSSCTYSSSKRSLSSPKAVSLSSSVNGTPKVQSLRLSTSFVCSPNQIIKLKSVSPSRSTQLRRAVGGLEIKMMSVDAGIGVMGTKLGMMSFFEEDGTVVPVTVIGFKEGNIVTQVKTESTDGYNAVQVGYERLRDRKLTMPERGHLNKAGVIPMRHLQEFRLVS
VDDFTPSQKLLFEELFKEGDMVDISGTTIGKGFQGGIKRHNFKRGLMTHGSKSHRALGSIGAGTTPGHVYKGKKMPGRMGGTKTKIRKLKIMKIDTDLRVVMIKGAVPGKPGNLLRLAPAKIVGKNIPKN
0000000000000000000000000000000000000000000000000000000000000000000000000000000000000000000000000000000000000000000001100000000000000000000000111011011000000000000001000000000
0000000000000000000000000000110110000000000000000000001000000000000000000000001010000000000000000001000000000011000000000000000000
0000000000000000000000000000000000000000000000000000000000000000000000000000000000000000000000000000000000000000000001100000000000000000000000111011011000000000000001000000000
0000000000000000000000000000110110000000000000000000001000000000000000000000001010000000000000000001000000000011000000000000000000
0000000000000000000000000000000000000000000000000000000000000000000000000000000000000000000000000000000000000000000000000000000000000000000000000000000000000000000000000000000
0000000000000000000000000000000000000000000000000000000000000000000000000000000000000000000000000000000000000000000000000000000000
0000000000000000000000000000000000000000000000000000000000000000000000000000000000000000000000000000000000000000000000000000000000000000000000000000000000000000000000000000000
0000000000000000000000000000000000000000000000000000000000000000000000000000000000000000000000000000000000000000000000000000000000
0000000000000000000000000000000000000000000000000000000000000000000000000000000000000000000000000000000000000000000000000000000000000000000000000000000000000000000000000000000
0000000000000000000000000000000000000000000000000000000000000000000000000000000000000000000000000000000000000000000000000000000000
0000000000000000000000000000000000000000000000000000000000000000000000000000000000000000000000000000000000000000000000000000000000000000000000000000000000000000000000000000000
0000000000000000000000000000000000000000000000000000000000000000000000000000000000000000000000000000000000000000000000000000000000
0000000000000000000000000000000000000000000000000000000000000000000000000000000000000000000000000000000000000000000000000000000000000000000000000000000000000000000000000000000
0000000000000000000000000000000000000000000000000000000000000000000000000000000000000000000000000000000000000000000000000000000000
>P82192

MASTSLLQSTSSSFAGVRFHCRTSAAPRVGLSSFTVKAAAGTAVFVDKAEAETINRLKTNYIEKMVPLLKEEFSYSNILEVPKVVKIVVNCGIGDASQNAKGLDAAINELALITGQRPVKTKAKTSIAGFKVREGMTLGIAVTLRGNLMYSFLDRLINLALPRTRDFQGVNPNSF
DGHGNYSVGFREQSVFPEIKPEIVGKARGMDVCITTTAKTDKEAYKLLSLMGMPFREGSGPSTLVRKKKLKSHHFDAKKGRRY
0000000000000000000000000000000000000000000000000000000001000000000000000000111100000101011100100000000000000000000111111101000111000000010010101100000000000000000000000001011
10101011111000000000000000011110101000100000000000000000000000000000000000000000000
0000000000000000000000000000000000000000000000000000000001000000000000000000111100000101011100100000000000000000000111111101000111000000010010101100000000000000000000000001011
10101011111000000000000000011110101000100000000000000000000000000000000000000000000
0000000000000000000000000000000000000000000000000000000000000000000000000000000000000000000000000000000000000000000000000000000000000000000000000000000000000000000000000000000
00000000000000000000000000000000000000000000000000000000000000000000000000000000000
0000000000000000000000000000000000000000000000000000000000000000000000000000000000000000000000000000000000000000000000000000000000000000000000000000000000000000000000000000000
00000000000000000000000000000000000000000000000000000000000000000000000000000000000
0000000000000000000000000000000000000000000000000000000000000000000000000000000000000000000000000000000000000000000000000000000000000000000000000000000000000000000000000000000
00000000000000000000000000000000000000000000000000000000000000000000000000000000000
0000000000000000000000000000000000000000000000000000000000000000000000000000000000000000000000000000000000000000000000000000000000000000000000000000000000000000000000000000000
00000000000000000000000000000000000000000000000000000000000000000000000000000000000
0000000000000000000000000000000000000000000000000000000000000000000000000000000000000000000000000000000000000000000000000000000000000000000000000000000000000000000000000000000
00000000000000000000000000000000000000000000000000000000000000000000000000000000000
>P82195
MAAATSLSFFHSTLASSSSSSVQQLSLPPKFVNFRPQTLPLIQAKAHTRREDRTARHVRIRKKVEGTPERPRLCVFRSNKHLYVQVIDDTKMHTLAAASTMQKAISENIDYSAGPTVEVAQKIGEMIAKSCLEKGITKVAFDRGGYPYHGRVKALADAAREHGLVF
0000000000000000000000000000000000000000000000001100100110001000000000000001111110001010000111001000110000000111101110000000000000000000000000001101110000000010000000
0000000000000000000000000000000000000000000000001100100110001000000000000001111110001010000111001000110000000111101110000000000000000000000000001101110000000010000000
0000000000000000000000000000000000000000000000000000000000000000000000000000000000000000000000000000000000000000000000000000000000000000000000000000000000000000000000
0000000000000000000000000000000000000000000000000000000000000000000000000000000000000000000000000000000000000000000000000000000000000000000000000000000000000000000000
0000000000000000000000000000000000000000000000000000000000000000000000000000000000000000000000000000000000000000000000000000000000000000000000000000000000000000000000
0000000000000000000000000000000000000000000000000000000000000000000000000000000000000000000000000000000000000000000000000000000000000000000000000000000000000000000000
0000000000000000000000000000000000000000000000000000000000000000000000000000000000000000000000000000000000000000000000000000000000000000000000000000000000000000000000
>P82244
MATLSLLSTGVGAAITNRTPSASLTFITGSRTTNKRVSFNGGSARSGSLHCSFLAPSSSLSSNFSGLSLGLDLTSNTGVSTDRCRRFVVRAGKAALCLTKRSRSRKSLARTHGFRLRMSTTSGRALLKRRRAKGRKILCTKTNPSSGKRASP
00000000000000000000000000000000000000000000000000000000000000000000000000000000000000000000000000000001111111110110110011110111111011110001000000000000
00000000000000000000000000000000000000000000000000000000000000000000000000000000000000000000000000000001111111110110110011110111111011110001000000000000
00000000000000000000000000000000000000000000000000000000000000000000000000000000000000000000000000000000000000000000000000000000000000000000000000000000
00000000000000000000000000000000000000000000000000000000000000000000000000000000000000000000000000000000000000000000000000000000000000000000000000000000
00000000000000000000000000000000000000000000000000000000000000000000000000000000000000000000000000000000000000000000000000000000000000000000000000000000
00000000000000000000000000000000000000000000000000000000000000000000000000000000000000000000000000000000000000000000000000000000000000000000000000000000
00000000000000000000000000000000000000000000000000000000000000000000000000000000000000000000000000000000000000000000000000000000000000000000000000000000
>P82413
MASKVLPQALLVIPSNHSLQCPPLKKQLGFPIDSNRRFSLSSNCRSNLMVSRASSNLFSSNFSSIFSFPARNSFVVRSEAEDSSDAPAESVAVVAEEELPVESEAEAEERPPRQQRVKLGDIMGILNKKAVHAAEELRPVPGIRTGDIVQIRLEVPENKRRLSVYKGIVISRQNA
GIHTTIRIRRIIAGVGVEIVFPLYSPNIKEIKVVSHRKVRKARLYYLRDKLPRLSTFK
0000000000000000000000000000000000000000000000000000000000000000000000000000000000000000000000000000000000000000001111111000100100000000000000110000000000000000000000000000010
10100010000000000000000100000000000001011110110010000000001
0000000000000000000000000000000000000000000000000000000000000000000000000000000000000000000000000000000000000000001111111000100100000000000000110000000000000000000000000000010
10100010000000000000000100000000000001011110110010000000001
0000000000000000000000000000000000000000000000000000000000000000000000000000000000000000000000000000000000000000000000000000000000000000000000000000000000000000000000000000000
0000000000000000000000000000000000000000000000000000000000
0000000000000000000000000000000000000000000000000000000000000000000000000000000000000000000000000000000000000000000000000000000000000000000000000000000000000000000000000000000
0000000000000000000000000000000000000000000000000000000000
0000000000000000000000000000000000000000000000000000000000000000000000000000000000000000000000000000000000000000000000000000000000000000000000000000000000000000000000000000000
0000000000000000000000000000000000000000000000000000000000
0000000000000000000000000000000000000000000000000000000000000000000000000000000000000000000000000000000000000000000000000000000000000000000000000000000000000000000000000000000
0000000000000000000000000000000000000000000000000000000000
0000000000000000000000000000000000000000000000000000000000000000000000000000000000000000000000000000000000000000000000000000000000000000000000000000000000000000000000000000000
0000000000000000000000000000000000000000000000000000000000
>Q03338
MPPRNTYEKGNPKRQNSPYYKPSFLRREETTNDEEKFQGHGLKTELHSALKSSNLNLIRRTYQTGENPYLSDPHDRGSSSRFNRRYERGLKFYQKGEISKRIAQERTLQKQQEEEELKRKLKQEEDEKDKRKLIESGDLPNLELHEDKFLLDLSKFKIYYDNNHGYEWWDTAYLD
EKGELMEKYDMNGTSPAEEKLAEDIDEVDDDDDDEHPSIRYVAHPLPEKINEAKVSIKAYLTQHERKRLRRNRRKMAREAREIKIKLGLLPKPEPKVKLSNMMSVFENDQNITDPTAWEKVVKDQVDLRKRKHLEENERRHEDAIKRRKEAVNMNVEKPTVYHCKVFQFKNLQNP
KIRFKLKMNSKELSLKGLCLRIRDDGPGIIIVVGNEKSCKFYENLVMKRIKWNEDFELHTNTGDIKMDMHNNSISKTWEGYLQDCKFKGWFMKVCNDQDSLLRTLGQFDSEHFYSPVQT
0000000000000000000000000000000000000000000000000000000000000000000000000000000000000000000000000000000000000000000000000000000000000000000000000000000000000000000000000000000
0000000000000000000000000000000000000000000000000000000001000011001101100100010001000000000001010010011010000000000000000000000011001000100110010001000000000000000000000000001
10111011101101000000100100000000000001000100100001000000010100000000000000000000000000000011111000000000100010000000000000
0000000000000000000000000000000000000000000000000000000000000000000000000000000000000000000000000000000000000000000000000000000000000000000000000000000000000000000000000000000
0000000000000000000000000000000000000000000000000000000000000000000000000000000000000000000000000000000000000000000000000000000000000000000000000000000000000000000000000000000
00000000000000000000000000000000000000000000000000000000000000000000000000000000000000000000000000000000000000000000000
0000000000000000000000000000000000000000000000000000000000000000000000000000000000000000000000000000000000000000000000000000000000000000000000000000000000000000000000000000000
0000000000000000000000000000000000000000000000000000000000000000000000000000000000000000000000000000000000000000000000000000000000000000000000000000000000000000000000000000000
00000000000000000000000000000000000000000000000000000000000000000000000000000000000000000000000000000000000000000000000
0000000000000000000000000000000000000000000000000000000000000000000000000000000000000000000000000000000000000000000000000000000000000000000000000000000000000000000000000000000
0000000000000000000000000000000000000000000000000000000001000011001101100100010001000000000001010010011010000000000000000000000011001000100110010001000000000000000000000000001
10111011101101000000100100000000000001000100100001000000010100000000000000000000000000000011111000000000100010000000000000

0000000000000000000000000000000000000000000000000000000000000000000000000000000000000000000000000000000000000000000000000000000000000000000000000000000000000000000000000000000
0000000000000000000000000000000000000000000000000000000000000000000000000000000000000000000000000000000000000000000000000000000000000000000000000000000000000000000000000000000
00000000000000000000000000000000000000000000000000000000000000000000000000000000000000000000000000000000000000000000000
0000000000000000000000000000000000000000000000000000000000000000000000000000000000000000000000000000000000000000000000000000000000000000000000000000000000000000000000000000000
0000000000000000000000000000000000000000000000000000000000000000000000000000000000000000000000000000000000000000000000000000000000000000000000000000000000000000000000000000000
00000000000000000000000000000000000000000000000000000000000000000000000000000000000000000000000000000000000000000000000
0000000000000000000000000000000000000000000000000000000000000000000000000000000000000000000000000000000000000000000000000000000000000000000000000000000000000000000000000000000
0000000000000000000000000000000000000000000000000000000000000000000000000000000000000000000000000000000000000000000000000000000000000000000000000000000000000000000000000000000
00000000000000000000000000000000000000000000000000000000000000000000000000000000000000000000000000000000000000000000000
>Q03772
MTTSHRPQLEARSGAKAAAYTPTGIEHARLLPGHTTLKYRKFKEEENLRANCAQEDRSNDKSLEEAVMNEEKQDVVGSGNLQETRSEKDQKDSLQELLVTQKNKVEDKAELEGNEQLKGGNSSRRSWRKGTAFGRHKVTKETNIKEHATKKSASGYINDMTKSEYHQEFLHKHVR
0001111101110001000100001010110111100000000000000000000000000000000000000000000000000000000000000000000000000000000000000000000000000000000000000000000000000000000000000000
0000000000000000000000000000000000000000000000000000000000000000000000000000000000000000000000000000000000000000000000000000000000000000000000000000000000000000000000000000000
0000000000000000000000000000000000000000000000000000000000000000000000000000000000000000000000000000000000000000000000000000000000000000000000000000000000000000000000000000000
0001111101110001000100001010110111100000000000000000000000000000000000000000000000000000000000000000000000000000000000000000000000000000000000000000000000000000000000000000
0000000000000000000000000000000000000000000000000000000000000000000000000000000000000000000000000000000000000000000000000000000000000000000000000000000000000000000000000000000
0000000000000000000000000000000000000000000000000000000000000000000000000000000000000000000000000000000000000000000000000000000000000000000000000000000000000000000000000000000
0000000000000000000000000000000000000000000000000000000000000000000000000000000000000000000000000000000000000000000000000000000000000000000000000000000000000000000000000000000
>Q12046
MTSWRDKSAKVQVKESELPSSIPAQTGLTFNIWYNKWSQGFAGNTRFVSPFALQPQLHSGKTRGDNDGQLFFCLFFAKGMCCLGPKCEYLHHIPDEEDIGKLALRTEVLDCFGREKFADYREDMGGIGSFRKKNKTLYVGGIDGALNSKHLKPAQIESRIRFVFSRLGDIDRIRY
VESKNCGFVKFKYQANAEFAKEAMSNQTLLLPSDKEWDDRREGTGLLVKWANEDPDAAQKRLQEELKLESLNMMVHLINNNTNSAGTEVNNKNNERLDRTFPEASVDNVKKRLLPLDNGMESDDFIEKLKKVKKNISRENISSKPSVGKLGGPLLDYLSSDED
0000000000000000011100000000001001011111100000110100000000000000000000000110000111000000100000000000000000000000000010111000111100000000000000000000000000000000000000000000000
0000000000000000000010001100000000000000000001110000000000000000000000000000000000000000000000000000000000000000000000000000000000000000000000000000000000000000000
0000000000000000000000000000000000000000000000000000000000000000000000000000000000000000000000000000000000000000000000000000000000000000000000000000000000000000000000000000000
0000000000000000000000000000000000000000000000000000000000000000000000000000000000000000000000000000000000000000000000000000000000000000000000000000000000000000000
0000000000000000000000000000000000000000000000000000000000000000000000000000000000000000000000000000000000000000000000000000000000000000000000000000000000000000000000000000000
0000000000000000000000000000000000000000000000000000000000000000000000000000000000000000000000000000000000000000000000000000000000000000000000000000000000000000000
0000000000000000011100000000001001011111100000110100000000000000000000000110000111000000100000000000000000000000000010111000111100000000000000000000000000000000000000000000000
0000000000000000000010001100000000000000000001110000000000000000000000000000000000000000000000000000000000000000000000000000000000000000000000000000000000000000000
0000000000000000000000000000000000000000000000000000000000000000000000000000000000000000000000000000000000000000000000000000000000000000000000000000000000000000000000000000000
0000000000000000000000000000000000000000000000000000000000000000000000000000000000000000000000000000000000000000000000000000000000000000000000000000000000000000000
0000000000000000000000000000000000000000000000000000000000000000000000000000000000000000000000000000000000000000000000000000000000000000000000000000000000000000000000000000000
0000000000000000000000000000000000000000000000000000000000000000000000000000000000000000000000000000000000000000000000000000000000000000000000000000000000000000000
0000000000000000000000000000000000000000000000000000000000000000000000000000000000000000000000000000000000000000000000000000000000000000000000000000000000000000000000000000000
0000000000000000000000000000000000000000000000000000000000000000000000000000000000000000000000000000000000000000000000000000000000000000000000000000000000000000000
>Q12417
MDGNDHKVENLGDVDKFYSRIRWNNQFSYMATLPPHLQSEMEGQKSLLMRYDTYRKESSSFSGEGKKVTLQHVPTDFSEASQAVISKKDHDTHASAFVNKIFQPEVAEELIVNRYEKLLSQRPEWHAPWKLSRVINGHLGWVRCVAIDPVDNEWFITGSNDTTMKVWDLATGKLK
TTLAGHVMTVRDVAVSDRHPYLFSVSEDKTVKCWDLEKNQIIRDYYGHLSGVRTVSIHPTLDLIATAGRDSVIKLWDMRTRIPVITLVGHKGPINQVQCTPVDPQVVSSSTDATVRLWDVVAGKTMKVLTHHKRSVRATALHPKEFSVASACTDDIRSWGLAEGSLLTNFESEKT
GIINTLSINQDDVLFAGGDNGVLSFYDYKSGHKYQSLATREMVGSLEGERSVLCSTFDKTGLRLITGEADKSIKIWKQDETATKESEPGLAWNPNLSAKRF
0000000000000000000000000000000000000000000000000000000000000000000000000000000000000000000000000000000000000000000000000000000000000000000000000000000000000000000000000000000
0000000000000000000000000000000100000100100000000000000000000000000000000000000000000000000000000000000000000000000000000000000000000000000000000000000000000000000000000000000
00000000000000000000000000000000000000000000000000000000000000000000000000000000000000000000000000000
0000000000000000000000000000000000000000000000000000000000000000000000000000000000000000000000000000000000000000000000000000000000000000000000000000000000000000000000000000000
0000000000000000000000000000000000000000000000000000000000000000000000000000000000000000000000000000000000000000000000000000000000000000000000000000000000000000000000000000000
00000000000000000000000000000000000000000000000000000000000000000000000000000000000000000000000000000
0000000000000000000000000000000000000000000000000000000000000000000000000000000000000000000000000000000000000000000000000000000000000000000000000000000000000000000000000000000
0000000000000000000000000000000000000000000000000000000000000000000000000000000000000000000000000000000000000000000000000000000000000000000000000000000000000000000000000000000
00000000000000000000000000000000000000000000000000000000000000000000000000000000000000000000000000000
0000000000000000000000000000000000000000000000000000000000000000000000000000000000000000000000000000000000000000000000000000000000000000000000000000000000000000000000000000000
0000000000000000000000000000000100000100100000000000000000000000000000000000000000000000000000000000000000000000000000000000000000000000000000000000000000000000000000000000000
00000000000000000000000000000000000000000000000000000000000000000000000000000000000000000000000000000
0000000000000000000000000000000000000000000000000000000000000000000000000000000000000000000000000000000000000000000000000000000000000000000000000000000000000000000000000000000
0000000000000000000000000000000000000000000000000000000000000000000000000000000000000000000000000000000000000000000000000000000000000000000000000000000000000000000000000000000
00000000000000000000000000000000000000000000000000000000000000000000000000000000000000000000000000000
0000000000000000000000000000000000000000000000000000000000000000000000000000000000000000000000000000000000000000000000000000000000000000000000000000000000000000000000000000000
0000000000000000000000000000000000000000000000000000000000000000000000000000000000000000000000000000000000000000000000000000000000000000000000000000000000000000000000000000000
00000000000000000000000000000000000000000000000000000000000000000000000000000000000000000000000000000
0000000000000000000000000000000000000000000000000000000000000000000000000000000000000000000000000000000000000000000000000000000000000000000000000000000000000000000000000000000
0000000000000000000000000000000000000000000000000000000000000000000000000000000000000000000000000000000000000000000000000000000000000000000000000000000000000000000000000000000
00000000000000000000000000000000000000000000000000000000000000000000000000000000000000000000000000000
>Q16540
MARNVVYPLYRLGGPQLRVFRTNFFIQLVRPGVAQPEDTVQFRIPMEMTRVDLRNYLEGIYNVPVAAVRTRVQHGSNKRRDHRNVRIKKPDYKVAYVQLAHGQTFTFPDLFPEKDESPEGSAADDLYSMLEEERQQRQSSDPRRGGVPSWFGL
000100000000000000000000000011010000101000001000000000000000011111101111110011100010001001000000000000000000000000000000000000000000000000000000000000000
000100000000000000000000000011010000101000001000000000000000011111101111110011100010001001000000000000000000000000000000000000000000000000000000000000000
000000000000000000000000000000000000000000000000000000000000000000000000000000000000000000000000000000000000000000000000000000000000000000000000000000000
000000000000000000000000000000000000000000000000000000000000000000000000000000000000000000000000000000000000000000000000000000000000000000000000000000000
000000000000000000000000000000000000000000000000000000000000000000000000000000000000000000000000000000000000000000000000000000000000000000000000000000000
000000000000000000000000000000000000000000000000000000000000000000000000000000000000000000000000000000000000000000000000000000000000000000000000000000000
000000000000000000000000000000000000000000000000000000000000000000000000000000000000000000000000000000000000000000000000000000000000000000000000000000000

```
>Q4KEY0
MLILTRKVGESINIGDDITITILGVSGQQVRIGINAPKDVAVHREEIYQRIQAGLTAPDKRETP
1111110000000000000101001111010000111011111101100110001111000000
0000000000000000000000000000000000000000000000000000000000000000
0000000000000000000000000000000000000000000000000000000000000000
0000000000000000000000000000000000000000000000000000000000000000
0000000000000000000000000000000000000000000000000000000000000000
0000000000000000000000000000000000000000000000000000000000000000
0000000000000000000000000000000000000000000000000000000000000000
>Q57902
MMPEICPRCGLPKELCVCEEIAKEEQKIKIYVTKRRFGKLMTIIEGFDTSVIDLKELAKKLKDICACGGTVKDNTIELQGDHRKKVAEELVKMGFSRDSIEIR
0000000000000000000000000000000011011000000000000000000000000000000000000000000000000000000000000000000
0000000000000000000000000000000000000000000000000000000000000000000000000000000000000000000000000000000
0000000000000000000000000000000011011000000000000000000000000000000000000000000000000000000000000000000
0000000000000000000000000000000000000000000000000000000000000000000000000000000000000000000000000000000
0000000000000000000000000000000000000000000000000000000000000000000000000000000000000000000000000000000
0000000000000000000000000000000000000000000000000000000000000000000000000000000000000000000000000000000
0000000000000000000000000000000000000000000000000000000000000000000000000000000000000000000000000000000
>Q58876
MRVEYSKDLIRKGISTISQLKKAKIRVEKDDKKISYKDAKPGKIDVNEFKKAIYLLIEADDFLYKKAPKHELNEEEAKEFCKLIIKCQEHLNKILANFGFEFEEKEIDEGALYIVSNKKLFKKLKNKNPNLKVVCTEGMLDIEDMRAIGVPEKALEGLKKKVEIARKNVERFIEK
YKPEKIFVVVEDDKDELLYLRAKNLYNAEKLDADEILD
0000000000000000000000000000000000000000000000000000000000000000000000000000000000000000000000000000000000000000000011100000100010000000110000000000000000000000100100010100000
00000000000000000000000000000000000000
0000000000000000000000000000000000000000000000000000000000000000000000000000000000000000000000000000000000000000000000000000000000000000000000000000000000000000000000000000000
00000000000000000000000000000000000000
0000000000000000000000000000000000000000000000000000000000000000000000000000000000000000000000000000000000000000000011100000100010000000110000000000000000000000100100010100000
00000000000000000000000000000000000000
0000000000000000000000000000000000000000000000000000000000000000000000000000000000000000000000000000000000000000000000000000000000000000000000000000000000000000000000000000000
00000000000000000000000000000000000000
0000000000000000000000000000000000000000000000000000000000000000000000000000000000000000000000000000000000000000000000000000000000000000000000000000000000000000000000000000000
00000000000000000000000000000000000000
0000000000000000000000000000000000000000000000000000000000000000000000000000000000000000000000000000000000000000000000000000000000000000000000000000000000000000000000000000000
00000000000000000000000000000000000000
0000000000000000000000000000000000000000000000000000000000000000000000000000000000000000000000000000000000000000000000000000000000000000000000000000000000000000000000000000000
00000000000000000000000000000000000000
>Q5L3T3
MIDKVRAFIHRHQLLSEGAAVIVGVSGGPDSLALLHVFLSLRDEWKLQVIAAHVDHMFRGRESEEEMEFVKRFCVERRILCETAQIDVPAFQRSAGLGAQEAARICRYRFFAELMEKHQAGYVAVGHHGDDQVETILMRLVRGSTSKGYAGIPVKRPFHGGYLIRPFLAVSRAEI
EAYCRQMGLSPRCDPSNEKDDYTRNRFRHHIVPLLRQENPRLHERFQQYSEMMAEDEQFLEELAADALNKVMEKQHRDAALSIGPFLELPRPLQRRVLQLLLLRLYGGVPPTLTSVHIGHILMLCERGRPSGMIDLPKGLKVIRSYDRCLFTFDAESGEKGYWFELPVPALLPLP
NGYAIISEFGEHYPRKQAGNDWFVVDPASVSLPLRVRTRRRGDRMVLKGTGGTKKLKEIFIEAKIPRMERDRWPIVEDADGRILWVPGLKKSAFEAQNRGQARYILLQYQAMNS
0000000000000000000000000000000000000000000000000000000000000000000000000000000000000000000000000000110010011000000000000000010001000100110011100010011100100000000010000000000
0000000000000000000001110001000000000000000000000000000000000000000000000000000000000000000000000000100010000100111110100100000001011101111010000000000000000000000000000000000
000000000000000000000000000000000000001010010010111011101100110100100100000000000000000001000000000000000000000000
0000000000000000000000000000000000000000000000000000000000000000000000000000000000000000000000000000000000000000000000000000000000000000000000000000000000000000000000000000000
0000000000000000000000000000000000000000000000000000000000000000000000000000000000000000000000000000000000000000000000000000000000000000000000000000000000000000000000000000000
000000000000000000000000000000000000000000000000000000000000000000000000000000000000000000000000000000000000000000
0000000000000000000000000000000000000000000000000000000000000000000000000000000000000000000000000000110010011000000000000000010001000100110011100010011100100000000010000000000
0000000000000000000001110001000000000000000000000000000000000000000000000000000000000000000000000000100010000100111110100100000001011101111010000000000000000000000000000000000
000000000000000000000000000000000000001010010010111011101100110100100100000000000000000001000000000000000000000000
0000000000000000000000000000000000000000000000000000000000000000000000000000000000000000000000000000000000000000000000000000000000000000000000000000000000000000000000000000000
0000000000000000000000000000000000000000000000000000000000000000000000000000000000000000000000000000000000000000000000000000000000000000000000000000000000000000000000000000000
000000000000000000000000000000000000000000000000000000000000000000000000000000000000000000000000000000000000000000
0000000000000000000000000000000000000000000000000000000000000000000000000000000000000000000000000000000000000000000000000000000000000000000000000000000000000000000000000000000
0000000000000000000000000000000000000000000000000000000000000000000000000000000000000000000000000000000000000000000000000000000000000000000000000000000000000000000000000000000
000000000000000000000000000000000000000000000000000000000000000000000000000000000000000000000000000000000000000000
0000000000000000000000000000000000000000000000000000000000000000000000000000000000000000000000000000000000000000000000000000000000000000000000000000000000000000000000000000000
0000000000000000000000000000000000000000000000000000000000000000000000000000000000000000000000000000000000000000000000000000000000000000000000000000000000000000000000000000000
000000000000000000000000000000000000000000000000000000000000000000000000000000000000000000000000000000000000000000
0000000000000000000000000000000000000000000000000000000000000000000000000000000000000000000000000000000000000000000000000000000000000000000000000000000000000000000000000000000
0000000000000000000000000000000000000000000000000000000000000000000000000000000000000000000000000000000000000000000000000000000000000000000000000000000000000000000000000000000
000000000000000000000000000000000000000000000000000000000000000000000000000000000000000000000000000000000000000000
>Q7LL14
MNQNTLYVNNLNDKINKNDLRTALYMLFSTYGTVVDIVALKTPKMRGQAHVVFFDPSAAAIAMKALKNFIFFGKEMKIQYAHSKSKIIERIVAENDSRGPLKRLRDEADLE
000000101100110110000000000000000011111110111010100000000000000000000000000000000100001111111110000000000000000
000000000000000000000000000000000000000000000000000000000000000000000000000000000000000000000000000000000000000
000000000000000000000000000000000000000000000000000000000000000000000000000000000000000000000000000000000000000
000000101100110110000000000000000011111110111010100000000000000000000000000000000100001111111110000000000000000
000000000000000000000000000000000000000000000000000000000000000000000000000000000000000000000000000000000000000
000000000000000000000000000000000000000000000000000000000000000000000000000000000000000000000000000000000000000
000000000000000000000000000000000000000000000000000000000000000000000000000000000000000000000000000000000000000
```

>Q8N983
MTARGTPSRFLASVLHNGLGRYVQQLQRLSFSVSRDGASSRGAREFVEREVIDFARRNPGVVIYVNSRPCCVPRVVAEYLNGAVREESIHCKSVEEISTLVQKLADQSGLDVIRIRKPFHTDNPSIQGQWHPFTNKPTTFRGLRPREVQDPAPAQDTGLRLSAVAPQILLPGWPD
PPDLPTVDPISSSLTSAPAPMLSAVSCLPIVPALTTVCSA
0111000000000000000000000000000000010111000000000000000000000000000011110100000000000000110000000000000000000000010111000000000000000000000000000000000000000000000000000000000
0000000000000000000000000000000000000000
0111000000000000000000000000000000010111000000000000000000000000000011110100000000000000110000000000000000000000010111000000000000000000000000000000000000000000000000000000000
0000000000000000000000000000000000000000
0000000000000000000000000000000000000000000000000000000000000000000000000000000000000000000000000000000000000000000000000000000000000000000000000000000000000000000000000000000
0000000000000000000000000000000000000000
0000000000000000000000000000000000000000000000000000000000000000000000000000000000000000000000000000000000000000000000000000000000000000000000000000000000000000000000000000000
0000000000000000000000000000000000000000
0000000000000000000000000000000000000000000000000000000000000000000000000000000000000000000000000000000000000000000000000000000000000000000000000000000000000000000000000000000
0000000000000000000000000000000000000000
0000000000000000000000000000000000000000000000000000000000000000000000000000000000000000000000000000000000000000000000000000000000000000000000000000000000000000000000000000000
0000000000000000000000000000000000000000
0000000000000000000000000000000000000000000000000000000000000000000000000000000000000000000000000000000000000000000000000000000000000000000000000000000000000000000000000000000
0000000000000000000000000000000000000000
>Q97Z79
MIYSRSKLPSEGEILIATVKQVFDYGSYVSLDEYGGLQAFLPWSEVSSKWVKNIRDVLKENRKVIVKVIRVDRRKGTVDVSLKKVTDDERRKKNLQWKKIQRLDKILELVSQKLKLSEKDAWEQVAWKLEAKYGDPITAIEKAVKEGEKILIDAGVPEIWVKPLLEEASKHAEER
KVKMSGLITVRTNEPLGVEKIKEVISKALENIEQDYESLLNIKIYTIGAPRYRVDVVGTNPKEASEALNQIISNLIKIGKEENVDISVVKK
0000000000000000000000000000000000000000000000000000000000000000000000000000000000000000000000000000000000000000000000000000000000000000000000000000000000000000000000000000000
0000000000000000000000000000000000000000000000011010100000000000000000000000000000000000000
0000000000000000000000000000000000000000000000000000000000000000000000000000000000000000000000000000000000000000000000000000000000000000000000000000000000000000000000000000000
0000000000000000000000000000000000000000000000000000000000000000000000000000000000000000000
0000000000000000000000000000000000000000000000000000000000000000000000000000000000000000000000000000000000000000000000000000000000000000000000000000000000000000000000000000000
0000000000000000000000000000000000000000000000011010100000000000000000000000000000000000000
0000000000000000000000000000000000000000000000000000000000000000000000000000000000000000000000000000000000000000000000000000000000000000000000000000000000000000000000000000000
0000000000000000000000000000000000000000000000000000000000000000000000000000000000000000000
0000000000000000000000000000000000000000000000000000000000000000000000000000000000000000000000000000000000000000000000000000000000000000000000000000000000000000000000000000000
0000000000000000000000000000000000000000000000000000000000000000000000000000000000000000000
0000000000000000000000000000000000000000000000000000000000000000000000000000000000000000000000000000000000000000000000000000000000000000000000000000000000000000000000000000000
0000000000000000000000000000000000000000000000000000000000000000000000000000000000000000000
0000000000000000000000000000000000000000000000000000000000000000000000000000000000000000000000000000000000000000000000000000000000000000000000000000000000000000000000000000000
0000000000000000000000000000000000000000000000000000000000000000000000000000000000000000000
>Q980W2
MSLRDLKEENRIVIWPSYFFSPTRSKGRRLARIPYKIKTEELVSTLRELGLDPIVIENKKYPRDRKINFLIAVKKVKSKNYTLKIIHNALMGTRQTNPNKSN
010101100011101001000111111011011000000000000000000000000011111010000000001001110001000000000000000000
000000000000000000000000000000000000000000000000000000000000000000000000000000000000000000000000000000
000000000000000000000000000000000000000000000000000000000000000000000000000000000000000000000000000000
000000000000000000000000000000000000000000000000000000000000000000000000000000000000000000000000000000
000000000000000000000000000000000000000000000000000000000000000000000000000000000000000000000000000000
000000000000000000000000000000000000000000000000000000000000000000000000000000000000000000000000000000
010101100011101001000111111011011000000000000000000000000011111010000000001001110001000000000000000000
>Q9BYC9
MVFLTAQLWLRNRVTDRYFRIQEVLKHARHFRGRKNRCYRLAVRTVIRAFVKCTKARYLKKKNMRTLWINRITAASQEHGLKYPALIGNLVKCQVELNRKVLADLAIYEPKTFKSLAALASRRRHEGFAAALGDGKEPEGIFSRVVQYH
00000000011111101001000000001111011011111011001110011001111111011001000000000000011100010000000001110000000000000000000000000001000000000000000000000
00000000011111101001000000001111011011111011001110011001111111011001000000000000011100010000000001110000000000000000000000000001000000000000000000000
00000000000000000000000000000000000000000000000000000000000000000000000000000000000000000000000000000000000000000000000000000000000000000000000000000
00000000000000000000000000000000000000000000000000000000000000000000000000000000000000000000000000000000000000000000000000000000000000000000000000000
00000000000000000000000000000000000000000000000000000000000000000000000000000000000000000000000000000000000000000000000000000000000000000000000000000
00000000000000000000000000000000000000000000000000000000000000000000000000000000000000000000000000000000000000000000000000000000000000000000000000000
00000000000000000000000000000000000000000000000000000000000000000000000000000000000000000000000000000000000000000000000000000000000000000000000000000
>Q9BYD1
MSSFSRAPQQWATFARIWYLLDGKMQPPGKLAAMASIRLQGLHKPVYHALSDCGDHVVIMNTRHIAFSGNKWEQKVYSSHTGYPGGFRQVTAAQLHLRDPVAIVKLAIYGMLPKNLHRRTMMERLHLFPDEYIPEDILKNLVEELPQPRKIPKRLDEYTQEEIDAFPRLWTPPED
YRL
0000000000000000000000000011110000000000000000011000000000000000000100100010011100111110000000000000000000101110011111000000000000000000000000000000000000000000000000000000000
110
0000000000000000000000000011110000000000000000011000000000000000000100100010011100111110000000000000000000101110011111000000000000000000000000000000000000000000000000000000000
110
0000000000000000000000000000000000000000000000000000000000000000000000000000000000000000000000000000000000000000000000000000000000000000000000000000000000000000000000000000000
000
0000000000000000000000000000000000000000000000000000000000000000000000000000000000000000000000000000000000000000000000000000000000000000000000000000000000000000000000000000000
000
0000000000000000000000000000000000000000000000000000000000000000000000000000000000000000000000000000000000000000000000000000000000000000000000000000000000000000000000000000000
000
0000000000000000000000000000000000000000000000000000000000000000000000000000000000000000000000000000000000000000000000000000000000000000000000000000000000000000000000000000000
000
0000000000000000000000000000000000000000000000000000000000000000000000000000000000000000000000000000000000000000000000000000000000000000000000000000000000000000000000000000000
000

>Q9HD33
MAAAGLALLCRRVSSALKSSRSLITPQVPACTGFFLSLLPKSTPNVTSFHQYRLLHTTLSRKGLEEFFDDPKNWGQEKVKSGAAWTCQQLRNKSNEDLHKLWYVLLKERNMLLTLEQEAKRQRLPMPSPERLDKVVDSMDALDKVVQEREDALRLLQTGQERARPGAWRRDIFGR
IIWHKFKQWVIPWHLNKRYNRKRFFALPYVDHFLRLEREKRARIKARKENLERKKAKILLKKFPHLAEAQKSSLV
00000000000000000000000000000000000000000000000000000000000000000000000000000000000000000000000000000000000000000000100110100000000000000000000000000000000000000001010001001000
101010101110001011001110001000000000010010000010011011101000000000000000000
00000000000000000000000000000000000000000000000000000000000000000000000000000000000000000000000000000000000000000000100110100000000000000000000000000000000000000001010001001000
101010101110001011001110001000000000010010000010011011101000000000000000000
0000000000000000000000000000000000000000000000000000000000000000000000000000000000000000000000000000000000000000000000000000000000000000000000000000000000000000000000000000000
000000000000000000000000000000000000000000000000000000000000000000000000000
0000000000000000000000000000000000000000000000000000000000000000000000000000000000000000000000000000000000000000000000000000000000000000000000000000000000000000000000000000000
000000000000000000000000000000000000000000000000000000000000000000000000000
0000000000000000000000000000000000000000000000000000000000000000000000000000000000000000000000000000000000000000000000000000000000000000000000000000000000000000000000000000000
000000000000000000000000000000000000000000000000000000000000000000000000000
0000000000000000000000000000000000000000000000000000000000000000000000000000000000000000000000000000000000000000000000000000000000000000000000000000000000000000000000000000000
000000000000000000000000000000000000000000000000000000000000000000000000000
0000000000000000000000000000000000000000000000000000000000000000000000000000000000000000000000000000000000000000000000000000000000000000000000000000000000000000000000000000000
000000000000000000000000000000000000000000000000000000000000000000000000000
>Q9M3I4
MKKNSFISVISDEKKEENKGSVEFQVFCFTNKIRRLTLHLELHKKDYSSQRGLRKTLGKRQRLLAYLLKINGVRYKELISKLNIRELKTR
000001000000000011110000100010000001001000101111101001000010010001000000000000000000000000
000001000000000011110000100010000001001000101111101001000010010001000000000000000000000000
000000000000000000000000000000000000000000000000000000000000000000000000000000000000000000
000000000000000000000000000000000000000000000000000000000000000000000000000000000000000000
000000000000000000000000000000000000000000000000000000000000000000000000000000000000000000
000000000000000000000000000000000000000000000000000000000000000000000000000000000000000000
000000000000000000000000000000000000000000000000000000000000000000000000000000000000000000
>Q9M3K7
MDKSKRPFIKSKRSFRRRLPPIQSGDRIDYRNMSLISRFISEQGKILSRRVNRLTLKQQRLITSAIKQARILSLLPFLNNEKQFERTESTTRTANFRTKNK
00000000000000000000000000000000000000000000111000001001101100100010011000001100000000000000000000000
00000000000000000000000000000000000000000000111000001001101100100010011000001100000000000000000000000
00000000000000000000000000000000000000000000000000000000000000000000000000000000000000000000000000000
00000000000000000000000000000000000000000000000000000000000000000000000000000000000000000000000000000
00000000000000000000000000000000000000000000000000000000000000000000000000000000000000000000000000000
00000000000000000000000000000000000000000000000000000000000000000000000000000000000000000000000000000
00000000000000000000000000000000000000000000000000000000000000000000000000000000000000000000000000000
>Q9P0J6
MANLFIRKMVNPLLYLSRHTVKPRALSTFLFGSIRGAAPVAVEPGAAVRSLLSPGLLPHLLPALGFKNKTVLKKRCKDCYLVKRRGRWYVYCKTHPRHKQRQM
0000000000000000000000000000000000000000000000000000000000000000001111100110100011111111101000011010110
0000000000000000000000000000000000000000000000000000000000000000001111100110100011111111101000011010110
0000000000000000000000000000000000000000000000000000000000000000000000000000000000000000000000000000000
0000000000000000000000000000000000000000000000000000000000000000000000000000000000000000000000000000000
0000000000000000000000000000000000000000000000000000000000000000000000000000000000000000000000000000000
0000000000000000000000000000000000000000000000000000000000000000000000000000000000000000000000000000000
0000000000000000000000000000000000000000000000000000000000000000000000000000000000000000000000000000000
>Q9RT21
MAELISKEGNKVEFKVSVPAAEVNRAYDQVWAGLARDVRVPGFRPGKAPRKVIENRVGKGYVESQVRDRLLETHYSQGLRELGLNLVDATVDPQDVQSGQAFEFTVKGETYPEVKLGDWQGLKVSAQAPEITDEVLEQTLSDLRERNASFEKAERPIEAADQVTIQELGEGDSEE
GGSYPIYLDMAEEHVRNALLGKSAGDVVDITVPAHQHGDHEHAEHTVRVKVVEVSSKKLQDLNDEFATSLNYESMDKLRTDLREELERRAQQEGDNLRREELVGHLVEGMTVEIPQALIDRRREGMMSEIQDDLRRQGVQWKEYEAFMQEQGKLDEFEADLTKNAETRVRRDLAL
EQLATDLNAQVNEAEFNQTLMNLAQANGMNVQQLVQQLGQDGVQSYYISLLRERGLQRALAQLSGEGQSTEAASPKATGTEAAGTEQSEPAQTETAQNDAGQTETAQSEGEQQSE
0000000000000000000000000000000000000010100100000000000000000000000000000000000000000000000000000000000000000000000000000000000000000000000000000000000000000000000000000000000
0000000000000000000000000000000000000000000000000000000000000000000000000000000000000000000000000000000000000000000000000000000000000000000000000000000000000000000000000000000
0000000000000000000000000000000000000000000000000000000000000000000000000000000000000000000000000000000000000000000
0000000000000000000000000000000000000010100100000000000000000000000000000000000000000000000000000000000000000000000000000000000000000000000000000000000000000000000000000000000
0000000000000000000000000000000000000000000000000000000000000000000000000000000000000000000000000000000000000000000000000000000000000000000000000000000000000000000000000000000
0000000000000000000000000000000000000000000000000000000000000000000000000000000000000000000000000000000000000000000
0000000000000000000000000000000000000000000000000000000000000000000000000000000000000000000000000000000000000000000000000000000000000000000000000000000000000000000000000000000
0000000000000000000000000000000000000000000000000000000000000000000000000000000000000000000000000000000000000000000000000000000000000000000000000000000000000000000000000000000
0000000000000000000000000000000000000000000000000000000000000000000000000000000000000000000000000000000000000000000
0000000000000000000000000000000000000000000000000000000000000000000000000000000000000000000000000000000000000000000000000000000000000000000000000000000000000000000000000000000
0000000000000000000000000000000000000000000000000000000000000000000000000000000000000000000000000000000000000000000000000000000000000000000000000000000000000000000000000000000
0000000000000000000000000000000000000000000000000000000000000000000000000000000000000000000000000000000000000000000
0000000000000000000000000000000000000000000000000000000000000000000000000000000000000000000000000000000000000000000000000000000000000000000000000000000000000000000000000000000
0000000000000000000000000000000000000000000000000000000000000000000000000000000000000000000000000000000000000000000000000000000000000000000000000000000000000000000000000000000
0000000000000000000000000000000000000000000000000000000000000000000000000000000000000000000000000000000000000000000
0000000000000000000000000000000000000000000000000000000000000000000000000000000000000000000000000000000000000000000000000000000000000000000000000000000000000000000000000000000
0000000000000000000000000000000000000000000000000000000000000000000000000000000000000000000000000000000000000000000000000000000000000000000000000000000000000000000000000000000
0000000000000000000000000000000000000000000000000000000000000000000000000000000000000000000000000000000000000000000
0000000000000000000000000000000000000000000000000000000000000000000000000000000000000000000000000000000000000000000000000000000000000000000000000000000000000000000000000000000
0000000000000000000000000000000000000000000000000000000000000000000000000000000000000000000000000000000000000000000000000000000000000000000000000000000000000000000000000000000
0000000000000000000000000000000000000000000000000000000000000000000000000000000000000000000000000000000000000000000

>Q9WZH4
MRVITTHRSPDFDAFASCVAAKKLLDDHIIVLPSNPARNLSDFLKVYSDRFEFVWDHEFEGEITELVIVDAPSLDRIPESIRKKAQGAKITVYDHHVDESPYDGIVSKVGATITILVELIREKNIPLDPTEATLFMIALYDDTGNLLFSSTTPRDLEIAKFLLENGANLDEVALY
TREELTPRQMELLDDLIENARDYEVNGVPITISMIECEDFVGGLGLIVSKAWEMMGKETFIAIVKMGKKIYVIGRTSSPDVDLGSLMKDLGGGGHTRAASATITGKEIDEVLKEVLNRLHDHVVPLLRARDIMSSPVKVVLSNMTIKEVDRLMKQTGHSGFPVVEGNRLVGIVTK
KAVEKAMNHGLGDRPVKSIMSTNLVVASPDTPVTRLRELMVEHAIGRIPILENGILVGIVTRSDVLRAIFGKPFKKYVMPVFQANGQIFRDVSKLLVERVDPKILNLFRLLGKFGDEVNMPVYVVGGFVRDLLLGIKNLDIDIVVEGNALEFAEYAKRFLPGKLVKHDKFMTASL
FLKGGLRIDIATARLEYYESPAKLPDVEMSTIKKDLYRRDFTINAMAIKLNPKDFGLLIDFFGGYRDLKEGVIRVLHTLSFVDDPTRILRAIRFEQRFDFRIEETTERLLKQAVEEGYLERTTGPRLRQELEKILEEKNPLKSIRRMAQFDVIKHLFPKTYYTPSMDEKMENLFR
NIPWVEENFGEVDRFYAVLHVFLEFYDDESWKEVRDRYSLRRNLINEIRHVEKSAPALLEMLSERVPASFVYPLVKGVSNETICHFLAYLSGEKEGLFKSYLLKIKNTKLEKINGEYLIRKGITSGKIIGEVLEKILMKKLDGDTRDEEEILEEVLASLETEG
0000000000000000000000000000000000000000000000000000000000000000000000000000000000000000000000000000000000000000000000000000000000000000000000000000000000000000000000000000000
0000000000000000000000000000000000000000000000000000000000000000000000000000000000000000000000000000000000000000000000000000000000000000000000000000000000000000000000000000000
0000000000000000000000000000000000000000000000000000000000000000000000000000000000000000000000000000000000000000000000000000000000000000000000000000000000000000000000100101000
0000001000100000000000000000000000000111000000000000000000000000000000000000000000000000001000000000000000000000000000000000000000000011010000000000000000000000000000000000000000000000
0000000000000001000000000000000000000010111000100000000000000000000000000001000000000000000000000000000000000000011100000000011011000100000000000000000000000000000
0000000000000000000000000000000000000000000000000000000000000000000000000000000000000000000000000000000000000000000000000000000000000000000000000000000000000000000000000000000
0000000000000000000000000000000000000000000000000000000000000000000000000000000000000000000000000000000000000000000000000000000000000000000000000000000000000000000000000000000
0000000000000000000000000000000000000000000000000000000000000000000000000000000000000000000000000000000000000000000000000000000000000000000000000000000000000000000000000000000
0000000000000000000000000000000000000000000000000000000000000000000000000000000000000000000000000000000000000000000000000000000000000000000000000000000000000000000000000000000
0000000000000000000000000000000000000000000000000000000000000000000000000000000000000000000000000000000000000000000000000000000000000000000000000000000000000000000
0000000000000000000000000000000000000000000000000000000000000000000000000000000000000000000000000000000000000000000000000000000000000000000000000000000000000000000000000000000
0000000000000000000000000000000000000000000000000000000000000000000000000000000000000000000000000000000000000000000000000000000000000000000000000000000000000000000000000000000
0000000000000000000000000000000000000000000000000000000000000000000000000000000000000000000000000000000000000000000000000000000000000000000000000000000000000000000000100101000
0000001000100000000000000000000000000111000000000000000000000000000000000000000000000000001000000000000000000000000000000000000000000011010000000000000000000000000000000000000000000000
0000000000000001000000000000000000000010111000100000000000000000000000000001000000000000000000000000000000000000011100000000011011000100000000000000000000000000000
0000000000000000000000000000000000000000000000000000000000000000000000000000000000000000000000000000000000000000000000000000000000000000000000000000000000000000000000000000000
0000000000000000000000000000000000000000000000000000000000000000000000000000000000000000000000000000000000000000000000000000000000000000000000000000000000000000000000000000000
0000000000000000000000000000000000000000000000000000000000000000000000000000000000000000000000000000000000000000000000000000000000000000000000000000000000000000000000000000000
0000000000000000000000000000000000000000000000000000000000000000000000000000000000000000000000000000000000000000000000000000000000000000000000000000000000000000000000000000000
0000000000000000000000000000000000000000000000000000000000000000000000000000000000000000000000000000000000000000000000000000000000000000000000000000000000000000000
0000000000000000000000000000000000000000000000000000000000000000000000000000000000000000000000000000000000000000000000000000000000000000000000000000000000000000000000000000000
0000000000000000000000000000000000000000000000000000000000000000000000000000000000000000000000000000000000000000000000000000000000000000000000000000000000000000000000000000000
0000000000000000000000000000000000000000000000000000000000000000000000000000000000000000000000000000000000000000000000000000000000000000000000000000000000000000000000000000000
0000000000000000000000000000000000000000000000000000000000000000000000000000000000000000000000000000000000000000000000000000000000000000000000000000000000000000000000000000000
0000000000000000000000000000000000000000000000000000000000000000000000000000000000000000000000000000000000000000000000000000000000000000000000000000000000000000000
0000000000000000000000000000000000000000000000000000000000000000000000000000000000000000000000000000000000000000000000000000000000000000000000000000000000000000000000000000000
0000000000000000000000000000000000000000000000000000000000000000000000000000000000000000000000000000000000000000000000000000000000000000000000000000000000000000000000000000000
0000000000000000000000000000000000000000000000000000000000000000000000000000000000000000000000000000000000000000000000000000000000000000000000000000000000000000000000000000000
0000000000000000000000000000000000000000000000000000000000000000000000000000000000000000000000000000000000000000000000000000000000000000000000000000000000000000000000000000000
0000000000000000000000000000000000000000000000000000000000000000000000000000000000000000000000000000000000000000000000000000000000000000000000000000000000000000000
0000000000000000000000000000000000000000000000000000000000000000000000000000000000000000000000000000000000000000000000000000000000000000000000000000000000000000000000000000000
0000000000000000000000000000000000000000000000000000000000000000000000000000000000000000000000000000000000000000000000000000000000000000000000000000000000000000000000000000000
0000000000000000000000000000000000000000000000000000000000000000000000000000000000000000000000000000000000000000000000000000000000000000000000000000000000000000000000000000000
0000000000000000000000000000000000000000000000000000000000000000000000000000000000000000000000000000000000000000000000000000000000000000000000000000000000000000000000000000000
0000000000000000000000000000000000000000000000000000000000000000000000000000000000000000000000000000000000000000000000000000000000000000000000000000000000000000000
>Q9Y6G3
MAVAAVKWVMSKRTILKHLFPVQNGALYCVCHKSTYSPLPDDYNCNVELALTSDGRTIVCYHPSVDIPYEHTKPIPRPDPVHNNEETHDQVLKTRLEEKVEHLEEGPMIEQLSKMFFTTKHRWYPHGRYHRCRKNLNPPKDR
0000000000000000000000000000000000000000000000000000000000000000000000000000000000000000000000000000000000000000000000011001111111001100010000
0000000000000000000000000000000000000000000000000000000000000000000000000000000000000000000000000000000000000000000000011001111111001100010000
0000000000000000000000000000000000000000000000000000000000000000000000000000000000000000000000000000000000000000000000000000000000000000000000
0000000000000000000000000000000000000000000000000000000000000000000000000000000000000000000000000000000000000000000000000000000000000000000000
0000000000000000000000000000000000000000000000000000000000000000000000000000000000000000000000000000000000000000000000000000000000000000000000
0000000000000000000000000000000000000000000000000000000000000000000000000000000000000000000000000000000000000000000000000000000000000000000000
0000000000000000000000000000000000000000000000000000000000000000000000000000000000000000000000000000000000000000000000000000000000000000000000
>P0DJ23
MAQRITYRRRCSYNTRSNKVRKVKTPGGRLVAQYVKKVVNYTKCSEAGCNVALNGIAQVRPAEYATIARSAKTVSRVYGGELCHTCVRSRIIRAFLTEEVKIVKRKMISKPKTDKKKKRSQKK
010111111111111111010111111111110111111110100101100000111010011011100110111111101100100000100100000001001000100000000000000000
010111111111111111010111111111110111111110100101100000111010011011100110111111101100100000100100000001001000100000000000000000
000000000000000000000000000000000000000000000000000000000000000000000000000000000000000000000000000000000000000000000000000
000000000000000000000000000000000000000000000000000000000000000000000000000000000000000000000000000000000000000000000000000
000000000000000000000000000000000000000000000000000000000000000000000000000000000000000000000000000000000000000000000000000
000000000000000000000000000000000000000000000000000000000000000000000000000000000000000000000000000000000000000000000000000
000000000000000000000000000000000000000000000000000000000000000000000000000000000000000000000000000000000000000000000000000
>P0DJ25
MQIFVKTLTGKTITLDVEASDTIENVKAKIQDKEGIPPDQQRLIFAGKQLEDGRTLSDYNIQKESTLHLVLRLRGGGMEPTIAALAKKYNCEKKVCRDCYARLPPKATNCRKRKCGHSNSLRLKKKPKE
000000000000000000000000000000000000000000000000000000000000000000000000000000000000000000001010100111010001101111111010010110010
000000000000000000000000000000000000000000000000000000000000000000000000000000000000000000001010100111010001101111111010010110010
000000000000000000000000000000000000000000000000000000000000000000000000000000000000000000000000000000000000000000000000000000000
000000000000000000000000000000000000000000000000000000000000000000000000000000000000000000000000000000000000000000000000000000000
000000000000000000000000000000000000000000000000000000000000000000000000000000000000000000000000000000000000000000000000000000000
000000000000000000000000000000000000000000000000000000000000000000000000000000000000000000000000000000000000000000000000000000000
000000000000000000000000000000000000000000000000000000000000000000000000000000000000000000000000000000000000000000000000000000000
>P0DJ58

MKHNNQLPVAQLRKHQQFRIKTFFNQAAQKKARLHARRAQAAAVFPRPTEKLQPVVRKQTQRYNKSTKLGRGFTLQELKAAGISAAFAQSIGIKVDHRRKNRCQESLELNKKRLLAYVSKLVLFPRHQGKAKKGLVNDTADTSSAAQNALQTSVPLPSVSKREKAVSNIAELRKK
KVYRIIRQEKTNQKWDGKRKAKAQAAAEPKA
1110110100011110001010000110010011001100000000000000100111110111100101100010001000010000000001011111011000000000000000000000010000110000000000000000000000000000011010000000000
0011001101111110111001000000000
1110110100011110001010000110010011001100000000000000100111110111100101100010001000010000000001011111011000000000000000000000010000110000000000000000000000000000011010000000000
0011001101111110111001000000000
0000000000000000000000000000000000000000000000000000000000000000000000000000000000000000000000000000000000000000000000000000000000000000000000000000000000000000000000000000000
0000000000000000000000000000000
0000000000000000000000000000000000000000000000000000000000000000000000000000000000000000000000000000000000000000000000000000000000000000000000000000000000000000000000000000000
0000000000000000000000000000000
0000000000000000000000000000000000000000000000000000000000000000000000000000000000000000000000000000000000000000000000000000000000000000000000000000000000000000000000000000000
0000000000000000000000000000000
0000000000000000000000000000000000000000000000000000000000000000000000000000000000000000000000000000000000000000000000000000000000000000000000000000000000000000000000000000000
0000000000000000000000000000000
0000000000000000000000000000000000000000000000000000000000000000000000000000000000000000000000000000000000000000000000000000000000000000000000000000000000000000000000000000000
0000000000000000000000000000000
>P41250
MPSPRPVLLRGARAALLLLLPPRLLARPSLLLRRSLSAASCPPISLPAAASRSSMDGAGAEEVLAPLRLAVRQQGDLVRKLKEDKAPQVDVDKAVAELKARKRVLEAKELALQPKDDIVDRAKMEDTLKRRFFYDQAFAIYGGVSGLYDFGPVGCALKNNIIQTWRQHFIQEEQI
LEIDCTMLTPEPVLKTSGHVDKFADFMVKDVKNGECFRADHLLKAHLQKLMSDKKCSVEKKSEMESVLAQLDNYGQQELADLFVNYNVKSPITGNDLSPPVSFNLMFKTFIGPGGNMPGYLRPETAQGIFLNFKRLLEFNQGKLPFAAAQIGNSFRNEISPRSGLIRVREFTMAE
IEHFVDPSEKDHPKFQNVADLHLYLYSAKAQVSGQSARKMRLGDAVEQGVINNTVLGYFIGRIYLYLTKVGISPDKLRFRQHMENEMAHYACDCWDAESKTSYGWIEIVGCADRSCYDLSCHARATKVPLVAEKPLKEPKTVNVVQFEPSKGAIGKAYKKDAKLVMEYLAICDEC
YITEMEMLLNEKGEFTIETEGKTFQLTKDMINVKRFQKTLYVEEVVPNVIEPSFGLGRIMYTVFEHTFHVREGDEQRTFFSFPAVVAPFKCSVLPLSQNQEFMPFVKELSEALTRHGVSHKVDDSSGSIGRRYARTDEIGVAFGVTIDFDTVNKTPHTATLRDRDSMRQIRAEIS
ELPSIVQDLANGNITWADVEARYPLFEGQETGKKETIEE
0000000000000000000000000000000000000000000000000000000000000000000000000000000000000000000000000000000000000000000000000000000000000000000000000000000000000000000000000000000
0000000000000000000000000000000000000000000000000000000000000000000000000000000000000000000000000000000010000000100010000010000000000110010000100000000000001001101000010000000
0000000000000000000000000000000000000000000000000000000000000000000000000000010000000000000000000000000000000000000000000000000000000001000000000000000000000000000000000000000000
0000000000000000000000000000000000000000000000000000000000000000000000000000000000000000000000000000000000000000000000000000000000000000000000000000000000000000000000000000000
000000000000000000000000000000000000000
0000000000000000000000000000000000000000000000000000000000000000000000000000000000000000000000000000000000000000000000000000000000000000000000000000000000000000000000000000000
0000000000000000000000000000000000000000000000000000000000000000000000000000000000000000000000000000000000000000000000000000000000000000000000000000000000000000000000000000000
0000000000000000000000000000000000000000000000000000000000000000000000000000000000000000000000000000000000000000000000000000000000000000000000000000000000000000000000000000000
0000000000000000000000000000000000000000000000000000000000000000000000000000000000000000000000000000000000000000000000000000000000000000000000000000000000000000000000000000000
000000000000000000000000000000000000000
0000000000000000000000000000000000000000000000000000000000000000000000000000000000000000000000000000000000000000000000000000000000000000000000000000000000000000000000000000000
0000000000000000000000000000000000000000000000000000000000000000000000000000000000000000000000000000000010000000100010000010000000000110010000100000000000001001101000010000000
0000000000000000000000000000000000000000000000000000000000000000000000000000010000000000000000000000000000000000000000000000000000000001000000000000000000000000000000000000000000
0000000000000000000000000000000000000000000000000000000000000000000000000000000000000000000000000000000000000000000000000000000000000000000000000000000000000000000000000000000
000000000000000000000000000000000000000
0000000000000000000000000000000000000000000000000000000000000000000000000000000000000000000000000000000000000000000000000000000000000000000000000000000000000000000000000000000
0000000000000000000000000000000000000000000000000000000000000000000000000000000000000000000000000000000000000000000000000000000000000000000000000000000000000000000000000000000
0000000000000000000000000000000000000000000000000000000000000000000000000000000000000000000000000000000000000000000000000000000000000000000000000000000000000000000000000000000
0000000000000000000000000000000000000000000000000000000000000000000000000000000000000000000000000000000000000000000000000000000000000000000000000000000000000000000000000000000
000000000000000000000000000000000000000
0000000000000000000000000000000000000000000000000000000000000000000000000000000000000000000000000000000000000000000000000000000000000000000000000000000000000000000000000000000
0000000000000000000000000000000000000000000000000000000000000000000000000000000000000000000000000000000000000000000000000000000000000000000000000000000000000000000000000000000
0000000000000000000000000000000000000000000000000000000000000000000000000000000000000000000000000000000000000000000000000000000000000000000000000000000000000000000000000000000
0000000000000000000000000000000000000000000000000000000000000000000000000000000000000000000000000000000000000000000000000000000000000000000000000000000000000000000000000000000
000000000000000000000000000000000000000
0000000000000000000000000000000000000000000000000000000000000000000000000000000000000000000000000000000000000000000000000000000000000000000000000000000000000000000000000000000
0000000000000000000000000000000000000000000000000000000000000000000000000000000000000000000000000000000000000000000000000000000000000000000000000000000000000000000000000000000
0000000000000000000000000000000000000000000000000000000000000000000000000000000000000000000000000000000000000000000000000000000000000000000000000000000000000000000000000000000
0000000000000000000000000000000000000000000000000000000000000000000000000000000000000000000000000000000000000000000000000000000000000000000000000000000000000000000000000000000
000000000000000000000000000000000000000
0000000000000000000000000000000000000000000000000000000000000000000000000000000000000000000000000000000000000000000000000000000000000000000000000000000000000000000000000000000
0000000000000000000000000000000000000000000000000000000000000000000000000000000000000000000000000000000000000000000000000000000000000000000000000000000000000000000000000000000
0000000000000000000000000000000000000000000000000000000000000000000000000000000000000000000000000000000000000000000000000000000000000000000000000000000000000000000000000000000
0000000000000000000000000000000000000000000000000000000000000000000000000000000000000000000000000000000000000000000000000000000000000000000000000000000000000000000000000000000
000000000000000000000000000000000000000
>Q24C27
MVFNKFVQVGRVVYINYGADKGKLAVIVNIINQNRILIDGEHIVRQVIPIRRVHLTKFQIDNVELNQRTVLLKKKIAKFDLTKKYAETSFAKKQAIKTKRANLGDFDRFRVMVLKKKLARTSAKSK
010100000000000110000000000010000110000000000000101101001000000111110111000000000000000001000000100000000000100110111001000000
010100000000000110000000000010000110000000000000101101001000000111110111000000000000000001000000100000000000100110111001000000
000000000000000000000000000000000000000000000000000000000000000000000000000000000000000000000000000000000000000000000000000000
000000000000000000000000000000000000000000000000000000000000000000000000000000000000000000000000000000000000000000000000000000
000000000000000000000000000000000000000000000000000000000000000000000000000000000000000000000000000000000000000000000000000000
000000000000000000000000000000000000000000000000000000000000000000000000000000000000000000000000000000000000000000000000000000
000000000000000000000000000000000000000000000000000000000000000000000000000000000000000000000000000000000000000000000000000000
>O32225
MNKTAANNQRIMPIVKDAFRIHANPFPWKKAIGSGFASGFPVLVGALAGHTDYGLIASIGGFVYLYAGGESYKKRALKLLLVSIGIALSFGLGTLLSGMLWMMAAVLGLIGAAAMFIFSALGIQGPAPMFFVLAFLVSSGLPADPSQALFRAGLTFLGGIFAMGIALIGWLWNKH
GPEGAVLQKTYHQLAACLSAVGTPGFHNAQHQTVLMLRTARQTVLGKGNRRKKSRHDERFFRLLEKADDIFIAIIHFSAETPDRETAQIEKALRQTGDMLANARVFNTFQINENESEPRRKLFEEINEVLYIASGRFDKQLDGEPSRLPSSFYFLRNAFDWKSPVLIRALKYGIV

LFAADMFALAFGFARSYWIPLSAAAVMLGTTVVFTLHRAIQRSAGTVIGVILAGAILFFKPGGVYIAFCIAALQCLLEMLIVRNYALAVPFLTANALVITESMHGGAGAGYFMIARLTDVAVGSVIGLLGTMLLWRRFSTKRLPELVRNVIRLEGQFMEKLLTGQIADEHKLRVS
LVRLRDAYDKALGEFPHANADALWPAISGAQHLGYYLLSAQAHRRPSAPFSDEEINQLRAFFEQAEQSFIMKRMPADIQVPHVPHYPRISKELSALYSGLRTAFEK
0000000000000000000000000000000000000000000000000000000000000000000000000000000000000000000000000000000000000000000000000000000000000000000000000000000000000000000000000000000
0000000000000000000000000000000000000000000000000000000000000000000000000000000000000000000000000000000000000000000000000000000000000000000000000000000000000000000000000000000
0000000000000000000000000000000000000000000000000000000000000000000000000000000000000000000000000000000000000000000000000000000000000000000000000000000000000000000000000000000
0000000000000000000000000000000000000000000000000000000000000000000000000000000000000000000000000000000000
0000000000000000000000000000000000000000000000000000000000000000000000000000000000000000000000000000000000000000000000000000000000000000000000000000000000000000000000000000000
0000000000000000000000000000000000000000000000000000000000000000000000000000000000000000000000000000000000000000000000000000000000000000000000000000000000000000000000000000000
0000000000000000000000000000000000000000000000000000000000000000000000000000000000000000000000000000000000000000000000000000000000000000000000000000000000000000000000000000000
0000000000000000000000000000000000000000000000000000000000000000000000000000000000000000000000000000000000
0000000000000000000000000000000000000000000000000000000000000000000000000000000000000000000000000000000000000000000000000000000000000000000000000000000000000000000000000000000
0000000000000000000000000000000000000000000000000000000000000000000000000000000000000000000000000000000000000000000000000000000000000000000000000000000000000000000000000000000
0000000000000000000000000000000000000000000000000000000000000000000000000000000000000000000000000000000000000000000000000000000000000000000000000000000000000000000000000000000
0000000000000000000000000000000000000000000000000000000000000000000000000000000000000000000000000000000000
0000000000000000000000000000000000000000000000000000000000000000000000000000000000000000000000000000000000000000000000000000000000000000000000000000000000000000000000000000000
0000000000000000000000000000000000000000000000000000000000000000000000000000000000000000000000000000000000000000000000000000000000000000000000000000000000000000000000000000000
0000000000000000000000000000000000000000000000000000000000000000000000000000000000000000000000000000000000000000000000000000000000000000000000000000000000000000000000000000000
0000000000000000000000000000000000000000000000000000000000000000000000000000000000000000000000000000000000
0000000000000000000000000000000000000000000000000000000000000000000000000000000000000000000000000000000000000000000000000000000000000000000000000000000000000000000000000000000
0000000000000000000000000000000000000000000000000000000000000000000000000000000000000000000000000000000000000000000000000000000000000000000000000000000000000000000000000000000
0000000000000000000000000000000000000000000000000000000000000000000000000000000000000000000000000000000000000000000000000000000000000000000000000000000000000000000000000000000
0000000000000000000000000000000000000000000000000000000000000000000000000000000000000000000000000000000000
0000000000000000000000000000000000000000000000000000000000000000000000000000000000000000000000000000000000000000000000000000000000000000000000000000000000000000000000000000000
0000000000000000000000000000000000000000000000000000000000000000000000000000000000000000000000000000000000000000000000000000000000000000000000000000000000000000000000000000000
0000000000000000000000000000000000000000000000000000000000000000000000000000000000000000000000000000000000000000000000000000000000000000000000000000000000000000000000000000000
0000000000000000000000000000000000000000000000000000000000000000000000000000000000000000000000000000000000
0000000000000000000000000000000000000000000000000000000000000000000000000000000000000000000000000000000000000000000000000000000000000000000000000000000000000000000000000000000
0000000000000000000000000000000000000000000000000000000000000000000000000000000000000000000000000000000000000000000000000000000000000000000000000000000000000000000000000000000
0000000000000000000000000000000000000000000000000000000000000000000000000000000000000000000000000000000000000000000000000000000000000000000000000000000000000000000000000000000
0000000000000000000000000000000000000000000000000000000000000000000000000000000000000000000000000000000000
>A2XDG4
MSRPNTRNKSKRPRADDCESPSAVFKKIHSTGAITKGDIKQLYMVWKPVCHGCHGNSKDSPNCFCGLIPAANGVRKSGLWQRTNEIIRALGPNPSTDLRDSTETPAGLTNLGATCYANSILQCLYMNTSFRLGIFSLEPDILKMHPVLDQLARLFAQLHSSKMAFIDSAPFIKTL
ELDNGVQQDSHEFLTLFLSLLEGSLSHSKVPGARTIVQHLFRGSVSHVTRCSSCGRDSEASSKMEDFYELELNIKGLNNLEQSLDDYLSTEALDGENQYFCESCQKRVDATRCIKLRSLPPVVNFQLKRYVFLPKTTTKKKISSAFSFPGQLDMGKRLSNPSSSYTYGLSAILIH
KGSAANSGHYVAHVKDESNGQWWEFDDEHVSKLGLHPFGEKPGKSSNKTDQKPQGSSTADSVTNDDNNSCHEAAFTSTMEEMFSSTDAYMLMYKRIAKDENGIESNNISSNNSLPHHFVDEIDERNTSYVKECEEYESKKDVHLAYITERRQEVKSVLTEAPATPEEDSYFWIST
DWLRQWADNVNPPSPIITGVRVHSSIDNSPIQCEHGKVPASKVTSMKRLSAGAWHKLFSKYGGGPTLSSDDFCMECLKDGAKNSVSADVYRDRKASLRSIAEAALAGNNPDGPLYFVSRPWLTQWLRRKNVDIPSDADSGPTIALTCTHGNLLPEHASGAKRVTVPEDLWLFLYE
TSGMKIDDIVTFPSDSQPCGICSQQLSVVASVEDNLRAVKLKQRQSHEKLTSGKSLALHPGQKYYLVPSSWLSEWRAYITATGKNISSLPEPQSLEVTINSLICEKHSRLLQRPLDLVCKRGTITQKASNTDGLTMISESDWILFSEEWNVAHGKGLCAEIVFSKSSQDNLQSSE
AVPILVEDLDQSTNDLSNDLGGREPYVRTDPEVCEECIGEKESCALVEKLNYQNEDIQVYLVRGKEAPKSIREASAAVPVPDRRTSKRSRRTTSGNSISLKVSGSTTVYQLKLMIWESLGIVKENQELHKGSVEIEDDFATLADKCIFPGDVLWVKDSEIYENRDIADEISEQKV
VVQTEEGFRGTLLTSSASAQLCQDISFSD
0000000000000000000000000000000000000000000000000000000000000000000000000000000000000000000000000000000000000000000000000000000000000000000000000000000000000000000000000000000
0000000000000000000000000000000000000000000000000000000000000000000000000000000000000000000000000000000000000000000000000000000000000000000000000000000000000000000000000000000
0000000000000000000000000000000000000000000000000000000000000000000000000000000000000000000000000000000000000000000000000000000000000000000000000000000000000000000000000000000
0000000000000000000000000000000000000000000000000000000000000000000000000000000000000000000000000000000000000000000000000000000000000000000000000000000000000000000000000000000
0000000000000000000000000000000000000000000000000000000000000000000000000000000000000000000000000000000000000000000000000000000000000000000000000000000000000000000000000000000
0000000000000000000000000000000000000000000000000000000000000000000000000000000000000000000000000000000000000000000000000000000000000000000000000000000000000000000000000000000
00000000000000000000000000000
0000000000000000000000000000000000000000000000000000000000000000000000000000000000000000000000000000000000000000000000000000000000000000000000000000000000000000000000000000000
0000000000000000000000000000000000000000000000000000000000000000000000000000000000000000000000000000000000000000000000000000000000000000000000000000000000000000000000000000000
0000000000000000000000000000000000000000000000000000000000000000000000000000000000000000000000000000000000000000000000000000000000000000000000000000000000000000000000000000000
0000000000000000000000000000000000000000000000000000000000000000000000000000000000000000000000000000000000000000000000000000000000000000000000000000000000000000000000000000000
0000000000000000000000000000000000000000000000000000000000000000000000000000000000000000000000000000000000000000000000000000000000000000000000000000000000000000000000000000000
0000000000000000000000000000000000000000000000000000000000000000000000000000000000000000000000000000000000000000000000000000000000000000000000000000000000000000000000000000000
00000000000000000000000000000
0000000000000000000000000000000000000000000000000000000000000000000000000000000000000000000000000000000000000000000000000000000000000000000000000000000000000000000000000000000
0000000000000000000000000000000000000000000000000000000000000000000000000000000000000000000000000000000000000000000000000000000000000000000000000000000000000000000000000000000
0000000000000000000000000000000000000000000000000000000000000000000000000000000000000000000000000000000000000000000000000000000000000000000000000000000000000000000000000000000
0000000000000000000000000000000000000000000000000000000000000000000000000000000000000000000000000000000000000000000000000000000000000000000000000000000000000000000000000000000
0000000000000000000000000000000000000000000000000000000000000000000000000000000000000000000000000000000000000000000000000000000000000000000000000000000000000000000000000000000
0000000000000000000000000000000000000000000000000000000000000000000000000000000000000000000000000000000000000000000000000000000000000000000000000000000000000000000000000000000
00000000000000000000000000000
0000000000000000000000000000000000000000000000000000000000000000000000000000000000000000000000000000000000000000000000000000000000000000000000000000000000000000000000000000000
0000000000000000000000000000000000000000000000000000000000000000000000000000000000000000000000000000000000000000000000000000000000000000000000000000000000000000000000000000000
0000000000000000000000000000000000000000000000000000000000000000000000000000000000000000000000000000000000000000000000000000000000000000000000000000000000000000000000000000000
0000000000000000000000000000000000000000000000000000000000000000000000000000000000000000000000000000000000000000000000000000000000000000000000000000000000000000000000000000000
0000000000000000000000000000000000000000000000000000000000000000000000000000000000000000000000000000000000000000000000000000000000000000000000000000000000000000000000000000000
0000000000000000000000000000000000000000000000000000000000000000000000000000000000000000000000000000000000000000000000000000000000000000000000000000000000000000000000000000000
00000000000000000000000000000
0000000000000000000000000000000000000000000000000000000000000000000000000000000000000000000000000000000000000000000000000000000000000000000000000000000000000000000000000000000
0000000000000000000000000000000000000000000000000000000000000000000000000000000000000000000000000000000000000000000000000000000000000000000000000000000000000000000000000000000
0000000000000000000000000000000000000000000000000000000000000000000000000000000000000000000000000000000000000000000000000000000000000000000000000000000000000000000000000000000

0000000000000000000000000000000000000000000000000000000000000000000000000000000000000000000000000000000000000000000000000000000000000000000000000000000000000000000000000000000
0000000000000000000000000000000000000000000000000000000000000000000000000000000000000000000000000000000000000000000000000000000000000000000000000000000000000000000000000000000
0000000000000000000000000000000000000000000000000000000000000000000000000000000000000000000000000000000000000000000000000000000000000000000000000000000000000000000000000000000
00000000000000000000000000000
0000000000000000000000000000000000000000000000000000000000000000000000000000000000000000000000000000000000000000000000000000000000000000000000000000000000000000000000000000000
0000000000000000000000000000000000000000000000000000000000000000000000000000000000000000000000000000000000000000000000000000000000000000000000000000000000000000000000000000000
0000000000000000000000000000000000000000000000000000000000000000000000000000000000000000000000000000000000000000000000000000000000000000000000000000000000000000000000000000000
0000000000000000000000000000000000000000000000000000000000000000000000000000000000000000000000000000000000000000000000000000000000000000000000000000000000000000000000000000000
0000000000000000000000000000000000000000000000000000000000000000000000000000000000000000000000000000000000000000000000000000000000000000000000000000000000000000000000000000000
0000000000000000000000000000000000000000000000000000000000000000000000000000000000000000000000000000000000000000000000000000000000000000000000000000000000000000000000000000000
00000000000000000000000000000
0000000000000000000000000000000000000000000000000000000000000000000000000000000000000000000000000000000000000000000000000000000000000000000000000000000000000000000000000000000
0000000000000000000000000000000000000000000000000000000000000000000000000000000000000000000000000000000000000000000000000000000000000000000000000000000000000000000000000000000
0000000000000000000000000000000000000000000000000000000000000000000000000000000000000000000000000000000000000000000000000000000000000000000000000000000000000000000000000000000
0000000000000000000000000000000000000000000000000000000000000000000000000000000000000000000000000000000000000000000000000000000000000000000000000000000000000000000000000000000
0000000000000000000000000000000000000000000000000000000000000000000000000000000000000000000000000000000000000000000000000000000000000000000000000000000000000000000000000000000
0000000000000000000000000000000000000000000000000000000000000000000000000000000000000000000000000000000000000000000000000000000000000000000000000000000000000000000000000000000
00000000000000000000000000000
>C0SUT9
MGTELMRICVKEDSDDLPSVPPGFESYATFTLKRVVPATTSDKAKTPAIESVSATEQAKMEVESDEAKAARALRRRPWINHSGCDDDGDCAANNDNAASQNPDQNCDVKPALPKGVVRGCEECKDCQKVTARWHPDEARRPDLEDAPVFYPSEEEFEDTLNYIAKIRPEAEKYGI
CRIVPPPSWKPPCPLKEKQVWEGSKFTTRVQRVDKLQNRSSMKKISKLPNQMRKKKRKCMKMGMDSVTNGMGDPCSASTGMNELETFGFEPGPGFTLKDFQKYADEFKAQYFKKSETSTDDKCKVDNSIDCWEPALEDVEGEYWRIVDKATEEIEVLYGADLETGVFGSGFPKIS
SSHNASSSEDKYAKSGWNLNNFPRLPGSLLKYEGSDISGVLVPWLYIGMCFSSFCWHVEDHHLYSLNYMHWGAPKLWYGVGGKDAVKLEEAMRKHLPDLFEEQPDLLHKLVTQLSPSKLKTAGVPVHRCVQHAGEFVLTFPRAYHAGFNSGFNCAEAVNVAPVDWLPHGQIAIEL
YCQQGRKTSISHDKLLLGAAREVVKADWELNLLRKNTVDNLRWKAFSAKDGILAKTLKARIDMERTRREFLCNSSLALKMHSNFDATNERECCICFFDLHLSAAGCRCSPEKYSCLTHVKELCSCPWVTKYFLFRYDIDELNVLVEAVEGKLSSVYRWARQDLGLALSTDVSGSK
MEIDEEGKVHKDPTPQTTALSGKDLQLKVTSKEVSKELEKTSKLSHVNLLLKEKEEQITSSHCMKPVKEETVCDSSDPNVSACQPSEGGIICMTAVKSASGKKNSQSLPNDVILLSDDEYDIPRKRGSVRRDAISSGKKLEIRERPTHVLALEASAKIAAPICQREGDSLRDTRN
TISLPTNDQKTMRRDVPSSTSHAEVNAEATGLTQDICNRMATNSHGGGKPTSCKSKNSGGLAIVDVVDGTRSSSGTPSCSQNNSPDRFIRQKGPRIAKVVRRINCNVEPLSYGCVLSGKSWCSRRAIFPKGFRSRVKYINILDPTNMCFYISEILDAGRNSPLFMVYLESNPSEV
FVHMSPTRCWEMVRERVNQEITKQHKAGKSDLPPLQPSGSPDGFEMFGYSSPAIVQAIEALDVNRVCTDYWDSRPYSRPQVQFPANPLLREANTSGRSNVGNLQLNPGHHISPTGINSILKVLFKKASMEELSSLQEVLSETNSDMVTELVKEEIQNRR
0000000000000000000000000000000000000000000000000000000000000000000000000000000000000000000000000000000000000000000000000000000000000000000000000000000000000000000000000000000
0000000000000000000000000000000000000000000000000000000000000000000000000000000000000000000000000000000000000000000000000000000000000000000000000000000000000000000000000000000
0000000000000000000000000000000000000000000000000000000000000000000000000000000000000000000000000000000000000000000000000000000000000000000000000000000000000000000000000000000
0000000000000000000000000000000000000000000000000000000000000000000000000000000000000000000000000000000000000000000000000000000000000000000000000000000000000000000000000000000
0000000000000000000000000000000000000000000000000000000000000000000000000000000000000000000000000000000000000000000000000000000000000000000000000000000000000000000000000000000
0000000000000000000000000000000000000000000000000000000000000000000000000000000000000000000000000000000000000000000000000000000000000000000000000000000000000000000000000000000
000000000000000000000000000000000000000000000000000000000000000000000000000000000000000000000000000000000000000000000000000000000000000000000000000000000000000
0000000000000000000000000000000000000000000000000000000000000000000000000000000000000000000000000000000000000000000000000000000000000000000000000000000000000000000000000000000
0000000000000000000000000000000000000000000000000000000000000000000000000000000000000000000000000000000000000000000000000000000000000000000000000000000000000000000000000000000
0000000000000000000000000000000000000000000000000000000000000000000000000000000000000000000000000000000000000000000000000000000000000000000000000000000000000000000000000000000
0000000000000000000000000000000000000000000000000000000000000000000000000000000000000000000000000000000000000000000000000000000000000000000000000000000000000000000000000000000
0000000000000000000000000000000000000000000000000000000000000000000000000000000000000000000000000000000000000000000000000000000000000000000000000000000000000000000000000000000
0000000000000000000000000000000000000000000000000000000000000000000000000000000000000000000000000000000000000000000000000000000000000000000000000000000000000000000000000000000
000000000000000000000000000000000000000000000000000000000000000000000000000000000000000000000000000000000000000000000000000000000000000000000000000000000000000
0000000000000000000000000000000000000000000000000000000000000000000000000000000000000000000000000000000000000000000000000000000000000000000000000000000000000000000000000000000
0000000000000000000000000000000000000000000000000000000000000000000000000000000000000000000000000000000000000000000000000000000000000000000000000000000000000000000000000000000
0000000000000000000000000000000000000000000000000000000000000000000000000000000000000000000000000000000000000000000000000000000000000000000000000000000000000000000000000000000
0000000000000000000000000000000000000000000000000000000000000000000000000000000000000000000000000000000000000000000000000000000000000000000000000000000000000000000000000000000
0000000000000000000000000000000000000000000000000000000000000000000000000000000000000000000000000000000000000000000000000000000000000000000000000000000000000000000000000000000
0000000000000000000000000000000000000000000000000000000000000000000000000000000000000000000000000000000000000000000000000000000000000000000000000000000000000000000000000000000
000000000000000000000000000000000000000000000000000000000000000000000000000000000000000000000000000000000000000000000000000000000000000000000000000000000000000
0000000000000000000000000000000000000000000000000000000000000000000000000000000000000000000000000000000000000000000000000000000000000000000000000000000000000000000000000000000
0000000000000000000000000000000000000000000000000000000000000000000000000000000000000000000000000000000000000000000000000000000000000000000000000000000000000000000000000000000
0000000000000000000000000000000000000000000000000000000000000000000000000000000000000000000000000000000000000000000000000000000000000000000000000000000000000000000000000000000
0000000000000000000000000000000000000000000000000000000000000000000000000000000000000000000000000000000000000000000000000000000000000000000000000000000000000000000000000000000
0000000000000000000000000000000000000000000000000000000000000000000000000000000000000000000000000000000000000000000000000000000000000000000000000000000000000000000000000000000
0000000000000000000000000000000000000000000000000000000000000000000000000000000000000000000000000000000000000000000000000000000000000000000000000000000000000000000000000000000
000000000000000000000000000000000000000000000000000000000000000000000000000000000000000000000000000000000000000000000000000000000000000000000000000000000000000
0000000000000000000000000000000000000000000000000000000000000000000000000000000000000000000000000000000000000000000000000000000000000000000000000000000000000000000000000000000
0000000000000000000000000000000000000000000000000000000000000000000000000000000000000000000000000000000000000000000000000000000000000000000000000000000000000000000000000000000
0000000000000000000000000000000000000000000000000000000000000000000000000000000000000000000000000000000000000000000000000000000000000000000000000000000000000000000000000000000
0000000000000000000000000000000000000000000000000000000000000000000000000000000000000000000000000000000000000000000000000000000000000000000000000000000000000000000000000000000
0000000000000000000000000000000000000000000000000000000000000000000000000000000000000000000000000000000000000000000000000000000000000000000000000000000000000000000000000000000
0000000000000000000000000000000000000000000000000000000000000000000000000000000000000000000000000000000000000000000000000000000000000000000000000000000000000000000000000000000
000000000000000000000000000000000000000000000000000000000000000000000000000000000000000000000000000000000000000000000000000000000000000000000000000000000000000
0000000000000000000000000000000000000000000000000000000000000000000000000000000000000000000000000000000000000000000000000000000000000000000000000000000000000000000000000000000
0000000000000000000000000000000000000000000000000000000000000000000000000000000000000000000000000000000000000000000000000000000000000000000000000000000000000000000000000000000
0000000000000000000000000000000000000000000000000000000000000000000000000000000000000000000000000000000000000000000000000000000000000000000000000000000000000000000000000000000
0000000000000000000000000000000000000000000000000000000000000000000000000000000000000000000000000000000000000000000000000000000000000000000000000000000000000000000000000000000
0000000000000000000000000000000000000000000000000000000000000000000000000000000000000000000000000000000000000000000000000000000000000000000000000000000000000000000000000000000
0000000000000000000000000000000000000000000000000000000000000000000000000000000000000000000000000000000000000000000000000000000000000000000000000000000000000000000000000000000
000000000000000000000000000000000000000000000000000000000000000000000000000000000000000000000000000000000000000000000000000000000000000000000000000000000000000

0000000000000000000000000000000000000000000000000000000000000000000000000000000000000000000000000000000000000000000000000000000000000000000000000000000000000000000000000000000
0000000000000000000000000000000000000000000000000000000000000000000000000000000000000000000000000000000000000000000000000000000000000000000000000000000000000000000000000000000
0000000000000000000000000000000000000000000000000000000000000000000000000000000000000000000000000000000000000000000000000000000000000000000000000000000000000000000000000000000
0000000000000000000000000000000000000000000000000000000000000000000000000000000000000000000000000000000000000000000000000000000000000000000000000000000000000000000000000000000
0000000000000000000000000000000000000000000000000000000000000000000000000000000000000000000000000000000000000000000000000000000000000000000000000000000000000000000000000000000
0000000000000000000000000000000000000000000000000000000000000000000000000000000000000000000000000000000000000000000000000000000000000000000000000000000000000000000000000000000
000000000000000000000000000000000000000000000000000000000000000000000000000000000000000000000000000000000000000000000000000000000000000000000000000000000000000
>C1D141
MTRPRVFIDGEAGTTGLQIRARLSGRTDLELLSIDPARRKDPQARAELLNAANVSILCLHDDAAREAVALTTNPEARLLDASTAHRVHPEWVFGFPELSAQQPDRIRAARYVANPGCYSTGAIALLAPLTAAGLLSADFPVSIQGYSGYTGGGRALVDAHEKGEAHPMRGPFLSY
ALGLGHKHIPETMRYGGLSRTPIFTPNVGAWAQGMTVTIPLHLSDLGVSAQALHGALTAHYAGQKYVRVAAMESNPEILDPQTLNGTNDLDLFVYASQDGERAVLAARLDNLGKGAGGAAVQNLNLMLGLPDA
0000000000000000000000000000000000000000000000000000000000000000000000000000000000000000000000000000000000000000000000000000000000000000000000000000000000000000000000000000000
000000000000000000000000000000000000000000000000000000000000000000000000000000000000000000000000000000000000000000000000000000000000
0000000000000000000000000000000000000000000000000000000000000000000000000000000000000000000000000000000000000000000000000000000000000000000000000000000000000000000000000000000
000000000000000000000000000000000000000000000000000000000000000000000000000000000000000000000000000000000000000000000000000000000000
0000000000000000000000000000000000000000000000000000000000000000000000000000000000000000000000000000000000000000000000000000000000000000000000000000000000000000000000000000000
000000000000000000000000000000000000000000000000000000000000000000000000000000000000000000000000000000000000000000000000000000000000
0000000000000000000000000000000000000000000000000000000000000000000000000000000000000000000000000000000000000000000000000000000000000000000000000000000000000000000000000000000
000000000000000000000000000000000000000000000000000000000000000000000000000000000000000000000000000000000000000000000000000000000000
0000000000000000000000000000000000000000000000000000000000000000000000000000000000000000000000000000000000000000000000000000000000000000000000000000000000000000000000000000000
000000000000000000000000000000000000000000000000000000000000000000000000000000000000000000000000000000000000000000000000000000000000
0000000000000000000000000000000000000000000000000000000000000000000000000000000000000000000000000000000000000000000000000000000000000000000000000000000000000000000000000000000
000000000000000000000000000000000000000000000000000000000000000000000000000000000000000000000000000000000000000000000000000000000000
0000000000000000000000000000000000000000000000000000000000000000000000000000000000000000000000000000000000000000000000000000000000000000000000000000000000000000000000000000000
000000000000000000000000000000000000000000000000000000000000000000000000000000000000000000000000000000000000000000000000000000000000
>F5HEZ4
MATYEVLCEVARKLGTDDREVVLFLLNVFIPQPTLAQLIGALRALKEEGRLTFPLLAECLFRAGRRDLLRDLLHLDPRFLERHLAGTMSYFSPYQLTVLHVDGELCARDIRSLIFLSKDTIGSRSTPQTFLHWVYCMENLDLLGPTDVDALMSMLRSLSRVDLQRQVQTLMGLHL
SGPSHSQHYRHTP
0000000000000000000000000000000000000000000000000000000000000000000000000000000000000000000000000000000000000000000000000000000000000000000000000000000000000000000000000000000
0000000000000
0000000000000000000000000000000000000000000000000000000000000000000000000000000000000000000000000000000000000000000000000000000000000000000000000000000000000000000000000000000
0000000000000
0000000000000000000000000000000000000000000000000000000000000000000000000000000000000000000000000000000000000000000000000000000000000000000000000000000000000000000000000000000
0000000000000
0000000000000000000000000000000000000000000000000000000000000000000000000000000000000000000000000000000000000000000000000000000000000000000000000000000000000000000000000000000
0000000000000
0000000000000000000000000000000000000000000000000000000000000000000000000000000000000000000000000000000000000000000000000000000000000000000000000000000000000000000000000000000
0000000000000
0000000000000000000000000000000000000000000000000000000000000000000000000000000000000000000000000000000000000000000000000000000000000000000000000000000000000000000000000000000
0000000000000
0000000000000000000000000000000000000000000000000000000000000000000000000000000000000000000000000000000000000000000000000000000000000000000000000000000000000000000000000000000
0000000000000
>O14511
MRQVCCSALPPPPLEKGRCSSYSDSSSSSSERSSSSSSSSSESGSSSRSSSNNSSISRPAAPPEPRPQQQPQPRSPAARRAAARSRAAAAGGMRRDPAPGFSMLLFGVSLACYSPSLKSVQDQAYKAPVVVEGKVQGLVPAGGSSSNSTREPPASGRVALVKVLDKWPLRSGGLQ
REQVISVGSCVPLERNQRYIFFLEPTEQPLVFKTAFAPLDTNGKNLKKEVGKILCTDCATRPKLKKMKSQTGQVGEKQSLKCEAAAGNPQPSYRWFKDGKELNRSRDIRIKYGNGRKNSRLQFNKVKVEDAGEYVCEAENILGKDTVRGRLYVNSVSTTLSSWSGHARKCNETAK
SYCVNGGVCYYIEGINQLSCKCPNGFFGQRCLEKLPLRLYMPDPKQKAEELYQKRVLTITGICVALLVVGIVCVVAYCKTKKQRKQMHNHLRQNMCPAHQNRSLANGPSHPRLDPEEIQMADYISKNVPATDHVIRRETETTFSGSHSCSPSHHCSTATPTSSHRHESHTWSLER
SESLTSDSQSGIMLSSVGTSKCNSPACVEARARRAAAYNLEERRRATAPPYHDSVDSLRDSPHSERYVSALTTPARLSPVDFHYSLATQVPTFEITSPNSAHAVSLPPAAPISYRLAEQQPLLRHPAPPGPGPGPGPGPGPGADMQRSYDSYYYPAAGPGPRRGTCALGGSLGSL
PASPFRIPEDDEYETTQECAPPPPPRPRARGASRRTSAGPRRWRRSRLNGLAAQRARAARDSLSLSSGSGGGSASASDDDADDADGALAAESTPFLGLRGAHDALRSDSPPLCPAADSRTYYSLDSHSTRASSRHSRGPPPRAKQDSAPL
0000000000000000000000000000000000000000000000000000000000000000000000000000000000000000000000000000000000000000000000000000000000000000000000000000000000000000000000000000000
0000000000000000000000000000000000000000000000000000000000000000000000000000000000000000000000000000000000000000000000000000000000000000000000000000000000000000000000000000000
0000000000000000000000000000000000000000000000000000000000000000000000000000000000000000000000000000000000000000000000000000000000000000000000000000000000000000000000000000000
0000000000000000000000000000000000000000000000000000000000000000000000000000000000000000000000000000000000000000000000000000000000000000000000000000000000000000000000000000000
000000000000000000000000000000000000000000000000000000000000000000000000000000000000000000000000000000000000000000000000000000000000000000000000000000
0000000000000000000000000000000000000000000000000000000000000000000000000000000000000000000000000000000000000000000000000000000000000000000000000000000000000000000000000000000
0000000000000000000000000000000000000000000000000000000000000000000000000000000000000000000000000000000000000000000000000000000000000000000000000000000000000000000000000000000
0000000000000000000000000000000000000000000000000000000000000000000000000000000000000000000000000000000000000000000000000000000000000000000000000000000000000000000000000000000
0000000000000000000000000000000000000000000000000000000000000000000000000000000000000000000000000000000000000000000000000000000000000000000000000000000000000000000000000000000
000000000000000000000000000000000000000000000000000000000000000000000000000000000000000000000000000000000000000000000000000000000000000000000000000000
0000000000000000000000000000000000000000000000000000000000000000000000000000000000000000000000000000000000000000000000000000000000000000000000000000000000000000000000000000000
0000000000000000000000000000000000000000000000000000000000000000000000000000000000000000000000000000000000000000000000000000000000000000000000000000000000000000000000000000000
0000000000000000000000000000000000000000000000000000000000000000000000000000000000000000000000000000000000000000000000000000000000000000000000000000000000000000000000000000000
0000000000000000000000000000000000000000000000000000000000000000000000000000000000000000000000000000000000000000000000000000000000000000000000000000000000000000000000000000000
000000000000000000000000000000000000000000000000000000000000000000000000000000000000000000000000000000000000000000000000000000000000000000000000000000
0000000000000000000000000000000000000000000000000000000000000000000000000000000000000000000000000000000000000000000000000000000000000000000000000000000000000000000000000000000
0000000000000000000000000000000000000000000000000000000000000000000000000000000000000000000000000000000000000000000000000000000000000000000000000000000000000000000000000000000
0000000000000000000000000000000000000000000000000000000000000000000000000000000000000000000000000000000000000000000000000000000000000000000000000000000000000000000000000000000
0000000000000000000000000000000000000000000000000000000000000000000000000000000000000000000000000000000000000000000000000000000000000000000000000000000000000000000000000000000
000000000000000000000000000000000000000000000000000000000000000000000000000000000000000000000000000000000000000000000000000000000000000000000000000000
0000000000000000000000000000000000000000000000000000000000000000000000000000000000000000000000000000000000000000000000000000000000000000000000000000000000000000000000000000000
0000000000000000000000000000000000000000000000000000000000000000000000000000000000000000000000000000000000000000000000000000000000000000000000000000000000000000000000000000000

0000000000000000000000000000000000000000000000000000000000000000000000000000000000000000000000000000000000000000000000000000000000000000000000000000000000000000000000000000000
0000000000000000000000000000000000000000000000000000000000000000000000000000000000000000000000000000000000000000000000000000000000000000000000000000000000000000000000000000000
000000000000000000000000000000000000000000000000000000000000000000000000000000000000000000000000000000000000000000000000000000000000000000000000000000
0000000000000000000000000000000000000000000000000000000000000000000000000000000000000000000000000000000000000000000000000000000000000000000000000000000000000000000000000000000
0000000000000000000000000000000000000000000000000000000000000000000000000000000000000000000000000000000000000000000000000000000000000000000000000000000000000000000000000000000
0000000000000000000000000000000000000000000000000000000000000000000000000000000000000000000000000000000000000000000000000000000000000000000000000000000000000000000000000000000
0000000000000000000000000000000000000000000000000000000000000000000000000000000000000000000000000000000000000000000000000000000000000000000000000000000000000000000000000000000
000000000000000000000000000000000000000000000000000000000000000000000000000000000000000000000000000000000000000000000000000000000000000000000000000000
0000000000000000000000000000000000000000000000000000000000000000000000000000000000000000000000000000000000000000000000000000000000000000000000000000000000000000000000000000000
0000000000000000000000000000000000000000000000000000000000000000000000000000000000000000000000000000000000000000000000000000000000000000000000000000000000000000000000000000000
0000000000000000000000000000000000000000000000000000000000000000000000000000000000000000000000000000000000000000000000000000000000000000000000000000000000000000000000000000000
0000000000000000000000000000000000000000000000000000000000000000000000000000000000000000000000000000000000000000000000000000000000000000000000000000000000000000000000000000000
000000000000000000000000000000000000000000000000000000000000000000000000000000000000000000000000000000000000000000000000000000000000000000000000000000
>O23685
MIHFVLLVSRQGKVRLTKWYSPYAQKERSKVIRELSGVILNRGPKLCNFVEWRGYKVVYKRYASLYFCMCIDQEDNELEVLEIIHHYVEILDRYFGSVCELDLIFNFHKAYYILDELLIAGELQESSKKTVARIISAQDQLVEVAKEEASSISNIIAQATNR
000000000000000000000000000000000000000000000000000000000000000000000000000000000000000000000000000000000000000000000000000000000000000000000000000000000000000000
000000000000000000000000000000000000000000000000000000000000000000000000000000000000000000000000000000000000000000000000000000000000000000000000000000000000000000
000000000000000000000000000000000000000000000000000000000000000000000000000000000000000000000000000000000000000000000000000000000000000000000000000000000000000000
000000000000000000000000000000000000000000000000000000000000000000000000000000000000000000000000000000000000000000000000000000000000000000000000000000000000000000
000000000000000000000000000000000000000000000000000000000000000000000000000000000000000000000000000000000000000000000000000000000000000000000000000000000000000000
000000000000000000000000000000000000000000000000000000000000000000000000000000000000000000000000000000000000000000000000000000000000000000000000000000000000000000
000000000000000000000000000000000000000000000000000000000000000000000000000000000000000000000000000000000000000000000000000000000000000000000000000000000000000000
>O29518
MVVTAPVLPTLKTRTAPLSTLLSLTNFSTSGVMSAMSPKPSSSTSISLIIILPPYEVKLRTFIVYNSRLHVVPVEGLDEAVRTNFPRNLDYCGKKLRWWAEGVDERGPFIYFEVGLCHVVNVKSATLRSSPLNNIRCENLKAPPRQKLGYRSKSPYSHLHDLTSVVFCYVVGNLQ
HEQFLYLRGAIKFCADGDNQGLHAQGL
0000000000000000000000000000000000000000000000000000000000000000000000000000000000000000000000000000000000000000000000000000000000000000000000000000000000000000000000000000000
000000000000000000000000000
0000000000000000000000000000000000000000000000000000000000000000000000000000000000000000000000000000000000000000000000000000000000000000000000000000000000000000000000000000000
000000000000000000000000000
0000000000000000000000000000000000000000000000000000000000000000000000000000000000000000000000000000000000000000000000000000000000000000000000000000000000000000000000000000000
000000000000000000000000000
0000000000000000000000000000000000000000000000000000000000000000000000000000000000000000000000000000000000000000000000000000000000000000000000000000000000000000000000000000000
000000000000000000000000000
0000000000000000000000000000000000000000000000000000000000000000000000000000000000000000000000000000000000000000000000000000000000000000000000000000000000000000000000000000000
000000000000000000000000000
0000000000000000000000000000000000000000000000000000000000000000000000000000000000000000000000000000000000000000000000000000000000000000000000000000000000000000000000000000000
000000000000000000000000000
0000000000000000000000000000000000000000000000000000000000000000000000000000000000000000000000000000000000000000000000000000000000000000000000000000000000000000000000000000000
000000000000000000000000000
>O34391
MSIPVKKNLVSEAKYALKCPNAMSAEYITIHNTANDASAANEISYMIGNTSSTSFHFAVDDQEVIQGLPLNRNAWHTGDGTNGPGNRKSIGVEICYSKSGGPKYEAAEALAISFVAQLLKERGWGIDRVRKHQDWSGKYCPHRILSEGRWDQVKAAIEKELNGGVSAKKAAVSSS
ASEYHVKKGDTLSGIAASHGASVKTLQSINHITDPNHIKIGQVIKLPQTASASKSHAASSYPLPSGVIKVTSPLTQGTKVKQVQTALAALYFYPDKGAKNHGVDGVYGPKTANAVKRFQSVSGLTADGIYGPKTKAKMEEKL
0000000000000000000000000000000000000000000000000000000000000000000000000000000000000000000000000000000000000000000000000000000000000000000000000000000000000000000000000000000
0000000000000000000000000000000000000000000000000000000000000000000000000000000000000000000000000000000000000000000000000000000000000000
0000000000000000000000000000000000000000000000000000000000000000000000000000000000000000000000000000000000000000000000000000000000000000000000000000000000000000000000000000000
0000000000000000000000000000000000000000000000000000000000000000000000000000000000000000000000000000000000000000000000000000000000000000
0000000000000000000000000000000000000000000000000000000000000000000000000000000000000000000000000000000000000000000000000000000000000000000000000000000000000000000000000000000
0000000000000000000000000000000000000000000000000000000000000000000000000000000000000000000000000000000000000000000000000000000000000000
0000000000000000000000000000000000000000000000000000000000000000000000000000000000000000000000000000000000000000000000000000000000000000000000000000000000000000000000000000000
0000000000000000000000000000000000000000000000000000000000000000000000000000000000000000000000000000000000000000000000000000000000000000
0000000000000000000000000000000000000000000000000000000000000000000000000000000000000000000000000000000000000000000000000000000000000000000000000000000000000000000000000000000
0000000000000000000000000000000000000000000000000000000000000000000000000000000000000000000000000000000000000000000000000000000000000000
0000000000000000000000000000000000000000000000000000000000000000000000000000000000000000000000000000000000000000000000000000000000000000000000000000000000000000000000000000000
0000000000000000000000000000000000000000000000000000000000000000000000000000000000000000000000000000000000000000000000000000000000000000
0000000000000000000000000000000000000000000000000000000000000000000000000000000000000000000000000000000000000000000000000000000000000000000000000000000000000000000000000000000
0000000000000000000000000000000000000000000000000000000000000000000000000000000000000000000000000000000000000000000000000000000000000000
>O77760
MVGEEKMSLRNRLSKSRENPEEDEDQRKPAKESLEAPSNGRIDIKQLIAKKIKLTAEAEELKPFFMKEVGSHFDDFVTNLIEKSASLDNGGCALTTFSILEGEKNNHRAKDLRAPPEQGKIFIARRSLLDELLEVDHIRTIYHMFIALLILFILSTLVVDYIDEGRLVLEFSLLS
YAFGKFPTVVWTWWIMFLSTFSVPYFLFQRWATGYSKSSHPLINSLFHGFLFMVFQIGILGFGPTYVVLAYTLPPASRFIIIFEQIRFVMKAHSFVRENVPRVLNSAKEKSSTVPIPTVNQYLYFLFAPTLIYRDSYPRNPTVRWGYVAMQFAQVFGCFFYVYYIFERLCAPLFR
NIKQEPFSARVLVLCVFNSILPGVLILFLTFFAFLHCWLNAFAEMLRFGDRMFYKDWWNSTSYSNYYRTWNVVVHDWLYYYAYKDFLWFFSKRFKSAAMLAVFAVSAVVHEYALAVCLSFFYPVLFVLFMFFGMAFNFIVNDSRKKPIWNVMMWTSLFLGNGVLLCFYSQEWYAR
QHCPLKNPTFLDYVRPRSWTCRYVF
0000000000000000000000000000000000000000000000000000000000000000000000000000000000000000000000000000000000000000000000000000000000000000000000000000000000000000000000000000000
0000000000000000000000000000000000000000000000000000000000000000000000000000000000000000000000000000000000000000000000000000000000000000000000000000000000000000000000000000000
0000000000000000000000000000000000000000000000000000000000000000000000000000000000000000000000000000000000000000000000000000000000000000000000000000000000000000000000000000000
0000000000000000000000000
0000000000000000000000000000000000000000000000000000000000000000000000000000000000000000000000000000000000000000000000000000000000000000000000000000000000000000000000000000000
0000000000000000000000000000000000000000000000000000000000000000000000000000000000000000000000000000000000000000000000000000000000000000000000000000000000000000000000000000000
0000000000000000000000000000000000000000000000000000000000000000000000000000000000000000000000000000000000000000000000000000000000000000000000000000000000000000000000000000000
0000000000000000000000000

00000000000000000000000000000000000000000000000000000000000000000000000000000000000000000000000000000000000000000000000000000000000000000000000000000000000000000000000000000
00000000000000000000000000000000000000000000000000000000000000000000000000000000000000000000000000000000000000000000000000000000000000000000000000000000000000000000000000000
00000000000000000000000000000000000000000000000000000000000000000000000000000000000000000000000000000000000000000000000000000000000000000000000000000000000000000000000000000
0000000000000000000000000
00000000000000000000000000000000000000000000000000000000000000000000000000000000000000000000000000000000000000000000000000000000000000000000000000000000000000000000000000000
00000000000000000000000000000000000000000000000000000000000000000000000000000000000000000000000000000000000000000000000000000000000000000000000000000000000000000000000000000
00000000000000000000000000000000000000000000000000000000000000000000000000000000000000000000000000000000000000000000000000000000000000000000000000000000000000000000000000000
0000000000000000000000000
00000000000000000000000000000000000000000000000000000000000000000000000000000000000000000000000000000000000000000000000000000000000000000000000000000000000000000000000000000
00000000000000000000000000000000000000000000000000000000000000000000000000000000000000000000000000000000000000000000000000000000000000000000000000000000000000000000000000000
00000000000000000000000000000000000000000000000000000000000000000000000000000000000000000000000000000000000000000000000000000000000000000000000000000000000000000000000000000
0000000000000000000000000
00000000000000000000000000000000000000000000000000000000000000000000000000000000000000000000000000000000000000000000000000000000000000000000000000000000000000000000000000000
00000000000000000000000000000000000000000000000000000000000000000000000000000000000000000000000000000000000000000000000000000000000000000000000000000000000000000000000000000
00000000000000000000000000000000000000000000000000000000000000000000000000000000000000000000000000000000000000000000000000000000000000000000000000000000000000000000000000000
0000000000000000000000000
00000000000000000000000000000000000000000000000000000000000000000000000000000000000000000000000000000000000000000000000000000000000000000000000000000000000000000000000000000
00000000000000000000000000000000000000000000000000000000000000000000000000000000000000000000000000000000000000000000000000000000000000000000000000000000000000000000000000000
00000000000000000000000000000000000000000000000000000000000000000000000000000000000000000000000000000000000000000000000000000000000000000000000000000000000000000000000000000
0000000000000000000000000
>O94707
MEEKTPENEQSKKTFDPKDSMKIEETSTNGSSQPSQPSNIKLSIGSILESSNDNGDPEYSENGMGNMNMNTLPMATSTPMSYTKQPSEAKYPNSVWERKGVSDQEENTSSVKRQKTLPTQSSGEEEAKYSHPGAPTATSADSISMESRPSNLSTSLSKTTSYPQFQVRQFVSPII
SIDNSALEPFLNRYPASESLFPVTEYEYTPWLEFPLLYSSIGKFVRVTIDIKWLNAAINPRLCRREIWGTDVYTDDSDIATILAHCGCFSLLKPVRKIAVVDLYILPPLVHYKGTRKNQIESRSWSSRQDGISLKIKEVTWKPACASIFENSIHTLTLEERLQARLELSRSSTFK
I
00000000000000000000000000000000000000000000000000000000000000000000000000000000000000000000000000000000000000000000000000000000000000000000000000000000000000000000000000000
00000000000000000000000000000000000000000000000000000000000000000000000000000000000000000000000000000000000000000000000000000000000000000000000000000000000000000000000000000
0
00000000000000000000000000000000000000000000000000000000000000000000000000000000000000000000000000000000000000000000000000000000000000000000000000000000000000000000000000000
00000000000000000000000000000000000000000000000000000000000000000000000000000000000000000000000000000000000000000000000000000000000000000000000000000000000000000000000000000
0
00000000000000000000000000000000000000000000000000000000000000000000000000000000000000000000000000000000000000000000000000000000000000000000000000000000000000000000000000000
00000000000000000000000000000000000000000000000000000000000000000000000000000000000000000000000000000000000000000000000000000000000000000000000000000000000000000000000000000
0
00000000000000000000000000000000000000000000000000000000000000000000000000000000000000000000000000000000000000000000000000000000000000000000000000000000000000000000000000000
00000000000000000000000000000000000000000000000000000000000000000000000000000000000000000000000000000000000000000000000000000000000000000000000000000000000000000000000000000
0
00000000000000000000000000000000000000000000000000000000000000000000000000000000000000000000000000000000000000000000000000000000000000000000000000000000000000000000000000000
00000000000000000000000000000000000000000000000000000000000000000000000000000000000000000000000000000000000000000000000000000000000000000000000000000000000000000000000000000
0
00000000000000000000000000000000000000000000000000000000000000000000000000000000000000000000000000000000000000000000000000000000000000000000000000000000000000000000000000000
00000000000000000000000000000000000000000000000000000000000000000000000000000000000000000000000000000000000000000000000000000000000000000000000000000000000000000000000000000
0
00000000000000000000000000000000000000000000000000000000000000000000000000000000000000000000000000000000000000000000000000000000000000000000000000000000000000000000000000000
00000000000000000000000000000000000000000000000000000000000000000000000000000000000000000000000000000000000000000000000000000000000000000000000000000000000000000000000000000
0
>P01045
MKLITILFLCSRLLPSLTQESSQEIDCNDQDVFKAVDAALTKYNSENKSGNQFVLYRITEVARMDNPDTFYSLKYQIKEGDCPFQSNKTWQDCDYKDSAQAATGQCTATVAKRGNMKFSVAIQTCLITPAEGPVVTAQYECLGCVHPISTKSPDLEPVLRYAIQYFNNNTSHSHL
FDLKEVKRAQKQVVSGWNYEVNYSIAQTNCSKEEFSFLTPDCKSLSSGDTGECTDKAHVDVKLRISSFSQKCDLYPGEDFLPPMVCVGCPKPIPVDSPDLEEALNHSIAKLNAEHDGTFYFKIDTVKKATVQVVGGLKYSIVFIARETTCSKGSNEELTKSCEINIHGQILHCDA
NVYVVPWEEKVYPTVNCQPLGQTSLMKRPPGFSPFRSVQVMKTEGSTTVSLPHSAMSPVQDEERDSGKEQGPTHGHGWDHGKQIKLHGLGLGHKHKHDQGHGHHRSHGLGHGHQKQHGLGHGHKHGHGHGKHKNKGKNNGKHYDWRTPYLASSYEDSTTSSAQTQEKTEETTLSS
LAQPGVAITFPDFQDSDLIATVMPNTLPPHTESDDDWIPDIQTEPNSLAFKLISDFPETTSPKCPSRPWKPVNGVNPTVEMKESHDFDLVDALL
00000000000000000000000000000000000000000000000000000000000000000000000000000000000000000000000000000000000000000000000000000000000000000000000000000000000000000000000000000
00000000000000000000000000000000000000000000000000000000000000000000000000000000000000000000000000000000000000000000000000000000000000000000000000000000000000000000000000000
00000000000000000000000000000000000000000000000000000000000000000000000000000000000000000000000000000000000000000000000000000000000000000000000000000000000000000000000000000
0000000000000000000000000000000000000000000000000000000000000000000000000000000000000000000000
00000000000000000000000000000000000000000000000000000000000000000000000000000000000000000000000000000000000000000000000000000000000000000000000000000000000000000000000000000
00000000000000000000000000000000000000000000000000000000000000000000000000000000000000000000000000000000000000000000000000000000000000000000000000000000000000000000000000000
00000000000000000000000000000000000000000000000000000000000000000000000000000000000000000000000000000000000000000000000000000000000000000000000000000000000000000000000000000
0000000000000000000000000000000000000000000000000000000000000000000000000000000000000000000000
00000000000000000000000000000000000000000000000000000000000000000000000000000000000000000000000000000000000000000000000000000000000000000000000000000000000000000000000000000
00000000000000000000000000000000000000000000000000000000000000000000000000000000000000000000000000000000000000000000000000000000000000000000000000000000000000000000000000000
00000000000000000000000000000000000000000000000000000000000000000000000000000000000000000000000000000000000000000000000000000000000000000000000000000000000000000000000000000
0000000000000000000000000000000000000000000000000000000000000000000000000000000000000000000000
00000000000000000000000000000000000000000000000000000000000000000000000000000000000000000000000000000000000000000000000000000000000000000000000000000000000000000000000000000
00000000000000000000000000000000000000000000000000000000000000000000000000000000000000000000000000000000000000000000000000000000000000000000000000000000000000000000000000000
00000000000000000000000000000000000000000000000000000000000000000000000000000000000000000000000000000000000000000000000000000000000000000000000000000000000000000000000000000
0000000000000000000000000000000000000000000000000000000000000000000000000000000000000000000000
00000000000000000000000000000000000000000000000000000000000000000000000000000000000000000000000000000000000000000000000000000000000000000000000000000000000000000000000000000
00000000000000000000000000000000000000000000000000000000000000000000000000000000000000000000000000000000000000000000000000000000000000000000000000000000000000000000000000000

0000000000000000000000000000000000000000000000000000000000000000000000000000000000000000000000000000000000000000000000000000000000000000000000000000000000000000000000000000000
0000000000000000000000000000000000000000000000000000000000000000000000000000000000000000000000
0000000000000000000000000000000000000000000000000000000000000000000000000000000000000000000000000000000000000000000000000000000000000000000000000000000000000000000000000000000
0000000000000000000000000000000000000000000000000000000000000000000000000000000000000000000000000000000000000000000000000000000000000000000000000000000000000000000000000000000
0000000000000000000000000000000000000000000000000000000000000000000000000000000000000000000000000000000000000000000000000000000000000000000000000000000000000000000000000000000
0000000000000000000000000000000000000000000000000000000000000000000000000000000000000000000000
0000000000000000000000000000000000000000000000000000000000000000000000000000000000000000000000000000000000000000000000000000000000000000000000000000000000000000000000000000000
0000000000000000000000000000000000000000000000000000000000000000000000000000000000000000000000000000000000000000000000000000000000000000000000000000000000000000000000000000000
0000000000000000000000000000000000000000000000000000000000000000000000000000000000000000000000000000000000000000000000000000000000000000000000000000000000000000000000000000000
0000000000000000000000000000000000000000000000000000000000000000000000000000000000000000000000
>P04744
MAHHEVISRSGNAFLLNIRESVLLPGSMSEMHFFLLIGISSIHSDRVILAMKDYLVGGHSRKEVCEKYQMNNGYFSTTLGRLIRLNALAARLAPYYTDESSAFD
00000000000000000000000000000000000000000000000000000000000000000000000000000000000000000000000000000000
00000000000000000000000000000000000000000000000000000000000000000000000000000000000000000000000000000000
00000000000000000000000000000000000000000000000000000000000000000000000000000000000000000000000000000000
00000000000000000000000000000000000000000000000000000000000000000000000000000000000000000000000000000000
00000000000000000000000000000000000000000000000000000000000000000000000000000000000000000000000000000000
00000000000000000000000000000000000000000000000000000000000000000000000000000000000000000000000000000000
00000000000000000000000000000000000000000000000000000000000000000000000000000000000000000000000000000000
>P0AAX4
MKTINTVVAAMALSTLSFGVFAAEPVTASQAQNMNKIGVVSADGASTLDALEAKLAEKAAAAGASGYSITSATNNNKLSGTAVIYK
00000000000000000000000000000000000000000000000000000000000000000000000000000000000000
00000000000000000000000000000000000000000000000000000000000000000000000000000000000000
00000000000000000000000000000000000000000000000000000000000000000000000000000000000000
00000000000000000000000000000000000000000000000000000000000000000000000000000000000000
00000000000000000000000000000000000000000000000000000000000000000000000000000000000000
00000000000000000000000000000000000000000000000000000000000000000000000000000000000000
00000000000000000000000000000000000000000000000000000000000000000000000000000000000000
>P13730
MKLTIATALVGILLIACAHVANGSDCGCPKRTTPKPCTTARPTCAPVTTTTCRPPTTTRCPPPTTTRCPPPTRPAECTATTKRPTARPTTKRATTRRTTVRATTKRATTRRTTKRATTRRTTVRATTKRATTRRTTTKRAPTRRATTKRATTRRNPTRRTTTRRAPTKRATTKRA
TTRRNPTKRKTTRRTTVRATKTTKRATTKRAPTKRATTKRAPTKRATTKRAPTKRATTKRAPTKRATTKRAPTKRATTKRAPTKRATTKRAPTKRATTKRATARPTSKPCGCKPCGPGGEPCQGCAKRDALCQDLNNILRNLERKVRQCVCGEPQWLL
0000000000000000000000000000000000000000000000000000000000000000000000000000000000000000000000000000000000000000000000000000000000000000000000000000000000000000000000000000000
00000000000000000000000000000000000000000000000000000000000000000000000000000000000000000000000000000000000000000000000000000000000000000000000000000000000000
0000000000000000000000000000000000000000000000000000000000000000000000000000000000000000000000000000000000000000000000000000000000000000000000000000000000000000000000000000000
00000000000000000000000000000000000000000000000000000000000000000000000000000000000000000000000000000000000000000000000000000000000000000000000000000000000000
0000000000000000000000000000000000000000000000000000000000000000000000000000000000000000000000000000000000000000000000000000000000000000000000000000000000000000000000000000000
00000000000000000000000000000000000000000000000000000000000000000000000000000000000000000000000000000000000000000000000000000000000000000000000000000000000000
0000000000000000000000000000000000000000000000000000000000000000000000000000000000000000000000000000000000000000000000000000000000000000000000000000000000000000000000000000000
00000000000000000000000000000000000000000000000000000000000000000000000000000000000000000000000000000000000000000000000000000000000000000000000000000000000000
0000000000000000000000000000000000000000000000000000000000000000000000000000000000000000000000000000000000000000000000000000000000000000000000000000000000000000000000000000000
00000000000000000000000000000000000000000000000000000000000000000000000000000000000000000000000000000000000000000000000000000000000000000000000000000000000000
0000000000000000000000000000000000000000000000000000000000000000000000000000000000000000000000000000000000000000000000000000000000000000000000000000000000000000000000000000000
00000000000000000000000000000000000000000000000000000000000000000000000000000000000000000000000000000000000000000000000000000000000000000000000000000000000000
0000000000000000000000000000000000000000000000000000000000000000000000000000000000000000000000000000000000000000000000000000000000000000000000000000000000000000000000000000000
00000000000000000000000000000000000000000000000000000000000000000000000000000000000000000000000000000000000000000000000000000000000000000000000000000000000000
>P15513
MQVYMLLPLAVFASLTYQGACEETAAAQTSSDASTSSASSEHAENELSRAKRGSYRMMRLGRGLHMLRLGKRGGPVEPESEENLETLLNLLQGYYSDVPEYPSEFDDTDLAYPYEEYDAPAHPRYRRSTPPTDGVVAPDVLQKGSSEFEDFGDSQLDESDEGYYGYDPENYLYGD
FEDYLEPEEGGLGEEKRSLSMLRLGKRGLSMLRLGKREGEEGDEMDKKQDESLNDDFENDDIKRTLSMLRLGKRPMSMLRLGKRPMSMLRLGKRPMSMLRLGKRPMSMLRLGKRPMSMLRLGKRPMSMLRLGKRPMSMLRLGKRPMSMLRLGKRPMSMLRLGKRPMSMLRLGKRD
DDEKEKKSLNMSRLGKRSTQ
0000000000000000000000000000000000000000000000000000000000000000000000000000000000000000000000000000000000000000000000000000000000000000000000000000000000000000000000000000000
0000000000000000000000000000000000000000000000000000000000000000000000000000000000000000000000000000000000000000000000000000000000000000000000000000000000000000000000000000000
00000000000000000000
0000000000000000000000000000000000000000000000000000000000000000000000000000000000000000000000000000000000000000000000000000000000000000000000000000000000000000000000000000000
0000000000000000000000000000000000000000000000000000000000000000000000000000000000000000000000000000000000000000000000000000000000000000000000000000000000000000000000000000000
00000000000000000000
0000000000000000000000000000000000000000000000000000000000000000000000000000000000000000000000000000000000000000000000000000000000000000000000000000000000000000000000000000000
0000000000000000000000000000000000000000000000000000000000000000000000000000000000000000000000000000000000000000000000000000000000000000000000000000000000000000000000000000000
00000000000000000000
0000000000000000000000000000000000000000000000000000000000000000000000000000000000000000000000000000000000000000000000000000000000000000000000000000000000000000000000000000000
0000000000000000000000000000000000000000000000000000000000000000000000000000000000000000000000000000000000000000000000000000000000000000000000000000000000000000000000000000000
00000000000000000000
0000000000000000000000000000000000000000000000000000000000000000000000000000000000000000000000000000000000000000000000000000000000000000000000000000000000000000000000000000000
0000000000000000000000000000000000000000000000000000000000000000000000000000000000000000000000000000000000000000000000000000000000000000000000000000000000000000000000000000000
00000000000000000000
0000000000000000000000000000000000000000000000000000000000000000000000000000000000000000000000000000000000000000000000000000000000000000000000000000000000000000000000000000000
0000000000000000000000000000000000000000000000000000000000000000000000000000000000000000000000000000000000000000000000000000000000000000000000000000000000000000000000000000000
00000000000000000000

0000000000000000000000000000000000000000000000000000000000000000000000000000000000000000000000000000000000000000000000000000000000000000000000000000000000000000000000000000000
0000000000000000000000000000000000000000000000000000000000000000000000000000000000000000000000000000000000000000000000000000000000000000000000000000000000000000000000000000000
00000000000000000000
>P18490
MGSQTLEILRQGVWASLTGGYFYDPHQGVFCNVVHLYLWLYLLCSPFVAYLYFPSTWLTWCLYCVITSLTILMVKLANLALHRLYDRAQTMSEGNLKGQFVKVTKETEPRHDDEGGIEMKVIRPGGSRISGEQAINEASEENSIMSIDNVNSIIDLKVDVHRKNSSESIELMFYA
PSMLSGGSQQDQQSLAGSASVSKSIRSTGPGGNSSTSAAAQADMFNKYLTVYPEVVEAAGSSAGSADGGSSGGDSRSGAAVSTLGHTGAGTAPAQHVRTGHLSRKCSEVFSRRHRRRLERQSSLDTAAAAANSNAENKLMRNQSDTIATAAPSFLHSQPTNKARGQTNPRQHFIT
SAPSTGIGGGDAPTSSTAVVVIAPASNSLVNPGRSSRLQRHRSSETHDERLKHQSRGGLFQPILQLPHHSAASSIGALAVLGGGGNVAANDVEDMELGLVRNAGSGGVADACSRALQSWILDPFPDGYLEDDSYTKSDLGIEQPHQPTFRTQHRHHHHHHHLHHHLGGAIIRKDP
TSTMSALQLAAVTQPGTGGSVTGGGGAAGGGGSAAGMKRRRHSNATSYKHGSSQSNKSSLVGGGGSRGPSGEAGTSGGAGGTGGVRRIKSAALEVLCPQPSVSNLSPHPNSVEAISGQQQMRNPLPPPSKSLVRNQHLNLYPTQGYGDGTPSAGGGGPGNSSTCSSLFFGSSSAC
GSTTALIEPPVYPIVEHSDEKTAHEEHGDDCLGIGQGNADDDDEVDDVPIRRRTRALGCSQTHYSVESDVGGFLADEVGADEDVFKDFDDNLEHILSELQQTHNQLDDVLKTHDLHHHSHLHHHKAASVEGAGPSGGSVAVGVSAGNDDEDEETEDNNTGSRSPLLNDRNRQPAT
LREIQADKQLRQELSHQSGAVIPAPNPPVPIRSEADSGCPSSDCEQVSASSKDQLLSGIELELQQYQILQRCQLDEEQPCTSKMAAKSLGAIPKVVKYREVDELRRRRRGGSPADAAEAGNEFALVKQTKQASRNSSSSNSTHSISLTDSLTADIHKMLWLMHGGAVDDRGRPIT
GTSADGTPIPASSSLVPPNMSNAHFQFYQDAIQALQGTHPTSGSSVEHMTNIELARDKLRLDAKLMCEQLVAAAEANSGVRGNTLATQQMTQQQVGMLMRGGSSSRHPSSAPFSVQGLINVIRSERPAARSQLDALQQLSDAAAALAANASEAASVSASGAGGGASSGGGGVLSA
LGLANHPSNQISQISQLSQMSQPMLNVDGHFAPYCDYWRPACLLSATEKPAAPKSFYKYRFKWCGQEHEFKIAMDRLELLALFDRDLHWMHVLLASLLCTLVACLGAAILQHDHYKDLCALLFCAVIAGAQYSLVKSVQPDAASPVHGFNKTVAYSRAIYFCLAGGMLLLLKRLD
TDYGERTPDPVVFFGMRYSPADVVALLLQALYILLLCFPIIFSVGLCPQINTFLMYLLEQIDMHVFGGNAASSLLGSFLCVVRSVLAVMLLYGPLYGALDEQRGTQYILFSIFCAMLVPLGYHLSRCASDFSHLWRLIKTCIVSTYRDDEDEDLSQMQHIATGTVTGSGTANSTA
LQLTTSTPKQHRQTDVKTEHEQIELSSLEKLTVNEEHHEKDHGADDQLETDQDLPLQQKHSKSKTSSLGSSQQTLGKTISSSKRAITASSSCTSIGTGEPAVEAGALTEEAVEGEEQRRNTLAGEVGSKHELAENYDQAAKIDECEDKISSSSATNPGDMSTLTAGAGTATTDAT
PACLEADPDAEAEAPADEKQHQGILGTGTGTGTGTTEASENGSGGGANGNTNSNGTGNDLPDPLPRKLQATVTTRLKNDLVVMTLLAVSVLGLHCSTVFTALQPDLNVVLYSFIGVLGLLLHYIVPQMRKHMPWLCFSRPLLRQREFGQFEVLNAPKIMWFEKLYIYLSVLERNV
LFPLLAISSLTADSQLIVAKFGLPWGTLIVAICALKFVRNAYSDPTNQYLIIIFTVLLFRIDFAMATETFIIDYFFVSLAFRKCCDFLLKLQFIVTYIAPWQITWGSAFHAFAQPFSVPHSAMLFLQAAISAVLSTPLNPFLGSAIFLTSYVRPIKFWERDYNTRRIDHSNTRLS
SQLERDLGADDNNLNSIFYEHLTRSLQHSLCGDLLMGRWGNVNQGDCFVLASDDLNCLVHIIELGNGLCTFQMRGLEFRGTYCQQREVEAITEDVEDNDGCCCCDPGHLPQLLSANAMFSTRWLAWQVVAAQYVIEGYSISDNLASATLQVFEYRKVLITYYIKSIIYYVVKNPK
LEQWLASGPIQDALQHTLSRQFVDLDPIFNFNLDEDFDFRAVGITRSSFCYVYLKWINYCVDMRRDANSMGGAPPAQAPAAAGGASSAPATAGVAPPAPVTPAHNDSKSTPNLSAHGGQAGPSSGQSKSQSQQQLRRPQKSVAQETSVGGGGVVVGGTTVPGTGGVTGGGGDPQL
SSSHSFANISRQTSESAPGLGGYVAYMDQNVFVKLAKSSTTTGAGAGAPTSRKESQEKPALRLKLASVGKDAPLVSLCLALGLLARRSLATASHSALTGVEFFLHGLHALFKGDFRITSPRDEWVFADMELLHSVVAPAVKMALKLQQDHITNPDEFLDPHALYDAIDNCSNELV
ISHEADPVWRSAVLRGAPNLLALRHVMEDGSDEYRIIRLTKRCLSFRVIKLNRECVRGLWAGQQQELIYLRNRNPERGSIQNAKQALRNIINSSCDQPIGYPIYVSPLTTSYADTNAQLCEVIGGAITLDTIRHTVLGWWHRIRERCRQGCSSGSALEPHPGSGPVQLGACNFGS
GGSVVGAASTATGVGASTGSGLGVAAPGTAGSTGGESGDLAPVFISAPLYNTLTVNSYYSVRPGNVPGGPIPGNLGGNYVSDSLAVVRGGLAVMPVKPTSTTLIAGLLNRERDETSGSGIMGAGASGPTRATSSGGVRSRRPEVGSSRGRDHERRATLPIASGGAGGEPGKDLTA
PQTQVEHEPSPRSTKLSSSSGSLGLGMGVGLGNIITTPGDYPRKTKGPICLTAVDTGTSSSAAEGKPAGSGTVAGTGVGGVIRKPRIEIFKKVIIVDDTGIYDCLDIIDAVVWPTERMRNNGGRLSWKDWEPSAGMVGHVVHCWTPNHKDIRFRSHVNRYVYLVEIGDHYVPVEE
LGLREYNQIMGSTEEMANSRRSSIQRDFHEYNIQLKLAGLAPIIGGGESSTATTSDASKSHKAKIRAVSSSSSEDEDTSSTRSIPLPQGDDGDLTNFTRLVSMWKEIILDNNKRRLYDMGELSPELLQKLDDAVQQQQQLEDALAEESKEKGTATVTANEGEEGVGEMEIEPEQE
QKEVVYLEEQSPLEEVTVSKPDEQPAQPTIPASPVPAETSSTSSAKSTSSPSLCQEEEDAVDPEETPELASEESPSDENGESEAGTTV
0000000000000000000000000000000000000000000000000000000000000000000000000000000000000000000000000000000000000000000000000000000000000000000000000000000000000000000000000000000
0000000000000000000000000000000000000000000000000000000000000000000000000000000000000000000000000000000000000000000000000000000000000000000000000000000000000000000000000000000
0000000000000000000000000000000000000000000000000000000000000000000000000000000000000000000000000000000000000000000000000000000000000000000000000000000000000000000000000000000
0000000000000000000000000000000000000000000000000000000000000000000000000000000000000000000000000000000000000000000000000000000000000000000000000000000000000000000000000000000
0000000000000000000000000000000000000000000000000000000000000000000000000000000000000000000000000000000000000000000000000000000000000000000000000000000000000000000000000000000
0000000000000000000000000000000000000000000000000000000000000000000000000000000000000000000000000000000000000000000000000000000000000000000000000000000000000000000000000000000
0000000000000000000000000000000000000000000000000000000000000000000000000000000000000000000000000000000000000000000000000000000000000000000000000000000000000000000000000000000
0000000000000000000000000000000000000000000000000000000000000000000000000000000000000000000000000000000000000000000000000000000000000000000000000000000000000000000000000000000
0000000000000000000000000000000000000000000000000000000000000000000000000000000000000000000000000000000000000000000000000000000000000000000000000000000000000000000000000000000
0000000000000000000000000000000000000000000000000000000000000000000000000000000000000000000000000000000000000000000000000000000000000000000000000000000000000000000000000000000
0000000000000000000000000000000000000000000000000000000000000000000000000000000000000000000000000000000000000000000000000000000000000000000000000000000000000000000000000000000
0000000000000000000000000000000000000000000000000000000000000000000000000000000000000000000000000000000000000000000000000000000000000000000000000000000000000000000000000000000
0000000000000000000000000000000000000000000000000000000000000000000000000000000000000000000000000000000000000000000000000000000000000000000000000000000000000000000000000000000
0000000000000000000000000000000000000000000000000000000000000000000000000000000000000000000000000000000000000000000000000000000000000000000000000000000000000000000000000000000
0000000000000000000000000000000000000000000000000000000000000000000000000000000000000000000000000000000000000000000000000000000000000000000000000000000000000000000000000000000
0000000000000000000000000000000000000000000000000000000000000000000000000000000000000000000000000000000000000000000000000000000000000000000000000000000000000000000000000000000
0000000000000000000000000000000000000000000000000000000000000000000000000000000000000000000000000000000000000000000000000000000000000000000000000000000000000000000000000000000
0000000000000000000000000000000000000000000000000000000000000000000000000000000000000000000000000000000000000000000000000000000000000000000000000000000000000000000000000000000
0000000000000000000000000000000000000000000000000000000000000000000000000000000000000000000000000000000000000000000000000000000000000000000000000000000000000000000000000000000
0000000000000000000000000000000000000000000000000000000000000000000000000000000000000000
0000000000000000000000000000000000000000000000000000000000000000000000000000000000000000000000000000000000000000000000000000000000000000000000000000000000000000000000000000000
0000000000000000000000000000000000000000000000000000000000000000000000000000000000000000000000000000000000000000000000000000000000000000000000000000000000000000000000000000000
0000000000000000000000000000000000000000000000000000000000000000000000000000000000000000000000000000000000000000000000000000000000000000000000000000000000000000000000000000000
0000000000000000000000000000000000000000000000000000000000000000000000000000000000000000000000000000000000000000000000000000000000000000000000000000000000000000000000000000000
0000000000000000000000000000000000000000000000000000000000000000000000000000000000000000000000000000000000000000000000000000000000000000000000000000000000000000000000000000000
0000000000000000000000000000000000000000000000000000000000000000000000000000000000000000000000000000000000000000000000000000000000000000000000000000000000000000000000000000000
0000000000000000000000000000000000000000000000000000000000000000000000000000000000000000000000000000000000000000000000000000000000000000000000000000000000000000000000000000000
0000000000000000000000000000000000000000000000000000000000000000000000000000000000000000000000000000000000000000000000000000000000000000000000000000000000000000000000000000000
0000000000000000000000000000000000000000000000000000000000000000000000000000000000000000000000000000000000000000000000000000000000000000000000000000000000000000000000000000000
0000000000000000000000000000000000000000000000000000000000000000000000000000000000000000000000000000000000000000000000000000000000000000000000000000000000000000000000000000000
0000000000000000000000000000000000000000000000000000000000000000000000000000000000000000000000000000000000000000000000000000000000000000000000000000000000000000000000000000000
0000000000000000000000000000000000000000000000000000000000000000000000000000000000000000000000000000000000000000000000000000000000000000000000000000000000000000000000000000000
0000000000000000000000000000000000000000000000000000000000000000000000000000000000000000000000000000000000000000000000000000000000000000000000000000000000000000000000000000000
0000000000000000000000000000000000000000000000000000000000000000000000000000000000000000000000000000000000000000000000000000000000000000000000000000000000000000000000000000000
0000000000000000000000000000000000000000000000000000000000000000000000000000000000000000000000000000000000000000000000000000000000000000000000000000000000000000000000000000000
0000000000000000000000000000000000000000000000000000000000000000000000000000000000000000000000000000000000000000000000000000000000000000000000000000000000000000000000000000000
0000000000000000000000000000000000000000000000000000000000000000000000000000000000000000000000000000000000000000000000000000000000000000000000000000000000000000000000000000000
0000000000000000000000000000000000000000000000000000000000000000000000000000000000000000000000000000000000000000000000000000000000000000000000000000000000000000000000000000000
0000000000000000000000000000000000000000000000000000000000000000000000000000000000000000000000000000000000000000000000000000000000000000000000000000000000000000000000000000000
0000000000000000000000000000000000000000000000000000000000000000000000000000000000000000
0000000000000000000000000000000000000000000000000000000000000000000000000000000000000000000000000000000000000000000000000000000000000000000000000000000000000000000000000000000
0000000000000000000000000000000000000000000000000000000000000000000000000000000000000000000000000000000000000000000000000000000000000000000000000000000000000000000000000000000
0000000000000000000000000000000000000000000000000000000000000000000000000000000000000000000000000000000000000000000000000000000000000000000000000000000000000000000000000000000
0000000000000000000000000000000000000000000000000000000000000000000000000000000000000000000000000000000000000000000000000000000000000000000000000000000000000000000000000000000
0000000000000000000000000000000000000000000000000000000000000000000000000000000000000000000000000000000000000000000000000000000000000000000000000000000000000000000000000000000

00000000000000000000000000000000000000000000000000000000000000000000000000000000000000000000000000000000000000000000000000000000000000000000000000000000000000000000000000000000
00000000000000000000000000000000000000000000000000000000000000000000000000000000000000000000000000000000000000000000000000000000000000000000000000000000000000000000000000000000
00000000000000000000000000000000000000000000000000000000000000000000000000000000000000000000000000000000000000000000000000000000000000000000000000000000000000000000000000000000
00000000000000000000000000000000000000000000000000000000000000000000000000000000000000000000000000000000000000000000000000000000000000000000000000000000000000000000000000000000
00000000000000000000000000000000000000000000000000000000000000000000000000000000000000000000000000000000000000000000000000000000000000000000000000000000000000000000000000000000
00000000000000000000000000000000000000000000000000000000000000000000000000000000000000000000000000000000000000000000000000000000000000000000000000000000000000000000000000000000
00000000000000000000000000000000000000000000000000000000000000000000000000000000000000000000000000000000000000000000000000000000000000000000000000000000000000000000000000000000
00000000000000000000000000000000000000000000000000000000000000000000000000000000000000000000000000000000000000000000000000000000000000000000000000000000000000000000000000000000
00000000000000000000000000000000000000000000000000000000000000000000000000000000000000000000000000000000000000000000000000000000000000000000000000000000000000000000000000000000
00000000000000000000000000000000000000000000000000000000000000000000000000000000000000000000000000000000000000000000000000000000000000000000000000000000000000000000000000000000
00000000000000000000000000000000000000000000000000000000000000000000000000000000000000000000000000000000000000000000000000000000000000000000000000000000000000000000000000000000
00000000000000000000000000000000000000000000000000000000000000000000000000000000000000000000000000000000000000000000000000000000000000000000000000000000000000000000000000000000
00000000000000000000000000000000000000000000000000000000000000000000000000000000000000000000000000000000000000000000000000000000000000000000000000000000000000000000000000000000
00000000000000000000000000000000000000000000000000000000000000000000000000000000000000000000000000000000000000000000000000000000000000000000000000000000000000000000000000000000
0000000000000000000000000000000000000000000000000000000000000000000000000000000000000000
00000000000000000000000000000000000000000000000000000000000000000000000000000000000000000000000000000000000000000000000000000000000000000000000000000000000000000000000000000000
00000000000000000000000000000000000000000000000000000000000000000000000000000000000000000000000000000000000000000000000000000000000000000000000000000000000000000000000000000000
00000000000000000000000000000000000000000000000000000000000000000000000000000000000000000000000000000000000000000000000000000000000000000000000000000000000000000000000000000000
00000000000000000000000000000000000000000000000000000000000000000000000000000000000000000000000000000000000000000000000000000000000000000000000000000000000000000000000000000000
00000000000000000000000000000000000000000000000000000000000000000000000000000000000000000000000000000000000000000000000000000000000000000000000000000000000000000000000000000000
00000000000000000000000000000000000000000000000000000000000000000000000000000000000000000000000000000000000000000000000000000000000000000000000000000000000000000000000000000000
00000000000000000000000000000000000000000000000000000000000000000000000000000000000000000000000000000000000000000000000000000000000000000000000000000000000000000000000000000000
00000000000000000000000000000000000000000000000000000000000000000000000000000000000000000000000000000000000000000000000000000000000000000000000000000000000000000000000000000000
00000000000000000000000000000000000000000000000000000000000000000000000000000000000000000000000000000000000000000000000000000000000000000000000000000000000000000000000000000000
00000000000000000000000000000000000000000000000000000000000000000000000000000000000000000000000000000000000000000000000000000000000000000000000000000000000000000000000000000000
00000000000000000000000000000000000000000000000000000000000000000000000000000000000000000000000000000000000000000000000000000000000000000000000000000000000000000000000000000000
00000000000000000000000000000000000000000000000000000000000000000000000000000000000000000000000000000000000000000000000000000000000000000000000000000000000000000000000000000000
00000000000000000000000000000000000000000000000000000000000000000000000000000000000000000000000000000000000000000000000000000000000000000000000000000000000000000000000000000000
00000000000000000000000000000000000000000000000000000000000000000000000000000000000000000000000000000000000000000000000000000000000000000000000000000000000000000000000000000000
00000000000000000000000000000000000000000000000000000000000000000000000000000000000000000000000000000000000000000000000000000000000000000000000000000000000000000000000000000000
00000000000000000000000000000000000000000000000000000000000000000000000000000000000000000000000000000000000000000000000000000000000000000000000000000000000000000000000000000000
00000000000000000000000000000000000000000000000000000000000000000000000000000000000000000000000000000000000000000000000000000000000000000000000000000000000000000000000000000000
00000000000000000000000000000000000000000000000000000000000000000000000000000000000000000000000000000000000000000000000000000000000000000000000000000000000000000000000000000000
00000000000000000000000000000000000000000000000000000000000000000000000000000000000000000000000000000000000000000000000000000000000000000000000000000000000000000000000000000000
0000000000000000000000000000000000000000000000000000000000000000000000000000000000000000
00000000000000000000000000000000000000000000000000000000000000000000000000000000000000000000000000000000000000000000000000000000000000000000000000000000000000000000000000000000
00000000000000000000000000000000000000000000000000000000000000000000000000000000000000000000000000000000000000000000000000000000000000000000000000000000000000000000000000000000
00000000000000000000000000000000000000000000000000000000000000000000000000000000000000000000000000000000000000000000000000000000000000000000000000000000000000000000000000000000
00000000000000000000000000000000000000000000000000000000000000000000000000000000000000000000000000000000000000000000000000000000000000000000000000000000000000000000000000000000
00000000000000000000000000000000000000000000000000000000000000000000000000000000000000000000000000000000000000000000000000000000000000000000000000000000000000000000000000000000
00000000000000000000000000000000000000000000000000000000000000000000000000000000000000000000000000000000000000000000000000000000000000000000000000000000000000000000000000000000
00000000000000000000000000000000000000000000000000000000000000000000000000000000000000000000000000000000000000000000000000000000000000000000000000000000000000000000000000000000
00000000000000000000000000000000000000000000000000000000000000000000000000000000000000000000000000000000000000000000000000000000000000000000000000000000000000000000000000000000
00000000000000000000000000000000000000000000000000000000000000000000000000000000000000000000000000000000000000000000000000000000000000000000000000000000000000000000000000000000
00000000000000000000000000000000000000000000000000000000000000000000000000000000000000000000000000000000000000000000000000000000000000000000000000000000000000000000000000000000
00000000000000000000000000000000000000000000000000000000000000000000000000000000000000000000000000000000000000000000000000000000000000000000000000000000000000000000000000000000
00000000000000000000000000000000000000000000000000000000000000000000000000000000000000000000000000000000000000000000000000000000000000000000000000000000000000000000000000000000
00000000000000000000000000000000000000000000000000000000000000000000000000000000000000000000000000000000000000000000000000000000000000000000000000000000000000000000000000000000
00000000000000000000000000000000000000000000000000000000000000000000000000000000000000000000000000000000000000000000000000000000000000000000000000000000000000000000000000000000
00000000000000000000000000000000000000000000000000000000000000000000000000000000000000000000000000000000000000000000000000000000000000000000000000000000000000000000000000000000
00000000000000000000000000000000000000000000000000000000000000000000000000000000000000000000000000000000000000000000000000000000000000000000000000000000000000000000000000000000
00000000000000000000000000000000000000000000000000000000000000000000000000000000000000000000000000000000000000000000000000000000000000000000000000000000000000000000000000000000
00000000000000000000000000000000000000000000000000000000000000000000000000000000000000000000000000000000000000000000000000000000000000000000000000000000000000000000000000000000
00000000000000000000000000000000000000000000000000000000000000000000000000000000000000000000000000000000000000000000000000000000000000000000000000000000000000000000000000000000
0000000000000000000000000000000000000000000000000000000000000000000000000000000000000000
00000000000000000000000000000000000000000000000000000000000000000000000000000000000000000000000000000000000000000000000000000000000000000000000000000000000000000000000000000000
00000000000000000000000000000000000000000000000000000000000000000000000000000000000000000000000000000000000000000000000000000000000000000000000000000000000000000000000000000000
00000000000000000000000000000000000000000000000000000000000000000000000000000000000000000000000000000000000000000000000000000000000000000000000000000000000000000000000000000000
00000000000000000000000000000000000000000000000000000000000000000000000000000000000000000000000000000000000000000000000000000000000000000000000000000000000000000000000000000000
00000000000000000000000000000000000000000000000000000000000000000000000000000000000000000000000000000000000000000000000000000000000000000000000000000000000000000000000000000000
00000000000000000000000000000000000000000000000000000000000000000000000000000000000000000000000000000000000000000000000000000000000000000000000000000000000000000000000000000000
00000000000000000000000000000000000000000000000000000000000000000000000000000000000000000000000000000000000000000000000000000000000000000000000000000000000000000000000000000000
00000000000000000000000000000000000000000000000000000000000000000000000000000000000000000000000000000000000000000000000000000000000000000000000000000000000000000000000000000000
00000000000000000000000000000000000000000000000000000000000000000000000000000000000000000000000000000000000000000000000000000000000000000000000000000000000000000000000000000000
00000000000000000000000000000000000000000000000000000000000000000000000000000000000000000000000000000000000000000000000000000000000000000000000000000000000000000000000000000000
00000000000000000000000000000000000000000000000000000000000000000000000000000000000000000000000000000000000000000000000000000000000000000000000000000000000000000000000000000000
00000000000000000000000000000000000000000000000000000000000000000000000000000000000000000000000000000000000000000000000000000000000000000000000000000000000000000000000000000000
00000000000000000000000000000000000000000000000000000000000000000000000000000000000000000000000000000000000000000000000000000000000000000000000000000000000000000000000000000000
00000000000000000000000000000000000000000000000000000000000000000000000000000000000000000000000000000000000000000000000000000000000000000000000000000000000000000000000000000000

0000000000000000000000000000000000000000000000000000000000000000000000000000000000000000000000000000000000000000000000000000000000000000000000000000000000000000000000000000000
0000000000000000000000000000000000000000000000000000000000000000000000000000000000000000000000000000000000000000000000000000000000000000000000000000000000000000000000000000000
0000000000000000000000000000000000000000000000000000000000000000000000000000000000000000000000000000000000000000000000000000000000000000000000000000000000000000000000000000000
0000000000000000000000000000000000000000000000000000000000000000000000000000000000000000000000000000000000000000000000000000000000000000000000000000000000000000000000000000000
0000000000000000000000000000000000000000000000000000000000000000000000000000000000000000000000000000000000000000000000000000000000000000000000000000000000000000000000000000000
0000000000000000000000000000000000000000000000000000000000000000000000000000000000000000
0000000000000000000000000000000000000000000000000000000000000000000000000000000000000000000000000000000000000000000000000000000000000000000000000000000000000000000000000000000
0000000000000000000000000000000000000000000000000000000000000000000000000000000000000000000000000000000000000000000000000000000000000000000000000000000000000000000000000000000
0000000000000000000000000000000000000000000000000000000000000000000000000000000000000000000000000000000000000000000000000000000000000000000000000000000000000000000000000000000
0000000000000000000000000000000000000000000000000000000000000000000000000000000000000000000000000000000000000000000000000000000000000000000000000000000000000000000000000000000
0000000000000000000000000000000000000000000000000000000000000000000000000000000000000000000000000000000000000000000000000000000000000000000000000000000000000000000000000000000
0000000000000000000000000000000000000000000000000000000000000000000000000000000000000000000000000000000000000000000000000000000000000000000000000000000000000000000000000000000
0000000000000000000000000000000000000000000000000000000000000000000000000000000000000000000000000000000000000000000000000000000000000000000000000000000000000000000000000000000
0000000000000000000000000000000000000000000000000000000000000000000000000000000000000000000000000000000000000000000000000000000000000000000000000000000000000000000000000000000
0000000000000000000000000000000000000000000000000000000000000000000000000000000000000000000000000000000000000000000000000000000000000000000000000000000000000000000000000000000
0000000000000000000000000000000000000000000000000000000000000000000000000000000000000000000000000000000000000000000000000000000000000000000000000000000000000000000000000000000
0000000000000000000000000000000000000000000000000000000000000000000000000000000000000000000000000000000000000000000000000000000000000000000000000000000000000000000000000000000
0000000000000000000000000000000000000000000000000000000000000000000000000000000000000000000000000000000000000000000000000000000000000000000000000000000000000000000000000000000
0000000000000000000000000000000000000000000000000000000000000000000000000000000000000000000000000000000000000000000000000000000000000000000000000000000000000000000000000000000
0000000000000000000000000000000000000000000000000000000000000000000000000000000000000000000000000000000000000000000000000000000000000000000000000000000000000000000000000000000
0000000000000000000000000000000000000000000000000000000000000000000000000000000000000000000000000000000000000000000000000000000000000000000000000000000000000000000000000000000
0000000000000000000000000000000000000000000000000000000000000000000000000000000000000000000000000000000000000000000000000000000000000000000000000000000000000000000000000000000
0000000000000000000000000000000000000000000000000000000000000000000000000000000000000000000000000000000000000000000000000000000000000000000000000000000000000000000000000000000
0000000000000000000000000000000000000000000000000000000000000000000000000000000000000000000000000000000000000000000000000000000000000000000000000000000000000000000000000000000
0000000000000000000000000000000000000000000000000000000000000000000000000000000000000000000000000000000000000000000000000000000000000000000000000000000000000000000000000000000
0000000000000000000000000000000000000000000000000000000000000000000000000000000000000000
>P23145
MSLLAQWREDIRCVFERDPAARTTFEVLTTYPGVHAIMLYRLAHRLWRPNALPRPAAVVRARLVSNVDIHPGAVIGARFFIDHGACVVIGETAEIGRDVTLYHGVTLGGTTGAKGKRHPTLGDVVLVGAGAKILGPITIGANARVGANSVVVQDVPEGCTVVGIPGKVVKLREAG
QLNPYGIDLDHHLIPDPVGKAIACLLERIDSLEKRVEAGGLVAAAASSTFYEGCNPDNSICETNLRRSAPWSSGRPRRPAHAGDRVSGRAKGSD
0000000000000000000000000000000000000000000000000000000000000000000000000000000000000000000000000000000000000000000000000000000000000000000000000000000000000000000000000000000
0000000000000000000000000000000000000000000000000000000000000000000000000000000000000000000000
0000000000000000000000000000000000000000000000000000000000000000000000000000000000000000000000000000000000000000000000000000000000000000000000000000000000000000000000000000000
0000000000000000000000000000000000000000000000000000000000000000000000000000000000000000000000
0000000000000000000000000000000000000000000000000000000000000000000000000000000000000000000000000000000000000000000000000000000000000000000000000000000000000000000000000000000
0000000000000000000000000000000000000000000000000000000000000000000000000000000000000000000000
0000000000000000000000000000000000000000000000000000000000000000000000000000000000000000000000000000000000000000000000000000000000000000000000000000000000000000000000000000000
0000000000000000000000000000000000000000000000000000000000000000000000000000000000000000000000
0000000000000000000000000000000000000000000000000000000000000000000000000000000000000000000000000000000000000000000000000000000000000000000000000000000000000000000000000000000
0000000000000000000000000000000000000000000000000000000000000000000000000000000000000000000000
0000000000000000000000000000000000000000000000000000000000000000000000000000000000000000000000000000000000000000000000000000000000000000000000000000000000000000000000000000000
0000000000000000000000000000000000000000000000000000000000000000000000000000000000000000000000
0000000000000000000000000000000000000000000000000000000000000000000000000000000000000000000000000000000000000000000000000000000000000000000000000000000000000000000000000000000
0000000000000000000000000000000000000000000000000000000000000000000000000000000000000000000000
>P32057
MKILVYGINYSPELTGIGKYTGEMVEWLAAQGHEVRVITAPPYYPQWQVGENYSAWRYKREEGAATVWRCPLYVPKQPSTLKRLLHLGSFAVSSFFPLMAQRRWKPDRIIGVVPTLFCAPGMRLLAKLSGARTVLHIQDYEVDAMLGLGLAGKGKGGKVAQLATAFERSGLHNVD
NVSTISRSMMNKAIEKGVAAENVIFFPNWSEIARFQHVADADVDALRNQLDLPDNKKIILYSGNIGEKQGLENVIEAADRLRDEPLIFAIVGQGGGKARLEKMAQQRGLRNMQFFPLQSYDALPALLKMGDCHLVVQKRGAADAVLPSKLTNILAVGGNAVITAEAYTELGQLCE
TFPGIAVCVEPESVEALVAGIRQALLLPKHNTVAREYAERTLDKENVLRQFINDIRG
0000000000000000000000000000000000000000000000000000000000000000000000000000000000000000000000000000000000000000000000000000000000000000000000000000000000000000000000000000000
0000000000000000000000000000000000000000000000000000000000000000000000000000000000000000000000000000000000000000000000000000000000000000000000000000000000000000000000000000000
000000000000000000000000000000000000000000000000000000000
0000000000000000000000000000000000000000000000000000000000000000000000000000000000000000000000000000000000000000000000000000000000000000000000000000000000000000000000000000000
0000000000000000000000000000000000000000000000000000000000000000000000000000000000000000000000000000000000000000000000000000000000000000000000000000000000000000000000000000000
000000000000000000000000000000000000000000000000000000000
0000000000000000000000000000000000000000000000000000000000000000000000000000000000000000000000000000000000000000000000000000000000000000000000000000000000000000000000000000000
0000000000000000000000000000000000000000000000000000000000000000000000000000000000000000000000000000000000000000000000000000000000000000000000000000000000000000000000000000000
000000000000000000000000000000000000000000000000000000000
0000000000000000000000000000000000000000000000000000000000000000000000000000000000000000000000000000000000000000000000000000000000000000000000000000000000000000000000000000000
0000000000000000000000000000000000000000000000000000000000000000000000000000000000000000000000000000000000000000000000000000000000000000000000000000000000000000000000000000000
000000000000000000000000000000000000000000000000000000000
0000000000000000000000000000000000000000000000000000000000000000000000000000000000000000000000000000000000000000000000000000000000000000000000000000000000000000000000000000000
0000000000000000000000000000000000000000000000000000000000000000000000000000000000000000000000000000000000000000000000000000000000000000000000000000000000000000000000000000000
000000000000000000000000000000000000000000000000000000000
0000000000000000000000000000000000000000000000000000000000000000000000000000000000000000000000000000000000000000000000000000000000000000000000000000000000000000000000000000000
0000000000000000000000000000000000000000000000000000000000000000000000000000000000000000000000000000000000000000000000000000000000000000000000000000000000000000000000000000000
000000000000000000000000000000000000000000000000000000000
0000000000000000000000000000000000000000000000000000000000000000000000000000000000000000000000000000000000000000000000000000000000000000000000000000000000000000000000000000000
0000000000000000000000000000000000000000000000000000000000000000000000000000000000000000000000000000000000000000000000000000000000000000000000000000000000000000000000000000000
000000000000000000000000000000000000000000000000000000000
>P34451

MKHKRMMLPHYVRVYVCVRTDGENEKAGQSEEYDDDDKEENKKRRRNNGRRGPPEKKKSRRGGEEQTQRII
00000000000000000000000000000000000000000000000000000000000000000000000
00000000000000000000000000000000000000000000000000000000000000000000000
00000000000000000000000000000000000000000000000000000000000000000000000
00000000000000000000000000000000000000000000000000000000000000000000000
00000000000000000000000000000000000000000000000000000000000000000000000
00000000000000000000000000000000000000000000000000000000000000000000000
00000000000000000000000000000000000000000000000000000000000000000000000
>P36705
MVTVLLIFLCLPVIFSSSTFAAVSDLDPECLAPFAVYLIFTFVTATCVCSIITLLITSLQFFDYYYVRIVYRRHHPRYQNPQIAALLQLQP
0000000000000000000000000000000000000000000000000000000000000000000000000000000000000000000
0000000000000000000000000000000000000000000000000000000000000000000000000000000000000000000
0000000000000000000000000000000000000000000000000000000000000000000000000000000000000000000
0000000000000000000000000000000000000000000000000000000000000000000000000000000000000000000
0000000000000000000000000000000000000000000000000000000000000000000000000000000000000000000
0000000000000000000000000000000000000000000000000000000000000000000000000000000000000000000
0000000000000000000000000000000000000000000000000000000000000000000000000000000000000000000
>P38374
MAVQTPRQRLANAKFNKNNEKYRKYGKKKEGKTEKTAPVISKTWLGILLFLLVGGGVLQLISYIL
00000000000000000000000000000000000000000000000000000000000000000
00000000000000000000000000000000000000000000000000000000000000000
00000000000000000000000000000000000000000000000000000000000000000
00000000000000000000000000000000000000000000000000000000000000000
00000000000000000000000000000000000000000000000000000000000000000
00000000000000000000000000000000000000000000000000000000000000000
00000000000000000000000000000000000000000000000000000000000000000
>P39743
MSFKGFTKAVSRAPQSFRQKFKMGEQTEDPVYEDAERRFQELEQETKKLSEESKRYSTAVNGMLTHQIGFAKSMEEIFKPISGKMSDPNATIPEDNPQGIEASEQYRAIVAELQETLKPDLALVEEKIVTPCQELLKIITYIRKMATKRNHKKLDLDRHLNTYNKHEKKKEPTAK
DEERLYKAQAQVEVAQQEYDYYNDLLKTQLPILFSLEAEFVKPLFVSFYFMQLNIFYTLYNRLQDMKIPYFDLNSDIVESYIAKKGNVEEQTDALTITHFKLGYSKAKLEMTRRKYGVATAEGSPVSGASSGVGYGAGYDPATATSPTPTGYGYGAAAPSYAAQPAAQYGTAAAV
GTAAAVGTAAGAAAGAVPGTYPQYAAAQSPPLTGLGFQQSPQQQQGPPPAYSNPLTSPVAGTPAAAVAAAPGVETVTALYDYQAQAAGDLSFPAGAVIEIVQRTPDVNEWWTGRYNGQQGVFPGNYVQLNKN
0000000000000000000000000000000000000000000000000000000000000000000000000000000000000000000000000000000000000000000000000000000000000000000000000000000000000000000000000000000
0000000000000000000000000000000000000000000000000000000000000000000000000000000000000000000000000000000000000000000000000000000000000000000000000000000000000000000000000000000
000000000000000000000000000000000000000000000000000000000000000000000000000000000000000000000000000000000000000000000000000000000000
0000000000000000000000000000000000000000000000000000000000000000000000000000000000000000000000000000000000000000000000000000000000000000000000000000000000000000000000000000000
0000000000000000000000000000000000000000000000000000000000000000000000000000000000000000000000000000000000000000000000000000000000000000000000000000000000000000000000000000000
000000000000000000000000000000000000000000000000000000000000000000000000000000000000000000000000000000000000000000000000000000000000
0000000000000000000000000000000000000000000000000000000000000000000000000000000000000000000000000000000000000000000000000000000000000000000000000000000000000000000000000000000
0000000000000000000000000000000000000000000000000000000000000000000000000000000000000000000000000000000000000000000000000000000000000000000000000000000000000000000000000000000
000000000000000000000000000000000000000000000000000000000000000000000000000000000000000000000000000000000000000000000000000000000000
0000000000000000000000000000000000000000000000000000000000000000000000000000000000000000000000000000000000000000000000000000000000000000000000000000000000000000000000000000000
0000000000000000000000000000000000000000000000000000000000000000000000000000000000000000000000000000000000000000000000000000000000000000000000000000000000000000000000000000000
000000000000000000000000000000000000000000000000000000000000000000000000000000000000000000000000000000000000000000000000000000000000
0000000000000000000000000000000000000000000000000000000000000000000000000000000000000000000000000000000000000000000000000000000000000000000000000000000000000000000000000000000
0000000000000000000000000000000000000000000000000000000000000000000000000000000000000000000000000000000000000000000000000000000000000000000000000000000000000000000000000000000
000000000000000000000000000000000000000000000000000000000000000000000000000000000000000000000000000000000000000000000000000000000000
0000000000000000000000000000000000000000000000000000000000000000000000000000000000000000000000000000000000000000000000000000000000000000000000000000000000000000000000000000000
0000000000000000000000000000000000000000000000000000000000000000000000000000000000000000000000000000000000000000000000000000000000000000000000000000000000000000000000000000000
000000000000000000000000000000000000000000000000000000000000000000000000000000000000000000000000000000000000000000000000000000000000
0000000000000000000000000000000000000000000000000000000000000000000000000000000000000000000000000000000000000000000000000000000000000000000000000000000000000000000000000000000
0000000000000000000000000000000000000000000000000000000000000000000000000000000000000000000000000000000000000000000000000000000000000000000000000000000000000000000000000000000
000000000000000000000000000000000000000000000000000000000000000000000000000000000000000000000000000000000000000000000000000000000000
>P40001
MLMHFIPRRSSKAVQFNWLEIGVWRGGVYVKKGIVNARTNNSNFYKMPVVFGIVKGMGALCVHAWGMGYGGIIDCSPVTEHGNISVLQLEYMPWICLSKIMSCSVASRHSNVWIMDETIAPLVGCL
000000000000000000000000000000000000000000000000000000000000000000000000000000000000000000000000000000000000000000000000000000
000000000000000000000000000000000000000000000000000000000000000000000000000000000000000000000000000000000000000000000000000000
000000000000000000000000000000000000000000000000000000000000000000000000000000000000000000000000000000000000000000000000000000
000000000000000000000000000000000000000000000000000000000000000000000000000000000000000000000000000000000000000000000000000000
000000000000000000000000000000000000000000000000000000000000000000000000000000000000000000000000000000000000000000000000000000
000000000000000000000000000000000000000000000000000000000000000000000000000000000000000000000000000000000000000000000000000000
000000000000000000000000000000000000000000000000000000000000000000000000000000000000000000000000000000000000000000000000000000
>P44479
MRLLYVIKKENPMDQMPNCPKCQSEYVYHDSINFVCPDCGNEWDNNEIQESDDDQLIVKDSNGNLLAEGDNVLLIKDLKLKGSSEVLKKGTKFKNIRLVNGDHNVDCGKIMLKSEFLKKA
000000000000000000000000000000000000000000000000000000000000000000000000000000000000000000000000000000000000000000000000
000000000000000000000000000000000000000000000000000000000000000000000000000000000000000000000000000000000000000000000000
000000000000000000000000000000000000000000000000000000000000000000000000000000000000000000000000000000000000000000000000
000000000000000000000000000000000000000000000000000000000000000000000000000000000000000000000000000000000000000000000000
000000000000000000000000000000000000000000000000000000000000000000000000000000000000000000000000000000000000000000000000
000000000000000000000000000000000000000000000000000000000000000000000000000000000000000000000000000000000000000000000000
000000000000000000000000000000000000000000000000000000000000000000000000000000000000000000000000000000000000000000000000

>P46854
MSEIVIRHAETRDYEAIRQIHAQPEVYCNTLQVPHPSDHMWQERLADRPGIKQLVACIDGDVVGHLTIDVQQRPRRSHVADFGICVDSRWKNRGVASALMREMIEMCDNWLRVDRIELTVFVDNAPAIKVYKKYGFEIEGTGKKYALRNGEYVDAYYMARVK
000000000000000000000000000000000000000000000000000000000000000000000000000000000000000000000000000000000000000000000000000000000000000000000000000000000000000000
000000000000000000000000000000000000000000000000000000000000000000000000000000000000000000000000000000000000000000000000000000000000000000000000000000000000000000
000000000000000000000000000000000000000000000000000000000000000000000000000000000000000000000000000000000000000000000000000000000000000000000000000000000000000000
000000000000000000000000000000000000000000000000000000000000000000000000000000000000000000000000000000000000000000000000000000000000000000000000000000000000000000
000000000000000000000000000000000000000000000000000000000000000000000000000000000000000000000000000000000000000000000000000000000000000000000000000000000000000000
000000000000000000000000000000000000000000000000000000000000000000000000000000000000000000000000000000000000000000000000000000000000000000000000000000000000000000
000000000000000000000000000000000000000000000000000000000000000000000000000000000000000000000000000000000000000000000000000000000000000000000000000000000000000000
>P51592
MVSMQFVLQPLPGSDDQFIERIREVSDKVNRFGYGSHRIFEQLKIPVKEVVIGPAHIGVLLEDGKAFRVSFSINSEKLDLTKSDAKCSTSGGSGTASASKAPSSSRPMARSRARLLRATGRSNSTGQGSGSRSTGVIIGGSTSSRPLVTVPATYVPEELISQAEVVLQGKSRNLI
IRELQRTNLDVNLAVNNLLSRDDEEAEDTEEGADNYVPEDLISLLDNGFSGDNNSVIIDPSDGLFSEEIFSNYSSIRNLLFDRIRSERSNANANAADSNQSTTRSTSSGTALTGNSGLSAQISVNADREAFSRWRDRQYYGPRRWISKDDYTWEKDADSKKKEPSPMLSPIWISE
ELQPWPEKSSVRFKTIGALYSEFIALSESGDLYQWRWSDAEPYKSETENVYHPKTVSLNIVERVELISANFIRCSVVTETNRVATWMDEQLGYIGAKLEHSCCAFNEFISDSITKIYVCSLYTVVKTESNNIYWWGVLPFDQRRFLWDKFRTKTKKPFKVVATDINVGAQVIMKK
CPMYQSGSIGFTCSNGVPKVGQLLNSVWTFTDVCRMKIININTNSGVDKSQAAGNNLNAHGITPDKDLPKSTAMPSTGSSKNGQSFSNSKESTDRIDMPPPPSPASSTCSDTGSVTSHKRTKRATTKEDSNAPQEGRKDEELWEVKDVVFVEDKVGPVGKVLKVDGDFVAVRFPA
INAAAVAAAAAATSSTSNTASTSKEEGKEDDWQQCRLLRREDVQIFRTAMSTRGPDWLQKQPKKINVGGDAAGAQILTLAVDSRGIHVIKKVLGKIHYSLYNLYNCKQEQNCLFPTDCNSFIGSTPGNILMACNDDCSGNSSTIVLRDGNGALYPLAKDCLGSIKDPQWFDLPPV
KSITMSTISLPAMLSGVNLKSKVCMTALLFDTQKLMPHILRCDVKNSFAALGRLEREDQADTALVVEERCDGARNIFHACVIMCAPSSNKDSPPDSPSGGVEKKSLVGLSVARSIPTVSTSAYVSSIAFGASASSSNENSSFATMSSSAAGSASSTSRDNRTNLRDMMNRLINSD
QAEQSGSQPMATNNEDHAYIPWPAETPAASNLSASSSQNVSDSIEDDISKIIPSSSQSSMLSNIKLGSPTYTFDLAQRREHALTILQQMCVSPALRPYLCHMLSTKDAQGQTPFMLSVSCRAYEAGIILLNTILMLSEQDPQLKEAMIFPNGSPADQSPLHVICYNDTCSFTWTG
ADHINQNIFECKTCGLTGSLCCCTECARVCHKGHDCKLKRTAPTAYCDCWEKCKCKALIAGNLTKRFALLCKLVSCTDLVTKFNSKGESILLFLIQTVGRQIVEQRQYRFSVRVRNVSTAATGATGNNSVISNRKTSAAEIDNDMPDHDLEPPKFARKALERLLIDWNAVRSMIM
SGAERGDVPNPAGSASENSNSEGFNMFIQTQHGSTLLDKFTHSLIVKCTSDHLDTLLLTLVRELQNASVSNRSKEAEEVVRRFVRSVARVFVIFNLEKQPNPEKRKSHSSCNKYVQSCVKVFQTLHKISIEELCEVSEALIAPVRLGVVRPTAPFTMSSSNLDNSDDLFSVDPLA
PSNVESPSEQILVHDAGNDQSANFNIQQNYDVVAMETIRDASESEEVINREANSHNQDDELIENQRNEDGMQDDESDNDFTFNDAETESDSDDNQSNQEVQRSVQAGATVGSENDIGVLFLEDESGDSSAQEEDGSEDGESDDQSDEFNFNEQQLERRSTNSNARSDLAPQTMQW
AIRSRDTARSSVRVPTGSNMVFIDPMALRRSTVPASTTVTTPSIEPHTMATTASNLARAFGITIRQISELISILSYNVLNDIETSLKIQNDEAIAVQAFVEKRLKATWDWMFTVMDGTEAQLKFGAYLTNYTDPNHPLHPLNLSAQASSSQTPAPATSSSVNGVNIMGSNSRRDF
FTYCLSLMRSHTSEHRDALPVLDITALRHIAYVLDAFVYYMRNDSGFYDKQDTISGRINNLSPMTESYDTDDELANLEEFNADVQMSASSMPSGSQGTRRHAFFARSESTLSLGCSAPEGFELPLDMAMPLADKPHLLQPNSKRQELFANLPLLVTTNANNSGATNDGDGGSIFD
YTPTRLGFSNSLKRNERVYETVPIDSSKTGDGNVTNKAEGSTDSNIYVQLKKKQGSDDFKSHKEADGNQSKYEKVVLMETDDSLPSTSKSTEALMATRPEVIIAPNKASVSPATAARSVIVLAGGSCLKTIDSDINNYSASNLSTAEQAKCDTQYQKSTSDHLLLFPARGSQFYQ
SNFSELPSWNFLLSRWKLTLDLFGRVFMDDVGMEHGSVLPELRGFPVKEMRFRRHMEKLRNGQQRDLVLCKLERNRESLIVQTFKELNTQFGNQSRRIQPPITFNRVKVTFKDEPGEGSGVARSFYTSIAEALLASAKIPNLESVQVGTNHSKYVVPFSSILRSRTVSGSSRDQS
TLQRRGSNSKILWRSARERKALNLDARPYTPPNSSDNATPESLNDHLSVHLQQIGERLYPKIHSINQTHAPKITGMLLEIPTPQLLSVISSDETLRQKVNEAIEIITFKQKSETSAQSSQPKKSPSVVVVDPVDDDNEPLFYSPGKRGFYTPRQGFASFERINAFRNIGRLIGLC
LLQNELLPLFLQRHVLKYILGRKIKFHDLAFFDPALYESFRQIIQNAQTKEGEETINRMELCFVIDLMKEEGCGNRELIPGGRDVAVTSSNIFEYVRRYTEYRLIKSQEKALEALKDGVFDVLPDNSMINLTAEDLRLLLNGVGDINVSTLISYTTFNDESSEGPDKLLKFKKWF
WSIVEKMNIMERQDLVYFWTGSPALPASEEGFQPLPSVTIRPADDSHLPTANTCISRLYIPLYSSKSILRSKMLMAIKSKNFGFV
0000000000000000000000000000000000000000000000000000000000000000000000000000000000000000000000000000000000000000000000000000000000000000000000000000000000000000000000000000
0000000000000000000000000000000000000000000000000000000000000000000000000000000000000000000000000000000000000000000000000000000000000000000000000000000000000000000000000000
0000000000000000000000000000000000000000000000000000000000000000000000000000000000000000000000000000000000000000000000000000000000000000000000000000000000000000000000000000
0000000000000000000000000000000000000000000000000000000000000000000000000000000000000000000000000000000000000000000000000000000000000000000000000000000000000000000000000000
0000000000000000000000000000000000000000000000000000000000000000000000000000000000000000000000000000000000000000000000000000000000000000000000000000000000000000000000000000
0000000000000000000000000000000000000000000000000000000000000000000000000000000000000000000000000000000000000000000000000000000000000000000000000000000000000000000000000000
0000000000000000000000000000000000000000000000000000000000000000000000000000000000000000000000000000000000000000000000000000000000000000000000000000000000000000000000000000
0000000000000000000000000000000000000000000000000000000000000000000000000000000000000000000000000000000000000000000000000000000000000000000000000000000000000000000000000000
0000000000000000000000000000000000000000000000000000000000000000000000000000000000000000000000000000000000000000000000000000000000000000000000000000000000000000000000000000
0000000000000000000000000000000000000000000000000000000000000000000000000000000000000000000000000000000000000000000000000000000000000000000000000000000000000000000000000000
0000000000000000000000000000000000000000000000000000000000000000000000000000000000000000000000000000000000000000000000000000000000000000000000000000000000000000000000000000
0000000000000000000000000000000000000000000000000000000000000000000000000000000000000000000000000000000000000000000000000000000000000000000000000000000000000000000000000000
0000000000000000000000000000000000000000000000000000000000000000000000000000000000000000000000000000000000000000000000000000000000000000000000000000000000000000000000000000
0000000000000000000000000000000000000000000000000000000000000000000000000000000000000000000000000000000000000000000000000000000000000000000000000000000000000000000000000000
0000000000000000000000000000000000000000000000000000000000000000000000000000000000000000000000000000000000000000000000000000000000000000000000000000000000000000000000000000
0000000000000000000000000000000000000000000000000000000000000000000000000000000000000000000000000000000000000000000000000000000000000000000000000000000000000000000000000000
0000000000000000000000000000000000000000000000000000000000000000000000000000000000000
0000000000000000000000000000000000000000000000000000000000000000000000000000000000000000000000000000000000000000000000000000000000000000000000000000000000000000000000000000
0000000000000000000000000000000000000000000000000000000000000000000000000000000000000000000000000000000000000000000000000000000000000000000000000000000000000000000000000000
0000000000000000000000000000000000000000000000000000000000000000000000000000000000000000000000000000000000000000000000000000000000000000000000000000000000000000000000000000
0000000000000000000000000000000000000000000000000000000000000000000000000000000000000000000000000000000000000000000000000000000000000000000000000000000000000000000000000000
0000000000000000000000000000000000000000000000000000000000000000000000000000000000000000000000000000000000000000000000000000000000000000000000000000000000000000000000000000
0000000000000000000000000000000000000000000000000000000000000000000000000000000000000000000000000000000000000000000000000000000000000000000000000000000000000000000000000000
0000000000000000000000000000000000000000000000000000000000000000000000000000000000000000000000000000000000000000000000000000000000000000000000000000000000000000000000000000
0000000000000000000000000000000000000000000000000000000000000000000000000000000000000000000000000000000000000000000000000000000000000000000000000000000000000000000000000000
0000000000000000000000000000000000000000000000000000000000000000000000000000000000000000000000000000000000000000000000000000000000000000000000000000000000000000000000000000
0000000000000000000000000000000000000000000000000000000000000000000000000000000000000000000000000000000000000000000000000000000000000000000000000000000000000000000000000000
0000000000000000000000000000000000000000000000000000000000000000000000000000000000000000000000000000000000000000000000000000000000000000000000000000000000000000000000000000
0000000000000000000000000000000000000000000000000000000000000000000000000000000000000000000000000000000000000000000000000000000000000000000000000000000000000000000000000000
0000000000000000000000000000000000000000000000000000000000000000000000000000000000000000000000000000000000000000000000000000000000000000000000000000000000000000000000000000
0000000000000000000000000000000000000000000000000000000000000000000000000000000000000000000000000000000000000000000000000000000000000000000000000000000000000000000000000000
0000000000000000000000000000000000000000000000000000000000000000000000000000000000000000000000000000000000000000000000000000000000000000000000000000000000000000000000000000
0000000000000000000000000000000000000000000000000000000000000000000000000000000000000000000000000000000000000000000000000000000000000000000000000000000000000000000000000000
0000000000000000000000000000000000000000000000000000000000000000000000000000000000000
0000000000000000000000000000000000000000000000000000000000000000000000000000000000000000000000000000000000000000000000000000000000000000000000000000000000000000000000000000
0000000000000000000000000000000000000000000000000000000000000000000000000000000000000000000000000000000000000000000000000000000000000000000000000000000000000000000000000000
0000000000000000000000000000000000000000000000000000000000000000000000000000000000000000000000000000000000000000000000000000000000000000000000000000000000000000000000000000
0000000000000000000000000000000000000000000000000000000000000000000000000000000000000000000000000000000000000000000000000000000000000000000000000000000000000000000000000000
0000000000000000000000000000000000000000000000000000000000000000000000000000000000000000000000000000000000000000000000000000000000000000000000000000000000000000000000000000
0000000000000000000000000000000000000000000000000000000000000000000000000000000000000000000000000000000000000000000000000000000000000000000000000000000000000000000000000000
0000000000000000000000000000000000000000000000000000000000000000000000000000000000000000000000000000000000000000000000000000000000000000000000000000000000000000000000000000
0000000000000000000000000000000000000000000000000000000000000000000000000000000000000000000000000000000000000000000000000000000000000000000000000000000000000000000000000000

0000000000000000000000000000000000000000000000000000000000000000000000000000000000000000000000000000000000000000000000000000000000000000000000000000000000000000000000000000000
0000000000000000000000000000000000000000000000000000000000000000000000000000000000000000000000000000000000000000000000000000000000000000000000000000000000000000000000000000000
0000000000000000000000000000000000000000000000000000000000000000000000000000000000000000000000000000000000000000000000000000000000000000000000000000000000000000000000000000000
0000000000000000000000000000000000000000000000000000000000000000000000000000000000000000000000000000000000000000000000000000000000000000000000000000000000000000000000000000000
0000000000000000000000000000000000000000000000000000000000000000000000000000000000000000000000000000000000000000000000000000000000000000000000000000000000000000000000000000000
0000000000000000000000000000000000000000000000000000000000000000000000000000000000000000000000000000000000000000000000000000000000000000000000000000000000000000000000000000000
0000000000000000000000000000000000000000000000000000000000000000000000000000000000000000000000000000000000000000000000000000000000000000000000000000000000000000000000000000000
0000000000000000000000000000000000000000000000000000000000000000000000000000000000000000000000000000000000000000000000000000000000000000000000000000000000000000000000000000000
0000000000000000000000000000000000000000000000000000000000000000000000000000000000000
0000000000000000000000000000000000000000000000000000000000000000000000000000000000000000000000000000000000000000000000000000000000000000000000000000000000000000000000000000000
0000000000000000000000000000000000000000000000000000000000000000000000000000000000000000000000000000000000000000000000000000000000000000000000000000000000000000000000000000000
0000000000000000000000000000000000000000000000000000000000000000000000000000000000000000000000000000000000000000000000000000000000000000000000000000000000000000000000000000000
0000000000000000000000000000000000000000000000000000000000000000000000000000000000000000000000000000000000000000000000000000000000000000000000000000000000000000000000000000000
0000000000000000000000000000000000000000000000000000000000000000000000000000000000000000000000000000000000000000000000000000000000000000000000000000000000000000000000000000000
0000000000000000000000000000000000000000000000000000000000000000000000000000000000000000000000000000000000000000000000000000000000000000000000000000000000000000000000000000000
0000000000000000000000000000000000000000000000000000000000000000000000000000000000000000000000000000000000000000000000000000000000000000000000000000000000000000000000000000000
0000000000000000000000000000000000000000000000000000000000000000000000000000000000000000000000000000000000000000000000000000000000000000000000000000000000000000000000000000000
0000000000000000000000000000000000000000000000000000000000000000000000000000000000000000000000000000000000000000000000000000000000000000000000000000000000000000000000000000000
0000000000000000000000000000000000000000000000000000000000000000000000000000000000000000000000000000000000000000000000000000000000000000000000000000000000000000000000000000000
0000000000000000000000000000000000000000000000000000000000000000000000000000000000000000000000000000000000000000000000000000000000000000000000000000000000000000000000000000000
0000000000000000000000000000000000000000000000000000000000000000000000000000000000000000000000000000000000000000000000000000000000000000000000000000000000000000000000000000000
0000000000000000000000000000000000000000000000000000000000000000000000000000000000000000000000000000000000000000000000000000000000000000000000000000000000000000000000000000000
0000000000000000000000000000000000000000000000000000000000000000000000000000000000000000000000000000000000000000000000000000000000000000000000000000000000000000000000000000000
0000000000000000000000000000000000000000000000000000000000000000000000000000000000000000000000000000000000000000000000000000000000000000000000000000000000000000000000000000000
0000000000000000000000000000000000000000000000000000000000000000000000000000000000000000000000000000000000000000000000000000000000000000000000000000000000000000000000000000000
0000000000000000000000000000000000000000000000000000000000000000000000000000000000000
0000000000000000000000000000000000000000000000000000000000000000000000000000000000000000000000000000000000000000000000000000000000000000000000000000000000000000000000000000000
0000000000000000000000000000000000000000000000000000000000000000000000000000000000000000000000000000000000000000000000000000000000000000000000000000000000000000000000000000000
0000000000000000000000000000000000000000000000000000000000000000000000000000000000000000000000000000000000000000000000000000000000000000000000000000000000000000000000000000000
0000000000000000000000000000000000000000000000000000000000000000000000000000000000000000000000000000000000000000000000000000000000000000000000000000000000000000000000000000000
0000000000000000000000000000000000000000000000000000000000000000000000000000000000000000000000000000000000000000000000000000000000000000000000000000000000000000000000000000000
0000000000000000000000000000000000000000000000000000000000000000000000000000000000000000000000000000000000000000000000000000000000000000000000000000000000000000000000000000000
0000000000000000000000000000000000000000000000000000000000000000000000000000000000000000000000000000000000000000000000000000000000000000000000000000000000000000000000000000000
0000000000000000000000000000000000000000000000000000000000000000000000000000000000000000000000000000000000000000000000000000000000000000000000000000000000000000000000000000000
0000000000000000000000000000000000000000000000000000000000000000000000000000000000000000000000000000000000000000000000000000000000000000000000000000000000000000000000000000000
0000000000000000000000000000000000000000000000000000000000000000000000000000000000000000000000000000000000000000000000000000000000000000000000000000000000000000000000000000000
0000000000000000000000000000000000000000000000000000000000000000000000000000000000000000000000000000000000000000000000000000000000000000000000000000000000000000000000000000000
0000000000000000000000000000000000000000000000000000000000000000000000000000000000000000000000000000000000000000000000000000000000000000000000000000000000000000000000000000000
0000000000000000000000000000000000000000000000000000000000000000000000000000000000000000000000000000000000000000000000000000000000000000000000000000000000000000000000000000000
0000000000000000000000000000000000000000000000000000000000000000000000000000000000000000000000000000000000000000000000000000000000000000000000000000000000000000000000000000000
0000000000000000000000000000000000000000000000000000000000000000000000000000000000000000000000000000000000000000000000000000000000000000000000000000000000000000000000000000000
0000000000000000000000000000000000000000000000000000000000000000000000000000000000000000000000000000000000000000000000000000000000000000000000000000000000000000000000000000000
0000000000000000000000000000000000000000000000000000000000000000000000000000000000000
0000000000000000000000000000000000000000000000000000000000000000000000000000000000000000000000000000000000000000000000000000000000000000000000000000000000000000000000000000000
0000000000000000000000000000000000000000000000000000000000000000000000000000000000000000000000000000000000000000000000000000000000000000000000000000000000000000000000000000000
0000000000000000000000000000000000000000000000000000000000000000000000000000000000000000000000000000000000000000000000000000000000000000000000000000000000000000000000000000000
0000000000000000000000000000000000000000000000000000000000000000000000000000000000000000000000000000000000000000000000000000000000000000000000000000000000000000000000000000000
0000000000000000000000000000000000000000000000000000000000000000000000000000000000000000000000000000000000000000000000000000000000000000000000000000000000000000000000000000000
0000000000000000000000000000000000000000000000000000000000000000000000000000000000000000000000000000000000000000000000000000000000000000000000000000000000000000000000000000000
0000000000000000000000000000000000000000000000000000000000000000000000000000000000000000000000000000000000000000000000000000000000000000000000000000000000000000000000000000000
0000000000000000000000000000000000000000000000000000000000000000000000000000000000000000000000000000000000000000000000000000000000000000000000000000000000000000000000000000000
0000000000000000000000000000000000000000000000000000000000000000000000000000000000000000000000000000000000000000000000000000000000000000000000000000000000000000000000000000000
0000000000000000000000000000000000000000000000000000000000000000000000000000000000000000000000000000000000000000000000000000000000000000000000000000000000000000000000000000000
0000000000000000000000000000000000000000000000000000000000000000000000000000000000000000000000000000000000000000000000000000000000000000000000000000000000000000000000000000000
0000000000000000000000000000000000000000000000000000000000000000000000000000000000000000000000000000000000000000000000000000000000000000000000000000000000000000000000000000000
0000000000000000000000000000000000000000000000000000000000000000000000000000000000000000000000000000000000000000000000000000000000000000000000000000000000000000000000000000000
0000000000000000000000000000000000000000000000000000000000000000000000000000000000000000000000000000000000000000000000000000000000000000000000000000000000000000000000000000000
0000000000000000000000000000000000000000000000000000000000000000000000000000000000000000000000000000000000000000000000000000000000000000000000000000000000000000000000000000000
0000000000000000000000000000000000000000000000000000000000000000000000000000000000000000000000000000000000000000000000000000000000000000000000000000000000000000000000000000000
0000000000000000000000000000000000000000000000000000000000000000000000000000000000000
0000000000000000000000000000000000000000000000000000000000000000000000000000000000000000000000000000000000000000000000000000000000000000000000000000000000000000000000000000000
0000000000000000000000000000000000000000000000000000000000000000000000000000000000000000000000000000000000000000000000000000000000000000000000000000000000000000000000000000000
0000000000000000000000000000000000000000000000000000000000000000000000000000000000000000000000000000000000000000000000000000000000000000000000000000000000000000000000000000000
0000000000000000000000000000000000000000000000000000000000000000000000000000000000000000000000000000000000000000000000000000000000000000000000000000000000000000000000000000000
0000000000000000000000000000000000000000000000000000000000000000000000000000000000000000000000000000000000000000000000000000000000000000000000000000000000000000000000000000000
0000000000000000000000000000000000000000000000000000000000000000000000000000000000000000000000000000000000000000000000000000000000000000000000000000000000000000000000000000000
0000000000000000000000000000000000000000000000000000000000000000000000000000000000000000000000000000000000000000000000000000000000000000000000000000000000000000000000000000000
0000000000000000000000000000000000000000000000000000000000000000000000000000000000000000000000000000000000000000000000000000000000000000000000000000000000000000000000000000000
0000000000000000000000000000000000000000000000000000000000000000000000000000000000000000000000000000000000000000000000000000000000000000000000000000000000000000000000000000000

00000000000000000000000000000000000000000000000000000000000000000000000000000000000000000000000000000000000000000000000000000000000000000000000000000000000000000000000000000
00000000000000000000000000000000000000000000000000000000000000000000000000000000000000000000000000000000000000000000000000000000000000000000000000000000000000000000000000000
00000000000000000000000000000000000000000000000000000000000000000000000000000000000000000000000000000000000000000000000000000000000000000000000000000000000000000000000000000
00000000000000000000000000000000000000000000000000000000000000000000000000000000000000000000000000000000000000000000000000000000000000000000000000000000000000000000000000000
00000000000000000000000000000000000000000000000000000000000000000000000000000000000000000000000000000000000000000000000000000000000000000000000000000000000000000000000000000
00000000000000000000000000000000000000000000000000000000000000000000000000000000000000000000000000000000000000000000000000000000000000000000000000000000000000000000000000000
00000000000000000000000000000000000000000000000000000000000000000000000000000000000000000000000000000000000000000000000000000000000000000000000000000000000000000000000000000
0000000000000000000000000000000000000000000000000000000000000000000000000000000000000
>P54309
MADIYPLGKTHTEELNEIIVESAKEIAEPDTTMIQKLIDEHNPEPLLKGVRYYMCENDIEKKRRTYYDAAGQQLVDDTKTNNRTSHAWHKLFVDQKTQYLVGEPVTFTSDNKTLLEYVNELADDDFDDILNETVKNMSNKGIEYWHPFVDEEGEFDYVIFPAEEMIVVYKDNTRR
DILFALRYYSYKGIMGEETQKAELYTDTHVYYYEKIDGVYQMDYSYGENNPRPHMTKGGQAIGWGRVPIIPFKNNEEMVSDLKFYKDLIDNYDSITSSTMDSFSDFQQIVYVLKNYDGENPKEFTANLRYHSVIKVSGDGGVDTLRAEIPVDSAAKELERIQDELYKSAQAVDNS
PETIGGGATGPALENLYALLDLKANMAERKIRAGLRLFFWFFAEYLRNTGKGDFNPDKELTMTFTRTRIQNDSEIVQSLVQGVTGGIMSKETAVARNPFVQDPEEELARIEEEMNQYAEMQGNLLDDEGGDDDLEEDDPNAGAAESGGAGQVS
00000000000000000000000000000000000000000000000000000000000000000000000000000000000000000000000000000000000000000000000000000000000000000000000000000000000000000000000000000
00000000000000000000000000000000000000000000000000000000000000000000000000000000000000000000000000000000000000000000000000000000000000000000000000000000000000000000000000000
000000000000000000000000000000000000000000000000000000000000000000000000000000000000000000000000000000000000000000000000000000000000000000000000000000000
00000000000000000000000000000000000000000000000000000000000000000000000000000000000000000000000000000000000000000000000000000000000000000000000000000000000000000000000000000
00000000000000000000000000000000000000000000000000000000000000000000000000000000000000000000000000000000000000000000000000000000000000000000000000000000000000000000000000000
000000000000000000000000000000000000000000000000000000000000000000000000000000000000000000000000000000000000000000000000000000000000000000000000000000000
00000000000000000000000000000000000000000000000000000000000000000000000000000000000000000000000000000000000000000000000000000000000000000000000000000000000000000000000000000
00000000000000000000000000000000000000000000000000000000000000000000000000000000000000000000000000000000000000000000000000000000000000000000000000000000000000000000000000000
000000000000000000000000000000000000000000000000000000000000000000000000000000000000000000000000000000000000000000000000000000000000000000000000000000000
00000000000000000000000000000000000000000000000000000000000000000000000000000000000000000000000000000000000000000000000000000000000000000000000000000000000000000000000000000
00000000000000000000000000000000000000000000000000000000000000000000000000000000000000000000000000000000000000000000000000000000000000000000000000000000000000000000000000000
000000000000000000000000000000000000000000000000000000000000000000000000000000000000000000000000000000000000000000000000000000000000000000000000000000000
00000000000000000000000000000000000000000000000000000000000000000000000000000000000000000000000000000000000000000000000000000000000000000000000000000000000000000000000000000
00000000000000000000000000000000000000000000000000000000000000000000000000000000000000000000000000000000000000000000000000000000000000000000000000000000000000000000000000000
000000000000000000000000000000000000000000000000000000000000000000000000000000000000000000000000000000000000000000000000000000000000000000000000000000000
00000000000000000000000000000000000000000000000000000000000000000000000000000000000000000000000000000000000000000000000000000000000000000000000000000000000000000000000000000
00000000000000000000000000000000000000000000000000000000000000000000000000000000000000000000000000000000000000000000000000000000000000000000000000000000000000000000000000000
000000000000000000000000000000000000000000000000000000000000000000000000000000000000000000000000000000000000000000000000000000000000000000000000000000000
00000000000000000000000000000000000000000000000000000000000000000000000000000000000000000000000000000000000000000000000000000000000000000000000000000000000000000000000000000
00000000000000000000000000000000000000000000000000000000000000000000000000000000000000000000000000000000000000000000000000000000000000000000000000000000000000000000000000000
000000000000000000000000000000000000000000000000000000000000000000000000000000000000000000000000000000000000000000000000000000000000000000000000000000000
>P55535
MQAGDCIYRKRLEQPVLDYLAGTGADFFGRLEDQRQRAVELAVLGEVAGRGEQDRRVPVMALGVHGARISAREDQDGASSIGCVPISARIPMRLPPAPAGASRPHHNRRPHPSHAVLGMLAVCGHVKRSGTSLHHGRRPELA
0000000000000000000000000000000000000000000000000000000000000000000000000000000000000000000000000000000000000000000000000000000000000000000000
0000000000000000000000000000000000000000000000000000000000000000000000000000000000000000000000000000000000000000000000000000000000000000000000
0000000000000000000000000000000000000000000000000000000000000000000000000000000000000000000000000000000000000000000000000000000000000000000000
0000000000000000000000000000000000000000000000000000000000000000000000000000000000000000000000000000000000000000000000000000000000000000000000
0000000000000000000000000000000000000000000000000000000000000000000000000000000000000000000000000000000000000000000000000000000000000000000000
0000000000000000000000000000000000000000000000000000000000000000000000000000000000000000000000000000000000000000000000000000000000000000000000
0000000000000000000000000000000000000000000000000000000000000000000000000000000000000000000000000000000000000000000000000000000000000000000000
>P71037
MKIGIIGASGKAGNEILKEAKKRGHEVTAIVRNASKVQEQDVAILEKDVFELTAEDIKPFDAVVNAFGAAPGQEHLHVEAGRALISILKDAKHTRLLVVGGAGSLFVDEAKTTRLMDTPEFPKEYLPTASNQGENLKDLQQTDSISWTFLSPAAFFDPAGKRTGSYQKGKDNVIV
NAKGDSYISYADYAIAVLDELEHPEHKNERFTVVSEAE
00000000000000000000000000000000000000000000000000000000000000000000000000000000000000000000000000000000000000000000000000000000000000000000000000000000000000000000000000000
00000000000000000000000000000000000000
00000000000000000000000000000000000000000000000000000000000000000000000000000000000000000000000000000000000000000000000000000000000000000000000000000000000000000000000000000
00000000000000000000000000000000000000
00000000000000000000000000000000000000000000000000000000000000000000000000000000000000000000000000000000000000000000000000000000000000000000000000000000000000000000000000000
00000000000000000000000000000000000000
00000000000000000000000000000000000000000000000000000000000000000000000000000000000000000000000000000000000000000000000000000000000000000000000000000000000000000000000000000
00000000000000000000000000000000000000
00000000000000000000000000000000000000000000000000000000000000000000000000000000000000000000000000000000000000000000000000000000000000000000000000000000000000000000000000000
00000000000000000000000000000000000000
00000000000000000000000000000000000000000000000000000000000000000000000000000000000000000000000000000000000000000000000000000000000000000000000000000000000000000000000000000
00000000000000000000000000000000000000
00000000000000000000000000000000000000000000000000000000000000000000000000000000000000000000000000000000000000000000000000000000000000000000000000000000000000000000000000000
00000000000000000000000000000000000000
>P96633
MNLNFIVPDINMTFGDMKFMGLNRERYVYDRENNKRTDVLESRIYNIASAVQGGQIEVTIPEYAGAKEIPPFADIELKNPKISAMATSQRDSTYANVMWKLEADDIVVKGGSSVKPAAATGGNEKK
000000000000000000000000000000000000000000000000000000000000000000000000000000000000000000000000000000000000000000000000000000
000000000000000000000000000000000000000000000000000000000000000000000000000000000000000000000000000000000000000000000000000000
000000000000000000000000000000000000000000000000000000000000000000000000000000000000000000000000000000000000000000000000000000
000000000000000000000000000000000000000000000000000000000000000000000000000000000000000000000000000000000000000000000000000000
000000000000000000000000000000000000000000000000000000000000000000000000000000000000000000000000000000000000000000000000000000
000000000000000000000000000000000000000000000000000000000000000000000000000000000000000000000000000000000000000000000000000000
000000000000000000000000000000000000000000000000000000000000000000000000000000000000000000000000000000000000000000000000000000
>P9WIW2

MTAAPTAGGVVTSGVGVAGVGVGLLGMFGPVRVVHVGWLLPLSGVHIELDRLGGFFMALTGAVAAPVGCYLIGYVRREHLGRVPMAVVPLFVAAMLLVPAAGSVTTFLLAWELMAIASLILVLSEHARPQVRSAGLWYAVMTQLGFIAILVGLVVLAAAGGSDRFAGLGAVCDGV
RAAVFMLTLVGFGSKAGLVPLHAWLPRAHPEAPSPVSALMSAAMVNLGIYGIVRFDLQLLGPGPRWWGLALLAVGGTSALYGVLQASVAADLKRLLAYSTTENMGLITLALGAATLFADTGAYGPASIAAAAAMLHMIAHAAFKSLAFMAAGSVLAATGLRDLDLLGGLARRMPA
TTVFFGVAALGACGLPLGAGFVSEWLLVQSLIHAAPGHDPIVALTTPLAVGVVALATGLSVAAMTKAFGIGFLARPRSTQAEAAREAPASMRAGMAIAAGACLVLAVAPLLVAPMVRRAAATLPAAQAVKFTGLGAVVRLPAMSGSIAPGVIAAAVLAAALAVAVLARWRFRRRP
APARLPLWACGAADLTVRMQYTATSFAEPLQRVFGDVLRPDTDIEVTHTAESRYMAERITYRTAVADAIEQRLYTPVVGAVAAMAELLRRAHTGSVHRYLAYGALGVLIVLVVAR
0000000000000000000000000000000000000000000000000000000000000000000000000000000000000000000000000000000000000000000000000000000000000000000000000000000000000000000000000000000
0000000000000000000000000000000000000000000000000000000000000000000000000000000000000000000000000000000000000000000000000000000000000000000000000000000000000000000000000000000
0000000000000000000000000000000000000000000000000000000000000000000000000000000000000000000000000000000000000000000000000000000000000000000000000000000000000000000000000000000
0000000000000000000000000000000000000000000000000000000000000000000000000000000000000000000000000000000000000000000
0000000000000000000000000000000000000000000000000000000000000000000000000000000000000000000000000000000000000000000000000000000000000000000000000000000000000000000000000000000
0000000000000000000000000000000000000000000000000000000000000000000000000000000000000000000000000000000000000000000000000000000000000000000000000000000000000000000000000000000
0000000000000000000000000000000000000000000000000000000000000000000000000000000000000000000000000000000000000000000000000000000000000000000000000000000000000000000000000000000
0000000000000000000000000000000000000000000000000000000000000000000000000000000000000000000000000000000000000000000
0000000000000000000000000000000000000000000000000000000000000000000000000000000000000000000000000000000000000000000000000000000000000000000000000000000000000000000000000000000
0000000000000000000000000000000000000000000000000000000000000000000000000000000000000000000000000000000000000000000000000000000000000000000000000000000000000000000000000000000
0000000000000000000000000000000000000000000000000000000000000000000000000000000000000000000000000000000000000000000000000000000000000000000000000000000000000000000000000000000
0000000000000000000000000000000000000000000000000000000000000000000000000000000000000000000000000000000000000000000
0000000000000000000000000000000000000000000000000000000000000000000000000000000000000000000000000000000000000000000000000000000000000000000000000000000000000000000000000000000
0000000000000000000000000000000000000000000000000000000000000000000000000000000000000000000000000000000000000000000000000000000000000000000000000000000000000000000000000000000
0000000000000000000000000000000000000000000000000000000000000000000000000000000000000000000000000000000000000000000000000000000000000000000000000000000000000000000000000000000
0000000000000000000000000000000000000000000000000000000000000000000000000000000000000000000000000000000000000000000
0000000000000000000000000000000000000000000000000000000000000000000000000000000000000000000000000000000000000000000000000000000000000000000000000000000000000000000000000000000
0000000000000000000000000000000000000000000000000000000000000000000000000000000000000000000000000000000000000000000000000000000000000000000000000000000000000000000000000000000
0000000000000000000000000000000000000000000000000000000000000000000000000000000000000000000000000000000000000000000000000000000000000000000000000000000000000000000000000000000
0000000000000000000000000000000000000000000000000000000000000000000000000000000000000000000000000000000000000000000
0000000000000000000000000000000000000000000000000000000000000000000000000000000000000000000000000000000000000000000000000000000000000000000000000000000000000000000000000000000
0000000000000000000000000000000000000000000000000000000000000000000000000000000000000000000000000000000000000000000000000000000000000000000000000000000000000000000000000000000
0000000000000000000000000000000000000000000000000000000000000000000000000000000000000000000000000000000000000000000000000000000000000000000000000000000000000000000000000000000
0000000000000000000000000000000000000000000000000000000000000000000000000000000000000000000000000000000000000000000
0000000000000000000000000000000000000000000000000000000000000000000000000000000000000000000000000000000000000000000000000000000000000000000000000000000000000000000000000000000
0000000000000000000000000000000000000000000000000000000000000000000000000000000000000000000000000000000000000000000000000000000000000000000000000000000000000000000000000000000
0000000000000000000000000000000000000000000000000000000000000000000000000000000000000000000000000000000000000000000000000000000000000000000000000000000000000000000000000000000
0000000000000000000000000000000000000000000000000000000000000000000000000000000000000000000000000000000000000000000
>Q00108
MASIDVGETIGIEQVEPVKFRFVCHCGFCPPSFVTARGAGSVSLRVVVHYVPSPEREDPESPTRGVDEVDGACSEPPTPRPEPRFRAIEEMGKIVVLVSVCPLRPALQHRWVGAPARRHRSDSVARRARFEPWRGRASRPTVLTPASGDSDDVDTRTFGCGCGPGHPPVDCMCDR
QDWL
0000000000000000000000000000000000000000000000000000000000000000000000000000000000000000000000000000000000000000000000000000000000000000000000000000000000000000000000000000000
0000
0000000000000000000000000000000000000000000000000000000000000000000000000000000000000000000000000000000000000000000000000000000000000000000000000000000000000000000000000000000
0000
0000000000000000000000000000000000000000000000000000000000000000000000000000000000000000000000000000000000000000000000000000000000000000000000000000000000000000000000000000000
0000
0000000000000000000000000000000000000000000000000000000000000000000000000000000000000000000000000000000000000000000000000000000000000000000000000000000000000000000000000000000
0000
0000000000000000000000000000000000000000000000000000000000000000000000000000000000000000000000000000000000000000000000000000000000000000000000000000000000000000000000000000000
0000
0000000000000000000000000000000000000000000000000000000000000000000000000000000000000000000000000000000000000000000000000000000000000000000000000000000000000000000000000000000
0000
0000000000000000000000000000000000000000000000000000000000000000000000000000000000000000000000000000000000000000000000000000000000000000000000000000000000000000000000000000000
0000
>Q00127
MYIRINESRGESTSLDNRLISASRVPSVSSSARFLSTCAESSRLRVSTSLRSSSRSLFASVNSREIAAVPFVYTIAGMEDVIITLGIDDTVYFLEWAILSINDVLSLIKRPGVKFTTVDTSNTSTVTIAVPATMDPMERLFVWTEGYHGVFMDDLRKGATYEWLCVLASTAGVLS
PMDPPMPSLLDRFTDSDELEIFVHLGRNIEINGATALWDRTVRYAFVPPSIELRFKNVWNAILKGGGDPRSVMDRDAVTVCSTVGADALVVLPRGDGDDEVPVVAVRNADRYLEELSERSTNRTVTEILAAPTPPFPIPNFGIGVLTDRVKGLCEVFIIIKDSDRWMFDRYTFAL
NTCMIGNVISNGLGIYETTLAGLERTLQPPAPTSRPRFVCVVRDGSHTEAGQPVGALGKCQAKPRNLW
0000000000000000000000000000000000000000000000000000000000000000000000000000000000000000000000000000000000000000000000000000000000000000000000000000000000000000000000000000000
0000000000000000000000000000000000000000000000000000000000000000000000000000000000000000000000000000000000000000000000000000000000000000000000000000000000000000000000000000000
00000000000000000000000000000000000000000000000000000000000000000000
0000000000000000000000000000000000000000000000000000000000000000000000000000000000000000000000000000000000000000000000000000000000000000000000000000000000000000000000000000000
0000000000000000000000000000000000000000000000000000000000000000000000000000000000000000000000000000000000000000000000000000000000000000000000000000000000000000000000000000000
00000000000000000000000000000000000000000000000000000000000000000000
0000000000000000000000000000000000000000000000000000000000000000000000000000000000000000000000000000000000000000000000000000000000000000000000000000000000000000000000000000000
0000000000000000000000000000000000000000000000000000000000000000000000000000000000000000000000000000000000000000000000000000000000000000000000000000000000000000000000000000000
00000000000000000000000000000000000000000000000000000000000000000000
0000000000000000000000000000000000000000000000000000000000000000000000000000000000000000000000000000000000000000000000000000000000000000000000000000000000000000000000000000000
0000000000000000000000000000000000000000000000000000000000000000000000000000000000000000000000000000000000000000000000000000000000000000000000000000000000000000000000000000000
00000000000000000000000000000000000000000000000000000000000000000000
0000000000000000000000000000000000000000000000000000000000000000000000000000000000000000000000000000000000000000000000000000000000000000000000000000000000000000000000000000000
0000000000000000000000000000000000000000000000000000000000000000000000000000000000000000000000000000000000000000000000000000000000000000000000000000000000000000000000000000000
00000000000000000000000000000000000000000000000000000000000000000000

0000000000000000000000000000000000000000000000000000000000000000000000000000000000000000000000000000000000000000000000000000000000000000000000000000000000000000000000000000000
0000000000000000000000000000000000000000000000000000000000000000000000000000000000000000000000000000000000000000000000000000000000000000000000000000000000000000000000000000000
00000000000000000000000000000000000000000000000000000000000000000000
0000000000000000000000000000000000000000000000000000000000000000000000000000000000000000000000000000000000000000000000000000000000000000000000000000000000000000000000000000000
0000000000000000000000000000000000000000000000000000000000000000000000000000000000000000000000000000000000000000000000000000000000000000000000000000000000000000000000000000000
00000000000000000000000000000000000000000000000000000000000000000000
>Q09057
MNNPLVNQAAMVLPVFLLSACLGGGGSFDLDSVDTEAPRPAPKYQDVSSEKPQAQKDQGGYGFAMRLKRRNWYPGAEESEVKLNESDWEATGLPTKPKELPKRQKSVIEKVETDGDSDIYSSPYLTPSNHQNGSAGNGVNQPKNQATGHENFQYVYSGWFYKHAASEKDFSNKKI
KSGDDGYIFYHGEKPSRQLPASGKVIYKGVWHFVTDTKKGQDFREIIQPSKKQGDRYSGFSGDGSEEYSNKNESTLKDDHEGYGFTSNLEVDFGNKKLTGKLIRNNASLNNNTNNDKHTTQYYSLDAQITGNRFNGTATATDKKENETKLHPFVSDSSSLSGGFFGPQGEELGFR
FLSDDQKVAVVGSAKTKDKLENGAAASGSTGAAASGGAAGTSSENSKLTTVLDAVELTLNDKKIKNLDNFSNAAQLVVDGIMIPLLPKDSESGNTQADKGKNGGTEFTRKFEHTPESDKKDAQAGTQTNGAQTASNTAGDTNGKTKTYEVEVCCSNLNYLKYGMLTRKNSKSAMQ
AGGNSSQADAKTEQVEQSMFLQGERTDEKEIPTDQNVVYRGSWYGHIANGTSWSGNASDKEGGNRAEFTVNFADKKITGKLTAENRQAQTFTIEGMIQGNGFEGTAKTAESGFDLDQKNTTRTPKAYITDAKVKGGFYGPKAEELGGWFAYPGDKQTEKATATSSDGNSASSATV
VFGAKRQQPVQ
0000000000000000000000000000000000000000000000000000000000000000000000000000000000000000000000000000000000000000000000000000000000000000000000000000000000000000000000000000000
0000000000000000000000000000000000000000000000000000000000000000000000000000000000000000000000000000000000000000000000000000000000000000000000000000000000000000000000000000000
0000000000000000000000000000000000000000000000000000000000000000000000000000000000000000000000000000000000000000000000000000000000000000000000000000000000000000000000000000000
0000000000000000000000000000000000000000000000000000000000000000000000000000000000000000000000000000000000000000000000000000000000000000000000000000000000000000000000000000000
00000000000
0000000000000000000000000000000000000000000000000000000000000000000000000000000000000000000000000000000000000000000000000000000000000000000000000000000000000000000000000000000
0000000000000000000000000000000000000000000000000000000000000000000000000000000000000000000000000000000000000000000000000000000000000000000000000000000000000000000000000000000
0000000000000000000000000000000000000000000000000000000000000000000000000000000000000000000000000000000000000000000000000000000000000000000000000000000000000000000000000000000
0000000000000000000000000000000000000000000000000000000000000000000000000000000000000000000000000000000000000000000000000000000000000000000000000000000000000000000000000000000
00000000000
0000000000000000000000000000000000000000000000000000000000000000000000000000000000000000000000000000000000000000000000000000000000000000000000000000000000000000000000000000000
0000000000000000000000000000000000000000000000000000000000000000000000000000000000000000000000000000000000000000000000000000000000000000000000000000000000000000000000000000000
0000000000000000000000000000000000000000000000000000000000000000000000000000000000000000000000000000000000000000000000000000000000000000000000000000000000000000000000000000000
0000000000000000000000000000000000000000000000000000000000000000000000000000000000000000000000000000000000000000000000000000000000000000000000000000000000000000000000000000000
00000000000
0000000000000000000000000000000000000000000000000000000000000000000000000000000000000000000000000000000000000000000000000000000000000000000000000000000000000000000000000000000
0000000000000000000000000000000000000000000000000000000000000000000000000000000000000000000000000000000000000000000000000000000000000000000000000000000000000000000000000000000
0000000000000000000000000000000000000000000000000000000000000000000000000000000000000000000000000000000000000000000000000000000000000000000000000000000000000000000000000000000
0000000000000000000000000000000000000000000000000000000000000000000000000000000000000000000000000000000000000000000000000000000000000000000000000000000000000000000000000000000
00000000000
0000000000000000000000000000000000000000000000000000000000000000000000000000000000000000000000000000000000000000000000000000000000000000000000000000000000000000000000000000000
0000000000000000000000000000000000000000000000000000000000000000000000000000000000000000000000000000000000000000000000000000000000000000000000000000000000000000000000000000000
0000000000000000000000000000000000000000000000000000000000000000000000000000000000000000000000000000000000000000000000000000000000000000000000000000000000000000000000000000000
0000000000000000000000000000000000000000000000000000000000000000000000000000000000000000000000000000000000000000000000000000000000000000000000000000000000000000000000000000000
00000000000
0000000000000000000000000000000000000000000000000000000000000000000000000000000000000000000000000000000000000000000000000000000000000000000000000000000000000000000000000000000
0000000000000000000000000000000000000000000000000000000000000000000000000000000000000000000000000000000000000000000000000000000000000000000000000000000000000000000000000000000
0000000000000000000000000000000000000000000000000000000000000000000000000000000000000000000000000000000000000000000000000000000000000000000000000000000000000000000000000000000
0000000000000000000000000000000000000000000000000000000000000000000000000000000000000000000000000000000000000000000000000000000000000000000000000000000000000000000000000000000
00000000000
0000000000000000000000000000000000000000000000000000000000000000000000000000000000000000000000000000000000000000000000000000000000000000000000000000000000000000000000000000000
0000000000000000000000000000000000000000000000000000000000000000000000000000000000000000000000000000000000000000000000000000000000000000000000000000000000000000000000000000000
0000000000000000000000000000000000000000000000000000000000000000000000000000000000000000000000000000000000000000000000000000000000000000000000000000000000000000000000000000000
0000000000000000000000000000000000000000000000000000000000000000000000000000000000000000000000000000000000000000000000000000000000000000000000000000000000000000000000000000000
00000000000
>Q0E590
MIATLGNLIIPVIFVNYVASETYKLTQRAFDTNFSSTRYDLKNSISRSLYDNPCMGDCVNYTCVVNAFGELEICVTAVDTVRIRHPAAGVLQRDVKYCRGSCVNDTNAPWCIVDTSGSFDLCGPFDAGSKVDSLAYEFLEDRVSFTGRSYGCESGCERTTAPCRVGGTSLPVLCS
PIAFVLPPTTPTLKRRTKRNAFVDCFSRNTLLGNNVSSSFIKYLDMFNAEWIDYNVRGHQDDDRIRTIHYGHPYCRR
0000000000000000000000000000000000000000000000000000000000000000000000000000000000000000000000000000000000000000000000000000000000000000000000000000000000000000000000000000000
00000000000000000000000000000000000000000000000000000000000000000000000000000
0000000000000000000000000000000000000000000000000000000000000000000000000000000000000000000000000000000000000000000000000000000000000000000000000000000000000000000000000000000
00000000000000000000000000000000000000000000000000000000000000000000000000000
0000000000000000000000000000000000000000000000000000000000000000000000000000000000000000000000000000000000000000000000000000000000000000000000000000000000000000000000000000000
00000000000000000000000000000000000000000000000000000000000000000000000000000
0000000000000000000000000000000000000000000000000000000000000000000000000000000000000000000000000000000000000000000000000000000000000000000000000000000000000000000000000000000
00000000000000000000000000000000000000000000000000000000000000000000000000000
0000000000000000000000000000000000000000000000000000000000000000000000000000000000000000000000000000000000000000000000000000000000000000000000000000000000000000000000000000000
00000000000000000000000000000000000000000000000000000000000000000000000000000
0000000000000000000000000000000000000000000000000000000000000000000000000000000000000000000000000000000000000000000000000000000000000000000000000000000000000000000000000000000
00000000000000000000000000000000000000000000000000000000000000000000000000000
0000000000000000000000000000000000000000000000000000000000000000000000000000000000000000000000000000000000000000000000000000000000000000000000000000000000000000000000000000000
00000000000000000000000000000000000000000000000000000000000000000000000000000
>Q0WVZ1
MESIGDDDELRSKTVSLPRRISFTILLLLLLISLSTRVSGSLDKSGRRVIEARTGQDLIWVVQLSDLHFSVHHPERAIDFKNIVGPALALINPSLVLITGDLTDGKSKDMLTMKLNEDEWLEYESVMQDVVKRSGLNKSIFYDLRGNHDNFGVPSVGSSVDFFSKYSINGQMGRK
GNVNTITVETSERKHLFVGIDTTMHIGLRGPTNLFGHPTDELLSSLDSHLSQWDNQSAKPVAKISFGHFPLSFTALSHSQKSLKDVFLKHSISAYLCGHLHSRFGKNLKRHHHSGGISLSDNDLFQLNMRQSGAESTSNCSFGALPAAEFWEWEMGDWRKNRAMRIMAIDRGHVS
YVDLDFKSKPQKTIILPTFPLDSRFMSTSFARHKYECQHMISSSYDAIRAIVFSHSLVVEVVARVYDSSPGFDNLVMEAPMRKHGGDSSSSGATFFSLPWNYRAFEDPLPDRFWLQIEVTDIKGRLTLSEMRPFSINGLSSKVSWTWNEFRVMGCQWAALYYPILWPALYSLFLV

FLIPKCIIIVFKKQYTLKKFIAKKGPITLVLWILQDLCRMPVVWFGYMAYLFYLIFFPWFSGEVFADSGDRAYMTIMGWVVTSSGADRKHEYIGQPDVMVVVIPHVVFVVIPSVLVVCCLVAEREIYKDHIRTVSGKKEDDHDRGRKKRSQRRSLLFSNRRLFRKSVLLASLALY
WKHFKNCWALGRAYEMNVVHFPGYSLVVPLLLLYVICKTHKVP
0000000000000000000000000000000000000000000000000000000000000000000000000000000000000000000000000000000000000000000000000000000000000000000000000000000000000000000000000000000
0000000000000000000000000000000000000000000000000000000000000000000000000000000000000000000000000000000000000000000000000000000000000000000000000000000000000000000000000000000
0000000000000000000000000000000000000000000000000000000000000000000000000000000000000000000000000000000000000000000000000000000000000000000000000000000000000000000000000000000
0000000000000000000000000000000000000000000000000000000000000000000000000000000000000000000000000000000000000000000000000000000000000000000000000000000000000000000000000000000
0000000000000000000000000000000000000000000
0000000000000000000000000000000000000000000000000000000000000000000000000000000000000000000000000000000000000000000000000000000000000000000000000000000000000000000000000000000
0000000000000000000000000000000000000000000000000000000000000000000000000000000000000000000000000000000000000000000000000000000000000000000000000000000000000000000000000000000
0000000000000000000000000000000000000000000000000000000000000000000000000000000000000000000000000000000000000000000000000000000000000000000000000000000000000000000000000000000
0000000000000000000000000000000000000000000000000000000000000000000000000000000000000000000000000000000000000000000000000000000000000000000000000000000000000000000000000000000
0000000000000000000000000000000000000000000
0000000000000000000000000000000000000000000000000000000000000000000000000000000000000000000000000000000000000000000000000000000000000000000000000000000000000000000000000000000
0000000000000000000000000000000000000000000000000000000000000000000000000000000000000000000000000000000000000000000000000000000000000000000000000000000000000000000000000000000
0000000000000000000000000000000000000000000000000000000000000000000000000000000000000000000000000000000000000000000000000000000000000000000000000000000000000000000000000000000
0000000000000000000000000000000000000000000000000000000000000000000000000000000000000000000000000000000000000000000000000000000000000000000000000000000000000000000000000000000
0000000000000000000000000000000000000000000
0000000000000000000000000000000000000000000000000000000000000000000000000000000000000000000000000000000000000000000000000000000000000000000000000000000000000000000000000000000
0000000000000000000000000000000000000000000000000000000000000000000000000000000000000000000000000000000000000000000000000000000000000000000000000000000000000000000000000000000
0000000000000000000000000000000000000000000000000000000000000000000000000000000000000000000000000000000000000000000000000000000000000000000000000000000000000000000000000000000
0000000000000000000000000000000000000000000000000000000000000000000000000000000000000000000000000000000000000000000000000000000000000000000000000000000000000000000000000000000
0000000000000000000000000000000000000000000
0000000000000000000000000000000000000000000000000000000000000000000000000000000000000000000000000000000000000000000000000000000000000000000000000000000000000000000000000000000
0000000000000000000000000000000000000000000000000000000000000000000000000000000000000000000000000000000000000000000000000000000000000000000000000000000000000000000000000000000
0000000000000000000000000000000000000000000000000000000000000000000000000000000000000000000000000000000000000000000000000000000000000000000000000000000000000000000000000000000
0000000000000000000000000000000000000000000000000000000000000000000000000000000000000000000000000000000000000000000000000000000000000000000000000000000000000000000000000000000
0000000000000000000000000000000000000000000
0000000000000000000000000000000000000000000000000000000000000000000000000000000000000000000000000000000000000000000000000000000000000000000000000000000000000000000000000000000
0000000000000000000000000000000000000000000000000000000000000000000000000000000000000000000000000000000000000000000000000000000000000000000000000000000000000000000000000000000
0000000000000000000000000000000000000000000000000000000000000000000000000000000000000000000000000000000000000000000000000000000000000000000000000000000000000000000000000000000
0000000000000000000000000000000000000000000000000000000000000000000000000000000000000000000000000000000000000000000000000000000000000000000000000000000000000000000000000000000
0000000000000000000000000000000000000000000
0000000000000000000000000000000000000000000000000000000000000000000000000000000000000000000000000000000000000000000000000000000000000000000000000000000000000000000000000000000
0000000000000000000000000000000000000000000000000000000000000000000000000000000000000000000000000000000000000000000000000000000000000000000000000000000000000000000000000000000
0000000000000000000000000000000000000000000000000000000000000000000000000000000000000000000000000000000000000000000000000000000000000000000000000000000000000000000000000000000
0000000000000000000000000000000000000000000000000000000000000000000000000000000000000000000000000000000000000000000000000000000000000000000000000000000000000000000000000000000
0000000000000000000000000000000000000000000
>Q12373
MKLRAEDVLANGTSRHKVQIDMERQVQIAKDLLAQKKFLEAAKRCQQTLDSLPKDGLLPDPELFTIFAQAVYNMEVQNSGNLFGDALLAGDDGSGSESESEPESDVSNGEEGNENGQTEIPNSRMFQFDQEEEDLTGDVDSGDSEDSGEGSEEEEENVEKEEERLALHELANFSP
ANEHDDEIEDVSQLRKSGFHIYFENDLYENALDLLAQALMLLGRPTADGQSLTENSRLRIGDVYILMGDIEREAEMFSRAIHHYLKALGYYKTLKPAEQVTEKVIQAEFLVCDALRWVDQVPAKDKLKRFKHAKALLEKHMTTRPKDSELQQARLAQIQDDIDEVQENQQHGSKR
PLSQPTTSIGFPALEKPLGDFNDLSQLVKKKPRRH
0000000000000000000000000000000000000000000000000000000000000000000000000000000000000000000000000000000000000000000000000000000000000000000000000000000000000000000000000000000
0000000000000000000000000000000000000000000000000000000000000000000000000000000000000000000000000000000000000000000000000000000000000000000000000000000000000000000000000000000
00000000000000000000000000000000000
0000000000000000000000000000000000000000000000000000000000000000000000000000000000000000000000000000000000000000000000000000000000000000000000000000000000000000000000000000000
0000000000000000000000000000000000000000000000000000000000000000000000000000000000000000000000000000000000000000000000000000000000000000000000000000000000000000000000000000000
00000000000000000000000000000000000
0000000000000000000000000000000000000000000000000000000000000000000000000000000000000000000000000000000000000000000000000000000000000000000000000000000000000000000000000000000
0000000000000000000000000000000000000000000000000000000000000000000000000000000000000000000000000000000000000000000000000000000000000000000000000000000000000000000000000000000
00000000000000000000000000000000000
0000000000000000000000000000000000000000000000000000000000000000000000000000000000000000000000000000000000000000000000000000000000000000000000000000000000000000000000000000000
0000000000000000000000000000000000000000000000000000000000000000000000000000000000000000000000000000000000000000000000000000000000000000000000000000000000000000000000000000000
00000000000000000000000000000000000
0000000000000000000000000000000000000000000000000000000000000000000000000000000000000000000000000000000000000000000000000000000000000000000000000000000000000000000000000000000
0000000000000000000000000000000000000000000000000000000000000000000000000000000000000000000000000000000000000000000000000000000000000000000000000000000000000000000000000000000
00000000000000000000000000000000000
0000000000000000000000000000000000000000000000000000000000000000000000000000000000000000000000000000000000000000000000000000000000000000000000000000000000000000000000000000000
0000000000000000000000000000000000000000000000000000000000000000000000000000000000000000000000000000000000000000000000000000000000000000000000000000000000000000000000000000000
00000000000000000000000000000000000
0000000000000000000000000000000000000000000000000000000000000000000000000000000000000000000000000000000000000000000000000000000000000000000000000000000000000000000000000000000
0000000000000000000000000000000000000000000000000000000000000000000000000000000000000000000000000000000000000000000000000000000000000000000000000000000000000000000000000000000
00000000000000000000000000000000000
>Q20123
MVKFQIIARDFYHGFIDSFKGITFVRRIREEEAKEVKVEPPKPVERTVLMMRREKQGIFKRPPEPPKKKDSFLKKLWQIYAMNIGFLVLWQVCILILGLFFSFFDRTDLGHNIGYILIIPIFFASRIIQALWFSDISGACMRALKLPPPPVVPFSSMLAGTLISALHQIFFLIQG
MLSQYLPIPLITPVIVYLHMALLNSMYCFDYFFDGYNLSFLRRKDIFESHWPYFLGFGTPLALACSISSNMFVNSVIFALLFPFFIITSYPANWNRKYEEEIPKIAFCRISYMFTELVGKFVKSITPTNNPTAARNNAQN
0000000000000000000000000000000000000000000000000000000000000000000000000000000000000000000000000000000000000000000000000000000000000000000000000000000000000000000000000000000
00000000000000000000000000000000000000000000000000000000000000000000000000000000000000000000000000000000000000000000000000000000000000000000
0000000000000000000000000000000000000000000000000000000000000000000000000000000000000000000000000000000000000000000000000000000000000000000000000000000000000000000000000000000
00000000000000000000000000000000000000000000000000000000000000000000000000000000000000000000000000000000000000000000000000000000000000000000

0000000000000000000000000000000000000000000000000000000000000000000000000000000000000000000000000000000000000000000000000000000000000000000000000000000000000000000000000000000
00000000000000000000000000000000000000000000000000000000000000000000000000000000000000000000000000000000000000000000000000000000000000000000
0000000000000000000000000000000000000000000000000000000000000000000000000000000000000000000000000000000000000000000000000000000000000000000000000000000000000000000000000000000
00000000000000000000000000000000000000000000000000000000000000000000000000000000000000000000000000000000000000000000000000000000000000000000
0000000000000000000000000000000000000000000000000000000000000000000000000000000000000000000000000000000000000000000000000000000000000000000000000000000000000000000000000000000
00000000000000000000000000000000000000000000000000000000000000000000000000000000000000000000000000000000000000000000000000000000000000000000
0000000000000000000000000000000000000000000000000000000000000000000000000000000000000000000000000000000000000000000000000000000000000000000000000000000000000000000000000000000
00000000000000000000000000000000000000000000000000000000000000000000000000000000000000000000000000000000000000000000000000000000000000000000
0000000000000000000000000000000000000000000000000000000000000000000000000000000000000000000000000000000000000000000000000000000000000000000000000000000000000000000000000000000
00000000000000000000000000000000000000000000000000000000000000000000000000000000000000000000000000000000000000000000000000000000000000000000
>Q21551
MGASQSAEQEARPEVVRIDRNEIPEEYKTVGVSSDVVSRVNATRVAGNDGESDRLRQELAREREEKARLREDMAKLSQLQQRKTAGISAAPVSISGNDLEERKKIFDDTVERVQKQFFAYHRENVCQDNENEIVRCLQENPGRVLKCAPLTEAFEKCVGEFRQQVLKGN
0000000000000000000000000000000000000000000000000000000000000000000000000000000000000000000000000000000000000000000000000000000000000000000000000000000000000000000000000
0000000000000000000000000000000000000000000000000000000000000000000000000000000000000000000000000000000000000000000000000000000000000000000000000000000000000000000000000
0000000000000000000000000000000000000000000000000000000000000000000000000000000000000000000000000000000000000000000000000000000000000000000000000000000000000000000000000
0000000000000000000000000000000000000000000000000000000000000000000000000000000000000000000000000000000000000000000000000000000000000000000000000000000000000000000000000
0000000000000000000000000000000000000000000000000000000000000000000000000000000000000000000000000000000000000000000000000000000000000000000000000000000000000000000000000
0000000000000000000000000000000000000000000000000000000000000000000000000000000000000000000000000000000000000000000000000000000000000000000000000000000000000000000000000
0000000000000000000000000000000000000000000000000000000000000000000000000000000000000000000000000000000000000000000000000000000000000000000000000000000000000000000000000
>Q28647
METKLPPASTPTSPSSPGLSPVPPADKVDGFSRRSLRRARPRRSHSSSQFRYQSNQQELTPLPLLKDVPASELHDLLSRKLAQCGVMFDFLDCVADLKGKEVKRAALNELVECVGSTRGVLIEPVYPDIIRMISVNIFRTLPPSENPEFDPEEDEPNLEPSWPHLQLVYEFFLRF
LESPDFQPSVAKRYVDQKFVLMLLELFDSEDPREREYLKTILHRVYGKFLGLRAYIRKQCNHIFLRFIYEFEHFNGVAELLEILGSIINGFALPLKTEHKQFLVRVLIPLHSVKSLSVFHAQLAYCVVQFLEKDATLTEHVIRGLLKYWPKTCTQKEVMFLGEVEEILDVIEPSQ
FVKIQEPLFKQVARCVSSPHFQVAERALYFWNNEYILSLIEDNCHTVLPAVFGTLYQVSKEHWNQTIVSLIYNVLKTFMEMNGKLFDELTASYKLEKQQEQQKARERQELWQGLEELRLRRLQGTQGTQGAREAPLQRFVPQVAATGGQS
0000000000000000000000000000000000000000000000000000000000000000000000000000000000000000000000000000000000000000000000000000000000000000000000000000000000000000000000000000000
0000000000000000000000000000000000000000000000000000000000000000000000000000000000000000000000000000000000000000000000000000000000000000000000000000000000000000000000000000000
000000000000000000000000000000000000000000000000000000000000000000000000000000000000000000000000000000000000000000000000000000000000000000000000000000
0000000000000000000000000000000000000000000000000000000000000000000000000000000000000000000000000000000000000000000000000000000000000000000000000000000000000000000000000000000
0000000000000000000000000000000000000000000000000000000000000000000000000000000000000000000000000000000000000000000000000000000000000000000000000000000000000000000000000000000
000000000000000000000000000000000000000000000000000000000000000000000000000000000000000000000000000000000000000000000000000000000000000000000000000000
0000000000000000000000000000000000000000000000000000000000000000000000000000000000000000000000000000000000000000000000000000000000000000000000000000000000000000000000000000000
0000000000000000000000000000000000000000000000000000000000000000000000000000000000000000000000000000000000000000000000000000000000000000000000000000000000000000000000000000000
000000000000000000000000000000000000000000000000000000000000000000000000000000000000000000000000000000000000000000000000000000000000000000000000000000
0000000000000000000000000000000000000000000000000000000000000000000000000000000000000000000000000000000000000000000000000000000000000000000000000000000000000000000000000000000
0000000000000000000000000000000000000000000000000000000000000000000000000000000000000000000000000000000000000000000000000000000000000000000000000000000000000000000000000000000
000000000000000000000000000000000000000000000000000000000000000000000000000000000000000000000000000000000000000000000000000000000000000000000000000000
0000000000000000000000000000000000000000000000000000000000000000000000000000000000000000000000000000000000000000000000000000000000000000000000000000000000000000000000000000000
0000000000000000000000000000000000000000000000000000000000000000000000000000000000000000000000000000000000000000000000000000000000000000000000000000000000000000000000000000000
000000000000000000000000000000000000000000000000000000000000000000000000000000000000000000000000000000000000000000000000000000000000000000000000000000
0000000000000000000000000000000000000000000000000000000000000000000000000000000000000000000000000000000000000000000000000000000000000000000000000000000000000000000000000000000
0000000000000000000000000000000000000000000000000000000000000000000000000000000000000000000000000000000000000000000000000000000000000000000000000000000000000000000000000000000
000000000000000000000000000000000000000000000000000000000000000000000000000000000000000000000000000000000000000000000000000000000000000000000000000000
0000000000000000000000000000000000000000000000000000000000000000000000000000000000000000000000000000000000000000000000000000000000000000000000000000000000000000000000000000000
0000000000000000000000000000000000000000000000000000000000000000000000000000000000000000000000000000000000000000000000000000000000000000000000000000000000000000000000000000000
000000000000000000000000000000000000000000000000000000000000000000000000000000000000000000000000000000000000000000000000000000000000000000000000000000
>Q2M3C7
MDGNSLLSVPSNLESSRMYDVLEPQQGRGCGSSGSGPGNSITACKKVLRSNSLLESTDYWLQNQRMPCQIGFVEDKSENCASVCFVNLDVNKDECSTEHLQQKLVNVSPDLPKLISSMNVQQPKENEIVVLSGLASGNLQADFEVSQCPWLPDICLVQCARGNRPNSTNCIIFEI
NKFLIGLELVQERQLHLETNILKLEDDTNCSLSSIEEDFLTASEHLEEESEVDESRNDYENINVSANVLESKQLKGATQVEWNCNKEKWLYALEDKYINKYPTPLIKTERSPENLTKNTALQSLDPSAKPSQWKREAVGNGRQATHYYHSEAFKGQMEKSQALYIPKDAYFSMMD
KDVPSACAVAEQRSNLNPGDHEDTRNALPPRQDGEVTTGKYATNLAESVLQDAFIRLSQSQSTLPQESAVSVSVGSSLLPSCYSTKDTVVSRSWNELPKIVVVQSPDGSDAAPQPGISSWPEMEVSVETSSILSGENSSRQPQSALEVALACAATVIGTISSPQATERLKMEQVV
SNFPPGSSGALQTQAPQGLKEPSINEYSFPSALCGMTQVASAVAVCGLGEREEVTCSVAPSGSLPPAAEASEAMPPLCGLASMELGKEAIAKGLLKEAALVLTRPNTYSSIGDFLDSMNRRIMETASKSQTLCSENVVRNELAHTLSNVILRHSIDEVHHKNMIIDPNDNRHSSE
ILDTLMESTNQLLLDVICFTFKKMSHIVRLGECPAVLSKETIRRRETEPSCQPSDPGASQAWTKATESSSSSPLSNSHNTSLVINNLVDGMYSKQDKGGVRPGLFKNPTLQSQLSRSHRVPDSSTATTSSKEIYLKGIAGEDTKSPHHSENECRASSEGQRSPTVSQSRSGSQEA
EESIHPNTQEKYNCATSRINEVQVNLSLLGDDLLLPAQSTLQTKHPDIYCITDFAEELADTVVSMATEIAAICLDNSSGKQPWFCAWKRGSEFLMTPNVPCRSLKRKKESQGSGTAVRKHKPPRLSEIKRKTDEHPELKEKLMNRVVDESMNLEDVPDSVNLFANEVAAKIMNLT
EFSMVDGMWQAQGYPRNRLLSGDRWSRLKASSCESIPEEDSEARAYVNSLGLMSTLSQPVSRASSVSKQSSCESITDEFSRFMVNQMENEGRGFELLLDYYAGKNASSILNSAMQQACRKSDHLSVRPSCPSKQSSTESITEEFYRYMLRDIERDSRESASSRRSSQDWTAGLLS
PSLRSPVCHRQSSMPDSRSPCSRLTVNVPIKANSLDGFAQNCPQDFLSVQPVSSASSSGLCKSDSCLYRRGGTDHITNMLIHETWASSIEALMRKNKIIVDDAEEADTEPVSGGSPSQAEKCANRLAASRMCSGPTLLVQESLDCPRKDSVTECKQPPVSSLSKTASLTNHSPLD
SKKETSSCQDPVPINHKRRSLCSREVPLIQIETDQREACAGEPEPFLSKSSLLEEAEGHSNDKNIPDVVRGGDTAVSACQIHSDSLDTRDVPEAEASTEARAPDEAPNPPSSSEESTGSWTQLANEEDNPDDTSSFLQLSERSMSNGNSSATSSLGIMDLDIYQESMPSSPMINE
LVEEKKILKGQSESTEAPASGPPTGTASPQRSLLVINFDLEPECPDAELRATLQWIAASELGIPTIYFKKSQENRIEKFLDVVQLVHRKSWKVGDIFHAVVQYCKMHEEQKDGRLSLFDWLLELG
0000000000000000000000000000000000000000000000000000000000000000000000000000000000000000000000000000000000000000000000000000000000000000000000000000000000000000000000000000000
0000000000000000000000000000000000000000000000000000000000000000000000000000000000000000000000000000000000000000000000000000000000000000000000000000000000000000000000000000000
0000000000000000000000000000000000000000000000000000000000000000000000000000000000000000000000000000000000000000000000000000000000000000000000000000000000000000000000000000000
0000000000000000000000000000000000000000000000000000000000000000000000000000000000000000000000000000000000000000000000000000000000000000000000000000000000000000000000000000000
0000000000000000000000000000000000000000000000000000000000000000000000000000000000000000000000000000000000000000000000000000000000000000000000000000000000000000000000000000000
0000000000000000000000000000000000000000000000000000000000000000000000000000000000000000000000000000000000000000000000000000000000000000000000000000000000000000000000000000000
0000000000000000000000000000000000000000000000000000000000000000000000000000000000000000000000000000000000000000000000000000000000000000000000000000000000000000000000000000000
0000000000000000000000000000000000000000000000000000000000000000000000000000000000000000000000000000000000000000000000000000000000000000000000000000000000000000000000000000000
0000000000000000000000000000000000000000000000000000000000000000000000000000000000000000000000000000000000000000000000000000000000000000000000000000000000000000000000000000000
00000000000000000000000000000000000000000000000000000000000000000000000000000000000000000000000000000000000000000000000000000
0000000000000000000000000000000000000000000000000000000000000000000000000000000000000000000000000000000000000000000000000000000000000000000000000000000000000000000000000000000
0000000000000000000000000000000000000000000000000000000000000000000000000000000000000000000000000000000000000000000000000000000000000000000000000000000000000000000000000000000
0000000000000000000000000000000000000000000000000000000000000000000000000000000000000000000000000000000000000000000000000000000000000000000000000000000000000000000000000000000
0000000000000000000000000000000000000000000000000000000000000000000000000000000000000000000000000000000000000000000000000000000000000000000000000000000000000000000000000000000

0000000000000000000000000000000000000000000000000000000000000000000000000000000000000000000000000000000000000000000000000000000000000000000000000000000000000000000000000000000
0000000000000000000000000000000000000000000000000000000000000000000000000000000000000000000000000000000000000000000000000000000000000000000000000000000000000000000000000000000
0000000000000000000000000000000000000000000000000000000000000000000000000000000000000000000000000000000000000000000000000000000000000000000000000000000000000000000000000000000
0000000000000000000000000000000000000000000000000000000000000000000000000000000000000000000000000000000000000000000000000000000000000000000000000000000000000000000000000000000
0000000000000000000000000000000000000000000000000000000000000000000000000000000000000000000000000000000000000000000000000000000000000000000000000000000000000000000000000000000
00000000000000000000000000000000000000000000000000000000000000000000000000000000000000000000000000000000000000000000000000000
0000000000000000000000000000000000000000000000000000000000000000000000000000000000000000000000000000000000000000000000000000000000000000000000000000000000000000000000000000000
0000000000000000000000000000000000000000000000000000000000000000000000000000000000000000000000000000000000000000000000000000000000000000000000000000000000000000000000000000000
0000000000000000000000000000000000000000000000000000000000000000000000000000000000000000000000000000000000000000000000000000000000000000000000000000000000000000000000000000000
0000000000000000000000000000000000000000000000000000000000000000000000000000000000000000000000000000000000000000000000000000000000000000000000000000000000000000000000000000000
0000000000000000000000000000000000000000000000000000000000000000000000000000000000000000000000000000000000000000000000000000000000000000000000000000000000000000000000000000000
0000000000000000000000000000000000000000000000000000000000000000000000000000000000000000000000000000000000000000000000000000000000000000000000000000000000000000000000000000000
0000000000000000000000000000000000000000000000000000000000000000000000000000000000000000000000000000000000000000000000000000000000000000000000000000000000000000000000000000000
0000000000000000000000000000000000000000000000000000000000000000000000000000000000000000000000000000000000000000000000000000000000000000000000000000000000000000000000000000000
0000000000000000000000000000000000000000000000000000000000000000000000000000000000000000000000000000000000000000000000000000000000000000000000000000000000000000000000000000000
00000000000000000000000000000000000000000000000000000000000000000000000000000000000000000000000000000000000000000000000000000
0000000000000000000000000000000000000000000000000000000000000000000000000000000000000000000000000000000000000000000000000000000000000000000000000000000000000000000000000000000
0000000000000000000000000000000000000000000000000000000000000000000000000000000000000000000000000000000000000000000000000000000000000000000000000000000000000000000000000000000
0000000000000000000000000000000000000000000000000000000000000000000000000000000000000000000000000000000000000000000000000000000000000000000000000000000000000000000000000000000
0000000000000000000000000000000000000000000000000000000000000000000000000000000000000000000000000000000000000000000000000000000000000000000000000000000000000000000000000000000
0000000000000000000000000000000000000000000000000000000000000000000000000000000000000000000000000000000000000000000000000000000000000000000000000000000000000000000000000000000
0000000000000000000000000000000000000000000000000000000000000000000000000000000000000000000000000000000000000000000000000000000000000000000000000000000000000000000000000000000
0000000000000000000000000000000000000000000000000000000000000000000000000000000000000000000000000000000000000000000000000000000000000000000000000000000000000000000000000000000
0000000000000000000000000000000000000000000000000000000000000000000000000000000000000000000000000000000000000000000000000000000000000000000000000000000000000000000000000000000
0000000000000000000000000000000000000000000000000000000000000000000000000000000000000000000000000000000000000000000000000000000000000000000000000000000000000000000000000000000
00000000000000000000000000000000000000000000000000000000000000000000000000000000000000000000000000000000000000000000000000000
0000000000000000000000000000000000000000000000000000000000000000000000000000000000000000000000000000000000000000000000000000000000000000000000000000000000000000000000000000000
0000000000000000000000000000000000000000000000000000000000000000000000000000000000000000000000000000000000000000000000000000000000000000000000000000000000000000000000000000000
0000000000000000000000000000000000000000000000000000000000000000000000000000000000000000000000000000000000000000000000000000000000000000000000000000000000000000000000000000000
0000000000000000000000000000000000000000000000000000000000000000000000000000000000000000000000000000000000000000000000000000000000000000000000000000000000000000000000000000000
0000000000000000000000000000000000000000000000000000000000000000000000000000000000000000000000000000000000000000000000000000000000000000000000000000000000000000000000000000000
0000000000000000000000000000000000000000000000000000000000000000000000000000000000000000000000000000000000000000000000000000000000000000000000000000000000000000000000000000000
0000000000000000000000000000000000000000000000000000000000000000000000000000000000000000000000000000000000000000000000000000000000000000000000000000000000000000000000000000000
0000000000000000000000000000000000000000000000000000000000000000000000000000000000000000000000000000000000000000000000000000000000000000000000000000000000000000000000000000000
0000000000000000000000000000000000000000000000000000000000000000000000000000000000000000000000000000000000000000000000000000000000000000000000000000000000000000000000000000000
00000000000000000000000000000000000000000000000000000000000000000000000000000000000000000000000000000000000000000000000000000
0000000000000000000000000000000000000000000000000000000000000000000000000000000000000000000000000000000000000000000000000000000000000000000000000000000000000000000000000000000
0000000000000000000000000000000000000000000000000000000000000000000000000000000000000000000000000000000000000000000000000000000000000000000000000000000000000000000000000000000
0000000000000000000000000000000000000000000000000000000000000000000000000000000000000000000000000000000000000000000000000000000000000000000000000000000000000000000000000000000
0000000000000000000000000000000000000000000000000000000000000000000000000000000000000000000000000000000000000000000000000000000000000000000000000000000000000000000000000000000
0000000000000000000000000000000000000000000000000000000000000000000000000000000000000000000000000000000000000000000000000000000000000000000000000000000000000000000000000000000
0000000000000000000000000000000000000000000000000000000000000000000000000000000000000000000000000000000000000000000000000000000000000000000000000000000000000000000000000000000
0000000000000000000000000000000000000000000000000000000000000000000000000000000000000000000000000000000000000000000000000000000000000000000000000000000000000000000000000000000
0000000000000000000000000000000000000000000000000000000000000000000000000000000000000000000000000000000000000000000000000000000000000000000000000000000000000000000000000000000
0000000000000000000000000000000000000000000000000000000000000000000000000000000000000000000000000000000000000000000000000000000000000000000000000000000000000000000000000000000
00000000000000000000000000000000000000000000000000000000000000000000000000000000000000000000000000000000000000000000000000000
0000000000000000000000000000000000000000000000000000000000000000000000000000000000000000000000000000000000000000000000000000000000000000000000000000000000000000000000000000000
0000000000000000000000000000000000000000000000000000000000000000000000000000000000000000000000000000000000000000000000000000000000000000000000000000000000000000000000000000000
0000000000000000000000000000000000000000000000000000000000000000000000000000000000000000000000000000000000000000000000000000000000000000000000000000000000000000000000000000000
0000000000000000000000000000000000000000000000000000000000000000000000000000000000000000000000000000000000000000000000000000000000000000000000000000000000000000000000000000000
0000000000000000000000000000000000000000000000000000000000000000000000000000000000000000000000000000000000000000000000000000000000000000000000000000000000000000000000000000000
0000000000000000000000000000000000000000000000000000000000000000000000000000000000000000000000000000000000000000000000000000000000000000000000000000000000000000000000000000000
0000000000000000000000000000000000000000000000000000000000000000000000000000000000000000000000000000000000000000000000000000000000000000000000000000000000000000000000000000000
0000000000000000000000000000000000000000000000000000000000000000000000000000000000000000000000000000000000000000000000000000000000000000000000000000000000000000000000000000000
0000000000000000000000000000000000000000000000000000000000000000000000000000000000000000000000000000000000000000000000000000000000000000000000000000000000000000000000000000000
00000000000000000000000000000000000000000000000000000000000000000000000000000000000000000000000000000000000000000000000000000
>Q30KK6
MKLLLLLLTVTLLLAQVTPVMKCWGKSGRCRTTCKESEVYYILCKTEAKCCVDPKYVPVKPKLTDRNTSLESTSAV
0000000000000000000000000000000000000000000000000000000000000000000000000000
0000000000000000000000000000000000000000000000000000000000000000000000000000
0000000000000000000000000000000000000000000000000000000000000000000000000000
0000000000000000000000000000000000000000000000000000000000000000000000000000
0000000000000000000000000000000000000000000000000000000000000000000000000000
0000000000000000000000000000000000000000000000000000000000000000000000000000
0000000000000000000000000000000000000000000000000000000000000000000000000000
>Q3A947
MADKIKLEVVTPERVVANEHVDFVVAPGVEGEIGILPFHAPLITSLDIGILRYTVEGKTEKIALSGGFLEVKGNKVVVLANAAERGEEIDVERAQRALERARERLARRTPDIDVLRAELAMRRALNRLKAAGKM
00000000000000000000000000000000000000000000000000000000000000000000000000000000000000000000000000000000000000000000000000000000000000
00000000000000000000000000000000000000000000000000000000000000000000000000000000000000000000000000000000000000000000000000000000000000

00000000000000000000000000000000000000000000000000000000000000000000000000000000000000000000000000000000000000000000000000000000000000
00000000000000000000000000000000000000000000000000000000000000000000000000000000000000000000000000000000000000000000000000000000000000
00000000000000000000000000000000000000000000000000000000000000000000000000000000000000000000000000000000000000000000000000000000000000
00000000000000000000000000000000000000000000000000000000000000000000000000000000000000000000000000000000000000000000000000000000000000
00000000000000000000000000000000000000000000000000000000000000000000000000000000000000000000000000000000000000000000000000000000000000
>Q3T4N8
MTNEEKLIAEIDSAGLSAEMLESMPSPEMFPLIIVNLQGRIAELEARETEMLARVDTLIARLAHLGVA
00000000000000000000000000000000000000000000000000000000000000000000
00000000000000000000000000000000000000000000000000000000000000000000
00000000000000000000000000000000000000000000000000000000000000000000
00000000000000000000000000000000000000000000000000000000000000000000
00000000000000000000000000000000000000000000000000000000000000000000
00000000000000000000000000000000000000000000000000000000000000000000
00000000000000000000000000000000000000000000000000000000000000000000
>Q3V4U5
MAQVENVSLSQIIANPYLPPFSTTLFEIVIFTFIMITLYLIFRGSGLVRRRLVLLLIAIYVIVLQLFFTPYEYTTARLLPNGTVDYVVYTSQANIVMALDVLAGLMLILAVVYIILDYLRGFGKGDEEEGVIDL
00000000000000000000000000000000000000000000000000000000000000000000000000000000000000000000000000000000000000000000000000000000000000
00000000000000000000000000000000000000000000000000000000000000000000000000000000000000000000000000000000000000000000000000000000000000
00000000000000000000000000000000000000000000000000000000000000000000000000000000000000000000000000000000000000000000000000000000000000
00000000000000000000000000000000000000000000000000000000000000000000000000000000000000000000000000000000000000000000000000000000000000
00000000000000000000000000000000000000000000000000000000000000000000000000000000000000000000000000000000000000000000000000000000000000
00000000000000000000000000000000000000000000000000000000000000000000000000000000000000000000000000000000000000000000000000000000000000
00000000000000000000000000000000000000000000000000000000000000000000000000000000000000000000000000000000000000000000000000000000000000
>Q42456
MGTRSVALVLLAAVLLQALLPASAAEGLVRIALKKRPIDENSRVAARLSGEEGARRLGLRGANSLGGGGGEGDIVALKNYMNAQYFGEIGVGTPPQKFTVIFDTGSSNLWVPSAKCYFSIACFFHSRYKSGQSSTYQKNGKPAAIQYGTGSIAGFFSEDSVTVGDLVVKDQEFIE
ATKEPGLTFMVAKFDGILGLGFQEISVGDAVPVWYKMVEQGLVSEPVFSFWFNRHSDEGEGGEIVFGGMDPSHYKGNHTYVPVSQKGYWQFEMGDVLIGGKTTGFCASGCSAIADSGTSLLAGPTAIITEINEKIGATGVVSQECKTVVSQYGQQILDLLLAETQPSKICSQVGL
CTFDGKHGVSAGIKSVVDDEAGESNGLQSGPMCNACEMAVVWMQNQLAQNKTQDLILNYINQLCDKLPSPMGESSVDCGSLASMPEISFTIGGKKFALKPEEYILKVGEGAAAQCISGFTAMDIPPPRGPLWILGDVFMGAYHTVFDYGKMRVGFAKSA
0000000000000000000000000000000000000000000000000000000000000000000000000000000000000000000000000000000000000000000000000000000000000000000000000000000000000000000000000000000
0000000000000000000000000000000000000000000000000000000000000000000000000000000000000000000000000000000000000000000000000000000000000000000000000000000000000000000000000000000
000000000000000000000000000000000000000000000000000000000000000000000000000000000000000000000000000000000000000000000000000000000000000000000000000000000000000
0000000000000000000000000000000000000000000000000000000000000000000000000000000000000000000000000000000000000000000000000000000000000000000000000000000000000000000000000000000
0000000000000000000000000000000000000000000000000000000000000000000000000000000000000000000000000000000000000000000000000000000000000000000000000000000000000000000000000000000
000000000000000000000000000000000000000000000000000000000000000000000000000000000000000000000000000000000000000000000000000000000000000000000000000000000000000
0000000000000000000000000000000000000000000000000000000000000000000000000000000000000000000000000000000000000000000000000000000000000000000000000000000000000000000000000000000
0000000000000000000000000000000000000000000000000000000000000000000000000000000000000000000000000000000000000000000000000000000000000000000000000000000000000000000000000000000
000000000000000000000000000000000000000000000000000000000000000000000000000000000000000000000000000000000000000000000000000000000000000000000000000000000000000
0000000000000000000000000000000000000000000000000000000000000000000000000000000000000000000000000000000000000000000000000000000000000000000000000000000000000000000000000000000
0000000000000000000000000000000000000000000000000000000000000000000000000000000000000000000000000000000000000000000000000000000000000000000000000000000000000000000000000000000
000000000000000000000000000000000000000000000000000000000000000000000000000000000000000000000000000000000000000000000000000000000000000000000000000000000000000
0000000000000000000000000000000000000000000000000000000000000000000000000000000000000000000000000000000000000000000000000000000000000000000000000000000000000000000000000000000
0000000000000000000000000000000000000000000000000000000000000000000000000000000000000000000000000000000000000000000000000000000000000000000000000000000000000000000000000000000
000000000000000000000000000000000000000000000000000000000000000000000000000000000000000000000000000000000000000000000000000000000000000000000000000000000000000
0000000000000000000000000000000000000000000000000000000000000000000000000000000000000000000000000000000000000000000000000000000000000000000000000000000000000000000000000000000
0000000000000000000000000000000000000000000000000000000000000000000000000000000000000000000000000000000000000000000000000000000000000000000000000000000000000000000000000000000
000000000000000000000000000000000000000000000000000000000000000000000000000000000000000000000000000000000000000000000000000000000000000000000000000000000000000
0000000000000000000000000000000000000000000000000000000000000000000000000000000000000000000000000000000000000000000000000000000000000000000000000000000000000000000000000000000
0000000000000000000000000000000000000000000000000000000000000000000000000000000000000000000000000000000000000000000000000000000000000000000000000000000000000000000000000000000
000000000000000000000000000000000000000000000000000000000000000000000000000000000000000000000000000000000000000000000000000000000000000000000000000000000000000
>Q49LS9
MAAKSDGVLKMKKSDVAFTPLQNSEHSGSVQGLHPGAQPDSAGAGDADFANGESRCCGGGGSHSTCLRLSREQQKYTVWDCLWIVAAVAVYVADVGSDVWLSVDYYLEEDYWWFGLTLFFVVLGSFSVQLFSFRWFVHDFSTEDSAEAAADGSHMDGNKLLSGSASHGDVTAQHH
PATPQRQASTASRNTTTNSTASTGLGPRGPKRPAYYTFCVWGSQSVIHILQLGQIWRYIHTIYLGVQSRQSAETERWRYYWRMVYEFADVSMLHLLATFLESAPQLVLQLCIIIQTHKLLAVQGCRLFIYYLLILAENAALSALWYLYRSPLATDAFAVPALCVIFSSFLTGVVF
MLMYYAFFHPNGPRFGRSMSGHGLNLDPTAQFSTLPSEVATNSLRSNRGATATLERDAGKYSERDGCMPVFQVRPTVPSTPSSRAPRLEETVIKIDLCRNRYPAWERHVLDRSIRKAILAVDCSLTPPRLQYKDDALVQERLEYETTL
0000000000000000000000000000000000000000000000000000000000000000000000000000000000000000000000000000000000000000000000000000000000000000000000000000000000000000000000000000000
0000000000000000000000000000000000000000000000000000000000000000000000000000000000000000000000000000000000000000000000000000000000000000000000000000000000000000000000000000000
0000000000000000000000000000000000000000000000000000000000000000000000000000000000000000000000000000000000000000000000000000000000000000000000000000
0000000000000000000000000000000000000000000000000000000000000000000000000000000000000000000000000000000000000000000000000000000000000000000000000000000000000000000000000000000
0000000000000000000000000000000000000000000000000000000000000000000000000000000000000000000000000000000000000000000000000000000000000000000000000000000000000000000000000000000
0000000000000000000000000000000000000000000000000000000000000000000000000000000000000000000000000000000000000000000000000000000000000000000000000000
0000000000000000000000000000000000000000000000000000000000000000000000000000000000000000000000000000000000000000000000000000000000000000000000000000000000000000000000000000000
0000000000000000000000000000000000000000000000000000000000000000000000000000000000000000000000000000000000000000000000000000000000000000000000000000000000000000000000000000000
0000000000000000000000000000000000000000000000000000000000000000000000000000000000000000000000000000000000000000000000000000000000000000000000000000
0000000000000000000000000000000000000000000000000000000000000000000000000000000000000000000000000000000000000000000000000000000000000000000000000000000000000000000000000000000
0000000000000000000000000000000000000000000000000000000000000000000000000000000000000000000000000000000000000000000000000000000000000000000000000000000000000000000000000000000
0000000000000000000000000000000000000000000000000000000000000000000000000000000000000000000000000000000000000000000000000000000000000000000000000000
0000000000000000000000000000000000000000000000000000000000000000000000000000000000000000000000000000000000000000000000000000000000000000000000000000000000000000000000000000000
0000000000000000000000000000000000000000000000000000000000000000000000000000000000000000000000000000000000000000000000000000000000000000000000000000000000000000000000000000000
0000000000000000000000000000000000000000000000000000000000000000000000000000000000000000000000000000000000000000000000000000000000000000000000000000

000000000000000000000000000000000000000000000000000000000000000000000000000000000000000000000000000000000000000000000000000000000000000000000000000000000000000000000000000000000000
000000000000000000000000000000000000000000000000000000000000000000000000000000000000000000000000000000000000000000000000000000000000000000000000000000000000000000000000000000000000
00000000000000000000000000000000000000000000000000000000000000000000000000000000000000000000000000000000000000000000000000000000000000000000000000000000
000000000000000000000000000000000000000000000000000000000000000000000000000000000000000000000000000000000000000000000000000000000000000000000000000000000000000000000000000000000000
000000000000000000000000000000000000000000000000000000000000000000000000000000000000000000000000000000000000000000000000000000000000000000000000000000000000000000000000000000000000
00000000000000000000000000000000000000000000000000000000000000000000000000000000000000000000000000000000000000000000000000000000000000000000000000000000
>Q4G163
MSFKDKDERISCLEAYVTLTSKSSRFTDETEILKMSQRHSGQAGTEAGNGADSPPIVNSKYSTFRDFCSTSSFQDSGYNELKSCSFDNIDKEYLGKKEKGPTLLYEHPETSGLGLTHPLESPTQKKKCILPRKEKDKTPELCETPKISGKKCLPRRRLNVSFALLKGDFESQNSS
LESSISQVINLEKNIPSSASGFSRANNFSPLVTSTLKTEEVTSCSQKLRLNFSQQKTSTIDDSKDDCSLFEVECISPIQGNNFKDSITHDFSDSSLCINDENACPELLGSSVSGTTCGTDEDIFVTPISNLVANIRFNASQILSPSPEVRGSISTPEDSGFNSLSLEKSEDSLSD
QEGSFQELLQKHKGTPKVGDTIRKTRHLGRSRRLSTLREQSSQSETEEEKQIVHPDSEKRAAAASAISEGQLSSDESGDLTFSLKNLSKTPALQLVHELFMKSKRKRLQENSGHEFLEQGDGEKIAVLQCILAGLIGKKMGIEKLDILTELKYRNLKHILAMVLESLTAESLCSV
WKVSRNWREIVVQDKNANRRRKFYITQLKTDSEGAVLNVEDAATRLQLLNRSALRSVQAQARIPGSQREQGSTLSPWGEVLTPLASSSVTHLSSKQEEYVKVAKTLFTDEALKPCPRCQSPAKYQPYKKRGLCSRTACGFDFCVLCLCAYHGSEECSRGAAKPRNRKDALPGSAQ
SKRNLKRL
000000000000000000000000000000000000000000000000000000000000000000000000000000000000000000000000000000000000000000000000000000000000000000000000000000000000000000000000000000000000
000000000000000000000000000000000000000000000000000000000000000000000000000000000000000000000000000000000000000000000000000000000000000000000000000000000000000000000000000000000000
000000000000000000000000000000000000000000000000000000000000000000000000000000000000000000000000000000000000000000000000000000000000000000000000000000000000000000000000000000000000
000000000000000000000000000000000000000000000000000000000000000000000000000000000000000000000000000000000000000000000000000000000000000000000000000000000000000000000000000000000000
00000000
000000000000000000000000000000000000000000000000000000000000000000000000000000000000000000000000000000000000000000000000000000000000000000000000000000000000000000000000000000000000
000000000000000000000000000000000000000000000000000000000000000000000000000000000000000000000000000000000000000000000000000000000000000000000000000000000000000000000000000000000000
000000000000000000000000000000000000000000000000000000000000000000000000000000000000000000000000000000000000000000000000000000000000000000000000000000000000000000000000000000000000
000000000000000000000000000000000000000000000000000000000000000000000000000000000000000000000000000000000000000000000000000000000000000000000000000000000000000000000000000000000000
00000000
000000000000000000000000000000000000000000000000000000000000000000000000000000000000000000000000000000000000000000000000000000000000000000000000000000000000000000000000000000000000
000000000000000000000000000000000000000000000000000000000000000000000000000000000000000000000000000000000000000000000000000000000000000000000000000000000000000000000000000000000000
000000000000000000000000000000000000000000000000000000000000000000000000000000000000000000000000000000000000000000000000000000000000000000000000000000000000000000000000000000000000
000000000000000000000000000000000000000000000000000000000000000000000000000000000000000000000000000000000000000000000000000000000000000000000000000000000000000000000000000000000000
00000000
000000000000000000000000000000000000000000000000000000000000000000000000000000000000000000000000000000000000000000000000000000000000000000000000000000000000000000000000000000000000
000000000000000000000000000000000000000000000000000000000000000000000000000000000000000000000000000000000000000000000000000000000000000000000000000000000000000000000000000000000000
000000000000000000000000000000000000000000000000000000000000000000000000000000000000000000000000000000000000000000000000000000000000000000000000000000000000000000000000000000000000
000000000000000000000000000000000000000000000000000000000000000000000000000000000000000000000000000000000000000000000000000000000000000000000000000000000000000000000000000000000000
00000000
000000000000000000000000000000000000000000000000000000000000000000000000000000000000000000000000000000000000000000000000000000000000000000000000000000000000000000000000000000000000
000000000000000000000000000000000000000000000000000000000000000000000000000000000000000000000000000000000000000000000000000000000000000000000000000000000000000000000000000000000000
000000000000000000000000000000000000000000000000000000000000000000000000000000000000000000000000000000000000000000000000000000000000000000000000000000000000000000000000000000000000
000000000000000000000000000000000000000000000000000000000000000000000000000000000000000000000000000000000000000000000000000000000000000000000000000000000000000000000000000000000000
00000000
000000000000000000000000000000000000000000000000000000000000000000000000000000000000000000000000000000000000000000000000000000000000000000000000000000000000000000000000000000000000
000000000000000000000000000000000000000000000000000000000000000000000000000000000000000000000000000000000000000000000000000000000000000000000000000000000000000000000000000000000000
000000000000000000000000000000000000000000000000000000000000000000000000000000000000000000000000000000000000000000000000000000000000000000000000000000000000000000000000000000000000
000000000000000000000000000000000000000000000000000000000000000000000000000000000000000000000000000000000000000000000000000000000000000000000000000000000000000000000000000000000000
00000000
000000000000000000000000000000000000000000000000000000000000000000000000000000000000000000000000000000000000000000000000000000000000000000000000000000000000000000000000000000000000
000000000000000000000000000000000000000000000000000000000000000000000000000000000000000000000000000000000000000000000000000000000000000000000000000000000000000000000000000000000000
000000000000000000000000000000000000000000000000000000000000000000000000000000000000000000000000000000000000000000000000000000000000000000000000000000000000000000000000000000000000
000000000000000000000000000000000000000000000000000000000000000000000000000000000000000000000000000000000000000000000000000000000000000000000000000000000000000000000000000000000000
00000000
>Q52KW8
MPRKKVTDGSGPGNGAARPANRKKAAASGKKRSRHEFSSDEDDLDGSPGSDGGFQGHNNKRGPAKGATKAPTAVIVTEPEHSKEKIKLEGSKAKGQLLIFGATNWDLIGRKEVPKQQAAYRNLGQNLWGPHRYGCLTGVQVRSVASGSCAAHSLLITVEGKLWSWGRNDKGQLGH
GDIKRIDVPKLIESLKGEVFVHAACGRNHTLALTENGSVYAFGENKMGQLGLGNKTDAVPSPAQILYNGQPITKVACGAEFSMIMDCKGNLYSFGCPEYGQLGHNSDGKYIARAQRIEYDCELIARRIAIFIEKTKDGQILPVPNVVVRDIACGINHSLILDSQKRVFSWGFGGY
GRLGHSEQRDEMVPRLVKLFDFPGRGAAQIYAGATCSFAVSEMGGLFFWGATNTSRDSTMYPKAVQDLCGWKVRSLACGKSSIIVAADESTISWGPSPTFGELGYGDNKAKSSTTAQEVKTLDGIYSDQVIMGNSHTLVVARDETEQDKEKLKKLPEYNPRTL
000000000000000000000000000000000000000000000000000000000000000000000000000000000000000000000000000000000000000000000000000000000000000000000000000000000000000000000000000000000000
000000000000000000000000000000000000000000000000000000000000000000000000000000000000000000000000000000000000000000000000000000000000000000000000000000000000000000000000000000000000
00000000000000000000000000000000000000000000000000000000000000000000000000000000000000000000000000000000000000000000000000000000000000000000000000000000000000000000
000000000000000000000000000000000000000000000000000000000000000000000000000000000000000000000000000000000000000000000000000000000000000000000000000000000000000000000000000000000000
000000000000000000000000000000000000000000000000000000000000000000000000000000000000000000000000000000000000000000000000000000000000000000000000000000000000000000000000000000000000
00000000000000000000000000000000000000000000000000000000000000000000000000000000000000000000000000000000000000000000000000000000000000000000000000000000000000000000
000000000000000000000000000000000000000000000000000000000000000000000000000000000000000000000000000000000000000000000000000000000000000000000000000000000000000000000000000000000000
000000000000000000000000000000000000000000000000000000000000000000000000000000000000000000000000000000000000000000000000000000000000000000000000000000000000000000000000000000000000
00000000000000000000000000000000000000000000000000000000000000000000000000000000000000000000000000000000000000000000000000000000000000000000000000000000000000000000
000000000000000000000000000000000000000000000000000000000000000000000000000000000000000000000000000000000000000000000000000000000000000000000000000000000000000000000000000000000000
000000000000000000000000000000000000000000000000000000000000000000000000000000000000000000000000000000000000000000000000000000000000000000000000000000000000000000000000000000000000
00000000000000000000000000000000000000000000000000000000000000000000000000000000000000000000000000000000000000000000000000000000000000000000000000000000000000000000
000000000000000000000000000000000000000000000000000000000000000000000000000000000000000000000000000000000000000000000000000000000000000000000000000000000000000000000000000000000000
000000000000000000000000000000000000000000000000000000000000000000000000000000000000000000000000000000000000000000000000000000000000000000000000000000000000000000000000000000000000
00000000000000000000000000000000000000000000000000000000000000000000000000000000000000000000000000000000000000000000000000000000000000000000000000000000000000000000
000000000000000000000000000000000000000000000000000000000000000000000000000000000000000000000000000000000000000000000000000000000000000000000000000000000000000000000000000000000000
000000000000000000000000000000000000000000000000000000000000000000000000000000000000000000000000000000000000000000000000000000000000000000000000000000000000000000000000000000000000
00000000000000000000000000000000000000000000000000000000000000000000000000000000000000000000000000000000000000000000000000000000000000000000000000000000000000000000

0000000000000000000000000000000000000000000000000000000000000000000000000000000000000000000000000000000000000000000000000000000000000000000000000000000000000000000000000000000
0000000000000000000000000000000000000000000000000000000000000000000000000000000000000000000000000000000000000000000000000000000000000000000000000000000000000000000000000000000
0000000000000000000000000000000000000000000000000000000000000000000000000000000000000000000000000000000000000000000000000000000000000000000000000000000000000000000
>Q54VW7
MTKKSQENISLPQGVLYCWGDKFSGKPTFNPLIPNICFMDNGYNHSLALTETGELYGWGDNRHHQLSSNKAITSIDKPIKIQVIPNQRVKSISCGGNFSAAIMENGLLYVWGTLIEGGKCTESPTKVDLLKGVTCVSIGINHCAVVADSPTTPEKRSVYTWGTNRKGQLGVVGDS
ITNAPRRVTLGSKAISVCCGEEFTAAIVEGNEVFVWGYNKSRQICSNNSDEIISIPVKPFLGRDIVELSCTKNYIAARSGVGNVCVWGSNEQVCRIGDDKFINFPNKIKQISASTSHILVLSEKGEIFSWGNSEDNQLSHKLDVNSSPNINSSSGTTTPTTNTTTTTKKSTLQFN
NVYQLEGRNVLSVYSWGKSSGAIIEPGNFRVDIAESMRRKDNIGSPAPLFFRKLVNYLRGENSKAEGLFRLSGSMARCDDLEKRLDSNEIFPINKYEPYDAADIIKRYLKTLPEPLLTTQLCQKYEKDILNFHNNNINSNSNSSNPNNNNNNNNNNDKIENLILEWIEKLPLENR
QLLIYLLSFLQEISYSQIKYQKQNAMGEKNLAIVFAPNILTRGEIGNDEIVEDMIRLLPTIMKQYPLMEDLIIIDQAQQCLRGSRIPYIIDHWIRVTKERETANNSLFKPEIIKTIINSIVECTLEIKNNQNNQNNQKENNNNNNNINNSNNNNTNNTNISPTTLSPSTLSSSQQ
QQSTPSSLTSSPSPSQRNSLSTGNIANSLSLSSNALFSPNFLSSSKDGNNINNNNNNNNNNNNNNNNNNSSNSSSSSGSSLSSSPNLSPTIVNRSGYSLSFSGGTPSSRDSSVNNLLNTSGGAIQSIQRQFANSGSSSNNNNSNNSPSLIGSNSSPALTFSNSSGNLIETTKLYE
KVLALILSPVLPIGSIMKVLPSIFELDTNNDLFIRLLTTIKLVRTVRSNLDEFIQQQLVMIKEINQCSQFIDKAKQLNENVESVITDLLYVDESIKYWKQVQPLIQLYSQQIQSYFKWWFDLLEKSMTETEEKLFRAKKEFDKLETEKTKLEKQLNHLNSVTLETTDEQILLKKQ
FDQWCLINQIENCSRKIEISNQNLSRVNHDYCSIQSHFEAFKPHLLIVQEFVSIILKSVSLYIEKLDQIKSNTLQMFPQYFNAYIELIFTHHHEIKQQQLSIIKSLAHSITLQHQLLFIENVNQSKYQISLELETKFNSLKRVILENLKDSR
0000000000000000000000000000000000000000000000000000000000000000000000000000000000000000000000000000000000000000000000000000000000000000000000000000000000000000000000000000000
0000000000000000000000000000000000000000000000000000000000000000000000000000000000000000000000000000000000000000000000000000000000000000000000000000000000000000000000000000000
0000000000000000000000000000000000000000000000000000000000000000000000000000000000000000000000000000000000000000000000000000000000000000000000000000000000000000000000000000000
0000000000000000000000000000000000000000000000000000000000000000000000000000000000000000000000000000000000000000000000000000000000000000000000000000000000000000000000000000000
0000000000000000000000000000000000000000000000000000000000000000000000000000000000000000000000000000000000000000000000000000000000000000000000000000000000000000000000000000000
0000000000000000000000000000000000000000000000000000000000000000000000000000000000000000000000000000000000000000000000000000000000000000000000000000000000000000000000000000000
000000000000000000000000000000000000000000000000000000000000000000000000000000000000000000000000000000000000000000000000000000000000000000000000000000000
0000000000000000000000000000000000000000000000000000000000000000000000000000000000000000000000000000000000000000000000000000000000000000000000000000000000000000000000000000000
0000000000000000000000000000000000000000000000000000000000000000000000000000000000000000000000000000000000000000000000000000000000000000000000000000000000000000000000000000000
0000000000000000000000000000000000000000000000000000000000000000000000000000000000000000000000000000000000000000000000000000000000000000000000000000000000000000000000000000000
0000000000000000000000000000000000000000000000000000000000000000000000000000000000000000000000000000000000000000000000000000000000000000000000000000000000000000000000000000000
0000000000000000000000000000000000000000000000000000000000000000000000000000000000000000000000000000000000000000000000000000000000000000000000000000000000000000000000000000000
0000000000000000000000000000000000000000000000000000000000000000000000000000000000000000000000000000000000000000000000000000000000000000000000000000000000000000000000000000000
000000000000000000000000000000000000000000000000000000000000000000000000000000000000000000000000000000000000000000000000000000000000000000000000000000000
0000000000000000000000000000000000000000000000000000000000000000000000000000000000000000000000000000000000000000000000000000000000000000000000000000000000000000000000000000000
0000000000000000000000000000000000000000000000000000000000000000000000000000000000000000000000000000000000000000000000000000000000000000000000000000000000000000000000000000000
0000000000000000000000000000000000000000000000000000000000000000000000000000000000000000000000000000000000000000000000000000000000000000000000000000000000000000000000000000000
0000000000000000000000000000000000000000000000000000000000000000000000000000000000000000000000000000000000000000000000000000000000000000000000000000000000000000000000000000000
0000000000000000000000000000000000000000000000000000000000000000000000000000000000000000000000000000000000000000000000000000000000000000000000000000000000000000000000000000000
0000000000000000000000000000000000000000000000000000000000000000000000000000000000000000000000000000000000000000000000000000000000000000000000000000000000000000000000000000000
000000000000000000000000000000000000000000000000000000000000000000000000000000000000000000000000000000000000000000000000000000000000000000000000000000000
0000000000000000000000000000000000000000000000000000000000000000000000000000000000000000000000000000000000000000000000000000000000000000000000000000000000000000000000000000000
0000000000000000000000000000000000000000000000000000000000000000000000000000000000000000000000000000000000000000000000000000000000000000000000000000000000000000000000000000000
0000000000000000000000000000000000000000000000000000000000000000000000000000000000000000000000000000000000000000000000000000000000000000000000000000000000000000000000000000000
0000000000000000000000000000000000000000000000000000000000000000000000000000000000000000000000000000000000000000000000000000000000000000000000000000000000000000000000000000000
0000000000000000000000000000000000000000000000000000000000000000000000000000000000000000000000000000000000000000000000000000000000000000000000000000000000000000000000000000000
0000000000000000000000000000000000000000000000000000000000000000000000000000000000000000000000000000000000000000000000000000000000000000000000000000000000000000000000000000000
000000000000000000000000000000000000000000000000000000000000000000000000000000000000000000000000000000000000000000000000000000000000000000000000000000000
0000000000000000000000000000000000000000000000000000000000000000000000000000000000000000000000000000000000000000000000000000000000000000000000000000000000000000000000000000000
0000000000000000000000000000000000000000000000000000000000000000000000000000000000000000000000000000000000000000000000000000000000000000000000000000000000000000000000000000000
0000000000000000000000000000000000000000000000000000000000000000000000000000000000000000000000000000000000000000000000000000000000000000000000000000000000000000000000000000000
0000000000000000000000000000000000000000000000000000000000000000000000000000000000000000000000000000000000000000000000000000000000000000000000000000000000000000000000000000000
0000000000000000000000000000000000000000000000000000000000000000000000000000000000000000000000000000000000000000000000000000000000000000000000000000000000000000000000000000000
0000000000000000000000000000000000000000000000000000000000000000000000000000000000000000000000000000000000000000000000000000000000000000000000000000000000000000000000000000000
000000000000000000000000000000000000000000000000000000000000000000000000000000000000000000000000000000000000000000000000000000000000000000000000000000000
0000000000000000000000000000000000000000000000000000000000000000000000000000000000000000000000000000000000000000000000000000000000000000000000000000000000000000000000000000000
0000000000000000000000000000000000000000000000000000000000000000000000000000000000000000000000000000000000000000000000000000000000000000000000000000000000000000000000000000000
0000000000000000000000000000000000000000000000000000000000000000000000000000000000000000000000000000000000000000000000000000000000000000000000000000000000000000000000000000000
0000000000000000000000000000000000000000000000000000000000000000000000000000000000000000000000000000000000000000000000000000000000000000000000000000000000000000000000000000000
0000000000000000000000000000000000000000000000000000000000000000000000000000000000000000000000000000000000000000000000000000000000000000000000000000000000000000000000000000000
0000000000000000000000000000000000000000000000000000000000000000000000000000000000000000000000000000000000000000000000000000000000000000000000000000000000000000000000000000000
000000000000000000000000000000000000000000000000000000000000000000000000000000000000000000000000000000000000000000000000000000000000000000000000000000000
0000000000000000000000000000000000000000000000000000000000000000000000000000000000000000000000000000000000000000000000000000000000000000000000000000000000000000000000000000000
0000000000000000000000000000000000000000000000000000000000000000000000000000000000000000000000000000000000000000000000000000000000000000000000000000000000000000000000000000000
0000000000000000000000000000000000000000000000000000000000000000000000000000000000000000000000000000000000000000000000000000000000000000000000000000000000000000000000000000000
0000000000000000000000000000000000000000000000000000000000000000000000000000000000000000000000000000000000000000000000000000000000000000000000000000000000000000000000000000000
0000000000000000000000000000000000000000000000000000000000000000000000000000000000000000000000000000000000000000000000000000000000000000000000000000000000000000000000000000000
0000000000000000000000000000000000000000000000000000000000000000000000000000000000000000000000000000000000000000000000000000000000000000000000000000000000000000000000000000000
000000000000000000000000000000000000000000000000000000000000000000000000000000000000000000000000000000000000000000000000000000000000000000000000000000000
>Q58310
MVIIMSEAELKEAMKRNPHLRRYIENFKRTYMRIPDFMVSLSRELKELKYPNIIYPVGDPIFIHIFGTPETKTKYIVIEPTLETAEEKLKYKMILNKILELAPYEETPKSVEEFEEVLTRLFNACTKVTEAVGEEGFFQRIFRFADNKIKITPEERDKFLYILKRDLIGLGNLEP
IGRDPYLEDIHVIGPKNCHVVHKIFGMLPTNITWEDEIELADYLKNLGERMGRPVSDANPIVDGTLPDGSRINIIYSTDVSPKGPSFTIRKFTDVPISVTQLISWGTFSTEVAAYLWLCLEYGMSIFICGETASGKTTTLNAILPFIKPNSKIFSCEDTPEVKPPHPVWQQLVTR
ERGPEESRVTLFDLLRAALRSRPNYIIVGEIRSVEAAVAFQAMQTGHPVLSTFHAANVRKMIQRLNGDPINVPLTFMDNLNVALFQLAVYQRGKVLRRVVSIEEIEGYYKEVDGVVTRAVFQWEPDKDRHVFTGRNNSYVLEEKIAKAAGYEDPRDIYNELELRARILEEMIARE
IFDYYQVRDIIWAFYEKGLEGLPFPI
0000000000000000000000000000000000000000000000000000000000000000000000000000000000000000000000000000000000000000000000000000000000000000000000000000000000000000000000000000000
0000000000000000000000000000000000000000000000000000000000000000000000000000000000000000000000000000000000000000000000000000000000000000000000000000000000000000000000000000000
0000000000000000000000000000000000000000000000000000000000000000000000000000000000000000000000000000000000000000000000000000000000000000000000000000000000000000000000000000000
00000000000000000000000000

0000000000000000000000000000000000000000000000000000000000000000000000000000000000000000000000000000000000000000000000000000000000000000000000000000000000000000000000000000000
0000000000000000000000000000000000000000000000000000000000000000000000000000000000000000000000000000000000000000000000000000000000000000000000000000000000000000000000000000000
0000000000000000000000000000000000000000000000000000000000000000000000000000000000000000000000000000000000000000000000000000000000000000000000000000000000000000000000000000000
00000000000000000000000000
0000000000000000000000000000000000000000000000000000000000000000000000000000000000000000000000000000000000000000000000000000000000000000000000000000000000000000000000000000000
0000000000000000000000000000000000000000000000000000000000000000000000000000000000000000000000000000000000000000000000000000000000000000000000000000000000000000000000000000000
0000000000000000000000000000000000000000000000000000000000000000000000000000000000000000000000000000000000000000000000000000000000000000000000000000000000000000000000000000000
00000000000000000000000000
0000000000000000000000000000000000000000000000000000000000000000000000000000000000000000000000000000000000000000000000000000000000000000000000000000000000000000000000000000000
0000000000000000000000000000000000000000000000000000000000000000000000000000000000000000000000000000000000000000000000000000000000000000000000000000000000000000000000000000000
0000000000000000000000000000000000000000000000000000000000000000000000000000000000000000000000000000000000000000000000000000000000000000000000000000000000000000000000000000000
00000000000000000000000000
0000000000000000000000000000000000000000000000000000000000000000000000000000000000000000000000000000000000000000000000000000000000000000000000000000000000000000000000000000000
0000000000000000000000000000000000000000000000000000000000000000000000000000000000000000000000000000000000000000000000000000000000000000000000000000000000000000000000000000000
0000000000000000000000000000000000000000000000000000000000000000000000000000000000000000000000000000000000000000000000000000000000000000000000000000000000000000000000000000000
00000000000000000000000000
0000000000000000000000000000000000000000000000000000000000000000000000000000000000000000000000000000000000000000000000000000000000000000000000000000000000000000000000000000000
0000000000000000000000000000000000000000000000000000000000000000000000000000000000000000000000000000000000000000000000000000000000000000000000000000000000000000000000000000000
0000000000000000000000000000000000000000000000000000000000000000000000000000000000000000000000000000000000000000000000000000000000000000000000000000000000000000000000000000000
00000000000000000000000000
0000000000000000000000000000000000000000000000000000000000000000000000000000000000000000000000000000000000000000000000000000000000000000000000000000000000000000000000000000000
0000000000000000000000000000000000000000000000000000000000000000000000000000000000000000000000000000000000000000000000000000000000000000000000000000000000000000000000000000000
0000000000000000000000000000000000000000000000000000000000000000000000000000000000000000000000000000000000000000000000000000000000000000000000000000000000000000000000000000000
00000000000000000000000000
>Q59XM1
MDNLDPNSSLQVEKLRNRKSRAVWQNNNTTTHNNPYANLSTGEKSRSRHNTGSSYVSPYGGGNGEENAYTGNNNKSNTSGNLLQVPGAGGGGDLNSNKKQSRRMSIHVSARQHGRSFSQTGPIDMANLPALPKIGGVTTSGVGGAGGDVMTRTGGLTIEQKIFKELSQGSAAEVD
DYYKTLLKQKNLITRDIKDNINQNQKNILQLTKDLKETQEELIELRGTTKELYEVLGYFKESAQRRLELEFEPETQKELHSPQKSNQLGIPSNKKKDRSSIMVLKKMWDSQLQSLFKHVDGASKFVQPLPNRHIVAESGRWFEVNVGNWKPSYPTHLFIFNDLILIAVKKSSSSS
QEPTTGGSNGGSKSRLQAVQCWPLTQVSLQQIKSPKKDDDKMYFINLKSKSLSYVYSTDRYDHFVKVTEAFNKGRNEMIQSERLLDSRLSSPSNNNGDSKEEKRQLRESLRNSGNYKEGVTDDAGGAATGGGRKSAGTPNRNSTDYVLHDISARVHSRNRSQDLGNNFKLANNGK
SQFFNEIKTLEDRLDDVDVEISHNQYAEAVELISIIESKLRNIENALTNQRNGGKNVNIADELLLLDVSKLKIKNRKENVSNGLIFDLQHNIAKLKQDDIDNILTLFDNLEQLDRGVQGYLDSMSAYLSTTVSKLIVGLQGSTKIDVVNYLSNLMVINVSIVKRTIQTYEQIIAP
ILKRHGDVDSSGLINWCIDEFTKLCKQIKKHLYGTLLISSGINMETDEPIYKVERKLYDNFLKIMQPQLEELKSVGLNVDYIFESILNLE
0000000000000000000000000000000000000000000000000000000000000000000000000000000000000000000000000000000000000000000000000000000000000000000000000000000000000000000000000000000
0000000000000000000000000000000000000000000000000000000000000000000000000000000000000000000000000000000000000000000000000000000000000000000000000000000000000000000000000000000
0000000000000000000000000000000000000000000000000000000000000000000000000000000000000000000000000000000000000000000000000000000000000000000000000000000000000000000000000000000
0000000000000000000000000000000000000000000000000000000000000000000000000000000000000000000000000000000000000000000000000000000000000000000000000000000000000000000000000000000
000000000000000000000000000000000000000000000000000000000000000000000000000000000000000000
0000000000000000000000000000000000000000000000000000000000000000000000000000000000000000000000000000000000000000000000000000000000000000000000000000000000000000000000000000000
0000000000000000000000000000000000000000000000000000000000000000000000000000000000000000000000000000000000000000000000000000000000000000000000000000000000000000000000000000000
0000000000000000000000000000000000000000000000000000000000000000000000000000000000000000000000000000000000000000000000000000000000000000000000000000000000000000000000000000000
0000000000000000000000000000000000000000000000000000000000000000000000000000000000000000000000000000000000000000000000000000000000000000000000000000000000000000000000000000000
000000000000000000000000000000000000000000000000000000000000000000000000000000000000000000
0000000000000000000000000000000000000000000000000000000000000000000000000000000000000000000000000000000000000000000000000000000000000000000000000000000000000000000000000000000
0000000000000000000000000000000000000000000000000000000000000000000000000000000000000000000000000000000000000000000000000000000000000000000000000000000000000000000000000000000
0000000000000000000000000000000000000000000000000000000000000000000000000000000000000000000000000000000000000000000000000000000000000000000000000000000000000000000000000000000
0000000000000000000000000000000000000000000000000000000000000000000000000000000000000000000000000000000000000000000000000000000000000000000000000000000000000000000000000000000
000000000000000000000000000000000000000000000000000000000000000000000000000000000000000000
0000000000000000000000000000000000000000000000000000000000000000000000000000000000000000000000000000000000000000000000000000000000000000000000000000000000000000000000000000000
0000000000000000000000000000000000000000000000000000000000000000000000000000000000000000000000000000000000000000000000000000000000000000000000000000000000000000000000000000000
0000000000000000000000000000000000000000000000000000000000000000000000000000000000000000000000000000000000000000000000000000000000000000000000000000000000000000000000000000000
0000000000000000000000000000000000000000000000000000000000000000000000000000000000000000000000000000000000000000000000000000000000000000000000000000000000000000000000000000000
000000000000000000000000000000000000000000000000000000000000000000000000000000000000000000
0000000000000000000000000000000000000000000000000000000000000000000000000000000000000000000000000000000000000000000000000000000000000000000000000000000000000000000000000000000
0000000000000000000000000000000000000000000000000000000000000000000000000000000000000000000000000000000000000000000000000000000000000000000000000000000000000000000000000000000
0000000000000000000000000000000000000000000000000000000000000000000000000000000000000000000000000000000000000000000000000000000000000000000000000000000000000000000000000000000
0000000000000000000000000000000000000000000000000000000000000000000000000000000000000000000000000000000000000000000000000000000000000000000000000000000000000000000000000000000
000000000000000000000000000000000000000000000000000000000000000000000000000000000000000000
0000000000000000000000000000000000000000000000000000000000000000000000000000000000000000000000000000000000000000000000000000000000000000000000000000000000000000000000000000000
0000000000000000000000000000000000000000000000000000000000000000000000000000000000000000000000000000000000000000000000000000000000000000000000000000000000000000000000000000000
0000000000000000000000000000000000000000000000000000000000000000000000000000000000000000000000000000000000000000000000000000000000000000000000000000000000000000000000000000000
0000000000000000000000000000000000000000000000000000000000000000000000000000000000000000000000000000000000000000000000000000000000000000000000000000000000000000000000000000000
000000000000000000000000000000000000000000000000000000000000000000000000000000000000000000
0000000000000000000000000000000000000000000000000000000000000000000000000000000000000000000000000000000000000000000000000000000000000000000000000000000000000000000000000000000
0000000000000000000000000000000000000000000000000000000000000000000000000000000000000000000000000000000000000000000000000000000000000000000000000000000000000000000000000000000
0000000000000000000000000000000000000000000000000000000000000000000000000000000000000000000000000000000000000000000000000000000000000000000000000000000000000000000000000000000
0000000000000000000000000000000000000000000000000000000000000000000000000000000000000000000000000000000000000000000000000000000000000000000000000000000000000000000000000000000
000000000000000000000000000000000000000000000000000000000000000000000000000000000000000000
0000000000000000000000000000000000000000000000000000000000000000000000000000000000000000000000000000000000000000000000000000000000000000000000000000000000000000000000000000000
0000000000000000000000000000000000000000000000000000000000000000000000000000000000000000000000000000000000000000000000000000000000000000000000000000000000000000000000000000000
0000000000000000000000000000000000000000000000000000000000000000000000000000000000000000000000000000000000000000000000000000000000000000000000000000000000000000000000000000000
0000000000000000000000000000000000000000000000000000000000000000000000000000000000000000000000000000000000000000000000000000000000000000000000000000000000000000000000000000000
000000000000000000000000000000000000000000000000000000000000000000000000000000000000000000
>Q5AH02
MAIGNIAARYLSSLEETDTTATTTTTTTSNVLQPSNRLNSPTKFNRKSLDNNSQDLDALARALNITPSKPETPTSISKGSATSLLRTKFESPSATVSSFKSTSSSNGVSPTKKNHFVEITSDETPSWANKNYKDILNKSPTKFNTQSNVHTPLKQLNQPIGTPSSSSLSPAKNAS
KSSPGYEYLCRIEAIKQWLESVLQEQITQSASQLISYIRNGIHLAKLANVVLPTSKPVFLNDSKLQFKHTENINRFFQLLDFFNMPDLFRFELTDLYDAKNVPKVWFCLHALSYILHKQDPSYPAMNNLVGKVDFSADDIRTANRALVNSPLPNFSSADTGEGKSDTSNNNSSTT
SATAAFMDKVTSPVKKTPSPLKRPQQLQKKQLELVEDNKPELTQDSSGLSKISRDDPFTDRVDLAPPSTSNAKLELHTPPSKSLEFKIKSPIIDLSHRDSDYYTPELESHLPNIIKFQSLARGAVFRYLMFVDRILLKSYQDEFTNLFAIIRGNKARRKTVHKHRDELRLYSFEI

IELQSIIRKNFVINKKPNFTSITNDVETVELQSLIRGKLTRDWKKYVTNGLEKFTPQIIDFQSLVRMKSIYSKSNKVISYKDEILPSLIELQSIARSQLYHRFSRSNAIDETEIIKIQSIIRRNAVIEDLYTKLSKVRSNKRRLIELQSIARGGVARTKLCNSVLVTLIYEDGIL
NQLFAKIRGDNYRKKFNSQKSELLKYEKSSIIPVQTLFRGVLSRYTKEVTLSDIFDQIDSVITLQSVARGKLMRGSIYEFSDYYQKHIKQVVKAQAILQRVFAQNAYKQLITSKNPPLKVIRRFAPLLSNNDRDFQDEMTLSDLKDLIIEKCKANEEYENQIEQLDMKLGLLDKN
KISIEEFLKPTKGKTFKPIVENVKNLERLNKSLKKKIELWQTLFYFIQTNPIYLTKLFNSIPYTKNQTKSGQDLFQSVIQLFPVRDSSITYHSREEYFLVKLMIQLMQNDTANSNNLGDITKLHLTNWIDFFTNFNNHTFQRQHLKALLGKFVIRIVDNEQVDFESDPIRIYNQI
IDHEMKVYGRSEKSRDISPQAAIQLPEVSNKFVGNLMSLRETCSDLLSMLQKNASGNKALLQIPDHVKLICRQGYLCAQRKFPDKSDQQHLAVAGVIFVKHYLGSILQVPENYGILTGNNDTQKAKSKDNLRYLYRVMLQLFSMKPFNDNFLKPLNEYIMASTDTVKSIISQAII
NVGEIETVYELHDYDDLVTHQRPKLTISVNSLIQLEKSILQNVDIITTGNDDQLYKTCVEVEKLLISPQDMLTLTDLSSVTLNLNPTTQEESIVDSKTKTLFTQAKRCLLYIIRVQEEDDSDDLLELLISGIKPSHEQRFKEIVQYEKAEQDISLNNSKSVTANTVNKKKSTRPY
SGTSLGDLSNLTYHELKKMCLEIILKLESMGELTRKNSFQTLLNQIAMDIKTKDSQRQCRWQQLQVSQKTIKKLSEKENYLKTQLHNYKKHVESVLLELQSKSKSNDKNWKRRLFNIMVIPVFSKQYFYHRELRKHNRLPKFGSYKYSAKKLIDQKVLIDFSTANNVATASKLDF
MFSCHQVGKFTIEVASGTVNIPGATNTITLDELLALQYENKTKFELFDGMATFDSNNFMGLIFRKFYDLKKE
0000000000000000000000000000000000000000000000000000000000000000000000000000000000000000000000000000000000000000000000000000000000000000000000000000000000000000000000000000000
0000000000000000000000000000000000000000000000000000000000000000000000000000000000000000000000000000000000000000000000000000000000000000000000000000000000000000000000000000000
0000000000000000000000000000000000000000000000000000000000000000000000000000000000000000000000000000000000000000000000000000000000000000000000000000000000000000000000000000000
0000000000000000000000000000000000000000000000000000000000000000000000000000000000000000000000000000000000000000000000000000000000000000000000000000000000000000000000000000000
0000000000000000000000000000000000000000000000000000000000000000000000000000000000000000000000000000000000000000000000000000000000000000000000000000000000000000000000000000000
0000000000000000000000000000000000000000000000000000000000000000000000000000000000000000000000000000000000000000000000000000000000000000000000000000000000000000000000000000000
0000000000000000000000000000000000000000000000000000000000000000000000000000000000000000000000000000000000000000000000000000000000000000000000000000000000000000000000000000000
0000000000000000000000000000000000000000000000000000000000000000000000000000000000000000000000000000000000000000000000000000000000000000000000000000000000000000000000000000000
0000000000000000000000000000000000000000000000000000000000000000000000000000000000000000000000000000000000000000000000000000000000000000000000000000000000000000000000000000000
000000000000000000000000000000000000000000000000000000000000000000000000
0000000000000000000000000000000000000000000000000000000000000000000000000000000000000000000000000000000000000000000000000000000000000000000000000000000000000000000000000000000
0000000000000000000000000000000000000000000000000000000000000000000000000000000000000000000000000000000000000000000000000000000000000000000000000000000000000000000000000000000
0000000000000000000000000000000000000000000000000000000000000000000000000000000000000000000000000000000000000000000000000000000000000000000000000000000000000000000000000000000
0000000000000000000000000000000000000000000000000000000000000000000000000000000000000000000000000000000000000000000000000000000000000000000000000000000000000000000000000000000
0000000000000000000000000000000000000000000000000000000000000000000000000000000000000000000000000000000000000000000000000000000000000000000000000000000000000000000000000000000
0000000000000000000000000000000000000000000000000000000000000000000000000000000000000000000000000000000000000000000000000000000000000000000000000000000000000000000000000000000
0000000000000000000000000000000000000000000000000000000000000000000000000000000000000000000000000000000000000000000000000000000000000000000000000000000000000000000000000000000
0000000000000000000000000000000000000000000000000000000000000000000000000000000000000000000000000000000000000000000000000000000000000000000000000000000000000000000000000000000
0000000000000000000000000000000000000000000000000000000000000000000000000000000000000000000000000000000000000000000000000000000000000000000000000000000000000000000000000000000
000000000000000000000000000000000000000000000000000000000000000000000000
0000000000000000000000000000000000000000000000000000000000000000000000000000000000000000000000000000000000000000000000000000000000000000000000000000000000000000000000000000000
0000000000000000000000000000000000000000000000000000000000000000000000000000000000000000000000000000000000000000000000000000000000000000000000000000000000000000000000000000000
0000000000000000000000000000000000000000000000000000000000000000000000000000000000000000000000000000000000000000000000000000000000000000000000000000000000000000000000000000000
0000000000000000000000000000000000000000000000000000000000000000000000000000000000000000000000000000000000000000000000000000000000000000000000000000000000000000000000000000000
0000000000000000000000000000000000000000000000000000000000000000000000000000000000000000000000000000000000000000000000000000000000000000000000000000000000000000000000000000000
0000000000000000000000000000000000000000000000000000000000000000000000000000000000000000000000000000000000000000000000000000000000000000000000000000000000000000000000000000000
0000000000000000000000000000000000000000000000000000000000000000000000000000000000000000000000000000000000000000000000000000000000000000000000000000000000000000000000000000000
0000000000000000000000000000000000000000000000000000000000000000000000000000000000000000000000000000000000000000000000000000000000000000000000000000000000000000000000000000000
0000000000000000000000000000000000000000000000000000000000000000000000000000000000000000000000000000000000000000000000000000000000000000000000000000000000000000000000000000000
000000000000000000000000000000000000000000000000000000000000000000000000
0000000000000000000000000000000000000000000000000000000000000000000000000000000000000000000000000000000000000000000000000000000000000000000000000000000000000000000000000000000
0000000000000000000000000000000000000000000000000000000000000000000000000000000000000000000000000000000000000000000000000000000000000000000000000000000000000000000000000000000
0000000000000000000000000000000000000000000000000000000000000000000000000000000000000000000000000000000000000000000000000000000000000000000000000000000000000000000000000000000
0000000000000000000000000000000000000000000000000000000000000000000000000000000000000000000000000000000000000000000000000000000000000000000000000000000000000000000000000000000
0000000000000000000000000000000000000000000000000000000000000000000000000000000000000000000000000000000000000000000000000000000000000000000000000000000000000000000000000000000
0000000000000000000000000000000000000000000000000000000000000000000000000000000000000000000000000000000000000000000000000000000000000000000000000000000000000000000000000000000
0000000000000000000000000000000000000000000000000000000000000000000000000000000000000000000000000000000000000000000000000000000000000000000000000000000000000000000000000000000
0000000000000000000000000000000000000000000000000000000000000000000000000000000000000000000000000000000000000000000000000000000000000000000000000000000000000000000000000000000
0000000000000000000000000000000000000000000000000000000000000000000000000000000000000000000000000000000000000000000000000000000000000000000000000000000000000000000000000000000
000000000000000000000000000000000000000000000000000000000000000000000000
0000000000000000000000000000000000000000000000000000000000000000000000000000000000000000000000000000000000000000000000000000000000000000000000000000000000000000000000000000000
0000000000000000000000000000000000000000000000000000000000000000000000000000000000000000000000000000000000000000000000000000000000000000000000000000000000000000000000000000000
0000000000000000000000000000000000000000000000000000000000000000000000000000000000000000000000000000000000000000000000000000000000000000000000000000000000000000000000000000000
0000000000000000000000000000000000000000000000000000000000000000000000000000000000000000000000000000000000000000000000000000000000000000000000000000000000000000000000000000000
0000000000000000000000000000000000000000000000000000000000000000000000000000000000000000000000000000000000000000000000000000000000000000000000000000000000000000000000000000000
0000000000000000000000000000000000000000000000000000000000000000000000000000000000000000000000000000000000000000000000000000000000000000000000000000000000000000000000000000000
0000000000000000000000000000000000000000000000000000000000000000000000000000000000000000000000000000000000000000000000000000000000000000000000000000000000000000000000000000000
0000000000000000000000000000000000000000000000000000000000000000000000000000000000000000000000000000000000000000000000000000000000000000000000000000000000000000000000000000000
0000000000000000000000000000000000000000000000000000000000000000000000000000000000000000000000000000000000000000000000000000000000000000000000000000000000000000000000000000000
000000000000000000000000000000000000000000000000000000000000000000000000
0000000000000000000000000000000000000000000000000000000000000000000000000000000000000000000000000000000000000000000000000000000000000000000000000000000000000000000000000000000
0000000000000000000000000000000000000000000000000000000000000000000000000000000000000000000000000000000000000000000000000000000000000000000000000000000000000000000000000000000
0000000000000000000000000000000000000000000000000000000000000000000000000000000000000000000000000000000000000000000000000000000000000000000000000000000000000000000000000000000
0000000000000000000000000000000000000000000000000000000000000000000000000000000000000000000000000000000000000000000000000000000000000000000000000000000000000000000000000000000
0000000000000000000000000000000000000000000000000000000000000000000000000000000000000000000000000000000000000000000000000000000000000000000000000000000000000000000000000000000
0000000000000000000000000000000000000000000000000000000000000000000000000000000000000000000000000000000000000000000000000000000000000000000000000000000000000000000000000000000
0000000000000000000000000000000000000000000000000000000000000000000000000000000000000000000000000000000000000000000000000000000000000000000000000000000000000000000000000000000
0000000000000000000000000000000000000000000000000000000000000000000000000000000000000000000000000000000000000000000000000000000000000000000000000000000000000000000000000000000
0000000000000000000000000000000000000000000000000000000000000000000000000000000000000000000000000000000000000000000000000000000000000000000000000000000000000000000000000000000
000000000000000000000000000000000000000000000000000000000000000000000000
0000000000000000000000000000000000000000000000000000000000000000000000000000000000000000000000000000000000000000000000000000000000000000000000000000000000000000000000000000000
0000000000000000000000000000000000000000000000000000000000000000000000000000000000000000000000000000000000000000000000000000000000000000000000000000000000000000000000000000000

0000000000000000000000000000000000000000000000000000000000000000000000000000000000000000000000000000000000000000000000000000000000000000000000000000000000000000000000000000
0000000000000000000000000000000000000000000000000000000000000000000000000000000000000000000000000000000000000000000000000000000000000000000000000000000000000000000000000000
0000000000000000000000000000000000000000000000000000000000000000000000000000000000000000000000000000000000000000000000000000000000000000000000000000000000000000000000000000
0000000000000000000000000000000000000000000000000000000000000000000000000000000000000000000000000000000000000000000000000000000000000000000000000000000000000000000000000000
0000000000000000000000000000000000000000000000000000000000000000000000000000000000000000000000000000000000000000000000000000000000000000000000000000000000000000000000000000
0000000000000000000000000000000000000000000000000000000000000000000000000000000000000000000000000000000000000000000000000000000000000000000000000000000000000000000000000000
0000000000000000000000000000000000000000000000000000000000000000000000000000000000000000000000000000000000000000000000000000000000000000000000000000000000000000000000000000
000000000000000000000000000000000000000000000000000000000000000000000000
>Q5RBA3
MRHVQAEPSPSSEPEAGPSQPPVRQGALQGGLLMGYSPAGGATSPGVYQVSIFSPPAGTSEPHRALKRQAPPTEGPRELKRGPGLGAREGLPPEEPSTVGLLGPEGLGLGLGVASQHFSHRGLCVVEQRSSVTSSWTSGAWSPPCPPSNASCNTLHTRDWASPDPGGQGSLGESP
GPAPPGQLHTLDTDLHSLAQIGGKSPVAGVGNGGSLWPRESPGTANGHSPEHTPPGPGPPGPCPTKRRLLPAGEAPDVSSEEEGPAPRRRRGSLGHPTAANSSDAKATPFWSHLLPGPKEPVLDPTDCGPMGRRLKGARRLKLSPLRSLRKGPGLLSPPSASPVPTPAVSRTLLG
SFEESLLRGRFAPSGHIEGFTAEIGASGSYCPQHVTLPVTVTFFDVSEQNAPAPFLGIVDLNPLGRKGYSVPKVGTIQVTLFNPNQTVVKMFLVTFDFSDMPAAHMTFLRHRLFLVPVGEEGNANPTHRLLCYLLHLRFRSSRSGRLSLHGDIRLLFSRRSLELDTGLPYELQAV
TEAPHNPRYSPLP
0000000000000000000000000000000000000000000000000000000000000000000000000000000000000000000000000000000000000000000000000000000000000000000000000000000000000000000000000000000
0000000000000000000000000000000000000000000000000000000000000000000000000000000000000000000000000000000000000000000000000000000000000000000000000000000000000000000000000000000
0000000000000000000000000000000000000000000000000000000000000000000000000000000000000000000000000000000000000000000000000000000000000000000000000000000000000000000000000000000
0000000000000
0000000000000000000000000000000000000000000000000000000000000000000000000000000000000000000000000000000000000000000000000000000000000000000000000000000000000000000000000000000
0000000000000000000000000000000000000000000000000000000000000000000000000000000000000000000000000000000000000000000000000000000000000000000000000000000000000000000000000000000
0000000000000000000000000000000000000000000000000000000000000000000000000000000000000000000000000000000000000000000000000000000000000000000000000000000000000000000000000000000
0000000000000
0000000000000000000000000000000000000000000000000000000000000000000000000000000000000000000000000000000000000000000000000000000000000000000000000000000000000000000000000000000
0000000000000000000000000000000000000000000000000000000000000000000000000000000000000000000000000000000000000000000000000000000000000000000000000000000000000000000000000000000
0000000000000000000000000000000000000000000000000000000000000000000000000000000000000000000000000000000000000000000000000000000000000000000000000000000000000000000000000000000
0000000000000
0000000000000000000000000000000000000000000000000000000000000000000000000000000000000000000000000000000000000000000000000000000000000000000000000000000000000000000000000000000
0000000000000000000000000000000000000000000000000000000000000000000000000000000000000000000000000000000000000000000000000000000000000000000000000000000000000000000000000000000
0000000000000000000000000000000000000000000000000000000000000000000000000000000000000000000000000000000000000000000000000000000000000000000000000000000000000000000000000000000
0000000000000
0000000000000000000000000000000000000000000000000000000000000000000000000000000000000000000000000000000000000000000000000000000000000000000000000000000000000000000000000000000
0000000000000000000000000000000000000000000000000000000000000000000000000000000000000000000000000000000000000000000000000000000000000000000000000000000000000000000000000000000
0000000000000000000000000000000000000000000000000000000000000000000000000000000000000000000000000000000000000000000000000000000000000000000000000000000000000000000000000000000
0000000000000
0000000000000000000000000000000000000000000000000000000000000000000000000000000000000000000000000000000000000000000000000000000000000000000000000000000000000000000000000000000
0000000000000000000000000000000000000000000000000000000000000000000000000000000000000000000000000000000000000000000000000000000000000000000000000000000000000000000000000000000
0000000000000000000000000000000000000000000000000000000000000000000000000000000000000000000000000000000000000000000000000000000000000000000000000000000000000000000000000000000
0000000000000
0000000000000000000000000000000000000000000000000000000000000000000000000000000000000000000000000000000000000000000000000000000000000000000000000000000000000000000000000000000
0000000000000000000000000000000000000000000000000000000000000000000000000000000000000000000000000000000000000000000000000000000000000000000000000000000000000000000000000000000
0000000000000000000000000000000000000000000000000000000000000000000000000000000000000000000000000000000000000000000000000000000000000000000000000000000000000000000000000000000
0000000000000
>Q5T5M9
MELEGQWWRGQLAADIHQALRYKELKLPSYKGQSPQLSLRRYFADLIAIVSNRFTLCPSARHLAVYLLDLFMDRYDISIQQLHLVALSCLLLASKFEEKEDSVPKLEQLNSLGCMTNMNLVLTKQNLLHMELLLLETFQWNLCLPTAAHFIEYYLSEAVHETDLHDGWPMICLEK
TKLYMAKYADYFLEVSLQVAAACVASSRIILRLSPTWPTRLHRLTAYSWDFLVQCIERLLIAHDNDVKEANKQRGQAGPQSAQLSVFQTASQPSRPVHFQQPQYLHQTHQTSLQYRHPTSEQPSCQQIVSTTHTSSYTLQTCPAGFQTSVQGLGHMQTGVGMSLAIPVEVKPCLS
VSYNRSYQINEHYPCITPCFER
0000000000000000000000000000000000000000000000000000000000000000000000000000000000000000000000000000000000000000000000000000000000000000000000000000000000000000000000000000000
0000000000000000000000000000000000000000000000000000000000000000000000000000000000000000000000000000000000000000000000000000000000000000000000000000000000000000000000000000000
0000000000000000000000
0000000000000000000000000000000000000000000000000000000000000000000000000000000000000000000000000000000000000000000000000000000000000000000000000000000000000000000000000000000
0000000000000000000000000000000000000000000000000000000000000000000000000000000000000000000000000000000000000000000000000000000000000000000000000000000000000000000000000000000
0000000000000000000000
0000000000000000000000000000000000000000000000000000000000000000000000000000000000000000000000000000000000000000000000000000000000000000000000000000000000000000000000000000000
0000000000000000000000000000000000000000000000000000000000000000000000000000000000000000000000000000000000000000000000000000000000000000000000000000000000000000000000000000000
0000000000000000000000
0000000000000000000000000000000000000000000000000000000000000000000000000000000000000000000000000000000000000000000000000000000000000000000000000000000000000000000000000000000
0000000000000000000000000000000000000000000000000000000000000000000000000000000000000000000000000000000000000000000000000000000000000000000000000000000000000000000000000000000
0000000000000000000000
0000000000000000000000000000000000000000000000000000000000000000000000000000000000000000000000000000000000000000000000000000000000000000000000000000000000000000000000000000000
0000000000000000000000000000000000000000000000000000000000000000000000000000000000000000000000000000000000000000000000000000000000000000000000000000000000000000000000000000000
0000000000000000000000
0000000000000000000000000000000000000000000000000000000000000000000000000000000000000000000000000000000000000000000000000000000000000000000000000000000000000000000000000000000
0000000000000000000000000000000000000000000000000000000000000000000000000000000000000000000000000000000000000000000000000000000000000000000000000000000000000000000000000000000
0000000000000000000000
0000000000000000000000000000000000000000000000000000000000000000000000000000000000000000000000000000000000000000000000000000000000000000000000000000000000000000000000000000000
0000000000000000000000000000000000000000000000000000000000000000000000000000000000000000000000000000000000000000000000000000000000000000000000000000000000000000000000000000000
0000000000000000000000
>Q60353
MRYLNLKDTVLLGRNFNEYVRMFNLNEDLLSNKILDVASGVSSFCAEGNKKGYNITSSDKIYNLKPEEIEEKCKKDLDFMEKHLRGMFKNNFNWNEFKTVDEWKKTRERTYKTFIEDYKTNRKRYIYTTYPKTNFKDDEFAISLVGHFLLLYDNILNYQFHKETIDELLRISEEI
RIFPILNLRGEKSIFLDKILKEYKARIEKTDYEFMKGGNKVLIIRR

0000000000000000000000000000000000000000000000000000000000000000000000000000000000000000000000000000000000000000000000000000000000000000000000000000000000000000000000000000000
0000000000000000000000000000000000000000000000
0000000000000000000000000000000000000000000000000000000000000000000000000000000000000000000000000000000000000000000000000000000000000000000000000000000000000000000000000000000
0000000000000000000000000000000000000000000000
0000000000000000000000000000000000000000000000000000000000000000000000000000000000000000000000000000000000000000000000000000000000000000000000000000000000000000000000000000000
0000000000000000000000000000000000000000000000
0000000000000000000000000000000000000000000000000000000000000000000000000000000000000000000000000000000000000000000000000000000000000000000000000000000000000000000000000000000
0000000000000000000000000000000000000000000000
0000000000000000000000000000000000000000000000000000000000000000000000000000000000000000000000000000000000000000000000000000000000000000000000000000000000000000000000000000000
0000000000000000000000000000000000000000000000
0000000000000000000000000000000000000000000000000000000000000000000000000000000000000000000000000000000000000000000000000000000000000000000000000000000000000000000000000000000
0000000000000000000000000000000000000000000000
0000000000000000000000000000000000000000000000000000000000000000000000000000000000000000000000000000000000000000000000000000000000000000000000000000000000000000000000000000000
0000000000000000000000000000000000000000000000
>Q63PS2
MNAVQKKIKVLCVDDSALIRSLMTEIINSQPDMEVCATAPDPLVARELIKQHNPDVLTLDVEMPRMDGLDFLEKLMRLRPMPVVMVSSLTERGSEITLRALELGAVDFVTKPRVGIRDGMLDYSEKLADKVRAASRARVRQNPQPHAAAAAAAHGHAGAAAPLINNPLVSTEKLI
IVGASTGGTEAIREVLTPLPPDAPAVLIAQHMPPGFTRSFAQRLNGLCRISVKEAEHGERVLPGHAYIAPGHAHLLLARSGANYIAHLSDEPPVNRHRPSVDVLFRSAAQHAGKNALGVILTGMGRDGAAGLLEMKKAGAYTFAQDEASCVVFGMPREAIAMGGVDDVAPLSDMS
RRIMARLASMGDRVQRV
0000000000000000000000000000000000000000000000000000000000000000000000000000000000000000000000000000000000000000000000000000000000000000000000000000000000000000000000000000000
0000000000000000000000000000000000000000000000000000000000000000000000000000000000000000000000000000000000000000000000000000000000000000000000000000000000000000000000000000000
00000000000000000
0000000000000000000000000000000000000000000000000000000000000000000000000000000000000000000000000000000000000000000000000000000000000000000000000000000000000000000000000000000
0000000000000000000000000000000000000000000000000000000000000000000000000000000000000000000000000000000000000000000000000000000000000000000000000000000000000000000000000000000
00000000000000000
0000000000000000000000000000000000000000000000000000000000000000000000000000000000000000000000000000000000000000000000000000000000000000000000000000000000000000000000000000000
0000000000000000000000000000000000000000000000000000000000000000000000000000000000000000000000000000000000000000000000000000000000000000000000000000000000000000000000000000000
00000000000000000
0000000000000000000000000000000000000000000000000000000000000000000000000000000000000000000000000000000000000000000000000000000000000000000000000000000000000000000000000000000
0000000000000000000000000000000000000000000000000000000000000000000000000000000000000000000000000000000000000000000000000000000000000000000000000000000000000000000000000000000
00000000000000000
0000000000000000000000000000000000000000000000000000000000000000000000000000000000000000000000000000000000000000000000000000000000000000000000000000000000000000000000000000000
0000000000000000000000000000000000000000000000000000000000000000000000000000000000000000000000000000000000000000000000000000000000000000000000000000000000000000000000000000000
00000000000000000
0000000000000000000000000000000000000000000000000000000000000000000000000000000000000000000000000000000000000000000000000000000000000000000000000000000000000000000000000000000
0000000000000000000000000000000000000000000000000000000000000000000000000000000000000000000000000000000000000000000000000000000000000000000000000000000000000000000000000000000
00000000000000000
0000000000000000000000000000000000000000000000000000000000000000000000000000000000000000000000000000000000000000000000000000000000000000000000000000000000000000000000000000000
0000000000000000000000000000000000000000000000000000000000000000000000000000000000000000000000000000000000000000000000000000000000000000000000000000000000000000000000000000000
00000000000000000
>Q6B8P0
MSQSVEERTRIKNQRYESGVIPYAKMGYWDPNYVVKETDVLALFRVTPQPGVDPVEASAAVAGESSTATWTVVWTDLLTACDLYRAKAYKVDAVPNTTDQYFAFIAYDIDLFEEGSIANLTASIIGNVFGFKAVKALRLEDMRIPVAYLKTFQGPATGLIVERERMDKFGRPFLG
ATVKPKLGLSGKNYGRVVYEGLKGGLDFLKDDENINSQPFMRWKERFLYSMEAVNRAIAATGETKGHYMNVTAATMEEMYERAEFAKQLGSIIIMIDLVIGYTAIQTMAIWARKNDMILHLHRAGNSTYSRQKIHGMNFRVICKWMRMSGVDHIHAGTVVGKLEGDPLMIRGFYN
TLLQTHLEVNLPQGIFFEQDWASLRKVTPVASGGIHCGQMHQLLDYLGNDVVLQFGGGTIGHPDGIQAGATANRVALEAMVLARNEGRDYVAEGPQILRDAAKTCGPLQTALDLWKDITFNYTSTDTADFVETPTANV
0000000000000000000000000000000000000000000000000000000000000000000000000000000000000000000000000000000000000000000000000000000000000000000000000000000000000000000000000000000
0000000000000000000000000000000000000000000000000000000000000000000000000000000000000000000000000000000000000000000000000000000000000000000000000000000000000000000000000000000
000000000000000000000000000000000000000000000000000000000000000000000000000000000000000000000000000000000000000000000000000000000000000000
0000000000000000000000000000000000000000000000000000000000000000000000000000000000000000000000000000000000000000000000000000000000000000000000000000000000000000000000000000000
0000000000000000000000000000000000000000000000000000000000000000000000000000000000000000000000000000000000000000000000000000000000000000000000000000000000000000000000000000000
000000000000000000000000000000000000000000000000000000000000000000000000000000000000000000000000000000000000000000000000000000000000000000
0000000000000000000000000000000000000000000000000000000000000000000000000000000000000000000000000000000000000000000000000000000000000000000000000000000000000000000000000000000
0000000000000000000000000000000000000000000000000000000000000000000000000000000000000000000000000000000000000000000000000000000000000000000000000000000000000000000000000000000
000000000000000000000000000000000000000000000000000000000000000000000000000000000000000000000000000000000000000000000000000000000000000000
0000000000000000000000000000000000000000000000000000000000000000000000000000000000000000000000000000000000000000000000000000000000000000000000000000000000000000000000000000000
0000000000000000000000000000000000000000000000000000000000000000000000000000000000000000000000000000000000000000000000000000000000000000000000000000000000000000000000000000000
000000000000000000000000000000000000000000000000000000000000000000000000000000000000000000000000000000000000000000000000000000000000000000
0000000000000000000000000000000000000000000000000000000000000000000000000000000000000000000000000000000000000000000000000000000000000000000000000000000000000000000000000000000
0000000000000000000000000000000000000000000000000000000000000000000000000000000000000000000000000000000000000000000000000000000000000000000000000000000000000000000000000000000
000000000000000000000000000000000000000000000000000000000000000000000000000000000000000000000000000000000000000000000000000000000000000000
0000000000000000000000000000000000000000000000000000000000000000000000000000000000000000000000000000000000000000000000000000000000000000000000000000000000000000000000000000000
0000000000000000000000000000000000000000000000000000000000000000000000000000000000000000000000000000000000000000000000000000000000000000000000000000000000000000000000000000000
000000000000000000000000000000000000000000000000000000000000000000000000000000000000000000000000000000000000000000000000000000000000000000
0000000000000000000000000000000000000000000000000000000000000000000000000000000000000000000000000000000000000000000000000000000000000000000000000000000000000000000000000000000
0000000000000000000000000000000000000000000000000000000000000000000000000000000000000000000000000000000000000000000000000000000000000000000000000000000000000000000000000000000
000000000000000000000000000000000000000000000000000000000000000000000000000000000000000000000000000000000000000000000000000000000000000000
>Q6H3X3
MAAAASPAFLLRLPLLLLLSSWCRTGLADPHSLCYDITVIPKFRPGPRWCAVQGQVDEKTFLHYDCGSKTVTPVSPLGKKLNVTTAWKAQNPVLREVVDILTEQLLDIQLENYIPKEPLTLQARMSCEQKAEGHGSGSWQLSFDGQIFLLFDSENRMWTTVHPGARKMKEKWEND
KDMTMSFHYISMGDCTGWLEDFLMGMDSTLEPSAGAPPTMSSGTAQPRATATTLILCCLLIMCLLICSRHSLTQSHGHHPQSLQPPPHPPLLHPTWLLRRVLWSDSYQIAKRPLSGGHVTRVTLPIIGDDSHSLPCPLALYTINNGAARYSEPLQVSIS
0000000000000000000000000000000000000000000000000000000000000000000000000000000000000000000000000000000000000000000000000000000000000000000000000000000000000000000000000000000
000000000000000000000000000000000000000000000000000000000000000000000000000000000000000000000000000000000000000000000000000000000000000000000000000000000000000

0000000000000000000000000000000000000000000000000000000000000000000000000000000000000000000000000000000000000000000000000000000000000000000000000000000000000000000000000
000000000000000000000000000000000000000000000000000000000000000000000000000000000000000000000000000000000000000000000000000000000000000000000000000000000000000
0000000000000000000000000000000000000000000000000000000000000000000000000000000000000000000000000000000000000000000000000000000000000000000000000000000000000000000000000
000000000000000000000000000000000000000000000000000000000000000000000000000000000000000000000000000000000000000000000000000000000000000000000000000000000000000
0000000000000000000000000000000000000000000000000000000000000000000000000000000000000000000000000000000000000000000000000000000000000000000000000000000000000000000000000
000000000000000000000000000000000000000000000000000000000000000000000000000000000000000000000000000000000000000000000000000000000000000000000000000000000000000
0000000000000000000000000000000000000000000000000000000000000000000000000000000000000000000000000000000000000000000000000000000000000000000000000000000000000000000000000
000000000000000000000000000000000000000000000000000000000000000000000000000000000000000000000000000000000000000000000000000000000000000000000000000000000000000
0000000000000000000000000000000000000000000000000000000000000000000000000000000000000000000000000000000000000000000000000000000000000000000000000000000000000000000000000
000000000000000000000000000000000000000000000000000000000000000000000000000000000000000000000000000000000000000000000000000000000000000000000000000000000000000
0000000000000000000000000000000000000000000000000000000000000000000000000000000000000000000000000000000000000000000000000000000000000000000000000000000000000000000000000
000000000000000000000000000000000000000000000000000000000000000000000000000000000000000000000000000000000000000000000000000000000000000000000000000000000000000
>Q6NV75
MSDERRLPGSAVGWLVCGGLSLLANAWGILSVGAKQKKWKPLEFLLCTLAATHMLNVAVPIATYSVVQLRRQRPDFEWNEGLCKVFVSTFYTLTLATCFSVTSLSYHRMWMVCWPVNYRLSNAKKQAVHTVMGIWMVSFILSALPAVGWHDTSERFYTHGCRFIVAEIGLGFGVC
FLLLVGGSVAMGVICTAIALFQTLAVQVGRQADRRAFTVPTIVVEDAQGKRRSSIDGSEPAKTSLQTTGLVTTIVFIYDCLMGFPVLVVSFSSLRADASAPWMALCVLWCSVAQALLLPVFLWACDRYRADLKAVREKCMALMANDEESDDETSLEGGISPDLVLERSLDYGYGG
DFVALDRMAKYEISALEGGLPQLYPLRPLQEDKMQYLQVPPTRRFSHDDADVWAAVPLPAFLPRWGSGEDLAALAHLVLPAGPERRRASLLAFAEDAPPSRARRRSAESLLSLRPSALDSGPRGARDSPPGSPRRRPGPGPRSASASLLPDAFALTAFECEPQALRRPPGPFPAA
PAAPDGADPGEAPTPPSSAQRSPGPRPSAHSHAGSLRPGLSASWGEPGGLRAAGGGGSTSSFLSSPSESSGYATLHSDSLGSAS
0000000000000000000000000000000000000000000000000000000000000000000000000000000000000000000000000000000000000000000000000000000000000000000000000000000000000000000000000
0000000000000000000000000000000000000000000000000000000000000000000000000000000000000000000000000000000000000000000000000000000000000000000000000000000000000000000000000
0000000000000000000000000000000000000000000000000000000000000000000000000000000000000000000000000000000000000000000000000000000000000000000000000000000000000000000000000
000000000000000000000000000000000000000000000000000000000000000000000000000000000000
0000000000000000000000000000000000000000000000000000000000000000000000000000000000000000000000000000000000000000000000000000000000000000000000000000000000000000000000000
0000000000000000000000000000000000000000000000000000000000000000000000000000000000000000000000000000000000000000000000000000000000000000000000000000000000000000000000000
0000000000000000000000000000000000000000000000000000000000000000000000000000000000000000000000000000000000000000000000000000000000000000000000000000000000000000000000000
000000000000000000000000000000000000000000000000000000000000000000000000000000000000
0000000000000000000000000000000000000000000000000000000000000000000000000000000000000000000000000000000000000000000000000000000000000000000000000000000000000000000000000
0000000000000000000000000000000000000000000000000000000000000000000000000000000000000000000000000000000000000000000000000000000000000000000000000000000000000000000000000
0000000000000000000000000000000000000000000000000000000000000000000000000000000000000000000000000000000000000000000000000000000000000000000000000000000000000000000000000
000000000000000000000000000000000000000000000000000000000000000000000000000000000000
0000000000000000000000000000000000000000000000000000000000000000000000000000000000000000000000000000000000000000000000000000000000000000000000000000000000000000000000000
0000000000000000000000000000000000000000000000000000000000000000000000000000000000000000000000000000000000000000000000000000000000000000000000000000000000000000000000000
0000000000000000000000000000000000000000000000000000000000000000000000000000000000000000000000000000000000000000000000000000000000000000000000000000000000000000000000000
000000000000000000000000000000000000000000000000000000000000000000000000000000000000
0000000000000000000000000000000000000000000000000000000000000000000000000000000000000000000000000000000000000000000000000000000000000000000000000000000000000000000000000
0000000000000000000000000000000000000000000000000000000000000000000000000000000000000000000000000000000000000000000000000000000000000000000000000000000000000000000000000
0000000000000000000000000000000000000000000000000000000000000000000000000000000000000000000000000000000000000000000000000000000000000000000000000000000000000000000000000
000000000000000000000000000000000000000000000000000000000000000000000000000000000000
0000000000000000000000000000000000000000000000000000000000000000000000000000000000000000000000000000000000000000000000000000000000000000000000000000000000000000000000000
0000000000000000000000000000000000000000000000000000000000000000000000000000000000000000000000000000000000000000000000000000000000000000000000000000000000000000000000000
0000000000000000000000000000000000000000000000000000000000000000000000000000000000000000000000000000000000000000000000000000000000000000000000000000000000000000000000000
000000000000000000000000000000000000000000000000000000000000000000000000000000000000
0000000000000000000000000000000000000000000000000000000000000000000000000000000000000000000000000000000000000000000000000000000000000000000000000000000000000000000000000
0000000000000000000000000000000000000000000000000000000000000000000000000000000000000000000000000000000000000000000000000000000000000000000000000000000000000000000000000
0000000000000000000000000000000000000000000000000000000000000000000000000000000000000000000000000000000000000000000000000000000000000000000000000000000000000000000000000
000000000000000000000000000000000000000000000000000000000000000000000000000000000000
>Q7TXJ8
MEETSVAGDPGPDAGTSTAPNAAPEPVARRQRILFVGEAATLAHVVRPFVLARSLDPSRYEVHFACDPRFNKLLGPLPFPHHPIHTVPSEEVLLKIAQGRLFYNTRTLRKYIAADRKILNEIAPDVVVGDNRLSLSVSARLAGIPYIAIANAYWSPQARRRFPLPDVPWTRFFGV
RPVSILYRLYRPLIFALYCLPLNWLRRKHGLSSLGWDLCRIFTDGDYTLYADVPELVPTYNLPANHRYLGPVLWSPDVKPPTWWHSLPTDRPIIYATLGSSGGKNLLQVVLNALGRFTRDGDRGHRWPEPPEERAGQRLRRGLPAGRSGCSALRRGALQRRQPDDAAGVGGRGAG
DRAPQQHGPALEHGGP
0000000000000000000000000000000000000000000000000000000000000000000000000000000000000000000000000000000000000000000000000000000000000000000000000000000000000000000000000
0000000000000000000000000000000000000000000000000000000000000000000000000000000000000000000000000000000000000000000000000000000000000000000000000000000000000000000000000
0000000000000000
0000000000000000000000000000000000000000000000000000000000000000000000000000000000000000000000000000000000000000000000000000000000000000000000000000000000000000000000000
0000000000000000000000000000000000000000000000000000000000000000000000000000000000000000000000000000000000000000000000000000000000000000000000000000000000000000000000000
0000000000000000
0000000000000000000000000000000000000000000000000000000000000000000000000000000000000000000000000000000000000000000000000000000000000000000000000000000000000000000000000
0000000000000000000000000000000000000000000000000000000000000000000000000000000000000000000000000000000000000000000000000000000000000000000000000000000000000000000000000
0000000000000000
0000000000000000000000000000000000000000000000000000000000000000000000000000000000000000000000000000000000000000000000000000000000000000000000000000000000000000000000000
0000000000000000000000000000000000000000000000000000000000000000000000000000000000000000000000000000000000000000000000000000000000000000000000000000000000000000000000000
0000000000000000
0000000000000000000000000000000000000000000000000000000000000000000000000000000000000000000000000000000000000000000000000000000000000000000000000000000000000000000000000
0000000000000000000000000000000000000000000000000000000000000000000000000000000000000000000000000000000000000000000000000000000000000000000000000000000000000000000000000
0000000000000000
0000000000000000000000000000000000000000000000000000000000000000000000000000000000000000000000000000000000000000000000000000000000000000000000000000000000000000000000000
0000000000000000000000000000000000000000000000000000000000000000000000000000000000000000000000000000000000000000000000000000000000000000000000000000000000000000000000000
0000000000000000

0000000000000000000000000000000000000000000000000000000000000000000000000000000000000000000000000000000000000000000000000000000000000000000000000000000000000000000000000000000
0000000000000000000000000000000000000000000000000000000000000000000000000000000000000000000000000000000000000000000000000000000000000000000000000000000000000000000000000000000
0000000000000000
>Q7XJS0
MADLQRFLQDRCLGVSNLPQKGRSLFTARDFRPGEVILSQKPYICVPNNTSSESRCDGCFKTNNLKKCSACQVVWYCGSSCQKSEWKLHRDECKALTRLEKEKRKFVTPTIRLMVRLYIKRNLQNEKVLPITTTDNYSLVEALVSHMSEIDEKQMLLYAQMANLVNLILQFPSVD
LREIAENFSKFSCNAHSICDSELRPQGIGLFPLVSIINHSCSPNAVLVFEEQMAVVRAMDNISKDSEITISYIETAGSTLTRQKSLKEQYLFHCQCARCSNFGKPHDIEESAILEGYRCANEKCTGFLLRDPEEKGFVCQKCLLLRSKEEVKKLASDLKTVSEKAPTSPSAEDKQ
AAIELYKTIEKLQVKLYHSFSIPLMRTREKLLKMLMDVEIWREALNYCRLIVPVYQRVYPATHPLIGLQFYTQGKLEWLLGETKEAVSSLIKAFDILRISHGISTPFMKELSAKLEEARAEASYKQLALH
0000000000000000000000000000000000000000000000000000000000000000000000000000000000000000000000000000000000000000000000000000000000000000000000000000000000000000000000000000000
0000000000000000000000000000000000000000000000000000000000000000000000000000000000000000000000000000000000000000000000000000000000000000000000000000000000000000000000000000000
0000000000000000000000000000000000000000000000000000000000000000000000000000000000000000000000000000000000000000000000000000000000
0000000000000000000000000000000000000000000000000000000000000000000000000000000000000000000000000000000000000000000000000000000000000000000000000000000000000000000000000000000
0000000000000000000000000000000000000000000000000000000000000000000000000000000000000000000000000000000000000000000000000000000000000000000000000000000000000000000000000000000
0000000000000000000000000000000000000000000000000000000000000000000000000000000000000000000000000000000000000000000000000000000000
0000000000000000000000000000000000000000000000000000000000000000000000000000000000000000000000000000000000000000000000000000000000000000000000000000000000000000000000000000000
0000000000000000000000000000000000000000000000000000000000000000000000000000000000000000000000000000000000000000000000000000000000000000000000000000000000000000000000000000000
0000000000000000000000000000000000000000000000000000000000000000000000000000000000000000000000000000000000000000000000000000000000
0000000000000000000000000000000000000000000000000000000000000000000000000000000000000000000000000000000000000000000000000000000000000000000000000000000000000000000000000000000
0000000000000000000000000000000000000000000000000000000000000000000000000000000000000000000000000000000000000000000000000000000000000000000000000000000000000000000000000000000
0000000000000000000000000000000000000000000000000000000000000000000000000000000000000000000000000000000000000000000000000000000000
0000000000000000000000000000000000000000000000000000000000000000000000000000000000000000000000000000000000000000000000000000000000000000000000000000000000000000000000000000000
0000000000000000000000000000000000000000000000000000000000000000000000000000000000000000000000000000000000000000000000000000000000000000000000000000000000000000000000000000000
0000000000000000000000000000000000000000000000000000000000000000000000000000000000000000000000000000000000000000000000000000000000
0000000000000000000000000000000000000000000000000000000000000000000000000000000000000000000000000000000000000000000000000000000000000000000000000000000000000000000000000000000
0000000000000000000000000000000000000000000000000000000000000000000000000000000000000000000000000000000000000000000000000000000000000000000000000000000000000000000000000000000
0000000000000000000000000000000000000000000000000000000000000000000000000000000000000000000000000000000000000000000000000000000000
0000000000000000000000000000000000000000000000000000000000000000000000000000000000000000000000000000000000000000000000000000000000000000000000000000000000000000000000000000000
0000000000000000000000000000000000000000000000000000000000000000000000000000000000000000000000000000000000000000000000000000000000000000000000000000000000000000000000000000000
0000000000000000000000000000000000000000000000000000000000000000000000000000000000000000000000000000000000000000000000000000000000
>Q80X86
MGKTIPRFLEQLDLIKSFVGLATGAGALYLLYKAVRTGLKCHPPLCSNSPICIARLAIERERHGRDSGEIRRLLNSLDCKQDEYTRSMILHNITRCVYLLEAEASSCTMDDIDLVADMLDEKDNSVKIQALNALKAFSGIRKFRLKIQEHCIKVLELISTIWDLELHVAGLRLLN
NLPLPDYVHPQLRRVMPALMEIIQSDCILAQVQAVRLLSYLAQKNDLLYDILNCQVRPNFLNLFQSSQPGSLLFEVLVFAEHLSEGRNATHYRAVKWHYNEQSLHEALFGDESRLADRLLSLVIHPEEEVQIQACKVIVSLQCPQDLGSRPSSCRPSHSCFKTGK
0000000000000000000000000000000000000000000000000000000000000000000000000000000000000000000000000000000000000000000000000000000000000000000000000000000000000000000000000000000
000000000000000000000000000000000000000000000000000000000000000000000000000000000000000000000000000000000000000000000000000000000000000000000000000000000000000000000
0000000000000000000000000000000000000000000000000000000000000000000000000000000000000000000000000000000000000000000000000000000000000000000000000000000000000000000000000000000
000000000000000000000000000000000000000000000000000000000000000000000000000000000000000000000000000000000000000000000000000000000000000000000000000000000000000000000
0000000000000000000000000000000000000000000000000000000000000000000000000000000000000000000000000000000000000000000000000000000000000000000000000000000000000000000000000000000
000000000000000000000000000000000000000000000000000000000000000000000000000000000000000000000000000000000000000000000000000000000000000000000000000000000000000000000
0000000000000000000000000000000000000000000000000000000000000000000000000000000000000000000000000000000000000000000000000000000000000000000000000000000000000000000000000000000
000000000000000000000000000000000000000000000000000000000000000000000000000000000000000000000000000000000000000000000000000000000000000000000000000000000000000000000
0000000000000000000000000000000000000000000000000000000000000000000000000000000000000000000000000000000000000000000000000000000000000000000000000000000000000000000000000000000
000000000000000000000000000000000000000000000000000000000000000000000000000000000000000000000000000000000000000000000000000000000000000000000000000000000000000000000
0000000000000000000000000000000000000000000000000000000000000000000000000000000000000000000000000000000000000000000000000000000000000000000000000000000000000000000000000000000
000000000000000000000000000000000000000000000000000000000000000000000000000000000000000000000000000000000000000000000000000000000000000000000000000000000000000000000
0000000000000000000000000000000000000000000000000000000000000000000000000000000000000000000000000000000000000000000000000000000000000000000000000000000000000000000000000000000
000000000000000000000000000000000000000000000000000000000000000000000000000000000000000000000000000000000000000000000000000000000000000000000000000000000000000000000
>Q8FP19
MSMQPTDHIRLTAFVHGHVQGVGFRWWTRSQALELGLAGSATNLDDGRVCVVAEGPEDRCRELLTRLGEQPSRHRRVGTVSTVIEQWSEPRGVEGFTER
000000000000000000000000000000000000000000000000000000000000000000000000000000000000000000000000000
000000000000000000000000000000000000000000000000000000000000000000000000000000000000000000000000000
000000000000000000000000000000000000000000000000000000000000000000000000000000000000000000000000000
000000000000000000000000000000000000000000000000000000000000000000000000000000000000000000000000000
000000000000000000000000000000000000000000000000000000000000000000000000000000000000000000000000000
000000000000000000000000000000000000000000000000000000000000000000000000000000000000000000000000000
000000000000000000000000000000000000000000000000000000000000000000000000000000000000000000000000000
>Q8K4S3
MIPPAGSTPPGEALIPSVAPQDFWRSPISGYSGSVTRHISHRANNFKRHPKRRKYIRPSPPPPPNTPCPIELVDFEDLHPQRSFWELLFNGCILFGIEFSYAMETAYVTPVLLQMGLPDQLYSLVWFISPILGFLLQPLLGAWSDRCTSRFGRRRPFILVLAIGALLGLSLLLNG
RDIGMALADTATNHKWGILLTVCGVVLMDFSADSADNPSHAYMMDVCGPVDQDRGLNIHALMAGLGGGFGYVVGGIHWDKTSFGRALGGQLRVIYIFTAITLSVTTVFTLVSIPERPLRPLGEKRTAMKSPSLPLPPSPPVLLEEGAGDTLPSTTATSLYASFSSPISPPSPLTP
KYGSFISRDSSLTGINEFASSFGTSNIDSVLIDCFTAGHDNYLALPSSVPRQAISVSFPRAPDGFYCQERGLERREGPLTLGLDGDVLRVGSLDTSKPRASGILKRPQTLALPDVAGGNGPETSRRRNVTFSQQVANILLNGVKYESELTGSSEQSEQPLSLRRLCSTIYNMPRP
VRNLCVNHFLGWLSFEGMLLFYTDFMGEVVFQGDPKAPHASEAYQKYNSGVTMGCWGMCIYAFSAAFYSAILEKLEECLSVRTLYFIAYLLFGLGTGLATLSRNLYVVLSLCTHYGILFSTLCTLPYSLLCDYYQSKKFAGSSADGTRRGMGVDISLLSCQYFLAQILVSLVLGP
LTSAVGSANGVMYFASLVSFLGCLYSSLCVTYEIPSADAADEERQPLLLNV
0000000000000000000000000000000000000000000000000000000000000000000000000000000000000000000000000000000000000000000000000000000000000000000000000000000000000000000000000000000
0000000000000000000000000000000000000000000000000000000000000000000000000000000000000000000000000000000000000000000000000000000000000000000000000000000000000000000000000000000
0000000000000000000000000000000000000000000000000000000000000000000000000000000000000000000000000000000000000000000000000000000000000000000000000000000000000000000000000000000
0000000000000000000000000000000000000000000000000000000000000000000000000000000000000000000000000000000000000000000000000000000000000000000000000000000000000000000000000000000
000000000000000000000000000000000000000000000000000
0000000000000000000000000000000000000000000000000000000000000000000000000000000000000000000000000000000000000000000000000000000000000000000000000000000000000000000000000000000
0000000000000000000000000000000000000000000000000000000000000000000000000000000000000000000000000000000000000000000000000000000000000000000000000000000000000000000000000000000
0000000000000000000000000000000000000000000000000000000000000000000000000000000000000000000000000000000000000000000000000000000000000000000000000000000000000000000000000000000

0000000000000000000000000000000000000000000000000000000000000000000000000000000000000000000000000000000000000000000000000000000000000000000000000000000000000000000000000000000
000000000000000000000000000000000000000000000000000
0000000000000000000000000000000000000000000000000000000000000000000000000000000000000000000000000000000000000000000000000000000000000000000000000000000000000000000000000000000
0000000000000000000000000000000000000000000000000000000000000000000000000000000000000000000000000000000000000000000000000000000000000000000000000000000000000000000000000000000
0000000000000000000000000000000000000000000000000000000000000000000000000000000000000000000000000000000000000000000000000000000000000000000000000000000000000000000000000000000
0000000000000000000000000000000000000000000000000000000000000000000000000000000000000000000000000000000000000000000000000000000000000000000000000000000000000000000000000000000
000000000000000000000000000000000000000000000000000
0000000000000000000000000000000000000000000000000000000000000000000000000000000000000000000000000000000000000000000000000000000000000000000000000000000000000000000000000000000
0000000000000000000000000000000000000000000000000000000000000000000000000000000000000000000000000000000000000000000000000000000000000000000000000000000000000000000000000000000
0000000000000000000000000000000000000000000000000000000000000000000000000000000000000000000000000000000000000000000000000000000000000000000000000000000000000000000000000000000
0000000000000000000000000000000000000000000000000000000000000000000000000000000000000000000000000000000000000000000000000000000000000000000000000000000000000000000000000000000
000000000000000000000000000000000000000000000000000
0000000000000000000000000000000000000000000000000000000000000000000000000000000000000000000000000000000000000000000000000000000000000000000000000000000000000000000000000000000
0000000000000000000000000000000000000000000000000000000000000000000000000000000000000000000000000000000000000000000000000000000000000000000000000000000000000000000000000000000
0000000000000000000000000000000000000000000000000000000000000000000000000000000000000000000000000000000000000000000000000000000000000000000000000000000000000000000000000000000
0000000000000000000000000000000000000000000000000000000000000000000000000000000000000000000000000000000000000000000000000000000000000000000000000000000000000000000000000000000
000000000000000000000000000000000000000000000000000
0000000000000000000000000000000000000000000000000000000000000000000000000000000000000000000000000000000000000000000000000000000000000000000000000000000000000000000000000000000
0000000000000000000000000000000000000000000000000000000000000000000000000000000000000000000000000000000000000000000000000000000000000000000000000000000000000000000000000000000
0000000000000000000000000000000000000000000000000000000000000000000000000000000000000000000000000000000000000000000000000000000000000000000000000000000000000000000000000000000
0000000000000000000000000000000000000000000000000000000000000000000000000000000000000000000000000000000000000000000000000000000000000000000000000000000000000000000000000000000
000000000000000000000000000000000000000000000000000
0000000000000000000000000000000000000000000000000000000000000000000000000000000000000000000000000000000000000000000000000000000000000000000000000000000000000000000000000000000
0000000000000000000000000000000000000000000000000000000000000000000000000000000000000000000000000000000000000000000000000000000000000000000000000000000000000000000000000000000
0000000000000000000000000000000000000000000000000000000000000000000000000000000000000000000000000000000000000000000000000000000000000000000000000000000000000000000000000000000
0000000000000000000000000000000000000000000000000000000000000000000000000000000000000000000000000000000000000000000000000000000000000000000000000000000000000000000000000000000
000000000000000000000000000000000000000000000000000
>Q8N957
MEASLTRRLLFKDRHFTCSKIIGRRFACFAQRLSHRRKQSQCDLLNESTGQLPTTCSSAASNSINWNCRVKMTQQMQNLHLCQSKKHSAPSSPNAAKRLYRNLSEKLKGSHSSFDEAYFRTRTDRLSLRKTSVNFQGNEAMFEAVEQQDMDAVQILLYQYTPEELDLNTPNSEGL
TPLDIAIMTNNVPIARILLRTGARESPHFVSLESRAMHLNTLVQEAQERVSELSAQVENEGFTLDNTEKEKQLKAWEWRYRLYRRMKTGFEHARAPEMPTNVCLMVTSSTSLTVSFQEPLSVNAAVVTRYKVEWSMSEDFSPLAGEIIMDNLQTLRCTITGLTMGQQYFVQVSAY
NMKGWGPAQTTTPACASPSNWKDYDDREPRHKGQSEVLEGLLQQVRALHQHYSCRESTKLQTTGRKQSVSRSLKHLFHSSNKFVKTLKRGLYIAVIFYYKDNILVTNEDQVPIVEIDDSHTSSITQDFLWFTKLSCMWEDIRWLRQSIPISSSSSTVLQTRQKMLAATAQLQNLL
GTHNLGRVYYEPIKDRHGNILIVTIREVEMLYSFFNGKWMQISKLQSQRKSLSTPEEPTALDILLITIQDILSYHKRSHQRLFPGLYLGYLKLCSSVDQIKVLVTQKLPNILCHVKIRENNNISREEWEWIQKLSGSESMESVDHTSDCPMQLFFYELQMAVKALLQQINIPLHQ
ARNFRLYTQEVLEMGHNVSFLLLLPASDDVCTAPGQNNPYTPHSGFLNLPLQMFELGIVACFT
0000000000000000000000000000000000000000000000000000000000000000000000000000000000000000000000000000000000000000000000000000000000000000000000000000000000000000000000000000000
0000000000000000000000000000000000000000000000000000000000000000000000000000000000000000000000000000000000000000000000000000000000000000000000000000000000000000000000000000000
0000000000000000000000000000000000000000000000000000000000000000000000000000000000000000000000000000000000000000000000000000000000000000000000000000000000000000000000000000000
0000000000000000000000000000000000000000000000000000000000000000000000000000000000000000000000000000000000000000000000000000000000000000000000000000000000000000000000000000000
000000000000000000000000000000000000000000000000000000000000000
0000000000000000000000000000000000000000000000000000000000000000000000000000000000000000000000000000000000000000000000000000000000000000000000000000000000000000000000000000000
0000000000000000000000000000000000000000000000000000000000000000000000000000000000000000000000000000000000000000000000000000000000000000000000000000000000000000000000000000000
0000000000000000000000000000000000000000000000000000000000000000000000000000000000000000000000000000000000000000000000000000000000000000000000000000000000000000000000000000000
0000000000000000000000000000000000000000000000000000000000000000000000000000000000000000000000000000000000000000000000000000000000000000000000000000000000000000000000000000000
000000000000000000000000000000000000000000000000000000000000000
0000000000000000000000000000000000000000000000000000000000000000000000000000000000000000000000000000000000000000000000000000000000000000000000000000000000000000000000000000000
0000000000000000000000000000000000000000000000000000000000000000000000000000000000000000000000000000000000000000000000000000000000000000000000000000000000000000000000000000000
0000000000000000000000000000000000000000000000000000000000000000000000000000000000000000000000000000000000000000000000000000000000000000000000000000000000000000000000000000000
0000000000000000000000000000000000000000000000000000000000000000000000000000000000000000000000000000000000000000000000000000000000000000000000000000000000000000000000000000000
000000000000000000000000000000000000000000000000000000000000000
0000000000000000000000000000000000000000000000000000000000000000000000000000000000000000000000000000000000000000000000000000000000000000000000000000000000000000000000000000000
0000000000000000000000000000000000000000000000000000000000000000000000000000000000000000000000000000000000000000000000000000000000000000000000000000000000000000000000000000000
0000000000000000000000000000000000000000000000000000000000000000000000000000000000000000000000000000000000000000000000000000000000000000000000000000000000000000000000000000000
0000000000000000000000000000000000000000000000000000000000000000000000000000000000000000000000000000000000000000000000000000000000000000000000000000000000000000000000000000000
000000000000000000000000000000000000000000000000000000000000000
0000000000000000000000000000000000000000000000000000000000000000000000000000000000000000000000000000000000000000000000000000000000000000000000000000000000000000000000000000000
0000000000000000000000000000000000000000000000000000000000000000000000000000000000000000000000000000000000000000000000000000000000000000000000000000000000000000000000000000000
0000000000000000000000000000000000000000000000000000000000000000000000000000000000000000000000000000000000000000000000000000000000000000000000000000000000000000000000000000000
0000000000000000000000000000000000000000000000000000000000000000000000000000000000000000000000000000000000000000000000000000000000000000000000000000000000000000000000000000000
000000000000000000000000000000000000000000000000000000000000000
0000000000000000000000000000000000000000000000000000000000000000000000000000000000000000000000000000000000000000000000000000000000000000000000000000000000000000000000000000000
0000000000000000000000000000000000000000000000000000000000000000000000000000000000000000000000000000000000000000000000000000000000000000000000000000000000000000000000000000000
0000000000000000000000000000000000000000000000000000000000000000000000000000000000000000000000000000000000000000000000000000000000000000000000000000000000000000000000000000000
0000000000000000000000000000000000000000000000000000000000000000000000000000000000000000000000000000000000000000000000000000000000000000000000000000000000000000000000000000000
000000000000000000000000000000000000000000000000000000000000000
0000000000000000000000000000000000000000000000000000000000000000000000000000000000000000000000000000000000000000000000000000000000000000000000000000000000000000000000000000000
0000000000000000000000000000000000000000000000000000000000000000000000000000000000000000000000000000000000000000000000000000000000000000000000000000000000000000000000000000000
0000000000000000000000000000000000000000000000000000000000000000000000000000000000000000000000000000000000000000000000000000000000000000000000000000000000000000000000000000000
0000000000000000000000000000000000000000000000000000000000000000000000000000000000000000000000000000000000000000000000000000000000000000000000000000000000000000000000000000000
000000000000000000000000000000000000000000000000000000000000000
>Q8S2T0

MSEDKESDVVGEEEESHVIKEDKELNDASNETLTENGDQLLQMIAELRLENDFLRSQFEGLKDEVAQGRSLQKAEQVEADSAQLKQLQEQVASLSREIDVEKQTRVAAEQALEHLREAYSEADAKSQEYSSKFSQVEQKLDQEIKERDEKYADLDAKFTRLHKRAKQRIQEIQKE
KDDLDARFREVNETAERASSQHSSMQQELERTRQQANEALKAMDAERQQLRSANNKLRDTIEELRGSLQPKENKIETLQQSLLDKDQILEDLKKQLQAVEERKQIAVTELSAKHQKNLEGLEAQVVDALSERDKAAETISSLQVLLAEKESKIAEMEAAATGEAARLRAAAETLK
GELAHLKSENEKEKETWEASCDALKSKLEIAESNYLQAEIEVAKMRSQLGSEMSMQTQILSTKDAELKGAREEINRLQSEFSSYKIRAHALLQKKDMELAAAKDSEQIKSLEEALKEAEKEVYLVSAERDRAQQDLQSALASLEKELEERAGALKDASEQIKSLEVKLDSTVARN
QAEKQAWEEDLRVLEETWRRRCEALTAQNEASPAEGIEKELENAKLRNKRMKEEHESVRELADRLIEEKDREISRLVDEMTNLRKSMESKPVWNKSPSQVHHYGNNNTESQQQDVSNLSTSAAEHQILILARQQAQREEELAQTQRHILALQEEIEELERENRLHSQQEAVLKTE
LREMERKQKREGVDMTYLKNVILKLLETGEVEALLPVVGMLLQFSPEEIQKCQQAYHSSTTAATTEATPSPASEGSGLSVFSRFSFS
0000000000000000000000000000000000000000000000000000000000000000000000000000000000000000000000000000000000000000000000000000000000000000000000000000000000000000000000000000000
0000000000000000000000000000000000000000000000000000000000000000000000000000000000000000000000000000000000000000000000000000000000000000000000000000000000000000000000000000000
0000000000000000000000000000000000000000000000000000000000000000000000000000000000000000000000000000000000000000000000000000000000000000000000000000000000000000000000000000000
0000000000000000000000000000000000000000000000000000000000000000000000000000000000000000000000000000000000000000000000000000000000000000000000000000000000000000000000000000000
000000000000000000000000000000000000000000000000000000000000000000000000000000000000000
0000000000000000000000000000000000000000000000000000000000000000000000000000000000000000000000000000000000000000000000000000000000000000000000000000000000000000000000000000000
0000000000000000000000000000000000000000000000000000000000000000000000000000000000000000000000000000000000000000000000000000000000000000000000000000000000000000000000000000000
0000000000000000000000000000000000000000000000000000000000000000000000000000000000000000000000000000000000000000000000000000000000000000000000000000000000000000000000000000000
0000000000000000000000000000000000000000000000000000000000000000000000000000000000000000000000000000000000000000000000000000000000000000000000000000000000000000000000000000000
000000000000000000000000000000000000000000000000000000000000000000000000000000000000000
0000000000000000000000000000000000000000000000000000000000000000000000000000000000000000000000000000000000000000000000000000000000000000000000000000000000000000000000000000000
0000000000000000000000000000000000000000000000000000000000000000000000000000000000000000000000000000000000000000000000000000000000000000000000000000000000000000000000000000000
0000000000000000000000000000000000000000000000000000000000000000000000000000000000000000000000000000000000000000000000000000000000000000000000000000000000000000000000000000000
0000000000000000000000000000000000000000000000000000000000000000000000000000000000000000000000000000000000000000000000000000000000000000000000000000000000000000000000000000000
000000000000000000000000000000000000000000000000000000000000000000000000000000000000000
0000000000000000000000000000000000000000000000000000000000000000000000000000000000000000000000000000000000000000000000000000000000000000000000000000000000000000000000000000000
0000000000000000000000000000000000000000000000000000000000000000000000000000000000000000000000000000000000000000000000000000000000000000000000000000000000000000000000000000000
0000000000000000000000000000000000000000000000000000000000000000000000000000000000000000000000000000000000000000000000000000000000000000000000000000000000000000000000000000000
0000000000000000000000000000000000000000000000000000000000000000000000000000000000000000000000000000000000000000000000000000000000000000000000000000000000000000000000000000000
000000000000000000000000000000000000000000000000000000000000000000000000000000000000000
0000000000000000000000000000000000000000000000000000000000000000000000000000000000000000000000000000000000000000000000000000000000000000000000000000000000000000000000000000000
0000000000000000000000000000000000000000000000000000000000000000000000000000000000000000000000000000000000000000000000000000000000000000000000000000000000000000000000000000000
0000000000000000000000000000000000000000000000000000000000000000000000000000000000000000000000000000000000000000000000000000000000000000000000000000000000000000000000000000000
0000000000000000000000000000000000000000000000000000000000000000000000000000000000000000000000000000000000000000000000000000000000000000000000000000000000000000000000000000000
000000000000000000000000000000000000000000000000000000000000000000000000000000000000000
0000000000000000000000000000000000000000000000000000000000000000000000000000000000000000000000000000000000000000000000000000000000000000000000000000000000000000000000000000000
0000000000000000000000000000000000000000000000000000000000000000000000000000000000000000000000000000000000000000000000000000000000000000000000000000000000000000000000000000000
0000000000000000000000000000000000000000000000000000000000000000000000000000000000000000000000000000000000000000000000000000000000000000000000000000000000000000000000000000000
0000000000000000000000000000000000000000000000000000000000000000000000000000000000000000000000000000000000000000000000000000000000000000000000000000000000000000000000000000000
000000000000000000000000000000000000000000000000000000000000000000000000000000000000000
0000000000000000000000000000000000000000000000000000000000000000000000000000000000000000000000000000000000000000000000000000000000000000000000000000000000000000000000000000000
0000000000000000000000000000000000000000000000000000000000000000000000000000000000000000000000000000000000000000000000000000000000000000000000000000000000000000000000000000000
0000000000000000000000000000000000000000000000000000000000000000000000000000000000000000000000000000000000000000000000000000000000000000000000000000000000000000000000000000000
0000000000000000000000000000000000000000000000000000000000000000000000000000000000000000000000000000000000000000000000000000000000000000000000000000000000000000000000000000000
000000000000000000000000000000000000000000000000000000000000000000000000000000000000000
>Q8SWI4
MVRRSLYFLAVMGVVRSSSGLYIPSVVLQELGIASSQGCLMIAETNNGNFGIVSSGLENPVYITESPQGHREVSWQPVRGEDRAVPIYAPSAEEVRESISSVRGSEPGQYIAPQPTGFVPASTPVFGTIESASTAGAAVPVEGVFVASTENPASTGSSSTSTCPPKGTAGTTDNK
GKAGGAAADDKSKSSSSSSSKKKKKGAKSLVALGAVATTALFSIVM
0000000000000000000000000000000000000000000000000000000000000000000000000000000000000000000000000000000000000000000000000000000000000000000000000000000000000000000000000000000
0000000000000000000000000000000000000000000000
0000000000000000000000000000000000000000000000000000000000000000000000000000000000000000000000000000000000000000000000000000000000000000000000000000000000000000000000000000000
0000000000000000000000000000000000000000000000
0000000000000000000000000000000000000000000000000000000000000000000000000000000000000000000000000000000000000000000000000000000000000000000000000000000000000000000000000000000
0000000000000000000000000000000000000000000000
0000000000000000000000000000000000000000000000000000000000000000000000000000000000000000000000000000000000000000000000000000000000000000000000000000000000000000000000000000000
0000000000000000000000000000000000000000000000
0000000000000000000000000000000000000000000000000000000000000000000000000000000000000000000000000000000000000000000000000000000000000000000000000000000000000000000000000000000
0000000000000000000000000000000000000000000000
0000000000000000000000000000000000000000000000000000000000000000000000000000000000000000000000000000000000000000000000000000000000000000000000000000000000000000000000000000000
0000000000000000000000000000000000000000000000
0000000000000000000000000000000000000000000000000000000000000000000000000000000000000000000000000000000000000000000000000000000000000000000000000000000000000000000000000000000
0000000000000000000000000000000000000000000000
>Q91G84
MNNFNYFNGKMVEDILENPDEDILNPDKSKTKDIVIKEDFCGACLALPLAFAGAGTATATSGDTSGNKSKSSIFFWSVVISIIGLIATVWFLSGDCTTCVSEGNSRGKRTGSMVCSSTRR
000000000000000000000000000000000000000000000000000000000000000000000000000000000000000000000000000000000000000000000000
000000000000000000000000000000000000000000000000000000000000000000000000000000000000000000000000000000000000000000000000
000000000000000000000000000000000000000000000000000000000000000000000000000000000000000000000000000000000000000000000000
000000000000000000000000000000000000000000000000000000000000000000000000000000000000000000000000000000000000000000000000
000000000000000000000000000000000000000000000000000000000000000000000000000000000000000000000000000000000000000000000000
000000000000000000000000000000000000000000000000000000000000000000000000000000000000000000000000000000000000000000000000
000000000000000000000000000000000000000000000000000000000000000000000000000000000000000000000000000000000000000000000000
>Q92JM2
MNCPLSLQIVNVSYIVNTNSCSWIAFNNSKYPIKTIKINIININILGKINHMVIFCDNNIVIILWKIMVVIISSIIHRTYIRRWISRRNNIRRKASHACKQPNNTTGC
000000000000000000000000000000000000000000000000000000000000000000000000000000000000000000000000000000000000

000000000000000000000000000000000000000000000000000000000000000000000000000000000000000000000000000000000000
000000000000000000000000000000000000000000000000000000000000000000000000000000000000000000000000000000000000
000000000000000000000000000000000000000000000000000000000000000000000000000000000000000000000000000000000000
000000000000000000000000000000000000000000000000000000000000000000000000000000000000000000000000000000000000
000000000000000000000000000000000000000000000000000000000000000000000000000000000000000000000000000000000000
000000000000000000000000000000000000000000000000000000000000000000000000000000000000000000000000000000000000
>Q92QK6
MDISRAEQRILHHLAQGGRIEITREGKAIAEIRCFTRDGWVYPGVDLELFRKLKRKRAIKSSAGKPYRITERGLHLVRSELNNR
000000000000000000000000000000000000000000000000000000000000000000000000000000000000
000000000000000000000000000000000000000000000000000000000000000000000000000000000000
000000000000000000000000000000000000000000000000000000000000000000000000000000000000
000000000000000000000000000000000000000000000000000000000000000000000000000000000000
000000000000000000000000000000000000000000000000000000000000000000000000000000000000
000000000000000000000000000000000000000000000000000000000000000000000000000000000000
000000000000000000000000000000000000000000000000000000000000000000000000000000000000
>Q9H079
MASETHNVKKRNFCNKIEDHFIDLPRKKISNFTNKNMKEVKKSPKQLAAYINRTVGQTVKSPDKLRKVIYRRKKVHHPFPNPCYRKKQSPGSGGCDMANKENELACAGHLPEKLHHDSRTYLVNSSDSGSSQTESPSSKYSGFFSEVSQDHETMAQVLFSRNMRLNVALTFWRKR
SISELVAYLLRIEDLGVVVDCLPVLTNCLQEEKQYISLGCCVDLLPLVKSLLKSKFEEYVIVGLNWLQAVIKRWWSELSSKTEIINDGNIQILKQQLSGLWEQENHLTLVPGYTGNIAKDVDAYLLQLH
0000000000000000000000000000000000000000000000000000000000000000000000000000000000000000000000000000000000000000000000000000000000000000000000000000000000000000000000000
000000000000000000000000000000000000000000000000000000000000000000000000000000000000000000000000000000000000000000000000000000000
0000000000000000000000000000000000000000000000000000000000000000000000000000000000000000000000000000000000000000000000000000000000000000000000000000000000000000000000000
000000000000000000000000000000000000000000000000000000000000000000000000000000000000000000000000000000000000000000000000000000000
0000000000000000000000000000000000000000000000000000000000000000000000000000000000000000000000000000000000000000000000000000000000000000000000000000000000000000000000000
000000000000000000000000000000000000000000000000000000000000000000000000000000000000000000000000000000000000000000000000000000000
0000000000000000000000000000000000000000000000000000000000000000000000000000000000000000000000000000000000000000000000000000000000000000000000000000000000000000000000000
000000000000000000000000000000000000000000000000000000000000000000000000000000000000000000000000000000000000000000000000000000000
0000000000000000000000000000000000000000000000000000000000000000000000000000000000000000000000000000000000000000000000000000000000000000000000000000000000000000000000000
000000000000000000000000000000000000000000000000000000000000000000000000000000000000000000000000000000000000000000000000000000000
0000000000000000000000000000000000000000000000000000000000000000000000000000000000000000000000000000000000000000000000000000000000000000000000000000000000000000000000000
000000000000000000000000000000000000000000000000000000000000000000000000000000000000000000000000000000000000000000000000000000000
0000000000000000000000000000000000000000000000000000000000000000000000000000000000000000000000000000000000000000000000000000000000000000000000000000000000000000000000000
000000000000000000000000000000000000000000000000000000000000000000000000000000000000000000000000000000000000000000000000000000000
>Q9P3B1
MEHDLERLCFIGGYDNDNDKVIVVVTKNLELFKKYDDINLIKEAYNHVHKLIQKDERYTAVFFAHDSTVFSYLGLSLKAYYGMDYYLHKNVKAVYVIHTDWMSKVAIRTLLSIASPKFTRKFRYLNSISDLNKYIPLSHLKLPPIVYEFDRNVEPAIFPSNPSATSNFIFPVQQW
TRPPDALVEGMQVVSKSLQTEGLFRKSCSRKHLDIVIELYDNGCMVDLEAFGPIAACSLIKHLFRSLPSPLFSAEFLNGLTDHMDSGIDYAVSLQKLIDASMDKNSQKLARLIFSLLYQITQHEQENMMNAQNLALCIGPSFSKADNIAELMSMKDKEYNPYCYFLEYAILHWNT
LFANEENWDSYIPQDPTLLLPKTP
0000000000000000000000000000000000000000000000000000000000000000000000000000000000000000000000000000000000000000000000000000000000000000000000000000000000000000000000000
0000000000000000000000000000000000000000000000000000000000000000000000000000000000000000000000000000000000000000000000000000000000000000000000000000000000000000000000000
000000000000000000000000
0000000000000000000000000000000000000000000000000000000000000000000000000000000000000000000000000000000000000000000000000000000000000000000000000000000000000000000000000
0000000000000000000000000000000000000000000000000000000000000000000000000000000000000000000000000000000000000000000000000000000000000000000000000000000000000000000000000
000000000000000000000000
0000000000000000000000000000000000000000000000000000000000000000000000000000000000000000000000000000000000000000000000000000000000000000000000000000000000000000000000000
0000000000000000000000000000000000000000000000000000000000000000000000000000000000000000000000000000000000000000000000000000000000000000000000000000000000000000000000000
000000000000000000000000
0000000000000000000000000000000000000000000000000000000000000000000000000000000000000000000000000000000000000000000000000000000000000000000000000000000000000000000000000
0000000000000000000000000000000000000000000000000000000000000000000000000000000000000000000000000000000000000000000000000000000000000000000000000000000000000000000000000
000000000000000000000000
0000000000000000000000000000000000000000000000000000000000000000000000000000000000000000000000000000000000000000000000000000000000000000000000000000000000000000000000000
0000000000000000000000000000000000000000000000000000000000000000000000000000000000000000000000000000000000000000000000000000000000000000000000000000000000000000000000000
000000000000000000000000
0000000000000000000000000000000000000000000000000000000000000000000000000000000000000000000000000000000000000000000000000000000000000000000000000000000000000000000000000
0000000000000000000000000000000000000000000000000000000000000000000000000000000000000000000000000000000000000000000000000000000000000000000000000000000000000000000000000
000000000000000000000000
0000000000000000000000000000000000000000000000000000000000000000000000000000000000000000000000000000000000000000000000000000000000000000000000000000000000000000000000000
0000000000000000000000000000000000000000000000000000000000000000000000000000000000000000000000000000000000000000000000000000000000000000000000000000000000000000000000000
000000000000000000000000
>Q9Z2Z3
MASSEQAEQPSQPSSSPGSENVVPREPLIATAVKFLQNSRVRQSPLATRRAFLKKKGLTDDEIDLAFQQSGTATEEPPSLGLATPVAPVQTPHLIAQPCSPGSSRWRDYGALAIIMAGIAFGFHQLYKKYLLPLILGGREDRKQLERMASSLSELSGSVAQTVTQVQTTLASVQE
LLRQQQKVQELAHELAAAKATTSTNWILESQNINELKSEINSLKGLLLNRRQFPPSPSAPKIPSWQIPVKSPSPSSPAAVNHHSSSDISPVSNESTSSSPGKESHSPEGSTATYHLLGPQEEGEGVVDVKGQVRMEVQGEEEKREDKEEEEEEEEEEDVSHVDEEDVLGVQREDR
RGGDGQINEQVDKLRRPEGASNESERH
0000000000000000000000000000000000000000000000000000000000000000000000000000000000000000000000000000000000000000000000000000000000000000000000000000000000000000000000000
0000000000000000000000000000000000000000000000000000000000000000000000000000000000000000000000000000000000000000000000000000000000000000000000000000000000000000000000000
000000000000000000000000000
0000000000000000000000000000000000000000000000000000000000000000000000000000000000000000000000000000000000000000000000000000000000000000000000000000000000000000000000000
0000000000000000000000000000000000000000000000000000000000000000000000000000000000000000000000000000000000000000000000000000000000000000000000000000000000000000000000000
000000000000000000000000000

0000000000000000000000000000000000000000000000000000000000000000000000000000000000000000000000000000000000000000000000000000000000000000000000000000000000000000000000000000000
0000000000000000000000000000000000000000000000000000000000000000000000000000000000000000000000000000000000000000000000000000000000000000000000000000000000000000000000000000000
000000000000000000000000000
0000000000000000000000000000000000000000000000000000000000000000000000000000000000000000000000000000000000000000000000000000000000000000000000000000000000000000000000000000000
0000000000000000000000000000000000000000000000000000000000000000000000000000000000000000000000000000000000000000000000000000000000000000000000000000000000000000000000000000000
000000000000000000000000000
0000000000000000000000000000000000000000000000000000000000000000000000000000000000000000000000000000000000000000000000000000000000000000000000000000000000000000000000000000000
0000000000000000000000000000000000000000000000000000000000000000000000000000000000000000000000000000000000000000000000000000000000000000000000000000000000000000000000000000000
000000000000000000000000000
0000000000000000000000000000000000000000000000000000000000000000000000000000000000000000000000000000000000000000000000000000000000000000000000000000000000000000000000000000000
0000000000000000000000000000000000000000000000000000000000000000000000000000000000000000000000000000000000000000000000000000000000000000000000000000000000000000000000000000000
000000000000000000000000000
0000000000000000000000000000000000000000000000000000000000000000000000000000000000000000000000000000000000000000000000000000000000000000000000000000000000000000000000000000000
0000000000000000000000000000000000000000000000000000000000000000000000000000000000000000000000000000000000000000000000000000000000000000000000000000000000000000000000000000000
000000000000000000000000000
>DNA_A0A0H2ZDG9
MDKLTAMATFVKVVDAGSFTRAADALGLPKARVSQRVSDLEKHLGVRLLNRTTRALSLTHDGSAYFDKCQVLLQQIDELEATLRGGTATPIGRLRVDSLITIARWVIAPRLHDFQARYPRIQLRLSSSDRISNLLEDGIDCTIRGGALKDSSMIARHLCDIQMGLYASPEYLASI
GGVDSPNDLSQFRRLSWFSGRERNPFMWELESGPERFVVQSGDGMQFDEPDVAISACMAGSGICPGAPFAVAGFVRAGKLVPVLPQWHFSAAPVHVIYPGSRHLSVRVRCFVNWVMELFAENPEIQLTPIALALESGLAQRT
0000000000000000000000000000000000000000000000000000000000000000000000000000000000000000000000000000000000000000000000000000000000000000000000000000000000000000000000000000000
0000000000000000000000000000000000000000000000000000000000000000000000000000000000000000000000000000000000000000000000000000000000000000000000
0000000000000000000000000000000000000000000000000000000000000000000000000000000000000000000000000000000000000000000000000000000000000000000000000000000000000000000000000000000
0000000000000000000000000000000000000000000000000000000000000000000000000000000000000000000000000000000000000000000000000000000000000000000000
0000000000000000000000000000000000000000000000000000000000000000000000000000000000000000000000000000000000000000000000000000000000000000000000000000000000000000000000000000000
0000000000000000000000000000000000000000000000000000000000000000000000000000000000000000000000000000000000000000000000000000000000000000000000
0000000000000000000000000000000000000000000000000000000000000000000000000000000000000000000000000000000000000000000000000000000000000000000000000000000000000000000000000000000
0000000000000000000000000000000000000000000000000000000000000000000000000000000000000000000000000000000000000000000000000000000000000000000000
0000000000000000000000000000000000000000000000000000000000000000000000000000000000000000000000000000000000000000000000000000000000000000000000000000000000000000000000000000000
0000000000000000000000000000000000000000000000000000000000000000000000000000000000000000000000000000000000000000000000000000000000000000000000
0000000000000000000000000000000000000000000000000000000000000000000000000000000000000000000000000000000000000000000000000000000000000000000000000000000000000000000000000000000
0000000000000000000000000000000000000000000000000000000000000000000000000000000000000000000000000000000000000000000000000000000000000000000000
0000000000000000000000000000000000000000000000000000000000000000000000000000000000000000000000000000000000000000000000000000000000000000000000000000000000000000000000000000000
0000000000000000000000000000000000000000000000000000000000000000000000000000000000000000000000000000000000000000000000000000000000000000000000
>DNA_A2RU54
MGSKEDAGKGCPAAGGVSSFTIQSILGGGPSEAPREPVGWPARKRSLSVSSEEEEPDDGWKAPACFCPDQHGPKEQGPKHHPPIPFPCLGTPKGSGGSGPGGLERTPFLSPSHSDFKEEKERLLPAGSPSPGSERPRDGGAERQAGAAKKKTRTVFSRSQVYQLESTFDMKRYLS
SSERACLASSLQLTETQVKTWFQNRRNKWKRQLSAELEAANMAHASAQTLVSMPLVFRDSSLLRVPVPRSLAFPAPLYYPGSNLSALPLYNLYNKLDY
0000000000000000000000000000000000000000000000000000000000000000000000000000000000000000000000000000000000000000000000000000000000000000000000000000000000000000000000000000000
00000000000000000000000000000000000000000000000000000000000000000000000000000000000000000000000000
0000000000000000000000000000000000000000000000000000000000000000000000000000000000000000000000000000000000000000000000000000000000000000000000000000000000000000000000000000000
00000000000000000000000000000000000000000000000000000000000000000000000000000000000000000000000000
0000000000000000000000000000000000000000000000000000000000000000000000000000000000000000000000000000000000000000000000000000000000000000000000000000000000000000000000000000000
00000000000000000000000000000000000000000000000000000000000000000000000000000000000000000000000000
0000000000000000000000000000000000000000000000000000000000000000000000000000000000000000000000000000000000000000000000000000000000000000000000000000000000000000000000000000000
00000000000000000000000000000000000000000000000000000000000000000000000000000000000000000000000000
0000000000000000000000000000000000000000000000000000000000000000000000000000000000000000000000000000000000000000000000000000000000000000000000000000000000000000000000000000000
00000000000000000000000000000000000000000000000000000000000000000000000000000000000000000000000000
0000000000000000000000000000000000000000000000000000000000000000000000000000000000000000000000000000000000000000000000000000000000000000000000000000000000000000000000000000000
00000000000000000000000000000000000000000000000000000000000000000000000000000000000000000000000000
0000000000000000000000000000000000000000000000000000000000000000000000000000000000000000000000000000000000000000000000000000000000000000000000000000000000000000000000000000000
00000000000000000000000000000000000000000000000000000000000000000000000000000000000000000000000000
>DNA_D2SMN4
MGSPDSSSGSAQKPPRHQHQHQPPPPRRQGSAPELPPGFRFHPTDEELVVHYLKKKAAKAPLPVTIIAEVDLYKFDPWELPEKATFGEHEWYFFSPRDRKYPNGARPNRAATSGYWKATGTDKPILASATGCGREKVGVKKALVFYRGKPPRGLKTNWIMHEYRLTGASAGSTTT
SRPPPVTGGSRAPASLRLDDWVLCRIYKKTSKAAAAVGDEQRSMECEDSVEDAVTAYPPYATAGMAGAGAHGSNYVQLLHHHDSHEDNFQLDGLLTEHDVGLSAGAASLGHLAAAARATKQFLAPSSSTPFNWLEASTGGSILPQARNFPGFNRSRNVGSMSLSSTADDMAGAVD
VSDGGNAVNAMYLPVQDGTYHQHVILGAPLAPEAIAGAATSGFQHHVQISGVNWNP
0000000000000000000000000000000000000000000000000000000000000000000000000000000000000000000000000000000000000000000000000000000000000000000000000000000000000000000000000000000
0000000000000000000000000000000000000000000000000000000000000000000000000000000000000000000000000000000000000000000000000000000000000000000000000000000000000000000000000000000
00000000000000000000000000000000000000000000000000000000
0000000000000000000000000000000000000000000000000000000000000000000000000000000000000000000000000000000000000000000000000000000000000000000000000000000000000000000000000000000
0000000000000000000000000000000000000000000000000000000000000000000000000000000000000000000000000000000000000000000000000000000000000000000000000000000000000000000000000000000
00000000000000000000000000000000000000000000000000000000
0000000000000000000000000000000000000000000000000000000000000000000000000000000000000000000000000000000000000000000000000000000000000000000000000000000000000000000000000000000
0000000000000000000000000000000000000000000000000000000000000000000000000000000000000000000000000000000000000000000000000000000000000000000000000000000000000000000000000000000
00000000000000000000000000000000000000000000000000000000
0000000000000000000000000000000000000000000000000000000000000000000000000000000000000000000000000000000000000000000000000000000000000000000000000000000000000000000000000000000
0000000000000000000000000000000000000000000000000000000000000000000000000000000000000000000000000000000000000000000000000000000000000000000000000000000000000000000000000000000
00000000000000000000000000000000000000000000000000000000
0000000000000000000000000000000000000000000000000000000000000000000000000000000000000000000000000000000000000000000000000000000000000000000000000000000000000000000000000000000
0000000000000000000000000000000000000000000000000000000000000000000000000000000000000000000000000000000000000000000000000000000000000000000000000000000000000000000000000000000
00000000000000000000000000000000000000000000000000000000

0000000000000000000000000000000000000000000000000000000000000000000000000000000000000000000000000000000000000000000000000000000000000000000000000000000000000000000000000000000
0000000000000000000000000000000000000000000000000000000000000000000000000000000000000000000000000000000000000000000000000000000000000000000000000000000000000000000000000000000
00000000000000000000000000000000000000000000000000000000
0000000000000000000000000000000000000000000000000000000000000000000000000000000000000000000000000000000000000000000000000000000000000000000000000000000000000000000000000000000
0000000000000000000000000000000000000000000000000000000000000000000000000000000000000000000000000000000000000000000000000000000000000000000000000000000000000000000000000000000
00000000000000000000000000000000000000000000000000000000
>DNA_F9USS8
MVLTDIEYLLSYLEAHHVPTIKKKRHTYLTYHGLAEHYTYVLKDGIIKNSIILQDGREYNLSYIAKPDVISLLRDEVSRSTDQPFNVRIESEYATFYQVNRVAFWKYVNSTPELQNYVKNYYRKKLSENILRLQRMVMNGKKGAICAFIYSLVDLFGRKVNEGILIDFVVTNDDI
AGFCGISSRSSVNRMLKELRTDGVITVKNHKFIIQDVSYLLDQIAN
0000000000000000000000000000000000000000000000000000000000000000000000000000000000000000000000000000000000000000000000000000000000000000000000000000000000000000000000000000000
0000000000000000000000000000000000000000000000
0000000000000000000000000000000000000000000000000000000000000000000000000000000000000000000000000000000000000000000000000000000000000000000000000000000000000000000000000000000
0000000000000000000000000000000000000000000000
0000000000000000000000000000000000000000000000000000000000000000000000000000000000000000000000000000000000000000000000000000000000000000000000000000000000000000000000000000000
0000000000000000000000000000000000000000000000
0000000000000000000000000000000000000000000000000000000000000000000000000000000000000000000000000000000000000000000000000000000000000000000000000000000000000000000000000000000
0000000000000000000000000000000000000000000000
0000000000000000000000000000000000000000000000000000000000000000000000000000000000000000000000000000000000000000000000000000000000000000000000000000000000000000000000000000000
0000000000000000000000000000000000000000000000
0000000000000000000000000000000000000000000000000000000000000000000000000000000000000000000000000000000000000000000000000000000000000000000000000000000000000000000000000000000
0000000000000000000000000000000000000000000000
0000000000000000000000000000000000000000000000000000000000000000000000000000000000000000000000000000000000000000000000000000000000000000000000000000000000000000000000000000000
0000000000000000000000000000000000000000000000
>DNA_O14270
MPVAEIKNATQQPSSTNRVQAYAKLEFEKFSFFVQTLQVTMGRKASNSSDCDVHLGDTKAISRQHAKIFYSFPNQRFEISVMGKNGAFVDGEFVERGKSVPLRSGTRVQIGQISFSFLLPEGSEEDGHLKETGITPLSLQQGKIAYSDEFGGKPTGSFHTVTSNQEKDLLFSHIK
HESDLPLGLSPADTNISNATSIIEHPDAANAHTLASLNQPPKHLTVSPSSIQRLSPQPYVRPTSDERPIETDSSVSAPKVANHDEELKQGKSTSPSDTVLHPDLNGSPDTGDATQKPNLSYANLIARTLIANPNKKMTLGDICEWIANNWSYYRHQPPAWHNSIRHNLSLNKAFI
RIPRRQNEPGKGSFWMLDPSYIDQFEGNFFRRTKKPTPSATPAAHPDTARENELAAIQTKGISAGKTEQLNPQKETSRSKTHTSRGENVEDRPQSLLQNGIQPIIMRDGKLALNPEFFKNANGEQQAPNEQAVQAISLLQQHINKQLGPAAANNPEQATAIANALAVALAQKLQK
QQTQMQGPQQVQQQAKRRKAYTSQQLNPAPTAMPHPNITSPSPSISVTQRPAVNVGPPPYVRPSAPSKLPDTRQSIGDPLPPGAMANVSAGPSSVRSSSYNSTASESKSEITSHQNLHTIPINKPFTSDRPLYSSPNDTLERVETGNQGQRMNSIGNASSFSKRDIMENENGSFD
TNAKNGNNVDDSSSVRGMNLPSNSSDALRGVKRPLDETSSSYT
0000000000000000000000000000000000000000000000000000000000000000000000000000000000000000000000000000000000000000000000000000000000000000000000000000000000000000000000000000000
0000000000000000000000000000000000000000000000000000000000000000000000000000000000000000000000000000000000000000000000000000000000000000000000000000000000000000000000000000000
0000000000000000000000000000000000000000000000000000000000000000000000000000000000000000000000000000000000000000000000000000000000000000000000000000000000000000000000000000000
0000000000000000000000000000000000000000000000000000000000000000000000000000000000000000000000000000000000000000000000000000000000000000000000000000000000000000000000000000000
0000000000000000000000000000000000000000000
0000000000000000000000000000000000000000000000000000000000000000000000000000000000000000000000000000000000000000000000000000000000000000000000000000000000000000000000000000000
0000000000000000000000000000000000000000000000000000000000000000000000000000000000000000000000000000000000000000000000000000000000000000000000000000000000000000000000000000000
0000000000000000000000000000000000000000000000000000000000000000000000000000000000000000000000000000000000000000000000000000000000000000000000000000000000000000000000000000000
0000000000000000000000000000000000000000000000000000000000000000000000000000000000000000000000000000000000000000000000000000000000000000000000000000000000000000000000000000000
0000000000000000000000000000000000000000000
0000000000000000000000000000000000000000000000000000000000000000000000000000000000000000000000000000000000000000000000000000000000000000000000000000000000000000000000000000000
0000000000000000000000000000000000000000000000000000000000000000000000000000000000000000000000000000000000000000000000000000000000000000000000000000000000000000000000000000000
0000000000000000000000000000000000000000000000000000000000000000000000000000000000000000000000000000000000000000000000000000000000000000000000000000000000000000000000000000000
0000000000000000000000000000000000000000000000000000000000000000000000000000000000000000000000000000000000000000000000000000000000000000000000000000000000000000000000000000000
0000000000000000000000000000000000000000000
0000000000000000000000000000000000000000000000000000000000000000000000000000000000000000000000000000000000000000000000000000000000000000000000000000000000000000000000000000000
0000000000000000000000000000000000000000000000000000000000000000000000000000000000000000000000000000000000000000000000000000000000000000000000000000000000000000000000000000000
0000000000000000000000000000000000000000000000000000000000000000000000000000000000000000000000000000000000000000000000000000000000000000000000000000000000000000000000000000000
0000000000000000000000000000000000000000000000000000000000000000000000000000000000000000000000000000000000000000000000000000000000000000000000000000000000000000000000000000000
0000000000000000000000000000000000000000000
0000000000000000000000000000000000000000000000000000000000000000000000000000000000000000000000000000000000000000000000000000000000000000000000000000000000000000000000000000000
0000000000000000000000000000000000000000000000000000000000000000000000000000000000000000000000000000000000000000000000000000000000000000000000000000000000000000000000000000000
0000000000000000000000000000000000000000000000000000000000000000000000000000000000000000000000000000000000000000000000000000000000000000000000000000000000000000000000000000000
0000000000000000000000000000000000000000000000000000000000000000000000000000000000000000000000000000000000000000000000000000000000000000000000000000000000000000000000000000000
0000000000000000000000000000000000000000000
0000000000000000000000000000000000000000000000000000000000000000000000000000000000000000000000000000000000000000000000000000000000000000000000000000000000000000000000000000000
0000000000000000000000000000000000000000000000000000000000000000000000000000000000000000000000000000000000000000000000000000000000000000000000000000000000000000000000000000000
0000000000000000000000000000000000000000000000000000000000000000000000000000000000000000000000000000000000000000000000000000000000000000000000000000000000000000000000000000000
0000000000000000000000000000000000000000000000000000000000000000000000000000000000000000000000000000000000000000000000000000000000000000000000000000000000000000000000000000000
0000000000000000000000000000000000000000000
0000000000000000000000000000000000000000000000000000000000000000000000000000000000000000000000000000000000000000000000000000000000000000000000000000000000000000000000000000000
0000000000000000000000000000000000000000000000000000000000000000000000000000000000000000000000000000000000000000000000000000000000000000000000000000000000000000000000000000000
0000000000000000000000000000000000000000000000000000000000000000000000000000000000000000000000000000000000000000000000000000000000000000000000000000000000000000000000000000000
0000000000000000000000000000000000000000000000000000000000000000000000000000000000000000000000000000000000000000000000000000000000000000000000000000000000000000000000000000000
0000000000000000000000000000000000000000000
>DNA_O31456
MYYEKAVQKTINWIESHLHEQISNEDIVNVSSFSKFHFHRIFQKEVGMSVASYIRLRRLANAAAALLYTDHRIIDIALYYQFESQEAFTRTFKKMYHMPPGAYRTFMKRFTSKKEESYMEKKMKGWVLSGSHPFQFEMGIDRENVHQGKASGYLKSTMVQDIGEFATMMQQFKAD
RYLGKRLRLSSFIKTKGVQHFASLWMRVDSAADDVLQFDNMSNRPITGTTNWNHYAIVLDVPENSAVISFGVQLSGPGQVWMDHVVFEEVDESVPSTNLEMPGELLDEPVNLSFEEELQK
0000000000000000000000000000000000000000000000000000000000000000000000000000000000000000000000000000000000000000000000000000000000000000000000000000000000000000000000000000000
000000000000000000000000000000000000000000000000000000000000000000000000000000000000000000000000000000000000000000000000

0000000000000000000000000000000000000000000000000000000000000000000000000000000000000000000000000000000000000000000000000000000000000000000000000000000000000000000000000000000
000000000000000000000000000000000000000000000000000000000000000000000000000000000000000000000000000000000000000000000000
0000000000000000000000000000000000000000000000000000000000000000000000000000000000000000000000000000000000000000000000000000000000000000000000000000000000000000000000000000000
000000000000000000000000000000000000000000000000000000000000000000000000000000000000000000000000000000000000000000000000
0000000000000000000000000000000000000000000000000000000000000000000000000000000000000000000000000000000000000000000000000000000000000000000000000000000000000000000000000000000
000000000000000000000000000000000000000000000000000000000000000000000000000000000000000000000000000000000000000000000000
0000000000000000000000000000000000000000000000000000000000000000000000000000000000000000000000000000000000000000000000000000000000000000000000000000000000000000000000000000000
000000000000000000000000000000000000000000000000000000000000000000000000000000000000000000000000000000000000000000000000
0000000000000000000000000000000000000000000000000000000000000000000000000000000000000000000000000000000000000000000000000000000000000000000000000000000000000000000000000000000
000000000000000000000000000000000000000000000000000000000000000000000000000000000000000000000000000000000000000000000000
0000000000000000000000000000000000000000000000000000000000000000000000000000000000000000000000000000000000000000000000000000000000000000000000000000000000000000000000000000000
000000000000000000000000000000000000000000000000000000000000000000000000000000000000000000000000000000000000000000000000
>DNA_O32006
MELKNIVNSYNITNILGYLRRSRQDMEREKRTGEDTLTEQKELMNKILTAIEIPYELKMEIGSGESIDGRPVFKECLKDLEEGKYQAIAVKEITRLSRGSYSDAGQIVNLLQSKRLIIITPYKVYDPRNPVDMRQIRFELFMAREEFEMTRERMTGAKYTYAAQGKWISGLAPYG
YQLNKKTSKLDPVEDEAKVVQLIFNIFLNGLNGKDYSYTAIASHLTNLQIPTPSGKKRWNQYTIKAILQNEVYIGTVKYKVREKTKDGKRTIRPEKEQIVVQDAHAPIIDKEQFQQSQVKIANKVPLLPNKDEFELSELAGVCTCSKCGEPLSKYESKRIRKNKDGTESVYHVKS
LTCKKNKCTYVRYNDVENAILDYLSSLNDLNDSTLTKHINSMLSKYEDDNSNMKTKKQMSEHLSQKEKELKNKENFIFDKYESGIYSDELFLKRKAALDEEFKELQNAKNELNGLQDTQSEIDSNTVRNNINKIIDQYHIESSSEKKNELLRMVLKDVIVNMTQKRKGPIPAQFE
ITPILRFNFIFDLTATNSFH
0000000000000000000000000000000000000000000000000000000000000000000000000000000000000000000000000000000000000000000000000000000000000000000000000000000000000000000000000000000
0000000000000000000000000000000000000000000000000000000000000000000000000000000000000000000000000000000000000000000000000000000000000000000000000000000000000000000000000000000
0000000000000000000000000000000000000000000000000000000000000000000000000000000000000000000000000000000000000000000000000000000000000000000000000000000000000000000000000000000
00000000000000000000
0000000000000000000000000000000000000000000000000000000000000000000000000000000000000000000000000000000000000000000000000000000000000000000000000000000000000000000000000000000
0000000000000000000000000000000000000000000000000000000000000000000000000000000000000000000000000000000000000000000000000000000000000000000000000000000000000000000000000000000
0000000000000000000000000000000000000000000000000000000000000000000000000000000000000000000000000000000000000000000000000000000000000000000000000000000000000000000000000000000
00000000000000000000
0000000000000000000000000000000000000000000000000000000000000000000000000000000000000000000000000000000000000000000000000000000000000000000000000000000000000000000000000000000
0000000000000000000000000000000000000000000000000000000000000000000000000000000000000000000000000000000000000000000000000000000000000000000000000000000000000000000000000000000
0000000000000000000000000000000000000000000000000000000000000000000000000000000000000000000000000000000000000000000000000000000000000000000000000000000000000000000000000000000
00000000000000000000
0000000000000000000000000000000000000000000000000000000000000000000000000000000000000000000000000000000000000000000000000000000000000000000000000000000000000000000000000000000
0000000000000000000000000000000000000000000000000000000000000000000000000000000000000000000000000000000000000000000000000000000000000000000000000000000000000000000000000000000
0000000000000000000000000000000000000000000000000000000000000000000000000000000000000000000000000000000000000000000000000000000000000000000000000000000000000000000000000000000
00000000000000000000
0000000000000000000000000000000000000000000000000000000000000000000000000000000000000000000000000000000000000000000000000000000000000000000000000000000000000000000000000000000
0000000000000000000000000000000000000000000000000000000000000000000000000000000000000000000000000000000000000000000000000000000000000000000000000000000000000000000000000000000
0000000000000000000000000000000000000000000000000000000000000000000000000000000000000000000000000000000000000000000000000000000000000000000000000000000000000000000000000000000
00000000000000000000
0000000000000000000000000000000000000000000000000000000000000000000000000000000000000000000000000000000000000000000000000000000000000000000000000000000000000000000000000000000
0000000000000000000000000000000000000000000000000000000000000000000000000000000000000000000000000000000000000000000000000000000000000000000000000000000000000000000000000000000
0000000000000000000000000000000000000000000000000000000000000000000000000000000000000000000000000000000000000000000000000000000000000000000000000000000000000000000000000000000
00000000000000000000
0000000000000000000000000000000000000000000000000000000000000000000000000000000000000000000000000000000000000000000000000000000000000000000000000000000000000000000000000000000
0000000000000000000000000000000000000000000000000000000000000000000000000000000000000000000000000000000000000000000000000000000000000000000000000000000000000000000000000000000
0000000000000000000000000000000000000000000000000000000000000000000000000000000000000000000000000000000000000000000000000000000000000000000000000000000000000000000000000000000
00000000000000000000
>DNA_O32228
MDKRILAETFRLIKQKGFSFTMNDLAAALGTSKRTLYAYYSSKDQLVEAVVEQFIAEMKQIERDIYENESLNVLEKVKQMLISLPQGMELLNMGLLNELKKYHYDAWLTLDAFIKEEWAIVLALLNECKQQKRIRNINPDLFVHMYIGSINQVYDPEYPLKHQFTTGEVLESIVD
VLFNGITADEEADAT
0000000000000000000000000000000000000000000000000000000000000000000000000000000000000000000000000000000000000000000000000000000000000000000000000000000000000000000000000000000
000000000000000
0000000000000000000000000000000000000000000000000000000000000000000000000000000000000000000000000000000000000000000000000000000000000000000000000000000000000000000000000000000
000000000000000
0000000000000000000000000000000000000000000000000000000000000000000000000000000000000000000000000000000000000000000000000000000000000000000000000000000000000000000000000000000
000000000000000
0000000000000000000000000000000000000000000000000000000000000000000000000000000000000000000000000000000000000000000000000000000000000000000000000000000000000000000000000000000
000000000000000
0000000000000000000000000000000000000000000000000000000000000000000000000000000000000000000000000000000000000000000000000000000000000000000000000000000000000000000000000000000
000000000000000
0000000000000000000000000000000000000000000000000000000000000000000000000000000000000000000000000000000000000000000000000000000000000000000000000000000000000000000000000000000
000000000000000
0000000000000000000000000000000000000000000000000000000000000000000000000000000000000000000000000000000000000000000000000000000000000000000000000000000000000000000000000000000
000000000000000
>DNA_O60158
MTENEKSRSLPAERNPLYKDDTLDHTPLIPKCRAQVIEFPDGPATFVRLKCTNPESKVPHFLMRMAKDSSISATSMFRSAFPKATQEEEDLEMRWIRDNLNPIEDKRVAGLWVPPADALALAKDYSMTPFINALLEASSTPSTYATPSRPTAQKSETSEGEPESSTSATTTSVAR
RTRQRLAEHLENSKKTILQHDNKEEDKEIHSEENETKDEIKSEKKEPEIKKQEGGSSTEKVGQPSSSDDKAKGSTSKDQPSEEEEKTSDIQDRKIKTPIKPSLLGKIRSSVNKGMTDVASQVNRGMTDVASQVNKGVNGVASQVNKGMNGVANQVNKGVTGVASQVRKPVGKLEK
KFENLEKSIGDTLKSSIRSSPKSKKRSREDFEENEDYNAMVPVKRSRITKLESEVYYEKRKVRALGGIAIGLGVGAILPFLF
0000000000000000000000000000000000000000000000000000000000000000000000000000000000000000000000000000000000000000000000000000000000000000000000000000000000000000000000000000000
0000000000000000000000000000000000000000000000000000000000000000000000000000000000000000000000000000000000000000000000000000000000000000000000000000000000000000000000000000000
0000000000000000000000000000000000000000000000000000000000000000000000000000000000

000000000000000000000000000000000000000000000000000000000000000000000000000000000000000000000000000000000000000000000000000000000000000000000000000000000000000000000000000
000000000000000000000000000000000000000000000000000000000000000000000000000000000000000000000000000000000000000000000000000000000000000000000000000000000000000000000000000
0000000000000000000000000000000000000000000000000000000000000000000000000000000000
000000000000000000000000000000000000000000000000000000000000000000000000000000000000000000000000000000000000000000000000000000000000000000000000000000000000000000000000000
000000000000000000000000000000000000000000000000000000000000000000000000000000000000000000000000000000000000000000000000000000000000000000000000000000000000000000000000000
0000000000000000000000000000000000000000000000000000000000000000000000000000000000
000000000000000000000000000000000000000000000000000000000000000000000000000000000000000000000000000000000000000000000000000000000000000000000000000000000000000000000000000
000000000000000000000000000000000000000000000000000000000000000000000000000000000000000000000000000000000000000000000000000000000000000000000000000000000000000000000000000
0000000000000000000000000000000000000000000000000000000000000000000000000000000000
000000000000000000000000000000000000000000000000000000000000000000000000000000000000000000000000000000000000000000000000000000000000000000000000000000000000000000000000000
000000000000000000000000000000000000000000000000000000000000000000000000000000000000000000000000000000000000000000000000000000000000000000000000000000000000000000000000000
0000000000000000000000000000000000000000000000000000000000000000000000000000000000
000000000000000000000000000000000000000000000000000000000000000000000000000000000000000000000000000000000000000000000000000000000000000000000000000000000000000000000000000
000000000000000000000000000000000000000000000000000000000000000000000000000000000000000000000000000000000000000000000000000000000000000000000000000000000000000000000000000
0000000000000000000000000000000000000000000000000000000000000000000000000000000000
000000000000000000000000000000000000000000000000000000000000000000000000000000000000000000000000000000000000000000000000000000000000000000000000000000000000000000000000000
000000000000000000000000000000000000000000000000000000000000000000000000000000000000000000000000000000000000000000000000000000000000000000000000000000000000000000000000000
0000000000000000000000000000000000000000000000000000000000000000000000000000000000
>DNA_P03690
MIITTEKETILGNGSKSKAFSITASPKVFKILSSDLYTNKIRAVVRELITNMIDAHALNGNPEKFIIQVPGRLDPRFVCRDFGPGMSDFDIQGDDNSPGLYNSYFSSSKAESNDFIGGFGLGSKSPFSYTDTFSITSYHKGEIRGYVAYMDGDGPQIKPTFVKEMGPDDKTGIEI
VVPVEEKDFRNFAYEVSYIMRPFKDLAIINGLDREIDYFPDFDDYYGVNPERYWPDRGGLYAIYGGIVYPIDGVIRDRNWLSIRNEVNYIKFPMGSLDIAPSREALSLDDRTRKNIIERVKELSEKAFNEDVKRFKESTSPRHTYRELMKMGYSARDYMISNSVKFTTKNLSYKK
MQSMFEPDSKLCNAGVVYEVNLDPRLKRIKQSHETSAVASSYRLFGINTTKINIVIDNIKNRVNIVRGLARALDDSEFNNTLNIHHNERLLFINPEVESQIDLLPDIMAMFESDEVNIHYLSEIEALVKSYIPKVVKSKAPRPKAATAFKFEIKDGRWEKRNYLRLTSEADEITG
YVAYMHRSDIFSMDGTTSLCHPSMNILIRMANLIGINEFYVIRPLLQKKVKELGQCQCIFEALRDLYVDAFDDVDYDKYVGYSSSAKRYIDKIIKYPELDFMMKYFSIDEVSEEYTRLANMVSSLQGVYFNGGKDTIGHDIWTVTNLFDVLSNNASKNSDKMVAEFTKKFRIVSD
FIGYRNSLSDDEVSQIAKTMKALAA
000000000000000000000000000000000000000000000000000000000000000000000000000000000000000000000000000000000000000000000000000000000000000000000000000000000000000000000000000
000000000000000000000000000000000000000000000000000000000000000000000000000000000000000000000000000000000000000000000000000000000000000000000000000000000000000000000000000
000000000000000000000000000000000000000000000000000000000000000000000000000000000000000000000000000000000000000000000000000000000000000000000000000000000000000000000000000
000000000000000000000000000000000000000000000000000000000000000000000000000000000000000000000000000000000000000000000000000000000000000000000000000000000000000000000000000
0000000000000000000000000
000000000000000000000000000000000000000000000000000000000000000000000000000000000000000000000000000000000000000000000000000000000000000000000000000000000000000000000000000
000000000000000000000000000000000000000000000000000000000000000000000000000000000000000000000000000000000000000000000000000000000000000000000000000000000000000000000000000
000000000000000000000000000000000000000000000000000000000000000000000000000000000000000000000000000000000000000000000000000000000000000000000000000000000000000000000000000
000000000000000000000000000000000000000000000000000000000000000000000000000000000000000000000000000000000000000000000000000000000000000000000000000000000000000000000000000
0000000000000000000000000
000000000000000000000000000000000000000000000000000000000000000000000000000000000000000000000000000000000000000000000000000000000000000000000000000000000000000000000000000
000000000000000000000000000000000000000000000000000000000000000000000000000000000000000000000000000000000000000000000000000000000000000000000000000000000000000000000000000
000000000000000000000000000000000000000000000000000000000000000000000000000000000000000000000000000000000000000000000000000000000000000000000000000000000000000000000000000
000000000000000000000000000000000000000000000000000000000000000000000000000000000000000000000000000000000000000000000000000000000000000000000000000000000000000000000000000
0000000000000000000000000
000000000000000000000000000000000000000000000000000000000000000000000000000000000000000000000000000000000000000000000000000000000000000000000000000000000000000000000000000
000000000000000000000000000000000000000000000000000000000000000000000000000000000000000000000000000000000000000000000000000000000000000000000000000000000000000000000000000
000000000000000000000000000000000000000000000000000000000000000000000000000000000000000000000000000000000000000000000000000000000000000000000000000000000000000000000000000
000000000000000000000000000000000000000000000000000000000000000000000000000000000000000000000000000000000000000000000000000000000000000000000000000000000000000000000000000
0000000000000000000000000
000000000000000000000000000000000000000000000000000000000000000000000000000000000000000000000000000000000000000000000000000000000000000000000000000000000000000000000000000
000000000000000000000000000000000000000000000000000000000000000000000000000000000000000000000000000000000000000000000000000000000000000000000000000000000000000000000000000
000000000000000000000000000000000000000000000000000000000000000000000000000000000000000000000000000000000000000000000000000000000000000000000000000000000000000000000000000
000000000000000000000000000000000000000000000000000000000000000000000000000000000000000000000000000000000000000000000000000000000000000000000000000000000000000000000000000
0000000000000000000000000
000000000000000000000000000000000000000000000000000000000000000000000000000000000000000000000000000000000000000000000000000000000000000000000000000000000000000000000000000
000000000000000000000000000000000000000000000000000000000000000000000000000000000000000000000000000000000000000000000000000000000000000000000000000000000000000000000000000
000000000000000000000000000000000000000000000000000000000000000000000000000000000000000000000000000000000000000000000000000000000000000000000000000000000000000000000000000
000000000000000000000000000000000000000000000000000000000000000000000000000000000000000000000000000000000000000000000000000000000000000000000000000000000000000000000000000
0000000000000000000000000
000000000000000000000000000000000000000000000000000000000000000000000000000000000000000000000000000000000000000000000000000000000000000000000000000000000000000000000000000
000000000000000000000000000000000000000000000000000000000000000000000000000000000000000000000000000000000000000000000000000000000000000000000000000000000000000000000000000
000000000000000000000000000000000000000000000000000000000000000000000000000000000000000000000000000000000000000000000000000000000000000000000000000000000000000000000000000
000000000000000000000000000000000000000000000000000000000000000000000000000000000000000000000000000000000000000000000000000000000000000000000000000000000000000000000000000
0000000000000000000000000
>DNA_P0A9F0
MSESAFKDCFLTDVSADTRLFHFLARDYIVQEGQQPSWLFYLTRGRARLYATLANGRVSLIDFFAAPCFIGEIELIDKDHEPRAVQAIEECWCLALPMKHYRPLLLNDTLFLRKLCVTLSHKNYRNIVSLTQNQSFPLVNRLAAFILLSQEGDLYHEKHTQAAEYLGVSYRHLLY
VLAQFIHDGLLIKSKKGYLIKNRKQLSGLALEMDPENKFSGMMQ
000000000000000000000000000000000000000000000000000000000000000000000000000000000000000000000000000000000000000000000000000000000000000000000000000000000000000000000000000
00000000000000000000000000000000000000000000
000000000000000000000000000000000000000000000000000000000000000000000000000000000000000000000000000000000000000000000000000000000000000000000000000000000000000000000000000
00000000000000000000000000000000000000000000
000000000000000000000000000000000000000000000000000000000000000000000000000000000000000000000000000000000000000000000000000000000000000000000000000000000000000000000000000
00000000000000000000000000000000000000000000

0000000000000000000000000000000000000000000000000000000000000000000000000000000000000000000000000000000000000000000000000000000000000000000000000000000000000000000000000000000
00000000000000000000000000000000000000000000
0000000000000000000000000000000000000000000000000000000000000000000000000000000000000000000000000000000000000000000000000000000000000000000000000000000000000000000000000000000
00000000000000000000000000000000000000000000
0000000000000000000000000000000000000000000000000000000000000000000000000000000000000000000000000000000000000000000000000000000000000000000000000000000000000000000000000000000
00000000000000000000000000000000000000000000
0000000000000000000000000000000000000000000000000000000000000000000000000000000000000000000000000000000000000000000000000000000000000000000000000000000000000000000000000000000
00000000000000000000000000000000000000000000
>DNA_P10105
MMDVSSMYGNHPHHHHPHANAYDGYSTTTASAANASSYFAPQQHQPHLQLQQQQQHQHLQQPQQHLTYNGYESSSPGNYYPQQQAQLTPPPTSSHQVVQQHQQQQQAQQQQLYPHSHLFSPSAAEYGITTSTTTGNPGTPLHPSSHSPADSYYESDSVHSYYATAAVATVAPPSN
SSPITAANASATSNTQQQQQQAAIISSENGMMYTNLDCMYPTAQAQAPVHGYAGQIEEKYAAVLHASYAPGMVLEDQDPMMQQATQSQMWHHQQHLAGSYALDAMDSLGMHAHMHHGLPHGHLGNLANNPHQQQPQVQQQQQQPHQQPQHPQNQSPAAHQQHHQNSVSPNGGMNR
QQRGGVISPGSSTSSSTSASNGAHPASTQSKSPNHSSSIPTYKWMQLKRNVPKPQAPSYLPAPKLPASGIASMHDYQMNGQLDMCRGGGGGGGSGVGNGPVGVGGNGSPGIGGVLSVQNSLIMANSAAAAGSAHPNGMGVGLGSGSGLSSCSLSSNTNNSGRTNFTNKQLTELEKE
FHFNRYLTRARRIEIANTLQLNETQVKIWFQNRRMKQKKRVKEGLIPADILTQHSTSVISEKPPQQQQPQPPELQLKSQGSDLGGNELATGAPSTPTTAMTLTAPTSKQS
0000000000000000000000000000000000000000000000000000000000000000000000000000000000000000000000000000000000000000000000000000000000000000000000000000000000000000000000000000000
0000000000000000000000000000000000000000000000000000000000000000000000000000000000000000000000000000000000000000000000000000000000000000000000000000000000000000000000000000000
0000000000000000000000000000000000000000000000000000000000000000000000000000000000000000000000000000000000000000000000000000000000000000000000000000000000000000000000000000000
00000000000000000000000000000000000000000000000000000000000000000000000000000000000000000000000000000000000000
0000000000000000000000000000000000000000000000000000000000000000000000000000000000000000000000000000000000000000000000000000000000000000000000000000000000000000000000000000000
0000000000000000000000000000000000000000000000000000000000000000000000000000000000000000000000000000000000000000000000000000000000000000000000000000000000000000000000000000000
0000000000000000000000000000000000000000000000000000000000000000000000000000000000000000000000000000000000000000000000000000000000000000000000000000000000000000000000000000000
00000000000000000000000000000000000000000000000000000000000000000000000000000000000000000000000000000000000000
0000000000000000000000000000000000000000000000000000000000000000000000000000000000000000000000000000000000000000000000000000000000000000000000000000000000000000000000000000000
0000000000000000000000000000000000000000000000000000000000000000000000000000000000000000000000000000000000000000000000000000000000000000000000000000000000000000000000000000000
0000000000000000000000000000000000000000000000000000000000000000000000000000000000000000000000000000000000000000000000000000000000000000000000000000000000000000000000000000000
00000000000000000000000000000000000000000000000000000000000000000000000000000000000000000000000000000000000000
0000000000000000000000000000000000000000000000000000000000000000000000000000000000000000000000000000000000000000000000000000000000000000000000000000000000000000000000000000000
0000000000000000000000000000000000000000000000000000000000000000000000000000000000000000000000000000000000000000000000000000000000000000000000000000000000000000000000000000000
0000000000000000000000000000000000000000000000000000000000000000000000000000000000000000000000000000000000000000000000000000000000000000000000000000000000000000000000000000000
00000000000000000000000000000000000000000000000000000000000000000000000000000000000000000000000000000000000000
0000000000000000000000000000000000000000000000000000000000000000000000000000000000000000000000000000000000000000000000000000000000000000000000000000000000000000000000000000000
0000000000000000000000000000000000000000000000000000000000000000000000000000000000000000000000000000000000000000000000000000000000000000000000000000000000000000000000000000000
0000000000000000000000000000000000000000000000000000000000000000000000000000000000000000000000000000000000000000000000000000000000000000000000000000000000000000000000000000000
00000000000000000000000000000000000000000000000000000000000000000000000000000000000000000000000000000000000000
0000000000000000000000000000000000000000000000000000000000000000000000000000000000000000000000000000000000000000000000000000000000000000000000000000000000000000000000000000000
0000000000000000000000000000000000000000000000000000000000000000000000000000000000000000000000000000000000000000000000000000000000000000000000000000000000000000000000000000000
0000000000000000000000000000000000000000000000000000000000000000000000000000000000000000000000000000000000000000000000000000000000000000000000000000000000000000000000000000000
00000000000000000000000000000000000000000000000000000000000000000000000000000000000000000000000000000000000000
0000000000000000000000000000000000000000000000000000000000000000000000000000000000000000000000000000000000000000000000000000000000000000000000000000000000000000000000000000000
0000000000000000000000000000000000000000000000000000000000000000000000000000000000000000000000000000000000000000000000000000000000000000000000000000000000000000000000000000000
0000000000000000000000000000000000000000000000000000000000000000000000000000000000000000000000000000000000000000000000000000000000000000000000000000000000000000000000000000000
00000000000000000000000000000000000000000000000000000000000000000000000000000000000000000000000000000000000000
>DNA_P10180
MQPTLPQAAGTADMDLTAVQSINDWFFKKEQIYLLAQFWQQRATLAEKEVNTLKEQLSTGNPDSNLNSENSDTAAAAATAAAVAAVVAGATATNDIEDEQQQQLQQTASGGILESDSDKLLNSSIVAAAITLQQQNGSNLLANTNTPSPSPPLLSAEQQQQLQSSLQQSGGVGGA
CLNPKLFFNHAQQMMMMEAAAAAAAAAALQQQQQQQSPLHSPANEVAIPTEQPAATVATGAAAAAAAAAATPIATGNVKSGSTTSNANHTNSNNSHQDEEELDDEEEDEEEDEDEDDEEENASMQSNADDMELDAQQETRTEPSATTQQQHQQQDTEDLEENKDAGEASLNVSNNHN
TTDSNNSCSRKNNNGGNESEQHVASSAEDDDCANNNTNTSNNNNTSNTATSNTNNNNNNNNSSSGNSEKRKKKNNNNNNGQPAVLLAAKDKEIKALLDELQRLRAQEQTHLVQIQRLEEHLEVKRQHIIRLEARLDKQQINEALAEATALSAAASTNNNNNSQSSDNNKKLNTAAE
RPMDASSNADLPESTKAPVPAEDDEEDEDQAMLVDSEEAEDKPEDSHHDDDEDEDEDREAVNATTTDSNELKIKKEQHSPLDLNVLSPNSAIAAAAAAAAAAACANDPNKFQALLIERTKALAAEALKNGASDALSEDAHHQQQQHHQQQHQHQQQHHQQQHLHQQHHHHLQQQP
NSGSNSNPASNDHHHGHHLHGHGLLHPSSAHHLHHQTTESNSNSSTPTAAGNNNGSNNSSSNTNANSTAQLAASLASTLNGTKSLMQEDSNGLAAVAMAAHAQHAAALGPGFLPGLPAFQFAAAQVAAGGDGRGHYRFADSELQLPPGASMAGRLGESLIPKGDPMEAKLQEMLR
YNMDKYANQALDTLHISRRVRELLSVHNIGQRLFAKYILGLSQGTVSELLSKPKPWDKLTEKGRDSYRKMHAWACDDNAVMLLKSLIPKKDSGLPQYAGRGAGGAGGDDSMSEDRIAHILSEASSLMKQSSVAQHREQERRSHGGEDSHSNEDSKSPPQSCTSPFFKVENQLKQH
QHLNPEQAAAQQREREREQREREQQQRLRHDDQDKMARLYQELIARTPRETAFPSFLFSPSLFGGAAGMPGAASNAFPAMADENMRHVFEREIAKLQQHQQQQQAAQAQAQFPNFSSLMALQQQVLNGAQDLSLAAAAAKDIKLNGQRSSLEHSAGSSSCSKDGERDDAYPSSLH
GRKSEGGGTPAPPAPPSGPGTGAGAPPTAAPPTGGASSNSAAPSPLSNSILPPALSSQGEEFAATASPLQRMASITNSLITQPPVTPHHSTPQRPTKAVLPPITQQQFDMFNNLNTEDIVRRVKEALSQYSISQRLFGESVLGLSQGSVSDLLARPKPWHMLTQKGREPFIRMKM
FLEDENAVHKLVASQYKIAPEKLMRTGSYSGSPQMPQGLASKMQAASLPMQKMMSELKLQEPAQAQHLMQQMQAAAMSAAMQQQQVAQAQQQAQQAQQAQQHLQQQAQQHLQQQQHLAQQQHPHQQHHQAAAAAAALHHQSMLLTSPGLPPQHAISLPPSAGGAQPGGPGGNQGS
SNPSNSEKKPMLMPVHGTNAMRSLHQHMSPTVYEMAALTQDLDTHDITTKIKEALLANNIGQKIFGEAVLGLSQGSVSELLSKPKPWHMLSIKGREPFIRMQLWLSDANNVERLQLLKNERREASKRRRSTGPNQQDNSSDTSSNDTNDFYTSSPGPGSVGSGVGGAPPSKKQRV
LFSEEQKEALRLAFALDPYPNVGTIEFLANELGLATRTITNWFHNHRMRLKQQVPHGPAGQDNPIPSRESTSATPFDPVQFRILLQQRLLELHKERMGMSGAPIPYPPYFAAAAILGRSLAGIPGAAAAAGAAAAAAAVGASGGDELQALNQAFKEQMSGLDLSMPTLKRERSDD
YQDDLELEGGGHNLSDNESLEGQEPEDKTTDYEKVLHKSALAAAAAYMSNAVRSSRRKPAAPQWVNPAGAVTNPSAVVAAVAAAAAAAAADNERIINGVCVMQASEYGRDDTDSNKPTDGGNDSDHEHAQLEIDQRFMEPEVHIKQEEDDDEEQSGSVNLDNEDNATSEQKLKVIN
EEKLRMVRVRRLSSTGGGSSEEMPAPLAPPPPPPAASSSIVSGESTTSSSSSSNTSSSTPAVTTAAATAAAGWNY
0000000000000000000000000000000000000000000000000000000000000000000000000000000000000000000000000000000000000000000000000000000000000000000000000000000000000000000000000000000
0000000000000000000000000000000000000000000000000000000000000000000000000000000000000000000000000000000000000000000000000000000000000000000000000000000000000000000000000000000
0000000000000000000000000000000000000000000000000000000000000000000000000000000000000000000000000000000000000000000000000000000000000000000000000000000000000000000000000000000
0000000000000000000000000000000000000000000000000000000000000000000000000000000000000000000000000000000000000000000000000000000000000000000000000000000000000000000000000000000
0000000000000000000000000000000000000000000000000000000000000000000000000000000000000000000000000000000000000000000000000000000000000000000000000000000000000000000000000000000
0000000000000000000000000000000000000000000000000000000000000000000000000000000000000000000000000000000000000000000000000000000000000000000000000000000000000000000000000000000
0000000000000000000000000000000000000000000000000000000000000000000000000000000000000000000000000000000000000000000000000000000000000000000000000000000000000000000000000000000
0000000000000000000000000000000000000000000000000000000000000000000000000000000000000000000000000000000000000000000000000000000000000000000000000000000000000000000000000000000
0000000000000000000000000000000000000000000000000000000000000000000000000000000000000000000000000000000000000000000000000000000000000000000000000000000000000000000000000000000
0000000000000000000000000000000000000000000000000000000000000000000000000000000000000000000000000000000000000000000000000000000000000000000000000000000000000000000000000000000
0000000000000000000000000000000000000000000000000000000000000000000000000000000000000000000000000000000000000000000000000000000000000000000000000000000000000000000000000000000
0000000000000000000000000000000000000000000000000000000000000000000000000000000000000000000000000000000000000000000000000000000000000000000000000000000000000000000000000000000
000000000000000000000000000000000000000000000000000000000000000000000000000

000000000000000000000000000000000000000000000000000000000000000000000000000000000000000000000000000000000000000000000000000000000000000000000000000000000000000000000000000
000000000000000000000000000000000000000000000000000000000000000000000000000000000000000000000000000000000000000000000000000000000000000000000000000000000000000000000000000
000000000000000000000000000000000000000000000000000000000000000000000000000000000000000000000000000000000000000000000000000000000000000000000000000000000000000000000000000
000000000000000000000000000000000000000000000000000000000000000000000000000000000000000000000000000000000000000000000000000000000000000000000000000000000000000000000000000
000000000000000000000000000000000000000000000000000000000000000000000000000000000000000000000000000000000000000000000000000000000000000000000000000000000000000000000000000
000000000000000000000000000000000000000000000000000000000000000000000000000000000000000000000000000000000000000000000000000000000000000000000000000000000000000000000000000
000000000000000000000000000000000000000000000000000000000000000000000000000000000000000000000000000000000000000000000000000000000000000000000000000000000000000000000000000
000000000000000000000000000000000000000000000000000000000000000000000000000000000000000000000000000000000000000000000000000000000000000000000000000000000000000000000000000
000000000000000000000000000000000000000000000000000000000000000000000000000000000000000000000000000000000000000000000000000000000000000000000000000000000000000000000000000
000000000000000000000000000000000000000000000000000000000000000000000000000000000000000000000000000000000000000000000000000000000000000000000000000000000000000000000000000
000000000000000000000000000000000000000000000000000000000000000000000000000000000000000000000000000000000000000000000000000000000000000000000000000000000000000000000000000
000000000000000000000000000000000000000000000000000000000000000000000000000000000000000000000000000000000000000000000000000000000000000000000000000000000000000000000000000
000000000000000000000000000000000000000000000000000000000000000000000000000
000000000000000000000000000000000000000000000000000000000000000000000000000000000000000000000000000000000000000000000000000000000000000000000000000000000000000000000000000
000000000000000000000000000000000000000000000000000000000000000000000000000000000000000000000000000000000000000000000000000000000000000000000000000000000000000000000000000
000000000000000000000000000000000000000000000000000000000000000000000000000000000000000000000000000000000000000000000000000000000000000000000000000000000000000000000000000
000000000000000000000000000000000000000000000000000000000000000000000000000000000000000000000000000000000000000000000000000000000000000000000000000000000000000000000000000
000000000000000000000000000000000000000000000000000000000000000000000000000000000000000000000000000000000000000000000000000000000000000000000000000000000000000000000000000
000000000000000000000000000000000000000000000000000000000000000000000000000000000000000000000000000000000000000000000000000000000000000000000000000000000000000000000000000
000000000000000000000000000000000000000000000000000000000000000000000000000000000000000000000000000000000000000000000000000000000000000000000000000000000000000000000000000
000000000000000000000000000000000000000000000000000000000000000000000000000000000000000000000000000000000000000000000000000000000000000000000000000000000000000000000000000
000000000000000000000000000000000000000000000000000000000000000000000000000000000000000000000000000000000000000000000000000000000000000000000000000000000000000000000000000
000000000000000000000000000000000000000000000000000000000000000000000000000000000000000000000000000000000000000000000000000000000000000000000000000000000000000000000000000
000000000000000000000000000000000000000000000000000000000000000000000000000000000000000000000000000000000000000000000000000000000000000000000000000000000000000000000000000
000000000000000000000000000000000000000000000000000000000000000000000000000000000000000000000000000000000000000000000000000000000000000000000000000000000000000000000000000
000000000000000000000000000000000000000000000000000000000000000000000000000
000000000000000000000000000000000000000000000000000000000000000000000000000000000000000000000000000000000000000000000000000000000000000000000000000000000000000000000000000
000000000000000000000000000000000000000000000000000000000000000000000000000000000000000000000000000000000000000000000000000000000000000000000000000000000000000000000000000
000000000000000000000000000000000000000000000000000000000000000000000000000000000000000000000000000000000000000000000000000000000000000000000000000000000000000000000000000
000000000000000000000000000000000000000000000000000000000000000000000000000000000000000000000000000000000000000000000000000000000000000000000000000000000000000000000000000
000000000000000000000000000000000000000000000000000000000000000000000000000000000000000000000000000000000000000000000000000000000000000000000000000000000000000000000000000
000000000000000000000000000000000000000000000000000000000000000000000000000000000000000000000000000000000000000000000000000000000000000000000000000000000000000000000000000
000000000000000000000000000000000000000000000000000000000000000000000000000000000000000000000000000000000000000000000000000000000000000000000000000000000000000000000000000
000000000000000000000000000000000000000000000000000000000000000000000000000000000000000000000000000000000000000000000000000000000000000000000000000000000000000000000000000
000000000000000000000000000000000000000000000000000000000000000000000000000000000000000000000000000000000000000000000000000000000000000000000000000000000000000000000000000
000000000000000000000000000000000000000000000000000000000000000000000000000000000000000000000000000000000000000000000000000000000000000000000000000000000000000000000000000
000000000000000000000000000000000000000000000000000000000000000000000000000000000000000000000000000000000000000000000000000000000000000000000000000000000000000000000000000
000000000000000000000000000000000000000000000000000000000000000000000000000000000000000000000000000000000000000000000000000000000000000000000000000000000000000000000000000
000000000000000000000000000000000000000000000000000000000000000000000000000
000000000000000000000000000000000000000000000000000000000000000000000000000000000000000000000000000000000000000000000000000000000000000000000000000000000000000000000000000
000000000000000000000000000000000000000000000000000000000000000000000000000000000000000000000000000000000000000000000000000000000000000000000000000000000000000000000000000
000000000000000000000000000000000000000000000000000000000000000000000000000000000000000000000000000000000000000000000000000000000000000000000000000000000000000000000000000
000000000000000000000000000000000000000000000000000000000000000000000000000000000000000000000000000000000000000000000000000000000000000000000000000000000000000000000000000
000000000000000000000000000000000000000000000000000000000000000000000000000000000000000000000000000000000000000000000000000000000000000000000000000000000000000000000000000
000000000000000000000000000000000000000000000000000000000000000000000000000000000000000000000000000000000000000000000000000000000000000000000000000000000000000000000000000
000000000000000000000000000000000000000000000000000000000000000000000000000000000000000000000000000000000000000000000000000000000000000000000000000000000000000000000000000
000000000000000000000000000000000000000000000000000000000000000000000000000000000000000000000000000000000000000000000000000000000000000000000000000000000000000000000000000
000000000000000000000000000000000000000000000000000000000000000000000000000000000000000000000000000000000000000000000000000000000000000000000000000000000000000000000000000
000000000000000000000000000000000000000000000000000000000000000000000000000000000000000000000000000000000000000000000000000000000000000000000000000000000000000000000000000
000000000000000000000000000000000000000000000000000000000000000000000000000000000000000000000000000000000000000000000000000000000000000000000000000000000000000000000000000
000000000000000000000000000000000000000000000000000000000000000000000000000000000000000000000000000000000000000000000000000000000000000000000000000000000000000000000000000
000000000000000000000000000000000000000000000000000000000000000000000000000
000000000000000000000000000000000000000000000000000000000000000000000000000000000000000000000000000000000000000000000000000000000000000000000000000000000000000000000000000
000000000000000000000000000000000000000000000000000000000000000000000000000000000000000000000000000000000000000000000000000000000000000000000000000000000000000000000000000
000000000000000000000000000000000000000000000000000000000000000000000000000000000000000000000000000000000000000000000000000000000000000000000000000000000000000000000000000
000000000000000000000000000000000000000000000000000000000000000000000000000000000000000000000000000000000000000000000000000000000000000000000000000000000000000000000000000
000000000000000000000000000000000000000000000000000000000000000000000000000000000000000000000000000000000000000000000000000000000000000000000000000000000000000000000000000
000000000000000000000000000000000000000000000000000000000000000000000000000000000000000000000000000000000000000000000000000000000000000000000000000000000000000000000000000
000000000000000000000000000000000000000000000000000000000000000000000000000000000000000000000000000000000000000000000000000000000000000000000000000000000000000000000000000
000000000000000000000000000000000000000000000000000000000000000000000000000000000000000000000000000000000000000000000000000000000000000000000000000000000000000000000000000
000000000000000000000000000000000000000000000000000000000000000000000000000000000000000000000000000000000000000000000000000000000000000000000000000000000000000000000000000
000000000000000000000000000000000000000000000000000000000000000000000000000000000000000000000000000000000000000000000000000000000000000000000000000000000000000000000000000
000000000000000000000000000000000000000000000000000000000000000000000000000000000000000000000000000000000000000000000000000000000000000000000000000000000000000000000000000
000000000000000000000000000000000000000000000000000000000000000000000000000000000000000000000000000000000000000000000000000000000000000000000000000000000000000000000000000
000000000000000000000000000000000000000000000000000000000000000000000000000
000000000000000000000000000000000000000000000000000000000000000000000000000000000000000000000000000000000000000000000000000000000000000000000000000000000000000000000000000
000000000000000000000000000000000000000000000000000000000000000000000000000000000000000000000000000000000000000000000000000000000000000000000000000000000000000000000000000
000000000000000000000000000000000000000000000000000000000000000000000000000000000000000000000000000000000000000000000000000000000000000000000000000000000000000000000000000
000000000000000000000000000000000000000000000000000000000000000000000000000000000000000000000000000000000000000000000000000000000000000000000000000000000000000000000000000

0000000000000000000000000000000000000000000000000000000000000000000000000000000000000000000000000000000000000000000000000000000000000000000000000000000000000000000000000000000
0000000000000000000000000000000000000000000000000000000000000000000000000000000000000000000000000000000000000000000000000000000000000000000000000000000000000000000000000000000
0000000000000000000000000000000000000000000000000000000000000000000000000000000000000000000000000000000000000000000000000000000000000000000000000000000000000000000000000000000
0000000000000000000000000000000000000000000000000000000000000000000000000000000000000000000000000000000000000000000000000000000000000000000000000000000000000000000000000000000
0000000000000000000000000000000000000000000000000000000000000000000000000000000000000000000000000000000000000000000000000000000000000000000000000000000000000000000000000000000
0000000000000000000000000000000000000000000000000000000000000000000000000000000000000000000000000000000000000000000000000000000000000000000000000000000000000000000000000000000
0000000000000000000000000000000000000000000000000000000000000000000000000000000000000000000000000000000000000000000000000000000000000000000000000000000000000000000000000000000
0000000000000000000000000000000000000000000000000000000000000000000000000000000000000000000000000000000000000000000000000000000000000000000000000000000000000000000000000000000
000000000000000000000000000000000000000000000000000000000000000000000000000
>DNA_P13989
MWQINEVVLFDNDPYRILAIEDGQVVWMQISADKGVPQARAELLLMQYLDEGRLVRTDDPYVHLDLEEPSVDSVSFQKREEDYRKILPIINSKDRFDPKVRSELVEHVVQEHKVTKATVYKLLRRYWQRGQTPNALIPDYKNSGAPGERRSATGTAKIGRAREYGKGEGTKVTPE
IERLFRLTIEKHLLNQKGTKTTVAYRRFVDLFAQYFPRIPQEDYPTLRQFRYFYDREYPKAQRLKSRVKAGVYKKDVRPLSSTATSQALGPGSRYEIDATIADIYLVDHHDRQKIIGRPTLYIVIDVFSRMITGFYIGFENPSYVVAMQAFVNACSDKTAICAQHDIEISSSDWP
CVGLPDVLLADRGELMSHQVEALVSSFNVRVESAPPRRGDAKGIVESTFRTLQAEFKSFAPGIVEGSRIKSHGETDYRLDASLSVFEFTQIILRTILFRNNHLVMDKYDRDADFPTDLPSIPVQLWQWGMQHRTGSLRAVEQEQLRVALLPRRKVSISSFGVNLWGLYYSGSEIL
REGWLQRSTDIARPQHLEAAYDPVLVDTIYLFPQVGSRVFWRCNLTERSRQFKGLSFWEVWDIQAQEKHNKANAKQDELTKRRELEAFIQQTIQKANKLTPSTTEPKSTRIKQIKTNKKEAVTSERKKRAEHLKPSSSGDEAKVIPFNAVEADDQEDYSLPTYVPELFQDPPEKD
ES
0000000000000000000000000000000000000000000000000000000000000000000000000000000000000000000000000000000000000000000000000000000000000000000000000000000000000000000000000000000
0000000000000000000000000000000000000000000000000000000000000000000000000000000000000000000000000000000000000000000000000000000000000000000000000000000000000000000000000000000
0000000000000000000000000000000000000000000000000000000000000000000000000000000000000000000000000000000000000000000000000000000000000000000000000000000000000000000000000000000
0000000000000000000000000000000000000000000000000000000000000000000000000000000000000000000000000000000000000000000000000000000000000000000000000000000000000000000000000000000
00
0000000000000000000000000000000000000000000000000000000000000000000000000000000000000000000000000000000000000000000000000000000000000000000000000000000000000000000000000000000
0000000000000000000000000000000000000000000000000000000000000000000000000000000000000000000000000000000000000000000000000000000000000000000000000000000000000000000000000000000
0000000000000000000000000000000000000000000000000000000000000000000000000000000000000000000000000000000000000000000000000000000000000000000000000000000000000000000000000000000
0000000000000000000000000000000000000000000000000000000000000000000000000000000000000000000000000000000000000000000000000000000000000000000000000000000000000000000000000000000
00
0000000000000000000000000000000000000000000000000000000000000000000000000000000000000000000000000000000000000000000000000000000000000000000000000000000000000000000000000000000
0000000000000000000000000000000000000000000000000000000000000000000000000000000000000000000000000000000000000000000000000000000000000000000000000000000000000000000000000000000
0000000000000000000000000000000000000000000000000000000000000000000000000000000000000000000000000000000000000000000000000000000000000000000000000000000000000000000000000000000
0000000000000000000000000000000000000000000000000000000000000000000000000000000000000000000000000000000000000000000000000000000000000000000000000000000000000000000000000000000
00
0000000000000000000000000000000000000000000000000000000000000000000000000000000000000000000000000000000000000000000000000000000000000000000000000000000000000000000000000000000
0000000000000000000000000000000000000000000000000000000000000000000000000000000000000000000000000000000000000000000000000000000000000000000000000000000000000000000000000000000
0000000000000000000000000000000000000000000000000000000000000000000000000000000000000000000000000000000000000000000000000000000000000000000000000000000000000000000000000000000
0000000000000000000000000000000000000000000000000000000000000000000000000000000000000000000000000000000000000000000000000000000000000000000000000000000000000000000000000000000
00
0000000000000000000000000000000000000000000000000000000000000000000000000000000000000000000000000000000000000000000000000000000000000000000000000000000000000000000000000000000
0000000000000000000000000000000000000000000000000000000000000000000000000000000000000000000000000000000000000000000000000000000000000000000000000000000000000000000000000000000
0000000000000000000000000000000000000000000000000000000000000000000000000000000000000000000000000000000000000000000000000000000000000000000000000000000000000000000000000000000
0000000000000000000000000000000000000000000000000000000000000000000000000000000000000000000000000000000000000000000000000000000000000000000000000000000000000000000000000000000
00
0000000000000000000000000000000000000000000000000000000000000000000000000000000000000000000000000000000000000000000000000000000000000000000000000000000000000000000000000000000
0000000000000000000000000000000000000000000000000000000000000000000000000000000000000000000000000000000000000000000000000000000000000000000000000000000000000000000000000000000
0000000000000000000000000000000000000000000000000000000000000000000000000000000000000000000000000000000000000000000000000000000000000000000000000000000000000000000000000000000
0000000000000000000000000000000000000000000000000000000000000000000000000000000000000000000000000000000000000000000000000000000000000000000000000000000000000000000000000000000
00
0000000000000000000000000000000000000000000000000000000000000000000000000000000000000000000000000000000000000000000000000000000000000000000000000000000000000000000000000000000
0000000000000000000000000000000000000000000000000000000000000000000000000000000000000000000000000000000000000000000000000000000000000000000000000000000000000000000000000000000
0000000000000000000000000000000000000000000000000000000000000000000000000000000000000000000000000000000000000000000000000000000000000000000000000000000000000000000000000000000
0000000000000000000000000000000000000000000000000000000000000000000000000000000000000000000000000000000000000000000000000000000000000000000000000000000000000000000000000000000
00
>DNA_P16931
MQGRKGANTLGRSLEIDLLRSFVVIAEVRALSRAAARVGRTQSALSQQMKRLEDIVDQPLFQRTGRGVVLTHPGERLLVHAQRILRQHDEAMADLCGTGLTGTIRFGCPDDYAEVFLPPLLRQFSSQHPQAIVEIVCGPTPRLLEQLEKRAVDLAMISLPDDGANDDIIRREQLV
WIGYPGLEPAHFDPLPLALSDPDTLDHIAACDALHRAGRDYRVAYASSSLAGLIALVRSGQAFAVMTQTAVPADLAIVNGDPRLPPLPAVGITLKFDRKRPSHLTAAFAEHIRAVLPML
0000000000000000000000000000000000000000000000000000000000000000000000000000000000000000000000000000000000000000000000000000000000000000000000000000000000000000000000000000000
00000000000000000000000000000000000000000000000000000000000000000000000000000000000000000000000000000000000000000000000
0000000000000000000000000000000000000000000000000000000000000000000000000000000000000000000000000000000000000000000000000000000000000000000000000000000000000000000000000000000
00000000000000000000000000000000000000000000000000000000000000000000000000000000000000000000000000000000000000000000000
0000000000000000000000000000000000000000000000000000000000000000000000000000000000000000000000000000000000000000000000000000000000000000000000000000000000000000000000000000000
00000000000000000000000000000000000000000000000000000000000000000000000000000000000000000000000000000000000000000000000
0000000000000000000000000000000000000000000000000000000000000000000000000000000000000000000000000000000000000000000000000000000000000000000000000000000000000000000000000000000
00000000000000000000000000000000000000000000000000000000000000000000000000000000000000000000000000000000000000000000000
0000000000000000000000000000000000000000000000000000000000000000000000000000000000000000000000000000000000000000000000000000000000000000000000000000000000000000000000000000000
00000000000000000000000000000000000000000000000000000000000000000000000000000000000000000000000000000000000000000000000
0000000000000000000000000000000000000000000000000000000000000000000000000000000000000000000000000000000000000000000000000000000000000000000000000000000000000000000000000000000
00000000000000000000000000000000000000000000000000000000000000000000000000000000000000000000000000000000000000000000000
0000000000000000000000000000000000000000000000000000000000000000000000000000000000000000000000000000000000000000000000000000000000000000000000000000000000000000000000000000000
00000000000000000000000000000000000000000000000000000000000000000000000000000000000000000000000000000000000000000000000
>DNA_P25293

MSDPIRTKPKSSMQIDNAPTPHNTPASVLNPSYLKNGNPVRAQAQEQDDKIGTINEEDILANQPLLLQSIQDRLGSLVGQDSGYVGGLPKNVKEKLLSLKTLQSELFEVEKEFQVEMFELENKFLQKYKPIWEQRSRIISGQEQPKPEQIAKGQEIVESLNETELLVDEEEKAQN
DSEEEQVKGIPSFWLTALENLPIVCDTITDRDAEVLEYLQDIGLEYLTDGRPGFKLLFRFDSSANPFFTNDILCKTYFYQKELGYSGDFIYDHAEGCEISWKDNAHNVTVDLEMRKQRNKTTKQVRTIEKITPIESFFNFFDPPKIQNEDQDEELEEDLEERLALDYSIGEQLKD
KLIPRAVDWFTGAALEFEFEEDEEEADEDEDEEEDDDHGLEDDDGESAEEQDDFAGRPEQAPECKQS
0000000000000000000000000000000000000000000000000000000000000000000000000000000000000000000000000000000000000000000000000000000000000000000000000000000000000000000000000000000
0000000000000000000000000000000000000000000000000000000000000000000000000000000000000000000000000000000000000000000000000000000000000000000000000000000000000000000000000000000
0000000000000000000000000000000000000000000000000000000000000000000
0000000000000000000000000000000000000000000000000000000000000000000000000000000000000000000000000000000000000000000000000000000000000000000000000000000000000000000000000000000
0000000000000000000000000000000000000000000000000000000000000000000000000000000000000000000000000000000000000000000000000000000000000000000000000000000000000000000000000000000
0000000000000000000000000000000000000000000000000000000000000000000
0000000000000000000000000000000000000000000000000000000000000000000000000000000000000000000000000000000000000000000000000000000000000000000000000000000000000000000000000000000
0000000000000000000000000000000000000000000000000000000000000000000000000000000000000000000000000000000000000000000000000000000000000000000000000000000000000000000000000000000
0000000000000000000000000000000000000000000000000000000000000000000
0000000000000000000000000000000000000000000000000000000000000000000000000000000000000000000000000000000000000000000000000000000000000000000000000000000000000000000000000000000
0000000000000000000000000000000000000000000000000000000000000000000000000000000000000000000000000000000000000000000000000000000000000000000000000000000000000000000000000000000
0000000000000000000000000000000000000000000000000000000000000000000
0000000000000000000000000000000000000000000000000000000000000000000000000000000000000000000000000000000000000000000000000000000000000000000000000000000000000000000000000000000
0000000000000000000000000000000000000000000000000000000000000000000000000000000000000000000000000000000000000000000000000000000000000000000000000000000000000000000000000000000
0000000000000000000000000000000000000000000000000000000000000000000
0000000000000000000000000000000000000000000000000000000000000000000000000000000000000000000000000000000000000000000000000000000000000000000000000000000000000000000000000000000
0000000000000000000000000000000000000000000000000000000000000000000000000000000000000000000000000000000000000000000000000000000000000000000000000000000000000000000000000000000
0000000000000000000000000000000000000000000000000000000000000000000
0000000000000000000000000000000000000000000000000000000000000000000000000000000000000000000000000000000000000000000000000000000000000000000000000000000000000000000000000000000
0000000000000000000000000000000000000000000000000000000000000000000000000000000000000000000000000000000000000000000000000000000000000000000000000000000000000000000000000000000
0000000000000000000000000000000000000000000000000000000000000000000
>DNA_P27898
MGRTPCCEKVGLKRGRWTAEEDQLLANYIAEHGEGSWRSLPKNAGLLRCGKSCRLRWINYLRADVKRGNISKEEEDIIIKLHATLGNRWSLIASHLPGRTDNEIKNYWNSHLSRQIHTYRRKYTAGPDDTAIAIDMSKLQSADRRRGGRTPGRPPKASASRTKQADADQPGGEAK
GPAAAASSPRHSDVVNPGPNQPNSSSGSTGTAEEEGPSSEDASGPWVLEPIELGDLVWGEADSEMDALMPIGPGGTTRLPSKGLARSAARPRWTTCSTWTGMASRPICGAGRSRTSTARSCGRPPSRWKLLLLLLLLRRRPAPRTIASWRRSRLGSCPTRSDGSGHRTDQTDQIIG
SRVLARSLPSRGSWFRWPNNWEKNSTARAVKPPPCAPDVDACRVELLRI
0000000000000000000000000000000000000000000000000000000000000000000000000000000000000000000000000000000000000000000000000000000000000000000000000000000000000000000000000000000
0000000000000000000000000000000000000000000000000000000000000000000000000000000000000000000000000000000000000000000000000000000000000000000000000000000000000000000000000000000
0000000000000000000000000000000000000000000000000
0000000000000000000000000000000000000000000000000000000000000000000000000000000000000000000000000000000000000000000000000000000000000000000000000000000000000000000000000000000
0000000000000000000000000000000000000000000000000000000000000000000000000000000000000000000000000000000000000000000000000000000000000000000000000000000000000000000000000000000
0000000000000000000000000000000000000000000000000
0000000000000000000000000000000000000000000000000000000000000000000000000000000000000000000000000000000000000000000000000000000000000000000000000000000000000000000000000000000
0000000000000000000000000000000000000000000000000000000000000000000000000000000000000000000000000000000000000000000000000000000000000000000000000000000000000000000000000000000
0000000000000000000000000000000000000000000000000
0000000000000000000000000000000000000000000000000000000000000000000000000000000000000000000000000000000000000000000000000000000000000000000000000000000000000000000000000000000
0000000000000000000000000000000000000000000000000000000000000000000000000000000000000000000000000000000000000000000000000000000000000000000000000000000000000000000000000000000
0000000000000000000000000000000000000000000000000
0000000000000000000000000000000000000000000000000000000000000000000000000000000000000000000000000000000000000000000000000000000000000000000000000000000000000000000000000000000
0000000000000000000000000000000000000000000000000000000000000000000000000000000000000000000000000000000000000000000000000000000000000000000000000000000000000000000000000000000
0000000000000000000000000000000000000000000000000
0000000000000000000000000000000000000000000000000000000000000000000000000000000000000000000000000000000000000000000000000000000000000000000000000000000000000000000000000000000
0000000000000000000000000000000000000000000000000000000000000000000000000000000000000000000000000000000000000000000000000000000000000000000000000000000000000000000000000000000
0000000000000000000000000000000000000000000000000
0000000000000000000000000000000000000000000000000000000000000000000000000000000000000000000000000000000000000000000000000000000000000000000000000000000000000000000000000000000
0000000000000000000000000000000000000000000000000000000000000000000000000000000000000000000000000000000000000000000000000000000000000000000000000000000000000000000000000000000
0000000000000000000000000000000000000000000000000
>DNA_P32265
MTLISHDERLIKALAVAIVDRPRATLKELAEAAGVSKATLHRFCGTRDNLVQMLEDHGETVLNQIIQACDLEHAEPLEALQRLIKEHLTHRELLVFLVFQYRPDFLDPHGEGARWQSYLEALDAFFLRGQQKGVFRIDITAAVFTELFITLVYGMVDAERRGRAASSNSAHTLEQ
MFLHGASNPARS
0000000000000000000000000000000000000000000000000000000000000000000000000000000000000000000000000000000000000000000000000000000000000000000000000000000000000000000000000000000
000000000000
0000000000000000000000000000000000000000000000000000000000000000000000000000000000000000000000000000000000000000000000000000000000000000000000000000000000000000000000000000000
000000000000
0000000000000000000000000000000000000000000000000000000000000000000000000000000000000000000000000000000000000000000000000000000000000000000000000000000000000000000000000000000
000000000000
0000000000000000000000000000000000000000000000000000000000000000000000000000000000000000000000000000000000000000000000000000000000000000000000000000000000000000000000000000000
000000000000
0000000000000000000000000000000000000000000000000000000000000000000000000000000000000000000000000000000000000000000000000000000000000000000000000000000000000000000000000000000
000000000000
0000000000000000000000000000000000000000000000000000000000000000000000000000000000000000000000000000000000000000000000000000000000000000000000000000000000000000000000000000000
000000000000
0000000000000000000000000000000000000000000000000000000000000000000000000000000000000000000000000000000000000000000000000000000000000000000000000000000000000000000000000000000
000000000000
>DNA_P36093

MYHVPEMRLHYPLVNTQSNAAITPTRSYDNTLPSFNELSHQSTINLPFVQRETPNAYANVAQLATSPTQAKSGYYCRYYAVPFPTYPQQPQSPYQQAVLPYATIPNSNFQPSSFPVMAVMPPEVQFDGSFLNTLHPHTELPPIIQNTNDTSVARPNNLKSIAAASPTVTATTRTP
GVSSTSVLKPRVITTMWEDENTICYQVEANGISVVRRADNNMINGTKLLNVTKMTRGRRDGILRSEKVREVVKIGSMHLKGVWIPFERAYILAQREQILDHLYPLFVKDIESIVDARKPSNKASLTPKSSPAPIKQEPSDNKHEIATEIKPKSIDALSNGASTQGAGELPHLKIN
HIDTEAQTSRAKNELS
0000000000000000000000000000000000000000000000000000000000000000000000000000000000000000000000000000000000000000000000000000000000000000000000000000000000000000000000000000000
0000000000000000000000000000000000000000000000000000000000000000000000000000000000000000000000000000000000000000000000000000000000000000000000000000000000000000000000000000000
0000000000000000
0000000000000000000000000000000000000000000000000000000000000000000000000000000000000000000000000000000000000000000000000000000000000000000000000000000000000000000000000000000
0000000000000000000000000000000000000000000000000000000000000000000000000000000000000000000000000000000000000000000000000000000000000000000000000000000000000000000000000000000
0000000000000000
0000000000000000000000000000000000000000000000000000000000000000000000000000000000000000000000000000000000000000000000000000000000000000000000000000000000000000000000000000000
0000000000000000000000000000000000000000000000000000000000000000000000000000000000000000000000000000000000000000000000000000000000000000000000000000000000000000000000000000000
0000000000000000
0000000000000000000000000000000000000000000000000000000000000000000000000000000000000000000000000000000000000000000000000000000000000000000000000000000000000000000000000000000
0000000000000000000000000000000000000000000000000000000000000000000000000000000000000000000000000000000000000000000000000000000000000000000000000000000000000000000000000000000
0000000000000000
0000000000000000000000000000000000000000000000000000000000000000000000000000000000000000000000000000000000000000000000000000000000000000000000000000000000000000000000000000000
0000000000000000000000000000000000000000000000000000000000000000000000000000000000000000000000000000000000000000000000000000000000000000000000000000000000000000000000000000000
0000000000000000
0000000000000000000000000000000000000000000000000000000000000000000000000000000000000000000000000000000000000000000000000000000000000000000000000000000000000000000000000000000
0000000000000000000000000000000000000000000000000000000000000000000000000000000000000000000000000000000000000000000000000000000000000000000000000000000000000000000000000000000
0000000000000000
0000000000000000000000000000000000000000000000000000000000000000000000000000000000000000000000000000000000000000000000000000000000000000000000000000000000000000000000000000000
0000000000000000000000000000000000000000000000000000000000000000000000000000000000000000000000000000000000000000000000000000000000000000000000000000000000000000000000000000000
0000000000000000
>DNA_P38114
MNMDSGITSSHGSMDKTQKQSSEWAANQKHNQRVENTRVLMGPAVPAMPPVPSNFPPVPTGTIMSPQLSPFPDHRLRHHPLAHMMPADKNFLAYNMESFKSRVTKACDYCRKRKIRCTEIEPISGKCRNCIKYNKDCTFHFHEELKRRREEALNNKGNGKSVKKPRLDKENKFKD
ENFDIAVRSRNTSSTDSSPKLHTNLSQEYIGVSAGKSASDKEDTWPDFVPIDRTVLEKIELNHTKVAGKVFVLEEICKNMKGTIEKLAEKSKIDVIDKEYMKRPKRKQYSKALLTKQKMFHFRQNVLSHLTDEEFLSPINEMFTTTFKYSILQTKLVLDFSFRSASSPSSDNILY
PLPRLAIAKRLLKNIKCPSLASLLHIVDVDQCLQFADVHFDPAKGRLTSSQAFLLNICLCLGATVTNFEEKQELVDEDNHETYYFEKFELWRLRSFTFLNSVYYYHKLSVARADMTALKALLLLAKFAQQKISASSAVKVLSVAIKVALDLRLNLHSTYEDLELDEIIKRRRLWC
YCFSTDKFFSVVLSRPPFLKEENTDVLTDESYVELFRDKILPNLSIKYDDSKLEGVKDIVSVVNLLANHLEYVPYIQSYFLSRLSLIESQIYYSCFSIRTTLDDTLDEIIENVLENQKALDRMRDDLPTILSLENYKENMRILSLDSSKLDFEVSCCTTILLHLRWYHQKITLSL
FVISIIGDNLDQRESSRHDIAEIIRRSRLDFKRNCIEVLNILKDFEYYPTVQNEFLYFSLTTVFSMFLYLSEIMVNDEHAMETGYIIGLLRDTHTRMLGSEERCLSVHNLKWQTSLFFYTFFLRSTMEKFNLTSKYAKFYAFDSNYYEGVLNRLVKHTRESKDDMVELLKTSFIN
KEKMAAFGSFVTEDQEKMEVSFNIFNEITIQDLNFLQFSSIPKLWENKTLEPGEEYHHSNGTNTDNNETTGADDTDDNNNNNNNNNNKNGNNSSSTINNNNNNYSNSNNNDNDNNINDDDDDDDDDDDDDDDDDDDDNDDDYSNNGADDDEEDDDYDRSLFPTGLASLLDASYPE
RTANDYRDENEQSNKLFEKIEGHLEHGVFFYDRDFFFKNVCVKM
0000000000000000000000000000000000000000000000000000000000000000000000000000000000000000000000000000000000000000000000000000000000000000000000000000000000000000000000000000000
0000000000000000000000000000000000000000000000000000000000000000000000000000000000000000000000000000000000000000000000000000000000000000000000000000000000000000000000000000000
0000000000000000000000000000000000000000000000000000000000000000000000000000000000000000000000000000000000000000000000000000000000000000000000000000000000000000000000000000000
0000000000000000000000000000000000000000000000000000000000000000000000000000000000000000000000000000000000000000000000000000000000000000000000000000000000000000000000000000000
0000000000000000000000000000000000000000000000000000000000000000000000000000000000000000000000000000000000000000000000000000000000000000000000000000000000000000000000000000000
0000000000000000000000000000000000000000000000000000000000000000000000000000000000000000000000000000000000000000000000000000000000000000000000000000000000000000000000000000000
00000000000000000000000000000000000000000000
0000000000000000000000000000000000000000000000000000000000000000000000000000000000000000000000000000000000000000000000000000000000000000000000000000000000000000000000000000000
0000000000000000000000000000000000000000000000000000000000000000000000000000000000000000000000000000000000000000000000000000000000000000000000000000000000000000000000000000000
0000000000000000000000000000000000000000000000000000000000000000000000000000000000000000000000000000000000000000000000000000000000000000000000000000000000000000000000000000000
0000000000000000000000000000000000000000000000000000000000000000000000000000000000000000000000000000000000000000000000000000000000000000000000000000000000000000000000000000000
0000000000000000000000000000000000000000000000000000000000000000000000000000000000000000000000000000000000000000000000000000000000000000000000000000000000000000000000000000000
0000000000000000000000000000000000000000000000000000000000000000000000000000000000000000000000000000000000000000000000000000000000000000000000000000000000000000000000000000000
00000000000000000000000000000000000000000000
0000000000000000000000000000000000000000000000000000000000000000000000000000000000000000000000000000000000000000000000000000000000000000000000000000000000000000000000000000000
0000000000000000000000000000000000000000000000000000000000000000000000000000000000000000000000000000000000000000000000000000000000000000000000000000000000000000000000000000000
0000000000000000000000000000000000000000000000000000000000000000000000000000000000000000000000000000000000000000000000000000000000000000000000000000000000000000000000000000000
0000000000000000000000000000000000000000000000000000000000000000000000000000000000000000000000000000000000000000000000000000000000000000000000000000000000000000000000000000000
0000000000000000000000000000000000000000000000000000000000000000000000000000000000000000000000000000000000000000000000000000000000000000000000000000000000000000000000000000000
0000000000000000000000000000000000000000000000000000000000000000000000000000000000000000000000000000000000000000000000000000000000000000000000000000000000000000000000000000000
00000000000000000000000000000000000000000000
0000000000000000000000000000000000000000000000000000000000000000000000000000000000000000000000000000000000000000000000000000000000000000000000000000000000000000000000000000000
0000000000000000000000000000000000000000000000000000000000000000000000000000000000000000000000000000000000000000000000000000000000000000000000000000000000000000000000000000000
0000000000000000000000000000000000000000000000000000000000000000000000000000000000000000000000000000000000000000000000000000000000000000000000000000000000000000000000000000000
0000000000000000000000000000000000000000000000000000000000000000000000000000000000000000000000000000000000000000000000000000000000000000000000000000000000000000000000000000000
0000000000000000000000000000000000000000000000000000000000000000000000000000000000000000000000000000000000000000000000000000000000000000000000000000000000000000000000000000000
0000000000000000000000000000000000000000000000000000000000000000000000000000000000000000000000000000000000000000000000000000000000000000000000000000000000000000000000000000000
00000000000000000000000000000000000000000000
0000000000000000000000000000000000000000000000000000000000000000000000000000000000000000000000000000000000000000000000000000000000000000000000000000000000000000000000000000000
0000000000000000000000000000000000000000000000000000000000000000000000000000000000000000000000000000000000000000000000000000000000000000000000000000000000000000000000000000000
0000000000000000000000000000000000000000000000000000000000000000000000000000000000000000000000000000000000000000000000000000000000000000000000000000000000000000000000000000000
0000000000000000000000000000000000000000000000000000000000000000000000000000000000000000000000000000000000000000000000000000000000000000000000000000000000000000000000000000000
0000000000000000000000000000000000000000000000000000000000000000000000000000000000000000000000000000000000000000000000000000000000000000000000000000000000000000000000000000000
0000000000000000000000000000000000000000000000000000000000000000000000000000000000000000000000000000000000000000000000000000000000000000000000000000000000000000000000000000000
00000000000000000000000000000000000000000000
0000000000000000000000000000000000000000000000000000000000000000000000000000000000000000000000000000000000000000000000000000000000000000000000000000000000000000000000000000000
0000000000000000000000000000000000000000000000000000000000000000000000000000000000000000000000000000000000000000000000000000000000000000000000000000000000000000000000000000000

00
00
00
00
00000000000000000000000000000000000000000000000000
00
00
00
00
00
00
00000000000000000000000000000000000000000000000000
>DNA_P40969
MFNRTTQLKSKHPCSVCTRRKVKCDRMIPCGNCRKRGQDSECMKSTKLITASSSKEYLPDLLLFWQNYEYWITNIGLYKTKQRDLTRTPANLDTDTEECMFWMNYLQKDQSFQLMNFAMENLGALYFGSIGDISELYLRVEQYWDRRADKNHSVDGKYWDALIWSVFTMCIYYMP
VEKLAEIFSVYPLHEYLGSNKRLNWEDGMQLVMCQNFARCSLFQLKQCDFMAHPDIRLVQAYLILATTTFPYDEPLLANSLLTQCIHTFKNFHVDDFRPLLNDDPVESIAKVTLGRIFYRLCGCDYLQSGPRKPIALHTEVSSLLQHAAYLQDLPNVDVYREENSTEVLYWKIIS
LDRDLDQYLNKSSKPPLKTLDAIRRELDIFQYKVDSLEEDFRSNNSRFQKFIALFQISTVSWKLFKMYLIYYDTADSLLKVIHYSKVIISLIVNNFHAKSEFFNRHPMVMQTITRVVSFISFYQIFVESAAVKQLLVDLTELTANLPTIFGSKLDKLVYLTERLSKLKLLWDKVQ
LLDSGDSFYHPVFKILQNDIKIIELKNDEMFSLIKGLGSLVPLNKLRQESLLEEEDENNTEPSDFRTIVEEFQSEYNISDILS
00
00
00
00000000000000000000000000000000000000000000000000000000000000000000000000000000000000000000000
00
00
00
00000000000000000000000000000000000000000000000000000000000000000000000000000000000000000000000
00
00
00
00000000000000000000000000000000000000000000000000000000000000000000000000000000000000000000000
00
00
00
00000000000000000000000000000000000000000000000000000000000000000000000000000000000000000000000
00
00
00
00000000000000000000000000000000000000000000000000000000000000000000000000000000000000000000000
00
00
00
00000000000000000000000000000000000000000000000000000000000000000000000000000000000000000000000
00
00
00
00000000000000000000000000000000000000000000000000000000000000000000000000000000000000000000000
>DNA_P44487
MSHITKIKNWNTNLRKNMENIVNRTYTRIGQLLKQDISQGIYSIGDKLPTEREISEKFGVSRTIVREAMVMLEVEKLVEVKKGSGVYVVRTPESIHMEHSDLPDVGPFELLQARQLLESSIAEFAALQATKKDILNLKQILNREKELLTQNQDDYSADKDFHLALAEITQNDVLV
KLQEQLWQYRFNSAMWAQLHSRILQNDYHHLWIEDHQTILSAIQKKNANEARKAMWQHLENVKVKLFELSDVEDPHFDGYLFNTNPVVVGI
00
00000000000000000000000000000000000000000000000000000000000000000000000000000000000000000000000000000000
00
00000000000000000000000000000000000000000000000000000000000000000000000000000000000000000000000000000000
00
00000000000000000000000000000000000000000000000000000000000000000000000000000000000000000000000000000000
00
00000000000000000000000000000000000000000000000000000000000000000000000000000000000000000000000000000000
00
00000000000000000000000000000000000000000000000000000000000000000000000000000000000000000000000000000000
00
00000000000000000000000000000000000000000000000000000000000000000000000000000000000000000000000000000000
00
00000000000000000000000000000000000000000000000000000000000000000000000000000000000000000000000000000000
>DNA_P47792
MYSMLEHDMKSAVPPTHQSAQGMTQLNGGVTHGSAKPAVNSQQQQSSDPMDKVKRPMNAFMVWSRGQRRKMAQENPKMHNSEISKRLGAEWKLLTDAEKRPFIDEAKRLRALHMKEYPDYKYKPRRKTKPVLKKDNPAAKYPLSAGNLLAAAAAQGSGGSPRMDSYGWGHTGGYP
GMQTDALGYSQQLHRYDLSALQYPSAMATAQTYMNGANSYNPMSYSSTPQQPSPVMSMVKPEQVSHSPTGAHSHQRGPLQGDLRDMISMYIPGGDTTDSGAQRGYSNIQQHYLTGTAPLTHI
00
0000000000000000000000000000000000000000000000000000000000000000000000000000000000000000000000000000000000000000000000000000000000000000000
00
0000000000000000000000000000000000000000000000000000000000000000000000000000000000000000000000000000000000000000000000000000000000000000000

0000000000000000000000000000000000000000000000000000000000000000000000000000000000000000000000000000000000000000000000000000000000000000000000000000000000000000000000000000000
00000000000000000000000000000000000000000000000000000000000000000000000000000000000000000000000000000000000000000000000000
0000000000000000000000000000000000000000000000000000000000000000000000000000000000000000000000000000000000000000000000000000000000000000000000000000000000000000000000000000000
00000000000000000000000000000000000000000000000000000000000000000000000000000000000000000000000000000000000000000000000000
0000000000000000000000000000000000000000000000000000000000000000000000000000000000000000000000000000000000000000000000000000000000000000000000000000000000000000000000000000000
00000000000000000000000000000000000000000000000000000000000000000000000000000000000000000000000000000000000000000000000000
0000000000000000000000000000000000000000000000000000000000000000000000000000000000000000000000000000000000000000000000000000000000000000000000000000000000000000000000000000000
00000000000000000000000000000000000000000000000000000000000000000000000000000000000000000000000000000000000000000000000000
0000000000000000000000000000000000000000000000000000000000000000000000000000000000000000000000000000000000000000000000000000000000000000000000000000000000000000000000000000000
00000000000000000000000000000000000000000000000000000000000000000000000000000000000000000000000000000000000000000000000000
>DNA_P48786
MEEISDPKPNALEQVLPTVPNGKCTAPVQMESLAVDVQKVSGEAKVRICSCWCEIVRSPEDLTKLVPCNDFAEDIKLFDSDPMQQEAESSIGIPLIPKQVTMSHNHDHESGSEMVSNEVMQENHVIATENTYQKSDFDRINMGQKETMPEEVIHKSFLESSTSSIDILLNNHNSY
QSGLPPENAVTDCKQVQLGHRSDDAIKNSGLVELVIGQKNVAKSPSQLVETGKRGRGRPRKVQTGLEQLVIGQKTAAKSSSQLGDTGKRSRGRPRKVQNSPTSFLENINMEQKETIPEQVTQNSILESLTIPTDNQSRTYNSDQSELPPENAAKNCNHAQFGHQSDDTTKISGFK
ELVIGQETVAKSPSQLVDAGKRGRGRPRKVQTGLEQLVPVQETAAKSSSQLGDTGKRSRGRPRKVQDSPTSLGGNVKVVPEKGKDSQELSVNSSRSLRSRSQEKSIEPDVNNIVADEGADREKPRKKRKKRMEENRVDEFCRIRTHLRYLLHRIKYEKNFLDAYSGEGWKGQSLD
KIKPEKELKRAKAEIFGRKLKIRDLFQRLDLARSEGRLPEILFDSRGEIDSEDIFCAKCGSKDVTLSNDIILCDGACDRGFHQFCLDPPLLKEYIPPDDEGWLCPGCECKIDCIKLLNDSQETNILLGDSWEKVFAEEAAAAASGKNLDDNSGLPSDDSEDDDYDPGGPDLDEKV
QGDDSSTDESDYQSESDDMQVIRQKNSRGLPSDDSEDDEYDPSGLVTDQMYKDSSCSDFTSDSEDFTGVFDDYKDTGKAQGPLASTPDHVRNNEEGCGHPEQGDTAPLYPRRQVESLDYKKLNDIEFSKMCDILDILSSQLDVIICTGNQEEYGNTSSDSSDEDYMVTSSPDKNN
SDKEATAMERGRESGDLELDQKARESTHNRRYIKKFAVEGTDSFLSRSCEDSAAPVAGSKSTSKTLHGEHATQRLLQSFKENQYPQRAVKESLAAELALSVRQVSNWFNNRRWSFRHSSRIGSDVAKFDSNDTPRQKSIDMSGPSLKSVLDSATYSEIEKKEQDTASLGLTEGCD
RYMTLNMVADEGNVHTPCIAETREEKTEVGIKPQQNPL
0000000000000000000000000000000000000000000000000000000000000000000000000000000000000000000000000000000000000000000000000000000000000000000000000000000000000000000000000000000
0000000000000000000000000000000000000000000000000000000000000000000000000000000000000000000000000000000000000000000000000000000000000000000000000000000000000000000000000000000
0000000000000000000000000000000000000000000000000000000000000000000000000000000000000000000000000000000000000000000000000000000000000000000000000000000000000000000000000000000
0000000000000000000000000000000000000000000000000000000000000000000000000000000000000000000000000000000000000000000000000000000000000000000000000000000000000000000000000000000
0000000000000000000000000000000000000000000000000000000000000000000000000000000000000000000000000000000000000000000000000000000000000000000000000000000000000000000000000000000
0000000000000000000000000000000000000000000000000000000000000000000000000000000000000000000000000000000000000000000000000000000000000000000000000000000000000000000000000000000
00000000000000000000000000000000000000
0000000000000000000000000000000000000000000000000000000000000000000000000000000000000000000000000000000000000000000000000000000000000000000000000000000000000000000000000000000
0000000000000000000000000000000000000000000000000000000000000000000000000000000000000000000000000000000000000000000000000000000000000000000000000000000000000000000000000000000
0000000000000000000000000000000000000000000000000000000000000000000000000000000000000000000000000000000000000000000000000000000000000000000000000000000000000000000000000000000
0000000000000000000000000000000000000000000000000000000000000000000000000000000000000000000000000000000000000000000000000000000000000000000000000000000000000000000000000000000
0000000000000000000000000000000000000000000000000000000000000000000000000000000000000000000000000000000000000000000000000000000000000000000000000000000000000000000000000000000
0000000000000000000000000000000000000000000000000000000000000000000000000000000000000000000000000000000000000000000000000000000000000000000000000000000000000000000000000000000
00000000000000000000000000000000000000
0000000000000000000000000000000000000000000000000000000000000000000000000000000000000000000000000000000000000000000000000000000000000000000000000000000000000000000000000000000
0000000000000000000000000000000000000000000000000000000000000000000000000000000000000000000000000000000000000000000000000000000000000000000000000000000000000000000000000000000
0000000000000000000000000000000000000000000000000000000000000000000000000000000000000000000000000000000000000000000000000000000000000000000000000000000000000000000000000000000
0000000000000000000000000000000000000000000000000000000000000000000000000000000000000000000000000000000000000000000000000000000000000000000000000000000000000000000000000000000
0000000000000000000000000000000000000000000000000000000000000000000000000000000000000000000000000000000000000000000000000000000000000000000000000000000000000000000000000000000
0000000000000000000000000000000000000000000000000000000000000000000000000000000000000000000000000000000000000000000000000000000000000000000000000000000000000000000000000000000
00000000000000000000000000000000000000
0000000000000000000000000000000000000000000000000000000000000000000000000000000000000000000000000000000000000000000000000000000000000000000000000000000000000000000000000000000
0000000000000000000000000000000000000000000000000000000000000000000000000000000000000000000000000000000000000000000000000000000000000000000000000000000000000000000000000000000
0000000000000000000000000000000000000000000000000000000000000000000000000000000000000000000000000000000000000000000000000000000000000000000000000000000000000000000000000000000
0000000000000000000000000000000000000000000000000000000000000000000000000000000000000000000000000000000000000000000000000000000000000000000000000000000000000000000000000000000
0000000000000000000000000000000000000000000000000000000000000000000000000000000000000000000000000000000000000000000000000000000000000000000000000000000000000000000000000000000
0000000000000000000000000000000000000000000000000000000000000000000000000000000000000000000000000000000000000000000000000000000000000000000000000000000000000000000000000000000
00000000000000000000000000000000000000
0000000000000000000000000000000000000000000000000000000000000000000000000000000000000000000000000000000000000000000000000000000000000000000000000000000000000000000000000000000
0000000000000000000000000000000000000000000000000000000000000000000000000000000000000000000000000000000000000000000000000000000000000000000000000000000000000000000000000000000
0000000000000000000000000000000000000000000000000000000000000000000000000000000000000000000000000000000000000000000000000000000000000000000000000000000000000000000000000000000
0000000000000000000000000000000000000000000000000000000000000000000000000000000000000000000000000000000000000000000000000000000000000000000000000000000000000000000000000000000
0000000000000000000000000000000000000000000000000000000000000000000000000000000000000000000000000000000000000000000000000000000000000000000000000000000000000000000000000000000
0000000000000000000000000000000000000000000000000000000000000000000000000000000000000000000000000000000000000000000000000000000000000000000000000000000000000000000000000000000
00000000000000000000000000000000000000
0000000000000000000000000000000000000000000000000000000000000000000000000000000000000000000000000000000000000000000000000000000000000000000000000000000000000000000000000000000
0000000000000000000000000000000000000000000000000000000000000000000000000000000000000000000000000000000000000000000000000000000000000000000000000000000000000000000000000000000
0000000000000000000000000000000000000000000000000000000000000000000000000000000000000000000000000000000000000000000000000000000000000000000000000000000000000000000000000000000
0000000000000000000000000000000000000000000000000000000000000000000000000000000000000000000000000000000000000000000000000000000000000000000000000000000000000000000000000000000
0000000000000000000000000000000000000000000000000000000000000000000000000000000000000000000000000000000000000000000000000000000000000000000000000000000000000000000000000000000
0000000000000000000000000000000000000000000000000000000000000000000000000000000000000000000000000000000000000000000000000000000000000000000000000000000000000000000000000000000
00000000000000000000000000000000000000
0000000000000000000000000000000000000000000000000000000000000000000000000000000000000000000000000000000000000000000000000000000000000000000000000000000000000000000000000000000
0000000000000000000000000000000000000000000000000000000000000000000000000000000000000000000000000000000000000000000000000000000000000000000000000000000000000000000000000000000
0000000000000000000000000000000000000000000000000000000000000000000000000000000000000000000000000000000000000000000000000000000000000000000000000000000000000000000000000000000
0000000000000000000000000000000000000000000000000000000000000000000000000000000000000000000000000000000000000000000000000000000000000000000000000000000000000000000000000000000
0000000000000000000000000000000000000000000000000000000000000000000000000000000000000000000000000000000000000000000000000000000000000000000000000000000000000000000000000000000
0000000000000000000000000000000000000000000000000000000000000000000000000000000000000000000000000000000000000000000000000000000000000000000000000000000000000000000000000000000
00000000000000000000000000000000000000
>DNA_P70459

MKTPADTGFAFPDWAYKPESSPGSRQIQLWHFILELLRKEEYQGVIAWQGDYGEFVIKDPDEVARLWGVRKCKPQMNYDKLSRALRYYYNKRILHKTKGKRFTYKFNFNKLVLVNYPFIDMGLAGGAVPQSAPPVPSGGSHFRFPPSTPSEVLSPTEDPRSPPACSSSSSSLFSA
VVARRLGRGSVSDCSDGTSELEEPLGEDPRARPPGPPELGAFRGPPLARLPHDPGVFRVYPRPRGGPEPLSPFPVSPLAGPGSLLPPQLSPALPMTPTHLAYTPSPTLSPMYPSGGGGPSGSGGGSHFSFSPEDMKRYLQAHTQSVYNYHLSPRAFLHYPGLVVPQPQRPDKCPL
PPMAPETPPVPSSASSSSSSSSSPFKFKLQPPPLGRRQRAAGEKAPGGTDKSSGGSGSGGLAEGAGAVAPPPPPPQIKVEPISEGESEEVEVTDISDEDEEDGEVFKTPRAPPAPPKPEPGEAPGVAQCMPLKLRFKRRWSEDCRLEGGGCLSGGPEDEGEDKKVRGDVGPGESG
GPLTPRRVSSDLQHATAQLSLEHRDS
0000000000000000000000000000000000000000000000000000000000000000000000000000000000000000000000000000000000000000000000000000000000000000000000000000000000000000000000000000000
0000000000000000000000000000000000000000000000000000000000000000000000000000000000000000000000000000000000000000000000000000000000000000000000000000000000000000000000000000000
0000000000000000000000000000000000000000000000000000000000000000000000000000000000000000000000000000000000000000000000000000000000000000000000000000000000000000000000000000000
00000000000000000000000000
0000000000000000000000000000000000000000000000000000000000000000000000000000000000000000000000000000000000000000000000000000000000000000000000000000000000000000000000000000000
0000000000000000000000000000000000000000000000000000000000000000000000000000000000000000000000000000000000000000000000000000000000000000000000000000000000000000000000000000000
0000000000000000000000000000000000000000000000000000000000000000000000000000000000000000000000000000000000000000000000000000000000000000000000000000000000000000000000000000000
00000000000000000000000000
0000000000000000000000000000000000000000000000000000000000000000000000000000000000000000000000000000000000000000000000000000000000000000000000000000000000000000000000000000000
0000000000000000000000000000000000000000000000000000000000000000000000000000000000000000000000000000000000000000000000000000000000000000000000000000000000000000000000000000000
0000000000000000000000000000000000000000000000000000000000000000000000000000000000000000000000000000000000000000000000000000000000000000000000000000000000000000000000000000000
00000000000000000000000000
0000000000000000000000000000000000000000000000000000000000000000000000000000000000000000000000000000000000000000000000000000000000000000000000000000000000000000000000000000000
0000000000000000000000000000000000000000000000000000000000000000000000000000000000000000000000000000000000000000000000000000000000000000000000000000000000000000000000000000000
0000000000000000000000000000000000000000000000000000000000000000000000000000000000000000000000000000000000000000000000000000000000000000000000000000000000000000000000000000000
00000000000000000000000000
0000000000000000000000000000000000000000000000000000000000000000000000000000000000000000000000000000000000000000000000000000000000000000000000000000000000000000000000000000000
0000000000000000000000000000000000000000000000000000000000000000000000000000000000000000000000000000000000000000000000000000000000000000000000000000000000000000000000000000000
0000000000000000000000000000000000000000000000000000000000000000000000000000000000000000000000000000000000000000000000000000000000000000000000000000000000000000000000000000000
00000000000000000000000000
0000000000000000000000000000000000000000000000000000000000000000000000000000000000000000000000000000000000000000000000000000000000000000000000000000000000000000000000000000000
0000000000000000000000000000000000000000000000000000000000000000000000000000000000000000000000000000000000000000000000000000000000000000000000000000000000000000000000000000000
0000000000000000000000000000000000000000000000000000000000000000000000000000000000000000000000000000000000000000000000000000000000000000000000000000000000000000000000000000000
00000000000000000000000000
0000000000000000000000000000000000000000000000000000000000000000000000000000000000000000000000000000000000000000000000000000000000000000000000000000000000000000000000000000000
0000000000000000000000000000000000000000000000000000000000000000000000000000000000000000000000000000000000000000000000000000000000000000000000000000000000000000000000000000000
0000000000000000000000000000000000000000000000000000000000000000000000000000000000000000000000000000000000000000000000000000000000000000000000000000000000000000000000000000000
00000000000000000000000000
>DNA_P9WJA7
MSIDFPLGDDLAGYIAEAIAADPSFKGTLEDAEEARRLVDALIALRKHCQLSQVEVAKRMGVRQPTVSGFEKEPSDPKLSTLQRYARALDARLRLVLEVPTLREVPTWHRLSSYRGSARDHQVRVGADKEILMQTNWARHISVRQVEVA
00000000000000000000000000000000000000000000000000000000000000000000000000000000000000000000000000000000000000000000000000000000000000000000000
00000000000000000000000000000000000000000000000000000000000000000000000000000000000000000000000000000000000000000000000000000000000000000000000
00000000000000000000000000000000000000000000000000000000000000000000000000000000000000000000000000000000000000000000000000000000000000000000000
00000000000000000000000000000000000000000000000000000000000000000000000000000000000000000000000000000000000000000000000000000000000000000000000
00000000000000000000000000000000000000000000000000000000000000000000000000000000000000000000000000000000000000000000000000000000000000000000000
00000000000000000000000000000000000000000000000000000000000000000000000000000000000000000000000000000000000000000000000000000000000000000000000
00000000000000000000000000000000000000000000000000000000000000000000000000000000000000000000000000000000000000000000000000000000000000000000000
>DNA_Q01593
MKSLHVAANAGDLAEDCGILGGDADDTVLMDGIDEVGREIWLDDHGGDNNHVHGHQDDDLIVHHDPSIFYGDLPTLPDFPCMSSSSSSSTSPAPVNAIVSSASSSSAASSSTSSAASWAILRSDGEDPTPNQNQYASGNCDDSSGALQSTASMEIPLDSSQGFGCGEGGGDCIDM
METFGYMDLLDSNEFFDTSAIFSQDDDTQNPNLMDQTLERQEDQVVVPMMENNSGGDMQMMNSSLEQDDDLAAVFLEWLKNNKETVSAEDLRKVKIKKATIESAARRLGGGKEAMKQLLKLILEWVQTNHLQRRRTTTTTTNLSYQQSFQQDPFQNPNPNNNNLIPPSDQTCFSP
STWVPPPPQQQAFVSDPGFGYMPAPNYPPQPEFLPLLESPPSWPPPPQSGPMPHQQFPMPPTSQYNQFGDPTGFNGYNMNPYQYPYVPAGQMRDQRLLRLCSSATKEARKKRMARQRRFLSHHHRHNNNNNNNNNNQQNQTQIGETCAAVAPQLNPVATTATGGTWMYWPNVPAV
PPQLPPVMETQLPTMDRAGSASAMPRQQVVPDRRQGWKPEKNLRFLLQKVLKQSDVGNLGRIVLPKKEAETHLPELEARDGISLAMEDIGTSRVWNMRYRFWPNNKSRMYLLENTGDFVKTNGLQEGDFIVIYSDVKCGKYLIRGVKVRQPSGQKPEAPPSSAATKRQNKSQRNI
NNNSPSANVVVASPTSQTVK
0000000000000000000000000000000000000000000000000000000000000000000000000000000000000000000000000000000000000000000000000000000000000000000000000000000000000000000000000000000
0000000000000000000000000000000000000000000000000000000000000000000000000000000000000000000000000000000000000000000000000000000000000000000000000000000000000000000000000000000
0000000000000000000000000000000000000000000000000000000000000000000000000000000000000000000000000000000000000000000000000000000000000000000000000000000000000000000000000000000
0000000000000000000000000000000000000000000000000000000000000000000000000000000000000000000000000000000000000000000000000000000000000000000000000000000000000000000000000000000
00000000000000000000
0000000000000000000000000000000000000000000000000000000000000000000000000000000000000000000000000000000000000000000000000000000000000000000000000000000000000000000000000000000
0000000000000000000000000000000000000000000000000000000000000000000000000000000000000000000000000000000000000000000000000000000000000000000000000000000000000000000000000000000
0000000000000000000000000000000000000000000000000000000000000000000000000000000000000000000000000000000000000000000000000000000000000000000000000000000000000000000000000000000
0000000000000000000000000000000000000000000000000000000000000000000000000000000000000000000000000000000000000000000000000000000000000000000000000000000000000000000000000000000
00000000000000000000
0000000000000000000000000000000000000000000000000000000000000000000000000000000000000000000000000000000000000000000000000000000000000000000000000000000000000000000000000000000
0000000000000000000000000000000000000000000000000000000000000000000000000000000000000000000000000000000000000000000000000000000000000000000000000000000000000000000000000000000
0000000000000000000000000000000000000000000000000000000000000000000000000000000000000000000000000000000000000000000000000000000000000000000000000000000000000000000000000000000
0000000000000000000000000000000000000000000000000000000000000000000000000000000000000000000000000000000000000000000000000000000000000000000000000000000000000000000000000000000
00000000000000000000
0000000000000000000000000000000000000000000000000000000000000000000000000000000000000000000000000000000000000000000000000000000000000000000000000000000000000000000000000000000
0000000000000000000000000000000000000000000000000000000000000000000000000000000000000000000000000000000000000000000000000000000000000000000000000000000000000000000000000000000
0000000000000000000000000000000000000000000000000000000000000000000000000000000000000000000000000000000000000000000000000000000000000000000000000000000000000000000000000000000
0000000000000000000000000000000000000000000000000000000000000000000000000000000000000000000000000000000000000000000000000000000000000000000000000000000000000000000000000000000
00000000000000000000
0000000000000000000000000000000000000000000000000000000000000000000000000000000000000000000000000000000000000000000000000000000000000000000000000000000000000000000000000000000
0000000000000000000000000000000000000000000000000000000000000000000000000000000000000000000000000000000000000000000000000000000000000000000000000000000000000000000000000000000

0000000000000000000000000000000000000000000000000000000000000000000000000000000000000000000000000000000000000000000000000000000000000000000000000000000000000000000000000000000
0000000000000000000000000000000000000000000000000000000000000000000000000000000000000000000000000000000000000000000000000000000000000000000000000000000000000000000000000000000
00000000000000000000
0000000000000000000000000000000000000000000000000000000000000000000000000000000000000000000000000000000000000000000000000000000000000000000000000000000000000000000000000000000
0000000000000000000000000000000000000000000000000000000000000000000000000000000000000000000000000000000000000000000000000000000000000000000000000000000000000000000000000000000
0000000000000000000000000000000000000000000000000000000000000000000000000000000000000000000000000000000000000000000000000000000000000000000000000000000000000000000000000000000
0000000000000000000000000000000000000000000000000000000000000000000000000000000000000000000000000000000000000000000000000000000000000000000000000000000000000000000000000000000
00000000000000000000
0000000000000000000000000000000000000000000000000000000000000000000000000000000000000000000000000000000000000000000000000000000000000000000000000000000000000000000000000000000
0000000000000000000000000000000000000000000000000000000000000000000000000000000000000000000000000000000000000000000000000000000000000000000000000000000000000000000000000000000
0000000000000000000000000000000000000000000000000000000000000000000000000000000000000000000000000000000000000000000000000000000000000000000000000000000000000000000000000000000
0000000000000000000000000000000000000000000000000000000000000000000000000000000000000000000000000000000000000000000000000000000000000000000000000000000000000000000000000000000
00000000000000000000
>DNA_Q03513
MPSPTTVPVATAGRLAEPYIDPAAQVHAIASIIGDVRIAAGVRVAAGVSIRADEGAPFQVGKESILQEGAVIHGLEYGRVLGDDQADYSVWIGQRVAITHKALIHGPAYLGDDCFVGFRSTVFNARVGAGSVIMMHALVQDVEIPPGRYVPSGAIITTQQQADRLPEVRPEDREF
ARHIIGSPPVIVRSTPAATADFHSTPTPSPLRPSSSEATTVSAYNGQGRLSSEVITQVRSLLNQGYRIGTEHADKRRFRTSSWQPCAPIQSTNERQVLSELENCLSEHEGEYVRLLGIDTNTRSRVFEALIQRPDGSVPESLGSQPVAVASGGGRQSSYASVSGNLSAEVVNKVR
NLLAQGYRIGTEHADKRRFRTSSWQSCAPIQSSNERQVLAELENCLSEHEGEYVRLLGIDTASRSRVFEALIQDPQGPVGSAKAAAAPVSSATPSSHSYTSNGSSSSDVAGQVRGLLAQGYRISAEVADKRRFQTSSWQSLPALSGQSEATVLPALESILQEHKGKYVRLIGIDP
AARRRVAELLIQKP
0000000000000000000000000000000000000000000000000000000000000000000000000000000000000000000000000000000000000000000000000000000000000000000000000000000000000000000000000000000
0000000000000000000000000000000000000000000000000000000000000000000000000000000000000000000000000000000000000000000000000000000000000000000000000000000000000000000000000000000
0000000000000000000000000000000000000000000000000000000000000000000000000000000000000000000000000000000000000000000000000000000000000000000000000000000000000000000000000000000
00000000000000
0000000000000000000000000000000000000000000000000000000000000000000000000000000000000000000000000000000000000000000000000000000000000000000000000000000000000000000000000000000
0000000000000000000000000000000000000000000000000000000000000000000000000000000000000000000000000000000000000000000000000000000000000000000000000000000000000000000000000000000
0000000000000000000000000000000000000000000000000000000000000000000000000000000000000000000000000000000000000000000000000000000000000000000000000000000000000000000000000000000
00000000000000
0000000000000000000000000000000000000000000000000000000000000000000000000000000000000000000000000000000000000000000000000000000000000000000000000000000000000000000000000000000
0000000000000000000000000000000000000000000000000000000000000000000000000000000000000000000000000000000000000000000000000000000000000000000000000000000000000000000000000000000
0000000000000000000000000000000000000000000000000000000000000000000000000000000000000000000000000000000000000000000000000000000000000000000000000000000000000000000000000000000
00000000000000
0000000000000000000000000000000000000000000000000000000000000000000000000000000000000000000000000000000000000000000000000000000000000000000000000000000000000000000000000000000
0000000000000000000000000000000000000000000000000000000000000000000000000000000000000000000000000000000000000000000000000000000000000000000000000000000000000000000000000000000
0000000000000000000000000000000000000000000000000000000000000000000000000000000000000000000000000000000000000000000000000000000000000000000000000000000000000000000000000000000
00000000000000
0000000000000000000000000000000000000000000000000000000000000000000000000000000000000000000000000000000000000000000000000000000000000000000000000000000000000000000000000000000
0000000000000000000000000000000000000000000000000000000000000000000000000000000000000000000000000000000000000000000000000000000000000000000000000000000000000000000000000000000
0000000000000000000000000000000000000000000000000000000000000000000000000000000000000000000000000000000000000000000000000000000000000000000000000000000000000000000000000000000
00000000000000
0000000000000000000000000000000000000000000000000000000000000000000000000000000000000000000000000000000000000000000000000000000000000000000000000000000000000000000000000000000
0000000000000000000000000000000000000000000000000000000000000000000000000000000000000000000000000000000000000000000000000000000000000000000000000000000000000000000000000000000
0000000000000000000000000000000000000000000000000000000000000000000000000000000000000000000000000000000000000000000000000000000000000000000000000000000000000000000000000000000
00000000000000
0000000000000000000000000000000000000000000000000000000000000000000000000000000000000000000000000000000000000000000000000000000000000000000000000000000000000000000000000000000
0000000000000000000000000000000000000000000000000000000000000000000000000000000000000000000000000000000000000000000000000000000000000000000000000000000000000000000000000000000
0000000000000000000000000000000000000000000000000000000000000000000000000000000000000000000000000000000000000000000000000000000000000000000000000000000000000000000000000000000
00000000000000
>DNA_Q04052
MSAKKRPSGNAAFELPKRRRTYQACISCRSRKVKCDLGPVDNPHDPPCARCKRELKKCIFSSNKGTSNDLPPNSINAISLPSLGKSKQEIQNDSTSPILSDVPLSRKGISSEKSFKSEGMKWKLELSSMQNALEFLAQAAGTVAKEGAKEIIKEKSTTPKPLKSSLDATNKSATD
EGLKRLSKSDSTNTLYENTADMLNHTLNTNRKTSQLMEEIGKVRPPPTRKIDDFDYIGPDSLLTKEEAIELIEAFFLTMHPFFPNIPLQLHDPKELAEYPILFCAILTVSARYHPFDTLGLDNGEDGMRHIEVHDKLWVYCQKLISQTIWAEASTRSIGTVLAFIIFTEWNPRSI
HYKWSDYANDPELNNVNARGSKNISTRKDEEGLTGVGAIRRSDRMSWMLTGSAVRLAQDMGFIENSSKVFIVTHISETTSAMNMNQRSLLAESFSVLNLNLGKIENDGNESNEDYLGNEKFYLNEILPDEESKLRWKRVFENSENDHDNEKNFLTDWEREFLNDEYVLYYSNKKD
DTNLAQNHIPPFPLRFSFAQRAKIEIIRILSIAYETIYCEKNKRKLATTDQRHNLSVLSVFSPLIEGWLSNYRELLVPLSDVPFSLADRKNKKQIFDNIDRINGESIITDFNYCQLYIFSLALQVDGKTSRLNMNEIVTSARYVELAYRSAKEILSSAKRVSRQGMLKYMPVRWV
IRIIRSIAFIVKCYLTLTGSELATNPDARNILKLSAISVDETFDIIRDTAVTLKEATPDELHLCQRYAAILMYLCTEMKLRKKSYLERPPLLRDGTTPLESNRESSLEGQDLTKKPIFSKRIGYNKTETTFEPSERPLTEEINSNSQNSNDTSSKGIVDPFVEQNNDITTALLNN
ELFQGPSLSDEVTDWFGASEDIGLEFVEPWTELIEQRYMQCGDGDNNNFENLYNLFVNSNNINNDINNSRPITRK
0000000000000000000000000000000000000000000000000000000000000000000000000000000000000000000000000000000000000000000000000000000000000000000000000000000000000000000000000000000
0000000000000000000000000000000000000000000000000000000000000000000000000000000000000000000000000000000000000000000000000000000000000000000000000000000000000000000000000000000
0000000000000000000000000000000000000000000000000000000000000000000000000000000000000000000000000000000000000000000000000000000000000000000000000000000000000000000000000000000
0000000000000000000000000000000000000000000000000000000000000000000000000000000000000000000000000000000000000000000000000000000000000000000000000000000000000000000000000000000
0000000000000000000000000000000000000000000000000000000000000000000000000000000000000000000000000000000000000000000000000000000000000000000000000000000000000000000000000000000
000000000000000000000000000000000000000000000000000000000000000000000000000
0000000000000000000000000000000000000000000000000000000000000000000000000000000000000000000000000000000000000000000000000000000000000000000000000000000000000000000000000000000
0000000000000000000000000000000000000000000000000000000000000000000000000000000000000000000000000000000000000000000000000000000000000000000000000000000000000000000000000000000
0000000000000000000000000000000000000000000000000000000000000000000000000000000000000000000000000000000000000000000000000000000000000000000000000000000000000000000000000000000
0000000000000000000000000000000000000000000000000000000000000000000000000000000000000000000000000000000000000000000000000000000000000000000000000000000000000000000000000000000
0000000000000000000000000000000000000000000000000000000000000000000000000000000000000000000000000000000000000000000000000000000000000000000000000000000000000000000000000000000
000000000000000000000000000000000000000000000000000000000000000000000000000
0000000000000000000000000000000000000000000000000000000000000000000000000000000000000000000000000000000000000000000000000000000000000000000000000000000000000000000000000000000
0000000000000000000000000000000000000000000000000000000000000000000000000000000000000000000000000000000000000000000000000000000000000000000000000000000000000000000000000000000
0000000000000000000000000000000000000000000000000000000000000000000000000000000000000000000000000000000000000000000000000000000000000000000000000000000000000000000000000000000
0000000000000000000000000000000000000000000000000000000000000000000000000000000000000000000000000000000000000000000000000000000000000000000000000000000000000000000000000000000

0000000000000000000000000000000000000000000000000000000000000000000000000000000000000000000000000000000000000000000000000000000000000000000000000000000000000000000000000000000
000000000000000000000000000000000000000000000000000000000000000000000000000
0000000000000000000000000000000000000000000000000000000000000000000000000000000000000000000000000000000000000000000000000000000000000000000000000000000000000000000000000000000
0000000000000000000000000000000000000000000000000000000000000000000000000000000000000000000000000000000000000000000000000000000000000000000000000000000000000000000000000000000
0000000000000000000000000000000000000000000000000000000000000000000000000000000000000000000000000000000000000000000000000000000000000000000000000000000000000000000000000000000
0000000000000000000000000000000000000000000000000000000000000000000000000000000000000000000000000000000000000000000000000000000000000000000000000000000000000000000000000000000
0000000000000000000000000000000000000000000000000000000000000000000000000000000000000000000000000000000000000000000000000000000000000000000000000000000000000000000000000000000
000000000000000000000000000000000000000000000000000000000000000000000000000
0000000000000000000000000000000000000000000000000000000000000000000000000000000000000000000000000000000000000000000000000000000000000000000000000000000000000000000000000000000
0000000000000000000000000000000000000000000000000000000000000000000000000000000000000000000000000000000000000000000000000000000000000000000000000000000000000000000000000000000
0000000000000000000000000000000000000000000000000000000000000000000000000000000000000000000000000000000000000000000000000000000000000000000000000000000000000000000000000000000
0000000000000000000000000000000000000000000000000000000000000000000000000000000000000000000000000000000000000000000000000000000000000000000000000000000000000000000000000000000
0000000000000000000000000000000000000000000000000000000000000000000000000000000000000000000000000000000000000000000000000000000000000000000000000000000000000000000000000000000
000000000000000000000000000000000000000000000000000000000000000000000000000
0000000000000000000000000000000000000000000000000000000000000000000000000000000000000000000000000000000000000000000000000000000000000000000000000000000000000000000000000000000
0000000000000000000000000000000000000000000000000000000000000000000000000000000000000000000000000000000000000000000000000000000000000000000000000000000000000000000000000000000
0000000000000000000000000000000000000000000000000000000000000000000000000000000000000000000000000000000000000000000000000000000000000000000000000000000000000000000000000000000
0000000000000000000000000000000000000000000000000000000000000000000000000000000000000000000000000000000000000000000000000000000000000000000000000000000000000000000000000000000
0000000000000000000000000000000000000000000000000000000000000000000000000000000000000000000000000000000000000000000000000000000000000000000000000000000000000000000000000000000
000000000000000000000000000000000000000000000000000000000000000000000000000
0000000000000000000000000000000000000000000000000000000000000000000000000000000000000000000000000000000000000000000000000000000000000000000000000000000000000000000000000000000
0000000000000000000000000000000000000000000000000000000000000000000000000000000000000000000000000000000000000000000000000000000000000000000000000000000000000000000000000000000
0000000000000000000000000000000000000000000000000000000000000000000000000000000000000000000000000000000000000000000000000000000000000000000000000000000000000000000000000000000
0000000000000000000000000000000000000000000000000000000000000000000000000000000000000000000000000000000000000000000000000000000000000000000000000000000000000000000000000000000
0000000000000000000000000000000000000000000000000000000000000000000000000000000000000000000000000000000000000000000000000000000000000000000000000000000000000000000000000000000
000000000000000000000000000000000000000000000000000000000000000000000000000
>DNA_Q05854
MGRPRKNVSQEKIQQLKRELELAGNRTDVLLQDKKGRSRSCLLCRRRKQRCDHKLPSCTACLKAGIKCVQPSKYSSSTSNSNTNNNTPTAGTVPPTPHPVIKRELQDSSIGAGAGAATSLNDMTIIKPISTSNSNVDAGDANEFRKTIKSVTTNSNPNLMRQDKDQYTIFLEKKL
KSLETLLDLSPGCNQYNYELSQYKKVSHLFSNNTSDYSRPNSSNMVILPLPSPSNKPLENTNNNGSNVNAATNDTSASTNNINNNNAICQSASLLNDPLETLDFTKCIFAKYNLKKEFLMYDPIFELNEKLSRSFLDTFFTRLQFKYPILDEQEIYTFYDHYLHNKILIPPSSPA
TSSAAPPSNSHSYSEIEFHFLSGRMWLVFSISAYLLMTTGKYKGFPPHRYFSTAIRHITKCGLHLNYVQQIELLTLLVLYIIRTDRDSLILYDIIKDVMGISKKKLHLNQWYPNDPFANKKLRLFWCVYLLERMICVAVGKPYTIKESEINLPLFNNDSFYTKGVHAAAPSTNDH
GVQFINQSLKLRRIESQFVETLQLLKNDSRSVKQSIDQLPLVRKFFEDLEVWRKSYSTLDVKNFENETLKLYYYRSVRLLIQPYLEFFAPEDRLFRECQAAAGQICQLYKIFHQKTLNGHSTPAVHTVFVAGVTLIYCMWLARNFDDQRRKKLGDASKHTRPLISASLFSTMDDL
RACSVCLYVMTERSNFARTFRDTFDQLMNATVGNLIERCGPDSSELIFMASSVAKRTEPKNINDEANKAISSGDTLHDSNSANAANLSNSNDKNISHNGGMPPAVARIFGKGQAEEHAGFVENSQVDLAEQEKFKKKQGVLEKTSVPKSLAHLLTKMDDRSRISNSSMSYTTSSS
SSSSSSSSSSTLSFPSSQEKNLKINVNNDNNGMTISSVNREHNNNHNNNNDNNNNNNNNNNNSNNNNNVNNNDNESNSRSTTNNSCNNGNNSQYVRNNNVTMENDVERPIQDQYIVKKPTNQTEFDWQVFQQQAFLQQQLAQHNLQAYLSSLNTDTMTNRSPSKSSSISTASSHS
DPIPIAMTQSPTPYPQTSNMLPQQHVSRPLPQQQREQPQQHITSPQRFSESNFTNQLNNGMINSNPLQSAIFSNHTSENKQLRDVEESNFSTSPLRADYGNNIISSIPASFTSNSIPVSVKQARNGSSSGDILFSNGAHDMINNISTWTNNSVLDALNSKSILQTIFPQSQEPSS
LSMDKQQQQHQQQNMCSENNVTANNFQQTQNDPSYNRNLFMMSNQEGVQYNLDETEKNGPKTQVEANTSANLHFDNVIPTVTNADIRKKRSNWDNMMTSGPVEDFWTINDDYGFLT
0000000000000000000000000000000000000000000000000000000000000000000000000000000000000000000000000000000000000000000000000000000000000000000000000000000000000000000000000000000
0000000000000000000000000000000000000000000000000000000000000000000000000000000000000000000000000000000000000000000000000000000000000000000000000000000000000000000000000000000
0000000000000000000000000000000000000000000000000000000000000000000000000000000000000000000000000000000000000000000000000000000000000000000000000000000000000000000000000000000
0000000000000000000000000000000000000000000000000000000000000000000000000000000000000000000000000000000000000000000000000000000000000000000000000000000000000000000000000000000
0000000000000000000000000000000000000000000000000000000000000000000000000000000000000000000000000000000000000000000000000000000000000000000000000000000000000000000000000000000
0000000000000000000000000000000000000000000000000000000000000000000000000000000000000000000000000000000000000000000000000000000000000000000000000000000000000000000000000000000
0000000000000000000000000000000000000000000000000000000000000000000000000000000000000000000000000000000000000000000000000000000000000000000000000000000000000000000000000000000
00000000000000000000000000000000000000000000000000000000000000000000000000000000000000000000000000000000000000000000
0000000000000000000000000000000000000000000000000000000000000000000000000000000000000000000000000000000000000000000000000000000000000000000000000000000000000000000000000000000
0000000000000000000000000000000000000000000000000000000000000000000000000000000000000000000000000000000000000000000000000000000000000000000000000000000000000000000000000000000
0000000000000000000000000000000000000000000000000000000000000000000000000000000000000000000000000000000000000000000000000000000000000000000000000000000000000000000000000000000
0000000000000000000000000000000000000000000000000000000000000000000000000000000000000000000000000000000000000000000000000000000000000000000000000000000000000000000000000000000
0000000000000000000000000000000000000000000000000000000000000000000000000000000000000000000000000000000000000000000000000000000000000000000000000000000000000000000000000000000
0000000000000000000000000000000000000000000000000000000000000000000000000000000000000000000000000000000000000000000000000000000000000000000000000000000000000000000000000000000
0000000000000000000000000000000000000000000000000000000000000000000000000000000000000000000000000000000000000000000000000000000000000000000000000000000000000000000000000000000
00000000000000000000000000000000000000000000000000000000000000000000000000000000000000000000000000000000000000000000
0000000000000000000000000000000000000000000000000000000000000000000000000000000000000000000000000000000000000000000000000000000000000000000000000000000000000000000000000000000
0000000000000000000000000000000000000000000000000000000000000000000000000000000000000000000000000000000000000000000000000000000000000000000000000000000000000000000000000000000
0000000000000000000000000000000000000000000000000000000000000000000000000000000000000000000000000000000000000000000000000000000000000000000000000000000000000000000000000000000
0000000000000000000000000000000000000000000000000000000000000000000000000000000000000000000000000000000000000000000000000000000000000000000000000000000000000000000000000000000
0000000000000000000000000000000000000000000000000000000000000000000000000000000000000000000000000000000000000000000000000000000000000000000000000000000000000000000000000000000
0000000000000000000000000000000000000000000000000000000000000000000000000000000000000000000000000000000000000000000000000000000000000000000000000000000000000000000000000000000
0000000000000000000000000000000000000000000000000000000000000000000000000000000000000000000000000000000000000000000000000000000000000000000000000000000000000000000000000000000
00000000000000000000000000000000000000000000000000000000000000000000000000000000000000000000000000000000000000000000
0000000000000000000000000000000000000000000000000000000000000000000000000000000000000000000000000000000000000000000000000000000000000000000000000000000000000000000000000000000
0000000000000000000000000000000000000000000000000000000000000000000000000000000000000000000000000000000000000000000000000000000000000000000000000000000000000000000000000000000
0000000000000000000000000000000000000000000000000000000000000000000000000000000000000000000000000000000000000000000000000000000000000000000000000000000000000000000000000000000
0000000000000000000000000000000000000000000000000000000000000000000000000000000000000000000000000000000000000000000000000000000000000000000000000000000000000000000000000000000
0000000000000000000000000000000000000000000000000000000000000000000000000000000000000000000000000000000000000000000000000000000000000000000000000000000000000000000000000000000
0000000000000000000000000000000000000000000000000000000000000000000000000000000000000000000000000000000000000000000000000000000000000000000000000000000000000000000000000000000
0000000000000000000000000000000000000000000000000000000000000000000000000000000000000000000000000000000000000000000000000000000000000000000000000000000000000000000000000000000
00000000000000000000000000000000000000000000000000000000000000000000000000000000000000000000000000000000000000000000
0000000000000000000000000000000000000000000000000000000000000000000000000000000000000000000000000000000000000000000000000000000000000000000000000000000000000000000000000000000
0000000000000000000000000000000000000000000000000000000000000000000000000000000000000000000000000000000000000000000000000000000000000000000000000000000000000000000000000000000

0000000000000000000000000000000000000000000000000000000000000000000000000000000000000000000000000000000000000000000000000000000000000000000000000000000000000000000000000000000
0000000000000000000000000000000000000000000000000000000000000000000000000000000000000000000000000000000000000000000000000000000000000000000000000000000000000000000000000000000
0000000000000000000000000000000000000000000000000000000000000000000000000000000000000000000000000000000000000000000000000000000000000000000000000000000000000000000000000000000
0000000000000000000000000000000000000000000000000000000000000000000000000000000000000000000000000000000000000000000000000000000000000000000000000000000000000000000000000000000
0000000000000000000000000000000000000000000000000000000000000000000000000000000000000000000000000000000000000000000000000000000000000000000000000000000000000000000000000000000
00000000000000000000000000000000000000000000000000000000000000000000000000000000000000000000000000000000000000000000
0000000000000000000000000000000000000000000000000000000000000000000000000000000000000000000000000000000000000000000000000000000000000000000000000000000000000000000000000000000
0000000000000000000000000000000000000000000000000000000000000000000000000000000000000000000000000000000000000000000000000000000000000000000000000000000000000000000000000000000
0000000000000000000000000000000000000000000000000000000000000000000000000000000000000000000000000000000000000000000000000000000000000000000000000000000000000000000000000000000
0000000000000000000000000000000000000000000000000000000000000000000000000000000000000000000000000000000000000000000000000000000000000000000000000000000000000000000000000000000
0000000000000000000000000000000000000000000000000000000000000000000000000000000000000000000000000000000000000000000000000000000000000000000000000000000000000000000000000000000
0000000000000000000000000000000000000000000000000000000000000000000000000000000000000000000000000000000000000000000000000000000000000000000000000000000000000000000000000000000
0000000000000000000000000000000000000000000000000000000000000000000000000000000000000000000000000000000000000000000000000000000000000000000000000000000000000000000000000000000
00000000000000000000000000000000000000000000000000000000000000000000000000000000000000000000000000000000000000000000
0000000000000000000000000000000000000000000000000000000000000000000000000000000000000000000000000000000000000000000000000000000000000000000000000000000000000000000000000000000
0000000000000000000000000000000000000000000000000000000000000000000000000000000000000000000000000000000000000000000000000000000000000000000000000000000000000000000000000000000
0000000000000000000000000000000000000000000000000000000000000000000000000000000000000000000000000000000000000000000000000000000000000000000000000000000000000000000000000000000
0000000000000000000000000000000000000000000000000000000000000000000000000000000000000000000000000000000000000000000000000000000000000000000000000000000000000000000000000000000
0000000000000000000000000000000000000000000000000000000000000000000000000000000000000000000000000000000000000000000000000000000000000000000000000000000000000000000000000000000
0000000000000000000000000000000000000000000000000000000000000000000000000000000000000000000000000000000000000000000000000000000000000000000000000000000000000000000000000000000
0000000000000000000000000000000000000000000000000000000000000000000000000000000000000000000000000000000000000000000000000000000000000000000000000000000000000000000000000000000
00000000000000000000000000000000000000000000000000000000000000000000000000000000000000000000000000000000000000000000
>DNA_Q06615
MLHSPVFPAFGHHLAQHPAMPLAMELPRTPKASFNIDSILSRTDRPASKLSMEMPSWQPPSPPSMPYRYSYGMMPYPPVWLIKPTVGYPNMAQQQPMRMPRGECLCPDPTCKERVLPFSHCPNGAMNPLSWRTGPCKMKRIRTVFTPEQLEKLEKEFLKQQYMVGTERVDLASTL
NLTETQVKVWFQNRRIKWRKQSLEQKKAKLSQFGVIPADSSDHTDDSRETEEDEDDLDVEL
0000000000000000000000000000000000000000000000000000000000000000000000000000000000000000000000000000000000000000000000000000000000000000000000000000000000000000000000000000000
0000000000000000000000000000000000000000000000000000000000000
0000000000000000000000000000000000000000000000000000000000000000000000000000000000000000000000000000000000000000000000000000000000000000000000000000000000000000000000000000000
0000000000000000000000000000000000000000000000000000000000000
0000000000000000000000000000000000000000000000000000000000000000000000000000000000000000000000000000000000000000000000000000000000000000000000000000000000000000000000000000000
0000000000000000000000000000000000000000000000000000000000000
0000000000000000000000000000000000000000000000000000000000000000000000000000000000000000000000000000000000000000000000000000000000000000000000000000000000000000000000000000000
0000000000000000000000000000000000000000000000000000000000000
0000000000000000000000000000000000000000000000000000000000000000000000000000000000000000000000000000000000000000000000000000000000000000000000000000000000000000000000000000000
0000000000000000000000000000000000000000000000000000000000000
0000000000000000000000000000000000000000000000000000000000000000000000000000000000000000000000000000000000000000000000000000000000000000000000000000000000000000000000000000000
0000000000000000000000000000000000000000000000000000000000000
0000000000000000000000000000000000000000000000000000000000000000000000000000000000000000000000000000000000000000000000000000000000000000000000000000000000000000000000000000000
0000000000000000000000000000000000000000000000000000000000000
>DNA_Q07687
MTGVFDSLVADMHSTQIAASSTYHQHQQPPSGGGAGPGGNSSSSSSLHKPQESPTLPVSTATDSSYYTNQQHPAGGGGGGGSPYAHMGSYQYQASGLNNVPYSAKSSYDLGYTAAYTSYAPYGTSSSPANNEPEKEDLEPEIRIVNGKPKKVRKPRTIYSSFQLAALQRRFQKTQ
YLALPERAELAASSLGLTQTQVKIWFQNRRSKFKKMWKSGEIPSEQHPGASASPPCASPPVSAPASWDFGVPQRMAGGGGPGSGGSGAGSSGSSPSSAASAFLGNYPWYHQTSGSASHLQATAPLLHPTQTPQPHHHHHHHGGGGAPVSAGTIF
0000000000000000000000000000000000000000000000000000000000000000000000000000000000000000000000000000000000000000000000000000000000000000000000000000000000000000000000000000000
0000000000000000000000000000000000000000000000000000000000000000000000000000000000000000000000000000000000000000000000000000000000000000000000000000
0000000000000000000000000000000000000000000000000000000000000000000000000000000000000000000000000000000000000000000000000000000000000000000000000000000000000000000000000000000
0000000000000000000000000000000000000000000000000000000000000000000000000000000000000000000000000000000000000000000000000000000000000000000000000000
0000000000000000000000000000000000000000000000000000000000000000000000000000000000000000000000000000000000000000000000000000000000000000000000000000000000000000000000000000000
0000000000000000000000000000000000000000000000000000000000000000000000000000000000000000000000000000000000000000000000000000000000000000000000000000
0000000000000000000000000000000000000000000000000000000000000000000000000000000000000000000000000000000000000000000000000000000000000000000000000000000000000000000000000000000
0000000000000000000000000000000000000000000000000000000000000000000000000000000000000000000000000000000000000000000000000000000000000000000000000000
0000000000000000000000000000000000000000000000000000000000000000000000000000000000000000000000000000000000000000000000000000000000000000000000000000000000000000000000000000000
0000000000000000000000000000000000000000000000000000000000000000000000000000000000000000000000000000000000000000000000000000000000000000000000000000
0000000000000000000000000000000000000000000000000000000000000000000000000000000000000000000000000000000000000000000000000000000000000000000000000000000000000000000000000000000
0000000000000000000000000000000000000000000000000000000000000000000000000000000000000000000000000000000000000000000000000000000000000000000000000000
0000000000000000000000000000000000000000000000000000000000000000000000000000000000000000000000000000000000000000000000000000000000000000000000000000000000000000000000000000000
0000000000000000000000000000000000000000000000000000000000000000000000000000000000000000000000000000000000000000000000000000000000000000000000000000
>DNA_Q09441
MESRKRKSELEHYIDKLTDPPEKQRKMAEFYNSLRMFYKRRWNATLKLPHVQGVEVNLYRLYDTVMALGGWQKVAASDKWSDIAEMFGCKDDILCGDHAIKIIYMRYLSKFEQVETIGDVDDYVDNEMSRSRGRNATSFFATNECPISNNRMVQEYQHRDERGQIINEPDYARLT
KSLISGLPNEIDFAMNVCMLLSHAGPKQLRICHAPTLLTLLVAHTGVYDEDDETMADMGKEWKRTTKHNFRDFWASSGVPLDMLMTFLDREIEAEYIDEDDQFFTGVSETFNVKDSRCWRLNQVTTIIRNLSFEPANRVTIVKTWPVMKFLIMCASCKWSPLYVAALDALSNLAT
DIDLTDKTLVYISQHAILRIITDGIFSLDKFKLVRSLEILTGLCGFEGNEAIICDWLNSATIAHIFEVVGVKDIMMCVYTLECLYQISEMGDTACDLISESPKAIQQLVSMATLEAVSFGPAGLAGMKVVEYQPSFTQGSNQQQNPHHSQGGHQLGHSNVRMGGLNHNQHHQQIG
PPGAPGAAPMTVRQVIQNNLALQQVQRENLNREQYSTQSSQPHPPHTNVPPSPSILAHHSSGLVQQNGMKFDRRTGNLPVRPIAPSTNSGESQLEQLTEKWIRQNCVFEPAMSTPRGELYAAYVDDLRNLYHSMSGSLAMFSGVMKNLYPDVNFRMAQNGIMIVAQGIRLIRPHR
LAPAASQSASESHPLMKKMLTSEPKEENGVLINGHAVQESGAERIIKTEPIRTDEPSVLIESQEQSVPKKQNDINGEETDEVELNSKIVTPEKKSITNGTVEVCQEPIQASKLFDENYANQIEGSEVSIEIREQFVNDTDNVKLESEKINGIICNGNSKVKKESMASRAAHIVAV
AAACNGDLERMNVVSNGNDIEKEIEKETEKVEEETNEEKNKEVVETEVVDDAQESEKRKCVPPSSNPTDYMCDWDCCSIYYASPSHVLKHLSEEHVAEELRLLCRWNGCADPTPRNRWSLITHIQDSHCNEAQLKAASQKRKEGGGIAPVRGAPRAEVISRDINNHPGYAKNAAF
DAIRRHAFNFLSRELTDEAEGPVTKSIRLTSCLILRNLARYSAEGRQKLRRHESHICWLALSRLESAHALSQLLSELHQAPAAEEEQQKMLSEVPSSASLSSMAGSSSQLPTVPDSPTSSVASAPMKESTSVSNKPTVHINRMLNFSSLNEKTPTSPQFTAGSSPHRHQPIQQHI
PSQPSPLVQTTPVRAGAGI
0000000000000000000000000000000000000000000000000000000000000000000000000000000000000000000000000000000000000000000000000000000000000000000000000000000000000000000000000000000
0000000000000000000000000000000000000000000000000000000000000000000000000000000000000000000000000000000000000000000000000000000000000000000000000000000000000000000000000000000
0000000000000000000000000000000000000000000000000000000000000000000000000000000000000000000000000000000000000000000000000000000000000000000000000000000000000000000000000000000
0000000000000000000000000000000000000000000000000000000000000000000000000000000000000000000000000000000000000000000000000000000000000000000000000000000000000000000000000000000

0000000000000000000000000000000000000000000000000000000000000000000000000000000000000000000000000000000000000000000000000000000000000000000000000000000000000000000000000000000
0000000000000000000000000000000000000000000000000000000000000000000000000000000000000000000000000000000000000000000000000000000000000000000000000000000000000000000000000000000
0000000000000000000000000000000000000000000000000000000000000000000000000000000000000000000000000000000000000000000000000000000000000000000000000000000000000000000000000000000
0000000000000000000
0000000000000000000000000000000000000000000000000000000000000000000000000000000000000000000000000000000000000000000000000000000000000000000000000000000000000000000000000000000
0000000000000000000000000000000000000000000000000000000000000000000000000000000000000000000000000000000000000000000000000000000000000000000000000000000000000000000000000000000
0000000000000000000000000000000000000000000000000000000000000000000000000000000000000000000000000000000000000000000000000000000000000000000000000000000000000000000000000000000
0000000000000000000000000000000000000000000000000000000000000000000000000000000000000000000000000000000000000000000000000000000000000000000000000000000000000000000000000000000
0000000000000000000000000000000000000000000000000000000000000000000000000000000000000000000000000000000000000000000000000000000000000000000000000000000000000000000000000000000
0000000000000000000000000000000000000000000000000000000000000000000000000000000000000000000000000000000000000000000000000000000000000000000000000000000000000000000000000000000
0000000000000000000000000000000000000000000000000000000000000000000000000000000000000000000000000000000000000000000000000000000000000000000000000000000000000000000000000000000
0000000000000000000
0000000000000000000000000000000000000000000000000000000000000000000000000000000000000000000000000000000000000000000000000000000000000000000000000000000000000000000000000000000
0000000000000000000000000000000000000000000000000000000000000000000000000000000000000000000000000000000000000000000000000000000000000000000000000000000000000000000000000000000
0000000000000000000000000000000000000000000000000000000000000000000000000000000000000000000000000000000000000000000000000000000000000000000000000000000000000000000000000000000
0000000000000000000000000000000000000000000000000000000000000000000000000000000000000000000000000000000000000000000000000000000000000000000000000000000000000000000000000000000
0000000000000000000000000000000000000000000000000000000000000000000000000000000000000000000000000000000000000000000000000000000000000000000000000000000000000000000000000000000
0000000000000000000000000000000000000000000000000000000000000000000000000000000000000000000000000000000000000000000000000000000000000000000000000000000000000000000000000000000
0000000000000000000000000000000000000000000000000000000000000000000000000000000000000000000000000000000000000000000000000000000000000000000000000000000000000000000000000000000
0000000000000000000
0000000000000000000000000000000000000000000000000000000000000000000000000000000000000000000000000000000000000000000000000000000000000000000000000000000000000000000000000000000
0000000000000000000000000000000000000000000000000000000000000000000000000000000000000000000000000000000000000000000000000000000000000000000000000000000000000000000000000000000
0000000000000000000000000000000000000000000000000000000000000000000000000000000000000000000000000000000000000000000000000000000000000000000000000000000000000000000000000000000
0000000000000000000000000000000000000000000000000000000000000000000000000000000000000000000000000000000000000000000000000000000000000000000000000000000000000000000000000000000
0000000000000000000000000000000000000000000000000000000000000000000000000000000000000000000000000000000000000000000000000000000000000000000000000000000000000000000000000000000
0000000000000000000000000000000000000000000000000000000000000000000000000000000000000000000000000000000000000000000000000000000000000000000000000000000000000000000000000000000
0000000000000000000000000000000000000000000000000000000000000000000000000000000000000000000000000000000000000000000000000000000000000000000000000000000000000000000000000000000
0000000000000000000
0000000000000000000000000000000000000000000000000000000000000000000000000000000000000000000000000000000000000000000000000000000000000000000000000000000000000000000000000000000
0000000000000000000000000000000000000000000000000000000000000000000000000000000000000000000000000000000000000000000000000000000000000000000000000000000000000000000000000000000
0000000000000000000000000000000000000000000000000000000000000000000000000000000000000000000000000000000000000000000000000000000000000000000000000000000000000000000000000000000
0000000000000000000000000000000000000000000000000000000000000000000000000000000000000000000000000000000000000000000000000000000000000000000000000000000000000000000000000000000
0000000000000000000000000000000000000000000000000000000000000000000000000000000000000000000000000000000000000000000000000000000000000000000000000000000000000000000000000000000
0000000000000000000000000000000000000000000000000000000000000000000000000000000000000000000000000000000000000000000000000000000000000000000000000000000000000000000000000000000
0000000000000000000000000000000000000000000000000000000000000000000000000000000000000000000000000000000000000000000000000000000000000000000000000000000000000000000000000000000
0000000000000000000
0000000000000000000000000000000000000000000000000000000000000000000000000000000000000000000000000000000000000000000000000000000000000000000000000000000000000000000000000000000
0000000000000000000000000000000000000000000000000000000000000000000000000000000000000000000000000000000000000000000000000000000000000000000000000000000000000000000000000000000
0000000000000000000000000000000000000000000000000000000000000000000000000000000000000000000000000000000000000000000000000000000000000000000000000000000000000000000000000000000
0000000000000000000000000000000000000000000000000000000000000000000000000000000000000000000000000000000000000000000000000000000000000000000000000000000000000000000000000000000
0000000000000000000000000000000000000000000000000000000000000000000000000000000000000000000000000000000000000000000000000000000000000000000000000000000000000000000000000000000
0000000000000000000000000000000000000000000000000000000000000000000000000000000000000000000000000000000000000000000000000000000000000000000000000000000000000000000000000000000
0000000000000000000000000000000000000000000000000000000000000000000000000000000000000000000000000000000000000000000000000000000000000000000000000000000000000000000000000000000
0000000000000000000
0000000000000000000000000000000000000000000000000000000000000000000000000000000000000000000000000000000000000000000000000000000000000000000000000000000000000000000000000000000
0000000000000000000000000000000000000000000000000000000000000000000000000000000000000000000000000000000000000000000000000000000000000000000000000000000000000000000000000000000
0000000000000000000000000000000000000000000000000000000000000000000000000000000000000000000000000000000000000000000000000000000000000000000000000000000000000000000000000000000
0000000000000000000000000000000000000000000000000000000000000000000000000000000000000000000000000000000000000000000000000000000000000000000000000000000000000000000000000000000
0000000000000000000000000000000000000000000000000000000000000000000000000000000000000000000000000000000000000000000000000000000000000000000000000000000000000000000000000000000
0000000000000000000000000000000000000000000000000000000000000000000000000000000000000000000000000000000000000000000000000000000000000000000000000000000000000000000000000000000
0000000000000000000000000000000000000000000000000000000000000000000000000000000000000000000000000000000000000000000000000000000000000000000000000000000000000000000000000000000
0000000000000000000
>DNA_Q11178
MLQPPTLTPNANGEEFRGSVSTDRSSASPSNIDEIMEDDVNQKDEDEDLNLVDRVSKLETNFVFIQHQLSSIMNHLHAPQCKCTTCSKVNQPGAEEQKNDTSVLSQLFPNASDQQLKTLLDLSKMNKLPSQVVHKDQQNGSSFLPSANKTSENPKPIELKPLKLGQGYQMANGGG
GGKIHTERLSEPARKQSRKVGFPPPVASYYQRKESSNPLVPPTPLSSIPNLITNSNAVSANTSTASPGPSSEGSGDDHLEAPNEVAHMHSSTVPATPTTPGANLFTQSMVDMLKLNAQNAAHSTGSPGFLRGRGRGRPKLIGDELDADLVDYMVSLKNSDPHGGHFTASQALHMA
RQYILERAPGLLEEHGGHVKLKLTWAMKLVSRIGERQREIELGLPAGTLSNMGRNLTNIPAGGNFMADMMAQNIFSQHMMMVNQQLEGGSPPASSSSTATTSTATKTVKQESKNGHQNEENLNVKQEASTMPEIINIKELNLPFLNNFLAELNDNNSGLIDGEIGAAGPSMAALF
APNA
0000000000000000000000000000000000000000000000000000000000000000000000000000000000000000000000000000000000000000000000000000000000000000000000000000000000000000000000000000000
0000000000000000000000000000000000000000000000000000000000000000000000000000000000000000000000000000000000000000000000000000000000000000000000000000000000000000000000000000000
0000000000000000000000000000000000000000000000000000000000000000000000000000000000000000000000000000000000000000000000000000000000000000000000000000000000000000000000000000000
0000
0000000000000000000000000000000000000000000000000000000000000000000000000000000000000000000000000000000000000000000000000000000000000000000000000000000000000000000000000000000
0000000000000000000000000000000000000000000000000000000000000000000000000000000000000000000000000000000000000000000000000000000000000000000000000000000000000000000000000000000
0000000000000000000000000000000000000000000000000000000000000000000000000000000000000000000000000000000000000000000000000000000000000000000000000000000000000000000000000000000
0000
0000000000000000000000000000000000000000000000000000000000000000000000000000000000000000000000000000000000000000000000000000000000000000000000000000000000000000000000000000000
0000000000000000000000000000000000000000000000000000000000000000000000000000000000000000000000000000000000000000000000000000000000000000000000000000000000000000000000000000000
0000000000000000000000000000000000000000000000000000000000000000000000000000000000000000000000000000000000000000000000000000000000000000000000000000000000000000000000000000000
0000

0000000000000000000000000000000000000000000000000000000000000000000000000000000000000000000000000000000000000000000000000000000000000000000000000000000000000000000000000000000
0000000000000000000000000000000000000000000000000000000000000000000000000000000000000000000000000000000000000000000000000000000000000000000000000000000000000000000000000000000
0000000000000000000000000000000000000000000000000000000000000000000000000000000000000000000000000000000000000000000000000000000000000000000000000000000000000000000000000000000
0000
0000000000000000000000000000000000000000000000000000000000000000000000000000000000000000000000000000000000000000000000000000000000000000000000000000000000000000000000000000000
0000000000000000000000000000000000000000000000000000000000000000000000000000000000000000000000000000000000000000000000000000000000000000000000000000000000000000000000000000000
0000000000000000000000000000000000000000000000000000000000000000000000000000000000000000000000000000000000000000000000000000000000000000000000000000000000000000000000000000000
0000
0000000000000000000000000000000000000000000000000000000000000000000000000000000000000000000000000000000000000000000000000000000000000000000000000000000000000000000000000000000
0000000000000000000000000000000000000000000000000000000000000000000000000000000000000000000000000000000000000000000000000000000000000000000000000000000000000000000000000000000
0000000000000000000000000000000000000000000000000000000000000000000000000000000000000000000000000000000000000000000000000000000000000000000000000000000000000000000000000000000
0000
0000000000000000000000000000000000000000000000000000000000000000000000000000000000000000000000000000000000000000000000000000000000000000000000000000000000000000000000000000000
0000000000000000000000000000000000000000000000000000000000000000000000000000000000000000000000000000000000000000000000000000000000000000000000000000000000000000000000000000000
0000000000000000000000000000000000000000000000000000000000000000000000000000000000000000000000000000000000000000000000000000000000000000000000000000000000000000000000000000000
0000
>DNA_Q14934
MGAASCEDEELEFKLVFGEEKEAPPLGAGGLGEELDSEDAPPCCRLALGEPPPYGAAPIGIPRPPPPRPGMHSPPPRPAPSPGTWESQPARSVRLGGPGGGAGGAGGGRVLECPSIRITSISPTPEPPAALEDNPDAWGDGSPRDYPPPEGFGGYREAGGQGGGAFFSPSPGSSS
LSSWSFFSDASDEAALYAACDEVESELNEAASRFGLGSPLPSPRASPRPWTPEDPWSLYGPSPGGRGPEDSWLLLSAPGPTPASPRPASPCGKRRYSSSGTPSSASPALSRRGSLGEEGSEPPPPPPLPLARDPGSPGPFDYVGAPPAESIPQKTRRTSSEQAVALPRSEEPASC
NGKLPLGAEESVAPPGGSRKEVAGMDYLAVPSPLAWSKARIGGHSPIFRTSALPPLDWPLPSQYEQLELRIEVQPRAHHRAHYETEGSRGAVKAAPGGHPVVKLLGYSEKPLTLQMFIGTADERNLRPHAFYQVHRITGKMVATASYEAVVSGTKVLEMTLLPENNMAANIDCAG
ILKLRNSDIELRKGETDIGRKNTRVRLVFRVHVPQGGGKVVSVQAASVPIECSQRSAQELPQVEAYSPSACSVRGGEELVLTGSNFLPDSKVVFIERGPDGKLQWEEEATVNRLQSNEVTLTLTVPEYSNKRVSRPVQVYFYVSNGRRKRSPTQSFRFLPVICKEEPLPDSSLRG
FPSASATPFGTDMDFSPPRPPYPSYPHEDPACETPYLSEGFGYGMPPLYPQTGPPPSYRPGLRMFPETRGTTGCAQPPAVSFLPRPFPSDPYGGRGSSFSLGLPFSPPAPFRPPPLPASPPLEGPFPSQSDVHPLPAEGYNKVGPGYGPGEGAPEQEKSRGGYSSGFRDSVPIQG
ITLEEVSEIIGRDLSGFPAPPGEEPPA
0000000000000000000000000000000000000000000000000000000000000000000000000000000000000000000000000000000000000000000000000000000000000000000000000000000000000000000000000000000
0000000000000000000000000000000000000000000000000000000000000000000000000000000000000000000000000000000000000000000000000000000000000000000000000000000000000000000000000000000
0000000000000000000000000000000000000000000000000000000000000000000000000000000000000000000000000000000000000000000000000000000000000000000000000000000000000000000000000000000
0000000000000000000000000000000000000000000000000000000000000000000000000000000000000000000000000000000000000000000000000000000000000000000000000000000000000000000000000000000
0000000000000000000000000000000000000000000000000000000000000000000000000000000000000000000000000000000000000000000000000000000000000000000000000000000000000000000000000000000
000000000000000000000000000
0000000000000000000000000000000000000000000000000000000000000000000000000000000000000000000000000000000000000000000000000000000000000000000000000000000000000000000000000000000
0000000000000000000000000000000000000000000000000000000000000000000000000000000000000000000000000000000000000000000000000000000000000000000000000000000000000000000000000000000
0000000000000000000000000000000000000000000000000000000000000000000000000000000000000000000000000000000000000000000000000000000000000000000000000000000000000000000000000000000
0000000000000000000000000000000000000000000000000000000000000000000000000000000000000000000000000000000000000000000000000000000000000000000000000000000000000000000000000000000
0000000000000000000000000000000000000000000000000000000000000000000000000000000000000000000000000000000000000000000000000000000000000000000000000000000000000000000000000000000
000000000000000000000000000
0000000000000000000000000000000000000000000000000000000000000000000000000000000000000000000000000000000000000000000000000000000000000000000000000000000000000000000000000000000
0000000000000000000000000000000000000000000000000000000000000000000000000000000000000000000000000000000000000000000000000000000000000000000000000000000000000000000000000000000
0000000000000000000000000000000000000000000000000000000000000000000000000000000000000000000000000000000000000000000000000000000000000000000000000000000000000000000000000000000
0000000000000000000000000000000000000000000000000000000000000000000000000000000000000000000000000000000000000000000000000000000000000000000000000000000000000000000000000000000
0000000000000000000000000000000000000000000000000000000000000000000000000000000000000000000000000000000000000000000000000000000000000000000000000000000000000000000000000000000
000000000000000000000000000
0000000000000000000000000000000000000000000000000000000000000000000000000000000000000000000000000000000000000000000000000000000000000000000000000000000000000000000000000000000
0000000000000000000000000000000000000000000000000000000000000000000000000000000000000000000000000000000000000000000000000000000000000000000000000000000000000000000000000000000
0000000000000000000000000000000000000000000000000000000000000000000000000000000000000000000000000000000000000000000000000000000000000000000000000000000000000000000000000000000
0000000000000000000000000000000000000000000000000000000000000000000000000000000000000000000000000000000000000000000000000000000000000000000000000000000000000000000000000000000
0000000000000000000000000000000000000000000000000000000000000000000000000000000000000000000000000000000000000000000000000000000000000000000000000000000000000000000000000000000
000000000000000000000000000
0000000000000000000000000000000000000000000000000000000000000000000000000000000000000000000000000000000000000000000000000000000000000000000000000000000000000000000000000000000
0000000000000000000000000000000000000000000000000000000000000000000000000000000000000000000000000000000000000000000000000000000000000000000000000000000000000000000000000000000
0000000000000000000000000000000000000000000000000000000000000000000000000000000000000000000000000000000000000000000000000000000000000000000000000000000000000000000000000000000
0000000000000000000000000000000000000000000000000000000000000000000000000000000000000000000000000000000000000000000000000000000000000000000000000000000000000000000000000000000
0000000000000000000000000000000000000000000000000000000000000000000000000000000000000000000000000000000000000000000000000000000000000000000000000000000000000000000000000000000
000000000000000000000000000
0000000000000000000000000000000000000000000000000000000000000000000000000000000000000000000000000000000000000000000000000000000000000000000000000000000000000000000000000000000
0000000000000000000000000000000000000000000000000000000000000000000000000000000000000000000000000000000000000000000000000000000000000000000000000000000000000000000000000000000
0000000000000000000000000000000000000000000000000000000000000000000000000000000000000000000000000000000000000000000000000000000000000000000000000000000000000000000000000000000
0000000000000000000000000000000000000000000000000000000000000000000000000000000000000000000000000000000000000000000000000000000000000000000000000000000000000000000000000000000
0000000000000000000000000000000000000000000000000000000000000000000000000000000000000000000000000000000000000000000000000000000000000000000000000000000000000000000000000000000
000000000000000000000000000
0000000000000000000000000000000000000000000000000000000000000000000000000000000000000000000000000000000000000000000000000000000000000000000000000000000000000000000000000000000
0000000000000000000000000000000000000000000000000000000000000000000000000000000000000000000000000000000000000000000000000000000000000000000000000000000000000000000000000000000
0000000000000000000000000000000000000000000000000000000000000000000000000000000000000000000000000000000000000000000000000000000000000000000000000000000000000000000000000000000
0000000000000000000000000000000000000000000000000000000000000000000000000000000000000000000000000000000000000000000000000000000000000000000000000000000000000000000000000000000
0000000000000000000000000000000000000000000000000000000000000000000000000000000000000000000000000000000000000000000000000000000000000000000000000000000000000000000000000000000
000000000000000000000000000
>DNA_Q15270
MDGRAELPAFPRAGAPPLAASDTVPAAPEGAGAARPAAPLRPTSFSVLDILDPNKFNSRRRRCVLLGPVAPAACAPCASAPCAPAPAASGRPPRAEELERRALAGAGGVGAAGAEPPNAGDPFKAGEAETNDTNGYSSGGGGHSPSADSGDEVPDDEDDDEDEAPETEAARGAEE
ARGGGGGLGARGSGCQGAAETDASPGATVDEAAAPGPRENSPVAQGPPGGAAAPGGAGTTPQGTATAAKPKRKRTGSDSKSGKPRRARTAFTYEQLVALENKFKATRYLSVCERLNLALSLSLTETQVKIWFQNRRTKWKKQNPGADTSAPTGGGGGPGPGAGPGTGLPGGLSPL
SPSPPMGAPLGMHGPAGYPAHGPGGLVCAAQLPFLSSPAVLSPFVLGSQTYGAPAFYAPHL

0000000000000000000000000000000000000000000000000000000000000000000000000000000000000000000000000000000000000000000000000000000000000000000000000000000000000000000000000000000
0000000000000000000000000000000000000000000000000000000000000000000000000000000000000000000000000000000000000000000000000000000000000000000000000000000000000000000000000000000
0000000000000000000000000000000000000000000000000000000000000
0000000000000000000000000000000000000000000000000000000000000000000000000000000000000000000000000000000000000000000000000000000000000000000000000000000000000000000000000000000
0000000000000000000000000000000000000000000000000000000000000000000000000000000000000000000000000000000000000000000000000000000000000000000000000000000000000000000000000000000
0000000000000000000000000000000000000000000000000000000000000
0000000000000000000000000000000000000000000000000000000000000000000000000000000000000000000000000000000000000000000000000000000000000000000000000000000000000000000000000000000
0000000000000000000000000000000000000000000000000000000000000000000000000000000000000000000000000000000000000000000000000000000000000000000000000000000000000000000000000000000
0000000000000000000000000000000000000000000000000000000000000
0000000000000000000000000000000000000000000000000000000000000000000000000000000000000000000000000000000000000000000000000000000000000000000000000000000000000000000000000000000
0000000000000000000000000000000000000000000000000000000000000000000000000000000000000000000000000000000000000000000000000000000000000000000000000000000000000000000000000000000
0000000000000000000000000000000000000000000000000000000000000
0000000000000000000000000000000000000000000000000000000000000000000000000000000000000000000000000000000000000000000000000000000000000000000000000000000000000000000000000000000
0000000000000000000000000000000000000000000000000000000000000000000000000000000000000000000000000000000000000000000000000000000000000000000000000000000000000000000000000000000
0000000000000000000000000000000000000000000000000000000000000
0000000000000000000000000000000000000000000000000000000000000000000000000000000000000000000000000000000000000000000000000000000000000000000000000000000000000000000000000000000
0000000000000000000000000000000000000000000000000000000000000000000000000000000000000000000000000000000000000000000000000000000000000000000000000000000000000000000000000000000
0000000000000000000000000000000000000000000000000000000000000
0000000000000000000000000000000000000000000000000000000000000000000000000000000000000000000000000000000000000000000000000000000000000000000000000000000000000000000000000000000
0000000000000000000000000000000000000000000000000000000000000000000000000000000000000000000000000000000000000000000000000000000000000000000000000000000000000000000000000000000
0000000000000000000000000000000000000000000000000000000000000
>DNA_Q1ECI2
MSPQRMKLSSPPVTNNEPTATASAVKSCGGGGKETSSSTTRHPVYHGVRKRRWGKWVSEIREPRKKSRIWLGSFPVPEMAAKAYDVAAFCLKGRKAQLNFPEEIEDLPRPSTCTPRDIQVAAAKAANAVKIIKMGDDDVAGIDDGDDFWEGIELPELMMSGGGWSPEPFVAGDDA
TWLVDGDLYQYQFMACL
0000000000000000000000000000000000000000000000000000000000000000000000000000000000000000000000000000000000000000000000000000000000000000000000000000000000000000000000000000000
00000000000000000
0000000000000000000000000000000000000000000000000000000000000000000000000000000000000000000000000000000000000000000000000000000000000000000000000000000000000000000000000000000
00000000000000000
0000000000000000000000000000000000000000000000000000000000000000000000000000000000000000000000000000000000000000000000000000000000000000000000000000000000000000000000000000000
00000000000000000
0000000000000000000000000000000000000000000000000000000000000000000000000000000000000000000000000000000000000000000000000000000000000000000000000000000000000000000000000000000
00000000000000000
0000000000000000000000000000000000000000000000000000000000000000000000000000000000000000000000000000000000000000000000000000000000000000000000000000000000000000000000000000000
00000000000000000
0000000000000000000000000000000000000000000000000000000000000000000000000000000000000000000000000000000000000000000000000000000000000000000000000000000000000000000000000000000
00000000000000000
0000000000000000000000000000000000000000000000000000000000000000000000000000000000000000000000000000000000000000000000000000000000000000000000000000000000000000000000000000000
00000000000000000
>DNA_Q2NF79
MDSSTITRKNILDDVTSLLTSFGFKTSNIYDRNCFDLLARKKDILIILKILVNIDSLTPTQAEELSKISGTFLASPIIIGYKSKHNYLEEDIVYERHEIPVISPQTLCNIIVNDIHPEIFAKRGGYYVKINGNLLKNLREKNNLSLKELADISHVSRETIYKYEKGNSQTYPETA
MMLEQVLNSPITESINLLETNNTKTTTLDKKIQEPIELIKLGYDIKSSNKAPFDAISERQEETEEVRQLKEKLESKLDEIEKIRAEINKKTKHHNLLITNMERNRSDNTLNKIANCTEDISLITGHDALFVLEHKKDKEFIKKIPAIYTWELKNMDNMEELLKLIKERKEEANDS
0000000000000000000000000000000000000000000000000000000000000000000000000000000000000000000000000000000000000000000000000000000000000000000000000000000000000000000000000000000
0000000000000000000000000000000000000000000000000000000000000000000000000000000000000000000000000000000000000000000000000000000000000000000000000000000000000000000000000000000
0000000000000000000000000000000000000000000000000000000000000000000000000000000000000000000000000000000000000000000000000000000000000000000000000000000000000000000000000000000
0000000000000000000000000000000000000000000000000000000000000000000000000000000000000000000000000000000000000000000000000000000000000000000000000000000000000000000000000000000
0000000000000000000000000000000000000000000000000000000000000000000000000000000000000000000000000000000000000000000000000000000000000000000000000000000000000000000000000000000
0000000000000000000000000000000000000000000000000000000000000000000000000000000000000000000000000000000000000000000000000000000000000000000000000000000000000000000000000000000
0000000000000000000000000000000000000000000000000000000000000000000000000000000000000000000000000000000000000000000000000000000000000000000000000000000000000000000000000000000
0000000000000000000000000000000000000000000000000000000000000000000000000000000000000000000000000000000000000000000000000000000000000000000000000000000000000000000000000000000
0000000000000000000000000000000000000000000000000000000000000000000000000000000000000000000000000000000000000000000000000000000000000000000000000000000000000000000000000000000
0000000000000000000000000000000000000000000000000000000000000000000000000000000000000000000000000000000000000000000000000000000000000000000000000000000000000000000000000000000
0000000000000000000000000000000000000000000000000000000000000000000000000000000000000000000000000000000000000000000000000000000000000000000000000000000000000000000000000000000
0000000000000000000000000000000000000000000000000000000000000000000000000000000000000000000000000000000000000000000000000000000000000000000000000000000000000000000000000000000
0000000000000000000000000000000000000000000000000000000000000000000000000000000000000000000000000000000000000000000000000000000000000000000000000000000000000000000000000000000
0000000000000000000000000000000000000000000000000000000000000000000000000000000000000000000000000000000000000000000000000000000000000000000000000000000000000000000000000000000
>DNA_Q50EX6
MGRQPCCDKLGVKKGPWTAEEDKKLISFILTNGQCCWRAVPKLAGLKRCGKSCRLRWTNYLRPDLKRGLLSDAEEKLVIDLHSRLGNRWSKIAARLPGRTDNEIKNHWNTHIKKKLLKMGIDPVTHEPLKKEANLSDQPTTESDQNKENGHQQVQVVPQSTNVTAAAATSTEFDN
NSSFSSSASSSENSSCTTDESKLVFDNLSENDPLLSCLLEADTPLIDSPWEFPMSSTTTVEEPKSFDSIISNMTSWEDTFNWLSGYQEFGINDFGFDNCFNHVELDIFKTIDNVENRHG
0000000000000000000000000000000000000000000000000000000000000000000000000000000000000000000000000000000000000000000000000000000000000000000000000000000000000000000000000000000
00000000000000000000000000000000000000000000000000000000000000000000000000000000000000000000000000000000000000000000000
0000000000000000000000000000000000000000000000000000000000000000000000000000000000000000000000000000000000000000000000000000000000000000000000000000000000000000000000000000000
00000000000000000000000000000000000000000000000000000000000000000000000000000000000000000000000000000000000000000000000
0000000000000000000000000000000000000000000000000000000000000000000000000000000000000000000000000000000000000000000000000000000000000000000000000000000000000000000000000000000
00000000000000000000000000000000000000000000000000000000000000000000000000000000000000000000000000000000000000000000000
0000000000000000000000000000000000000000000000000000000000000000000000000000000000000000000000000000000000000000000000000000000000000000000000000000000000000000000000000000000
00000000000000000000000000000000000000000000000000000000000000000000000000000000000000000000000000000000000000000000000
0000000000000000000000000000000000000000000000000000000000000000000000000000000000000000000000000000000000000000000000000000000000000000000000000000000000000000000000000000000
00000000000000000000000000000000000000000000000000000000000000000000000000000000000000000000000000000000000000000000000

0000000000000000000000000000000000000000000000000000000000000000000000000000000000000000000000000000000000000000000000000000000000000000000000000000000000000000000000000000000
00000000000000000000000000000000000000000000000000000000000000000000000000000000000000000000000000000000000000000000000
0000000000000000000000000000000000000000000000000000000000000000000000000000000000000000000000000000000000000000000000000000000000000000000000000000000000000000000000000000000
00000000000000000000000000000000000000000000000000000000000000000000000000000000000000000000000000000000000000000000000
>DNA_Q53N87
MVSILQLQTRTEASPASSASAAATRIFAVRRQQQEQEGEEEEEEEFEFQERMDLSGAQGELPIPMHASAAASPFAGMGAHGGAGGGHVVELHRHEHVGNNGQAMAMASPPPTNVAVAAEQEGSPVAGKKRGGMAVVGGGGGVAVKYRECLKNHAAAIGGNATDGCGEFMPSGEEGS
LEALKCSACGCHRNFHRKEADDLDADSCAAALRAAAGRHHHLLGPALPHHHHKNGGGLLVAGGDPYGAAYAAARALPPPPPPPPHGHHHHHQIIMPLNMIHTSESDEMDVSGGGGGVGRGGGSSSSSKKRFRTKFTAEQKARMLEFAERVGWRLQKLDDAMVHHFCQEIGVKRRV
LKVWMHNNKHNLAKKPLPSSPPPPPQIPPMSMPPSPPPPQIPPMSMPPSPPPMPMPMPPSPPQLKLE
0000000000000000000000000000000000000000000000000000000000000000000000000000000000000000000000000000000000000000000000000000000000000000000000000000000000000000000000000000000
0000000000000000000000000000000000000000000000000000000000000000000000000000000000000000000000000000000000000000000000000000000000000000000000000000000000000000000000000000000
0000000000000000000000000000000000000000000000000000000000000000000
0000000000000000000000000000000000000000000000000000000000000000000000000000000000000000000000000000000000000000000000000000000000000000000000000000000000000000000000000000000
0000000000000000000000000000000000000000000000000000000000000000000000000000000000000000000000000000000000000000000000000000000000000000000000000000000000000000000000000000000
0000000000000000000000000000000000000000000000000000000000000000000
0000000000000000000000000000000000000000000000000000000000000000000000000000000000000000000000000000000000000000000000000000000000000000000000000000000000000000000000000000000
0000000000000000000000000000000000000000000000000000000000000000000000000000000000000000000000000000000000000000000000000000000000000000000000000000000000000000000000000000000
0000000000000000000000000000000000000000000000000000000000000000000
0000000000000000000000000000000000000000000000000000000000000000000000000000000000000000000000000000000000000000000000000000000000000000000000000000000000000000000000000000000
0000000000000000000000000000000000000000000000000000000000000000000000000000000000000000000000000000000000000000000000000000000000000000000000000000000000000000000000000000000
0000000000000000000000000000000000000000000000000000000000000000000
0000000000000000000000000000000000000000000000000000000000000000000000000000000000000000000000000000000000000000000000000000000000000000000000000000000000000000000000000000000
0000000000000000000000000000000000000000000000000000000000000000000000000000000000000000000000000000000000000000000000000000000000000000000000000000000000000000000000000000000
0000000000000000000000000000000000000000000000000000000000000000000
0000000000000000000000000000000000000000000000000000000000000000000000000000000000000000000000000000000000000000000000000000000000000000000000000000000000000000000000000000000
0000000000000000000000000000000000000000000000000000000000000000000000000000000000000000000000000000000000000000000000000000000000000000000000000000000000000000000000000000000
0000000000000000000000000000000000000000000000000000000000000000000
0000000000000000000000000000000000000000000000000000000000000000000000000000000000000000000000000000000000000000000000000000000000000000000000000000000000000000000000000000000
0000000000000000000000000000000000000000000000000000000000000000000000000000000000000000000000000000000000000000000000000000000000000000000000000000000000000000000000000000000
0000000000000000000000000000000000000000000000000000000000000000000
>DNA_Q55C24
MSSSDESGSSSSESEKKVKKTPVKKPKKEPKVSKKGKKEGKKTKKKPKDENAPRRYLSPFIFFSKDHRSVIKNSHPNCSFGEIGSLLGQEWAKISAEDKKKYEKLAAEDKKRWELEKKNYDEKLKTQSQAESSSDSSDESD
000000000000000000000000000000000000000000000000000000000000000000000000000000000000000000000000000000000000000000000000000000000000000000000
000000000000000000000000000000000000000000000000000000000000000000000000000000000000000000000000000000000000000000000000000000000000000000000
000000000000000000000000000000000000000000000000000000000000000000000000000000000000000000000000000000000000000000000000000000000000000000000
000000000000000000000000000000000000000000000000000000000000000000000000000000000000000000000000000000000000000000000000000000000000000000000
000000000000000000000000000000000000000000000000000000000000000000000000000000000000000000000000000000000000000000000000000000000000000000000
000000000000000000000000000000000000000000000000000000000000000000000000000000000000000000000000000000000000000000000000000000000000000000000
000000000000000000000000000000000000000000000000000000000000000000000000000000000000000000000000000000000000000000000000000000000000000000000
>DNA_Q5PP28
METPVGLRFCPTDEEIVVDYLWPKNSDRDTSHVDRFINTVPVCRLDPWELPCQSRIKLKDVAWCFFRPKENKYGRGDQQMRKTKSGFWKSTGRPKPIMRNRQQIGEKKILMFYTSKESKSDWVIHEYHGFSHNQMMMTYTLCKVMFNGGMREKSSSSPSSSGVSGIEQSRRDSLI
PQLVNNSEGSSLHREDPSQFGDVLQEAPIEDAKLTEELVKWLMNDEDDAQIEDAIPIEEWETWLNDIDDAKEKSIMFMHDNRSDYRPPNSLTGVFSDDVSSDDNDSDLLTPKTNSIQTSSTCDSFGSSNHRIDQIKDLQESPTSTINLVSLTQEVSQALITSIDTAEKKKNPYDD
AQGTEIGEHKLGQETIKKKRAGFFHRMIQKFVKKIHLCSSISRT
0000000000000000000000000000000000000000000000000000000000000000000000000000000000000000000000000000000000000000000000000000000000000000000000000000000000000000000000000000000
0000000000000000000000000000000000000000000000000000000000000000000000000000000000000000000000000000000000000000000000000000000000000000000000000000000000000000000000000000000
00000000000000000000000000000000000000000000
0000000000000000000000000000000000000000000000000000000000000000000000000000000000000000000000000000000000000000000000000000000000000000000000000000000000000000000000000000000
0000000000000000000000000000000000000000000000000000000000000000000000000000000000000000000000000000000000000000000000000000000000000000000000000000000000000000000000000000000
00000000000000000000000000000000000000000000
0000000000000000000000000000000000000000000000000000000000000000000000000000000000000000000000000000000000000000000000000000000000000000000000000000000000000000000000000000000
0000000000000000000000000000000000000000000000000000000000000000000000000000000000000000000000000000000000000000000000000000000000000000000000000000000000000000000000000000000
00000000000000000000000000000000000000000000
0000000000000000000000000000000000000000000000000000000000000000000000000000000000000000000000000000000000000000000000000000000000000000000000000000000000000000000000000000000
0000000000000000000000000000000000000000000000000000000000000000000000000000000000000000000000000000000000000000000000000000000000000000000000000000000000000000000000000000000
00000000000000000000000000000000000000000000
0000000000000000000000000000000000000000000000000000000000000000000000000000000000000000000000000000000000000000000000000000000000000000000000000000000000000000000000000000000
0000000000000000000000000000000000000000000000000000000000000000000000000000000000000000000000000000000000000000000000000000000000000000000000000000000000000000000000000000000
00000000000000000000000000000000000000000000
0000000000000000000000000000000000000000000000000000000000000000000000000000000000000000000000000000000000000000000000000000000000000000000000000000000000000000000000000000000
0000000000000000000000000000000000000000000000000000000000000000000000000000000000000000000000000000000000000000000000000000000000000000000000000000000000000000000000000000000
00000000000000000000000000000000000000000000
0000000000000000000000000000000000000000000000000000000000000000000000000000000000000000000000000000000000000000000000000000000000000000000000000000000000000000000000000000000
0000000000000000000000000000000000000000000000000000000000000000000000000000000000000000000000000000000000000000000000000000000000000000000000000000000000000000000000000000000
00000000000000000000000000000000000000000000
>DNA_Q623D4
MVSKAEKPKQSGMFSVESLLETTTPQKSSVKDENLLKPPAPITPKTPLMLPGLHHMAPYFGAQLDPVMIYFSQTGNRLPVMSSDSSPESSASSPLSMQHHTLPWLSSQREDSPTSDDAKIQIGLTKCMLRKHKNNRKPRTPFSTQQLISLERKFQSKQYLSIAERAEFSASLQLT
ETQVKIWFQNRRAKSKRLQEAEVEKVKFAQVSAYAAAVNDPSLMAFYPGQW
0000000000000000000000000000000000000000000000000000000000000000000000000000000000000000000000000000000000000000000000000000000000000000000000000000000000000000000000000000000
000000000000000000000000000000000000000000000000000

0000000000000000000000000000000000000000000000000000000000000000000000000000000000000000000000000000000000000000000000000000000000000000000000000000000000000000000000000000000
000000000000000000000000000000000000000000000000000
0000000000000000000000000000000000000000000000000000000000000000000000000000000000000000000000000000000000000000000000000000000000000000000000000000000000000000000000000000000
000000000000000000000000000000000000000000000000000
0000000000000000000000000000000000000000000000000000000000000000000000000000000000000000000000000000000000000000000000000000000000000000000000000000000000000000000000000000000
000000000000000000000000000000000000000000000000000
0000000000000000000000000000000000000000000000000000000000000000000000000000000000000000000000000000000000000000000000000000000000000000000000000000000000000000000000000000000
000000000000000000000000000000000000000000000000000
0000000000000000000000000000000000000000000000000000000000000000000000000000000000000000000000000000000000000000000000000000000000000000000000000000000000000000000000000000000
000000000000000000000000000000000000000000000000000
0000000000000000000000000000000000000000000000000000000000000000000000000000000000000000000000000000000000000000000000000000000000000000000000000000000000000000000000000000000
000000000000000000000000000000000000000000000000000
>DNA_Q63943
MGRKKIQIQRITDERNRQVTFTKRKFGLMKKAYELSVLCDCEIALIIFNHSNKLFQYASTDMDKVLLKYTEYNEPHESRTNADIIETLRKKGFNGCDSPEPDGEDSLEQSPLLEDKYRRASEELDGLFRRYGSSVPAPNFAMPVTVPVSNQSSMQFSNPSSSLVTPSLVTSSLTD
PRLLSPQQPALQRNSVSPGLPQRPASAGAMLGGDLNSANGACPSPVGNGYVSARASPGLLPVANGNSLNKVIPAKSPPPPTHNTQLGAPSRKPDLRVITSQGGKGLMHHLTEDHLDLNNAQRLGVSQSTHSLTTPVVSVATPSLLSQGLPFSSMPTAYNTDYQLPSAELSSLPAF
SSPAGLALGNVTAWQQPQPPQQPQPPQPPQSQPQPPQPQPQQPPQQQPHLVPVSLSNLIPGSPLPHVGAALTVTTHPHISIKSEPVSPSRERSPAPPPPAVFPAARPEPGEGLSSPAGGSYETGDRDDGRGDFGPTLGLLRPAPEPEAEGSAVKRMRLDTWTLK
0000000000000000000000000000000000000000000000000000000000000000000000000000000000000000000000000000000000000000000000000000000000000000000000000000000000000000000000000000000
0000000000000000000000000000000000000000000000000000000000000000000000000000000000000000000000000000000000000000000000000000000000000000000000000000000000000000000000000000000
00000000000000000000000000000000000000000000000000000000000000000000000000000000000000000000000000000000000000000000000000000000000000000000000000000000000000000000
0000000000000000000000000000000000000000000000000000000000000000000000000000000000000000000000000000000000000000000000000000000000000000000000000000000000000000000000000000000
0000000000000000000000000000000000000000000000000000000000000000000000000000000000000000000000000000000000000000000000000000000000000000000000000000000000000000000000000000000
00000000000000000000000000000000000000000000000000000000000000000000000000000000000000000000000000000000000000000000000000000000000000000000000000000000000000000000
0000000000000000000000000000000000000000000000000000000000000000000000000000000000000000000000000000000000000000000000000000000000000000000000000000000000000000000000000000000
0000000000000000000000000000000000000000000000000000000000000000000000000000000000000000000000000000000000000000000000000000000000000000000000000000000000000000000000000000000
00000000000000000000000000000000000000000000000000000000000000000000000000000000000000000000000000000000000000000000000000000000000000000000000000000000000000000000
0000000000000000000000000000000000000000000000000000000000000000000000000000000000000000000000000000000000000000000000000000000000000000000000000000000000000000000000000000000
0000000000000000000000000000000000000000000000000000000000000000000000000000000000000000000000000000000000000000000000000000000000000000000000000000000000000000000000000000000
00000000000000000000000000000000000000000000000000000000000000000000000000000000000000000000000000000000000000000000000000000000000000000000000000000000000000000000
0000000000000000000000000000000000000000000000000000000000000000000000000000000000000000000000000000000000000000000000000000000000000000000000000000000000000000000000000000000
0000000000000000000000000000000000000000000000000000000000000000000000000000000000000000000000000000000000000000000000000000000000000000000000000000000000000000000000000000000
00000000000000000000000000000000000000000000000000000000000000000000000000000000000000000000000000000000000000000000000000000000000000000000000000000000000000000000
0000000000000000000000000000000000000000000000000000000000000000000000000000000000000000000000000000000000000000000000000000000000000000000000000000000000000000000000000000000
0000000000000000000000000000000000000000000000000000000000000000000000000000000000000000000000000000000000000000000000000000000000000000000000000000000000000000000000000000000
00000000000000000000000000000000000000000000000000000000000000000000000000000000000000000000000000000000000000000000000000000000000000000000000000000000000000000000
0000000000000000000000000000000000000000000000000000000000000000000000000000000000000000000000000000000000000000000000000000000000000000000000000000000000000000000000000000000
0000000000000000000000000000000000000000000000000000000000000000000000000000000000000000000000000000000000000000000000000000000000000000000000000000000000000000000000000000000
00000000000000000000000000000000000000000000000000000000000000000000000000000000000000000000000000000000000000000000000000000000000000000000000000000000000000000000
>DNA_Q6X7K0
MWTMGYNEGGADSFNGGRKLRPLIPRLTSCPTAAVNTNSDHRFNMAVVTMTAEQNKRELMMLNSEPQHPPVMVSSRWNPTPDQLRVLEELYRQGTRTPSADHIQQITAQLRRYGKIEGKNVFYWFQNHKARERQKRRRQMETGHEETVLSTASLVSNHGFDKKDPPGYKVEQVKN
WICSVGCDTQPEKPSRDYHLEEPANIRVEHNARCGGDERRSFLGINTTWQMMQLPPSFYSSSHHHHQRNLILNSPTVSSNMSNSNNAVSASKDTVTVSPVFLRTREATNTETCHRNGDDNKDQEQHEDCSNGELDHQEQTLELFPLRKEGFCSDGEKDKNISGIHCFYEFLPLKN
0000000000000000000000000000000000000000000000000000000000000000000000000000000000000000000000000000000000000000000000000000000000000000000000000000000000000000000000000000000
0000000000000000000000000000000000000000000000000000000000000000000000000000000000000000000000000000000000000000000000000000000000000000000000000000000000000000000000000000000
0000000000000000000000000000000000000000000000000000000000000000000000000000000000000000000000000000000000000000000000000000000000000000000000000000000000000000000000000000000
0000000000000000000000000000000000000000000000000000000000000000000000000000000000000000000000000000000000000000000000000000000000000000000000000000000000000000000000000000000
0000000000000000000000000000000000000000000000000000000000000000000000000000000000000000000000000000000000000000000000000000000000000000000000000000000000000000000000000000000
0000000000000000000000000000000000000000000000000000000000000000000000000000000000000000000000000000000000000000000000000000000000000000000000000000000000000000000000000000000
0000000000000000000000000000000000000000000000000000000000000000000000000000000000000000000000000000000000000000000000000000000000000000000000000000000000000000000000000000000
0000000000000000000000000000000000000000000000000000000000000000000000000000000000000000000000000000000000000000000000000000000000000000000000000000000000000000000000000000000
0000000000000000000000000000000000000000000000000000000000000000000000000000000000000000000000000000000000000000000000000000000000000000000000000000000000000000000000000000000
0000000000000000000000000000000000000000000000000000000000000000000000000000000000000000000000000000000000000000000000000000000000000000000000000000000000000000000000000000000
0000000000000000000000000000000000000000000000000000000000000000000000000000000000000000000000000000000000000000000000000000000000000000000000000000000000000000000000000000000
0000000000000000000000000000000000000000000000000000000000000000000000000000000000000000000000000000000000000000000000000000000000000000000000000000000000000000000000000000000
0000000000000000000000000000000000000000000000000000000000000000000000000000000000000000000000000000000000000000000000000000000000000000000000000000000000000000000000000000000
0000000000000000000000000000000000000000000000000000000000000000000000000000000000000000000000000000000000000000000000000000000000000000000000000000000000000000000000000000000
>DNA_Q7T103
MYSQQPFPAFAFNAGLMQDPANCHFGGYTGLGHPQPFSFAFSTLKSENGESGVQGMGDCTTPVMPWNSLASFDHQVQMENNQQGNPPRAPSPTLSDSRIKVKEEVVHETDSGEESPEPKYPSPPNPSLYYPNAWTGAPFWQVNPTPGNNINPMPNQTLVKNTSLPGNTTYPTPAN
QSPNTPVDCVTSSMESSRCSSTNSPNGAINERATTIPNGEMLDGGQSSDNEEEVPSESEMEQFAKDLKHKRVSLGYTQADVGYALGVLYGKMFSQTTICRFESLQLSFKNMCQLKPFLERWVVEAENNDNLQELINREQVIAQTRKRKRRTNIENIVKGTLESYFMKCPKPGAQE
MVQIAKELNMDKDVVRVWFCNRRQKGKRQGMPTVEENDGEGYDVAQTMGSPPVGHYALQQVVTPQGYMAAPQIYASAFHKNDLFPQTVPHGMAMGGHIG
0000000000000000000000000000000000000000000000000000000000000000000000000000000000000000000000000000000000000000000000000000000000000000000000000000000000000000000000000000000
0000000000000000000000000000000000000000000000000000000000000000000000000000000000000000000000000000000000000000000000000000000000000000000000000000000000000000000000000000000
000000000000000000000000000000000000000000000000000000000000000000000000000000000000000000000000000
0000000000000000000000000000000000000000000000000000000000000000000000000000000000000000000000000000000000000000000000000000000000000000000000000000000000000000000000000000000
0000000000000000000000000000000000000000000000000000000000000000000000000000000000000000000000000000000000000000000000000000000000000000000000000000000000000000000000000000000
000000000000000000000000000000000000000000000000000000000000000000000000000000000000000000000000000
0000000000000000000000000000000000000000000000000000000000000000000000000000000000000000000000000000000000000000000000000000000000000000000000000000000000000000000000000000000
0000000000000000000000000000000000000000000000000000000000000000000000000000000000000000000000000000000000000000000000000000000000000000000000000000000000000000000000000000000
000000000000000000000000000000000000000000000000000000000000000000000000000000000000000000000000000

0000000000000000000000000000000000000000000000000000000000000000000000000000000000000000000000000000000000000000000000000000000000000000000000000000000000000000000000000000000
0000000000000000000000000000000000000000000000000000000000000000000000000000000000000000000000000000000000000000000000000000000000000000000000000000000000000000000000000000000
000000000000000000000000000000000000000000000000000000000000000000000000000000000000000000000000000
0000000000000000000000000000000000000000000000000000000000000000000000000000000000000000000000000000000000000000000000000000000000000000000000000000000000000000000000000000000
0000000000000000000000000000000000000000000000000000000000000000000000000000000000000000000000000000000000000000000000000000000000000000000000000000000000000000000000000000000
000000000000000000000000000000000000000000000000000000000000000000000000000000000000000000000000000
0000000000000000000000000000000000000000000000000000000000000000000000000000000000000000000000000000000000000000000000000000000000000000000000000000000000000000000000000000000
0000000000000000000000000000000000000000000000000000000000000000000000000000000000000000000000000000000000000000000000000000000000000000000000000000000000000000000000000000000
000000000000000000000000000000000000000000000000000000000000000000000000000000000000000000000000000
0000000000000000000000000000000000000000000000000000000000000000000000000000000000000000000000000000000000000000000000000000000000000000000000000000000000000000000000000000000
0000000000000000000000000000000000000000000000000000000000000000000000000000000000000000000000000000000000000000000000000000000000000000000000000000000000000000000000000000000
000000000000000000000000000000000000000000000000000000000000000000000000000000000000000000000000000
>DNA_Q7XQN1
MGRVPCCEKDNVKRGQWTPEEDNKLLSYITQYGTRNWRLIPKNAGLQRCGKSCRLRWTNYLRPDLKHGEFTDAEEQTIIKLHSVVGNRWSVIAAQLPGRTDNDVKNHWNTKLKKKLSGMGIDPVTHKSFSHLMAEIATTLAPPQVAHLAEAALGCFKDEMLHLLTKKRPSDFPSP
AVHDGAGAGASASALAAPCFPAAPPHHPQADDTIERIKLGLSRAIMSDPSTASAAAAAAAPSAPAEDKPWPPGDMSEGLAGMYATYNPAAHAHAQAQAEFRYDGASAAQGYVLGGDGDQGTSMWSHQSLYSGSSGTEEARRELPEKGNDSVGSSGGDDDAADDGKDSGKGAASDM
SGLFASDCVLWDLPDELTNHMV
0000000000000000000000000000000000000000000000000000000000000000000000000000000000000000000000000000000000000000000000000000000000000000000000000000000000000000000000000000000
0000000000000000000000000000000000000000000000000000000000000000000000000000000000000000000000000000000000000000000000000000000000000000000000000000000000000000000000000000000
0000000000000000000000
0000000000000000000000000000000000000000000000000000000000000000000000000000000000000000000000000000000000000000000000000000000000000000000000000000000000000000000000000000000
0000000000000000000000000000000000000000000000000000000000000000000000000000000000000000000000000000000000000000000000000000000000000000000000000000000000000000000000000000000
0000000000000000000000
0000000000000000000000000000000000000000000000000000000000000000000000000000000000000000000000000000000000000000000000000000000000000000000000000000000000000000000000000000000
0000000000000000000000000000000000000000000000000000000000000000000000000000000000000000000000000000000000000000000000000000000000000000000000000000000000000000000000000000000
0000000000000000000000
0000000000000000000000000000000000000000000000000000000000000000000000000000000000000000000000000000000000000000000000000000000000000000000000000000000000000000000000000000000
0000000000000000000000000000000000000000000000000000000000000000000000000000000000000000000000000000000000000000000000000000000000000000000000000000000000000000000000000000000
0000000000000000000000
0000000000000000000000000000000000000000000000000000000000000000000000000000000000000000000000000000000000000000000000000000000000000000000000000000000000000000000000000000000
0000000000000000000000000000000000000000000000000000000000000000000000000000000000000000000000000000000000000000000000000000000000000000000000000000000000000000000000000000000
0000000000000000000000
0000000000000000000000000000000000000000000000000000000000000000000000000000000000000000000000000000000000000000000000000000000000000000000000000000000000000000000000000000000
0000000000000000000000000000000000000000000000000000000000000000000000000000000000000000000000000000000000000000000000000000000000000000000000000000000000000000000000000000000
0000000000000000000000
0000000000000000000000000000000000000000000000000000000000000000000000000000000000000000000000000000000000000000000000000000000000000000000000000000000000000000000000000000000
0000000000000000000000000000000000000000000000000000000000000000000000000000000000000000000000000000000000000000000000000000000000000000000000000000000000000000000000000000000
0000000000000000000000
>DNA_Q86IH1
MNTVEENNTKITDNNNNNNNNNNNNNNNNNNNKNNDGENLSQQLIDSNNKKLRSPIQVYFDSDVSSCSLQSQSSPQQLPTQQPIQQQINNIFNHFLPQNQAPLLQAQNQQPINNINQVSNFQIQPIPLTFQLPNNINTNTNNTNMINNVQQFQNNFLNNNDFSLTDPVPSPFVLF
NNNNNNNNNNNNNNNNNINNFHHNNINNNFDNNNSNYNTINTHPNNTNNNSSNINQIQSNIYPQQNLHNPFLPIHNNNNINNNNINKNNNNYNNNNNNKNNNNNNNNNNNNNNPQLFSMEPPNGFNHSTTDNTSSVSSVPSNKKKSSKTKQKSKPLTIQQQQQHKSNYHQQPNQN
SQHLQSKPNSPILISSPLNSQQNSSPPQPSPTQSFLSPPQYSQSPQNNNFNNNNNNISNNNNNNNNNNNNNNNNNNNNNNNNNNNNNNNNNNNNNNNNNNNNNINNSTNNNNNNSNTNNNTNTNTNNNNNKNNNNNNNNEIENNNNEELILLWYNSIIKNIIVDENHFKIENQFN
KENGYLVFKQLFKNHFGSIGTIGKEMAEYIQLHCEVMNEITELSKFHFQSIDNFYNNYIRIINGYSLIPAKDSGATIKARPKKGAKLSKESKDILENWIKNHIAHPYPTNDEKEQLQRQTGLTPNQISNWFINTRRRKVPTLCDEAGIITNSNVMPQMTINNNNNNGGNSNFKNN
NNNTITTTSTSNNNNNNNNNNHNEMECDDGENEESSEYDD
0000000000000000000000000000000000000000000000000000000000000000000000000000000000000000000000000000000000000000000000000000000000000000000000000000000000000000000000000000000
0000000000000000000000000000000000000000000000000000000000000000000000000000000000000000000000000000000000000000000000000000000000000000000000000000000000000000000000000000000
0000000000000000000000000000000000000000000000000000000000000000000000000000000000000000000000000000000000000000000000000000000000000000000000000000000000000000000000000000000
0000000000000000000000000000000000000000000000000000000000000000000000000000000000000000000000000000000000000000000000000000000000000000000000000000000000000000000000000000000
0000000000000000000000000000000000000000
0000000000000000000000000000000000000000000000000000000000000000000000000000000000000000000000000000000000000000000000000000000000000000000000000000000000000000000000000000000
0000000000000000000000000000000000000000000000000000000000000000000000000000000000000000000000000000000000000000000000000000000000000000000000000000000000000000000000000000000
0000000000000000000000000000000000000000000000000000000000000000000000000000000000000000000000000000000000000000000000000000000000000000000000000000000000000000000000000000000
0000000000000000000000000000000000000000000000000000000000000000000000000000000000000000000000000000000000000000000000000000000000000000000000000000000000000000000000000000000
0000000000000000000000000000000000000000
0000000000000000000000000000000000000000000000000000000000000000000000000000000000000000000000000000000000000000000000000000000000000000000000000000000000000000000000000000000
0000000000000000000000000000000000000000000000000000000000000000000000000000000000000000000000000000000000000000000000000000000000000000000000000000000000000000000000000000000
0000000000000000000000000000000000000000000000000000000000000000000000000000000000000000000000000000000000000000000000000000000000000000000000000000000000000000000000000000000
0000000000000000000000000000000000000000000000000000000000000000000000000000000000000000000000000000000000000000000000000000000000000000000000000000000000000000000000000000000
0000000000000000000000000000000000000000
0000000000000000000000000000000000000000000000000000000000000000000000000000000000000000000000000000000000000000000000000000000000000000000000000000000000000000000000000000000
0000000000000000000000000000000000000000000000000000000000000000000000000000000000000000000000000000000000000000000000000000000000000000000000000000000000000000000000000000000
0000000000000000000000000000000000000000000000000000000000000000000000000000000000000000000000000000000000000000000000000000000000000000000000000000000000000000000000000000000
0000000000000000000000000000000000000000000000000000000000000000000000000000000000000000000000000000000000000000000000000000000000000000000000000000000000000000000000000000000
0000000000000000000000000000000000000000
0000000000000000000000000000000000000000000000000000000000000000000000000000000000000000000000000000000000000000000000000000000000000000000000000000000000000000000000000000000
0000000000000000000000000000000000000000000000000000000000000000000000000000000000000000000000000000000000000000000000000000000000000000000000000000000000000000000000000000000
0000000000000000000000000000000000000000000000000000000000000000000000000000000000000000000000000000000000000000000000000000000000000000000000000000000000000000000000000000000
0000000000000000000000000000000000000000000000000000000000000000000000000000000000000000000000000000000000000000000000000000000000000000000000000000000000000000000000000000000
0000000000000000000000000000000000000000

0000000000000000000000000000000000000000000000000000000000000000000000000000000000000000000000000000000000000000000000000000000000000000000000000000000000000000000000000000000
0000000000000000000000000000000000000000000000000000000000000000000000000000000000000000000000000000000000000000000000000000000000000000000000000000000000000000000000000000000
0000000000000000000000000000000000000000000000000000000000000000000000000000000000000000000000000000000000000000000000000000000000000000000000000000000000000000000000000000000
0000000000000000000000000000000000000000000000000000000000000000000000000000000000000000000000000000000000000000000000000000000000000000000000000000000000000000000000000000000
0000000000000000000000000000000000000000
0000000000000000000000000000000000000000000000000000000000000000000000000000000000000000000000000000000000000000000000000000000000000000000000000000000000000000000000000000000
0000000000000000000000000000000000000000000000000000000000000000000000000000000000000000000000000000000000000000000000000000000000000000000000000000000000000000000000000000000
0000000000000000000000000000000000000000000000000000000000000000000000000000000000000000000000000000000000000000000000000000000000000000000000000000000000000000000000000000000
0000000000000000000000000000000000000000000000000000000000000000000000000000000000000000000000000000000000000000000000000000000000000000000000000000000000000000000000000000000
0000000000000000000000000000000000000000
>DNA_Q8CN77
MYVENSYLSKQLCFLFYVSSKEIIKKYTDYLKEYGLTYTGYIVLMAIEDDEKLNIKKLGERVFLDSGTLTPLLKKLEKKNYVIRTREEQDERNLQISLTERGKDIQNVLSDISQSVFNEFNITQEETQNLVEDLQNFVTKNFDKTVEK
0000000000000000000000000000000000000000000000000000000000000000000000000000000000000000000000000000000000000000000000000000000000000000000000000000
0000000000000000000000000000000000000000000000000000000000000000000000000000000000000000000000000000000000000000000000000000000000000000000000000000
0000000000000000000000000000000000000000000000000000000000000000000000000000000000000000000000000000000000000000000000000000000000000000000000000000
0000000000000000000000000000000000000000000000000000000000000000000000000000000000000000000000000000000000000000000000000000000000000000000000000000
0000000000000000000000000000000000000000000000000000000000000000000000000000000000000000000000000000000000000000000000000000000000000000000000000000
0000000000000000000000000000000000000000000000000000000000000000000000000000000000000000000000000000000000000000000000000000000000000000000000000000
0000000000000000000000000000000000000000000000000000000000000000000000000000000000000000000000000000000000000000000000000000000000000000000000000000
>DNA_Q8GWF1
MEMNSPHEKAVQAIRYGHSCAMRLKRRLNHPMADGGPLSSYDLAKSIVESFSNAISILSAKPETEDDQFSDLSSRDSSPPPQGSPSKKRKIDSTNSSENWRDDSPDPIYYDGYLWRKYGQKSIKKSNHQRSYYRCSYNKDHNCEARKHEQKIKDNPPVYRTTYFGHHTCKTEHNL
DAIFIAGQDPLDDFKSTQMIRFGKDQDQEKESRSNGFSLSVKHEEDIIKEQAIDQYREITSNDQDCQDVIEEYLSSPSGSYPPSSSSGSESADFNSDLLFDNPDSWDRYDQFYF
0000000000000000000000000000000000000000000000000000000000000000000000000000000000000000000000000000000000000000000000000000000000000000000000000000000000000000000000000000000
000000000000000000000000000000000000000000000000000000000000000000000000000000000000000000000000000000000000000000
0000000000000000000000000000000000000000000000000000000000000000000000000000000000000000000000000000000000000000000000000000000000000000000000000000000000000000000000000000000
000000000000000000000000000000000000000000000000000000000000000000000000000000000000000000000000000000000000000000
0000000000000000000000000000000000000000000000000000000000000000000000000000000000000000000000000000000000000000000000000000000000000000000000000000000000000000000000000000000
000000000000000000000000000000000000000000000000000000000000000000000000000000000000000000000000000000000000000000
0000000000000000000000000000000000000000000000000000000000000000000000000000000000000000000000000000000000000000000000000000000000000000000000000000000000000000000000000000000
000000000000000000000000000000000000000000000000000000000000000000000000000000000000000000000000000000000000000000
0000000000000000000000000000000000000000000000000000000000000000000000000000000000000000000000000000000000000000000000000000000000000000000000000000000000000000000000000000000
000000000000000000000000000000000000000000000000000000000000000000000000000000000000000000000000000000000000000000
0000000000000000000000000000000000000000000000000000000000000000000000000000000000000000000000000000000000000000000000000000000000000000000000000000000000000000000000000000000
000000000000000000000000000000000000000000000000000000000000000000000000000000000000000000000000000000000000000000
0000000000000000000000000000000000000000000000000000000000000000000000000000000000000000000000000000000000000000000000000000000000000000000000000000000000000000000000000000000
000000000000000000000000000000000000000000000000000000000000000000000000000000000000000000000000000000000000000000
>DNA_Q8K402
MALSSRAHAFSVEALMGRPSKRKAQDPREEMQPELQEEQFVEEGEEILRSPSRDSQQPEKRLKAESSKTVFSCSDESNSQESLQEESVIQVELQGSDLWKRFHDIGTEMIITKAGRRMFPSVRIKVKGMDPVKQYYVILDVVPVDSKRYRYVYHSSQWMVAGNTDHSCITPRFYV
HPDSPCSGENWMRQIISFDRVKLTNNEMDDKGHIILQSMHKYNPRVHVVEQDSRIDLSLIESFPTEGVKTFSFKETEFTTVTAYQNQQITKLKIDRNPFAKGFRDPGRNRGVLDGFLETYPWMPSFSMDFKTFVTDTQSGSSGSSPVTSSGGAPSPLNSLLSPSCSPPMVYIPPS
SFGMTYPDAYLHSVNIPFCYRICPTNNWRSQPFVLPTPERLPSFIIPQTLPPLMMEVPVVSSRGIINPNSGLHEDCNGQCLQASHSANQMLYGLQNPGNIFHPNAIAQEAISYPFHPPNGCYRYTISMPPRLENVASHLSENGTSQISFTEGSCDHSHWYPAINHYL
0000000000000000000000000000000000000000000000000000000000000000000000000000000000000000000000000000000000000000000000000000000000000000000000000000000000000000000000000000000
0000000000000000000000000000000000000000000000000000000000000000000000000000000000000000000000000000000000000000000000000000000000000000000000000000000000000000000000000000000
00000000000000000000000000000000000000000000000000000000000000000000000000000000000000000000000000000000000000000000000000000000000000000000000000000000000000000000000
0000000000000000000000000000000000000000000000000000000000000000000000000000000000000000000000000000000000000000000000000000000000000000000000000000000000000000000000000000000
0000000000000000000000000000000000000000000000000000000000000000000000000000000000000000000000000000000000000000000000000000000000000000000000000000000000000000000000000000000
00000000000000000000000000000000000000000000000000000000000000000000000000000000000000000000000000000000000000000000000000000000000000000000000000000000000000000000000
0000000000000000000000000000000000000000000000000000000000000000000000000000000000000000000000000000000000000000000000000000000000000000000000000000000000000000000000000000000
0000000000000000000000000000000000000000000000000000000000000000000000000000000000000000000000000000000000000000000000000000000000000000000000000000000000000000000000000000000
00000000000000000000000000000000000000000000000000000000000000000000000000000000000000000000000000000000000000000000000000000000000000000000000000000000000000000000000
0000000000000000000000000000000000000000000000000000000000000000000000000000000000000000000000000000000000000000000000000000000000000000000000000000000000000000000000000000000
0000000000000000000000000000000000000000000000000000000000000000000000000000000000000000000000000000000000000000000000000000000000000000000000000000000000000000000000000000000
00000000000000000000000000000000000000000000000000000000000000000000000000000000000000000000000000000000000000000000000000000000000000000000000000000000000000000000000
0000000000000000000000000000000000000000000000000000000000000000000000000000000000000000000000000000000000000000000000000000000000000000000000000000000000000000000000000000000
0000000000000000000000000000000000000000000000000000000000000000000000000000000000000000000000000000000000000000000000000000000000000000000000000000000000000000000000000000000
00000000000000000000000000000000000000000000000000000000000000000000000000000000000000000000000000000000000000000000000000000000000000000000000000000000000000000000000
0000000000000000000000000000000000000000000000000000000000000000000000000000000000000000000000000000000000000000000000000000000000000000000000000000000000000000000000000000000
0000000000000000000000000000000000000000000000000000000000000000000000000000000000000000000000000000000000000000000000000000000000000000000000000000000000000000000000000000000
00000000000000000000000000000000000000000000000000000000000000000000000000000000000000000000000000000000000000000000000000000000000000000000000000000000000000000000000
0000000000000000000000000000000000000000000000000000000000000000000000000000000000000000000000000000000000000000000000000000000000000000000000000000000000000000000000000000000
0000000000000000000000000000000000000000000000000000000000000000000000000000000000000000000000000000000000000000000000000000000000000000000000000000000000000000000000000000000
00000000000000000000000000000000000000000000000000000000000000000000000000000000000000000000000000000000000000000000000000000000000000000000000000000000000000000000000
>DNA_Q8L3U3
MNNNWLGFSLSPYEQNHHRKDVYSSTTTTVVDVAGEYCYDPTAASDESSAIQTSFPSPFGVVVDAFTRDNNSHSRDWDINGCACNNIHNDEQDGPKLENFLGRTTTIYNTNENVGDGSGSGCYGGGDGGGGSLGLSMIKTWLRNQPVDNVDNQENGNAAKGLSLSMNSSTSCDNN
NDSNNNVVAQGKTIDDSVEATPKKTIESFGQRTSIYRGVTRHRWTGRYEAHLWDNSCKREGQTRKGRQVYLGGYDKEEKAARAYDLAALKYWGTTTTTNFPMSEYEKEVEEMKHMTRQEYVASLRRKSSGFSRGASIYRGVTRHHQHGRWQARIGRVAGNKDLYLGTFGTQEEAA
EAYDIAAIKFRGLTAVTNFDMNRYNVKAILESPSLPIGSAAKRLKEANRPVPSMMMISNNVSESENSASGWQNAAVQHHQGVDLSLLHQHQERYNGYYYNGGNLSSESARACFKQEDDQHHFLSNTQSLMTNIDHQSSVSDDSVTVCGNVVGYGGYQGFAAPVNCDAYAASEFDY
NARNHYYFAQQQQTQQSPGGDFPAAMTNNVGSNMYYHGEGGGEVAPTFTVWNDN
0000000000000000000000000000000000000000000000000000000000000000000000000000000000000000000000000000000000000000000000000000000000000000000000000000000000000000000000000000000
0000000000000000000000000000000000000000000000000000000000000000000000000000000000000000000000000000000000000000000000000000000000000000000000000000000000000000000000000000000

0000000000000000000000000000000000000000000000000000000000000000000000000000000000000000000000000000000000000000000000000000000000000000000000000000000000000000000000000000000
000000000000000000000000000000000000000000000000000000
0000000000000000000000000000000000000000000000000000000000000000000000000000000000000000000000000000000000000000000000000000000000000000000000000000000000000000000000000000000
0000000000000000000000000000000000000000000000000000000000000000000000000000000000000000000000000000000000000000000000000000000000000000000000000000000000000000000000000000000
0000000000000000000000000000000000000000000000000000000000000000000000000000000000000000000000000000000000000000000000000000000000000000000000000000000000000000000000000000000
000000000000000000000000000000000000000000000000000000
0000000000000000000000000000000000000000000000000000000000000000000000000000000000000000000000000000000000000000000000000000000000000000000000000000000000000000000000000000000
0000000000000000000000000000000000000000000000000000000000000000000000000000000000000000000000000000000000000000000000000000000000000000000000000000000000000000000000000000000
0000000000000000000000000000000000000000000000000000000000000000000000000000000000000000000000000000000000000000000000000000000000000000000000000000000000000000000000000000000
000000000000000000000000000000000000000000000000000000
0000000000000000000000000000000000000000000000000000000000000000000000000000000000000000000000000000000000000000000000000000000000000000000000000000000000000000000000000000000
0000000000000000000000000000000000000000000000000000000000000000000000000000000000000000000000000000000000000000000000000000000000000000000000000000000000000000000000000000000
0000000000000000000000000000000000000000000000000000000000000000000000000000000000000000000000000000000000000000000000000000000000000000000000000000000000000000000000000000000
000000000000000000000000000000000000000000000000000000
0000000000000000000000000000000000000000000000000000000000000000000000000000000000000000000000000000000000000000000000000000000000000000000000000000000000000000000000000000000
0000000000000000000000000000000000000000000000000000000000000000000000000000000000000000000000000000000000000000000000000000000000000000000000000000000000000000000000000000000
0000000000000000000000000000000000000000000000000000000000000000000000000000000000000000000000000000000000000000000000000000000000000000000000000000000000000000000000000000000
000000000000000000000000000000000000000000000000000000
0000000000000000000000000000000000000000000000000000000000000000000000000000000000000000000000000000000000000000000000000000000000000000000000000000000000000000000000000000000
0000000000000000000000000000000000000000000000000000000000000000000000000000000000000000000000000000000000000000000000000000000000000000000000000000000000000000000000000000000
0000000000000000000000000000000000000000000000000000000000000000000000000000000000000000000000000000000000000000000000000000000000000000000000000000000000000000000000000000000
000000000000000000000000000000000000000000000000000000
0000000000000000000000000000000000000000000000000000000000000000000000000000000000000000000000000000000000000000000000000000000000000000000000000000000000000000000000000000000
0000000000000000000000000000000000000000000000000000000000000000000000000000000000000000000000000000000000000000000000000000000000000000000000000000000000000000000000000000000
0000000000000000000000000000000000000000000000000000000000000000000000000000000000000000000000000000000000000000000000000000000000000000000000000000000000000000000000000000000
000000000000000000000000000000000000000000000000000000
>DNA_Q8RZX9
MDSSSCLVDDTNSGGSSTDKLRALAAAAAETAPLERMGSGASAVVDAAEPGAEADSGSGGRVCGGGGGGAGGAGGKLPSSKFKGVVPQPNGRWGAQIYERHQRVWLGTFAGEDDAARAYDVAAQRFRGRDAVTNFRPLAEADPDAAAELRFLATRSKAEVVDMLRKHTYFDELAQ
SKRTFAASTPSAATTTASLSNGHLSSPRSPFAPAAARDHLFDKTVTPSDVGKLNRLVIPKQHAEKHFPLQLPSAGGESKGVLLNFEDAAGKVWRFRYSYWNSSQSYVLTKGWSRFVKEKGLHAGDVVGFYRSAASAGDDGKLFIDCKLVRSTGAALASPADQPAPSPVKAVRLFG
VDLLTAPAPVEQMAGCKRARDLAATTPPQAAAFKKQCIELALV
0000000000000000000000000000000000000000000000000000000000000000000000000000000000000000000000000000000000000000000000000000000000000000000000000000000000000000000000000000000
0000000000000000000000000000000000000000000000000000000000000000000000000000000000000000000000000000000000000000000000000000000000000000000000000000000000000000000000000000000
0000000000000000000000000000000000000000000
0000000000000000000000000000000000000000000000000000000000000000000000000000000000000000000000000000000000000000000000000000000000000000000000000000000000000000000000000000000
0000000000000000000000000000000000000000000000000000000000000000000000000000000000000000000000000000000000000000000000000000000000000000000000000000000000000000000000000000000
0000000000000000000000000000000000000000000
0000000000000000000000000000000000000000000000000000000000000000000000000000000000000000000000000000000000000000000000000000000000000000000000000000000000000000000000000000000
0000000000000000000000000000000000000000000000000000000000000000000000000000000000000000000000000000000000000000000000000000000000000000000000000000000000000000000000000000000
0000000000000000000000000000000000000000000
0000000000000000000000000000000000000000000000000000000000000000000000000000000000000000000000000000000000000000000000000000000000000000000000000000000000000000000000000000000
0000000000000000000000000000000000000000000000000000000000000000000000000000000000000000000000000000000000000000000000000000000000000000000000000000000000000000000000000000000
0000000000000000000000000000000000000000000
0000000000000000000000000000000000000000000000000000000000000000000000000000000000000000000000000000000000000000000000000000000000000000000000000000000000000000000000000000000
0000000000000000000000000000000000000000000000000000000000000000000000000000000000000000000000000000000000000000000000000000000000000000000000000000000000000000000000000000000
0000000000000000000000000000000000000000000
0000000000000000000000000000000000000000000000000000000000000000000000000000000000000000000000000000000000000000000000000000000000000000000000000000000000000000000000000000000
0000000000000000000000000000000000000000000000000000000000000000000000000000000000000000000000000000000000000000000000000000000000000000000000000000000000000000000000000000000
0000000000000000000000000000000000000000000
0000000000000000000000000000000000000000000000000000000000000000000000000000000000000000000000000000000000000000000000000000000000000000000000000000000000000000000000000000000
0000000000000000000000000000000000000000000000000000000000000000000000000000000000000000000000000000000000000000000000000000000000000000000000000000000000000000000000000000000
0000000000000000000000000000000000000000000
>DNA_Q90229
MSTILTDLSSSLSCHAASKDSPTLPESSATDLGYYSTHGGTHSPHDYFQSQPYPQPINHHYPYHQFNLNGLGGPGTYSPKSDYPYGGSYRQYGHYRESAMAVQEPVSVKEEPEPEVRMVNGKPKKIRKPRTIYSSYQLAALQRRFQKAQYLALPERAELAAQLGLTQTQVKIWFQ
NRRSKFKKLYKNGEVPGMEHSPDNSDSMACNSPASPPVWDSNPPSRVPHPQAQPLPHNSSPSYLEDYNPGTTMNRTWRGTPAAAQLDAPYPSGTGSVLAGCWWKN
0000000000000000000000000000000000000000000000000000000000000000000000000000000000000000000000000000000000000000000000000000000000000000000000000000000000000000000000000000000
000000000000000000000000000000000000000000000000000000000000000000000000000000000000000000000000000000000
0000000000000000000000000000000000000000000000000000000000000000000000000000000000000000000000000000000000000000000000000000000000000000000000000000000000000000000000000000000
000000000000000000000000000000000000000000000000000000000000000000000000000000000000000000000000000000000
0000000000000000000000000000000000000000000000000000000000000000000000000000000000000000000000000000000000000000000000000000000000000000000000000000000000000000000000000000000
000000000000000000000000000000000000000000000000000000000000000000000000000000000000000000000000000000000
0000000000000000000000000000000000000000000000000000000000000000000000000000000000000000000000000000000000000000000000000000000000000000000000000000000000000000000000000000000
000000000000000000000000000000000000000000000000000000000000000000000000000000000000000000000000000000000
0000000000000000000000000000000000000000000000000000000000000000000000000000000000000000000000000000000000000000000000000000000000000000000000000000000000000000000000000000000
000000000000000000000000000000000000000000000000000000000000000000000000000000000000000000000000000000000
0000000000000000000000000000000000000000000000000000000000000000000000000000000000000000000000000000000000000000000000000000000000000000000000000000000000000000000000000000000
000000000000000000000000000000000000000000000000000000000000000000000000000000000000000000000000000000000
0000000000000000000000000000000000000000000000000000000000000000000000000000000000000000000000000000000000000000000000000000000000000000000000000000000000000000000000000000000
000000000000000000000000000000000000000000000000000000000000000000000000000000000000000000000000000000000
>DNA_Q90472

MDGGGLDEEFINVYPSAFGTHSSRCTSGAPVLSAVDRPTSVCNESISPYFSFPSNIGSGSFTFGCHLENSYKVPQNAVFPPGVAKQNGQFANKPVDHGEASSWLKEFAFYQGCARSYPRIPAFIDLPVVQRAMMGDLRHETCLTMEGHQHWDWSNNCSSQLYCFQDQTRSPHIWK
PSLTEEAAAASFCQRGRKKRVPYTKFQLKELEREYNTTKFITKENRRRIASSTNLSERQVTIWFQNRRVKDKKRPDVCIKC
0000000000000000000000000000000000000000000000000000000000000000000000000000000000000000000000000000000000000000000000000000000000000000000000000000000000000000000000000000000
000000000000000000000000000000000000000000000000000000000000000000000000000000000
0000000000000000000000000000000000000000000000000000000000000000000000000000000000000000000000000000000000000000000000000000000000000000000000000000000000000000000000000000000
000000000000000000000000000000000000000000000000000000000000000000000000000000000
0000000000000000000000000000000000000000000000000000000000000000000000000000000000000000000000000000000000000000000000000000000000000000000000000000000000000000000000000000000
000000000000000000000000000000000000000000000000000000000000000000000000000000000
0000000000000000000000000000000000000000000000000000000000000000000000000000000000000000000000000000000000000000000000000000000000000000000000000000000000000000000000000000000
000000000000000000000000000000000000000000000000000000000000000000000000000000000
0000000000000000000000000000000000000000000000000000000000000000000000000000000000000000000000000000000000000000000000000000000000000000000000000000000000000000000000000000000
000000000000000000000000000000000000000000000000000000000000000000000000000000000
0000000000000000000000000000000000000000000000000000000000000000000000000000000000000000000000000000000000000000000000000000000000000000000000000000000000000000000000000000000
000000000000000000000000000000000000000000000000000000000000000000000000000000000
0000000000000000000000000000000000000000000000000000000000000000000000000000000000000000000000000000000000000000000000000000000000000000000000000000000000000000000000000000000
000000000000000000000000000000000000000000000000000000000000000000000000000000000
>DNA_Q93356
MTSYSYFTIPSTATTGFNYPVQPMTMFSGAPYNAAMIPRKNRRERTTYSRQQLEILETLFNETQYPDVFARERVADQIRLQESRIQVWFKNRRAKYRLQEKQKPKRSNEKSQEHKSEDQQQTDVLDGEPLKGGSPGYQPQIKSELESCDGAVASGKLGTPKSISPVETTASTTSS
NTSAAELQWNGDHKILGFGKNETTTSAAVSPTADNASTPSSSSSITATSSLPTTSSSLSVYNYPAIYPQWGLDYSTYANPQYAQFSHNPYAGTPFWYSNGNNL
0000000000000000000000000000000000000000000000000000000000000000000000000000000000000000000000000000000000000000000000000000000000000000000000000000000000000000000000000000000
0000000000000000000000000000000000000000000000000000000000000000000000000000000000000000000000000000000
0000000000000000000000000000000000000000000000000000000000000000000000000000000000000000000000000000000000000000000000000000000000000000000000000000000000000000000000000000000
0000000000000000000000000000000000000000000000000000000000000000000000000000000000000000000000000000000
0000000000000000000000000000000000000000000000000000000000000000000000000000000000000000000000000000000000000000000000000000000000000000000000000000000000000000000000000000000
0000000000000000000000000000000000000000000000000000000000000000000000000000000000000000000000000000000
0000000000000000000000000000000000000000000000000000000000000000000000000000000000000000000000000000000000000000000000000000000000000000000000000000000000000000000000000000000
0000000000000000000000000000000000000000000000000000000000000000000000000000000000000000000000000000000
0000000000000000000000000000000000000000000000000000000000000000000000000000000000000000000000000000000000000000000000000000000000000000000000000000000000000000000000000000000
0000000000000000000000000000000000000000000000000000000000000000000000000000000000000000000000000000000
0000000000000000000000000000000000000000000000000000000000000000000000000000000000000000000000000000000000000000000000000000000000000000000000000000000000000000000000000000000
0000000000000000000000000000000000000000000000000000000000000000000000000000000000000000000000000000000
0000000000000000000000000000000000000000000000000000000000000000000000000000000000000000000000000000000000000000000000000000000000000000000000000000000000000000000000000000000
0000000000000000000000000000000000000000000000000000000000000000000000000000000000000000000000000000000
>DNA_Q93899
MQSFDIESLIGVNKVPSLVEMVAASRASSFSPPFEQQHHDPMGVVAAAAAAAAAGRHHPYDNRDDGQMRRYRTAFSREQIGRLEREFAKENYVSRKTRGELAAELNLPEGTIKVWFQNRRMKDKRQRVGGLAWPFPPQMAAYMLNPFAYEMWMKTAAASQFGATGPGNGAYGNNG
SSTSPSAAGSLPFLPPLGFPSFLSQNSTKSPSSPHSDDSSKSKNTSSDDDESKPVNFSNSPSSSSPSPYSTD
0000000000000000000000000000000000000000000000000000000000000000000000000000000000000000000000000000000000000000000000000000000000000000000000000000000000000000000000000000000
000000000000000000000000000000000000000000000000000000000000000000000000
0000000000000000000000000000000000000000000000000000000000000000000000000000000000000000000000000000000000000000000000000000000000000000000000000000000000000000000000000000000
000000000000000000000000000000000000000000000000000000000000000000000000
0000000000000000000000000000000000000000000000000000000000000000000000000000000000000000000000000000000000000000000000000000000000000000000000000000000000000000000000000000000
000000000000000000000000000000000000000000000000000000000000000000000000
0000000000000000000000000000000000000000000000000000000000000000000000000000000000000000000000000000000000000000000000000000000000000000000000000000000000000000000000000000000
000000000000000000000000000000000000000000000000000000000000000000000000
0000000000000000000000000000000000000000000000000000000000000000000000000000000000000000000000000000000000000000000000000000000000000000000000000000000000000000000000000000000
000000000000000000000000000000000000000000000000000000000000000000000000
0000000000000000000000000000000000000000000000000000000000000000000000000000000000000000000000000000000000000000000000000000000000000000000000000000000000000000000000000000000
000000000000000000000000000000000000000000000000000000000000000000000000
0000000000000000000000000000000000000000000000000000000000000000000000000000000000000000000000000000000000000000000000000000000000000000000000000000000000000000000000000000000
000000000000000000000000000000000000000000000000000000000000000000000000
>DNA_Q949N0
MGRGSVTSLAPGFRFHPTDEELVRYYLKRKICNKPFKFDAISVTDVYKSEPWDLPDKSRLKSRDLEWYFFSMLDKKYRNGSKTNRATEMGYWKTTGKDREILNGSKVVGMKKTLVYHKGRAPRGERTNWVMHEYRLVDQDLDKTGVHQDAFVLCRIFQKSGSGPKNGEQYGAPFV
EEEWEEEDDMTFVPDQEDLGSEDHVYVHMDDIDQKSENFVVYDAIPIPLNFIHGESSNNVETNYSDSINYIQQTGNYMDSGGYFEQPAESYEKDQKPIIRDRDGSLQNEGIGCGVQDKHSETLQSSDNIFGTDTSCYNDFPVESNYLIGEAFLDPNSNLLENDGLYLETNDLSST
QQDGFDFEDYLTFFDETFDPSQLMGNEDVFFDQEELFQEVETKELEKEETSRSKHVVEEKEKDEASCSKQVDADATEFEPDYKYPLLKKASHMLGAIPAPLANASEFPTKDAAIRLHAAQSSGSVHVTAGMITISDSNMGWSYGKNENLDLILSLGLVQGNTAPEKSGNSSAWAM
LIFMCFWVLLLSVSFKVSILVSSR
0000000000000000000000000000000000000000000000000000000000000000000000000000000000000000000000000000000000000000000000000000000000000000000000000000000000000000000000000000000
0000000000000000000000000000000000000000000000000000000000000000000000000000000000000000000000000000000000000000000000000000000000000000000000000000000000000000000000000000000
0000000000000000000000000000000000000000000000000000000000000000000000000000000000000000000000000000000000000000000000000000000000000000000000000000000000000000000000000000000
000000000000000000000000
0000000000000000000000000000000000000000000000000000000000000000000000000000000000000000000000000000000000000000000000000000000000000000000000000000000000000000000000000000000
0000000000000000000000000000000000000000000000000000000000000000000000000000000000000000000000000000000000000000000000000000000000000000000000000000000000000000000000000000000
0000000000000000000000000000000000000000000000000000000000000000000000000000000000000000000000000000000000000000000000000000000000000000000000000000000000000000000000000000000
000000000000000000000000
0000000000000000000000000000000000000000000000000000000000000000000000000000000000000000000000000000000000000000000000000000000000000000000000000000000000000000000000000000000
0000000000000000000000000000000000000000000000000000000000000000000000000000000000000000000000000000000000000000000000000000000000000000000000000000000000000000000000000000000
0000000000000000000000000000000000000000000000000000000000000000000000000000000000000000000000000000000000000000000000000000000000000000000000000000000000000000000000000000000
000000000000000000000000
0000000000000000000000000000000000000000000000000000000000000000000000000000000000000000000000000000000000000000000000000000000000000000000000000000000000000000000000000000000
0000000000000000000000000000000000000000000000000000000000000000000000000000000000000000000000000000000000000000000000000000000000000000000000000000000000000000000000000000000

0000000000000000000000000000000000000000000000000000000000000000000000000000000000000000000000000000000000000000000000000000000000000000000000000000000000000000000000000000000
000000000000000000000000
0000000000000000000000000000000000000000000000000000000000000000000000000000000000000000000000000000000000000000000000000000000000000000000000000000000000000000000000000000000
0000000000000000000000000000000000000000000000000000000000000000000000000000000000000000000000000000000000000000000000000000000000000000000000000000000000000000000000000000000
0000000000000000000000000000000000000000000000000000000000000000000000000000000000000000000000000000000000000000000000000000000000000000000000000000000000000000000000000000000
000000000000000000000000
0000000000000000000000000000000000000000000000000000000000000000000000000000000000000000000000000000000000000000000000000000000000000000000000000000000000000000000000000000000
0000000000000000000000000000000000000000000000000000000000000000000000000000000000000000000000000000000000000000000000000000000000000000000000000000000000000000000000000000000
0000000000000000000000000000000000000000000000000000000000000000000000000000000000000000000000000000000000000000000000000000000000000000000000000000000000000000000000000000000
000000000000000000000000
0000000000000000000000000000000000000000000000000000000000000000000000000000000000000000000000000000000000000000000000000000000000000000000000000000000000000000000000000000000
0000000000000000000000000000000000000000000000000000000000000000000000000000000000000000000000000000000000000000000000000000000000000000000000000000000000000000000000000000000
0000000000000000000000000000000000000000000000000000000000000000000000000000000000000000000000000000000000000000000000000000000000000000000000000000000000000000000000000000000
000000000000000000000000
>DNA_Q97W73
MEEKVGNLKPNMESVNVTVRVLEASEARQIQTKNGVRTISEAIVGDETGRVKLTLWGKHAGSIKEGQVVKIENAWTTAFKGQVQLNAGSKTKIAEASEDGFPESSQIPENTPTAPQQMRGGGRGFRGGGRRYGRRGGRRQENEEGEEE
0000000000000000000000000000000000000000000000000000000000000000000000000000000000000000000000000000000000000000000000000000000000000000000000000000
0000000000000000000000000000000000000000000000000000000000000000000000000000000000000000000000000000000000000000000000000000000000000000000000000000
0000000000000000000000000000000000000000000000000000000000000000000000000000000000000000000000000000000000000000000000000000000000000000000000000000
0000000000000000000000000000000000000000000000000000000000000000000000000000000000000000000000000000000000000000000000000000000000000000000000000000
0000000000000000000000000000000000000000000000000000000000000000000000000000000000000000000000000000000000000000000000000000000000000000000000000000
0000000000000000000000000000000000000000000000000000000000000000000000000000000000000000000000000000000000000000000000000000000000000000000000000000
0000000000000000000000000000000000000000000000000000000000000000000000000000000000000000000000000000000000000000000000000000000000000000000000000000
>DNA_Q9ADP7
MAARDPEATKARIFEAAVAEFARHGIAGARIDRIAAEARANKQLIYAYYGNKGELFASVLEKKMLDLAISVPVDPDDIEGWIDRLLDYHAAHPELLRLLFWEGMEYGTAELPHEAERQEHYARKVAAVRDGQERGVITDAIPAPDLLFLLVAMANWAVVVPQMKRILVGGGDAGT
DGLRDSIKKAARRIVDR
0000000000000000000000000000000000000000000000000000000000000000000000000000000000000000000000000000000000000000000000000000000000000000000000000000000000000000000000000000000
00000000000000000
0000000000000000000000000000000000000000000000000000000000000000000000000000000000000000000000000000000000000000000000000000000000000000000000000000000000000000000000000000000
00000000000000000
0000000000000000000000000000000000000000000000000000000000000000000000000000000000000000000000000000000000000000000000000000000000000000000000000000000000000000000000000000000
00000000000000000
0000000000000000000000000000000000000000000000000000000000000000000000000000000000000000000000000000000000000000000000000000000000000000000000000000000000000000000000000000000
00000000000000000
0000000000000000000000000000000000000000000000000000000000000000000000000000000000000000000000000000000000000000000000000000000000000000000000000000000000000000000000000000000
00000000000000000
0000000000000000000000000000000000000000000000000000000000000000000000000000000000000000000000000000000000000000000000000000000000000000000000000000000000000000000000000000000
00000000000000000
0000000000000000000000000000000000000000000000000000000000000000000000000000000000000000000000000000000000000000000000000000000000000000000000000000000000000000000000000000000
00000000000000000
>DNA_Q9BVI0
MTKHPPNRRGISFEVGAQLEARDRLKNWYPAHIEDIDYEEGKVLIHFKRWNHRYDEWFCWDSPYLRPLEKIQLRKEGLHEEDGSSEFQINEQVLACWSDCRFYPAKVTAVNKDGTYTVKFYDGVVQTVKHIHVKAFSKDQNIVGNARPKETDHKSLSSSPDKREKFKEQRKATVN
VKKDKEDKPLKTEKRPKQPDKEGKLICSEKGKVSEKSLPKNEKEDKENISENDREYSGDAQVDKKPENDIVKSPQENLREPKRKRGRPPSIAPTAVDSNSQTLQPITLELRRRKISKGCEVPLKRPRLDKNSSQEKSKNYSENTDKDLSRRRSSRLSTNGTHEILDPDLVVSDLV
DTDPLQDTLSSTKESEEGQLKSALEAGQVSSALTCHSFGDGSGAAGLELNCPSMGENTMKTEPTSPLVELQEISTVEVTNTFKKTDDFGSSNAPAVDLDHKFRCKVVDCLKFFRKAKLLHYHMKYFHGMEKSLEPEESPGKRHVQTRGPSASDKPSQETLTRKRVSASSPTTKDK
EKNKEKKFKEFVRVKPKKKKKKKKKTKPECPCSEEISDTSQEPSPPKAFAVTRCGSSHKPGVHMSPQLHGPESGHHKGKVKALEEDNLSESSSESFLWSDDEYGQDVDVTTNPDEELDGDDRYDFEVVRCICEVQEENDFMIQCEECQCWQHGVCMGLLEENVPEKYTCYVCQDP
PGQRPGFKYWYDKEWLSRGHMHGLAFLEENYSHQNAKKIVATHQLLGDVQRVIEVLHGLQLKMSILQSREHPDLPLWCQPWKQHSGEGRSHFRNIPVTDTRSKEEAPSYRTLNGAVEKPRPLALPLPRSVEESYITSEHCYQKPRAYYPAVEQKLVVETRGSALDDAVNPLHENG
DDSLSPRLGWPLDQDRSKGDSDPKPGSPKVKEYVSKKALPEEAPARKLLDRGGEGLLSSQHQWQFNLLTHVESLQDEVTHRMDSIEKELDVLESWLDYTGELEPPEPLARLPQLKHCIKQLLMDLGKVQQIALCCST
0000000000000000000000000000000000000000000000000000000000000000000000000000000000000000000000000000000000000000000000000000000000000000000000000000000000000000000000000000000
0000000000000000000000000000000000000000000000000000000000000000000000000000000000000000000000000000000000000000000000000000000000000000000000000000000000000000000000000000000
0000000000000000000000000000000000000000000000000000000000000000000000000000000000000000000000000000000000000000000000000000000000000000000000000000000000000000000000000000000
0000000000000000000000000000000000000000000000000000000000000000000000000000000000000000000000000000000000000000000000000000000000000000000000000000000000000000000000000000000
0000000000000000000000000000000000000000000000000000000000000000000000000000000000000000000000000000000000000000000000000000000000000000000000000000000000000000000000000000000
00000000000000000000000000000000000000000000000000000000000000000000000000000000000000000000000000000000000000000000000000000000000000000
0000000000000000000000000000000000000000000000000000000000000000000000000000000000000000000000000000000000000000000000000000000000000000000000000000000000000000000000000000000
0000000000000000000000000000000000000000000000000000000000000000000000000000000000000000000000000000000000000000000000000000000000000000000000000000000000000000000000000000000
0000000000000000000000000000000000000000000000000000000000000000000000000000000000000000000000000000000000000000000000000000000000000000000000000000000000000000000000000000000
0000000000000000000000000000000000000000000000000000000000000000000000000000000000000000000000000000000000000000000000000000000000000000000000000000000000000000000000000000000
0000000000000000000000000000000000000000000000000000000000000000000000000000000000000000000000000000000000000000000000000000000000000000000000000000000000000000000000000000000
00000000000000000000000000000000000000000000000000000000000000000000000000000000000000000000000000000000000000000000000000000000000000000
0000000000000000000000000000000000000000000000000000000000000000000000000000000000000000000000000000000000000000000000000000000000000000000000000000000000000000000000000000000
0000000000000000000000000000000000000000000000000000000000000000000000000000000000000000000000000000000000000000000000000000000000000000000000000000000000000000000000000000000
0000000000000000000000000000000000000000000000000000000000000000000000000000000000000000000000000000000000000000000000000000000000000000000000000000000000000000000000000000000
0000000000000000000000000000000000000000000000000000000000000000000000000000000000000000000000000000000000000000000000000000000000000000000000000000000000000000000000000000000
0000000000000000000000000000000000000000000000000000000000000000000000000000000000000000000000000000000000000000000000000000000000000000000000000000000000000000000000000000000
00000000000000000000000000000000000000000000000000000000000000000000000000000000000000000000000000000000000000000000000000000000000000000
0000000000000000000000000000000000000000000000000000000000000000000000000000000000000000000000000000000000000000000000000000000000000000000000000000000000000000000000000000000
0000000000000000000000000000000000000000000000000000000000000000000000000000000000000000000000000000000000000000000000000000000000000000000000000000000000000000000000000000000
0000000000000000000000000000000000000000000000000000000000000000000000000000000000000000000000000000000000000000000000000000000000000000000000000000000000000000000000000000000
0000000000000000000000000000000000000000000000000000000000000000000000000000000000000000000000000000000000000000000000000000000000000000000000000000000000000000000000000000000

0000000000000000000000000000000000000000000000000000000000000000000000000000000000000000000000000000000000000000000000000000000000000000000000000000000000000000000000000000000
00000000000000000000000000000000000000000000000000000000000000000000000000000000000000000000000000000000000000000000000000000000000000000
0000000000000000000000000000000000000000000000000000000000000000000000000000000000000000000000000000000000000000000000000000000000000000000000000000000000000000000000000000000
0000000000000000000000000000000000000000000000000000000000000000000000000000000000000000000000000000000000000000000000000000000000000000000000000000000000000000000000000000000
0000000000000000000000000000000000000000000000000000000000000000000000000000000000000000000000000000000000000000000000000000000000000000000000000000000000000000000000000000000
0000000000000000000000000000000000000000000000000000000000000000000000000000000000000000000000000000000000000000000000000000000000000000000000000000000000000000000000000000000
0000000000000000000000000000000000000000000000000000000000000000000000000000000000000000000000000000000000000000000000000000000000000000000000000000000000000000000000000000000
00000000000000000000000000000000000000000000000000000000000000000000000000000000000000000000000000000000000000000000000000000000000000000
0000000000000000000000000000000000000000000000000000000000000000000000000000000000000000000000000000000000000000000000000000000000000000000000000000000000000000000000000000000
0000000000000000000000000000000000000000000000000000000000000000000000000000000000000000000000000000000000000000000000000000000000000000000000000000000000000000000000000000000
0000000000000000000000000000000000000000000000000000000000000000000000000000000000000000000000000000000000000000000000000000000000000000000000000000000000000000000000000000000
0000000000000000000000000000000000000000000000000000000000000000000000000000000000000000000000000000000000000000000000000000000000000000000000000000000000000000000000000000000
0000000000000000000000000000000000000000000000000000000000000000000000000000000000000000000000000000000000000000000000000000000000000000000000000000000000000000000000000000000
00000000000000000000000000000000000000000000000000000000000000000000000000000000000000000000000000000000000000000000000000000000000000000
0000000000000000000000000000000000000000000000000000000000000000000000000000000000000000000000000000000000000000000000000000000000000000000000000000000000000000000000000000000
0000000000000000000000000000000000000000000000000000000000000000000000000000000000000000000000000000000000000000000000000000000000000000000000000000000000000000000000000000000
0000000000000000000000000000000000000000000000000000000000000000000000000000000000000000000000000000000000000000000000000000000000000000000000000000000000000000000000000000000
0000000000000000000000000000000000000000000000000000000000000000000000000000000000000000000000000000000000000000000000000000000000000000000000000000000000000000000000000000000
0000000000000000000000000000000000000000000000000000000000000000000000000000000000000000000000000000000000000000000000000000000000000000000000000000000000000000000000000000000
00000000000000000000000000000000000000000000000000000000000000000000000000000000000000000000000000000000000000000000000000000000000000000
>DNA_Q9BZM3
MSRSFYVDSLIIKDTSRPAPSLPEPHPGPDFFIPLGMPPPLVMSVSGPGCPSRKSGAFCVCPLCVTSHLHSSRGSVGAGSGGAGAGVTGAGGSGVAGAAGALPLLKGQFSSAPGDAQFCPRVNHAHHHHHPPQHHHHHHQPQQPGSAAAAAAAAAAAAAAAALGHPQHHAPVCTA
TTYNVADPRRFHCLTMGGSDASQVPNGKRMRTAFTSTQLLELEREFSSNMYLSRLRRIEIATYLNLSEKQVKIWFQNRRVKHKKEGKGTQRNSHAGCKCVGSQVHYARSEDEDSLSPASANDDKEISPL
0000000000000000000000000000000000000000000000000000000000000000000000000000000000000000000000000000000000000000000000000000000000000000000000000000000000000000000000000000000
000000000000000000000000000000000000000000000000000000000000000000000000000000000000000000000000000000000000000000000000000000000
0000000000000000000000000000000000000000000000000000000000000000000000000000000000000000000000000000000000000000000000000000000000000000000000000000000000000000000000000000000
000000000000000000000000000000000000000000000000000000000000000000000000000000000000000000000000000000000000000000000000000000000
0000000000000000000000000000000000000000000000000000000000000000000000000000000000000000000000000000000000000000000000000000000000000000000000000000000000000000000000000000000
000000000000000000000000000000000000000000000000000000000000000000000000000000000000000000000000000000000000000000000000000000000
0000000000000000000000000000000000000000000000000000000000000000000000000000000000000000000000000000000000000000000000000000000000000000000000000000000000000000000000000000000
000000000000000000000000000000000000000000000000000000000000000000000000000000000000000000000000000000000000000000000000000000000
0000000000000000000000000000000000000000000000000000000000000000000000000000000000000000000000000000000000000000000000000000000000000000000000000000000000000000000000000000000
000000000000000000000000000000000000000000000000000000000000000000000000000000000000000000000000000000000000000000000000000000000
0000000000000000000000000000000000000000000000000000000000000000000000000000000000000000000000000000000000000000000000000000000000000000000000000000000000000000000000000000000
000000000000000000000000000000000000000000000000000000000000000000000000000000000000000000000000000000000000000000000000000000000
0000000000000000000000000000000000000000000000000000000000000000000000000000000000000000000000000000000000000000000000000000000000000000000000000000000000000000000000000000000
000000000000000000000000000000000000000000000000000000000000000000000000000000000000000000000000000000000000000000000000000000000
>DNA_Q9DEN4
MTLSGSGSASDMSGQTVLSADDADIDVVGEGDEALDKDSECESTAGHTDEVGELGGKEIPRSPSGSGTEAEGKGESQQQQQEGIQNKPKNSLVKPPYSYIALITMSILQSPQKKLTLSGICEFISNRFPYYREKFPAWQNSIRHNLSLNDCFVKIPREPGNPGKGNYWTLDPQSE
DMFDNGSFLRRRKRFKRQQQDSLREQTALMMQSFGAYSLASPYGRHYGLHPAAYTHPAALQYPYIPPVGHMLPPAVPLLPSSELTRKAFSSQLSPSLQLQLSSLSSTAASIIKSEPSSRPSFSIENIIGVSAASSAAPHTFLRPPVTVQSALMSHQPLALSRSTAAIGPILSVPT
NLISGQFLPTAAAAVAKWPAQ
0000000000000000000000000000000000000000000000000000000000000000000000000000000000000000000000000000000000000000000000000000000000000000000000000000000000000000000000000000000
0000000000000000000000000000000000000000000000000000000000000000000000000000000000000000000000000000000000000000000000000000000000000000000000000000000000000000000000000000000
000000000000000000000
0000000000000000000000000000000000000000000000000000000000000000000000000000000000000000000000000000000000000000000000000000000000000000000000000000000000000000000000000000000
0000000000000000000000000000000000000000000000000000000000000000000000000000000000000000000000000000000000000000000000000000000000000000000000000000000000000000000000000000000
000000000000000000000
0000000000000000000000000000000000000000000000000000000000000000000000000000000000000000000000000000000000000000000000000000000000000000000000000000000000000000000000000000000
0000000000000000000000000000000000000000000000000000000000000000000000000000000000000000000000000000000000000000000000000000000000000000000000000000000000000000000000000000000
000000000000000000000
0000000000000000000000000000000000000000000000000000000000000000000000000000000000000000000000000000000000000000000000000000000000000000000000000000000000000000000000000000000
0000000000000000000000000000000000000000000000000000000000000000000000000000000000000000000000000000000000000000000000000000000000000000000000000000000000000000000000000000000
000000000000000000000
0000000000000000000000000000000000000000000000000000000000000000000000000000000000000000000000000000000000000000000000000000000000000000000000000000000000000000000000000000000
0000000000000000000000000000000000000000000000000000000000000000000000000000000000000000000000000000000000000000000000000000000000000000000000000000000000000000000000000000000
000000000000000000000
0000000000000000000000000000000000000000000000000000000000000000000000000000000000000000000000000000000000000000000000000000000000000000000000000000000000000000000000000000000
0000000000000000000000000000000000000000000000000000000000000000000000000000000000000000000000000000000000000000000000000000000000000000000000000000000000000000000000000000000
000000000000000000000
0000000000000000000000000000000000000000000000000000000000000000000000000000000000000000000000000000000000000000000000000000000000000000000000000000000000000000000000000000000
0000000000000000000000000000000000000000000000000000000000000000000000000000000000000000000000000000000000000000000000000000000000000000000000000000000000000000000000000000000
000000000000000000000
>DNA_Q9HUW0
MTTETLVSGTTPVSDNANLKQHLTTPTQEGQTLRDSVEKALHNYFAHLEGQPVTDVYNMVLCEVEAPLLETVMNHVKGNQTKASELLGLNRGTLRKKLKQYDLL
00000000000000000000000000000000000000000000000000000000000000000000000000000000000000000000000000000000
00000000000000000000000000000000000000000000000000000000000000000000000000000000000000000000000000000000
00000000000000000000000000000000000000000000000000000000000000000000000000000000000000000000000000000000
00000000000000000000000000000000000000000000000000000000000000000000000000000000000000000000000000000000
00000000000000000000000000000000000000000000000000000000000000000000000000000000000000000000000000000000

00000000000000000000000000000000000000000000000000000000000000000000000000000000000000000000000000000000
00000000000000000000000000000000000000000000000000000000000000000000000000000000000000000000000000000000
>DNA_Q9KPI4
MLLEGIETLLVLSKEKTMSRTGSQLYISQSAVSKRIANLEKKLGKKLIVPAGRHIKLTADAEQLIASIGPTFNELHGLIFEQQTLEDNTLLRMDCSETLVAGYLGQTMGQHRKQDPHWVITTNHTPRIVENVQSGKATLGFCAGYLPANHGLLTFHLGDEPFTVISQAPLHALPS
ELITNDLANPANTYQAAMLHKQGIRPAMEMDSYTAAAQLALGGMLPALVPRSIVSTLKIEPQHCFDFAELADLTRPIHICLRASRYRSSRVQALITALHDAVPKAVLAPSA
0000000000000000000000000000000000000000000000000000000000000000000000000000000000000000000000000000000000000000000000000000000000000000000000000000000000000000000000000000000
000000000000000000000000000000000000000000000000000000000000000000000000000000000000000000000000000000000000000
0000000000000000000000000000000000000000000000000000000000000000000000000000000000000000000000000000000000000000000000000000000000000000000000000000000000000000000000000000000
000000000000000000000000000000000000000000000000000000000000000000000000000000000000000000000000000000000000000
0000000000000000000000000000000000000000000000000000000000000000000000000000000000000000000000000000000000000000000000000000000000000000000000000000000000000000000000000000000
000000000000000000000000000000000000000000000000000000000000000000000000000000000000000000000000000000000000000
0000000000000000000000000000000000000000000000000000000000000000000000000000000000000000000000000000000000000000000000000000000000000000000000000000000000000000000000000000000
000000000000000000000000000000000000000000000000000000000000000000000000000000000000000000000000000000000000000
0000000000000000000000000000000000000000000000000000000000000000000000000000000000000000000000000000000000000000000000000000000000000000000000000000000000000000000000000000000
000000000000000000000000000000000000000000000000000000000000000000000000000000000000000000000000000000000000000
0000000000000000000000000000000000000000000000000000000000000000000000000000000000000000000000000000000000000000000000000000000000000000000000000000000000000000000000000000000
000000000000000000000000000000000000000000000000000000000000000000000000000000000000000000000000000000000000000
0000000000000000000000000000000000000000000000000000000000000000000000000000000000000000000000000000000000000000000000000000000000000000000000000000000000000000000000000000000
000000000000000000000000000000000000000000000000000000000000000000000000000000000000000000000000000000000000000
>DNA_Q9LXV2
MRKPEVAIAASTHQVKKMKKGLWSPEEDSKLMQYMLSNGQGCWSDVAKNAGLQRCGKSCRLRWINYLRPDLKRGAFSPQEEDLIIRFHSILGNRWSQIAARLPGRTDNEIKNFWNSTIKKRLKKMSDTSNLINNSSSSPNTASDSSSNSASSLDIKDIIGSFMSLQEQGFVNPSL
THIQTNNPFPTGNMISHPCNDDFTPYVDGIYGVNAGVQGELYFPPLECEEGDWYNANINNHLDELNTNGSGNAPEGMRPVEEFWDLDQLMNTEVPSFYFNFKQSI
0000000000000000000000000000000000000000000000000000000000000000000000000000000000000000000000000000000000000000000000000000000000000000000000000000000000000000000000000000000
000000000000000000000000000000000000000000000000000000000000000000000000000000000000000000000000000000000
0000000000000000000000000000000000000000000000000000000000000000000000000000000000000000000000000000000000000000000000000000000000000000000000000000000000000000000000000000000
000000000000000000000000000000000000000000000000000000000000000000000000000000000000000000000000000000000
0000000000000000000000000000000000000000000000000000000000000000000000000000000000000000000000000000000000000000000000000000000000000000000000000000000000000000000000000000000
000000000000000000000000000000000000000000000000000000000000000000000000000000000000000000000000000000000
0000000000000000000000000000000000000000000000000000000000000000000000000000000000000000000000000000000000000000000000000000000000000000000000000000000000000000000000000000000
000000000000000000000000000000000000000000000000000000000000000000000000000000000000000000000000000000000
0000000000000000000000000000000000000000000000000000000000000000000000000000000000000000000000000000000000000000000000000000000000000000000000000000000000000000000000000000000
000000000000000000000000000000000000000000000000000000000000000000000000000000000000000000000000000000000
0000000000000000000000000000000000000000000000000000000000000000000000000000000000000000000000000000000000000000000000000000000000000000000000000000000000000000000000000000000
000000000000000000000000000000000000000000000000000000000000000000000000000000000000000000000000000000000
0000000000000000000000000000000000000000000000000000000000000000000000000000000000000000000000000000000000000000000000000000000000000000000000000000000000000000000000000000000
000000000000000000000000000000000000000000000000000000000000000000000000000000000000000000000000000000000
>DNA_Q9NR48
MDPRNTAMLGLGSDSEGFSRKSPSAISTGTLVSKREVELEKNTKEEEDLRKRNRERNIEAGKDDGLTDAQQQFSVKETNFSEGNLKLKIGLQAKRTKKPPKNLENYVCRPAIKTTIKHPRKALKSGKMTDEKNEHCPSKRDPSKLYKKADDVAAIECQSEEVIRLHSQGENNPLS
KKLSPVHSEMADYINATPSTLLGSRDPDLKDRALLNGGTSVTEKLAQLIATCPPSKSSKTKPKKLGTGTTAGLVSKDLIRKAGVGSVAGIIHKDLIKKPTISTAVGLVTKDPGKKPVFNAAVGLVNKDSVKKLGTGTTAVFINKNLGKKPGTITTVGLLSKDSGKKLGIGIVPGL
VHKESGKKLGLGTVVGLVNKDLGKKLGSTVGLVAKDCAKKIVASSAMGLVNKDIGKKLMSCPLAGLISKDAINLKAEALLPTQEPLKASCSTNINNQESQELSESLKDSATSKTFEKNVVRQNKESILEKFSVRKEIINLEKEMFNEGTCIQQDSFSSSEKGSYETSKHEKQPPV
YCTSPDFKMGGASDVSTAKSPFSAVGESNLPSPSPTVSVNPLTRSPPETSSQLAPNPLLLSSTTELIEEISESVGKNQFTSESTHLNVGHRSVGHSISIECKGIDKEVNDSKTTHIDIPRISSSLGKKPSLTSESSIHTITPSVVNFTSLFSNKPFLKLGAVSASDKHCQVAESL
STSLQSKPLKKRKGRKPRWTKVVARSTCRSPKGLELERSELFKNVSCSSLSNSNSEPAKFMKNIGPPSFVDHDFLKRRLPKLSKSTAPSLALLADSEKPSHKSFATHKLSSSMCVSSDLLSDIYKPKRGRPKSKEMPQLEGPPKRTLKIPASKVFSLQSKEEQEPPILQPEIEIP
SFKQGLSVSPFPKKRGRPKRQMRSPVKMKPPVLSVAPFVATESPSKLESESDNHRSSSDFFESEDQLQDPDDLDDSHRPSVCSMSDLEMEPDKKITKRNNGQLMKTIIRKINKMKTLKRKKLLNQILSSSVESSNKGKVQSKLHNTVSSLAATFGSKLGQQINVSKKGTIYIGKR
RGRKPKTVLNGILSGSPTSLAVLEQTAQQAAGSALGQILPPLLPSSASSSEILPSPICSQSSGTSGGQSPVSSDAGFVEPSSVPYLHLHSRQGSMIQTLAMKKASKGRRRLSPPTLLPNSPSHLSELTSLKEATPSPISESHSDETIPSDSGIGTDNNSTSDRAEKFCGQKKRRH
SFEHVSLIPPETSTVLSSLKEKHKHKCKRRNHDYLSYDKMKRQKRKRKKKYPQLRNRQDPDFIAELEELISRLSEIRITHRSHHFIPRDLLPTIFRINFNSFYTHPSFPLDPLHYIRKPDLKKKRGRPPKMREAMAEMPFMHSLSFPLSSTGFYPSYGMPYSPSPLTAAPIGLGY
YGRYPPTLYPPPPSPSFTTPLPPPSYMHAGHLLLNPAKYHKKKHKLLRQEAFLTTSRTPLLSMSTYPSVPPEMAYGWMVEHKHRHRHKHREHRSSEQPQVSMDTGSSRSVLESLKRYRFGKDAVGERYKHKEKHRCHMSCPHLSPSKSLINREEQWVHREPSESSPLALGLQTPL
QIDCSESSPSLSLGGFTPNSEPASSDEHTNLFTSAIGSCRVSNPNSSGRKKLTDSPGLFSAQDTSLNRLHRKESLPSNERAVQTLAGSQPTSDKPSQRPSESTNCSPTRKRSSSESTSSTVNGVPSRSPRLVASGDDSVDSLLQRMVQNEDQEPMEKSIDAVIATASAPPSSSPG
RSHSKDRTLGKPDSLLVPAVTSDSCNNSISLLSEKLTSSCSPHHIKRSVVEAMQRQARKMCNYDKILATKKNLDHVNKILKAKKLQRQARTGNNFVKRRPGRPRKCPLQAVVSMQAFQAAQFVNPELNRDEEGAALHLSPDTVTDVIEAVVQSVNLNPEHKKGLKRKGWLLEEQT
RKKQKPLPEEEEQENNKSFNEAPVEIPSPSETPAKPSEPESTLQPVLSLIPREKKPPRPPKKKYQKAGLYSDVYKTTDPKSRLIQLKKEKLEYTPGEHEYGLFPAPIHVVFFVSGKYLRQKRIDFQLPYDILWQWKHNQLYKKPDVPLYKKIRSNVYVDVKPLSGYEATTCNCKK
PDDDTRKGCVDDCLNRMIFAECSPNTCPCGEQCCNQRIQRHEWVQCLERFRAEEKGWGIRTKEPLKAGQFIIEYLGEVVSEQEFRNRMIEQYHNHSDHYCLNLDSGMVIDSYRMGNEARFINHSCDPNCEMQKWSVNGVYRIGLYALKDMPAGTELTYDYNFHSFNVEKQQLCKC
GFEKCRGIIGGKSQRVNGLTSSKNSQPMATHKKSGRSKEKRKSKHKLKKRRGHLSEEPSENINTPTRLTPQLQMKPMSNRERNFVLKHHVFLVRNWEKIRQKQEEVKHTSDNIHSASLYTRWNGICRDDGNIKSDVFMTQFSALQTARSVRTRRLAAAEENIEVARAARLAQIFK
EICDGIISYKDSSRQALAAPLLNLPPKKKNADYYEKISDPLDLITIEKQILTGYYKTVEAFDADMLKVFRNAEKYYGRKSPVGRDVCRLRKAYYNARHEASAQIDEIVGETASEADSSETSVSEKENGHEKDDDVIRCICGLYKDEGLMIQCDKCMVWQHCDCMGVNSDVEHYLC
EQCDPRPVDREVPMIPRPHYAQPGCVYFICLLRDDLLLRQGDCVYLMRDSRRTPDGHPVRQSYRLLSHINRDKLDIFRIEKLWKNEKEERFAFGHHYFRPHETHHSPSRRFYHNELFRVPLYEIIPLEAVVGTCCVLDLYTYCKGRPKGVKEQDVYICDYRLDKSAHLFYKIHRN
RYPVCTKPYAFDHFPKKLTPKKDFSPHYVPDNYKRNGGRSSWKSERSKPPLKDLGQEDDALPLIEEVLASQEQAANEIPSLEEPEREGATANVSEGEKKTEESSQEPQSTCTPEERRHNQRERLNQILLNLLEKIPGKNAIDVTYLLEEGSGRKLRRRTLFIPENSFRK
0000000000000000000000000000000000000000000000000000000000000000000000000000000000000000000000000000000000000000000000000000000000000000000000000000000000000000000000000000000
0000000000000000000000000000000000000000000000000000000000000000000000000000000000000000000000000000000000000000000000000000000000000000000000000000000000000000000000000000000
0000000000000000000000000000000000000000000000000000000000000000000000000000000000000000000000000000000000000000000000000000000000000000000000000000000000000000000000000000000
0000000000000000000000000000000000000000000000000000000000000000000000000000000000000000000000000000000000000000000000000000000000000000000000000000000000000000000000000000000
0000000000000000000000000000000000000000000000000000000000000000000000000000000000000000000000000000000000000000000000000000000000000000000000000000000000000000000000000000000
0000000000000000000000000000000000000000000000000000000000000000000000000000000000000000000000000000000000000000000000000000000000000000000000000000000000000000000000000000000
0000000000000000000000000000000000000000000000000000000000000000000000000000000000000000000000000000000000000000000000000000000000000000000000000000000000000000000000000000000
0000000000000000000000000000000000000000000000000000000000000000000000000000000000000000000000000000000000000000000000000000000000000000000000000000000000000000000000000000000
0000000000000000000000000000000000000000000000000000000000000000000000000000000000000000000000000000000000000000000000000000000000000000000000000000000000000000000000000000000
0000000000000000000000000000000000000000000000000000000000000000000000000000000000000000000000000000000000000000000000000000000000000000000000000000000000000000000000000000000
0000000000000000000000000000000000000000000000000000000000000000000000000000000000000000000000000000000000000000000000000000000000000000000000000000000000000000000000000000000
0000000000000000000000000000000000000000000000000000000000000000000000000000000000000000000000000000000000000000000000000000000000000000000000000000000000000000000000000000000
0000000000000000000000000000000000000000000000000000000000000000000000000000000000000000000000000000000000000000000000000000000000000000000000000000000000000000000000000000000
0000000000000000000000000000000000000000000000000000000000000000000000000000000000000000000000000000000000000000000000000000000000000000000000000000000000000000000000000000000
0000000000000000000000000000000000000000000000000000000000000000000000000000000000000000000000000000000000000000000000000000000000000000000000000000000000000000000000000000000

00
00
00
00
00
00
00
00
00
00
00
00
00
00
00
00
00
00
00
00
00
00
00
00
00
00
00
00
00
00
00
00
00
00
00
00
00
00
00
00
00
00
00
00
00
00
00
00
00
00
00
00
00
00
00
00
00
00
00
00
00
00
00
00
00
00
00
00

0000000000000000000000000000000000000000000000000000000000000000000000000000000000000000000000000000000000000000000000000000000000000000000000000000000000000000000000000000000
0000000000000000000000000000000000000000000000000000000000000000000000000000000000000000000000000000000000000000000000000000000000000000000000000000000000000000000000000
0000000000000000000000000000000000000000000000000000000000000000000000000000000000000000000000000000000000000000000000000000000000000000000000000000000000000000000000000000000
0000000000000000000000000000000000000000000000000000000000000000000000000000000000000000000000000000000000000000000000000000000000000000000000000000000000000000000000000000000
0000000000000000000000000000000000000000000000000000000000000000000000000000000000000000000000000000000000000000000000000000000000000000000000000000000000000000000000000000000
0000000000000000000000000000000000000000000000000000000000000000000000000000000000000000000000000000000000000000000000000000000000000000000000000000000000000000000000000000000
0000000000000000000000000000000000000000000000000000000000000000000000000000000000000000000000000000000000000000000000000000000000000000000000000000000000000000000000000000000
0000000000000000000000000000000000000000000000000000000000000000000000000000000000000000000000000000000000000000000000000000000000000000000000000000000000000000000000000000000
0000000000000000000000000000000000000000000000000000000000000000000000000000000000000000000000000000000000000000000000000000000000000000000000000000000000000000000000000000000
0000000000000000000000000000000000000000000000000000000000000000000000000000000000000000000000000000000000000000000000000000000000000000000000000000000000000000000000000000000
0000000000000000000000000000000000000000000000000000000000000000000000000000000000000000000000000000000000000000000000000000000000000000000000000000000000000000000000000000000
0000000000000000000000000000000000000000000000000000000000000000000000000000000000000000000000000000000000000000000000000000000000000000000000000000000000000000000000000000000
0000000000000000000000000000000000000000000000000000000000000000000000000000000000000000000000000000000000000000000000000000000000000000000000000000000000000000000000000000000
0000000000000000000000000000000000000000000000000000000000000000000000000000000000000000000000000000000000000000000000000000000000000000000000000000000000000000000000000000000
0000000000000000000000000000000000000000000000000000000000000000000000000000000000000000000000000000000000000000000000000000000000000000000000000000000000000000000000000000000
0000000000000000000000000000000000000000000000000000000000000000000000000000000000000000000000000000000000000000000000000000000000000000000000000000000000000000000000000000000
0000000000000000000000000000000000000000000000000000000000000000000000000000000000000000000000000000000000000000000000000000000000000000000000000000000000000000000000000000000
0000000000000000000000000000000000000000000000000000000000000000000000000000000000000000000000000000000000000000000000000000000000000000000000000000000000000000000000000000000
0000000000000000000000000000000000000000000000000000000000000000000000000000000000000000000000000000000000000000000000000000000000000000000000000000000000000000000000000
0000000000000000000000000000000000000000000000000000000000000000000000000000000000000000000000000000000000000000000000000000000000000000000000000000000000000000000000000000000
0000000000000000000000000000000000000000000000000000000000000000000000000000000000000000000000000000000000000000000000000000000000000000000000000000000000000000000000000000000
0000000000000000000000000000000000000000000000000000000000000000000000000000000000000000000000000000000000000000000000000000000000000000000000000000000000000000000000000000000
0000000000000000000000000000000000000000000000000000000000000000000000000000000000000000000000000000000000000000000000000000000000000000000000000000000000000000000000000000000
0000000000000000000000000000000000000000000000000000000000000000000000000000000000000000000000000000000000000000000000000000000000000000000000000000000000000000000000000000000
0000000000000000000000000000000000000000000000000000000000000000000000000000000000000000000000000000000000000000000000000000000000000000000000000000000000000000000000000000000
0000000000000000000000000000000000000000000000000000000000000000000000000000000000000000000000000000000000000000000000000000000000000000000000000000000000000000000000000000000
0000000000000000000000000000000000000000000000000000000000000000000000000000000000000000000000000000000000000000000000000000000000000000000000000000000000000000000000000000000
0000000000000000000000000000000000000000000000000000000000000000000000000000000000000000000000000000000000000000000000000000000000000000000000000000000000000000000000000000000
0000000000000000000000000000000000000000000000000000000000000000000000000000000000000000000000000000000000000000000000000000000000000000000000000000000000000000000000000000000
0000000000000000000000000000000000000000000000000000000000000000000000000000000000000000000000000000000000000000000000000000000000000000000000000000000000000000000000000000000
0000000000000000000000000000000000000000000000000000000000000000000000000000000000000000000000000000000000000000000000000000000000000000000000000000000000000000000000000000000
0000000000000000000000000000000000000000000000000000000000000000000000000000000000000000000000000000000000000000000000000000000000000000000000000000000000000000000000000000000
0000000000000000000000000000000000000000000000000000000000000000000000000000000000000000000000000000000000000000000000000000000000000000000000000000000000000000000000000000000
0000000000000000000000000000000000000000000000000000000000000000000000000000000000000000000000000000000000000000000000000000000000000000000000000000000000000000000000000000000
0000000000000000000000000000000000000000000000000000000000000000000000000000000000000000000000000000000000000000000000000000000000000000000000000000000000000000000000000000000
0000000000000000000000000000000000000000000000000000000000000000000000000000000000000000000000000000000000000000000000000000000000000000000000000000000000000000000000000
>DNA_Q9S7B2
MKERQRWRPEEDAVLRAYVRQYGPREWHLVSQRMNVALDRDAKSCLERWKNYLRPGIKKGSLTEEEQRLVIRLQAKHGNKWKKIAAEVPGRTAKRLGKWWEVFKEKQQRELRDSRRPPPEPSPDERGRYEWLLENFAEKLVGERPQQAAAAPSPLLMAAPVLPPWLSSNAGPAAA
AAAAVAHPPPRPPSPSVTLSLASAAVAPGPPAPAPWMPDRAAADAAPYGFPSPSQHGGAAPPGMAVVDGQALAELAECCRELEEGRRAWAAHRREAAWRLKRVEQQLEMEREMRRREVWEEFEAKMRTMRLEQAAAAERVERDHREKVAELRRDAQVKEEKMAEQWAAKHARVAK
FVEQMGGCSRSWSSATDMNC
0000000000000000000000000000000000000000000000000000000000000000000000000000000000000000000000000000000000000000000000000000000000000000000000000000000000000000000000000000000
0000000000000000000000000000000000000000000000000000000000000000000000000000000000000000000000000000000000000000000000000000000000000000000000000000000000000000000000000000000
00000000000000000000
0000000000000000000000000000000000000000000000000000000000000000000000000000000000000000000000000000000000000000000000000000000000000000000000000000000000000000000000000000000
0000000000000000000000000000000000000000000000000000000000000000000000000000000000000000000000000000000000000000000000000000000000000000000000000000000000000000000000000000000
00000000000000000000
0000000000000000000000000000000000000000000000000000000000000000000000000000000000000000000000000000000000000000000000000000000000000000000000000000000000000000000000000000000
0000000000000000000000000000000000000000000000000000000000000000000000000000000000000000000000000000000000000000000000000000000000000000000000000000000000000000000000000000000
00000000000000000000
0000000000000000000000000000000000000000000000000000000000000000000000000000000000000000000000000000000000000000000000000000000000000000000000000000000000000000000000000000000
0000000000000000000000000000000000000000000000000000000000000000000000000000000000000000000000000000000000000000000000000000000000000000000000000000000000000000000000000000000
00000000000000000000
0000000000000000000000000000000000000000000000000000000000000000000000000000000000000000000000000000000000000000000000000000000000000000000000000000000000000000000000000000000
0000000000000000000000000000000000000000000000000000000000000000000000000000000000000000000000000000000000000000000000000000000000000000000000000000000000000000000000000000000
00000000000000000000
0000000000000000000000000000000000000000000000000000000000000000000000000000000000000000000000000000000000000000000000000000000000000000000000000000000000000000000000000000000
0000000000000000000000000000000000000000000000000000000000000000000000000000000000000000000000000000000000000000000000000000000000000000000000000000000000000000000000000000000
00000000000000000000
0000000000000000000000000000000000000000000000000000000000000000000000000000000000000000000000000000000000000000000000000000000000000000000000000000000000000000000000000000000
0000000000000000000000000000000000000000000000000000000000000000000000000000000000000000000000000000000000000000000000000000000000000000000000000000000000000000000000000000000
00000000000000000000
>DNA_Q9SGU3
MGKGRAPCCDKNKVKRGPWSPQEDLTLITFIQKHGHQNWRSLPKLAGLLRCGKSCRLRWINYLRPDVKRGNFSKKEEDAIIHYHQTLGNKWSKIASFLPGRTDNEIKNVWNTHLKKRLTPSSSSSSLSSTHDQSTKADHDKNCDGAQEEIHSGLNESQNSATSSHHQGECMHTKP
ELHEVNGLNEIQFLLDHDDFDDITSEFLQDNDILFPLDSLLHNHQTHISTQEMTREVTKSQSFDHPQPDIPCGFEDTNEESDLRRQLVESTTPNNEYDEWFNFIDNQTYFDDFNFVGEVCL
0000000000000000000000000000000000000000000000000000000000000000000000000000000000000000000000000000000000000000000000000000000000000000000000000000000000000000000000000000000
0000000000000000000000000000000000000000000000000000000000000000000000000000000000000000000000000000000000000000000000000
0000000000000000000000000000000000000000000000000000000000000000000000000000000000000000000000000000000000000000000000000000000000000000000000000000000000000000000000000000000
0000000000000000000000000000000000000000000000000000000000000000000000000000000000000000000000000000000000000000000000000

00000000000000000000000000000000000000000000000000000000000000000000000000000000000000000000000000000000000000000000000000000000000000000000000000000000000000000000000000000000
00000000000000000000000000000000000000000000000000000000000000000000000000000000000000000000000000000000000000000000000000
00000000000000000000000000000000000000000000000000000000000000000000000000000000000000000000000000000000000000000000000000000000000000000000000000000000000000000000000000000000
00000000000000000000000000000000000000000000000000000000000000000000000000000000000000000000000000000000000000000000000000
00000000000000000000000000000000000000000000000000000000000000000000000000000000000000000000000000000000000000000000000000000000000000000000000000000000000000000000000000000000
00000000000000000000000000000000000000000000000000000000000000000000000000000000000000000000000000000000000000000000000000
00000000000000000000000000000000000000000000000000000000000000000000000000000000000000000000000000000000000000000000000000000000000000000000000000000000000000000000000000000000
00000000000000000000000000000000000000000000000000000000000000000000000000000000000000000000000000000000000000000000000000
00000000000000000000000000000000000000000000000000000000000000000000000000000000000000000000000000000000000000000000000000000000000000000000000000000000000000000000000000000000
00000000000000000000000000000000000000000000000000000000000000000000000000000000000000000000000000000000000000000000000000
>DNA_Q9SM27
MIQDQANDLLAMKKTFTKSKWKPEEDRILKDYVIQYGDRTWTHVPKRTGLPHNPASCRFRWMNHLKPSLKKGPFTDEEEKRVLQLHAVLGNKWSQMAREFPGRTDNEIKNFWNARRMRLKGKGLPVYPDEVREQAIRTAAQYGVKVELLNAHYSQDSLMAGNVEKPQELNNLALN
QCSPYYQSTLANVQPSRNRVMEPETTFPFTGGSAMNEQNPTLLCNPYVESTQEQLPDSHLFGNVTYSSPPMPLIHEVENLELPSFQGFDFHEEPSSFGAEQYNPMLNLEPHNTLVQSPLIGQTPTDFPSSFYDELLDELLESVVNGSLGEIPKTDTSSESQLFQSSLRSHTDATP
DIANTTGYVGSNERNTTNDDDWIRLLLDEGFI
00000000000000000000000000000000000000000000000000000000000000000000000000000000000000000000000000000000000000000000000000000000000000000000000000000000000000000000000000000000
00000000000000000000000000000000000000000000000000000000000000000000000000000000000000000000000000000000000000000000000000000000000000000000000000000000000000000000000000000000
00000000000000000000000000000000
00000000000000000000000000000000000000000000000000000000000000000000000000000000000000000000000000000000000000000000000000000000000000000000000000000000000000000000000000000000
00000000000000000000000000000000000000000000000000000000000000000000000000000000000000000000000000000000000000000000000000000000000000000000000000000000000000000000000000000000
00000000000000000000000000000000
00000000000000000000000000000000000000000000000000000000000000000000000000000000000000000000000000000000000000000000000000000000000000000000000000000000000000000000000000000000
00000000000000000000000000000000000000000000000000000000000000000000000000000000000000000000000000000000000000000000000000000000000000000000000000000000000000000000000000000000
00000000000000000000000000000000
00000000000000000000000000000000000000000000000000000000000000000000000000000000000000000000000000000000000000000000000000000000000000000000000000000000000000000000000000000000
00000000000000000000000000000000000000000000000000000000000000000000000000000000000000000000000000000000000000000000000000000000000000000000000000000000000000000000000000000000
00000000000000000000000000000000
00000000000000000000000000000000000000000000000000000000000000000000000000000000000000000000000000000000000000000000000000000000000000000000000000000000000000000000000000000000
00000000000000000000000000000000000000000000000000000000000000000000000000000000000000000000000000000000000000000000000000000000000000000000000000000000000000000000000000000000
00000000000000000000000000000000
00000000000000000000000000000000000000000000000000000000000000000000000000000000000000000000000000000000000000000000000000000000000000000000000000000000000000000000000000000000
00000000000000000000000000000000000000000000000000000000000000000000000000000000000000000000000000000000000000000000000000000000000000000000000000000000000000000000000000000000
00000000000000000000000000000000
00000000000000000000000000000000000000000000000000000000000000000000000000000000000000000000000000000000000000000000000000000000000000000000000000000000000000000000000000000000
00000000000000000000000000000000000000000000000000000000000000000000000000000000000000000000000000000000000000000000000000000000000000000000000000000000000000000000000000000000
00000000000000000000000000000000
>DNA_Q9SYL8
MAEEQREISHENNVSLGSAETAIPLTNVSISPTKKEEQKTVYLVLFGRTIYVESSSLESEETVMPIVDVPKSPAKEDLTTHCSLDAESSEKGFPVDPYMGILGNNPNINSNVTCAGNKDLTEDIIKFKKMRSLSKEKIVEEKGTRVLTELPPYTWTIKKTLKESDINHQCRLLLN
TTDAENHIMRHLPTDDQKKIQEGIGVDVKAYDHDTYTEHDMVFKRHVKTSKSYVFNGGWAKAFVGRRELKEGDKIGLFWAKKSQTFMSQEQQGTSPLNTDEALKSHHSFSQNPNYIESNLSRRYQTNFSLGSQRISGQFLMIPNPGFVDTSSMTNTHDTSLANFQTVPTTTNPKI
ILKEEKENEEEQQYWTIKKELTKSDACQRLTLSKSSVEEHILKHLLPEDSQKIDKGKPGITVKVYDNDTDTEHELCLAFQCSYVLKNGWVKTFVKRRGLEEGDMIGLFWECSTSKLHFSVLFRVNAKAPAAEEKEAY
00000000000000000000000000000000000000000000000000000000000000000000000000000000000000000000000000000000000000000000000000000000000000000000000000000000000000000000000000000000
00000000000000000000000000000000000000000000000000000000000000000000000000000000000000000000000000000000000000000000000000000000000000000000000000000000000000000000000000000000
000000000000000000000000000000000000000000000000000000000000000000000000000000000000000000000000000000000000000000000000000000000000000000
00000000000000000000000000000000000000000000000000000000000000000000000000000000000000000000000000000000000000000000000000000000000000000000000000000000000000000000000000000000
00000000000000000000000000000000000000000000000000000000000000000000000000000000000000000000000000000000000000000000000000000000000000000000000000000000000000000000000000000000
000000000000000000000000000000000000000000000000000000000000000000000000000000000000000000000000000000000000000000000000000000000000000000
00000000000000000000000000000000000000000000000000000000000000000000000000000000000000000000000000000000000000000000000000000000000000000000000000000000000000000000000000000000
00000000000000000000000000000000000000000000000000000000000000000000000000000000000000000000000000000000000000000000000000000000000000000000000000000000000000000000000000000000
000000000000000000000000000000000000000000000000000000000000000000000000000000000000000000000000000000000000000000000000000000000000000000
00000000000000000000000000000000000000000000000000000000000000000000000000000000000000000000000000000000000000000000000000000000000000000000000000000000000000000000000000000000
00000000000000000000000000000000000000000000000000000000000000000000000000000000000000000000000000000000000000000000000000000000000000000000000000000000000000000000000000000000
000000000000000000000000000000000000000000000000000000000000000000000000000000000000000000000000000000000000000000000000000000000000000000
00000000000000000000000000000000000000000000000000000000000000000000000000000000000000000000000000000000000000000000000000000000000000000000000000000000000000000000000000000000
00000000000000000000000000000000000000000000000000000000000000000000000000000000000000000000000000000000000000000000000000000000000000000000000000000000000000000000000000000000
000000000000000000000000000000000000000000000000000000000000000000000000000000000000000000000000000000000000000000000000000000000000000000
00000000000000000000000000000000000000000000000000000000000000000000000000000000000000000000000000000000000000000000000000000000000000000000000000000000000000000000000000000000
00000000000000000000000000000000000000000000000000000000000000000000000000000000000000000000000000000000000000000000000000000000000000000000000000000000000000000000000000000000
000000000000000000000000000000000000000000000000000000000000000000000000000000000000000000000000000000000000000000000000000000000000000000
00000000000000000000000000000000000000000000000000000000000000000000000000000000000000000000000000000000000000000000000000000000000000000000000000000000000000000000000000000000
00000000000000000000000000000000000000000000000000000000000000000000000000000000000000000000000000000000000000000000000000000000000000000000000000000000000000000000000000000000
000000000000000000000000000000000000000000000000000000000000000000000000000000000000000000000000000000000000000000000000000000000000000000
>DNA_Q9T0D3
MPGEQTGETPTVAGVGGGGAGCSAGNSGGSSGCGAGGGGGGSGGGGGGGGDSQRSIPTPFLTKTYQLVEDPVYDELISWNEDGTTFIVWRPAEFARDLLPKYFKHNNFSSFVRQLNTYGFRKVVPDRWEFSNDCFKRGEKILLRDIQRRKISQPAMAAAAAAAAAAAVAASAVTVA
AVPVVAHIVSPSNSGEEQVISSNSSPAAAAAAIGGVVGGGSLQRTTSCTTAPELVEENERLRKDNERLRKEMTKLKGLYANIYTLMANFTPGQEDCAHLLPEGKPLDLLPERQEMSEAIMASEIETGIGLKLGEDLTPRLFGVSIGVKRARREEELGAAEEEDDDRREAAAQEGE
QSSDVKAEPMEENNSGNHNGSWLELGK
00000000000000000000000000000000000000000000000000000000000000000000000000000000000000000000000000000000000000000000000000000000000000000000000000000000000000000000000000000000
00000000000000000000000000000000000000000000000000000000000000000000000000000000000000000000000000000000000000000000000000000000000000000000000000000000000000000000000000000000
000000000000000000000000000

0000000000000000000000000000000000000000000000000000000000000000000000000000000000000000000000000000000000000000000000000000000000000000000000000000000000000000000000000000000
0000000000000000000000000000000000000000000000000000000000000000000000000000000000000000000000000000000000000000000000000000000000000000000000000000000000000000000000000000000
000000000000000000000000000
0000000000000000000000000000000000000000000000000000000000000000000000000000000000000000000000000000000000000000000000000000000000000000000000000000000000000000000000000000000
0000000000000000000000000000000000000000000000000000000000000000000000000000000000000000000000000000000000000000000000000000000000000000000000000000000000000000000000000000000
000000000000000000000000000
0000000000000000000000000000000000000000000000000000000000000000000000000000000000000000000000000000000000000000000000000000000000000000000000000000000000000000000000000000000
0000000000000000000000000000000000000000000000000000000000000000000000000000000000000000000000000000000000000000000000000000000000000000000000000000000000000000000000000000000
000000000000000000000000000
0000000000000000000000000000000000000000000000000000000000000000000000000000000000000000000000000000000000000000000000000000000000000000000000000000000000000000000000000000000
0000000000000000000000000000000000000000000000000000000000000000000000000000000000000000000000000000000000000000000000000000000000000000000000000000000000000000000000000000000
000000000000000000000000000
0000000000000000000000000000000000000000000000000000000000000000000000000000000000000000000000000000000000000000000000000000000000000000000000000000000000000000000000000000000
0000000000000000000000000000000000000000000000000000000000000000000000000000000000000000000000000000000000000000000000000000000000000000000000000000000000000000000000000000000
000000000000000000000000000
0000000000000000000000000000000000000000000000000000000000000000000000000000000000000000000000000000000000000000000000000000000000000000000000000000000000000000000000000000000
0000000000000000000000000000000000000000000000000000000000000000000000000000000000000000000000000000000000000000000000000000000000000000000000000000000000000000000000000000000
000000000000000000000000000
>DNA_Q9TZL0
MTSQYSNYSCPLNNIQVNVIGSSRTSDDHFLEQMLTKRKKTNISQDLHYLLLGLNPRELYQISFKLARIDEKKYKFNGNFNEGDYYPHKNSEIPMEDTEEIVHSDGYQTGARWMKTGVYFPKIGISKEQPDKERCLLLESLHAYFPVLVFTTSSGISLEVPVWSQKFVTTMTSSN
RSVKRREKRKLEETAGPAAKKTPDIIETTPEDVVPEVYNAQYFYQMQNNTVISSESKFSSPLALQQDLTEDFGTFAQLDAHFNVSVQSCSSSSLSSPAALKHDSTVSSDSDFDDKNSEEFDAVFEYIDQQFSNHDQNPMDQQSKSIDLNQTTWAPQSINNPGYLSTASSPAALNQ
DSSASEKSSIVRDKKSDENCLDEFDRVFNELDQQFTPKEDQVQDLSNCTTWNQEPINNSIQSNQFINPELFYFIFHKCLIISWPSCLSCCRGSILPQHLRTPPLVPISQLLIISPLVLPPYHNFTTGSATVSPFASTPANMTIFNFTSDSSTTYSNSTTAQVFPDVANTPDLSYF
NIAPAVDLSLFIDPFVQSGRDLSIERSIRNFTNTLKKAHRQKKTAILTFQSTEPMQLEFKYCTKMLLRTDKPVGNFSIQLSGPYLEHLLNLSHHLCRFSEYNLFVKAHFPRGSYGSLTSMTGPAVEIPETQENITIIWVKKDFPENVNKYPVAINSLITEAIASNGTNQVLLTSK
YPSDCTIRFRKCGFMIEGVLVVKKKETVIIDRQILGELNRLKHDLLQ
0000000000000000000000000000000000000000000000000000000000000000000000000000000000000000000000000000000000000000000000000000000000000000000000000000000000000000000000000000000
0000000000000000000000000000000000000000000000000000000000000000000000000000000000000000000000000000000000000000000000000000000000000000000000000000000000000000000000000000000
0000000000000000000000000000000000000000000000000000000000000000000000000000000000000000000000000000000000000000000000000000000000000000000000000000000000000000000000000000000
0000000000000000000000000000000000000000000000000000000000000000000000000000000000000000000000000000000000000000000000000000000000000000000000000000000000000000000000000000000
00000000000000000000000000000000000000000000000
0000000000000000000000000000000000000000000000000000000000000000000000000000000000000000000000000000000000000000000000000000000000000000000000000000000000000000000000000000000
0000000000000000000000000000000000000000000000000000000000000000000000000000000000000000000000000000000000000000000000000000000000000000000000000000000000000000000000000000000
0000000000000000000000000000000000000000000000000000000000000000000000000000000000000000000000000000000000000000000000000000000000000000000000000000000000000000000000000000000
0000000000000000000000000000000000000000000000000000000000000000000000000000000000000000000000000000000000000000000000000000000000000000000000000000000000000000000000000000000
00000000000000000000000000000000000000000000000
0000000000000000000000000000000000000000000000000000000000000000000000000000000000000000000000000000000000000000000000000000000000000000000000000000000000000000000000000000000
0000000000000000000000000000000000000000000000000000000000000000000000000000000000000000000000000000000000000000000000000000000000000000000000000000000000000000000000000000000
0000000000000000000000000000000000000000000000000000000000000000000000000000000000000000000000000000000000000000000000000000000000000000000000000000000000000000000000000000000
0000000000000000000000000000000000000000000000000000000000000000000000000000000000000000000000000000000000000000000000000000000000000000000000000000000000000000000000000000000
00000000000000000000000000000000000000000000000
0000000000000000000000000000000000000000000000000000000000000000000000000000000000000000000000000000000000000000000000000000000000000000000000000000000000000000000000000000000
0000000000000000000000000000000000000000000000000000000000000000000000000000000000000000000000000000000000000000000000000000000000000000000000000000000000000000000000000000000
0000000000000000000000000000000000000000000000000000000000000000000000000000000000000000000000000000000000000000000000000000000000000000000000000000000000000000000000000000000
0000000000000000000000000000000000000000000000000000000000000000000000000000000000000000000000000000000000000000000000000000000000000000000000000000000000000000000000000000000
00000000000000000000000000000000000000000000000
0000000000000000000000000000000000000000000000000000000000000000000000000000000000000000000000000000000000000000000000000000000000000000000000000000000000000000000000000000000
0000000000000000000000000000000000000000000000000000000000000000000000000000000000000000000000000000000000000000000000000000000000000000000000000000000000000000000000000000000
0000000000000000000000000000000000000000000000000000000000000000000000000000000000000000000000000000000000000000000000000000000000000000000000000000000000000000000000000000000
0000000000000000000000000000000000000000000000000000000000000000000000000000000000000000000000000000000000000000000000000000000000000000000000000000000000000000000000000000000
00000000000000000000000000000000000000000000000
0000000000000000000000000000000000000000000000000000000000000000000000000000000000000000000000000000000000000000000000000000000000000000000000000000000000000000000000000000000
0000000000000000000000000000000000000000000000000000000000000000000000000000000000000000000000000000000000000000000000000000000000000000000000000000000000000000000000000000000
0000000000000000000000000000000000000000000000000000000000000000000000000000000000000000000000000000000000000000000000000000000000000000000000000000000000000000000000000000000
0000000000000000000000000000000000000000000000000000000000000000000000000000000000000000000000000000000000000000000000000000000000000000000000000000000000000000000000000000000
00000000000000000000000000000000000000000000000
>DNA_Q9UUA2
MFSCQSLYKFSHSFRKRIPVMFQRAQQKSSLLHTQNESSHQPSLNKLGGFSSASLNFNSSRSSTNDDQQTFSSQSDNLPSSPITLPAKRGRSAASLKQLDNTVGFDVSKPSLPVFENSGLGSKYSTEIANGVYIDENDFDDDLLLENDIDQKPIPWSSSPIEHTKLTKSMLSSEK
RSKNHLSKIYEDHTSEKGASSVISSNITRQGIKRSRTLPWAVDPYRYGDPDPKRTSTSADISQHTVSNDSSNKLSNGRSSSLDSLAKKRMSKSKSTPQISKKFSVPLNSASKSPIGSSLFKTSDSRKKSVPSIFLSDEQKRILDMVVEQQHSIFFTGSAGTGKSVLLRKIIEVLK
SKYRKQSDRVAVTASTGLAACNIGGVTLHSFAGVGLARESVDLLVSKIKKNKKCVNRWLRTRVLIIDEVSMVDAELMDKLEEVARVIRKDSKPFGGIQLVLTGDFFQLPPVPENGKESKFCFESQTWKSALDFTIGLTHVFRQKDEEFVKMLNELRLGKLSDESVRKFKVLNRTI
EYEDGLLPTELFPTRYEVERSNDMRMQQINQNPVTFTAIDSGTVRDKEFRDRLLQGCMAPATLVLKVNAQVMLIKNIDDQLVNGSLGKVIGFIDDETYQMEKKDAEMQGRNAFEYDSLDISPFDLPDVKQKKYKLIAMRKASSTAIKWPLVRFKLPNGGERTIVVQRETWNIELP
NGEVQASRSQIPLILAYAISIHKAQGQTLDRVKVDLGRVFEKGQAYVALSRATTQEGLQVLNFSPAKVMAHPKVVQFYKQLASVNGLPIRNENKAPVQMRGVKNK
0000000000000000000000000000000000000000000000000000000000000000000000000000000000000000000000000000000000000000000000000000000000000000000000000000000000000000000000000000000
0000000000000000000000000000000000000000000000000000000000000000000000000000000000000000000000000000000000000000000000000000000000000000000000000000000000000000000000000000000
0000000000000000000000000000000000000000000000000000000000000000000000000000000000000000000000000000000000000000000000000000000000000000000000000000000000000000000000000000000

0000000000000000000000000000000000000000000000000000000000000000000000000000000000000000000000000000000000000000000000000000000000000000000000000000000000000000000000000000000
000000000000000000000000000000000000000000000000000000000000000000000000000000000000000000000000000000000
0000000000000000000000000000000000000000000000000000000000000000000000000000000000000000000000000000000000000000000000000000000000000000000000000000000000000000000000000000000
0000000000000000000000000000000000000000000000000000000000000000000000000000000000000000000000000000000000000000000000000000000000000000000000000000000000000000000000000000000
0000000000000000000000000000000000000000000000000000000000000000000000000000000000000000000000000000000000000000000000000000000000000000000000000000000000000000000000000000000
0000000000000000000000000000000000000000000000000000000000000000000000000000000000000000000000000000000000000000000000000000000000000000000000000000000000000000000000000000000
000000000000000000000000000000000000000000000000000000000000000000000000000000000000000000000000000000000
0000000000000000000000000000000000000000000000000000000000000000000000000000000000000000000000000000000000000000000000000000000000000000000000000000000000000000000000000000000
0000000000000000000000000000000000000000000000000000000000000000000000000000000000000000000000000000000000000000000000000000000000000000000000000000000000000000000000000000000
0000000000000000000000000000000000000000000000000000000000000000000000000000000000000000000000000000000000000000000000000000000000000000000000000000000000000000000000000000000
0000000000000000000000000000000000000000000000000000000000000000000000000000000000000000000000000000000000000000000000000000000000000000000000000000000000000000000000000000000
000000000000000000000000000000000000000000000000000000000000000000000000000000000000000000000000000000000
0000000000000000000000000000000000000000000000000000000000000000000000000000000000000000000000000000000000000000000000000000000000000000000000000000000000000000000000000000000
0000000000000000000000000000000000000000000000000000000000000000000000000000000000000000000000000000000000000000000000000000000000000000000000000000000000000000000000000000000
0000000000000000000000000000000000000000000000000000000000000000000000000000000000000000000000000000000000000000000000000000000000000000000000000000000000000000000000000000000
0000000000000000000000000000000000000000000000000000000000000000000000000000000000000000000000000000000000000000000000000000000000000000000000000000000000000000000000000000000
000000000000000000000000000000000000000000000000000000000000000000000000000000000000000000000000000000000
0000000000000000000000000000000000000000000000000000000000000000000000000000000000000000000000000000000000000000000000000000000000000000000000000000000000000000000000000000000
0000000000000000000000000000000000000000000000000000000000000000000000000000000000000000000000000000000000000000000000000000000000000000000000000000000000000000000000000000000
0000000000000000000000000000000000000000000000000000000000000000000000000000000000000000000000000000000000000000000000000000000000000000000000000000000000000000000000000000000
0000000000000000000000000000000000000000000000000000000000000000000000000000000000000000000000000000000000000000000000000000000000000000000000000000000000000000000000000000000
000000000000000000000000000000000000000000000000000000000000000000000000000000000000000000000000000000000
0000000000000000000000000000000000000000000000000000000000000000000000000000000000000000000000000000000000000000000000000000000000000000000000000000000000000000000000000000000
0000000000000000000000000000000000000000000000000000000000000000000000000000000000000000000000000000000000000000000000000000000000000000000000000000000000000000000000000000000
0000000000000000000000000000000000000000000000000000000000000000000000000000000000000000000000000000000000000000000000000000000000000000000000000000000000000000000000000000000
0000000000000000000000000000000000000000000000000000000000000000000000000000000000000000000000000000000000000000000000000000000000000000000000000000000000000000000000000000000
000000000000000000000000000000000000000000000000000000000000000000000000000000000000000000000000000000000
0000000000000000000000000000000000000000000000000000000000000000000000000000000000000000000000000000000000000000000000000000000000000000000000000000000000000000000000000000000
0000000000000000000000000000000000000000000000000000000000000000000000000000000000000000000000000000000000000000000000000000000000000000000000000000000000000000000000000000000
0000000000000000000000000000000000000000000000000000000000000000000000000000000000000000000000000000000000000000000000000000000000000000000000000000000000000000000000000000000
0000000000000000000000000000000000000000000000000000000000000000000000000000000000000000000000000000000000000000000000000000000000000000000000000000000000000000000000000000000
000000000000000000000000000000000000000000000000000000000000000000000000000000000000000000000000000000000
>DNA_Q9XYB7
MPSSSPSMDESRRSAVPPKEPAGRICTVCSDRANGYNFGVLTCESCKAFFRRNASKHKEIKCPFSDSCQITSASRKFCQACRLNKCFAVGMNSEWLNDLKPKSSIVSGKFKRKKPDMKNNLKVEVDDTEEDLENDDEEQISVPKALLEKLINKANEKSKDRCTCKCQCGFYPITQ
RLTAYEPKDTTAVNSPQDISFSHHLHHSDSFYSSSTSTLSPMSVISCAPSSHDSSGYNTSQLVTQSPTNYTVSPASIPSSITELSPQMPSQYPPMLSPFQFGVMAQMAAPANFLNFPPMPERTWTPIQAVSTVPVTETLPPNLLEQIHSKIDKYIGVLNEDEITLLEELHVQNEP
LNAPLIQWHNPKSIDGVFRIIEEALRRIVNMACQLSLFRELHVDDRKNLLKSGFGELLIVRGLMAYDKSDNSWNHSFGVRGKMEVKVEVLKNPKLEEHYKAHMNLLSTFGEDVRNNEHLMLIFNAAVIFHPHVSNLRDSKRVHSTQAKYFQMLLKLLTFEYGKSRADIAYSNLLN
QVVELHRVNRTLLRVFYGLDIAQLDPLIRELCSFE
0000000000000000000000000000000000000000000000000000000000000000000000000000000000000000000000000000000000000000000000000000000000000000000000000000000000000000000000000000000
0000000000000000000000000000000000000000000000000000000000000000000000000000000000000000000000000000000000000000000000000000000000000000000000000000000000000000000000000000000
0000000000000000000000000000000000000000000000000000000000000000000000000000000000000000000000000000000000000000000000000000000000000000000000000000000000000000000000000000000
00000000000000000000000000000000000
0000000000000000000000000000000000000000000000000000000000000000000000000000000000000000000000000000000000000000000000000000000000000000000000000000000000000000000000000000000
0000000000000000000000000000000000000000000000000000000000000000000000000000000000000000000000000000000000000000000000000000000000000000000000000000000000000000000000000000000
0000000000000000000000000000000000000000000000000000000000000000000000000000000000000000000000000000000000000000000000000000000000000000000000000000000000000000000000000000000
00000000000000000000000000000000000
0000000000000000000000000000000000000000000000000000000000000000000000000000000000000000000000000000000000000000000000000000000000000000000000000000000000000000000000000000000
0000000000000000000000000000000000000000000000000000000000000000000000000000000000000000000000000000000000000000000000000000000000000000000000000000000000000000000000000000000
0000000000000000000000000000000000000000000000000000000000000000000000000000000000000000000000000000000000000000000000000000000000000000000000000000000000000000000000000000000
00000000000000000000000000000000000
0000000000000000000000000000000000000000000000000000000000000000000000000000000000000000000000000000000000000000000000000000000000000000000000000000000000000000000000000000000
0000000000000000000000000000000000000000000000000000000000000000000000000000000000000000000000000000000000000000000000000000000000000000000000000000000000000000000000000000000
0000000000000000000000000000000000000000000000000000000000000000000000000000000000000000000000000000000000000000000000000000000000000000000000000000000000000000000000000000000
00000000000000000000000000000000000
0000000000000000000000000000000000000000000000000000000000000000000000000000000000000000000000000000000000000000000000000000000000000000000000000000000000000000000000000000000
0000000000000000000000000000000000000000000000000000000000000000000000000000000000000000000000000000000000000000000000000000000000000000000000000000000000000000000000000000000
0000000000000000000000000000000000000000000000000000000000000000000000000000000000000000000000000000000000000000000000000000000000000000000000000000000000000000000000000000000
00000000000000000000000000000000000
0000000000000000000000000000000000000000000000000000000000000000000000000000000000000000000000000000000000000000000000000000000000000000000000000000000000000000000000000000000
0000000000000000000000000000000000000000000000000000000000000000000000000000000000000000000000000000000000000000000000000000000000000000000000000000000000000000000000000000000
0000000000000000000000000000000000000000000000000000000000000000000000000000000000000000000000000000000000000000000000000000000000000000000000000000000000000000000000000000000
00000000000000000000000000000000000
0000000000000000000000000000000000000000000000000000000000000000000000000000000000000000000000000000000000000000000000000000000000000000000000000000000000000000000000000000000
0000000000000000000000000000000000000000000000000000000000000000000000000000000000000000000000000000000000000000000000000000000000000000000000000000000000000000000000000000000
0000000000000000000000000000000000000000000000000000000000000000000000000000000000000000000000000000000000000000000000000000000000000000000000000000000000000000000000000000000
00000000000000000000000000000000000
